# A database on differentially expressed microRNAs during rodent bladder healing

**DOI:** 10.1038/s41598-021-01413-0

**Published:** 2021-11-08

**Authors:** Clara Ibel Chamorro, Jesper Eisfeldt, Oliver Willacy, Nikolai Juul, Magdalena Fossum

**Affiliations:** 1 Laboratory of Tissue Engineering, Department of Women’s and Children’s Health, Bioclinicum, Karolinska Institutet, Stockholm, Sweden; 2 Department of Pediatric Surgery, Copenhagen University Hospital, Copenhagen, Denmark; 3 Laboratory of Tissue Engineering, Department of Health Sciences, University of Copenhagen, Copenhagen, Denmark; 4 Department of Molecular Medicine and Surgery, Karolinska Institutet, Stockholm, Sweden; 5 Department of Clinical Genetics, Karolinska University Hospital, Stockholm, Sweden

**Keywords:** Cell biology, Computational biology and bioinformatics, Molecular medicine, Biomedical engineering, Urology, Bladder

## Abstract

Urinary bladder wound healing relies on multiple biological events that are finely tuned in a spatial–temporal manner. MicroRNAs are small non-coding RNA molecules with regulatory functions. We hypothesized that microRNAs are important molecules in the coordination of normal urinary bladder wound healing. We aimed at identifying microRNAs expressed during bladder wound healing using Affymetrix global array for microRNA profiling of the rodent urinary bladder during healing of a surgically created wound. Results were validated in the rat bladders by real-time PCR (RT-PCR) using three of the differentially expressed (DE) microRNAs. The model was thereafter validated in human cells, by measuring the expression of eight of the DE microRNAs upon in vitro wound-healing assays in primary urothelial cells. Our results indicated that 508 (40%) of all rodent microRNAs were expressed in the urinary bladder during wound healing. Thirteen of these microRNAs (1%) were DE (false discovery rate (FDR) < 0.05, P < 0.05, |logfold|> 0.25) in wounded compared to non-wounded bladders. Bioinformatic analyses helped us to identify target molecules for the DE microRNAs, and biological pathways involved in tissue repair. All data are made available in an open-access database for other researchers to explore.

## Introduction

Engineered cell-seeded grafts have been introduced to treat several urogenital disorders^1,2^. The final outcome of any transplanted tissue or engineered graft greatly depends on the host wound healing response. Wound healing in adult tissue is a multistep process comprising overlapping cellular responses including hemostasis, inflammation, cellular proliferation, extracellular matrix synthesis and remodeling. In a normal wound, once the hemostasis and inflammation steps have ensued, proliferation and cellular migration take over and replace the damaged tissue. Eventually, the proliferation ceases and the tissue matures with scar formation.

In the rodent urinary bladder, the healing process is highly similar to what has been observed in the skin, at both a morphological and a molecular level^3,4^. Due to their reliability and reproducibility, rodent models are frequently used in biomedical research^5^. In comparison to the human genome, the rat genome encodes a similar number of protein-coding genes (22,000 for rats, 20,000 for humans)^5,6^. Molecular and developmental processes in rodents are well characterized and share great similarities with humans^7^. Similarly, healing and trauma models in rats have proven translationally valuable for human wound healing and trauma research^8–11^.

The knowledge about the molecular mechanisms that coordinate bladder healing may be highly relevant to the clinical practice. It may help us understand conditions, such as chronic cystitis and post-irradiation cystitis, and it may improve the surgical treatments of congenital conditions, such as bladder exstrophy. Further understanding of micro-molecular events may unlock the development of pharmaceuticals for treating the diseased urinary bladder.

The non-coding small RNA molecules (19–23 nucleotides long), known as microRNAs, are important players during tissue homeostasis. These molecules act as epigenetic regulators of gene expression at the post-transcriptional level^12^. Mature microRNA molecules can target the degradation of complementary messenger RNAs (mRNA) or by inhibiting the interaction between mRNA and the translational machinery (down-regulating control)^13^. MicroRNAs interact mainly with the 3’untranslated region of target mRNAs, but the molecules can also interact with other coding and promoter regions of the genes^12^. A single microRNA can target hundreds of mRNAs, and a single gene can also be targeted by different microRNAs^14^.

MicroRNAs have been intensively studied and today we understand that these molecules regulate several cellular and developmental pathways. MicroRNAs have shown differing expression patterns during tissue repair in bones^15,16^, lungs^17^ skin^18,19^ and oral mucosa^20^. These molecules mostly contribute to tissue repair by coordinating cell proliferation and inflammation among other functions^18,19,21,22^. Today, only a few studies address the molecular signatures during tissue repair of the urinary bladder in a comprehensive way, and the contribution of microRNAs in normal bladder healing has, to our knowledge, not been explored^3,23^.

In our previous study, we histologically evaluated the wound healing pattern in the rat urinary bladder and characterized the expression of 84 mRNA wound healing related genes^3^.

In this study, we hypothesized, that we could expand our understanding of the regulatory networks governing urinary bladder healing, by identifying differentially expressed microRNAs in vivo and validating the findings in human bladder healing models in vitro*.* In doing so, we aimed at generating a large pool of bioinformatic data, and to make it available for other researchers in order to inspire and stimulate further investigations. We identified several potential microRNA markers and targets in the data set and other researchers within the field are encouraged to continue its exploration.

## Results

### MicroRNA molecular changes

**Figure 1:**
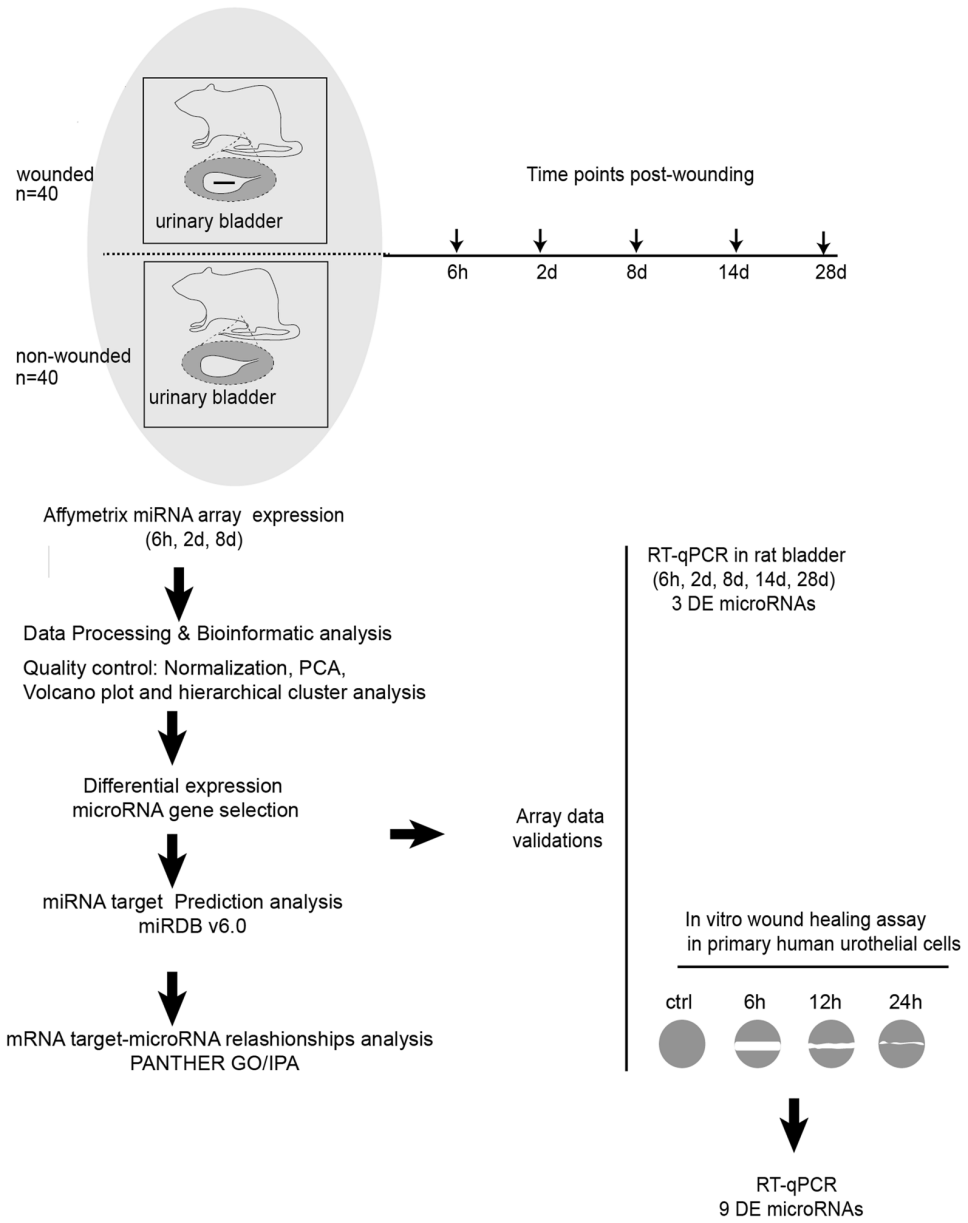
Cartoon describing the algorithm of the experimental design. A set of male rats received one-centimeter-long longitudinal incisions through all the layers of the urinary bladder wall. The bladder tissue was harvested at 6 h, 2 days, 8 days, 14 days and 28 days post-injury. A parallel set of sham operated rats were used as a control. DE microRNA genes were captured with Affymetrix array using samples form 0 h, 6 h, 2 days and 8 days post injury. Samples from all the time points were included for real time PCR validations in the rat bladder tissues. Relevance of the results was also validated in in vitro wound healing assays in human primary urothelial cells. The predicted target genes associated to the DE microRNAs and the associated biological functions associated were obtained through bioinformatic analysis.

We generated a microarray data set using rat bladder tissue at three different time points post wounding (Fig. 1).

**Figure 2:**
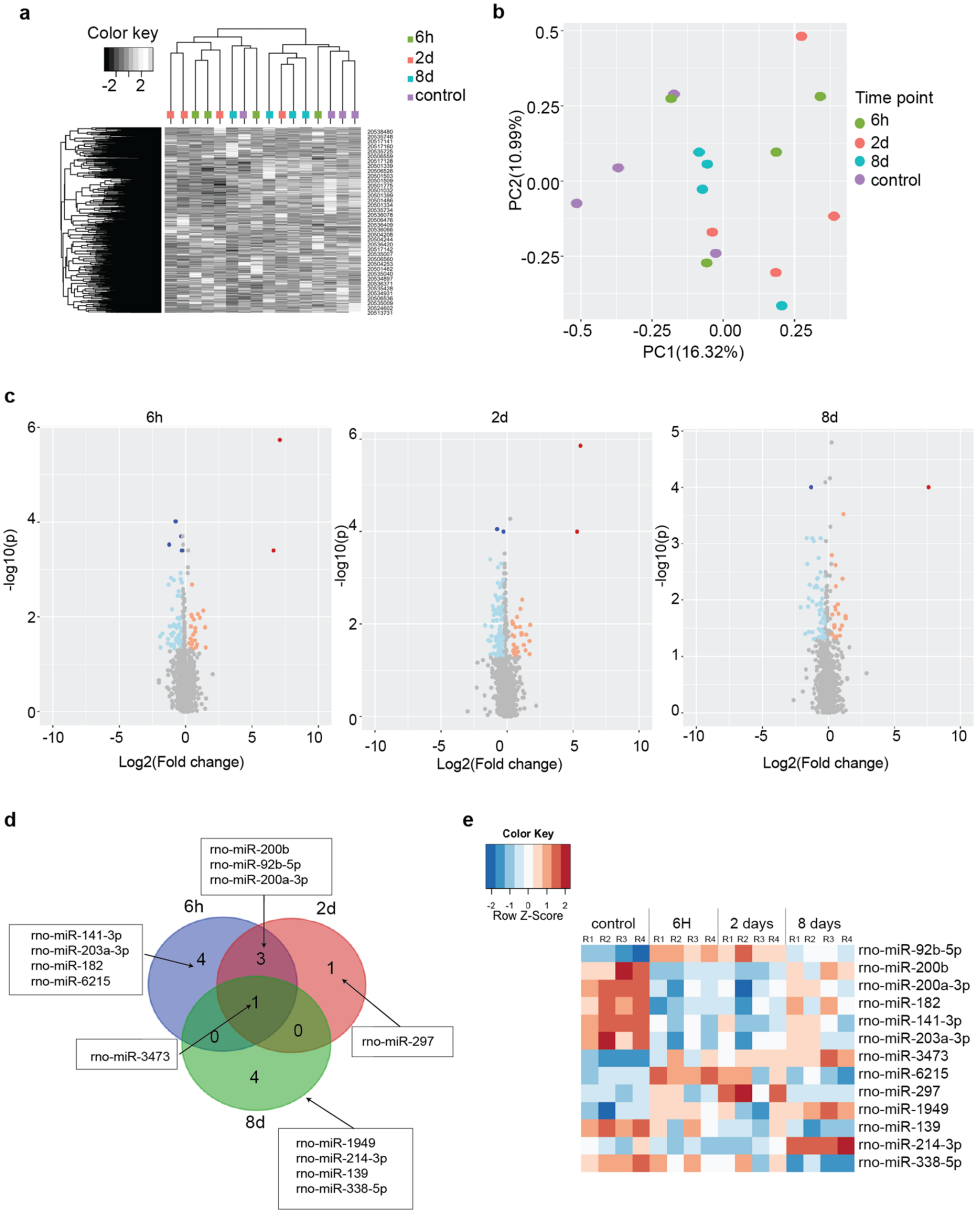
MicroRNA changes during bladder healing. (**a**). Heat map visualizing the expression levels of all genes for all replicates and conditions; the dendrograms indicate the results of hierarchical clustering. (**b**) PCA plot illustrating the microRNA expression landscape. (**c**) Volcano plots showing the statistical significance (p value) versus magnitude of change (fold change). Each point in the volcanos represents a microRNA transcript. Transcripts at the upper left and upper right corners are most likely to be differentially expressed. (**d**) Venn diagram illustrating the overlap in differentially expressed microRNA genes at 6 h, 2 days and 8 days post wounding. Thresholding was performed using a p value of 0,05 and FDR 0,05. e. Heat maps summarizing the expression of DE microRNAs across all the time points analyzed.

Significant differential gene expressions were observed between control and wounded bladders at every time point analyzed: 6 h, 2 days, and 8 days (Fig. 2).

The global microRNA expression patterns were studied further using a heatmap and hierarchical clustering, revealing similar results as the principal component analysis (PCA) (Fig. 2a,b). In addition to identifying the DE microRNAs, we analyzed the 40 highest expressed microRNAs (supplementary fig. S1). These top microRNAs targeted roughly 5000 genes and differences between the expressions were not significant (supplementary table S1). The Gene Ontology terms (GO) terms related to the target genes indicated relevant functions such as “wound healing” (GO:0042060), “morphogenesis of an epithelial sheet” (GO:0002011) and “regulation of endothelial cell differentiation” (GO:0045601) (supplementary table S2).

PCA was performed to assess the overall microRNA expression patterns (Fig. 2b), indicating clear grouping based on timepoints. However, there was a considerable variation between replicates, indicating variation on an individual level. Notably, the first principal component (explaining 16% of the total variance) had biological interpretation and was responsible for separating the controls from the wounded individuals. The most significant changes occurred during the early phase of the healing process, mostly at 6 h and 2 days, while at 8 days, fewer DE genes were encountered (Fig. 2c).

In total, 133 DE microRNA genes were observed at 6 h, 153 at 2 days and 99 at 8 days (p < 0.05). FDR corrected p-values narrowed down the list to 9, 5 and 5 after 6 h, 2 days and 8 days, respectively. Altogether, 13 DE microRNAs passed our strength criteria for selection (FDR < 0.05, P < 0.05, |logfold|> 0.25) (Fig. 2d,e and Table 1).

**Table 1:** Literature review on the known functions of **thirteen** DE microRNAs in tissue repair, cell proliferation, apoptosis, and cancer progression.

*MicroRNA ID*	Known functions in tissue repair, cell proliferation, apoptosis, and cancer progression	Please see supplementary reference material
rno-miR-92b-5p	Inhibits cell proliferation and migration of human primary keratinocytes Has decreased expression during cutaneous wound healing Promotes migration of bladder cancer cells	(1–4)
rno-miR-139	Influences the phenotypic tonicity in smooth muscle cells Suppresses cell proliferation and promotes cell apoptosis Might be an attractive cancer biomarker	(5–9)
rno-miR-141-3p	Apoptosis related microRNA Inhibits fibroblast proliferation and migration Elevated values in bladder and prostate cancer Inhibits vascular smooth muscle cell proliferation and migration	(10–13)
rno-miR-182	Smooth muscle cell phenotypic modulator	(14)
rno-miR-1949	Translational regulation of retinoblastoma-1 and in subsequent bladder tumorigenesis following spinal cord injury	(15**–**17)
rno-miR-200a-3p	Epithelial to mesenchymal transition Regulation of corneal epithelial cell migration during wound healing	(18–19)
rno-miR-200b-3p	Epithelial to mesenchymal transition Regulator of angiogenesis Inhibition of miR-200b improves wound healing in diabetic mice	(18, 20–22)
rno-miR-203a-3p	Increases keratinocyte differentiation Promotes cell cycle exit Pro-migratory factor in cutaneous wound healing Suppresses hepatic fibrosis	(22–24)
rno-miR-214-3p	Delayed fracture healing Skeletal muscle development Adipogenesis Regulator of lung fibroblast/myofibroblasts differentiation Cardiac fibrosis Potential urinary biomarker of bladder cancer Mediates skeletal muscle myogenesis and vascular smooth muscle cell proliferation, migration, and differentiation	(25–31)
rno-miR-297	No previously known function in wound healing but a protective role during inflammation and apoptosis	(32)
rno-miR-338-5p	Promotes proliferation, invasion and inflammatory reactions in fibroblasts Myelinogenesis and promotes Myelin Repair	(33–35)
rno-miR-3473	Potential candidate as a biomarker in kidney injury Dysregulated upon kidney injury Involved in inflammatory responses	(36–38)
rno-miR-6215	Unknown function Expressed in rat kidney	(39- 40)

Significantly DE microRNAs at 6 h, 2 days, and 8 days post wounding (with corresponding fold change, p-values, and false discovery rates) have been compiled in the supplementary tables S3-5.

### Target prediction and biological pathway enrichment

**Figure 3:**
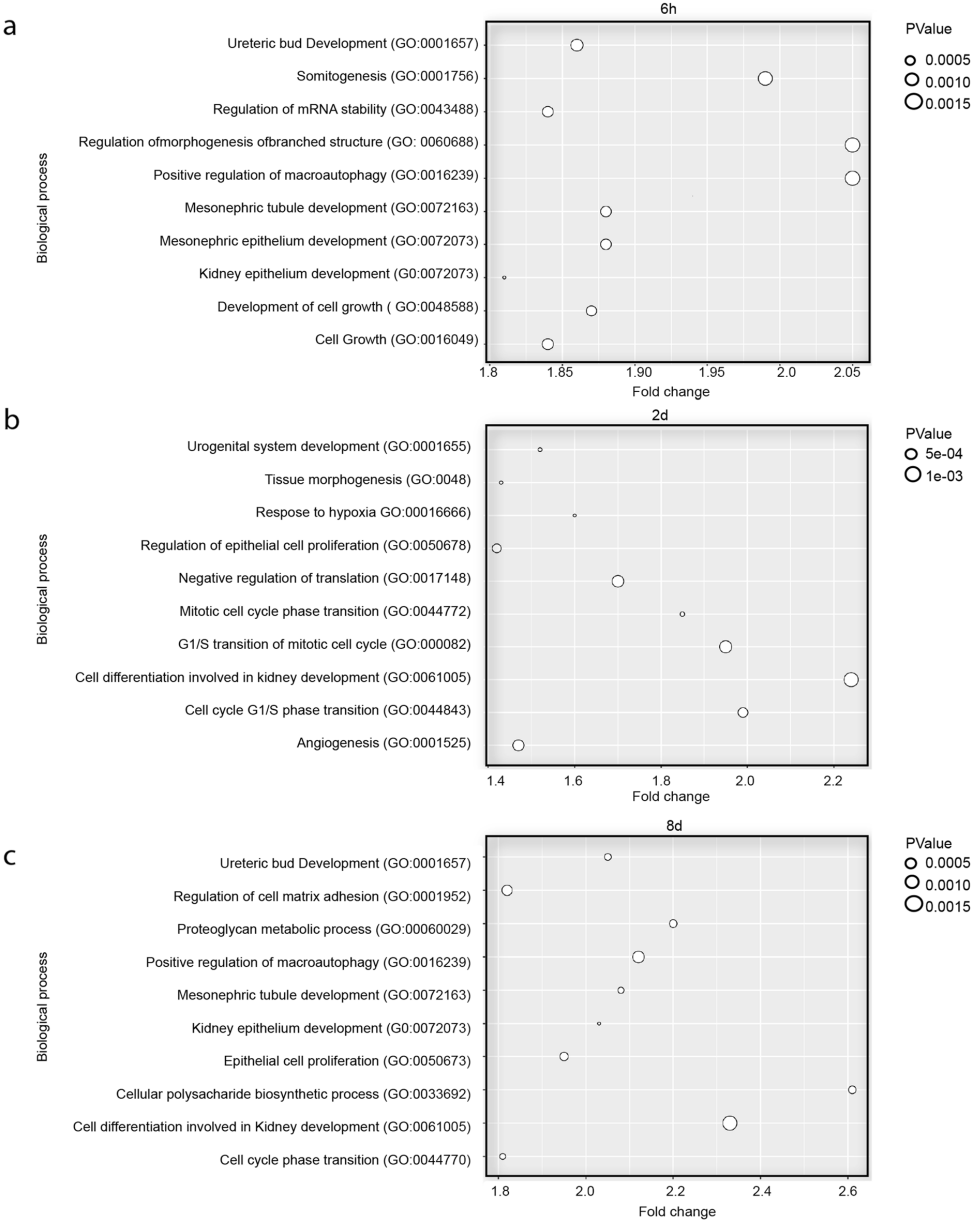
GO terms enrichment analysis. The graphs represent 10 different selected GO categories a). 6 h b). 2 days c). and 8 days post wounding. Both significance of the results and fold change are illustrated.

Using the online database for predicted microRNA targets: miRDB v6.0, we identified hundreds of mRNA coding genes that could be targeted for most of the DE microRNAs at the three time points examined (Table 2). Next, we evaluated the overlap between the predicted mRNA target with our previously reported DE mRNA during bladder healing and identified pairs of microRNA/mRNAs for further hypothesis generation and validation^3^ (Table 3). Furthermore, functional GO enrichment analysis on the predicted DE microRNA targets revealed several biological functions and processes relevant for tissue repair, such as epithelial migration, proliferation, stress response, differentiation, and angiogenesis (Fig. 3). The complete lists of all enriched Panther-GO terms at each time point in this study have been included in supplementary tables S6-8. We next performed an enrichment pathway analysis with the target genes for the DE microRNAs using Reactome, a curated data base of pathways and reactions in human biology^24^. Relevant biological pathways were enriched in our set of data. At 6 h and 2 days, signaling pathways, such as PI3k/AKT and ERK, which are involved in proliferation, migration and angiogenesis^25–27^, were represented (supplementary table S9). Furthermore, the E2F family of transcription factors, which regulates the transition from G1 to the S phase of the cell cycle, were significantly represented in the group of genes at day 2 (supplementary table S10). Another relevant biological pathway represented at days 2 and 8 was the nuclear receptor transcription signal, which is a key molecule in the regulation of cutaneous healing^28^ (supplementary table S10). Furthermore, a family member of the RUNX transcription factors, Runx1, which was found to regulate EMT marker genes and renal fibrosis, was among the most interesting biological pathways at day 8 (supplementary table S11)^29^.

**Table 2:** Total number of predicted targets for each of the DE microRNAs.

MicroRNA ID	Number of potential mRNA targets
Rno-miR-92b-5p	47
Rno-miR-139	373
Rno-miR-141-3p	522
Rno-miR-182	522
Rno-miR-1949	146
Rno-miR-200a-3p	522
Rno-miR-200b-3p	560
Rno-miR-203a-3p	523
Rno-miR-214-3p	533
Rno-miR-297	238
Rno-miR-338-5p	465
Rno-miR-3473	118
Rno-miR-6215	117

The gene targets of these microRNA were determined using the Rattus Norvegicus microRNA targets as listed in miRDB v6.0.

**Table 3:** Identification of potential relationships between DE microRNAs and DE mRNA during urinary bladder wound healing.

6 h			
microRNA	Target gene symbol	Molecule type	microRNA/mRNA expression values
rno-miR-200b	FGF7 RHOA VEGFA	Growth factor Enzyme Growth factor	−/ + −/ + −/ +
rno-miR-200a-3p	CTSV	Peptidase	−/ +
rno-miR-141-3p	CSF3 CTNNB1 CXCL2 FGA HGF ITGA6	Cytokine Transcription regulator Cytokine Other Growth factor Transmembrane Receptor	−/ + −/ + −/ + −/ + −/ + −/ +
rno-miR-182	F13A1 FGF10 FGF7 HBEGF RAC1 WISP1	Enzyme Growth factor Growth factor Growth factor Enzyme Other	−/ + −/ + −/ + −/ + −/ +
2 days			
rno-miR-92b-5p	MAPK3	Kinase	+ /−
rno-miR-182	F13A1 FGF10 FGF7 HBEGF ITGA1 ITGB6 RAC1 WISP1	Enzyme Growth factor Growth factor Growth factor Receptor Receptor Enzyme Other	−/ + −/− −/− −/ + −/− −/− −/− −/ +
rno-miR-200b	FGF7 RHOA VEGFA PTEN	Growth factor Enzyme Growth factor Phosphatase	−/− −/− −/−
rno-miR-200a-5p	CTSV	Peptidase	−/ +
rno-miR-297a-5p	MMP9 VEGFA	Peptidase Growth factor	+ / + + /−
8 days			
rno-miR-92b-5p	MAPK3	Kinase	+ / +
rno-miR-139	CSF3 SERPINE	Cytokine other	−/ + −/ +
rno-miR-214-3p	CTNNB1 MAPK1 MAPK3 PTEN WISP1	Transcription regulator Kinase Kinase Phosphatase other	−/ + −/ + −/ + −/ + −/ +
rno-miR-338-5p	CTGF IL6	Growth factor cytokine	−/ + −/ +

The list of DE microRNAs and the list of wound healing related DE mRNA in the rat experimental study were analyzed using the microRNA filter tool of Qiagen’s Ingenuity Pathway Analysis Software. In this analysis, we used mRNA expression data from 84 wound healing related genes from our previously published study and matched them with the microRNA expression data of our current microRNA expression study, at the respective time points^3^. The microRNA/mRNA expressions at each time point are showed (−) for down-regulation and ( +) for up-regulation.

### Validation of the microRNA array by RT-PCR

**Figure 4:**
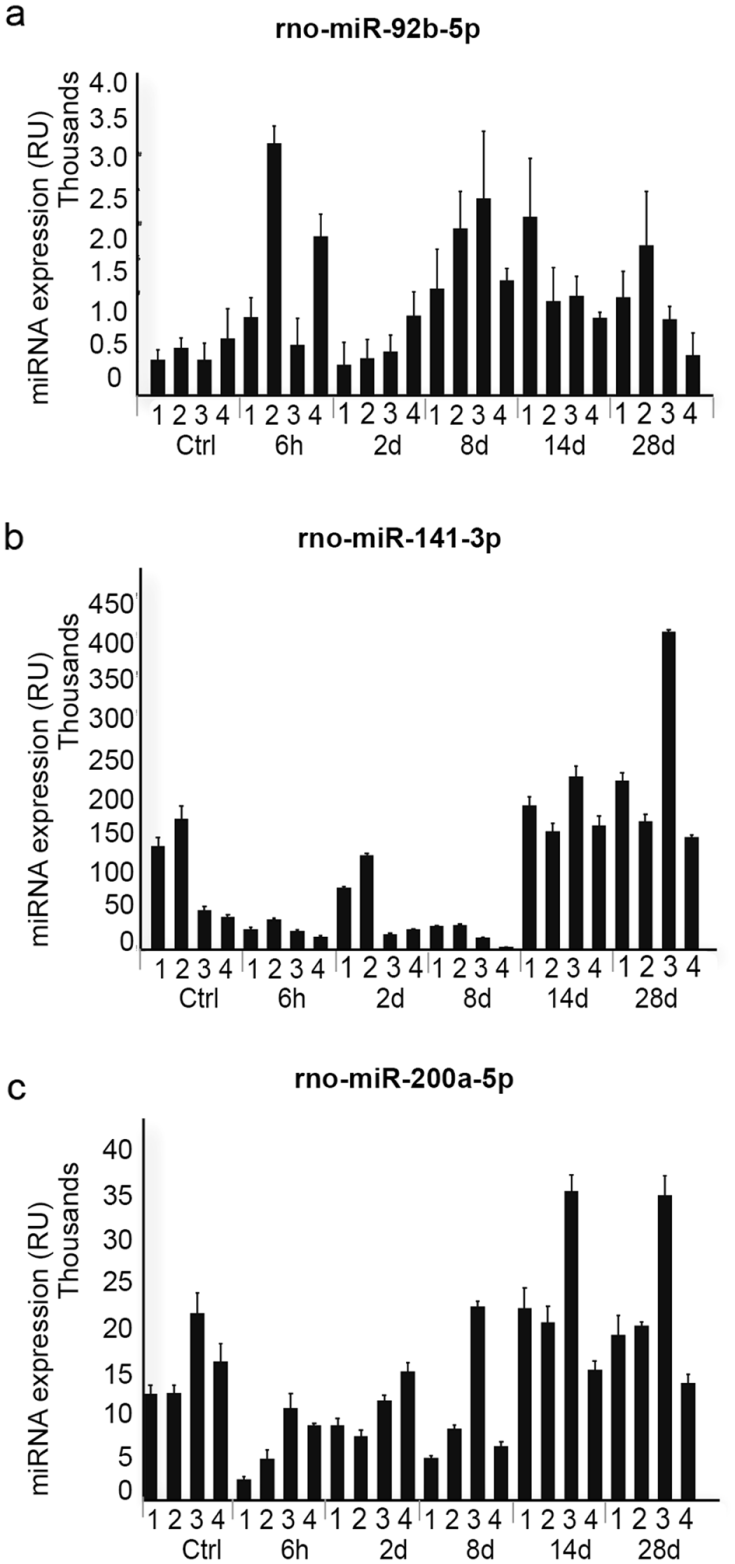
RT-PCR validation of 3 of DE genes in rat urinary bladder healing. (**a**-**c**) Real time quantitative PCR from bladder samples at all the time points included in the study, demostrating good consistency with the array (**a**) rno-miR-92b-5p (**b**) rnomiR141-3p and (**c**) rnomiR-200a-5p. The bar in the graphs shows the normalized average expression and standard deviations in every biological replicates (n = 4).

The expressions of three of the DE microRNAs were validated in rat bladder tissue and were consistent with the Affymetrix array results. We validated rno-miR-92b-5p, which had the highest up-regulation and two microRNAs with the most down-regulated patterns: rno-miR-141-3p, and rno-miR-200a-3p (Fig. 4).

**Figure 5:**
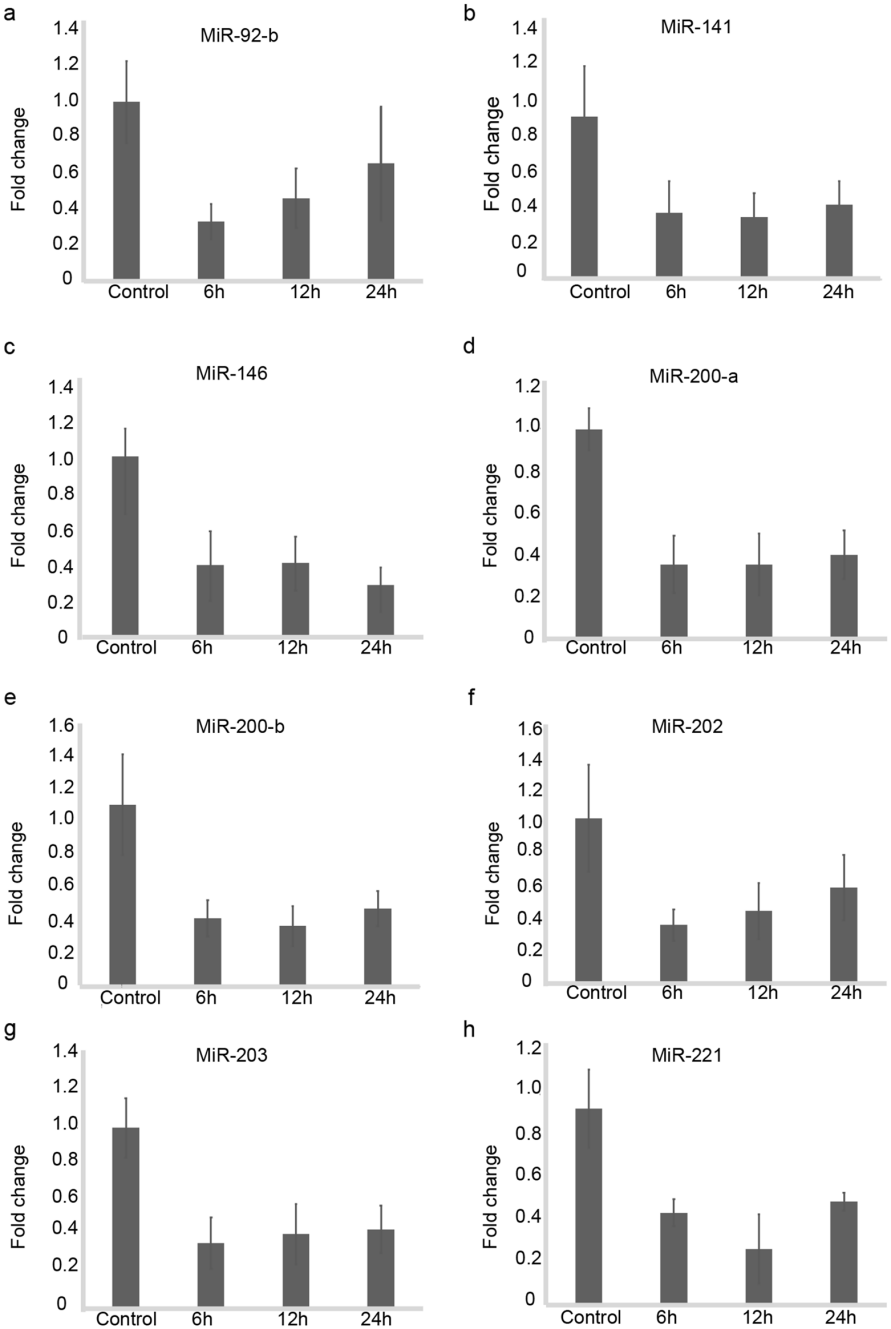
Validation of selected DE microRNAs in human primary urothelial cells upon in vitro wounding. Human primary urothelial cells were in vitro wounded, and the levels of 9 microRNAs were measured by RT-PCR at 6, 12 and 24 h post wounding. Bars represent average fold changes with their standard deviations in wounded urothelial cells at each timepoint and controls (non-wounded urothelial cells). (**a**) RT-PCR for miR-92-b (**b**) RT-PCR for miR-141 (**c**)  RT-PCR for miR-146 (**d**) RT-PCR for miR-200-a (**e**) RT-PCR for miR-200b (**f**) RT-PCR for miR-202 (**g**) RT-PCR for miR-203 (**h**) RT-PCR for miR-92-bRT-PCR for miR-202. One representative experiment is shown.

The relevance of 8 of the DE (Table 4) microRNAs in human cells was confirmed by analyzing their expression in primary human urothelial cells upon in vitro wounding (Fig. 5) at 6, 12 and at 24 h post wounding. Three of these microRNAs were chosen for validation due to their role in wound healing in other tissues (miR202, miR-221 and miR141).

**Table 4:** Information on TaqMan microRNA assays used in this study for array validation in rat bladder (rno) wound healing and human (hsa) urothelial cells upon in vitro scratch wounding.

TaqMan MicroRNA assay	Mature microRNA Sequence	Assay ID
rno-miR-141-3p	UAACACUGUCUGGUAAAGAUGG	000463
rno-miR-200a-5p	UAACACUGUCUGGUAACGAUGU	002274
rno-miR-92b-5p	AGGGACGGGACGCGGUGCAGUGUU	463385
RNU48	GATGACCCCAGGTAACTCTGAGTGTGTCGCTGATGCCATCACCGCAGCGCTCTGACC	001006
U6 snRNA	GTGCTCGCTTCGGCAGCACATATACTAAAATTGGAACGATACAGAGAAGATTAGCATGGCCCCTGCGCAAGGATGACACGCAAATTCGTGAAGCGTTCCATATTTT	001973
hsa-miR-92b	AGGGACGGGACGCGGUGCAGUG	002343
hsa-mi**R**-141	CAUCUUCCAGUACAGUGUUGGA	000463
hsa-miR-146a	UGAGAACUGAAUUCCAUGGGUU	000468
hsa-miR-200a	UAACACUGUCUGGUAACGAUGU	000502
hsa-miR-200b	UAAUACUGCCUGGUAAUGAUGA	002251
hsa-miR-202	AGAGGUAUAGGGCAUGGGAA	002363
hsa-miR-203	GUGAAAUGUUUAGGACCACUAG	000507
hsa-miR-221	AGCUACAUUGUCUGCUGGGUUUC	000524
hsa-miR-338	AACAAUAUCCUGGUGCUGAGUG	002658

## Discussion

While the role of microRNAs in skin wound healing has been extensively studied, their role in urinary bladder healing has, to our knowledge, not been previously explored. We therefore investigated the transcriptomic changes in microRNAs during normal bladder wound healing in a rodent model and validated the results with human cells**.**

By using Affymetrix microRNA array technology and a rodent model, we identified the microRNA expression profile at 6 h, 2 days, and 8 days post-wounding. These time points were chosen based on our previous rodent bladder wound healing study and selected to represent the three different stages of wound healing (inflammation, proliferation, and early tissue remodeling)^3^. We previously validated that these timepoints capture each specific stage histologically and at the mRNA level, however, some late-stage remodeling features, such as fibrosis, are likely underrepresented at day 8.

The total number of DE microRNAs was limited to nine, five and five microRNAs at 6 h, 2 days, and 8 days, respectively, corresponding to a total of 13 unique microRNAs (rno-miR-92b-5p, rno-miR-139, rno-miR-141-3p, rno-miR-182, rno-miR-1949, rno-miR-200a-3p, rno-miR-200b-3p, rno-miR-203a-3p, rno-miR-214-3p, rno-miR-297, rno-miR-338-5p, rno-miR-3473, rno-miR-6215).

Microarray technology was used in this study because it offers a comprehensive coverage of all known mature microRNA sequences in the miRbase, with an easy analysis flow and a small sample input. New technologies, such as RNA sequencing, could potentially be used in the future to discover new microRNA molecules that are currently unknown.

At the selected three time points, we saw a general pattern of microRNA down-regulation with only a few up-regulated microRNAs. A physiological explanation could be that most of the DE microRNAs are suppressors of wound healing and are turned off as a response to injury to allow a healing response.

In a review of the literature on known functions of the DE microRNAs we found that some of these microRNAs participate in wound healing in other tissues such as skin and bone tissue. Two examples are the miR-200 family members and miR-203. The miR-200 family consists of epithelium-specific microRNAs, acting as negative regulators of epithelial-mesenchymal transition, and also regulates keratinocyte migration, proliferation and differentiation^30^. MiR-203 is expressed in skin and other squamous epithelia and is described as an important microRNA for epidermal differentiation and suppresses epithelial stem cell proliferation^31–34^. Its expression is drastically enhanced in the tissue surrounding a wound^35^.

The other DE microRNAs are, as of today, not as well-described, but are expressed in other processes relevant to tissue healing such as inflammation (miR-297, miR-3473, miR-338); smooth muscle differentiation or proliferation (miR-214, miR-139, miR-141); epithelial cell proliferation (miR-139, miR-92b); stem cell proliferation (miR-139); and fibroblast proliferation and migration (miR-214). Some have also been proposed as potential biomarkers for kidney injury (miR-3473) or potentially having a role in different cancers by modulating genes involved in migration, invasion, and proliferation of cancer cells (miR-139, miR-214, miR-1949, miR-141). One of the DE microRNAs has not yet been associated to any specific function but is known to be expressed in the kidneys (miR-6215) (Table 1).

It is worth noticing, that some relevant microRNAs were likely missed due to the chosen strength parameters for selecting DE genes (FDR < 0.05, P < 0.05, |logfold|> 0.25), that only detected high-scale changes. Several of these wound related microRNAs had a significant and informative differential expression and allowed to differentiate controls from wounded animals (supplementary fig. S2). For example, the microRNAs miR-146, miR-184, miR-202, miR-204, miR-205 and miR-221, have been previously reported as important players in tissue healing^36–39^. These microRNAs control processes such as inflammatory response, proliferation, angiogenesis, migration, or apoptosis. The expression of some of these microRNAs showed a differential expression between wounded versus non-wounded urinary bladders in our studies, however, they were not included in the list of the DE microRNAs, due to failure to pass the strength parameters for selection. Since these microRNAs are potentially relevant for wound healing in the urinary bladder, we included 3 of them in our panel for validation in human urothelial cells (miR-202, miR-221 and miR-146).

We found a high level of agreement of the microRNA expressions between human urothelial cells in vitro and rat bladder healing in vivo in the early phases of healing. Two out of three microRNAs had the same expression patterns, and this could indicate a potential role for these microRNAs in human urinary tissue healing as well.

Given the larger agreement between DE microRNA expression profiles in rat and human cells detected in the early time points, we believe that our experimental model was best suited for modeling the inflammation and proliferation stages of human wound healing.

Methodological weaknesses include the inherent issues associated to the hybridization-based platforms of microarrays (high background and cross-hybridization issues). They also demand stringent filtering to allow good confidence of results, which may be at the expense of biologically important information.

In this study, we validated our results in commercially available human cells to procure clinical relevance using a simplified model of in vitro wounding. Although the model cannot re-create the complexity of the in vivo wound healing events it does provide clues on the involvement of the DE microRNAs in epithelial migration and proliferation, which is an important event during the early phase of wound healing. Whole human bladder 3D culture models may be relevant in future studies.

Currently there are thousands of European and US patents related to microRNA targeting in cancer and other diseases. MicroRNA based therapeutics are at present in preclinical and clinical trials. For example, two drugs targeting members of the miR-92 family, are currently in clinical trials, for the treatment of peripheral arterial disease and controlling ischemia, respectively (miRAgen therapeutics Inc, CO, US)^40^.

The clinical relevance of our findings will need further elucidation, both within basic science and clinical studies, however, above-mentioned ongoing trials indirectly supports the role of targeting microRNA for influencing tissue hemostasis and function also in the bladder.

In conclusion, we investigated total microRNA gene expression during urinary bladder healing in a rodent model. We identified several DE microRNAs previously associated with wound healing and tissue repair, and DE microRNAs that were not previously associated with wound healing and tissue repair.

Bioinformatic analysis on the set of the target genes for the DE microRNAs indicates key molecules for extracellular matrix regulation (RUNX), proliferation (EF2, ERK) and angiogenesis (PI3k/AKT). Further studies will be needed to test the involvement of DE microRNAs in those processes**.**

MicroRNAs, identified in this study, could have clinical relevance in their use as therapeutic tools for regulating urogenital tissue inflammation and tissue healing. For example, they may improve the postoperative outcomes after hypospadias repair, bladder exstrophy repair or bladder augmentation. They may also play a role in chronic inflammation such as in radiation cystitis, cystitis cystica or painful bladder syndrome and influence research initiatives within regenerative medicine. Additional functional analysis will be required to increase our understanding of the specific mechanisms associated with each of the identified microRNAs in bladder repair, and therefore our data is made publicly available for other researchers to explore.

## Methods

### Ethics

This study was approved by the National animal ethics laws and Institutional regulations on animal studies (Stockholm County Committee on Animal Research, 20150703 Reg. N107/15).

All methods were performed in accordance with commonly accepted norms of veterinary best practice. The experiments were carried out at the Unit for comparative Medicine, Karolinska Institutet, Solna Sweden. The study was carried out in compliance with the ARRIVE guidelines^41^.

### Animals and urinary bladder wound model

Male Sprague–Dawley rats (approximately 400 g) were used in the experiments as described in previous communications^3^. The animals were divided into two groups: bladder wounded and sham operated animals. Several time points (0 h, 6 h, 2 days, 8 days, 14 days, and 28 days) after wounding of the bladder were studied. Four animals were included per time point and experimental condition and the study design is described in a schematic drawing (Fig. 1). Rats were anesthetized by inhalation of isoflurane (Virbac, Carros, France) and kept on isoflurane by mask inhalation during the whole surgical procedure. Surgery was performed under sterile conditions. An abdominal incision was made in the lower midline using a scalpel. Subsequently, the bladder was mobilized out of the wound and a 1 cm long longitudinal incision through all layers of the bladder was created from the anterior bladder neck up to the bladder dome. The incision was then closed with a continuous 7-0 nylon suture (Ethicon, Somerville, NJ). Local anesthesia with bupivacaine (Marcaine) (Astra Zeneca, Södertälje, Sweden) was applied in the subcutaneous tissue before closure of the abdominal wall. The rectus muscle and skin were closed sequentially with continuous 4‐0 absorbable Vicryl suture (Ethicon, Somerville, NJ). Sham operations included an equivalent procedure at all steps except the incision and suturing of the bladder.

The rats were euthanized with carbon dioxide at the above indicated time points after the initial intervention, bladder biopsies including the wound were harvested and stored in RNAlater (Qiagen, Hilden, Germany) until RNA extraction. Samples from 6 h, 2 days and 8 days after wounding were used for all microRNA array analyses. RNAs extracted from all the time points including 14 days and 24 days were used for the validation studies.

Until the end of the study, no complications related to the surgical interventions or bladder operations were detected in any animals.

### RNA extraction

Bladder tissue biopsies were homogenized as previously described ^3^, using a Tissue Lyser, (Qiagen, Hilden, Germany) before RNA extraction. Total RNA was extracted using the Qiazol Lysis Reagent® (Thermo Fisher Scientific, Waltham, MA), following the manufacturer’s instructions. The quantity of RNA was determined using the NanoDrop® ND-1000 Spectrophotometer (NanoDrop Technologies inc, Wilmington, DE). RNA integrity (RIN) was determined using an Agilent 2100 Bioanalyser (RNA 6000 Nano LabChip kit, Agilent Technologies).

### MicroRNA profiling by Affymetrix® GeneChip® microRNA Arrays

The Affymetrix microRNA GeneChip Array plates (Affymetrix, Thermo Fisher) were used in this study according to the manufacturer’s instructions at the core facility for Bioinformatics and Expression Analysis (BEA, Karolinska Institutet, Stockholm, Sweden). In brief, two hundred micrograms of total RNA from bladder samples (collected after 6 h, 2 days and 8 days post wounding) were biotin-labeled for the Affymetrix GeneChip microRNA array 4.1 using the FlashTag biotin-HSR RNA labeling kit (Thermo Fisher Scientific). Each microarray contained sequences to interrogate all mature microRNA sequences in MiRBase Release 17. Hybridization, washing, and scanning of slides were performed according to the Affymetrix protocols. The scanned images were processed using the Affymetrix GeneChip Command Console. CEL files from scanning were imported to Transcriptome Analysis Console (TAC v4.00) and analyzed using the RMA method, that generated signals and Detection Above Background (DABG) p-values. Wounded and non-wounded bladders were compared for each time point using the Moderate t-test and the false discovery rates were calculated.

### Bioinformatic analysis, detection of DE microRNAs

The raw data were analyzed using the Applied BioSystems Transcriptome Analysis Console (TAC) software as described above. The TAC software was used to determine the intensity levels of each probe in each individual, and to perform differential expression analysis between different conditions and time points, and to filter probes not displaying a signal in any of the individuals.

To provide information on the overall structure of the analyzed data set, several common methods for analysis of gene expression of microarray data were implemented using custom scripts by using the R ggplot package^42^. The method included a principal component analysis (PCA), that is a dimensionality reduction technique providing information on the direction of the highest variability in the data. By these means, the first component contained genes describing the most variation, the second a bit less, and the last component containing genes with very little variation.

Heatmaps and hierarchical clusters of genes with similar expression patterns and genes that were more similar were placed together in a dendrogram. Branches in the dendrogram represented similarities in gene expression patterns.

Volcano plots were used to display the statistical significance and the magnitude of gene expression changes and allowed for rapid visualization and identification of genes with large fold changes, that were also significant.

Next, the data was searched for DE microRNAs. Any microRNA displaying a p-value < 0.05, and absolute fold change > 0.25 was considered DE. The resulting DE microRNA was then analyzed with two separate settings: first, the DE microRNA underwent FDR correction, (setting an FDR threshold at < 0.05), and next, the microRNA was analyzed using no FDR correction.

The gene targets of these microRNAs were determined using the Rattus Norvegicus microRNA targets as listed in miRDB v6.0^43^.

The resulting gene target list were analyzed using the microRNA target filter tool of QIAGEN’s Ingenuity Pathway Analysis software in combination with a list of 84 mRNA gene expression data from previously published work^3^. Lastly, the targets of the FDR corrected DE microRNA were analyzed using the PANTHER GO biological process enrichment tool (pantherdb.org) as well as the REACTOME pathway analysis tool (https://reactome.org/PathwayBrowser/#TOOL=AT).

### Validation of DE microRNAs by quantitative RT-PCR

Quantitative real-time reverse transcriptase–PCR was performed as described previously^44^. In brief, the total RNA from rat bladder samples and from primary human urothelial cells was reversely transcribed using the TaqMan microRNA reverse transcription kit and the multiplex RT primer pool containing microRNA specific stem-loop primers (Thermo Fisher Scientific, Foster City, CA). Thereafter, the expression of each microRNA was determined by TaqMan expression assays (Thermo Fisher Scientific) and normalized based on the values of U6 small nuclear RNA (in all rat samples) and the RNU48 (in all human samples) Information for all the TaqMan assays used in this study can be found in Table 4.

### Human primary urothelial cell culture

Primary human urothelial cells (Catalog #4320) and supplements were purchased from ScienCell Research Laboratories and cultured at a minimum density of 5,000 cells/ cm^2^ in a poly-L-lysine (Catalog #041) coated culture flask and using Urothelial Cell Medium (UCM, Catalog #4321) and growth factor supplements (Catalog #4352). Cells were used for the experiments at passage number four.

### In vitro wound healing assay in human urothelial cells

Sub-confluent primary urothelial cells were plated into 35 mm poly-L-lysine-coated culture dishes at a density of 40,000 cells/cm^2^. 24 h after plating, the cells formed a 100% confluent monolayer. The monolayer was scraped with seven straight longitudinal and seven straight vertical lines with a p200 pipet tip. The cellular debris was removed from the petri dishes by washing twice with 1 × PBS and 2 ml of urothelial cell medium without cell growth supplements added to the plates. After 6, 12 and 24 h, the cells were harvested for RNA extraction and gene expression analysis as described above.

### Statistical analysis

The statistical significance in the RT-PCR analysis of selected microRNA molecules in both rat bladder and human urothelial cells was evaluated by calculating the average normalized gene expression values in each biological replicate and time point. These data were imported in the Prism 5.0 software (GraphPad, San Diego, CA). The nonparametric Mann–Whitney U test was used to evaluate the significance of microRNA genes of wounded vs. control groups. The bars represent the average values of gene expression and their respective standard deviation.

## Supplementary Information

Supplementary Information 1.

Supplementary Information 2.

Supplementary Information 3.

Supplementary Information 4.

Supplementary Information 5.

## Data Availability

The microarray data can be found at Gene Expression Omnibus (GSE176515).
