# Supplementary material for: A database on differentially expressed microRNAs during rodent bladder healing
Source: Sci Rep. 2021 Nov 8;11:21881. doi: 10.1038/s41598-021-01413-0 (PMC8575992; doi:10.1038/s41598-021-01413-0)
Supplement: Supplementary file 1 — Supplementary Information 1. [file 41598_2021_1413_MOESM1_ESM.pdf]

Supplementary material
Manuscript ID 37b7d554-82c2-48e4-bcff-08125556c418
A database on differentially expressed microRNAs during rodent bladder healing

Supplementary information:

Supplementary Table 1: Target gene for the top-40 highest expressed microRNAs
Column 1: Gene ID
Column 2: numbers of microRNAs targeting
Column 3: Identity of the microRNAs

Supplementary Table 2: GO associated with the target genes for the TOP- 40 highest expressed microRNAs
Contains gene ontology analysis in the target genes for the TOP- 40 highest expressed microRNAs. The PANTHER GO biological process enrichment tool was used in this analysis

Column 1: Biological Process
Column 2: Number of genes in the reference
Column 3: Number of genes targeted by DEGs
Column 4: Fraction expected
Column 5: Enrichment(+) or depletion(-)
Column 6: Fold change
Column 7: P value
Column 8: False discovery rate

Supplementary Table 3: List of total significant differentially expressed microRNA at 6 hours post wounding. Table shows in
Column 1: microRNA ID,
Column 2: lo2 fold change,
Column3: Significance of the difference (p value)
Column 4: False discovery rate (FDR)
Column 5: Transcript ID in the Affymetrix array design

Supplementary Table 4: List of total significant differentially expressed microRNA at 2 days post wounded
Column 1: microRNA ID,
Column 2: lo2 fold change,
Column3: Significance of the difference (p value)
Column 4: False discovery rate (FDR)
Column 5: Transcript ID in the Affymetrix array design

Supplementary Table 5 List of total significant differentially expressed microRNA at 8 days post wounded

Column 1: microRNA ID,
Column 2: lo2 fold change,
Column3: Significance of the difference (p value)
Column 4: False discovery rate (FDR)
Column 5: Transcript ID in the Affymetrix array design

Supplementary table 6. Contains gene ontology analysis in differentially expressed genes at 6h post wounding. The PANTHER GO biological process enrichment tool was used in this analysis

Column 1: Biological Process
Column 2: Number of genes in the reference
Column 3: Number of genes targeted by DEGs
Column 4: Fraction expected
Column 5: Enrichment (+) or depletion(-)
Column 6: Fold change
Column 7: P value
Column 8: False discovery rate

Supplementary table 7. Contains gene ontology analysis in differentially expressed genes at 2d post wounding. The PANTHER GO biological process enrichment tool was used in this analysis

Column 1: Biological Process
Column 2: Number of genes in the reference
Column 3: Number of genes targeted by DEGs
Column 4: Fraction expected
Column 5: Enrichment(+) or depletion(-)
Column 6: Fold change
Column 7: P value
Column 8: False discovery rate

Supplementary table 8. Contains gene ontology analysis in differentially expressed genes at 8d post wounding. The PANTHER GO biological process enrichment tool was used in this analysis
Column 1: Biological Process
Column 2: Number of genes in the reference
Column 3: Number of genes targeted by DEGs
Column 4: Fraction expected
Column 5: Enrichment (+) or depletion (-)
Column 6: Fold change
Column 7: P value
Column 8: False discovery rate

Supplementary table 9, 10 and 11: Showing the 25 most relevant pathways sorted by p-value at 6h, 2d and 8 days respectively.
Analysis was performed against REACTOME version 77 on 31/08/2021. The web link to these results is:
https://reactome.org/PathwayBrowser/#/ANALYSIS=MjAyMTA4MzExNDAxNThfMjI3ODc%3D
A statistical (hypergeometric distribution) test that determines whether certain Reactome pathways are over-represented (enriched) in the submitted data. It answers the question ‘Does my list contain more proteins for pathway X than would be expected by chance?’ This test produces a probability score, which is corrected for false discovery rate using the Benjamani-Hochberg method

Supplementary figure S1: Expression profiling of the top 40 highest expressed microRNAs in the experimental urinary wound healing model showing a a. PCA plot and b. a heatmap

Supplementary figure S2 expression profiling in a set of selected wound healing related microRNA, illustrating a a.PCA plot and b. a heatmap.
