# Supplementary figures and images for: A database on differentially expressed microRNAs during rodent bladder healing
Source: Sci Rep. 2021 Nov 8;11:21881. doi: 10.1038/s41598-021-01413-0 (PMC8575992; doi:10.1038/s41598-021-01413-0)

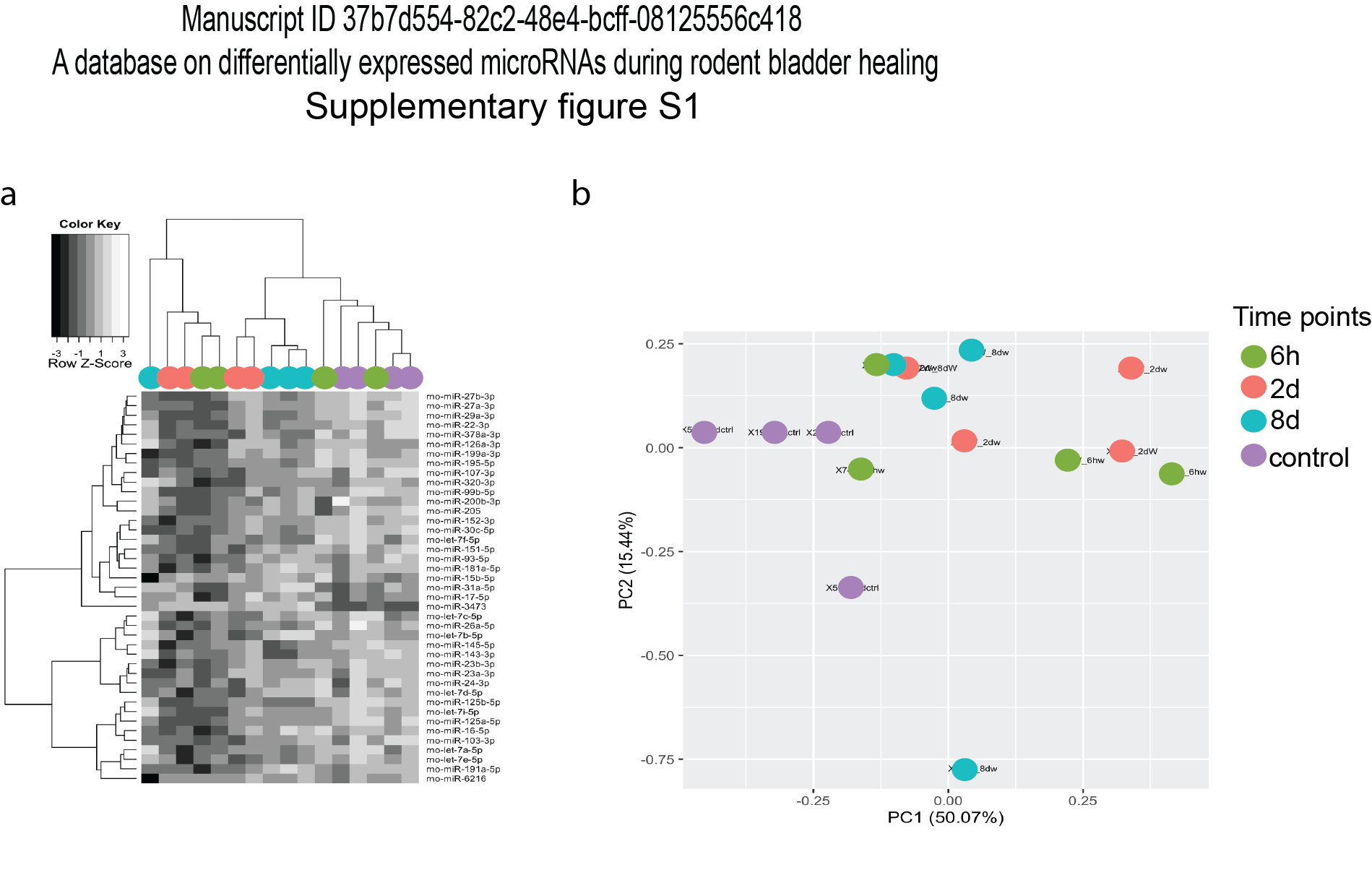

Supplement: Supplementary file 2 — Supplementary Information 2. [file 41598_2021_1413_MOESM2_ESM.png]

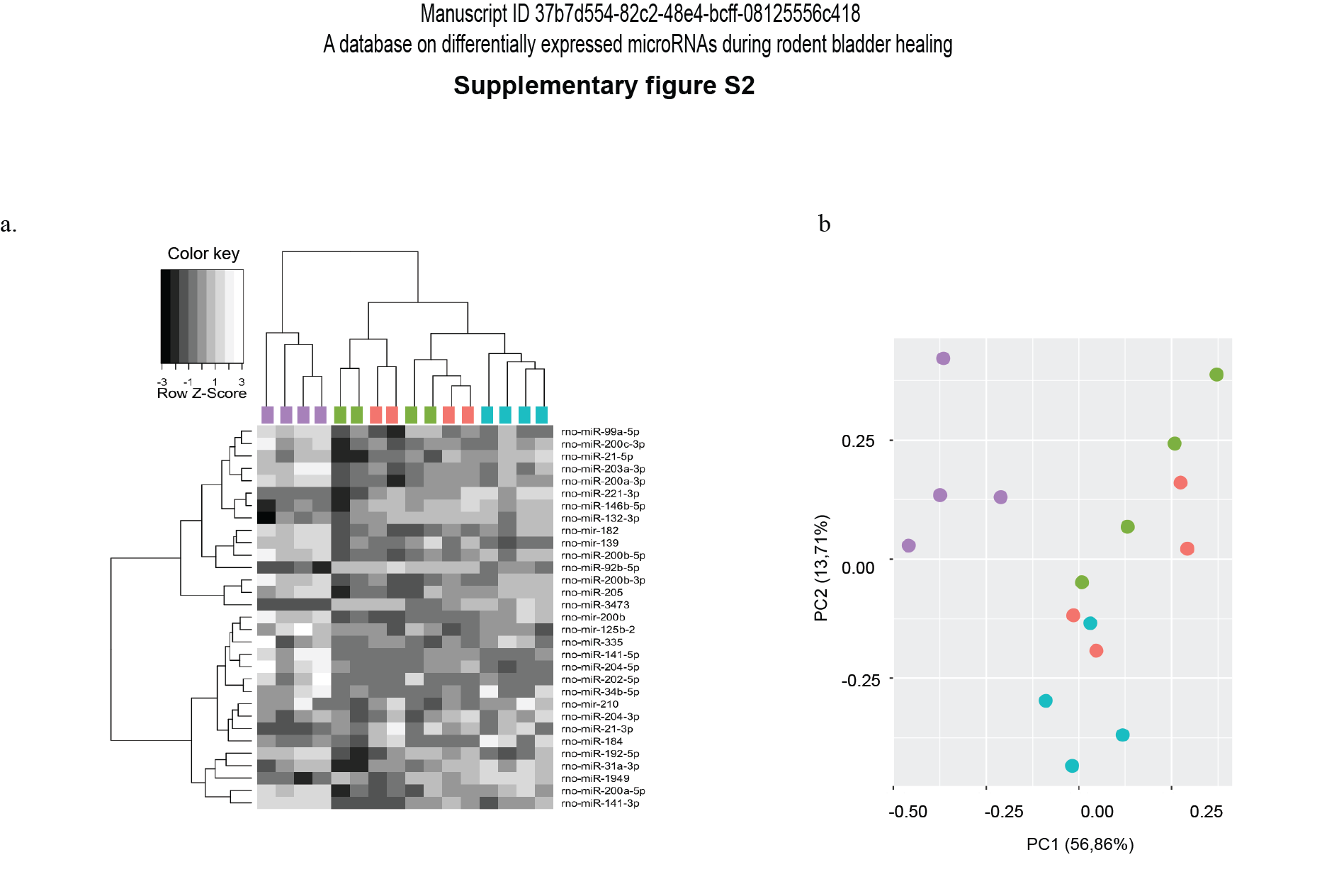

Supplement: Supplementary file 3 — Supplementary Information 3. [file 41598_2021_1413_MOESM3_ESM.png]
