# Supplementary material for: A database on differentially expressed microRNAs during rodent bladder healing
Source: Sci Rep. 2021 Nov 8;11:21881. doi: 10.1038/s41598-021-01413-0 (PMC8575992; doi:10.1038/s41598-021-01413-0)
Supplement: Supplementary file 4 — Supplementary Information 4. [file 41598_2021_1413_MOESM4_ESM.pdf]

Suplemmentary material
Manuscript ID 37b7d554-82c2-48e4-bcff-08125556c418A.
"A database on differentially expressed microRNAs during rodent bladder healing"

Supplementary table S1: target genes for the top-40 highest expressed microRNAs

Gene	counts	mirnas
NM_001107740	1	rno-miR-145-5p
NM_057115	2	rno-miR-145-5p|rno-miR-200b-3p
NM_001130568	1	rno-miR-145-5p
NM_053623	9	rno-miR-145-5p|rno-miR-26a-5p|rno-miR-6216|rno-miR-16-5p|rno-miR-195-5p|rno-miR-181a-5p|rno-miR-17-5p|rno-miR-15b-5p|rno-miR-93-5p
NM_052801	1	rno-miR-145-5p
NM_001017456	1	rno-miR-145-5p
NM_001109007	3	rno-miR-145-5p|rno-miR-125b-5p|rno-miR-125a-5p
NM_001100736	10	rno-miR-145-5p|rno-let-7c-5p|rno-let-7b-5p|rno-let-7d-5p|rno-let-7a-5p|rno-let-7e-5p|rno-let-7i-5p|rno-miR-27b-3p|rno-miR-27a-3p|rno-let-7f-5p
NM_001105881	8	rno-miR-145-5p|rno-miR-23a-3p|rno-miR-23b-3p|rno-miR-27b-3p|rno-miR-27a-3p|rno-miR-200b-3p|rno-miR-181a-5p|rno-miR-31a-5p
NM_053698	1	rno-miR-145-5p
NM_001108501	4	rno-miR-145-5p|rno-miR-23a-3p|rno-miR-23b-3p|rno-miR-181a-5p
NM_001191693	4	rno-miR-145-5p|rno-miR-26a-5p|rno-miR-6216|rno-miR-152-3p

NM_001007617	7	rno-miR-145-5p|rno-miR-143-3p|rno-miR-23a-3p|rno-miR-23b-3p|rno-miR-16-5p|rno-miR-195-5p|rno-miR-15b-5p
NM_017076	8	rno-miR-145-5p|rno-let-7c-5p|rno-let-7b-5p|rno-let-7d-5p|rno-let-7a-5p|rno-let-7e-5p|rno-let-7i-5p|rno-let-7f-5p
NM_019225	1	rno-miR-145-5p
NM_001105934	3	rno-miR-145-5p|rno-miR-103-3p|rno-miR-107-3p
NM_001033675	3	rno-miR-145-5p|rno-miR-27b-3p|rno-miR-27a-3p
NM_001107654	5	rno-miR-145-5p|rno-miR-125b-5p|rno-miR-125a-5p|rno-miR-17-5p|rno-miR-93-5p
NM_001108707	10	rno-miR-145-5p|rno-let-7c-5p|rno-miR-26a-5p|rno-let-7b-5p|rno-let-7d-5p|rno-let-7a-5p|rno-let-7e-5p|rno-let-7i-5p|rno-miR-200b-3p|rno-let-7f-5p
NM_001107453	1	rno-miR-145-5p
NM_001039023	5	rno-miR-145-5p|rno-miR-23a-3p|rno-miR-23b-3p|rno-miR-199a-3p|rno-miR-22-3p
NM_024489	5	rno-miR-145-5p|rno-miR-103-3p|rno-miR-107-3p|rno-miR-27b-3p|rno-miR-27a-3p
NM_001305426	1	rno-miR-145-5p
NM_053758	1	rno-miR-145-5p
NM_001105912	3	rno-miR-145-5p|rno-miR-17-5p|rno-miR-93-5p
NM_001107769	3	rno-miR-145-5p|rno-miR-24-3p|rno-miR-199a-3p
NM_001107684	9	rno-miR-145-5p|rno-miR-16-5p|rno-miR-3473|rno-miR-27b-3p|rno-miR-195-5p|rno-miR-27a-3p|rno-miR-200b-3p|rno-miR-30c-5p|rno-miR-15b-5p
NM_053442	1	rno-miR-145-5p
NM_001106736	2	rno-miR-145-5p|rno-miR-30c-5p
NM_052829	1	rno-miR-145-5p
NM_013095	1	rno-miR-145-5p

NM_001110811	1	rno-miR-145-5p
NM_001047099	2	rno-miR-145-5p|rno-miR-181a-5p
NM_022946	1	rno-miR-145-5p
NM_001106668	2	rno-miR-145-5p|rno-miR-151-5p
NM_001108279	2	rno-miR-145-5p|rno-miR-181a-5p
NM_001170335	1	rno-miR-145-5p
NM_001100496	1	rno-miR-145-5p
NM_001008300	4	rno-miR-145-5p|rno-miR-30c-5p|rno-miR-17-5p|rno-miR-93-5p
NM_001106986	1	rno-miR-145-5p
NM_001107619	4	rno-miR-145-5p|rno-miR-16-5p|rno-miR-195-5p|rno-miR-15b-5p
NM_001106353	6	rno-miR-145-5p|rno-miR-26a-5p|rno-miR-103-3p|rno-miR-107-3p|rno-miR-30c-5p|rno-miR-152-3p
NM_001106641	3	rno-miR-145-5p|rno-miR-17-5p|rno-miR-93-5p
NM_001107548	8	rno-miR-145-5p|rno-miR-16-5p|rno-miR-103-3p|rno-miR-107-3p|rno-miR-27b-3p|rno-miR-195-5p|rno-miR-27a-3p|rno-miR-15b-5p
NM_053823	4	rno-miR-145-5p|rno-miR-29a-3p|rno-miR-17-5p|rno-miR-93-5p
NM_001271264	3	rno-miR-145-5p|rno-miR-26a-5p|rno-miR-181a-5p
NM_053722	15	rno-miR-145-5p|rno-let-7c-5p|rno-miR-26a-5p|rno-let-7b-5p|rno-miR-23a-3p|rno-miR-23b-3p|rno-let-7d-5p|rno-let-7a-5p|rno-let-7e-5p|rno-miR-6216|rno-let-7i-5p|rno-miR-320-3p|rno-miR-200b-3p|rno-miR-31a-5p|rno-let-7f-5p
NM_133542	3	rno-miR-145-5p|rno-miR-320-3p|rno-miR-181a-5p
NM_001113365	2	rno-miR-145-5p|rno-miR-181a-5p
NM_001106826	1	rno-miR-145-5p
NM_001177909	1	rno-miR-145-5p
NM_001135835	5	rno-miR-145-5p|rno-miR-16-5p|rno-miR-195-5p|rno-miR-31a-5p|rno-miR-15b-5p
NM_022923	1	rno-miR-145-5p
NM_001142941	2	rno-miR-145-5p|rno-miR-6216
NM_001130536	1	rno-miR-145-5p

NM_053420	1	rno-miR-145-5p
NM_001100692	12	rno-miR-145-5p|rno-miR-26a-5p|rno-miR-23a-3p|rno-miR-23b-3p|rno-miR-103-3p|rno-miR-107-3p|rno-miR-320-3p|rno-miR-27b-3p|rno-miR-205|rno-miR-27a-3p|rno-miR-181a-5p|rno-miR-152-3p
NM_001106838	2	rno-miR-145-5p|rno-miR-30c-5p
NM_172329	4	rno-miR-145-5p|rno-miR-24-3p|rno-miR-22-3p|rno-miR-29a-3p
NM_178095	1	rno-miR-145-5p
NM_031046	3	rno-miR-145-5p|rno-miR-103-3p|rno-miR-107-3p
NM_001135596	9	rno-miR-145-5p|rno-miR-6216|rno-miR-103-3p|rno-miR-107-3p|rno-miR-320-3p|rno-miR-200b-3p|rno-miR-181a-5p|rno-miR-152-3p|rno-miR-151-5p
NM_001107399	10	rno-miR-145-5p|rno-let-7c-5p|rno-let-7b-5p|rno-let-7d-5p|rno-let-7a-5p|rno-let-7e-5p|rno-let-7i-5p|rno-miR-205|rno-miR-29a-3p|rno-let-7f-5p
NM_001106289	1	rno-miR-145-5p
NM_001033062	1	rno-miR-145-5p
NM_001106659	1	rno-miR-145-5p
NM_001107024	4	rno-miR-145-5p|rno-miR-26a-5p|rno-miR-17-5p|rno-miR-93-5p
NM_019328	1	rno-miR-145-5p
NM_001107117	2	rno-miR-145-5p|rno-miR-6216
NM_001106375	2	rno-miR-145-5p|rno-miR-143-3p
NM_001170551	14	rno-miR-145-5p|rno-let-7c-5p|rno-miR-26a-5p|rno-let-7b-5p|rno-let-7d-5p|rno-let-7a-5p|rno-let-7e-5p|rno-miR-6216|rno-let-7i-5p|rno-miR-3473|rno-miR-378a-3p|rno-miR-17-5p|rno-let-7f-5p|rno-miR-93-5p
NM_001107173	5	rno-miR-145-5p|rno-miR-6216|rno-miR-378a-3p|rno-miR-17-5p|rno-miR-93-5p
NM_030827	3	rno-miR-145-5p|rno-miR-6216|rno-miR-199a-3p
NM_001107201	2	rno-miR-145-5p|rno-miR-181a-5p
NM_001107126	4	rno-miR-145-5p|rno-miR-26a-5p|rno-miR-125b-5p|rno-miR-125a-5p

NM_001012049	1	rno-miR-145-5p
NM_001025130	3	rno-miR-145-5p|rno-miR-26a-5p|rno-miR-6216
NM_001108935	4	rno-miR-145-5p|rno-miR-320-3p|rno-miR-200b-3p|rno-miR-30c-5p
NM_134454	2	rno-miR-145-5p|rno-miR-205
NM_012761	2	rno-miR-145-5p|rno-miR-30c-5p
NM_001034021	2	rno-miR-145-5p|rno-miR-181a-5p
NM_001126291	4	rno-miR-145-5p|rno-miR-199a-3p|rno-miR-27b-3p|rno-miR-27a-3p
NM_001109382	5	rno-miR-145-5p|rno-miR-16-5p|rno-miR-195-5p|rno-miR-205|rno-miR-15b-5p
NM_001106081	5	rno-miR-145-5p|rno-miR-6216|rno-miR-16-5p|rno-miR-195-5p|rno-miR-15b-5p
NM_001130506	2	rno-miR-145-5p|rno-miR-24-3p
NM_001105755	5	rno-miR-145-5p|rno-miR-3473|rno-miR-27b-3p|rno-miR-378a-3p|rno-miR-27a-3p
NM_001107365	3	rno-miR-145-5p|rno-miR-3473|rno-miR-29a-3p
NM_001107288	2	rno-miR-145-5p|rno-miR-31a-5p
NM_053879	3	rno-miR-145-5p|rno-miR-181a-5p|rno-miR-152-3p
NM_001170593	2	rno-miR-145-5p|rno-miR-199a-3p
NM_001012041	1	rno-miR-145-5p
NM_053382	4	rno-miR-145-5p|rno-miR-23a-3p|rno-miR-23b-3p|rno-miR-181a-5p
NM_001172159	3	rno-miR-145-5p|rno-miR-125b-5p|rno-miR-125a-5p
NM_134460	4	rno-miR-145-5p|rno-miR-320-3p|rno-miR-205|rno-miR-152-3p
NM_001109093	5	rno-miR-145-5p|rno-miR-26a-5p|rno-miR-125b-5p|rno-miR-125a-5p|rno-miR-181a-5p
NM_001107101	3	rno-miR-145-5p|rno-miR-181a-5p|rno-miR-30c-5p
NM_001033699	5	rno-miR-145-5p|rno-miR-24-3p|rno-miR-16-5p|rno-miR-195-5p|rno-miR-15b-5p
NM_022276	5	rno-miR-145-5p|rno-miR-23a-3p|rno-miR-23b-3p|rno-miR-125b-5p|rno-miR-125a-5p

NM_001105668	8	rno-miR-145-5p|rno-miR-23a-3p|rno-miR-23b-3p|rno-miR-200b-3p|rno-miR-31a-5p|rno-miR-17-5p|rno-miR-152-3p|rno-miR-93-5p
NM_139093	1	rno-miR-145-5p
NM_017107	7	rno-miR-145-5p|rno-miR-26a-5p|rno-miR-23a-3p|rno-miR-23b-3p|rno-miR-24-3p|rno-miR-103-3p|rno-miR-107-3p
NM_001170548	2	rno-miR-145-5p|rno-miR-143-3p
NM_001108415	1	rno-miR-145-5p
NM_001024791	1	rno-miR-145-5p
NM_001106084	2	rno-miR-145-5p|rno-miR-24-3p
NM_001108506	3	rno-miR-145-5p|rno-miR-23a-3p|rno-miR-23b-3p
NM_017120	1	rno-miR-145-5p
NM_145774	3	rno-miR-145-5p|rno-miR-320-3p|rno-miR-30c-5p
NM_001105862	11	rno-miR-145-5p|rno-let-7c-5p|rno-let-7b-5p|rno-miR-24-3p|rno-let-7d-5p|rno-let-7a-5p|rno-let-7e-5p|rno-let-7i-5p|rno-miR-320-3p|rno-miR-181a-5p|rno-let-7f-5p
NM_001105840	1	rno-miR-145-5p
NM_001106880	5	rno-miR-145-5p|rno-miR-24-3p|rno-miR-27b-3p|rno-miR-27a-3p|rno-miR-181a-5p
NM_001106588	1	rno-miR-145-5p
NM_001012064	2	rno-miR-145-5p|rno-miR-199a-3p
NM_001107881	3	rno-miR-145-5p|rno-miR-27b-3p|rno-miR-27a-3p
NM_001013191	7	rno-miR-145-5p|rno-miR-143-3p|rno-miR-125b-5p|rno-miR-125a-5p|rno-miR-27b-3p|rno-miR-27a-3p|rno-miR-30c-5p
NM_053964	1	rno-miR-145-5p
NM_001105725	4	rno-miR-145-5p|rno-miR-6216|rno-miR-17-5p|rno-miR-93-5p
NM_001134561	6	rno-miR-145-5p|rno-miR-26a-5p|rno-miR-6216|rno-miR-320-3p|rno-miR-27b-3p|rno-miR-27a-3p
NM_001100723	2	rno-miR-145-5p|rno-miR-24-3p
NM_031044	2	rno-miR-145-5p|rno-miR-24-3p
NM_001014258	1	rno-miR-145-5p

NM_031591	1	rno-miR-145-5p
NM_001015018	1	rno-miR-145-5p
NM_001271233	6	rno-miR-145-5p|rno-miR-22-3p|rno-miR-29a-3p|rno-miR-200b-3p|rno-miR-30c-5p|rno-miR-151-5p
NM_001106773	1	rno-miR-145-5p
NM_001127446	1	rno-miR-145-5p
NM_001271047	9	rno-miR-145-5p|rno-let-7c-5p|rno-let-7b-5p|rno-miR-24-3p|rno-let-7d-5p|rno-let-7a-5p|rno-let-7e-5p|rno-let-7i-5p|rno-let-7f-5p
NM_001107980	1	rno-miR-145-5p
NM_001107639	18	rno-miR-145-5p|rno-let-7c-5p|rno-let-7b-5p|rno-miR-23a-3p|rno-miR-23b-3p|rno-miR-24-3p|rno-let-7d-5p|rno-let-7a-5p|rno-let-7e-5p|rno-miR-6216|rno-miR-125b-5p|rno-let-7i-5p|rno-miR-125a-5p|rno-miR-99b-5p|rno-miR-200b-3p|rno-miR-31a-5p|rno-miR-30c-5p|rno-let-7f-5p
NM_012938	1	rno-miR-145-5p
NM_001014143	5	rno-miR-145-5p|rno-miR-27b-3p|rno-miR-27a-3p|rno-miR-17-5p|rno-miR-93-5p
NM_001270416	6	rno-miR-145-5p|rno-miR-16-5p|rno-miR-195-5p|rno-miR-17-5p|rno-miR-15b-5p|rno-miR-93-5p
NM_001013896	13	rno-miR-145-5p|rno-miR-26a-5p|rno-miR-23a-3p|rno-miR-23b-3p|rno-miR-16-5p|rno-miR-125b-5p|rno-miR-103-3p|rno-miR-125a-5p|rno-miR-107-3p|rno-miR-195-5p|rno-miR-17-5p|rno-miR-15b-5p|rno-miR-93-5p
NM_001109010	2	rno-miR-145-5p|rno-miR-26a-5p
NM_001100517	2	rno-miR-145-5p|rno-miR-24-3p
NM_012839	1	rno-miR-145-5p
NM_001289942	1	rno-miR-145-5p
NM_001277056	5	rno-miR-145-5p|rno-miR-26a-5p|rno-miR-27b-3p|rno-miR-27a-3p|rno-miR-152-3p
NM_001191975	4	rno-miR-145-5p|rno-miR-23a-3p|rno-miR-23b-3p|rno-miR-6216

NM_019274	6	rno-miR-145-5p|rno-miR-16-5p|rno-miR-27b-3p|rno-miR-195-5p|rno-miR-27a-3p|rno-miR-15b-5p
NM_001099647	1	rno-miR-145-5p
NM_013115	1	rno-miR-145-5p
NM_001109301	12	rno-miR-145-5p|rno-let-7c-5p|rno-let-7b-5p|rno-miR-23a-3p|rno-miR-23b-3p|rno-let-7d-5p|rno-let-7a-5p|rno-let-7e-5p|rno-let-7i-5p|rno-miR-27b-3p|rno-miR-27a-3p|rno-let-7f-5p
NM_001170546	2	rno-miR-145-5p|rno-miR-143-3p
NM_001109013	1	rno-miR-145-5p
NM_001108405	1	rno-miR-145-5p
NM_001289941	1	rno-miR-145-5p
NM_001024243	1	rno-miR-145-5p
NM_012959	1	rno-miR-145-5p
NM_001108251	1	rno-miR-145-5p
NM_001304287	1	rno-miR-145-5p
NM_001012144	1	rno-miR-145-5p
NM_001108462	3	rno-miR-145-5p|rno-miR-320-3p|rno-miR-15b-5p
NM_001135754	3	rno-miR-145-5p|rno-miR-29a-3p|rno-miR-152-3p
NM_001108382	9	rno-miR-145-5p|rno-let-7c-5p|rno-let-7b-5p|rno-miR-24-3p|rno-let-7d-5p|rno-let-7a-5p|rno-let-7e-5p|rno-let-7i-5p|rno-let-7f-5p
NM_001107833	1	rno-miR-145-5p
NM_001127503	6	rno-miR-145-5p|rno-miR-125b-5p|rno-miR-125a-5p|rno-miR-27b-3p|rno-miR-27a-3p|rno-miR-29a-3p
NM_001107736	2	rno-miR-145-5p|rno-miR-26a-5p
NM_001012093	1	rno-miR-145-5p
NM_184046	1	rno-miR-145-5p
NM_001105950	2	rno-miR-145-5p|rno-miR-6216
NM_001013178	1	rno-miR-145-5p
NM_021691	2	rno-miR-145-5p|rno-miR-6216

NM_001013108	7	rno-miR-145-5p|rno-miR-16-5p|rno-miR-103-3p|rno-miR-107-3p|rno-miR-195-5p|rno-miR-200b-3p|rno-miR-15b-5p
NM_001108622	2	rno-miR-145-5p|rno-miR-26a-5p
NM_001083966	8	rno-miR-145-5p|rno-miR-6216|rno-miR-27b-3p|rno-miR-27a-3p|rno-miR-29a-3p|rno-miR-30c-5p|rno-miR-17-5p|rno-miR-93-5p
NM_001107864	10	rno-miR-145-5p|rno-let-7c-5p|rno-let-7b-5p|rno-let-7d-5p|rno-let-7a-5p|rno-let-7e-5p|rno-let-7i-5p|rno-miR-320-3p|rno-miR-200b-3p|rno-let-7f-5p
NM_031327	2	rno-miR-145-5p|rno-miR-181a-5p
NM_001106276	3	rno-miR-145-5p|rno-miR-320-3p|rno-miR-22-3p
NM_001107348	8	rno-miR-145-5p|rno-miR-16-5p|rno-miR-320-3p|rno-miR-27b-3p|rno-miR-195-5p|rno-miR-27a-3p|rno-miR-31a-5p|rno-miR-15b-5p
NM_001008880	10	rno-miR-145-5p|rno-let-7c-5p|rno-let-7b-5p|rno-let-7d-5p|rno-let-7a-5p|rno-let-7e-5p|rno-miR-125b-5p|rno-let-7i-5p|rno-miR-125a-5p|rno-let-7f-5p
NM_001007645	2	rno-miR-145-5p|rno-miR-181a-5p
NM_001109521	3	rno-miR-145-5p|rno-miR-205|rno-miR-151-5p
NM_001106278	2	rno-miR-145-5p|rno-miR-30c-5p
NM_001025717	3	rno-miR-145-5p|rno-miR-320-3p|rno-miR-22-3p
NM_001108586	12	rno-miR-145-5p|rno-let-7c-5p|rno-miR-26a-5p|rno-let-7b-5p|rno-miR-23a-3p|rno-miR-23b-3p|rno-let-7d-5p|rno-let-7a-5p|rno-let-7e-5p|rno-miR-6216|rno-let-7i-5p|rno-let-7f-5p
NM_001177683	1	rno-miR-145-5p
NM_182843	1	rno-miR-145-5p
NM_053394	3	rno-miR-145-5p|rno-miR-143-3p|rno-miR-320-3p
NM_001108113	2	rno-miR-145-5p|rno-miR-29a-3p
NM_022392	4	rno-miR-145-5p|rno-miR-143-3p|rno-miR-24-3p|rno-miR-29a-3p
NM_017061	1	rno-miR-145-5p
NM_001013898	1	rno-miR-145-5p
NM_138505	2	rno-miR-145-5p|rno-miR-30c-5p

NM_001191758	2	rno-miR-145-5p|rno-miR-143-3p
NM_001025008	1	rno-miR-145-5p
NM_001108518	2	rno-miR-145-5p|rno-miR-29a-3p
NM_001107403	2	rno-miR-145-5p|rno-miR-30c-5p
NM_182950	6	rno-miR-145-5p|rno-miR-16-5p|rno-miR-195-5p|rno-miR-29a-3p|rno-miR-181a-5p|rno-miR-15b-5p
NM_001107361	5	rno-miR-145-5p|rno-miR-26a-5p|rno-miR-27b-3p|rno-miR-27a-3p|rno-miR-29a-3p
NM_023954	3	rno-miR-145-5p|rno-miR-143-3p|rno-miR-24-3p
NM_001108038	11	rno-miR-145-5p|rno-let-7c-5p|rno-let-7b-5p|rno-let-7d-5p|rno-let-7a-5p|rno-let-7e-5p|rno-let-7i-5p|rno-miR-17-5p|rno-miR-152-3p|rno-let-7f-5p|rno-miR-93-5p
NM_001100568	4	rno-miR-145-5p|rno-miR-320-3p|rno-miR-17-5p|rno-miR-93-5p
NM_053845	1	rno-miR-145-5p
NM_001127504	5	rno-miR-145-5p|rno-miR-143-3p|rno-miR-23a-3p|rno-miR-23b-3p|rno-miR-30c-5p
NM_001105759	1	rno-miR-145-5p
NM_001107952	4	rno-miR-145-5p|rno-miR-22-3p|rno-miR-17-5p|rno-miR-93-5p
NM_001108083	3	rno-miR-145-5p|rno-miR-6216|rno-miR-30c-5p
NM_001109230	1	rno-miR-145-5p
NM_001106057	5	rno-miR-145-5p|rno-miR-125b-5p|rno-miR-125a-5p|rno-miR-199a-3p|rno-miR-378a-3p
NM_001305427	1	rno-miR-145-5p
NM_001106618	4	rno-miR-145-5p|rno-miR-3473|rno-miR-27b-3p|rno-miR-27a-3p
NM_001289832	15	rno-miR-145-5p|rno-miR-16-5p|rno-miR-103-3p|rno-miR-107-3p|rno-miR-195-5p|rno-miR-378a-3p|rno-miR-205|rno-miR-29a-3p|rno-miR-200b-3p|rno-miR-31a-5p|rno-miR-30c-5p|rno-miR-17-5p|rno-miR-152-3p|rno-miR-15b-5p|rno-miR-93-5p
NM_052809	1	rno-miR-145-5p
NM_001108435	2	rno-miR-145-5p|rno-miR-6216

NM_001108380	3	rno-miR-145-5p|rno-miR-26a-5p|rno-miR-205
NM_145677	6	rno-miR-145-5p|rno-miR-16-5p|rno-miR-27b-3p|rno-miR-195-5p|rno-miR-27a-3p|rno-miR-15b-5p
NM_031707	6	rno-miR-145-5p|rno-miR-26a-5p|rno-miR-6216|rno-miR-22-3p|rno-miR-31a-5p|rno-miR-152-3p
NM_013130	4	rno-miR-145-5p|rno-miR-26a-5p|rno-miR-205|rno-miR-30c-5p
NM_133423	8	rno-miR-145-5p|rno-miR-16-5p|rno-miR-103-3p|rno-miR-107-3p|rno-miR-195-5p|rno-miR-30c-5p|rno-miR-152-3p|rno-miR-15b-5p
NM_001024752	1	rno-miR-145-5p
NM_001098793	2	rno-miR-145-5p|rno-miR-24-3p
NM_001106818	2	rno-miR-145-5p|rno-miR-6216
NM_031685	4	rno-miR-145-5p|rno-miR-27b-3p|rno-miR-27a-3p|rno-miR-200b-3p
NM_001107809	12	rno-miR-145-5p|rno-let-7c-5p|rno-let-7b-5p|rno-miR-24-3p|rno-let-7d-5p|rno-let-7a-5p|rno-let-7e-5p|rno-let-7i-5p|rno-miR-29a-3p|rno-miR-17-5p|rno-let-7f-5p|rno-miR-93-5p
NM_053596	1	rno-miR-145-5p
NM_001106410	4	rno-miR-145-5p|rno-miR-320-3p|rno-miR-22-3p|rno-miR-181a-5p
NM_001305276	2	rno-miR-145-5p|rno-miR-152-3p
NM_001191664	3	rno-miR-145-5p|rno-miR-125b-5p|rno-miR-125a-5p
NM_019306	4	rno-miR-145-5p|rno-miR-200b-3p|rno-miR-181a-5p|rno-miR-152-3p
NM_001034958	9	rno-miR-145-5p|rno-let-7c-5p|rno-let-7b-5p|rno-let-7d-5p|rno-let-7a-5p|rno-let-7e-5p|rno-let-7i-5p|rno-miR-152-3p|rno-let-7f-5p
NM_172325	1	rno-miR-145-5p
NM_001135017	1	rno-miR-145-5p
NM_139336	3	rno-miR-145-5p|rno-miR-17-5p|rno-miR-93-5p
NM_001106230	1	rno-miR-145-5p
NM_133306	12	rno-miR-145-5p|rno-let-7c-5p|rno-let-7b-5p|rno-let-7d-5p|rno-let-7a-5p|rno-let-7e-5p|rno-miR-6216|rno-let-7i-5p|rno-miR-29a-3p|rno-miR-17-5p|rno-let-7f-5p|rno-miR-93-5p
NM_001289943	1	rno-miR-145-5p

NM_001014028	6	rno-miR-145-5p|rno-miR-143-3p|rno-miR-27b-3p|rno-miR-205|rno-miR-27a-3p|rno-miR-30c-5p
NM_012923	5	rno-miR-145-5p|rno-miR-23a-3p|rno-miR-23b-3p|rno-miR-27b-3p|rno-miR-27a-3p
NM_001127449	4	rno-miR-145-5p|rno-miR-103-3p|rno-miR-107-3p|rno-miR-31a-5p
NM_001004099	1	rno-miR-145-5p
NM_001106269	1	rno-miR-145-5p
NM_001162897	2	rno-miR-145-5p|rno-miR-22-3p
NM_001271065	1	rno-miR-145-5p
NM_001007147	1	rno-miR-145-5p
NM_001270413	2	rno-miR-145-5p|rno-miR-3473
NM_001191555	12	rno-miR-145-5p|rno-miR-26a-5p|rno-miR-6216|rno-miR-16-5p|rno-miR-27b-3p|rno-miR-195-5p|rno-miR-205|rno-miR-27a-3p|rno-miR-30c-5p|rno-miR-17-5p|rno-miR-15b-5p|rno-miR-93-5p
NM_021856	13	rno-miR-145-5p|rno-miR-16-5p|rno-miR-103-3p|rno-miR-107-3p|rno-miR-195-5p|rno-miR-378a-3p|rno-miR-205|rno-miR-29a-3p|rno-miR-200b-3p|rno-miR-31a-5p|rno-miR-17-5p|rno-miR-15b-5p|rno-miR-93-5p
NM_001017443	1	rno-miR-145-5p
NM_013048	6	rno-miR-145-5p|rno-miR-143-3p|rno-miR-26a-5p|rno-miR-27b-3p|rno-miR-27a-3p|rno-miR-30c-5p
NM_001106082	1	rno-miR-145-5p
NM_001309455	8	rno-miR-145-5p|rno-miR-23a-3p|rno-miR-23b-3p|rno-miR-24-3p|rno-miR-27b-3p|rno-miR-27a-3p|rno-miR-181a-5p|rno-miR-30c-5p
NM_001106393	3	rno-miR-145-5p|rno-miR-125b-5p|rno-miR-125a-5p
NM_001136470	13	rno-miR-145-5p|rno-let-7c-5p|rno-let-7b-5p|rno-miR-24-3p|rno-let-7d-5p|rno-let-7a-5p|rno-let-7e-5p|rno-miR-16-5p|rno-let-7i-5p|rno-miR-195-5p|rno-miR-30c-5p|rno-let-7f-5p|rno-miR-15b-5p
NM_001105814	1	rno-miR-145-5p
NM_001107194	2	rno-miR-145-5p|rno-miR-22-3p

NM_001013881	4	rno-miR-145-5p|rno-miR-16-5p|rno-miR-195-5p|rno-miR-15b-5p
NM_001130501	7	rno-miR-145-5p|rno-miR-103-3p|rno-miR-107-3p|rno-miR-378a-3p|rno-miR-200b-3p|rno-miR-17-5p|rno-miR-93-5p
NM_001191596	9	rno-miR-145-5p|rno-miR-6216|rno-miR-3473|rno-miR-320-3p|rno-miR-27b-3p|rno-miR-22-3p|rno-miR-27a-3p|rno-miR-17-5p|rno-miR-93-5p
NM_001134569	1	rno-miR-145-5p
NM_001106465	6	rno-miR-145-5p|rno-miR-22-3p|rno-miR-205|rno-miR-30c-5p|rno-miR-17-5p|rno-miR-93-5p
NM_001106281	2	rno-miR-145-5p|rno-miR-22-3p
NM_001106387	1	rno-miR-145-5p
NM_001108210	1	rno-miR-145-5p
NM_080403	2	rno-miR-145-5p|rno-miR-30c-5p
NM_001025036	1	rno-miR-145-5p
NM_001106744	9	rno-miR-145-5p|rno-miR-16-5p|rno-miR-125b-5p|rno-miR-125a-5p|rno-miR-199a-3p|rno-miR-195-5p|rno-miR-205|rno-miR-152-3p|rno-miR-15b-5p
NM_031552	5	rno-miR-145-5p|rno-miR-143-3p|rno-miR-27b-3p|rno-miR-27a-3p|rno-miR-200b-3p
NM_031749	2	rno-miR-145-5p|rno-miR-143-3p
NM_001107260	1	rno-miR-145-5p
NM_001110151	7	rno-miR-145-5p|rno-miR-143-3p|rno-miR-23a-3p|rno-miR-23b-3p|rno-miR-3473|rno-miR-205|rno-miR-152-3p
NM_031032	4	rno-miR-145-5p|rno-miR-320-3p|rno-miR-200b-3p|rno-miR-152-3p
NM_001012181	4	rno-miR-145-5p|rno-miR-23a-3p|rno-miR-23b-3p|rno-miR-29a-3p
NM_130423	3	rno-miR-145-5p|rno-miR-125b-5p|rno-miR-125a-5p
NM_001191692	5	rno-miR-145-5p|rno-miR-143-3p|rno-miR-26a-5p|rno-miR-22-3p|rno-miR-29a-3p
NM_053683	1	rno-miR-145-5p
NM_001108970	6	rno-miR-145-5p|rno-miR-16-5p|rno-miR-195-5p|rno-miR-205|rno-miR-15b-5p|rno-miR-151-5p

NM_001108552	4	rno-miR-145-5p|rno-miR-6216|rno-miR-200b-3p|rno-miR-181a-5p
NM_017300	1	rno-miR-145-5p
NM_012971	6	rno-miR-145-5p|rno-miR-27b-3p|rno-miR-378a-3p|rno-miR-27a-3p|rno-miR-181a-5p|rno-miR-30c-5p
NM_001107122	2	rno-miR-145-5p|rno-miR-199a-3p
NM_001033653	5	rno-miR-145-5p|rno-miR-23a-3p|rno-miR-23b-3p|rno-miR-103-3p|rno-miR-107-3p
NM_019224	1	rno-miR-145-5p
NM_001108446	3	rno-miR-145-5p|rno-miR-6216|rno-miR-29a-3p
NM_001106856	1	rno-miR-145-5p
NM_001109510	1	rno-miR-145-5p
NM_001012152	2	rno-miR-145-5p|rno-miR-181a-5p
NM_019621	1	rno-miR-145-5p
NM_012870	2	rno-miR-145-5p|rno-miR-181a-5p
NM_001004282	1	rno-miR-145-5p
NM_001191702	9	rno-miR-145-5p|rno-miR-26a-5p|rno-miR-23a-3p|rno-miR-23b-3p|rno-miR-16-5p|rno-miR-195-5p|rno-miR-200b-3p|rno-miR-181a-5p|rno-miR-15b-5p
NM_001271214	3	rno-miR-145-5p|rno-miR-17-5p|rno-miR-93-5p
NM_016989	2	rno-miR-145-5p|rno-miR-6216
NM_001106613	8	rno-miR-145-5p|rno-miR-23a-3p|rno-miR-23b-3p|rno-miR-6216|rno-miR-199a-3p|rno-miR-27b-3p|rno-miR-27a-3p|rno-miR-200b-3p
NM_001170547	2	rno-miR-145-5p|rno-miR-143-3p
NM_001170487	1	rno-miR-145-5p
NM_001015012	4	rno-miR-145-5p|rno-miR-29a-3p|rno-miR-17-5p|rno-miR-93-5p
NM_012555	6	rno-miR-145-5p|rno-miR-125b-5p|rno-miR-125a-5p|rno-miR-200b-3p|rno-miR-181a-5p|rno-miR-152-3p
NM_001109291	3	rno-miR-145-5p|rno-miR-24-3p|rno-miR-22-3p
NM_001191597	4	rno-miR-145-5p|rno-miR-23a-3p|rno-miR-23b-3p|rno-miR-320-3p

NM_001033963	8	rno-miR-145-5p|rno-miR-6216|rno-miR-199a-3p|rno-miR-27b-3p|rno-miR-27a-3p|rno-miR-17-5p|rno-miR-152-3p|rno-miR-93-5p
NM_001100760	5	rno-miR-145-5p|rno-miR-125b-5p|rno-miR-125a-5p|rno-miR-17-5p|rno-miR-93-5p
NM_001106933	3	rno-miR-145-5p|rno-miR-200b-3p|rno-miR-30c-5p
NM_022706	4	rno-miR-145-5p|rno-miR-6216|rno-miR-200b-3p|rno-miR-30c-5p
NM_001100665	1	rno-miR-145-5p
NM_001107357	15	rno-miR-145-5p|rno-let-7c-5p|rno-let-7b-5p|rno-let-7d-5p|rno-let-7a-5p|rno-let-7e-5p|rno-miR-16-5p|rno-let-7i-5p|rno-miR-27b-3p|rno-miR-195-5p|rno-miR-27a-3p|rno-miR-17-5p|rno-let-7f-5p|rno-miR-15b-5p|rno-miR-93-5p
NM_013044	3	rno-miR-145-5p|rno-miR-23a-3p|rno-miR-23b-3p
NM_017343	1	rno-miR-145-5p
NM_138848	1	rno-miR-145-5p
NM_001107416	9	rno-miR-145-5p|rno-miR-23a-3p|rno-miR-23b-3p|rno-miR-6216|rno-miR-320-3p|rno-miR-205|rno-miR-200b-3p|rno-miR-181a-5p|rno-miR-30c-5p
NM_001107939	4	rno-miR-145-5p|rno-miR-16-5p|rno-miR-195-5p|rno-miR-15b-5p
NM_133395	4	rno-miR-145-5p|rno-miR-23a-3p|rno-miR-23b-3p|rno-miR-29a-3p
NM_001100806	5	rno-miR-145-5p|rno-miR-143-3p|rno-miR-24-3p|rno-miR-29a-3p|rno-miR-200b-3p
NM_001106947	1	rno-miR-145-5p
NM_001107611	5	rno-miR-145-5p|rno-miR-6216|rno-miR-125b-5p|rno-miR-125a-5p|rno-miR-200b-3p
NM_001169133	2	rno-miR-145-5p|rno-miR-30c-5p
NM_019343	3	rno-miR-145-5p|rno-miR-6216|rno-miR-205
NM_001107659	4	rno-miR-145-5p|rno-miR-143-3p|rno-miR-17-5p|rno-miR-93-5p
NM_001106683	4	rno-miR-145-5p|rno-miR-27b-3p|rno-miR-27a-3p|rno-miR-152-3p
NM_001106762	4	rno-miR-145-5p|rno-miR-16-5p|rno-miR-195-5p|rno-miR-15b-5p
NM_001037790	1	rno-miR-145-5p

NM_001134597	13	rno-miR-145-5p|rno-let-7c-5p|rno-let-7b-5p|rno-let-7d-5p|rno-let-7a-5p|rno-let-7e-5p|rno-miR-6216|rno-miR-16-5p|rno-let-7i-5p|rno-miR-195-5p|rno-miR-152-3p|rno-let-7f-5p|rno-miR-15b-5p
NM_001191883	1	rno-miR-145-5p
NM_001168000	7	rno-miR-145-5p|rno-miR-26a-5p|rno-miR-27b-3p|rno-miR-27a-3p|rno-miR-200b-3p|rno-miR-181a-5p|rno-miR-30c-5p
NM_053381	8	rno-miR-145-5p|rno-miR-6216|rno-miR-16-5p|rno-miR-125b-5p|rno-miR-125a-5p|rno-miR-195-5p|rno-miR-29a-3p|rno-miR-15b-5p
NM_001011997	2	rno-miR-145-5p|rno-miR-200b-3p
NM_001002290	2	rno-miR-145-5p|rno-miR-22-3p
NM_001127300	1	rno-miR-145-5p
NM_013016	3	rno-miR-145-5p|rno-miR-23a-3p|rno-miR-23b-3p
NM_001271384	5	rno-miR-145-5p|rno-miR-143-3p|rno-miR-103-3p|rno-miR-107-3p|rno-miR-200b-3p
NM_001107786	1	rno-miR-145-5p
NM_001261404	3	rno-miR-145-5p|rno-miR-181a-5p|rno-miR-152-3p
NM_001109038	5	rno-miR-145-5p|rno-miR-26a-5p|rno-miR-23a-3p|rno-miR-23b-3p|rno-miR-3473
NM_001134581	3	rno-miR-145-5p|rno-miR-26a-5p|rno-miR-181a-5p
NM_001271317	4	rno-miR-145-5p|rno-miR-320-3p|rno-miR-27b-3p|rno-miR-27a-3p
NM_001107805	1	rno-miR-145-5p
NM_001109267	1	rno-miR-145-5p
NM_001109177	3	rno-miR-145-5p|rno-miR-24-3p|rno-miR-181a-5p
NM_001108953	2	rno-miR-145-5p|rno-miR-200b-3p
NM_012940	1	rno-miR-145-5p
NM_001164103	5	rno-miR-145-5p|rno-miR-143-3p|rno-miR-27b-3p|rno-miR-27a-3p|rno-miR-200b-3p
NM_017037	2	rno-miR-145-5p|rno-miR-29a-3p
NM_001134568	6	rno-miR-145-5p|rno-miR-26a-5p|rno-let-7b-5p|rno-let-7i-5p|rno-miR-27b-3p|rno-miR-27a-3p

NM_001012147	2	rno-miR-145-5p|rno-miR-30c-5p
NM_182736	1	rno-miR-145-5p
NM_134414	1	rno-miR-145-5p
NM_001107032	4	rno-miR-145-5p|rno-miR-17-5p|rno-miR-152-3p|rno-miR-93-5p
NM_053883	4	rno-miR-145-5p|rno-miR-125b-5p|rno-miR-125a-5p|rno-miR-181a-5p
NM_001107213	6	rno-miR-145-5p|rno-miR-23a-3p|rno-miR-23b-3p|rno-miR-27b-3p|rno-miR-27a-3p|rno-miR-200b-3p
NM_001012166	1	rno-miR-145-5p
NM_181384	4	rno-miR-145-5p|rno-miR-16-5p|rno-miR-195-5p|rno-miR-15b-5p
NM_001025422	5	rno-miR-145-5p|rno-miR-16-5p|rno-miR-320-3p|rno-miR-195-5p|rno-miR-15b-5p
NM_001107216	2	rno-miR-145-5p|rno-miR-152-3p
NM_001100672	3	rno-miR-145-5p|rno-miR-26a-5p|rno-miR-6216
NM_001033903	1	rno-miR-145-5p
NM_080887	1	rno-miR-145-5p
NM_001173388	3	rno-miR-145-5p|rno-miR-23a-3p|rno-miR-23b-3p
NM_001011922	6	rno-miR-145-5p|rno-miR-6216|rno-miR-125b-5p|rno-miR-103-3p|rno-miR-125a-5p|rno-miR-107-3p
NM_152847	8	rno-miR-145-5p|rno-miR-143-3p|rno-miR-23a-3p|rno-miR-23b-3p|rno-miR-3473|rno-miR-195-5p|rno-miR-205|rno-miR-152-3p
NM_001037978	1	rno-miR-145-5p
NM_017298	1	rno-miR-145-5p
NM_001008346	2	rno-miR-145-5p|rno-miR-143-3p
NM_001100679	3	rno-miR-145-5p|rno-miR-31a-5p|rno-miR-152-3p
NM_001108355	18	rno-miR-145-5p|rno-let-7c-5p|rno-let-7b-5p|rno-miR-23a-3p|rno-miR-23b-3p|rno-let-7d-5p|rno-let-7a-5p|rno-let-7e-5p|rno-miR-6216|rno-miR-16-5p|rno-let-7i-5p|rno-miR-195-5p|rno-miR-200b-3p|rno-miR-181a-5p|rno-miR-17-5p|rno-let-7f-5p|rno-miR-15b-5p|rno-miR-93-5p
NM_078620	1	rno-miR-145-5p
NM_017182	3	rno-miR-145-5p|rno-miR-30c-5p|rno-miR-152-3p

NM_001109645	4	rno-miR-145-5p|rno-miR-199a-3p|rno-miR-181a-5p|rno-miR-30c-5p
NM_001197241	12	rno-miR-145-5p|rno-miR-26a-5p|rno-miR-6216|rno-miR-16-5p|rno-miR-27b-3p|rno-miR-195-5p|rno-miR-205|rno-miR-27a-3p|rno-miR-30c-5p|rno-miR-17-5p|rno-miR-15b-5p|rno-miR-93-5p
NM_017350	1	rno-miR-145-5p
NM_001127533	4	rno-miR-145-5p|rno-miR-16-5p|rno-miR-195-5p|rno-miR-15b-5p
NM_001007608	4	rno-miR-145-5p|rno-miR-103-3p|rno-miR-107-3p|rno-miR-152-3p
NM_001107940	2	rno-miR-145-5p|rno-miR-181a-5p
NM_001109624	1	rno-miR-145-5p
NM_031326	1	rno-miR-145-5p
NM_001127684	5	rno-miR-145-5p|rno-miR-6216|rno-miR-16-5p|rno-miR-195-5p|rno-miR-15b-5p
NM_001013127	1	rno-miR-145-5p
NM_001017505	2	rno-miR-145-5p|rno-miR-6216
NM_001109533	4	rno-miR-145-5p|rno-miR-191a-5p|rno-miR-320-3p|rno-miR-181a-5p
NM_017082	1	rno-miR-145-5p
NM_001109452	1	rno-miR-145-5p
NM_001017381	1	rno-miR-145-5p
NM_012578	3	rno-miR-145-5p|rno-miR-3473|rno-miR-205
NM_001107985	1	rno-miR-145-5p
NM_001134503	5	rno-miR-145-5p|rno-miR-26a-5p|rno-miR-6216|rno-miR-205|rno-miR-200b-3p
NM_001106849	10	rno-miR-145-5p|rno-miR-23a-3p|rno-miR-23b-3p|rno-miR-6216|rno-miR-16-5p|rno-miR-125b-5p|rno-miR-125a-5p|rno-miR-195-5p|rno-miR-29a-3p|rno-miR-15b-5p
NM_001191614	1	rno-miR-145-5p
NM_001106904	13	rno-miR-145-5p|rno-let-7c-5p|rno-let-7b-5p|rno-miR-23a-3p|rno-miR-23b-3p|rno-let-7d-5p|rno-let-7a-5p|rno-let-7e-5p|rno-let-7i-5p|rno-miR-29a-3p|rno-miR-200b-3p|rno-miR-181a-5p|rno-let-7f-5p
NM_134352	1	rno-miR-145-5p

NM_001107797	8	rno-miR-143-3p|rno-miR-26a-5p|rno-miR-16-5p|rno-miR-27b-3p|rno-miR-195-5p|rno-miR-27a-3p|rno-miR-31a-5p|rno-miR-15b-5p
NM_139325	1	rno-miR-143-3p
NM_001024344	1	rno-miR-143-3p
NM_001305281	5	rno-miR-143-3p|rno-miR-16-5p|rno-miR-195-5p|rno-miR-30c-5p|rno-miR-15b-5p
NM_001173357	1	rno-miR-143-3p
NM_001134982	2	rno-miR-143-3p|rno-miR-26a-5p
NM_001079890	1	rno-miR-143-3p
NM_001024783	1	rno-miR-143-3p
NM_001009708	1	rno-miR-143-3p
NM_019191	2	rno-miR-143-3p|rno-miR-152-3p
NM_001191579	5	rno-miR-143-3p|rno-miR-16-5p|rno-miR-195-5p|rno-miR-200b-3p|rno-miR-15b-5p
NM_199407	6	rno-miR-143-3p|rno-miR-16-5p|rno-miR-195-5p|rno-miR-205|rno-miR-30c-5p|rno-miR-15b-5p
NM_033299	1	rno-miR-143-3p
NM_001025140	2	rno-miR-143-3p|rno-miR-6216
NM_001108168	5	rno-miR-143-3p|rno-miR-26a-5p|rno-miR-6216|rno-miR-181a-5p|rno-miR-30c-5p
NM_145081	3	rno-miR-143-3p|rno-miR-27b-3p|rno-miR-27a-3p
NM_012817	1	rno-miR-143-3p
NM_012958	1	rno-miR-143-3p
NM_024145	2	rno-miR-143-3p|rno-miR-31a-5p
NM_001109109	3	rno-miR-143-3p|rno-miR-27b-3p|rno-miR-27a-3p
NM_031986	1	rno-miR-143-3p
NM_001115036	1	rno-miR-143-3p
NM_001025281	1	rno-miR-143-3p
NM_001044294	1	rno-miR-143-3p

NM_001134712	7	rno-miR-143-3p|rno-miR-16-5p|rno-miR-103-3p|rno-miR-107-3p|rno-miR-199a-3p|rno-miR-195-5p|rno-miR-15b-5p
NM_001107811	2	rno-miR-143-3p|rno-miR-199a-3p
NM_001106466	2	rno-miR-143-3p|rno-miR-6216
NM_001271212	1	rno-miR-143-3p
NM_022618	10	rno-miR-143-3p|rno-let-7c-5p|rno-miR-26a-5p|rno-let-7b-5p|rno-let-7d-5p|rno-let-7a-5p|rno-let-7e-5p|rno-let-7i-5p|rno-miR-181a-5p|rno-let-7f-5p
NM_001100653	2	rno-miR-143-3p|rno-miR-29a-3p
NM_001033883	5	rno-miR-143-3p|rno-miR-23a-3p|rno-miR-23b-3p|rno-miR-125b-5p|rno-miR-125a-5p
NM_001106529	10	rno-miR-143-3p|rno-miR-24-3p|rno-miR-16-5p|rno-miR-103-3p|rno-miR-107-3p|rno-miR-195-5p|rno-miR-30c-5p|rno-miR-17-5p|rno-miR-15b-5p|rno-miR-93-5p
NM_001124769	1	rno-miR-143-3p
NM_001106592	9	rno-miR-143-3p|rno-let-7c-5p|rno-let-7b-5p|rno-let-7d-5p|rno-let-7a-5p|rno-let-7e-5p|rno-let-7i-5p|rno-miR-29a-3p|rno-let-7f-5p
NM_001025755	1	rno-miR-143-3p
NM_001106938	4	rno-miR-143-3p|rno-miR-320-3p|rno-miR-27b-3p|rno-miR-27a-3p
NM_181090	7	rno-miR-143-3p|rno-miR-26a-5p|rno-miR-6216|rno-miR-320-3p|rno-miR-205|rno-miR-200b-3p|rno-miR-30c-5p
NM_001012127	1	rno-miR-143-3p
NM_133581	1	rno-miR-143-3p
NM_001006963	2	rno-miR-143-3p|rno-miR-320-3p
NM_001108270	3	rno-miR-143-3p|rno-miR-23a-3p|rno-miR-23b-3p
NM_139184	1	rno-miR-143-3p
NM_134326	1	rno-miR-143-3p
NM_053484	9	rno-miR-143-3p|rno-let-7c-5p|rno-let-7b-5p|rno-let-7d-5p|rno-let-7a-5p|rno-let-7e-5p|rno-miR-6216|rno-let-7i-5p|rno-let-7f-5p
NM_001008287	3	rno-miR-143-3p|rno-miR-6216|rno-miR-200b-3p

NM_022597	1	rno-miR-143-3p
NM_001107338	5	rno-miR-143-3p|rno-miR-16-5p|rno-miR-195-5p|rno-miR-29a-3p|rno-miR-15b-5p
NM_001195560	1	rno-miR-143-3p
NM_001100789	4	rno-miR-143-3p|rno-miR-26a-5p|rno-miR-200b-3p|rno-miR-30c-5p
NM_012610	4	rno-miR-143-3p|rno-miR-6216|rno-miR-27b-3p|rno-miR-27a-3p
NM_031725	1	rno-miR-143-3p
NM_019259	1	rno-miR-143-3p
NM_001029919	6	rno-miR-143-3p|rno-miR-27b-3p|rno-miR-22-3p|rno-miR-205|rno-miR-27a-3p|rno-miR-30c-5p
NM_138855	1	rno-miR-143-3p
NM_012666	1	rno-miR-143-3p
NM_001106831	2	rno-miR-143-3p|rno-miR-6216
NM_001031647	1	rno-miR-143-3p
NM_001134690	1	rno-miR-143-3p
NM_001039026	3	rno-miR-143-3p|rno-miR-103-3p|rno-miR-107-3p
NM_012569	7	rno-miR-143-3p|rno-miR-23a-3p|rno-miR-23b-3p|rno-miR-6216|rno-miR-125b-5p|rno-miR-125a-5p|rno-miR-181a-5p
NM_001107107	4	rno-miR-143-3p|rno-miR-320-3p|rno-miR-22-3p|rno-miR-205
NM_001177368	1	rno-miR-143-3p
NM_001109611	2	rno-miR-143-3p|rno-miR-26a-5p
NM_013047	6	rno-miR-143-3p|rno-miR-23a-3p|rno-miR-23b-3p|rno-miR-6216|rno-miR-30c-5p|rno-miR-152-3p
NM_001107118	4	rno-miR-143-3p|rno-miR-26a-5p|rno-miR-17-5p|rno-miR-93-5p
NM_001011933	1	rno-miR-143-3p
NM_012744	2	rno-miR-143-3p|rno-miR-31a-5p
NM_001109039	2	rno-miR-143-3p|rno-miR-22-3p
NM_001013244	2	rno-miR-143-3p|rno-miR-181a-5p
NM_001277450	2	rno-miR-143-3p|rno-miR-152-3p
NM_001106699	2	rno-miR-143-3p|rno-miR-181a-5p

NM_001271206	11	rno-miR-143-3p|rno-let-7c-5p|rno-let-7b-5p|rno-let-7d-5p|rno-let-7a-5p|rno-let-7e-5p|rno-let-7i-5p|rno-miR-17-5p|rno-miR-152-3p|rno-let-7f-5p|rno-miR-93-5p
NM_017323	3	rno-miR-143-3p|rno-miR-17-5p|rno-miR-93-5p
NM_012823	2	rno-miR-143-3p|rno-miR-26a-5p
NM_001029897	1	rno-miR-143-3p
NM_001108941	8	rno-miR-143-3p|rno-miR-16-5p|rno-miR-195-5p|rno-miR-378a-3p|rno-miR-181a-5p|rno-miR-30c-5p|rno-miR-152-3p|rno-miR-15b-5p
NM_001025645	1	rno-miR-143-3p
NM_001012358	4	rno-miR-143-3p|rno-miR-26a-5p|rno-miR-200b-3p|rno-miR-181a-5p
NM_001172150	3	rno-miR-143-3p|rno-miR-26a-5p|rno-miR-151-5p
NM_001169130	2	rno-miR-143-3p|rno-miR-24-3p
NM_001113544	1	rno-miR-143-3p
NM_001191100	8	rno-miR-143-3p|rno-miR-26a-5p|rno-miR-6216|rno-miR-125b-5p|rno-miR-103-3p|rno-miR-125a-5p|rno-miR-107-3p|rno-miR-152-3p
NM_001012183	3	rno-miR-143-3p|rno-miR-17-5p|rno-miR-93-5p
NM_001109286	1	rno-miR-143-3p
NM_001013922	2	rno-miR-143-3p|rno-miR-26a-5p
NM_001100657	1	rno-miR-143-3p
NM_001024772	2	rno-miR-143-3p|rno-miR-205
NM_001106142	1	rno-miR-143-3p
NM_001105942	1	rno-miR-143-3p
NM_001011979	1	rno-miR-143-3p
NM_144739	1	rno-miR-143-3p
NM_001134797	1	rno-miR-143-3p
NM_019344	14	rno-miR-143-3p|rno-let-7c-5p|rno-let-7b-5p|rno-let-7a-5p|rno-let-7e-5p|rno-let-7i-5p|rno-miR-27b-3p|rno-miR-205|rno-miR-27a-3p|rno-miR-30c-5p|rno-miR-17-5p|rno-miR-152-3p|rno-let-7f-5p|rno-miR-93-5p

NM_001106901	6	rno-miR-143-3p|rno-miR-16-5p|rno-miR-195-5p|rno-miR-17-5p|rno-miR-15b-5p|rno-miR-93-5p
NM_182952	1	rno-miR-143-3p
NM_001047857	2	rno-miR-143-3p|rno-miR-6216
NM_001130541	2	rno-miR-143-3p|rno-miR-181a-5p
NM_001108948	1	rno-miR-143-3p
NM_001124770	1	rno-miR-143-3p
NM_001173556	1	rno-miR-143-3p
NM_001134640	1	rno-miR-143-3p
NM_032067	2	rno-miR-143-3p|rno-miR-152-3p
NM_001106577	5	rno-miR-143-3p|rno-miR-200b-3p|rno-miR-30c-5p|rno-miR-17-5p|rno-miR-93-5p
NM_001108140	1	rno-miR-143-3p
NM_080398	2	rno-miR-143-3p|rno-miR-152-3p
NM_001143803	6	rno-miR-143-3p|rno-miR-23a-3p|rno-miR-23b-3p|rno-miR-16-5p|rno-miR-195-5p|rno-miR-15b-5p
NM_080583	1	rno-miR-143-3p
NM_001107715	1	rno-miR-143-3p
NM_053787	6	rno-miR-143-3p|rno-miR-16-5p|rno-miR-27b-3p|rno-miR-195-5p|rno-miR-27a-3p|rno-miR-15b-5p
NM_001271366	1	rno-miR-143-3p
NM_013153	16	rno-miR-143-3p|rno-let-7c-5p|rno-let-7b-5p|rno-miR-23a-3p|rno-miR-23b-3p|rno-let-7d-5p|rno-let-7a-5p|rno-let-7e-5p|rno-miR-16-5p|rno-let-7i-5p|rno-miR-195-5p|rno-miR-181a-5p|rno-miR-17-5p|rno-let-7f-5p|rno-miR-15b-5p|rno-miR-93-5p
NM_001305170	2	rno-miR-143-3p|rno-miR-26a-5p
NM_001014275	1	rno-miR-143-3p
NM_022266	2	rno-miR-143-3p|rno-miR-26a-5p
NM_001025418	6	rno-miR-143-3p|rno-miR-16-5p|rno-miR-195-5p|rno-miR-30c-5p|rno-miR-152-3p|rno-miR-15b-5p

NM_053539	2	rno-miR-143-3p|rno-miR-320-3p
NM_001014251	2	rno-miR-143-3p|rno-miR-320-3p
NM_001105886	2	rno-miR-143-3p|rno-miR-26a-5p
NM_021868	1	rno-miR-143-3p
NM_001009825	1	rno-miR-143-3p
NM_001106271	8	rno-miR-143-3p|rno-miR-16-5p|rno-miR-3473|rno-miR-195-5p|rno-miR-29a-3p|rno-miR-17-5p|rno-miR-15b-5p|rno-miR-93-5p
NM_001012186	2	rno-miR-143-3p|rno-miR-205
NM_001106739	7	rno-miR-143-3p|rno-miR-23a-3p|rno-miR-23b-3p|rno-miR-103-3p|rno-miR-107-3p|rno-miR-181a-5p|rno-miR-30c-5p
NM_024368	8	rno-miR-143-3p|rno-miR-26a-5p|rno-miR-27b-3p|rno-miR-205|rno-miR-27a-3p|rno-miR-181a-5p|rno-miR-30c-5p|rno-miR-152-3p
NM_001141935	1	rno-miR-143-3p
NM_001014206	1	rno-miR-143-3p
NM_001100570	3	rno-miR-143-3p|rno-miR-24-3p|rno-miR-22-3p
NM_001108634	1	rno-miR-143-3p
NM_001271791	5	rno-miR-143-3p|rno-miR-26a-5p|rno-miR-23a-3p|rno-miR-23b-3p|rno-miR-181a-5p
NM_053991	4	rno-miR-143-3p|rno-miR-27b-3p|rno-miR-27a-3p|rno-miR-30c-5p
NM_001106386	4	rno-miR-143-3p|rno-miR-30c-5p|rno-miR-17-5p|rno-miR-93-5p
NM_001108827	3	rno-miR-143-3p|rno-miR-23a-3p|rno-miR-23b-3p
NM_001109099	12	rno-miR-143-3p|rno-let-7c-5p|rno-let-7b-5p|rno-let-7d-5p|rno-let-7a-5p|rno-let-7e-5p|rno-miR-6216|rno-let-7i-5p|rno-miR-320-3p|rno-miR-17-5p|rno-let-7f-5p|rno-miR-93-5p
NM_001107920	4	rno-miR-143-3p|rno-miR-6216|rno-miR-103-3p|rno-miR-107-3p
NM_001308239	1	rno-miR-143-3p
NM_001109643	1	rno-miR-143-3p
NM_053516	1	rno-miR-143-3p
NM_001077677	2	rno-miR-143-3p|rno-miR-3473
NM_031350	2	rno-miR-143-3p|rno-miR-30c-5p

NM_001191073	1	rno-miR-143-3p
NM_001108785	1	rno-miR-143-3p
NM_021671	10	rno-miR-143-3p|rno-miR-26a-5p|rno-miR-16-5p|rno-miR-103-3p|rno-miR-107-3p|rno-miR-27b-3p|rno-miR-22-3p|rno-miR-195-5p|rno-miR-27a-3p|rno-miR-15b-5p
NM_001124768	1	rno-miR-143-3p
NM_001108494	1	rno-miR-143-3p
NM_001134711	7	rno-miR-143-3p|rno-miR-16-5p|rno-miR-103-3p|rno-miR-107-3p|rno-miR-199a-3p|rno-miR-195-5p|rno-miR-15b-5p
NM_001108961	2	rno-miR-143-3p|rno-miR-26a-5p
NM_001127377	5	rno-miR-143-3p|rno-miR-23a-3p|rno-miR-23b-3p|rno-miR-27b-3p|rno-miR-27a-3p
NM_012802	8	rno-miR-143-3p|rno-miR-24-3p|rno-miR-199a-3p|rno-miR-27b-3p|rno-miR-27a-3p|rno-miR-181a-5p|rno-miR-17-5p|rno-miR-93-5p
NM_024155	1	rno-miR-143-3p
NM_001106028	1	rno-miR-143-3p
NM_001170470	1	rno-miR-143-3p
NM_001128099	2	rno-miR-143-3p|rno-miR-6216
NM_001011913	1	rno-miR-143-3p
NM_001108511	6	rno-miR-143-3p|rno-miR-23a-3p|rno-miR-23b-3p|rno-miR-27b-3p|rno-miR-27a-3p|rno-miR-30c-5p
NM_001145175	1	rno-miR-143-3p
NM_031320	3	rno-miR-143-3p|rno-miR-320-3p|rno-miR-30c-5p
NM_001134509	4	rno-miR-143-3p|rno-miR-16-5p|rno-miR-195-5p|rno-miR-15b-5p
NM_001100640	2	rno-miR-143-3p|rno-miR-6216
NM_031727	5	rno-miR-143-3p|rno-miR-27b-3p|rno-miR-27a-3p|rno-miR-17-5p|rno-miR-93-5p

NM_001100887	20	rno-miR-143-3p|rno-let-7c-5p|rno-let-7b-5p|rno-miR-23a-3p|rno-miR-23b-3p|rno-let-7d-5p|rno-let-7a-5p|rno-let-7e-5p|rno-miR-6216|rno-miR-125b-5p|rno-let-7i-5p|rno-miR-125a-5p|rno-miR-320-3p|rno-miR-27b-3p|rno-miR-205|rno-miR-27a-3p|rno-miR-31a-5p|rno-miR-17-5p|rno-let-7f-5p|rno-miR-93-5p
NM_031810	1	rno-miR-143-3p
NM_139333	2	rno-miR-143-3p|rno-miR-24-3p
NM_001109433	1	rno-miR-143-3p
NM_001195277	17	rno-miR-143-3p|rno-let-7c-5p|rno-miR-26a-5p|rno-let-7b-5p|rno-let-7a-5p|rno-let-7e-5p|rno-miR-16-5p|rno-miR-103-3p|rno-let-7i-5p|rno-miR-107-3p|rno-miR-27b-3p|rno-miR-195-5p|rno-miR-27a-3p|rno-miR-29a-3p|rno-miR-152-3p|rno-let-7f-5p|rno-miR-15b-5p
NM_053304	3	rno-miR-143-3p|rno-miR-6216|rno-miR-29a-3p
NM_001033867	5	rno-miR-143-3p|rno-miR-6216|rno-miR-16-5p|rno-miR-195-5p|rno-miR-15b-5p
NM_001108488	1	rno-miR-143-3p
NM_001107673	12	rno-miR-143-3p|rno-let-7c-5p|rno-miR-26a-5p|rno-let-7b-5p|rno-let-7d-5p|rno-let-7a-5p|rno-let-7e-5p|rno-let-7i-5p|rno-miR-27b-3p|rno-miR-27a-3p|rno-miR-181a-5p|rno-let-7f-5p
NM_001109497	3	rno-miR-143-3p|rno-miR-26a-5p|rno-miR-30c-5p
NM_001197217	4	rno-miR-143-3p|rno-miR-125b-5p|rno-miR-125a-5p|rno-miR-30c-5p
NM_001134729	3	rno-miR-143-3p|rno-miR-17-5p|rno-miR-93-5p
NM_031081	2	rno-miR-143-3p|rno-miR-320-3p
NM_001034014	1	rno-miR-143-3p
NM_001013204	1	rno-miR-143-3p
NM_023950	1	rno-miR-143-3p
NM_001108198	1	rno-miR-143-3p
NM_001191547	2	rno-miR-143-3p|rno-miR-24-3p
NM_001106033	2	rno-miR-143-3p|rno-miR-24-3p
NM_001007756	2	rno-miR-143-3p|rno-miR-378a-3p

NM_053406	1	rno-miR-143-3p
NM_181373	2	rno-miR-143-3p|rno-miR-24-3p
NM_001106911	5	rno-miR-143-3p|rno-miR-27b-3p|rno-miR-27a-3p|rno-miR-181a-5p|rno-miR-30c-5p
NM_001108561	3	rno-miR-143-3p|rno-miR-24-3p|rno-miR-31a-5p
NM_012888	2	rno-miR-143-3p|rno-miR-24-3p
NM_022522	4	rno-miR-143-3p|rno-miR-125b-5p|rno-miR-125a-5p|rno-miR-200b-3p
NM_001014059	1	rno-miR-143-3p
NM_012987	1	rno-miR-143-3p
NM_001106964	3	rno-miR-143-3p|rno-miR-17-5p|rno-miR-93-5p
NM_001047116	4	rno-miR-143-3p|rno-miR-24-3p|rno-miR-205|rno-miR-30c-5p
NM_001134527	1	rno-miR-143-3p
NM_001108416	1	rno-miR-143-3p
NM_001105810	1	rno-miR-143-3p
NM_053343	7	rno-miR-143-3p|rno-miR-16-5p|rno-miR-103-3p|rno-miR-107-3p|rno-miR-195-5p|rno-miR-181a-5p|rno-miR-15b-5p
NM_001024262	2	rno-miR-143-3p|rno-miR-320-3p
NM_001112716	2	rno-miR-143-3p|rno-miR-24-3p
NM_139094	1	rno-miR-143-3p
NM_057207	4	rno-miR-143-3p|rno-miR-205|rno-miR-17-5p|rno-miR-93-5p
NM_001109321	5	rno-miR-143-3p|rno-miR-26a-5p|rno-miR-23a-3p|rno-miR-23b-3p|rno-miR-320-3p
NM_001037217	2	rno-miR-143-3p|rno-miR-200b-3p
NM_001113543	1	rno-miR-143-3p
NM_001108473	2	rno-miR-143-3p|rno-miR-22-3p
NM_023096	5	rno-miR-143-3p|rno-miR-125b-5p|rno-miR-125a-5p|rno-miR-22-3p|rno-miR-181a-5p
NM_053725	3	rno-miR-143-3p|rno-miR-199a-3p|rno-miR-30c-5p
NM_021760	2	rno-miR-143-3p|rno-miR-29a-3p
NM_173111	1	rno-miR-143-3p

NM_001017486	1	rno-miR-143-3p
NM_053455	4	rno-miR-143-3p|rno-miR-320-3p|rno-miR-378a-3p|rno-miR-181a-5p
NM_001106871	1	rno-miR-143-3p
NM_001130502	4	rno-miR-143-3p|rno-miR-29a-3p|rno-miR-31a-5p|rno-miR-30c-5p
NM_001025655	7	rno-miR-143-3p|rno-miR-16-5p|rno-miR-195-5p|rno-miR-29a-3p|rno-miR-17-5p|rno-miR-15b-5p|rno-miR-93-5p
NM_001014128	1	rno-miR-143-3p
NM_175760	3	rno-miR-143-3p|rno-miR-17-5p|rno-miR-93-5p
NM_031599	1	rno-miR-143-3p
NM_001044269	3	rno-miR-143-3p|rno-miR-17-5p|rno-miR-93-5p
NM_001135899	3	rno-miR-143-3p|rno-miR-23a-3p|rno-miR-23b-3p
NM_001134548	10	rno-miR-143-3p|rno-let-7c-5p|rno-let-7b-5p|rno-let-7a-5p|rno-let-7e-5p|rno-let-7i-5p|rno-miR-200b-3p|rno-miR-17-5p|rno-let-7f-5p|rno-miR-93-5p
NM_001185025	1	rno-miR-143-3p
NM_001025642	4	rno-miR-143-3p|rno-miR-27b-3p|rno-miR-22-3p|rno-miR-27a-3p
NM_031605	2	rno-miR-143-3p|rno-miR-320-3p
NM_001030036	1	rno-miR-143-3p
NM_001313815	11	rno-miR-143-3p|rno-miR-6216|rno-miR-16-5p|rno-miR-103-3p|rno-miR-107-3p|rno-miR-320-3p|rno-miR-195-5p|rno-miR-29a-3p|rno-miR-181a-5p|rno-miR-152-3p|rno-miR-15b-5p
NM_001012130	1	rno-miR-143-3p
NM_001191578	2	rno-miR-143-3p|rno-miR-200b-3p
NM_012765	5	rno-miR-143-3p|rno-miR-23a-3p|rno-miR-23b-3p|rno-miR-27b-3p|rno-miR-27a-3p
NM_001044273	1	rno-miR-143-3p
NM_001107832	1	rno-miR-143-3p
NM_181081	4	rno-miR-143-3p|rno-miR-24-3p|rno-miR-22-3p|rno-miR-152-3p
NM_001108653	6	rno-miR-143-3p|rno-miR-26a-5p|rno-miR-16-5p|rno-miR-195-5p|rno-miR-29a-3p|rno-miR-15b-5p

NM_022855	8	rno-miR-143-3p|rno-miR-23a-3p|rno-miR-23b-3p|rno-miR-6216|rno-miR-103-3p|rno-miR-107-3p|rno-miR-320-3p|rno-miR-200b-3p
NM_001134691	1	rno-miR-143-3p
NM_198733	4	rno-miR-143-3p|rno-miR-27b-3p|rno-miR-27a-3p|rno-miR-31a-5p
NM_001128187	3	rno-miR-143-3p|rno-miR-27b-3p|rno-miR-27a-3p
NM_001033656	3	rno-miR-143-3p|rno-miR-27b-3p|rno-miR-27a-3p
NM_053932	6	rno-miR-143-3p|rno-miR-26a-5p|rno-miR-24-3p|rno-miR-29a-3p|rno-miR-200b-3p|rno-miR-30c-5p
NM_001106305	3	rno-miR-143-3p|rno-miR-23a-3p|rno-miR-23b-3p
NM_001007621	1	rno-miR-143-3p
NM_032613	5	rno-miR-143-3p|rno-miR-3473|rno-miR-27b-3p|rno-miR-27a-3p|rno-miR-29a-3p
NM_212495	1	rno-miR-143-3p
NM_001024797	5	rno-miR-143-3p|rno-miR-27b-3p|rno-miR-27a-3p|rno-miR-17-5p|rno-miR-93-5p
NM_053603	3	rno-miR-143-3p|rno-miR-103-3p|rno-miR-107-3p
NM_001035255	2	rno-miR-143-3p|rno-miR-152-3p
NM_198786	4	rno-miR-143-3p|rno-miR-199a-3p|rno-miR-27b-3p|rno-miR-27a-3p
NM_172317	1	rno-miR-143-3p
NM_172243	5	rno-miR-143-3p|rno-miR-23a-3p|rno-miR-23b-3p|rno-miR-27b-3p|rno-miR-27a-3p
NM_001271137	8	rno-miR-143-3p|rno-let-7c-5p|rno-let-7b-5p|rno-let-7d-5p|rno-let-7a-5p|rno-let-7e-5p|rno-let-7i-5p|rno-let-7f-5p
NM_012614	3	rno-miR-143-3p|rno-miR-27b-3p|rno-miR-27a-3p
NM_001107792	4	rno-miR-143-3p|rno-miR-16-5p|rno-miR-195-5p|rno-miR-15b-5p
NM_001106334	1	rno-miR-143-3p
NM_001106885	1	rno-miR-143-3p
NM_001107054	1	rno-miR-143-3p

NM_022257	10	rno-let-7c-5p|rno-let-7b-5p|rno-let-7d-5p|rno-let-7a-5p|rno-let-7e-5p|rno-miR-16-5p|rno-let-7i-5p|rno-miR-195-5p|rno-let-7f-5p|rno-miR-15b-5p
NM_001105961	6	rno-let-7c-5p|rno-let-7b-5p|rno-let-7a-5p|rno-let-7e-5p|rno-let-7i-5p|rno-let-7f-5p
NM_001014026	7	rno-let-7c-5p|rno-let-7b-5p|rno-let-7d-5p|rno-let-7a-5p|rno-let-7e-5p|rno-let-7i-5p|rno-let-7f-5p
NM_001168633	8	rno-let-7c-5p|rno-let-7b-5p|rno-let-7d-5p|rno-let-7a-5p|rno-let-7e-5p|rno-let-7i-5p|rno-miR-181a-5p|rno-let-7f-5p
NM_001109199	7	rno-let-7c-5p|rno-let-7b-5p|rno-let-7d-5p|rno-let-7a-5p|rno-let-7e-5p|rno-let-7i-5p|rno-let-7f-5p
NM_001135576	10	rno-let-7c-5p|rno-let-7b-5p|rno-let-7d-5p|rno-let-7a-5p|rno-let-7e-5p|rno-miR-6216|rno-let-7i-5p|rno-miR-200b-3p|rno-miR-152-3p|rno-let-7f-5p
NM_024371	11	rno-let-7c-5p|rno-let-7b-5p|rno-let-7d-5p|rno-let-7a-5p|rno-let-7e-5p|rno-let-7i-5p|rno-miR-27b-3p|rno-miR-22-3p|rno-miR-27a-3p|rno-miR-200b-3p|rno-let-7f-5p
NM_031621	7	rno-let-7c-5p|rno-let-7b-5p|rno-let-7d-5p|rno-let-7a-5p|rno-let-7e-5p|rno-let-7i-5p|rno-let-7f-5p
NM_001106875	7	rno-let-7c-5p|rno-let-7b-5p|rno-let-7d-5p|rno-let-7a-5p|rno-let-7e-5p|rno-let-7i-5p|rno-let-7f-5p
NM_019211	8	rno-let-7c-5p|rno-let-7b-5p|rno-let-7d-5p|rno-let-7a-5p|rno-let-7e-5p|rno-miR-6216|rno-let-7i-5p|rno-let-7f-5p
NM_001108673	9	rno-let-7c-5p|rno-let-7b-5p|rno-let-7d-5p|rno-let-7a-5p|rno-let-7e-5p|rno-let-7i-5p|rno-miR-27b-3p|rno-miR-27a-3p|rno-let-7f-5p
NM_019146	9	rno-let-7c-5p|rno-let-7b-5p|rno-let-7d-5p|rno-let-7a-5p|rno-let-7e-5p|rno-miR-125b-5p|rno-let-7i-5p|rno-miR-125a-5p|rno-let-7f-5p
NM_001105910	7	rno-let-7c-5p|rno-let-7b-5p|rno-let-7d-5p|rno-let-7a-5p|rno-let-7e-5p|rno-let-7i-5p|rno-let-7f-5p

NM_001257347	7	rno-let-7c-5p|rno-let-7b-5p|rno-let-7d-5p|rno-let-7a-5p|rno-let-7e-5p|rno-let-7i-5p|rno-let-7f-5p
NM_133606	11	rno-let-7c-5p|rno-let-7b-5p|rno-let-7d-5p|rno-let-7a-5p|rno-let-7e-5p|rno-miR-16-5p|rno-let-7i-5p|rno-miR-199a-3p|rno-miR-195-5p|rno-let-7f-5p|rno-miR-15b-5p
NM_001100729	8	rno-let-7c-5p|rno-let-7b-5p|rno-let-7d-5p|rno-let-7a-5p|rno-let-7e-5p|rno-let-7i-5p|rno-miR-30c-5p|rno-let-7f-5p
NM_001108854	7	rno-let-7c-5p|rno-let-7b-5p|rno-let-7d-5p|rno-let-7a-5p|rno-let-7e-5p|rno-let-7i-5p|rno-let-7f-5p
NM_001011962	9	rno-let-7c-5p|rno-let-7b-5p|rno-let-7d-5p|rno-let-7a-5p|rno-let-7e-5p|rno-let-7i-5p|rno-miR-17-5p|rno-let-7f-5p|rno-miR-93-5p
NM_001037765	7	rno-let-7c-5p|rno-let-7b-5p|rno-let-7d-5p|rno-let-7a-5p|rno-let-7e-5p|rno-let-7i-5p|rno-let-7f-5p
NM_024163	7	rno-let-7c-5p|rno-let-7b-5p|rno-let-7d-5p|rno-let-7a-5p|rno-let-7e-5p|rno-let-7i-5p|rno-let-7f-5p
NM_031358	7	rno-let-7c-5p|rno-let-7b-5p|rno-let-7d-5p|rno-let-7a-5p|rno-let-7e-5p|rno-let-7i-5p|rno-let-7f-5p
NM_001271193	8	rno-let-7c-5p|rno-let-7b-5p|rno-let-7d-5p|rno-let-7a-5p|rno-let-7e-5p|rno-let-7i-5p|rno-miR-30c-5p|rno-let-7f-5p
NM_001034128	9	rno-let-7c-5p|rno-let-7b-5p|rno-let-7d-5p|rno-let-7a-5p|rno-let-7e-5p|rno-let-7i-5p|rno-miR-22-3p|rno-miR-30c-5p|rno-let-7f-5p
NM_001107865	7	rno-let-7c-5p|rno-let-7b-5p|rno-let-7d-5p|rno-let-7a-5p|rno-let-7e-5p|rno-let-7i-5p|rno-let-7f-5p
NM_001109376	12	rno-let-7c-5p|rno-let-7b-5p|rno-miR-23a-3p|rno-miR-23b-3p|rno-let-7d-5p|rno-let-7a-5p|rno-let-7e-5p|rno-let-7i-5p|rno-miR-30c-5p|rno-miR-17-5p|rno-let-7f-5p|rno-miR-93-5p
NM_001006981	7	rno-let-7c-5p|rno-let-7b-5p|rno-let-7d-5p|rno-let-7a-5p|rno-let-7e-5p|rno-let-7i-5p|rno-let-7f-5p
NM_001126283	8	rno-let-7c-5p|rno-let-7b-5p|rno-let-7d-5p|rno-let-7a-5p|rno-let-7e-5p|rno-let-7i-5p|rno-miR-181a-5p|rno-let-7f-5p

NM_001106813	9	rno-let-7c-5p|rno-let-7b-5p|rno-let-7d-5p|rno-let-7a-5p|rno-let-7e-5p|rno-let-7i-5p|rno-miR-27b-3p|rno-miR-27a-3p|rno-let-7f-5p
NM_012854	7	rno-let-7c-5p|rno-let-7b-5p|rno-let-7d-5p|rno-let-7a-5p|rno-let-7e-5p|rno-let-7i-5p|rno-let-7f-5p
NM_133407	8	rno-let-7c-5p|rno-let-7b-5p|rno-let-7d-5p|rno-let-7a-5p|rno-let-7e-5p|rno-let-7i-5p|rno-miR-200b-3p|rno-let-7f-5p
NM_001025125	7	rno-let-7c-5p|rno-let-7b-5p|rno-let-7d-5p|rno-let-7a-5p|rno-let-7e-5p|rno-let-7i-5p|rno-let-7f-5p
NM_172066	10	rno-let-7c-5p|rno-let-7b-5p|rno-let-7d-5p|rno-let-7a-5p|rno-let-7e-5p|rno-let-7i-5p|rno-miR-22-3p|rno-miR-200b-3p|rno-miR-30c-5p|rno-let-7f-5p
NM_001109001	10	rno-let-7c-5p|rno-let-7b-5p|rno-let-7d-5p|rno-let-7a-5p|rno-let-7e-5p|rno-miR-125b-5p|rno-let-7i-5p|rno-miR-125a-5p|rno-miR-22-3p|rno-let-7f-5p
NM_012908	8	rno-let-7c-5p|rno-let-7b-5p|rno-miR-24-3p|rno-let-7d-5p|rno-let-7a-5p|rno-let-7e-5p|rno-let-7i-5p|rno-let-7f-5p
NM_001109098	8	rno-let-7c-5p|rno-let-7b-5p|rno-let-7d-5p|rno-let-7a-5p|rno-let-7e-5p|rno-let-7i-5p|rno-miR-30c-5p|rno-let-7f-5p
NM_001109281	7	rno-let-7c-5p|rno-let-7b-5p|rno-let-7d-5p|rno-let-7a-5p|rno-let-7e-5p|rno-let-7i-5p|rno-let-7f-5p
NM_001044280	7	rno-let-7c-5p|rno-let-7b-5p|rno-let-7d-5p|rno-let-7a-5p|rno-let-7e-5p|rno-let-7i-5p|rno-let-7f-5p
NM_213627	7	rno-let-7c-5p|rno-let-7b-5p|rno-let-7d-5p|rno-let-7a-5p|rno-let-7e-5p|rno-let-7i-5p|rno-let-7f-5p
NM_012535	7	rno-let-7c-5p|rno-let-7b-5p|rno-let-7d-5p|rno-let-7a-5p|rno-let-7e-5p|rno-let-7i-5p|rno-let-7f-5p
NM_001012134	9	rno-let-7c-5p|rno-let-7b-5p|rno-let-7d-5p|rno-let-7a-5p|rno-let-7e-5p|rno-let-7i-5p|rno-miR-27b-3p|rno-miR-27a-3p|rno-let-7f-5p
NM_213563	7	rno-let-7c-5p|rno-let-7b-5p|rno-let-7d-5p|rno-let-7a-5p|rno-let-7e-5p|rno-let-7i-5p|rno-let-7f-5p

NM_001025711	7	rno-let-7c-5p|rno-let-7b-5p|rno-let-7d-5p|rno-let-7a-5p|rno-let-7e-5p|rno-let-7i-5p|rno-let-7f-5p
NM_001107959	11	rno-let-7c-5p|rno-miR-26a-5p|rno-let-7b-5p|rno-miR-23a-3p|rno-miR-23b-3p|rno-let-7d-5p|rno-let-7a-5p|rno-let-7e-5p|rno-let-7i-5p|rno-miR-99b-5p|rno-let-7f-5p
NM_001191882	7	rno-let-7c-5p|rno-let-7b-5p|rno-let-7d-5p|rno-let-7a-5p|rno-let-7e-5p|rno-let-7i-5p|rno-let-7f-5p
NM_001107596	11	rno-let-7c-5p|rno-miR-26a-5p|rno-let-7b-5p|rno-miR-23a-3p|rno-miR-23b-3p|rno-let-7d-5p|rno-let-7a-5p|rno-let-7e-5p|rno-let-7i-5p|rno-miR-320-3p|rno-let-7f-5p
NM_001108546	15	rno-let-7c-5p|rno-let-7b-5p|rno-miR-23a-3p|rno-miR-23b-3p|rno-let-7d-5p|rno-let-7a-5p|rno-let-7e-5p|rno-let-7i-5p|rno-miR-29a-3p|rno-miR-200b-3p|rno-miR-181a-5p|rno-miR-30c-5p|rno-miR-17-5p|rno-let-7f-5p|rno-miR-93-5p
NM_001142367	7	rno-let-7c-5p|rno-let-7b-5p|rno-let-7d-5p|rno-let-7a-5p|rno-let-7e-5p|rno-let-7i-5p|rno-let-7f-5p
NM_198779	12	rno-let-7c-5p|rno-let-7b-5p|rno-let-7d-5p|rno-let-7a-5p|rno-let-7e-5p|rno-miR-103-3p|rno-let-7i-5p|rno-miR-107-3p|rno-miR-29a-3p|rno-miR-181a-5p|rno-miR-152-3p|rno-let-7f-5p
NM_001107439	8	rno-let-7c-5p|rno-let-7b-5p|rno-let-7d-5p|rno-let-7a-5p|rno-let-7e-5p|rno-miR-6216|rno-let-7i-5p|rno-let-7f-5p
NM_001033693	7	rno-let-7c-5p|rno-let-7b-5p|rno-let-7d-5p|rno-let-7a-5p|rno-let-7e-5p|rno-let-7i-5p|rno-let-7f-5p
NM_001108286	9	rno-let-7c-5p|rno-let-7b-5p|rno-let-7d-5p|rno-let-7a-5p|rno-let-7e-5p|rno-let-7i-5p|rno-miR-27b-3p|rno-miR-27a-3p|rno-let-7f-5p
NM_001100749	8	rno-let-7c-5p|rno-let-7b-5p|rno-let-7d-5p|rno-let-7a-5p|rno-let-7e-5p|rno-let-7i-5p|rno-miR-152-3p|rno-let-7f-5p
NM_001108467	7	rno-let-7c-5p|rno-let-7b-5p|rno-let-7d-5p|rno-let-7a-5p|rno-let-7e-5p|rno-let-7i-5p|rno-let-7f-5p

NM_001005874	7	rno-let-7c-5p|rno-let-7b-5p|rno-let-7d-5p|rno-let-7a-5p|rno-let-7e-5p|rno-let-7i-5p|rno-let-7f-5p
NM_001285415	7	rno-let-7c-5p|rno-let-7b-5p|rno-let-7d-5p|rno-let-7a-5p|rno-let-7e-5p|rno-let-7i-5p|rno-let-7f-5p
NM_139192	11	rno-let-7c-5p|rno-let-7b-5p|rno-let-7d-5p|rno-let-7a-5p|rno-let-7e-5p|rno-let-7i-5p|rno-miR-320-3p|rno-miR-199a-3p|rno-miR-200b-3p|rno-miR-181a-5p|rno-let-7f-5p
NM_031622	9	rno-let-7c-5p|rno-miR-26a-5p|rno-let-7b-5p|rno-let-7d-5p|rno-let-7a-5p|rno-let-7e-5p|rno-miR-6216|rno-let-7i-5p|rno-let-7f-5p
NM_198789	9	rno-let-7c-5p|rno-let-7b-5p|rno-miR-23a-3p|rno-miR-23b-3p|rno-let-7d-5p|rno-let-7a-5p|rno-let-7e-5p|rno-let-7i-5p|rno-let-7f-5p
NM_001108185	8	rno-let-7c-5p|rno-let-7b-5p|rno-let-7d-5p|rno-let-7a-5p|rno-let-7e-5p|rno-let-7i-5p|rno-miR-200b-3p|rno-let-7f-5p
NM_001134571	8	rno-let-7c-5p|rno-let-7b-5p|rno-miR-24-3p|rno-let-7d-5p|rno-let-7a-5p|rno-let-7e-5p|rno-let-7i-5p|rno-let-7f-5p
NM_001271270	8	rno-let-7c-5p|rno-miR-26a-5p|rno-let-7b-5p|rno-let-7d-5p|rno-let-7a-5p|rno-let-7e-5p|rno-let-7i-5p|rno-let-7f-5p
NM_001105967	8	rno-let-7c-5p|rno-let-7b-5p|rno-let-7d-5p|rno-let-7a-5p|rno-let-7e-5p|rno-let-7i-5p|rno-miR-29a-3p|rno-let-7f-5p
NM_001109014	12	rno-let-7c-5p|rno-let-7b-5p|rno-let-7d-5p|rno-let-7a-5p|rno-let-7e-5p|rno-miR-6216|rno-miR-16-5p|rno-let-7i-5p|rno-miR-195-5p|rno-miR-29a-3p|rno-let-7f-5p|rno-miR-15b-5p
NM_001013096	13	rno-let-7c-5p|rno-let-7b-5p|rno-let-7d-5p|rno-let-7a-5p|rno-let-7e-5p|rno-miR-6216|rno-miR-103-3p|rno-let-7i-5p|rno-miR-107-3p|rno-miR-29a-3p|rno-miR-17-5p|rno-let-7f-5p|rno-miR-93-5p
NM_012492	12	rno-let-7c-5p|rno-miR-26a-5p|rno-let-7b-5p|rno-let-7d-5p|rno-let-7a-5p|rno-let-7e-5p|rno-miR-16-5p|rno-let-7i-5p|rno-miR-195-5p|rno-miR-200b-3p|rno-let-7f-5p|rno-miR-15b-5p
NM_001025720	8	rno-let-7c-5p|rno-let-7b-5p|rno-let-7d-5p|rno-let-7a-5p|rno-let-7e-5p|rno-let-7i-5p|rno-miR-378a-3p|rno-let-7f-5p

NM_001106995	12	rno-let-7c-5p|rno-let-7b-5p|rno-let-7d-5p|rno-let-7a-5p|rno-let-7e-5p|rno-miR-125b-5p|rno-let-7i-5p|rno-miR-125a-5p|rno-miR-27b-3p|rno-miR-27a-3p|rno-miR-181a-5p|rno-let-7f-5p
NM_001107451	7	rno-let-7c-5p|rno-let-7b-5p|rno-let-7d-5p|rno-let-7a-5p|rno-let-7e-5p|rno-let-7i-5p|rno-let-7f-5p
NM_001014093	7	rno-let-7c-5p|rno-let-7b-5p|rno-let-7d-5p|rno-let-7a-5p|rno-let-7e-5p|rno-let-7i-5p|rno-let-7f-5p
NM_001038495	10	rno-let-7c-5p|rno-let-7b-5p|rno-let-7d-5p|rno-let-7a-5p|rno-let-7e-5p|rno-let-7i-5p|rno-miR-3473|rno-miR-30c-5p|rno-miR-152-3p|rno-let-7f-5p
NM_001013900	8	rno-let-7c-5p|rno-let-7b-5p|rno-miR-24-3p|rno-let-7d-5p|rno-let-7a-5p|rno-let-7e-5p|rno-let-7i-5p|rno-let-7f-5p
NM_001108487	7	rno-let-7c-5p|rno-let-7b-5p|rno-let-7d-5p|rno-let-7a-5p|rno-let-7e-5p|rno-let-7i-5p|rno-let-7f-5p
NM_001031653	7	rno-let-7c-5p|rno-let-7b-5p|rno-miR-24-3p|rno-let-7a-5p|rno-let-7e-5p|rno-let-7i-5p|rno-let-7f-5p
NM_001134865	7	rno-let-7c-5p|rno-let-7b-5p|rno-let-7d-5p|rno-let-7a-5p|rno-let-7e-5p|rno-let-7i-5p|rno-let-7f-5p
NM_001106277	8	rno-let-7c-5p|rno-let-7b-5p|rno-miR-24-3p|rno-let-7d-5p|rno-let-7a-5p|rno-let-7e-5p|rno-let-7i-5p|rno-let-7f-5p
NM_001112737	6	rno-let-7c-5p|rno-let-7b-5p|rno-let-7a-5p|rno-let-7e-5p|rno-let-7i-5p|rno-let-7f-5p
NM_001109025	8	rno-let-7c-5p|rno-let-7b-5p|rno-let-7d-5p|rno-let-7a-5p|rno-let-7e-5p|rno-let-7i-5p|rno-miR-200b-3p|rno-let-7f-5p
NM_001191932	7	rno-let-7c-5p|rno-let-7b-5p|rno-let-7d-5p|rno-let-7a-5p|rno-let-7e-5p|rno-let-7i-5p|rno-let-7f-5p
NM_001008298	9	rno-let-7c-5p|rno-let-7b-5p|rno-miR-23a-3p|rno-miR-23b-3p|rno-let-7d-5p|rno-let-7a-5p|rno-let-7e-5p|rno-let-7i-5p|rno-let-7f-5p
NM_001106429	6	rno-let-7c-5p|rno-let-7b-5p|rno-let-7a-5p|rno-let-7e-5p|rno-let-7i-5p|rno-let-7f-5p

NM_133571	12	rno-let-7c-5p|rno-let-7b-5p|rno-let-7d-5p|rno-let-7a-5p|rno-let-7e-5p|rno-miR-16-5p|rno-miR-103-3p|rno-let-7i-5p|rno-miR-107-3p|rno-miR-195-5p|rno-let-7f-5p|rno-miR-15b-5p
NM_001271324	10	rno-let-7c-5p|rno-miR-26a-5p|rno-let-7b-5p|rno-let-7d-5p|rno-let-7a-5p|rno-let-7e-5p|rno-miR-103-3p|rno-let-7i-5p|rno-miR-107-3p|rno-let-7f-5p
NM_001109346	7	rno-let-7c-5p|rno-let-7b-5p|rno-let-7d-5p|rno-let-7a-5p|rno-let-7e-5p|rno-let-7i-5p|rno-let-7f-5p
NM_001106388	10	rno-let-7c-5p|rno-let-7b-5p|rno-miR-23a-3p|rno-miR-23b-3p|rno-let-7d-5p|rno-let-7a-5p|rno-let-7e-5p|rno-let-7i-5p|rno-miR-30c-5p|rno-let-7f-5p
NM_001033868	8	rno-let-7c-5p|rno-let-7b-5p|rno-let-7d-5p|rno-let-7a-5p|rno-let-7e-5p|rno-let-7i-5p|rno-miR-30c-5p|rno-let-7f-5p
NM_001191595	7	rno-let-7c-5p|rno-let-7b-5p|rno-let-7d-5p|rno-let-7a-5p|rno-let-7e-5p|rno-let-7i-5p|rno-let-7f-5p
NM_001017374	9	rno-let-7c-5p|rno-miR-26a-5p|rno-let-7b-5p|rno-let-7d-5p|rno-let-7a-5p|rno-let-7e-5p|rno-let-7i-5p|rno-miR-31a-5p|rno-let-7f-5p
NM_012685	7	rno-let-7c-5p|rno-let-7b-5p|rno-let-7d-5p|rno-let-7a-5p|rno-let-7e-5p|rno-let-7i-5p|rno-let-7f-5p
NM_001037441	7	rno-let-7c-5p|rno-let-7b-5p|rno-let-7d-5p|rno-let-7a-5p|rno-let-7e-5p|rno-let-7i-5p|rno-let-7f-5p
NM_001005889	9	rno-let-7c-5p|rno-let-7b-5p|rno-let-7d-5p|rno-let-7a-5p|rno-let-7e-5p|rno-let-7i-5p|rno-miR-181a-5p|rno-miR-31a-5p|rno-let-7f-5p
NM_001047849	7	rno-let-7c-5p|rno-let-7b-5p|rno-miR-24-3p|rno-let-7a-5p|rno-let-7e-5p|rno-let-7i-5p|rno-let-7f-5p
NM_022601	8	rno-let-7c-5p|rno-let-7b-5p|rno-miR-24-3p|rno-let-7a-5p|rno-let-7e-5p|rno-miR-6216|rno-let-7i-5p|rno-let-7f-5p
NM_001271261	10	rno-let-7c-5p|rno-let-7b-5p|rno-miR-23a-3p|rno-miR-23b-3p|rno-let-7d-5p|rno-let-7a-5p|rno-let-7e-5p|rno-let-7i-5p|rno-miR-29a-3p|rno-let-7f-5p

NM_012548	10	rno-let-7c-5p|rno-let-7b-5p|rno-let-7d-5p|rno-let-7a-5p|rno-let-7e-5p|rno-miR-125b-5p|rno-let-7i-5p|rno-miR-125a-5p|rno-miR-199a-3p|rno-let-7f-5p
NM_001107023	7	rno-let-7c-5p|rno-let-7b-5p|rno-let-7d-5p|rno-let-7a-5p|rno-let-7e-5p|rno-let-7i-5p|rno-let-7f-5p
NM_001106369	20	rno-let-7c-5p|rno-miR-26a-5p|rno-let-7b-5p|rno-let-7d-5p|rno-let-7a-5p|rno-let-7e-5p|rno-miR-16-5p|rno-miR-125b-5p|rno-miR-103-3p|rno-let-7i-5p|rno-miR-125a-5p|rno-miR-107-3p|rno-miR-27b-3p|rno-miR-195-5p|rno-miR-205|rno-miR-27a-3p|rno-miR-200b-3p|rno-miR-181a-5p|rno-let-7f-5p|rno-miR-15b-5p
NM_001014021	7	rno-let-7c-5p|rno-let-7b-5p|rno-let-7d-5p|rno-let-7a-5p|rno-let-7e-5p|rno-let-7i-5p|rno-let-7f-5p
NM_001108883	9	rno-let-7c-5p|rno-let-7b-5p|rno-miR-23a-3p|rno-miR-23b-3p|rno-let-7d-5p|rno-let-7a-5p|rno-let-7e-5p|rno-let-7i-5p|rno-let-7f-5p
NM_001033710	7	rno-let-7c-5p|rno-let-7b-5p|rno-let-7d-5p|rno-let-7a-5p|rno-let-7e-5p|rno-let-7i-5p|rno-let-7f-5p
NM_001134862	9	rno-let-7c-5p|rno-let-7b-5p|rno-let-7d-5p|rno-let-7a-5p|rno-let-7e-5p|rno-let-7i-5p|rno-miR-320-3p|rno-miR-205|rno-let-7f-5p
NM_001191884	7	rno-let-7c-5p|rno-let-7b-5p|rno-let-7d-5p|rno-let-7a-5p|rno-let-7e-5p|rno-let-7i-5p|rno-let-7f-5p
NM_012686	7	rno-let-7c-5p|rno-let-7b-5p|rno-let-7d-5p|rno-let-7a-5p|rno-let-7e-5p|rno-let-7i-5p|rno-let-7f-5p
NM_022623	11	rno-let-7c-5p|rno-let-7b-5p|rno-let-7d-5p|rno-let-7a-5p|rno-let-7e-5p|rno-let-7i-5p|rno-miR-27b-3p|rno-miR-27a-3p|rno-miR-17-5p|rno-let-7f-5p|rno-miR-93-5p
NM_001106686	10	rno-let-7c-5p|rno-let-7b-5p|rno-let-7d-5p|rno-let-7a-5p|rno-let-7e-5p|rno-miR-103-3p|rno-let-7i-5p|rno-miR-107-3p|rno-miR-152-3p|rno-let-7f-5p
NM_019265	7	rno-let-7c-5p|rno-let-7b-5p|rno-let-7d-5p|rno-let-7a-5p|rno-let-7e-5p|rno-let-7i-5p|rno-let-7f-5p

NM_024141	7	rno-let-7c-5p|rno-let-7b-5p|rno-let-7d-5p|rno-let-7a-5p|rno-let-7e-5p|rno-let-7i-5p|rno-let-7f-5p
NM_001108275	8	rno-let-7c-5p|rno-let-7b-5p|rno-let-7d-5p|rno-let-7a-5p|rno-let-7e-5p|rno-let-7i-5p|rno-miR-30c-5p|rno-let-7f-5p
NM_019272	9	rno-let-7c-5p|rno-let-7b-5p|rno-let-7d-5p|rno-let-7a-5p|rno-let-7e-5p|rno-miR-125b-5p|rno-let-7i-5p|rno-miR-125a-5p|rno-let-7f-5p
NM_001106788	7	rno-let-7c-5p|rno-let-7b-5p|rno-let-7d-5p|rno-let-7a-5p|rno-let-7e-5p|rno-let-7i-5p|rno-let-7f-5p
NM_001134520	6	rno-let-7c-5p|rno-let-7b-5p|rno-let-7a-5p|rno-let-7e-5p|rno-let-7i-5p|rno-let-7f-5p
NM_001105878	12	rno-let-7c-5p|rno-let-7b-5p|rno-let-7d-5p|rno-let-7a-5p|rno-let-7e-5p|rno-miR-16-5p|rno-let-7i-5p|rno-miR-195-5p|rno-miR-17-5p|rno-let-7f-5p|rno-miR-15b-5p|rno-miR-93-5p
NM_001100547	11	rno-let-7c-5p|rno-let-7b-5p|rno-let-7d-5p|rno-let-7a-5p|rno-let-7e-5p|rno-miR-125b-5p|rno-let-7i-5p|rno-miR-125a-5p|rno-miR-205|rno-miR-31a-5p|rno-let-7f-5p
NM_080899	8	rno-let-7c-5p|rno-let-7b-5p|rno-let-7d-5p|rno-let-7a-5p|rno-let-7e-5p|rno-miR-6216|rno-let-7i-5p|rno-let-7f-5p
NM_001144870	9	rno-let-7c-5p|rno-let-7b-5p|rno-miR-23a-3p|rno-miR-23b-3p|rno-let-7d-5p|rno-let-7a-5p|rno-let-7e-5p|rno-let-7i-5p|rno-let-7f-5p
NM_022259	9	rno-let-7c-5p|rno-let-7b-5p|rno-let-7d-5p|rno-let-7a-5p|rno-let-7e-5p|rno-let-7i-5p|rno-miR-320-3p|rno-let-7f-5p|rno-miR-151-5p
NM_001134747	8	rno-let-7c-5p|rno-let-7b-5p|rno-miR-24-3p|rno-let-7d-5p|rno-let-7a-5p|rno-let-7e-5p|rno-let-7i-5p|rno-let-7f-5p
NM_001106324	11	rno-let-7c-5p|rno-let-7b-5p|rno-miR-23a-3p|rno-miR-23b-3p|rno-miR-24-3p|rno-let-7d-5p|rno-let-7a-5p|rno-let-7e-5p|rno-let-7i-5p|rno-miR-320-3p|rno-let-7f-5p
NM_001013186	7	rno-let-7c-5p|rno-let-7b-5p|rno-let-7d-5p|rno-let-7a-5p|rno-let-7e-5p|rno-let-7i-5p|rno-let-7f-5p

NM_001107904	9	rno-let-7c-5p|rno-let-7b-5p|rno-miR-24-3p|rno-let-7d-5p|rno-let-7a-5p|rno-let-7e-5p|rno-let-7i-5p|rno-miR-200b-3p|rno-let-7f-5p
NM_001014200	10	rno-let-7c-5p|rno-let-7b-5p|rno-let-7d-5p|rno-let-7a-5p|rno-let-7e-5p|rno-miR-103-3p|rno-let-7i-5p|rno-miR-107-3p|rno-miR-29a-3p|rno-let-7f-5p
NM_001191714	11	rno-let-7c-5p|rno-let-7b-5p|rno-let-7d-5p|rno-let-7a-5p|rno-let-7e-5p|rno-let-7i-5p|rno-miR-27b-3p|rno-miR-27a-3p|rno-miR-31a-5p|rno-miR-30c-5p|rno-let-7f-5p
NM_001037973	7	rno-let-7c-5p|rno-let-7b-5p|rno-let-7d-5p|rno-let-7a-5p|rno-let-7e-5p|rno-let-7i-5p|rno-let-7f-5p
NM_001129880	8	rno-let-7c-5p|rno-let-7b-5p|rno-let-7d-5p|rno-let-7a-5p|rno-let-7e-5p|rno-miR-6216|rno-let-7i-5p|rno-let-7f-5p
NM_001106657	10	rno-let-7c-5p|rno-let-7b-5p|rno-let-7d-5p|rno-let-7a-5p|rno-let-7e-5p|rno-miR-103-3p|rno-let-7i-5p|rno-miR-107-3p|rno-miR-29a-3p|rno-let-7f-5p
NM_001017472	7	rno-let-7c-5p|rno-let-7b-5p|rno-let-7d-5p|rno-let-7a-5p|rno-let-7e-5p|rno-let-7i-5p|rno-let-7f-5p
NM_001134961	6	rno-let-7c-5p|rno-let-7b-5p|rno-let-7a-5p|rno-let-7e-5p|rno-let-7i-5p|rno-let-7f-5p
NM_001004265	7	rno-let-7c-5p|rno-let-7b-5p|rno-let-7d-5p|rno-let-7a-5p|rno-let-7e-5p|rno-let-7i-5p|rno-let-7f-5p
NM_001031648	7	rno-let-7c-5p|rno-let-7b-5p|rno-let-7d-5p|rno-let-7a-5p|rno-let-7e-5p|rno-let-7i-5p|rno-let-7f-5p
NM_001107018	16	rno-let-7c-5p|rno-let-7b-5p|rno-let-7d-5p|rno-let-7a-5p|rno-let-7e-5p|rno-miR-16-5p|rno-miR-103-3p|rno-let-7i-5p|rno-miR-107-3p|rno-miR-320-3p|rno-miR-195-5p|rno-miR-181a-5p|rno-miR-17-5p|rno-let-7f-5p|rno-miR-15b-5p|rno-miR-93-5p

NM_001130537	16	rno-let-7c-5p|rno-let-7b-5p|rno-let-7d-5p|rno-let-7a-5p|rno-let-7e-5p|rno-miR-16-5p|rno-miR-103-3p|rno-let-7i-5p|rno-miR-107-3p|rno-miR-27b-3p|rno-miR-22-3p|rno-miR-195-5p|rno-miR-27a-3p|rno-miR-30c-5p|rno-let-7f-5p|rno-miR-15b-5p
NM_022383	7	rno-let-7c-5p|rno-let-7b-5p|rno-let-7d-5p|rno-let-7a-5p|rno-let-7e-5p|rno-let-7i-5p|rno-let-7f-5p
NM_001007733	7	rno-let-7c-5p|rno-let-7b-5p|rno-let-7d-5p|rno-let-7a-5p|rno-let-7e-5p|rno-let-7i-5p|rno-let-7f-5p
NM_183056	8	rno-let-7c-5p|rno-let-7b-5p|rno-let-7d-5p|rno-let-7a-5p|rno-let-7e-5p|rno-let-7i-5p|rno-miR-31a-5p|rno-let-7f-5p
NM_001127638	9	rno-let-7c-5p|rno-let-7b-5p|rno-let-7d-5p|rno-let-7a-5p|rno-let-7e-5p|rno-let-7i-5p|rno-miR-27b-3p|rno-miR-27a-3p|rno-let-7f-5p
NM_001034854	8	rno-let-7c-5p|rno-let-7b-5p|rno-let-7d-5p|rno-let-7a-5p|rno-let-7e-5p|rno-let-7i-5p|rno-miR-30c-5p|rno-let-7f-5p
NM_001108423	7	rno-let-7c-5p|rno-let-7b-5p|rno-let-7d-5p|rno-let-7a-5p|rno-let-7e-5p|rno-let-7i-5p|rno-let-7f-5p
NM_001024355	7	rno-let-7c-5p|rno-let-7b-5p|rno-let-7d-5p|rno-let-7a-5p|rno-let-7e-5p|rno-let-7i-5p|rno-let-7f-5p
NM_001012351	9	rno-let-7c-5p|rno-let-7b-5p|rno-miR-23a-3p|rno-miR-23b-3p|rno-let-7d-5p|rno-let-7a-5p|rno-let-7e-5p|rno-let-7i-5p|rno-let-7f-5p
NM_001170595	9	rno-let-7c-5p|rno-let-7b-5p|rno-let-7d-5p|rno-let-7a-5p|rno-let-7e-5p|rno-let-7i-5p|rno-miR-17-5p|rno-let-7f-5p|rno-miR-93-5p
NM_001108575	8	rno-let-7c-5p|rno-let-7b-5p|rno-let-7d-5p|rno-let-7a-5p|rno-let-7e-5p|rno-let-7i-5p|rno-miR-378a-3p|rno-let-7f-5p
NM_001135009	11	rno-let-7c-5p|rno-let-7b-5p|rno-miR-23a-3p|rno-miR-23b-3p|rno-let-7d-5p|rno-let-7a-5p|rno-let-7e-5p|rno-let-7i-5p|rno-miR-29a-3p|rno-miR-152-3p|rno-let-7f-5p
NM_017045	8	rno-let-7c-5p|rno-miR-26a-5p|rno-let-7b-5p|rno-let-7a-5p|rno-let-7e-5p|rno-let-7i-5p|rno-miR-199a-3p|rno-let-7f-5p

NM_001130579	6	rno-let-7c-5p|rno-let-7b-5p|rno-let-7a-5p|rno-let-7e-5p|rno-let-7i-5p|rno-let-7f-5p
NM_001107566	7	rno-let-7c-5p|rno-let-7b-5p|rno-let-7d-5p|rno-let-7a-5p|rno-let-7e-5p|rno-let-7i-5p|rno-let-7f-5p
NM_130426	9	rno-let-7c-5p|rno-let-7b-5p|rno-let-7d-5p|rno-let-7a-5p|rno-let-7e-5p|rno-miR-125b-5p|rno-let-7i-5p|rno-miR-125a-5p|rno-let-7f-5p
NM_053341	7	rno-let-7c-5p|rno-let-7b-5p|rno-let-7d-5p|rno-let-7a-5p|rno-let-7e-5p|rno-let-7i-5p|rno-let-7f-5p
NM_012589	7	rno-let-7c-5p|rno-let-7b-5p|rno-let-7d-5p|rno-let-7a-5p|rno-let-7e-5p|rno-let-7i-5p|rno-let-7f-5p
NM_022238	10	rno-let-7c-5p|rno-let-7b-5p|rno-miR-24-3p|rno-let-7d-5p|rno-let-7a-5p|rno-let-7e-5p|rno-let-7i-5p|rno-miR-27b-3p|rno-miR-27a-3p|rno-let-7f-5p
NM_198782	7	rno-let-7c-5p|rno-let-7b-5p|rno-let-7d-5p|rno-let-7a-5p|rno-let-7e-5p|rno-let-7i-5p|rno-let-7f-5p
NM_017029	9	rno-let-7c-5p|rno-let-7b-5p|rno-miR-24-3p|rno-let-7d-5p|rno-let-7a-5p|rno-let-7e-5p|rno-let-7i-5p|rno-miR-30c-5p|rno-let-7f-5p
NM_012997	7	rno-let-7c-5p|rno-let-7b-5p|rno-let-7d-5p|rno-let-7a-5p|rno-let-7e-5p|rno-let-7i-5p|rno-let-7f-5p
NM_053571	8	rno-let-7c-5p|rno-let-7b-5p|rno-let-7d-5p|rno-let-7a-5p|rno-let-7e-5p|rno-let-7i-5p|rno-miR-199a-3p|rno-let-7f-5p
NM_001012224	7	rno-let-7c-5p|rno-let-7b-5p|rno-let-7d-5p|rno-let-7a-5p|rno-let-7e-5p|rno-let-7i-5p|rno-let-7f-5p
NM_001107175	13	rno-let-7c-5p|rno-miR-26a-5p|rno-let-7b-5p|rno-let-7d-5p|rno-let-7a-5p|rno-let-7e-5p|rno-let-7i-5p|rno-miR-22-3p|rno-miR-205|rno-miR-29a-3p|rno-miR-17-5p|rno-let-7f-5p|rno-miR-93-5p
NM_001134530	10	rno-let-7c-5p|rno-let-7b-5p|rno-let-7d-5p|rno-let-7a-5p|rno-let-7e-5p|rno-miR-16-5p|rno-let-7i-5p|rno-miR-195-5p|rno-let-7f-5p|rno-miR-15b-5p

NM_001108802	8	rno-let-7c-5p|rno-let-7b-5p|rno-let-7a-5p|rno-let-7e-5p|rno-miR-125b-5p|rno-let-7i-5p|rno-miR-125a-5p|rno-let-7f-5p
NM_024381	7	rno-let-7c-5p|rno-let-7b-5p|rno-let-7d-5p|rno-let-7a-5p|rno-let-7e-5p|rno-let-7i-5p|rno-let-7f-5p
NM_133615	18	rno-let-7c-5p|rno-let-7b-5p|rno-miR-23a-3p|rno-miR-23b-3p|rno-miR-24-3p|rno-let-7d-5p|rno-let-7a-5p|rno-let-7e-5p|rno-miR-16-5p|rno-miR-125b-5p|rno-let-7i-5p|rno-miR-3473|rno-miR-125a-5p|rno-miR-22-3p|rno-miR-195-5p|rno-miR-181a-5p|rno-let-7f-5p|rno-miR-15b-5p
NM_001005886	14	rno-let-7c-5p|rno-let-7b-5p|rno-miR-24-3p|rno-let-7d-5p|rno-let-7a-5p|rno-let-7e-5p|rno-miR-6216|rno-miR-125b-5p|rno-let-7i-5p|rno-miR-3473|rno-miR-125a-5p|rno-miR-181a-5p|rno-miR-30c-5p|rno-let-7f-5p
NM_001100995	7	rno-let-7c-5p|rno-let-7b-5p|rno-let-7d-5p|rno-let-7a-5p|rno-let-7e-5p|rno-let-7i-5p|rno-let-7f-5p
NM_176075	8	rno-let-7c-5p|rno-let-7b-5p|rno-let-7d-5p|rno-let-7a-5p|rno-let-7e-5p|rno-let-7i-5p|rno-miR-30c-5p|rno-let-7f-5p
NM_001271381	11	rno-let-7c-5p|rno-let-7b-5p|rno-let-7d-5p|rno-let-7a-5p|rno-let-7e-5p|rno-miR-16-5p|rno-let-7i-5p|rno-miR-195-5p|rno-miR-205|rno-let-7f-5p|rno-miR-15b-5p
NM_133565	9	rno-let-7c-5p|rno-let-7b-5p|rno-let-7d-5p|rno-let-7a-5p|rno-let-7e-5p|rno-miR-6216|rno-let-7i-5p|rno-miR-30c-5p|rno-let-7f-5p
NM_024133	7	rno-let-7c-5p|rno-let-7b-5p|rno-let-7d-5p|rno-let-7a-5p|rno-let-7e-5p|rno-let-7i-5p|rno-let-7f-5p
NM_001126290	7	rno-let-7c-5p|rno-let-7b-5p|rno-let-7d-5p|rno-let-7a-5p|rno-let-7e-5p|rno-let-7i-5p|rno-let-7f-5p
NM_001107296	9	rno-let-7c-5p|rno-miR-26a-5p|rno-let-7b-5p|rno-let-7d-5p|rno-let-7a-5p|rno-let-7e-5p|rno-let-7i-5p|rno-miR-29a-3p|rno-let-7f-5p
NM_001108954	9	rno-let-7c-5p|rno-let-7b-5p|rno-let-7d-5p|rno-let-7a-5p|rno-let-7e-5p|rno-let-7i-5p|rno-miR-29a-3p|rno-miR-200b-3p|rno-let-7f-5p

NM_175764	12	rno-let-7c-5p|rno-let-7b-5p|rno-let-7d-5p|rno-let-7a-5p|rno-let-7e-5p|rno-miR-6216|rno-let-7i-5p|rno-miR-199a-3p|rno-miR-27b-3p|rno-miR-27a-3p|rno-miR-30c-5p|rno-let-7f-5p
NM_001270545	9	rno-let-7c-5p|rno-let-7b-5p|rno-miR-24-3p|rno-let-7d-5p|rno-let-7a-5p|rno-let-7e-5p|rno-let-7i-5p|rno-miR-320-3p|rno-let-7f-5p
NM_001108164	8	rno-let-7c-5p|rno-let-7b-5p|rno-let-7d-5p|rno-let-7a-5p|rno-let-7e-5p|rno-let-7i-5p|rno-miR-205|rno-let-7f-5p
NM_001013981	6	rno-let-7c-5p|rno-let-7b-5p|rno-let-7a-5p|rno-let-7e-5p|rno-let-7i-5p|rno-let-7f-5p
NM_031031	9	rno-let-7c-5p|rno-let-7b-5p|rno-let-7d-5p|rno-let-7a-5p|rno-let-7e-5p|rno-let-7i-5p|rno-miR-22-3p|rno-miR-30c-5p|rno-let-7f-5p
NM_001014011	7	rno-let-7c-5p|rno-let-7b-5p|rno-let-7d-5p|rno-let-7a-5p|rno-let-7e-5p|rno-let-7i-5p|rno-let-7f-5p
NM_001100805	7	rno-let-7c-5p|rno-let-7b-5p|rno-let-7d-5p|rno-let-7a-5p|rno-let-7e-5p|rno-let-7i-5p|rno-let-7f-5p
NM_133553	7	rno-let-7c-5p|rno-let-7b-5p|rno-let-7d-5p|rno-let-7a-5p|rno-let-7e-5p|rno-let-7i-5p|rno-let-7f-5p
NM_001191697	9	rno-let-7c-5p|rno-let-7b-5p|rno-let-7d-5p|rno-let-7a-5p|rno-let-7e-5p|rno-let-7i-5p|rno-miR-17-5p|rno-let-7f-5p|rno-miR-93-5p
NM_001108304	10	rno-let-7c-5p|rno-let-7b-5p|rno-let-7d-5p|rno-let-7a-5p|rno-let-7e-5p|rno-miR-103-3p|rno-let-7i-5p|rno-miR-107-3p|rno-let-7f-5p|rno-miR-15b-5p
NM_001170438	8	rno-let-7c-5p|rno-let-7b-5p|rno-miR-24-3p|rno-let-7d-5p|rno-let-7a-5p|rno-let-7e-5p|rno-let-7i-5p|rno-let-7f-5p
NM_001109508	7	rno-let-7c-5p|rno-let-7b-5p|rno-let-7d-5p|rno-let-7a-5p|rno-let-7e-5p|rno-let-7i-5p|rno-let-7f-5p
NM_001098782	8	rno-let-7c-5p|rno-let-7b-5p|rno-let-7d-5p|rno-let-7a-5p|rno-let-7e-5p|rno-let-7i-5p|rno-miR-3473|rno-let-7f-5p
NM_019367	7	rno-let-7c-5p|rno-let-7b-5p|rno-let-7d-5p|rno-let-7a-5p|rno-let-7e-5p|rno-let-7i-5p|rno-let-7f-5p

NM_001106247	8	rno-let-7c-5p|rno-let-7b-5p|rno-let-7d-5p|rno-let-7a-5p|rno-let-7e-5p|rno-let-7i-5p|rno-miR-30c-5p|rno-let-7f-5p
NM_001012225	13	rno-let-7c-5p|rno-miR-26a-5p|rno-let-7b-5p|rno-let-7d-5p|rno-let-7a-5p|rno-let-7e-5p|rno-miR-16-5p|rno-let-7i-5p|rno-miR-27b-3p|rno-miR-195-5p|rno-miR-27a-3p|rno-let-7f-5p|rno-miR-15b-5p
NM_001105927	9	rno-let-7c-5p|rno-let-7b-5p|rno-let-7d-5p|rno-let-7a-5p|rno-let-7e-5p|rno-let-7i-5p|rno-miR-17-5p|rno-let-7f-5p|rno-miR-93-5p
NM_001322798	9	rno-let-7c-5p|rno-let-7b-5p|rno-let-7d-5p|rno-let-7a-5p|rno-let-7e-5p|rno-miR-125b-5p|rno-let-7i-5p|rno-miR-125a-5p|rno-let-7f-5p
NM_181376	9	rno-let-7c-5p|rno-let-7b-5p|rno-let-7d-5p|rno-let-7a-5p|rno-let-7e-5p|rno-miR-103-3p|rno-let-7i-5p|rno-miR-107-3p|rno-let-7f-5p
NM_001107429	7	rno-let-7c-5p|rno-let-7b-5p|rno-let-7d-5p|rno-let-7a-5p|rno-let-7e-5p|rno-let-7i-5p|rno-let-7f-5p
NM_001107542	9	rno-let-7c-5p|rno-let-7b-5p|rno-let-7d-5p|rno-let-7a-5p|rno-let-7e-5p|rno-let-7i-5p|rno-miR-30c-5p|rno-miR-152-3p|rno-let-7f-5p
NM_130424	8	rno-let-7c-5p|rno-let-7b-5p|rno-let-7d-5p|rno-let-7a-5p|rno-let-7e-5p|rno-let-7i-5p|rno-miR-181a-5p|rno-let-7f-5p
NM_053502	7	rno-let-7c-5p|rno-let-7b-5p|rno-let-7d-5p|rno-let-7a-5p|rno-let-7e-5p|rno-let-7i-5p|rno-let-7f-5p
NM_001305171	5	rno-let-7c-5p|rno-let-7b-5p|rno-let-7a-5p|rno-let-7e-5p|rno-miR-29a-3p
NM_001107157	7	rno-let-7c-5p|rno-let-7b-5p|rno-let-7d-5p|rno-let-7a-5p|rno-let-7e-5p|rno-let-7i-5p|rno-let-7f-5p
NM_001005381	8	rno-let-7c-5p|rno-let-7b-5p|rno-let-7d-5p|rno-let-7a-5p|rno-let-7e-5p|rno-miR-6216|rno-let-7i-5p|rno-let-7f-5p
NM_001172084	10	rno-let-7c-5p|rno-let-7b-5p|rno-let-7d-5p|rno-let-7a-5p|rno-let-7e-5p|rno-let-7i-5p|rno-miR-3473|rno-miR-17-5p|rno-let-7f-5p|rno-miR-93-5p
NM_001008305	9	rno-let-7c-5p|rno-let-7b-5p|rno-let-7d-5p|rno-let-7a-5p|rno-let-7e-5p|rno-miR-125b-5p|rno-let-7i-5p|rno-miR-125a-5p|rno-let-7f-5p

NM_017019	8	rno-let-7c-5p|rno-let-7b-5p|rno-let-7d-5p|rno-let-7a-5p|rno-let-7e-5p|rno-let-7i-5p|rno-miR-181a-5p|rno-let-7f-5p
NM_001044279	7	rno-let-7c-5p|rno-let-7b-5p|rno-let-7d-5p|rno-let-7a-5p|rno-let-7e-5p|rno-let-7i-5p|rno-let-7f-5p
NM_001107362	15	rno-let-7c-5p|rno-let-7b-5p|rno-miR-23a-3p|rno-miR-23b-3p|rno-let-7d-5p|rno-let-7a-5p|rno-let-7e-5p|rno-miR-6216|rno-let-7i-5p|rno-miR-27b-3p|rno-miR-27a-3p|rno-miR-181a-5p|rno-miR-17-5p|rno-let-7f-5p|rno-miR-93-5p
NM_001107055	10	rno-let-7c-5p|rno-let-7b-5p|rno-let-7d-5p|rno-let-7a-5p|rno-let-7e-5p|rno-let-7i-5p|rno-miR-17-5p|rno-miR-152-3p|rno-let-7f-5p|rno-miR-93-5p
NM_172021	9	rno-let-7c-5p|rno-let-7b-5p|rno-let-7d-5p|rno-let-7a-5p|rno-let-7e-5p|rno-miR-103-3p|rno-let-7i-5p|rno-miR-107-3p|rno-let-7f-5p
NM_001173336	9	rno-let-7c-5p|rno-let-7b-5p|rno-let-7d-5p|rno-let-7a-5p|rno-let-7e-5p|rno-miR-125b-5p|rno-let-7i-5p|rno-miR-125a-5p|rno-let-7f-5p
NM_001100652	10	rno-let-7c-5p|rno-let-7b-5p|rno-let-7d-5p|rno-let-7a-5p|rno-let-7e-5p|rno-let-7i-5p|rno-miR-27b-3p|rno-miR-27a-3p|rno-let-7f-5p|rno-miR-151-5p
NM_001100813	13	rno-let-7c-5p|rno-let-7b-5p|rno-miR-23a-3p|rno-miR-23b-3p|rno-let-7d-5p|rno-let-7a-5p|rno-let-7e-5p|rno-miR-16-5p|rno-let-7i-5p|rno-miR-195-5p|rno-miR-200b-3p|rno-let-7f-5p|rno-miR-15b-5p
NM_013108	7	rno-let-7c-5p|rno-let-7b-5p|rno-let-7d-5p|rno-let-7a-5p|rno-let-7e-5p|rno-let-7i-5p|rno-let-7f-5p
NM_134405	8	rno-let-7c-5p|rno-let-7b-5p|rno-let-7d-5p|rno-let-7a-5p|rno-let-7e-5p|rno-let-7i-5p|rno-miR-199a-3p|rno-let-7f-5p
NM_001107758	11	rno-let-7c-5p|rno-let-7b-5p|rno-miR-23a-3p|rno-miR-23b-3p|rno-let-7d-5p|rno-let-7a-5p|rno-miR-191a-5p|rno-let-7e-5p|rno-let-7i-5p|rno-miR-199a-3p|rno-let-7f-5p

NM_022624	12	rno-let-7c-5p|rno-miR-26a-5p|rno-let-7b-5p|rno-let-7d-5p|rno-let-7a-5p|rno-let-7e-5p|rno-let-7i-5p|rno-miR-27b-3p|rno-miR-27a-3p|rno-miR-17-5p|rno-let-7f-5p|rno-miR-93-5p
NM_001107834	13	rno-let-7c-5p|rno-let-7b-5p|rno-let-7d-5p|rno-let-7a-5p|rno-let-7e-5p|rno-miR-16-5p|rno-let-7i-5p|rno-miR-320-3p|rno-miR-195-5p|rno-miR-17-5p|rno-let-7f-5p|rno-miR-15b-5p|rno-miR-93-5p
NM_001139484	8	rno-let-7c-5p|rno-let-7b-5p|rno-let-7a-5p|rno-let-7e-5p|rno-let-7i-5p|rno-miR-31a-5p|rno-miR-30c-5p|rno-let-7f-5p
NM_001033998	7	rno-let-7c-5p|rno-let-7b-5p|rno-let-7d-5p|rno-let-7a-5p|rno-let-7e-5p|rno-let-7i-5p|rno-let-7f-5p
NM_001109448	7	rno-let-7c-5p|rno-let-7b-5p|rno-let-7d-5p|rno-let-7a-5p|rno-let-7e-5p|rno-let-7i-5p|rno-let-7f-5p
NM_001025011	7	rno-let-7c-5p|rno-let-7b-5p|rno-let-7d-5p|rno-let-7a-5p|rno-let-7e-5p|rno-let-7i-5p|rno-let-7f-5p
NM_001105789	7	rno-let-7c-5p|rno-let-7b-5p|rno-let-7d-5p|rno-let-7a-5p|rno-let-7e-5p|rno-let-7i-5p|rno-let-7f-5p
NM_001134612	10	rno-let-7c-5p|rno-let-7b-5p|rno-let-7d-5p|rno-let-7a-5p|rno-let-7e-5p|rno-miR-6216|rno-let-7i-5p|rno-miR-181a-5p|rno-miR-152-3p|rno-let-7f-5p
NM_001005895	10	rno-let-7c-5p|rno-let-7b-5p|rno-let-7d-5p|rno-let-7a-5p|rno-let-7e-5p|rno-miR-125b-5p|rno-let-7i-5p|rno-miR-125a-5p|rno-miR-30c-5p|rno-let-7f-5p
NM_199409	9	rno-let-7c-5p|rno-let-7b-5p|rno-let-7d-5p|rno-let-7a-5p|rno-let-7e-5p|rno-let-7i-5p|rno-miR-17-5p|rno-let-7f-5p|rno-miR-93-5p
NM_001111115	7	rno-let-7c-5p|rno-let-7b-5p|rno-let-7d-5p|rno-let-7a-5p|rno-let-7e-5p|rno-let-7i-5p|rno-let-7f-5p
NM_001134544	7	rno-let-7c-5p|rno-let-7b-5p|rno-let-7d-5p|rno-let-7a-5p|rno-let-7e-5p|rno-let-7i-5p|rno-let-7f-5p
NM_001017447	7	rno-let-7c-5p|rno-let-7b-5p|rno-let-7d-5p|rno-let-7a-5p|rno-let-7e-5p|rno-let-7i-5p|rno-let-7f-5p

NM_001004083	12	rno-let-7c-5p|rno-let-7b-5p|rno-miR-23a-3p|rno-miR-23b-3p|rno-let-7d-5p|rno-let-7a-5p|rno-let-7e-5p|rno-miR-16-5p|rno-let-7i-5p|rno-miR-195-5p|rno-let-7f-5p|rno-miR-15b-5p
NM_024135	11	rno-let-7c-5p|rno-let-7b-5p|rno-miR-24-3p|rno-let-7d-5p|rno-let-7a-5p|rno-let-7e-5p|rno-let-7i-5p|rno-miR-27b-3p|rno-miR-22-3p|rno-miR-27a-3p|rno-let-7f-5p
NM_198757	7	rno-let-7c-5p|rno-let-7b-5p|rno-let-7d-5p|rno-let-7a-5p|rno-let-7e-5p|rno-let-7i-5p|rno-let-7f-5p
NM_001126301	7	rno-let-7c-5p|rno-let-7b-5p|rno-let-7d-5p|rno-let-7a-5p|rno-let-7e-5p|rno-let-7i-5p|rno-let-7f-5p
NM_017259	18	rno-let-7c-5p|rno-let-7b-5p|rno-let-7d-5p|rno-let-7a-5p|rno-let-7e-5p|rno-miR-16-5p|rno-miR-103-3p|rno-let-7i-5p|rno-miR-107-3p|rno-miR-320-3p|rno-miR-27b-3p|rno-miR-195-5p|rno-miR-27a-3p|rno-miR-29a-3p|rno-miR-17-5p|rno-let-7f-5p|rno-miR-15b-5p|rno-miR-93-5p
NM_001024305	7	rno-let-7c-5p|rno-let-7b-5p|rno-let-7d-5p|rno-let-7a-5p|rno-let-7e-5p|rno-let-7i-5p|rno-let-7f-5p
NM_001270598	8	rno-let-7c-5p|rno-let-7b-5p|rno-let-7d-5p|rno-let-7a-5p|rno-let-7e-5p|rno-let-7i-5p|rno-miR-181a-5p|rno-let-7f-5p
NM_001106906	7	rno-let-7c-5p|rno-let-7b-5p|rno-let-7d-5p|rno-let-7a-5p|rno-let-7e-5p|rno-let-7i-5p|rno-let-7f-5p
NM_001106484	9	rno-let-7c-5p|rno-let-7b-5p|rno-let-7d-5p|rno-let-7a-5p|rno-let-7e-5p|rno-let-7i-5p|rno-miR-181a-5p|rno-miR-30c-5p|rno-let-7f-5p
NM_001105980	7	rno-let-7c-5p|rno-let-7b-5p|rno-let-7d-5p|rno-let-7a-5p|rno-let-7e-5p|rno-let-7i-5p|rno-let-7f-5p
NM_001106447	7	rno-let-7c-5p|rno-let-7b-5p|rno-let-7d-5p|rno-let-7a-5p|rno-let-7e-5p|rno-let-7i-5p|rno-let-7f-5p
NM_001191756	10	rno-let-7c-5p|rno-let-7b-5p|rno-let-7d-5p|rno-let-7a-5p|rno-let-7e-5p|rno-miR-103-3p|rno-let-7i-5p|rno-miR-107-3p|rno-let-7f-5p|rno-miR-151-5p

NM_001108974	9	rno-let-7c-5p|rno-let-7b-5p|rno-let-7d-5p|rno-let-7a-5p|rno-let-7e-5p|rno-miR-103-3p|rno-let-7i-5p|rno-miR-107-3p|rno-let-7f-5p
NM_001107171	18	rno-let-7c-5p|rno-miR-26a-5p|rno-let-7b-5p|rno-miR-23a-3p|rno-miR-23b-3p|rno-let-7d-5p|rno-let-7a-5p|rno-let-7e-5p|rno-miR-6216|rno-miR-16-5p|rno-let-7i-5p|rno-miR-195-5p|rno-miR-29a-3p|rno-miR-30c-5p|rno-miR-17-5p|rno-let-7f-5p|rno-miR-15b-5p|rno-miR-93-5p
NM_001107071	8	rno-let-7c-5p|rno-let-7b-5p|rno-let-7d-5p|rno-let-7a-5p|rno-let-7e-5p|rno-let-7i-5p|rno-miR-30c-5p|rno-let-7f-5p
NM_001100669	8	rno-let-7c-5p|rno-miR-26a-5p|rno-let-7b-5p|rno-let-7d-5p|rno-let-7a-5p|rno-let-7e-5p|rno-let-7i-5p|rno-let-7f-5p
NM_001100717	9	rno-let-7c-5p|rno-let-7b-5p|rno-miR-23a-3p|rno-miR-23b-3p|rno-let-7d-5p|rno-let-7a-5p|rno-let-7e-5p|rno-let-7i-5p|rno-let-7f-5p
NM_001107585	7	rno-let-7c-5p|rno-let-7b-5p|rno-let-7d-5p|rno-let-7a-5p|rno-let-7e-5p|rno-let-7i-5p|rno-let-7f-5p
NM_001107312	7	rno-let-7c-5p|rno-let-7b-5p|rno-let-7d-5p|rno-let-7a-5p|rno-let-7e-5p|rno-let-7i-5p|rno-let-7f-5p
NM_001270599	8	rno-let-7c-5p|rno-let-7b-5p|rno-let-7d-5p|rno-let-7a-5p|rno-let-7e-5p|rno-let-7i-5p|rno-miR-181a-5p|rno-let-7f-5p
NM_012775	11	rno-let-7c-5p|rno-let-7b-5p|rno-let-7d-5p|rno-let-7a-5p|rno-let-7e-5p|rno-miR-6216|rno-miR-125b-5p|rno-let-7i-5p|rno-miR-125a-5p|rno-miR-181a-5p|rno-let-7f-5p
NM_021766	7	rno-let-7c-5p|rno-let-7b-5p|rno-let-7d-5p|rno-let-7a-5p|rno-let-7e-5p|rno-let-7i-5p|rno-let-7f-5p
NM_012665	6	rno-let-7c-5p|rno-let-7b-5p|rno-let-7a-5p|rno-let-7e-5p|rno-let-7i-5p|rno-let-7f-5p
NM_001107058	18	rno-let-7c-5p|rno-let-7b-5p|rno-miR-23a-3p|rno-miR-23b-3p|rno-let-7d-5p|rno-let-7a-5p|rno-let-7e-5p|rno-miR-16-5p|rno-miR-103-3p|rno-let-7i-5p|rno-miR-107-3p|rno-miR-22-3p|rno-miR-195-5p|rno-miR-181a-5p|rno-miR-17-5p|rno-let-7f-5p|rno-miR-15b-5p|rno-miR-93-5p

NM_040669	6	rno-let-7c-5p|rno-let-7b-5p|rno-let-7a-5p|rno-let-7e-5p|rno-let-7i-5p|rno-let-7f-5p
NM_001106216	6	rno-let-7c-5p|rno-let-7b-5p|rno-let-7a-5p|rno-let-7e-5p|rno-let-7i-5p|rno-let-7f-5p
NM_001025119	7	rno-let-7c-5p|rno-let-7b-5p|rno-let-7d-5p|rno-let-7a-5p|rno-let-7e-5p|rno-let-7i-5p|rno-let-7f-5p
NM_001033680	7	rno-let-7c-5p|rno-let-7b-5p|rno-let-7d-5p|rno-let-7a-5p|rno-let-7e-5p|rno-let-7i-5p|rno-let-7f-5p
NM_001012162	10	rno-let-7c-5p|rno-let-7b-5p|rno-miR-23a-3p|rno-miR-23b-3p|rno-let-7d-5p|rno-let-7a-5p|rno-let-7e-5p|rno-let-7i-5p|rno-miR-152-3p|rno-let-7f-5p
NM_001006980	10	rno-let-7c-5p|rno-let-7b-5p|rno-let-7d-5p|rno-let-7a-5p|rno-let-7e-5p|rno-let-7i-5p|rno-miR-27b-3p|rno-miR-27a-3p|rno-miR-152-3p|rno-let-7f-5p
NM_001106098	8	rno-let-7c-5p|rno-let-7b-5p|rno-miR-24-3p|rno-let-7d-5p|rno-let-7a-5p|rno-let-7e-5p|rno-let-7i-5p|rno-let-7f-5p
NM_173126	7	rno-let-7c-5p|rno-let-7b-5p|rno-let-7d-5p|rno-let-7a-5p|rno-let-7e-5p|rno-let-7i-5p|rno-let-7f-5p
NM_053829	14	rno-let-7c-5p|rno-let-7b-5p|rno-let-7d-5p|rno-let-7a-5p|rno-let-7e-5p|rno-miR-16-5p|rno-miR-103-3p|rno-let-7i-5p|rno-miR-3473|rno-miR-107-3p|rno-miR-195-5p|rno-miR-30c-5p|rno-let-7f-5p|rno-miR-15b-5p
NM_001271179	6	rno-let-7c-5p|rno-let-7b-5p|rno-let-7a-5p|rno-let-7e-5p|rno-let-7i-5p|rno-let-7f-5p
NM_053957	7	rno-let-7c-5p|rno-let-7b-5p|rno-let-7d-5p|rno-let-7a-5p|rno-let-7e-5p|rno-let-7i-5p|rno-let-7f-5p
NM_001100601	7	rno-let-7c-5p|rno-let-7b-5p|rno-let-7a-5p|rno-let-7e-5p|rno-let-7i-5p|rno-miR-320-3p|rno-let-7f-5p
NM_001106015	7	rno-let-7c-5p|rno-let-7b-5p|rno-let-7d-5p|rno-let-7a-5p|rno-let-7e-5p|rno-let-7i-5p|rno-let-7f-5p

NM_001107162	7	rno-let-7c-5p|rno-let-7b-5p|rno-let-7d-5p|rno-let-7a-5p|rno-let-7e-5p|rno-let-7i-5p|rno-let-7f-5p
NM_019212	7	rno-let-7c-5p|rno-let-7b-5p|rno-let-7d-5p|rno-let-7a-5p|rno-let-7e-5p|rno-let-7i-5p|rno-let-7f-5p
NM_001014004	7	rno-let-7c-5p|rno-let-7b-5p|rno-let-7d-5p|rno-let-7a-5p|rno-let-7e-5p|rno-let-7i-5p|rno-let-7f-5p
NM_001025700	7	rno-let-7c-5p|rno-let-7b-5p|rno-let-7d-5p|rno-let-7a-5p|rno-let-7e-5p|rno-let-7i-5p|rno-let-7f-5p
NM_017225	9	rno-let-7c-5p|rno-let-7b-5p|rno-let-7d-5p|rno-let-7a-5p|rno-let-7e-5p|rno-miR-125b-5p|rno-let-7i-5p|rno-miR-125a-5p|rno-let-7f-5p
NM_001108170	8	rno-let-7c-5p|rno-let-7b-5p|rno-let-7d-5p|rno-let-7a-5p|rno-let-7e-5p|rno-let-7i-5p|rno-miR-320-3p|rno-let-7f-5p
NM_001109082	9	rno-let-7c-5p|rno-let-7b-5p|rno-let-7d-5p|rno-let-7a-5p|rno-let-7e-5p|rno-let-7i-5p|rno-miR-320-3p|rno-miR-29a-3p|rno-let-7f-5p
NM_001017453	7	rno-let-7c-5p|rno-let-7b-5p|rno-let-7d-5p|rno-let-7a-5p|rno-let-7e-5p|rno-let-7i-5p|rno-let-7f-5p
NM_053321	8	rno-let-7c-5p|rno-let-7b-5p|rno-let-7d-5p|rno-let-7a-5p|rno-let-7e-5p|rno-let-7i-5p|rno-miR-22-3p|rno-let-7f-5p
NM_001191604	7	rno-let-7c-5p|rno-let-7b-5p|rno-let-7d-5p|rno-let-7a-5p|rno-let-7e-5p|rno-let-7i-5p|rno-let-7f-5p
NM_001110823	9	rno-let-7c-5p|rno-let-7b-5p|rno-let-7d-5p|rno-let-7a-5p|rno-let-7e-5p|rno-let-7i-5p|rno-miR-30c-5p|rno-miR-152-3p|rno-let-7f-5p
NM_001106343	12	rno-let-7c-5p|rno-miR-26a-5p|rno-let-7b-5p|rno-let-7d-5p|rno-let-7a-5p|rno-let-7e-5p|rno-miR-16-5p|rno-let-7i-5p|rno-miR-320-3p|rno-miR-195-5p|rno-let-7f-5p|rno-miR-15b-5p
NM_139090	7	rno-let-7c-5p|rno-let-7b-5p|rno-let-7d-5p|rno-let-7a-5p|rno-let-7e-5p|rno-let-7i-5p|rno-let-7f-5p
NM_080783	7	rno-let-7c-5p|rno-let-7b-5p|rno-let-7d-5p|rno-let-7a-5p|rno-let-7e-5p|rno-let-7i-5p|rno-let-7f-5p

NM_153728	7	rno-let-7c-5p|rno-let-7b-5p|rno-let-7d-5p|rno-let-7a-5p|rno-let-7e-5p|rno-let-7i-5p|rno-let-7f-5p
NM_001271163	9	rno-let-7c-5p|rno-miR-26a-5p|rno-let-7b-5p|rno-let-7d-5p|rno-let-7a-5p|rno-let-7e-5p|rno-miR-6216|rno-let-7i-5p|rno-let-7f-5p
NM_001108309	12	rno-let-7c-5p|rno-let-7b-5p|rno-miR-23a-3p|rno-miR-23b-3p|rno-let-7d-5p|rno-let-7a-5p|rno-let-7e-5p|rno-miR-16-5p|rno-let-7i-5p|rno-miR-195-5p|rno-let-7f-5p|rno-miR-15b-5p
NM_001106948	7	rno-let-7c-5p|rno-let-7b-5p|rno-let-7d-5p|rno-let-7a-5p|rno-let-7e-5p|rno-let-7i-5p|rno-let-7f-5p
NM_001107710	10	rno-let-7c-5p|rno-let-7b-5p|rno-let-7d-5p|rno-let-7a-5p|rno-let-7e-5p|rno-miR-16-5p|rno-let-7i-5p|rno-miR-195-5p|rno-let-7f-5p|rno-miR-15b-5p
NM_031802	9	rno-let-7c-5p|rno-let-7b-5p|rno-let-7d-5p|rno-let-7a-5p|rno-let-7e-5p|rno-let-7i-5p|rno-miR-17-5p|rno-let-7f-5p|rno-miR-93-5p
NM_053488	9	rno-let-7c-5p|rno-let-7b-5p|rno-let-7d-5p|rno-let-7a-5p|rno-let-7e-5p|rno-let-7i-5p|rno-miR-320-3p|rno-miR-29a-3p|rno-let-7f-5p
NM_001106810	9	rno-let-7c-5p|rno-let-7b-5p|rno-let-7d-5p|rno-let-7a-5p|rno-let-7e-5p|rno-miR-6216|rno-let-7i-5p|rno-miR-152-3p|rno-let-7f-5p
NM_001108206	7	rno-let-7c-5p|rno-let-7b-5p|rno-let-7d-5p|rno-let-7a-5p|rno-let-7e-5p|rno-let-7i-5p|rno-let-7f-5p
NM_001105783	8	rno-let-7c-5p|rno-let-7b-5p|rno-let-7d-5p|rno-let-7a-5p|rno-let-7e-5p|rno-let-7i-5p|rno-miR-199a-3p|rno-let-7f-5p
NM_001109235	10	rno-let-7c-5p|rno-let-7b-5p|rno-let-7d-5p|rno-let-7a-5p|rno-let-7e-5p|rno-miR-125b-5p|rno-let-7i-5p|rno-miR-125a-5p|rno-miR-200b-3p|rno-let-7f-5p
NM_138901	7	rno-let-7c-5p|rno-let-7b-5p|rno-let-7d-5p|rno-let-7a-5p|rno-let-7e-5p|rno-let-7i-5p|rno-let-7f-5p
NM_001131012	10	rno-let-7c-5p|rno-let-7b-5p|rno-let-7d-5p|rno-let-7a-5p|rno-let-7e-5p|rno-let-7i-5p|rno-miR-378a-3p|rno-miR-200b-3p|rno-miR-152-3p|rno-let-7f-5p

NM_001025715	7	rno-let-7c-5p|rno-let-7b-5p|rno-let-7d-5p|rno-let-7a-5p|rno-let-7e-5p|rno-let-7i-5p|rno-let-7f-5p
NM_001305240	8	rno-let-7c-5p|rno-let-7b-5p|rno-let-7a-5p|rno-let-7e-5p|rno-let-7i-5p|rno-miR-17-5p|rno-let-7f-5p|rno-miR-93-5p
NM_001271031	6	rno-let-7c-5p|rno-let-7b-5p|rno-let-7a-5p|rno-let-7e-5p|rno-let-7i-5p|rno-let-7f-5p
NM_053924	10	rno-let-7c-5p|rno-let-7b-5p|rno-let-7d-5p|rno-let-7a-5p|rno-let-7e-5p|rno-miR-125b-5p|rno-let-7i-5p|rno-miR-125a-5p|rno-miR-320-3p|rno-let-7f-5p
NM_001106420	15	rno-let-7c-5p|rno-let-7b-5p|rno-miR-23a-3p|rno-miR-23b-3p|rno-let-7d-5p|rno-let-7a-5p|rno-let-7e-5p|rno-miR-16-5p|rno-let-7i-5p|rno-miR-195-5p|rno-miR-29a-3p|rno-miR-200b-3p|rno-miR-30c-5p|rno-let-7f-5p|rno-miR-15b-5p
NM_001127561	10	rno-let-7c-5p|rno-let-7b-5p|rno-miR-23a-3p|rno-miR-23b-3p|rno-miR-24-3p|rno-let-7d-5p|rno-let-7a-5p|rno-let-7e-5p|rno-let-7i-5p|rno-let-7f-5p
NM_001277055	7	rno-let-7c-5p|rno-let-7b-5p|rno-let-7d-5p|rno-let-7a-5p|rno-let-7e-5p|rno-let-7i-5p|rno-let-7f-5p
NM_001037651	13	rno-let-7c-5p|rno-let-7b-5p|rno-miR-23a-3p|rno-miR-23b-3p|rno-let-7d-5p|rno-let-7a-5p|rno-let-7e-5p|rno-let-7i-5p|rno-miR-27b-3p|rno-miR-27a-3p|rno-miR-181a-5p|rno-miR-30c-5p|rno-let-7f-5p
NM_001107608	10	rno-let-7c-5p|rno-let-7b-5p|rno-let-7d-5p|rno-let-7a-5p|rno-let-7e-5p|rno-miR-6216|rno-let-7i-5p|rno-miR-199a-3p|rno-miR-200b-3p|rno-let-7f-5p
NM_001107915	9	rno-let-7c-5p|rno-let-7b-5p|rno-let-7d-5p|rno-let-7a-5p|rno-let-7e-5p|rno-miR-125b-5p|rno-let-7i-5p|rno-miR-125a-5p|rno-let-7f-5p
NM_001108917	7	rno-let-7c-5p|rno-let-7b-5p|rno-let-7d-5p|rno-let-7a-5p|rno-let-7e-5p|rno-let-7i-5p|rno-let-7f-5p
NM_001109334	8	rno-let-7c-5p|rno-let-7b-5p|rno-let-7d-5p|rno-let-7a-5p|rno-let-7e-5p|rno-let-7i-5p|rno-miR-22-3p|rno-let-7f-5p

NM_001034083	14	rno-let-7c-5p|rno-miR-26a-5p|rno-let-7b-5p|rno-miR-24-3p|rno-let-7d-5p|rno-let-7a-5p|rno-let-7e-5p|rno-let-7i-5p|rno-miR-27b-3p|rno-miR-205|rno-miR-27a-3p|rno-miR-181a-5p|rno-miR-152-3p|rno-let-7f-5p
NM_001106902	12	rno-let-7c-5p|rno-let-7b-5p|rno-let-7d-5p|rno-let-7a-5p|rno-let-7e-5p|rno-miR-125b-5p|rno-let-7i-5p|rno-miR-125a-5p|rno-miR-27b-3p|rno-miR-27a-3p|rno-miR-181a-5p|rno-let-7f-5p
NM_024353	8	rno-let-7c-5p|rno-let-7b-5p|rno-let-7d-5p|rno-let-7a-5p|rno-let-7e-5p|rno-miR-6216|rno-let-7i-5p|rno-let-7f-5p
NM_001162411	12	rno-let-7c-5p|rno-let-7b-5p|rno-let-7d-5p|rno-let-7a-5p|rno-let-7e-5p|rno-let-7i-5p|rno-miR-27b-3p|rno-miR-27a-3p|rno-miR-181a-5p|rno-miR-17-5p|rno-let-7f-5p|rno-miR-93-5p
NM_001126278	7	rno-let-7c-5p|rno-let-7b-5p|rno-let-7d-5p|rno-let-7a-5p|rno-let-7e-5p|rno-let-7i-5p|rno-let-7f-5p
NM_001134562	7	rno-let-7c-5p|rno-let-7b-5p|rno-let-7d-5p|rno-let-7a-5p|rno-let-7e-5p|rno-let-7i-5p|rno-let-7f-5p
NM_001013118	7	rno-let-7c-5p|rno-let-7b-5p|rno-let-7d-5p|rno-let-7a-5p|rno-let-7e-5p|rno-let-7i-5p|rno-let-7f-5p
NM_001100475	12	rno-let-7c-5p|rno-let-7b-5p|rno-miR-24-3p|rno-let-7d-5p|rno-let-7a-5p|rno-let-7e-5p|rno-miR-6216|rno-let-7i-5p|rno-miR-27b-3p|rno-miR-27a-3p|rno-miR-29a-3p|rno-let-7f-5p
NM_001106896	9	rno-let-7c-5p|rno-let-7b-5p|rno-let-7d-5p|rno-let-7a-5p|rno-let-7e-5p|rno-let-7i-5p|rno-miR-27b-3p|rno-miR-27a-3p|rno-let-7f-5p
NM_031148	7	rno-let-7c-5p|rno-let-7b-5p|rno-let-7d-5p|rno-let-7a-5p|rno-let-7e-5p|rno-let-7i-5p|rno-let-7f-5p
NM_001107946	14	rno-let-7c-5p|rno-miR-26a-5p|rno-let-7b-5p|rno-miR-23a-3p|rno-miR-23b-3p|rno-let-7d-5p|rno-let-7a-5p|rno-let-7e-5p|rno-let-7i-5p|rno-miR-27b-3p|rno-miR-27a-3p|rno-miR-200b-3p|rno-miR-181a-5p|rno-let-7f-5p
NM_001002828	8	rno-let-7c-5p|rno-let-7b-5p|rno-let-7d-5p|rno-let-7a-5p|rno-let-7e-5p|rno-let-7i-5p|rno-miR-30c-5p|rno-let-7f-5p

NM_001100630	9	rno-let-7c-5p|rno-miR-26a-5p|rno-let-7b-5p|rno-let-7d-5p|rno-let-7a-5p|rno-let-7e-5p|rno-let-7i-5p|rno-miR-200b-3p|rno-let-7f-5p
NM_001105920	7	rno-let-7c-5p|rno-let-7b-5p|rno-let-7d-5p|rno-let-7a-5p|rno-let-7e-5p|rno-let-7i-5p|rno-let-7f-5p
NM_001001512	9	rno-let-7c-5p|rno-let-7b-5p|rno-let-7d-5p|rno-let-7a-5p|rno-let-7e-5p|rno-let-7i-5p|rno-miR-27b-3p|rno-miR-27a-3p|rno-let-7f-5p
NM_001108526	8	rno-let-7c-5p|rno-let-7b-5p|rno-let-7d-5p|rno-let-7a-5p|rno-let-7e-5p|rno-let-7i-5p|rno-miR-181a-5p|rno-let-7f-5p
NM_001134609	7	rno-let-7c-5p|rno-let-7b-5p|rno-let-7d-5p|rno-let-7a-5p|rno-let-7e-5p|rno-let-7i-5p|rno-let-7f-5p
NM_024377	7	rno-let-7c-5p|rno-let-7b-5p|rno-let-7d-5p|rno-let-7a-5p|rno-let-7e-5p|rno-let-7i-5p|rno-let-7f-5p
NM_001127544	7	rno-let-7c-5p|rno-let-7b-5p|rno-let-7d-5p|rno-let-7a-5p|rno-let-7e-5p|rno-let-7i-5p|rno-let-7f-5p
NM_022289	11	rno-let-7c-5p|rno-let-7b-5p|rno-let-7d-5p|rno-let-7a-5p|rno-let-7e-5p|rno-miR-16-5p|rno-let-7i-5p|rno-miR-195-5p|rno-miR-30c-5p|rno-let-7f-5p|rno-miR-15b-5p
NM_001106974	12	rno-let-7c-5p|rno-let-7b-5p|rno-let-7d-5p|rno-let-7a-5p|rno-let-7e-5p|rno-miR-6216|rno-let-7i-5p|rno-miR-27b-3p|rno-miR-27a-3p|rno-miR-200b-3p|rno-miR-30c-5p|rno-let-7f-5p
NM_031795	10	rno-let-7c-5p|rno-let-7b-5p|rno-miR-24-3p|rno-let-7d-5p|rno-let-7a-5p|rno-let-7e-5p|rno-let-7i-5p|rno-miR-27b-3p|rno-miR-27a-3p|rno-let-7f-5p
NM_001109269	11	rno-let-7c-5p|rno-let-7b-5p|rno-let-7d-5p|rno-let-7a-5p|rno-let-7e-5p|rno-miR-125b-5p|rno-let-7i-5p|rno-miR-125a-5p|rno-miR-181a-5p|rno-miR-30c-5p|rno-let-7f-5p
NM_133405	9	rno-let-7c-5p|rno-let-7b-5p|rno-miR-24-3p|rno-let-7d-5p|rno-let-7a-5p|rno-let-7e-5p|rno-let-7i-5p|rno-miR-30c-5p|rno-let-7f-5p
NM_001106196	9	rno-let-7c-5p|rno-let-7b-5p|rno-let-7d-5p|rno-let-7a-5p|rno-let-7e-5p|rno-let-7i-5p|rno-miR-30c-5p|rno-miR-152-3p|rno-let-7f-5p

NM_080690	6	rno-let-7c-5p|rno-let-7b-5p|rno-let-7a-5p|rno-let-7e-5p|rno-let-7i-5p|rno-let-7f-5p
NM_001015007	7	rno-let-7c-5p|rno-let-7b-5p|rno-let-7d-5p|rno-let-7a-5p|rno-let-7e-5p|rno-let-7i-5p|rno-let-7f-5p
NM_012624	7	rno-let-7c-5p|rno-let-7b-5p|rno-let-7d-5p|rno-let-7a-5p|rno-let-7e-5p|rno-let-7i-5p|rno-let-7f-5p
NM_001079895	7	rno-let-7c-5p|rno-let-7b-5p|rno-let-7d-5p|rno-let-7a-5p|rno-let-7e-5p|rno-let-7i-5p|rno-let-7f-5p
NM_001134529	7	rno-let-7c-5p|rno-let-7b-5p|rno-let-7d-5p|rno-let-7a-5p|rno-let-7e-5p|rno-let-7i-5p|rno-let-7f-5p
NM_017360	9	rno-let-7c-5p|rno-let-7b-5p|rno-let-7d-5p|rno-let-7a-5p|rno-let-7e-5p|rno-let-7i-5p|rno-miR-17-5p|rno-let-7f-5p|rno-miR-93-5p
NM_001108331	7	rno-let-7c-5p|rno-let-7b-5p|rno-let-7d-5p|rno-let-7a-5p|rno-let-7e-5p|rno-let-7i-5p|rno-let-7f-5p
NM_001009536	7	rno-let-7c-5p|rno-let-7b-5p|rno-let-7d-5p|rno-let-7a-5p|rno-let-7e-5p|rno-let-7i-5p|rno-let-7f-5p
NM_001105867	7	rno-let-7c-5p|rno-let-7b-5p|rno-let-7d-5p|rno-let-7a-5p|rno-let-7e-5p|rno-let-7i-5p|rno-let-7f-5p
NM_001109478	7	rno-let-7c-5p|rno-let-7b-5p|rno-let-7d-5p|rno-let-7a-5p|rno-let-7e-5p|rno-let-7i-5p|rno-let-7f-5p
NM_001108229	7	rno-let-7c-5p|rno-let-7b-5p|rno-let-7d-5p|rno-let-7a-5p|rno-let-7e-5p|rno-let-7i-5p|rno-let-7f-5p
NM_001034149	9	rno-let-7c-5p|rno-let-7b-5p|rno-miR-24-3p|rno-let-7d-5p|rno-let-7a-5p|rno-let-7e-5p|rno-miR-6216|rno-let-7i-5p|rno-let-7f-5p
NM_001017488	7	rno-let-7c-5p|rno-let-7b-5p|rno-let-7d-5p|rno-let-7a-5p|rno-let-7e-5p|rno-let-7i-5p|rno-let-7f-5p
NM_001105740	7	rno-let-7c-5p|rno-let-7b-5p|rno-let-7d-5p|rno-let-7a-5p|rno-let-7e-5p|rno-let-7i-5p|rno-let-7f-5p
NM_001012099	9	rno-let-7c-5p|rno-let-7b-5p|rno-let-7d-5p|rno-let-7a-5p|rno-let-7e-5p|rno-let-7i-5p|rno-miR-200b-3p|rno-miR-152-3p|rno-let-7f-5p

NM_031057	8	rno-let-7c-5p|rno-let-7b-5p|rno-let-7d-5p|rno-let-7a-5p|rno-let-7e-5p|rno-let-7i-5p|rno-miR-205|rno-let-7f-5p
NM_001009599	8	rno-let-7c-5p|rno-let-7b-5p|rno-let-7d-5p|rno-let-7a-5p|rno-let-7e-5p|rno-let-7i-5p|rno-miR-3473|rno-let-7f-5p
NM_133596	7	rno-let-7c-5p|rno-let-7b-5p|rno-let-7d-5p|rno-let-7a-5p|rno-let-7e-5p|rno-let-7i-5p|rno-let-7f-5p
NM_148892	6	rno-let-7c-5p|rno-let-7b-5p|rno-let-7a-5p|rno-let-7e-5p|rno-let-7i-5p|rno-let-7f-5p
NM_001108319	7	rno-let-7c-5p|rno-let-7b-5p|rno-let-7d-5p|rno-let-7a-5p|rno-let-7e-5p|rno-let-7i-5p|rno-let-7f-5p
NM_001191828	7	rno-let-7c-5p|rno-let-7b-5p|rno-let-7d-5p|rno-let-7a-5p|rno-let-7e-5p|rno-let-7i-5p|rno-let-7f-5p
NM_001106384	9	rno-let-7c-5p|rno-let-7b-5p|rno-let-7d-5p|rno-let-7a-5p|rno-let-7e-5p|rno-let-7i-5p|rno-miR-30c-5p|rno-miR-152-3p|rno-let-7f-5p
NM_053748	8	rno-let-7c-5p|rno-let-7b-5p|rno-let-7d-5p|rno-let-7a-5p|rno-let-7e-5p|rno-let-7i-5p|rno-miR-29a-3p|rno-let-7f-5p
NM_080766	7	rno-let-7c-5p|rno-let-7b-5p|rno-let-7d-5p|rno-let-7a-5p|rno-let-7e-5p|rno-let-7i-5p|rno-let-7f-5p
NM_001108600	6	rno-let-7c-5p|rno-let-7b-5p|rno-let-7a-5p|rno-let-7e-5p|rno-let-7i-5p|rno-let-7f-5p
NM_001013103	7	rno-let-7c-5p|rno-let-7b-5p|rno-let-7d-5p|rno-let-7a-5p|rno-let-7e-5p|rno-let-7i-5p|rno-let-7f-5p
NM_053500	9	rno-let-7c-5p|rno-let-7b-5p|rno-let-7d-5p|rno-let-7a-5p|rno-let-7e-5p|rno-let-7i-5p|rno-miR-27b-3p|rno-miR-27a-3p|rno-let-7f-5p
NM_001004090	7	rno-let-7c-5p|rno-let-7b-5p|rno-let-7d-5p|rno-let-7a-5p|rno-let-7e-5p|rno-let-7i-5p|rno-let-7f-5p
NM_001108641	7	rno-let-7c-5p|rno-let-7b-5p|rno-let-7d-5p|rno-let-7a-5p|rno-let-7e-5p|rno-let-7i-5p|rno-let-7f-5p
NM_017042	2	rno-miR-26a-5p|rno-miR-30c-5p
NM_001108887	2	rno-miR-26a-5p|rno-miR-191a-5p

NM_053963	3	rno-miR-26a-5p|rno-miR-23a-3p|rno-miR-23b-3p
NM_017166	1	rno-miR-26a-5p
NM_033499	1	rno-miR-26a-5p
NM_001191114	1	rno-miR-26a-5p
NM_001007742	2	rno-miR-26a-5p|rno-miR-30c-5p
NM_001277232	5	rno-miR-26a-5p|rno-miR-23a-3p|rno-miR-23b-3p|rno-miR-27b-3p|rno-miR-27a-3p
NM_017132	2	rno-miR-26a-5p|rno-miR-320-3p
NM_001105780	9	rno-miR-26a-5p|rno-miR-23a-3p|rno-miR-23b-3p|rno-miR-16-5p|rno-miR-27b-3p|rno-miR-195-5p|rno-miR-27a-3p|rno-miR-200b-3p|rno-miR-15b-5p
NM_001107293	4	rno-miR-26a-5p|rno-miR-125b-5p|rno-miR-125a-5p|rno-miR-30c-5p
NM_031333	1	rno-miR-26a-5p
NM_001168549	6	rno-miR-26a-5p|rno-miR-16-5p|rno-miR-126a-3p|rno-miR-195-5p|rno-miR-152-3p|rno-miR-15b-5p
NM_013160	3	rno-miR-26a-5p|rno-miR-24-3p|rno-miR-320-3p
NM_001142915	2	rno-miR-26a-5p|rno-miR-24-3p
NM_022690	1	rno-miR-26a-5p
NM_001276721	1	rno-miR-26a-5p
NM_001106517	5	rno-miR-26a-5p|rno-miR-16-5p|rno-miR-320-3p|rno-miR-195-5p|rno-miR-15b-5p
NM_001191802	4	rno-miR-26a-5p|rno-miR-16-5p|rno-miR-195-5p|rno-miR-15b-5p
NM_001127524	2	rno-miR-26a-5p|rno-miR-6216
NM_001173349	1	rno-miR-26a-5p
NM_001047087	5	rno-miR-26a-5p|rno-miR-23a-3p|rno-miR-23b-3p|rno-miR-320-3p|rno-miR-15b-5p
NM_001107674	14	rno-miR-26a-5p|rno-miR-23a-3p|rno-miR-23b-3p|rno-miR-6216|rno-miR-16-5p|rno-miR-103-3p|rno-miR-107-3p|rno-miR-27b-3p|rno-miR-195-5p|rno-miR-27a-3p|rno-miR-181a-5p|rno-miR-31a-5p|rno-miR-152-3p|rno-miR-15b-5p

NM_001191924	5	rno-miR-26a-5p|rno-miR-24-3p|rno-miR-199a-3p|rno-miR-27b-3p|rno-miR-27a-3p
NM_001013094	1	rno-miR-26a-5p
NM_001114330	2	rno-miR-26a-5p|rno-miR-181a-5p
NM_031043	1	rno-miR-26a-5p
NM_012717	1	rno-miR-26a-5p
NM_001191603	2	rno-miR-26a-5p|rno-miR-181a-5p
NM_001034117	2	rno-miR-26a-5p|rno-miR-30c-5p
NM_001030038	6	rno-miR-26a-5p|rno-miR-23a-3p|rno-miR-23b-3p|rno-miR-125b-5p|rno-miR-125a-5p|rno-miR-205
NM_001277214	5	rno-miR-26a-5p|rno-miR-23a-3p|rno-miR-23b-3p|rno-miR-27b-3p|rno-miR-27a-3p
NM_001109257	1	rno-miR-26a-5p
NM_001037349	1	rno-miR-26a-5p
NM_001100722	1	rno-miR-26a-5p
NM_145184	1	rno-miR-26a-5p
NM_057107	1	rno-miR-26a-5p
NM_133557	3	rno-miR-26a-5p|rno-miR-199a-3p|rno-miR-30c-5p
NM_001160228	10	rno-miR-26a-5p|rno-miR-23a-3p|rno-miR-23b-3p|rno-miR-125b-5p|rno-miR-3473|rno-miR-125a-5p|rno-miR-27b-3p|rno-miR-27a-3p|rno-miR-29a-3p|rno-miR-30c-5p
NM_001030041	3	rno-miR-26a-5p|rno-miR-3473|rno-miR-181a-5p
NM_001170477	3	rno-miR-26a-5p|rno-miR-23a-3p|rno-miR-23b-3p
NM_001109536	2	rno-miR-26a-5p|rno-miR-29a-3p
NM_001009533	1	rno-miR-26a-5p
NM_001271079	3	rno-miR-26a-5p|rno-miR-125b-5p|rno-miR-125a-5p
NM_001044287	2	rno-miR-26a-5p|rno-miR-181a-5p
NM_019356	1	rno-miR-26a-5p
NM_001191847	1	rno-miR-26a-5p

NM_001024293	5	rno-miR-26a-5p|rno-miR-6216|rno-miR-199a-3p|rno-miR-17-5p|rno-miR-93-5p
NM_001106425	4	rno-miR-26a-5p|rno-miR-103-3p|rno-miR-107-3p|rno-miR-320-3p
NM_001107057	6	rno-miR-26a-5p|rno-miR-191a-5p|rno-miR-6216|rno-miR-16-5p|rno-miR-195-5p|rno-miR-15b-5p
NM_001191552	5	rno-miR-26a-5p|rno-miR-23a-3p|rno-miR-23b-3p|rno-miR-24-3p|rno-miR-152-3p
NM_001024890	1	rno-miR-26a-5p
NM_001108118	2	rno-miR-26a-5p|rno-miR-152-3p
NM_001008554	1	rno-miR-26a-5p
NM_001199095	10	rno-miR-26a-5p|rno-miR-23a-3p|rno-miR-23b-3p|rno-miR-103-3p|rno-miR-107-3p|rno-miR-200b-3p|rno-miR-181a-5p|rno-miR-31a-5p|rno-miR-17-5p|rno-miR-93-5p
NM_001107738	1	rno-miR-26a-5p
NM_001191960	11	rno-miR-26a-5p|rno-miR-6216|rno-miR-16-5p|rno-miR-27b-3p|rno-miR-195-5p|rno-miR-27a-3p|rno-miR-200b-3p|rno-miR-181a-5p|rno-miR-17-5p|rno-miR-15b-5p|rno-miR-93-5p
NM_030990	2	rno-miR-26a-5p|rno-miR-29a-3p
NM_013040	1	rno-miR-26a-5p
NM_001008521	7	rno-miR-26a-5p|rno-miR-103-3p|rno-miR-107-3p|rno-miR-320-3p|rno-miR-30c-5p|rno-miR-17-5p|rno-miR-93-5p
NM_001107613	3	rno-miR-26a-5p|rno-miR-27b-3p|rno-miR-27a-3p
NM_001127299	2	rno-miR-26a-5p|rno-miR-6216
NM_001106157	2	rno-miR-26a-5p|rno-miR-320-3p
NM_001106936	2	rno-miR-26a-5p|rno-miR-181a-5p
NM_133578	5	rno-miR-26a-5p|rno-miR-23a-3p|rno-miR-23b-3p|rno-miR-199a-3p|rno-miR-181a-5p
NM_080907	1	rno-miR-26a-5p
NM_001108265	2	rno-miR-26a-5p|rno-miR-30c-5p
NM_001105843	3	rno-miR-26a-5p|rno-miR-27b-3p|rno-miR-27a-3p

NM_031673	1	rno-miR-26a-5p
NM_001305419	1	rno-miR-26a-5p
NM_001134979	1	rno-miR-26a-5p
NM_013176	1	rno-miR-26a-5p
NM_001105988	6	rno-miR-26a-5p|rno-miR-16-5p|rno-miR-195-5p|rno-miR-31a-5p|rno-miR-30c-5p|rno-miR-15b-5p
NM_001271439	3	rno-miR-26a-5p|rno-miR-6216|rno-miR-181a-5p
NM_001007264	2	rno-miR-26a-5p|rno-miR-200b-3p
NM_013156	2	rno-miR-26a-5p|rno-miR-320-3p
NM_001191052	3	rno-miR-26a-5p|rno-miR-6216|rno-miR-181a-5p
NM_001105770	1	rno-miR-26a-5p
NM_001008344	1	rno-miR-26a-5p
NM_001191837	1	rno-miR-26a-5p
NM_001109169	2	rno-miR-26a-5p|rno-miR-152-3p
NM_001108570	1	rno-miR-26a-5p
NM_001108443	2	rno-miR-26a-5p|rno-miR-200b-3p
NM_001113751	1	rno-miR-26a-5p
NM_001108747	6	rno-miR-26a-5p|rno-miR-24-3p|rno-miR-125b-5p|rno-miR-125a-5p|rno-miR-27b-3p|rno-miR-27a-3p
NM_001106467	7	rno-miR-26a-5p|rno-miR-23a-3p|rno-miR-23b-3p|rno-miR-6216|rno-miR-3473|rno-miR-320-3p|rno-miR-31a-5p
NM_031518	1	rno-miR-26a-5p
NM_001107090	2	rno-miR-26a-5p|rno-miR-200b-3p
NM_012776	1	rno-miR-26a-5p
NM_001107788	2	rno-miR-26a-5p|rno-miR-30c-5p
NM_001106270	8	rno-miR-26a-5p|rno-miR-23a-3p|rno-miR-23b-3p|rno-miR-16-5p|rno-miR-195-5p|rno-miR-17-5p|rno-miR-15b-5p|rno-miR-93-5p
NM_001126095	3	rno-miR-26a-5p|rno-miR-24-3p|rno-miR-31a-5p
NM_001135923	2	rno-miR-26a-5p|rno-miR-6216

NM_001106268	5	rno-miR-26a-5p|rno-miR-23a-3p|rno-miR-23b-3p|rno-miR-29a-3p|rno-miR-200b-3p
NM_001135583	2	rno-miR-26a-5p|rno-miR-205
NM_053779	4	rno-miR-26a-5p|rno-miR-27b-3p|rno-miR-27a-3p|rno-miR-200b-3p
NM_001305463	3	rno-miR-26a-5p|rno-miR-22-3p|rno-miR-30c-5p
NM_001013079	1	rno-miR-26a-5p
NM_019250	1	rno-miR-26a-5p
NM_001111310	1	rno-miR-26a-5p
NM_001135781	2	rno-miR-26a-5p|rno-miR-152-3p
NM_001271174	3	rno-miR-26a-5p|rno-miR-125b-5p|rno-miR-125a-5p
NM_017214	4	rno-miR-26a-5p|rno-miR-199a-3p|rno-miR-17-5p|rno-miR-93-5p
NM_001109061	5	rno-miR-26a-5p|rno-miR-16-5p|rno-miR-195-5p|rno-miR-152-3p|rno-miR-15b-5p
NM_133381	2	rno-miR-26a-5p|rno-miR-320-3p
NM_001025027	1	rno-miR-26a-5p
NM_001024800	4	rno-miR-26a-5p|rno-miR-103-3p|rno-miR-107-3p|rno-miR-30c-5p
NM_013041	1	rno-miR-26a-5p
NM_001107894	10	rno-miR-26a-5p|rno-miR-23a-3p|rno-miR-23b-3p|rno-miR-16-5p|rno-miR-320-3p|rno-miR-199a-3p|rno-miR-195-5p|rno-miR-205|rno-miR-181a-5p|rno-miR-15b-5p
NM_001134757	6	rno-miR-26a-5p|rno-miR-23a-3p|rno-miR-23b-3p|rno-miR-6216|rno-miR-103-3p|rno-miR-107-3p
NM_001100574	1	rno-miR-26a-5p
NM_001008557	1	rno-miR-26a-5p
NM_001025120	2	rno-miR-26a-5p|rno-miR-205
NM_001108834	2	rno-miR-26a-5p|rno-miR-152-3p
NM_012505	4	rno-miR-26a-5p|rno-miR-31a-5p|rno-miR-17-5p|rno-miR-93-5p
NM_001172127	3	rno-miR-26a-5p|rno-miR-6216|rno-miR-29a-3p
NM_001184973	3	rno-miR-26a-5p|rno-miR-24-3p|rno-miR-200b-3p
NM_133289	4	rno-miR-26a-5p|rno-miR-23a-3p|rno-miR-23b-3p|rno-miR-30c-5p

NM_001191780	1	rno-miR-26a-5p
NM_001126267	3	rno-miR-26a-5p|rno-miR-320-3p|rno-miR-22-3p
NM_001107335	2	rno-miR-26a-5p|rno-miR-6216
NM_001108050	3	rno-miR-26a-5p|rno-miR-125b-5p|rno-miR-125a-5p
NM_001100524	4	rno-miR-26a-5p|rno-miR-23a-3p|rno-miR-23b-3p|rno-miR-6216
NM_001258011	1	rno-miR-26a-5p
NM_031353	1	rno-miR-26a-5p
NM_001011996	1	rno-miR-26a-5p
NM_001143881	1	rno-miR-26a-5p
NM_080477	2	rno-miR-26a-5p|rno-miR-152-3p
NM_022851	3	rno-miR-26a-5p|rno-miR-27b-3p|rno-miR-27a-3p
NM_001108042	1	rno-miR-26a-5p
NM_001025039	3	rno-miR-26a-5p|rno-miR-23a-3p|rno-miR-23b-3p
NM_031135	3	rno-miR-26a-5p|rno-miR-200b-3p|rno-miR-30c-5p
NM_001025129	1	rno-miR-26a-5p
NM_001271198	4	rno-miR-26a-5p|rno-miR-16-5p|rno-miR-195-5p|rno-miR-15b-5p
NM_001106110	5	rno-miR-26a-5p|rno-miR-23a-3p|rno-miR-23b-3p|rno-miR-27b-3p|rno-miR-27a-3p
NM_001025688	1	rno-miR-26a-5p
NM_001106043	3	rno-miR-26a-5p|rno-miR-17-5p|rno-miR-93-5p
NM_001108122	5	rno-miR-26a-5p|rno-miR-6216|rno-miR-181a-5p|rno-miR-17-5p|rno-miR-93-5p
NM_001107556	1	rno-miR-26a-5p
NM_133396	1	rno-miR-26a-5p
NM_080771	2	rno-miR-26a-5p|rno-miR-152-3p
NM_001009258	1	rno-miR-26a-5p
NM_001031655	2	rno-miR-26a-5p|rno-miR-205
NM_001106941	1	rno-miR-26a-5p
NM_001191688	5	rno-miR-26a-5p|rno-miR-16-5p|rno-miR-320-3p|rno-miR-195-5p|rno-miR-15b-5p

NM_012981	6	rno-miR-26a-5p|rno-miR-16-5p|rno-miR-195-5p|rno-miR-378a-3p|rno-miR-152-3p|rno-miR-15b-5p
NM_177928	1	rno-miR-26a-5p
NM_001109482	6	rno-miR-26a-5p|rno-miR-23a-3p|rno-miR-23b-3p|rno-miR-320-3p|rno-miR-27b-3p|rno-miR-27a-3p
NM_138845	14	rno-miR-26a-5p|rno-miR-23a-3p|rno-miR-23b-3p|rno-miR-6216|rno-miR-16-5p|rno-miR-103-3p|rno-miR-107-3p|rno-miR-27b-3p|rno-miR-22-3p|rno-miR-195-5p|rno-miR-205|rno-miR-27a-3p|rno-miR-30c-5p|rno-miR-15b-5p
NM_001108015	3	rno-miR-26a-5p|rno-miR-22-3p|rno-miR-29a-3p
NM_001106704	3	rno-miR-26a-5p|rno-miR-17-5p|rno-miR-93-5p
NM_001277157	12	rno-miR-26a-5p|rno-miR-16-5p|rno-miR-320-3p|rno-miR-27b-3p|rno-miR-195-5p|rno-miR-378a-3p|rno-miR-205|rno-miR-27a-3p|rno-miR-181a-5p|rno-miR-17-5p|rno-miR-15b-5p|rno-miR-93-5p
NM_053739	2	rno-miR-26a-5p|rno-miR-30c-5p
NM_031603	2	rno-miR-26a-5p|rno-miR-31a-5p
NM_001173370	1	rno-miR-26a-5p
NM_001173427	3	rno-miR-26a-5p|rno-miR-125b-5p|rno-miR-125a-5p
NM_001106690	2	rno-miR-26a-5p|rno-miR-6216
NM_032080	1	rno-miR-26a-5p
NM_001107768	11	rno-miR-26a-5p|rno-miR-23a-3p|rno-miR-23b-3p|rno-miR-16-5p|rno-miR-320-3p|rno-miR-27b-3p|rno-miR-195-5p|rno-miR-27a-3p|rno-miR-200b-3p|rno-miR-31a-5p|rno-miR-15b-5p
NM_013103	2	rno-miR-26a-5p|rno-miR-24-3p
NM_173307	1	rno-miR-26a-5p
NM_001008374	3	rno-miR-26a-5p|rno-miR-27b-3p|rno-miR-27a-3p
NM_001134464	2	rno-miR-26a-5p|rno-miR-29a-3p
NM_001025016	1	rno-miR-26a-5p
NM_017017	1	rno-miR-26a-5p
NM_012791	1	rno-miR-26a-5p

NM_022856	6	rno-miR-26a-5p|rno-miR-23a-3p|rno-miR-23b-3p|rno-miR-200b-3p|rno-miR-181a-5p|rno-miR-30c-5p
NM_057128	4	rno-miR-26a-5p|rno-miR-378a-3p|rno-miR-30c-5p|rno-miR-152-3p
NM_053389	1	rno-miR-26a-5p
NM_001024779	3	rno-miR-26a-5p|rno-miR-23a-3p|rno-miR-23b-3p
NM_134378	3	rno-miR-26a-5p|rno-miR-378a-3p|rno-miR-200b-3p
NM_001309462	3	rno-miR-26a-5p|rno-miR-29a-3p|rno-miR-30c-5p
NM_001108036	13	rno-miR-26a-5p|rno-miR-23a-3p|rno-miR-23b-3p|rno-miR-16-5p|rno-miR-125b-5p|rno-miR-125a-5p|rno-miR-320-3p|rno-miR-199a-3p|rno-miR-27b-3p|rno-miR-195-5p|rno-miR-27a-3p|rno-miR-152-3p|rno-miR-15b-5p
NM_001109276	1	rno-miR-26a-5p
NM_001013099	1	rno-miR-26a-5p
NM_001034107	1	rno-miR-26a-5p
NM_001107971	5	rno-miR-26a-5p|rno-miR-27b-3p|rno-miR-27a-3p|rno-miR-17-5p|rno-miR-93-5p
NM_001033964	2	rno-miR-26a-5p|rno-miR-152-3p
NM_001107682	1	rno-miR-26a-5p
NM_001037770	2	rno-miR-26a-5p|rno-miR-205
NM_001107259	9	rno-miR-26a-5p|rno-miR-23a-3p|rno-miR-23b-3p|rno-miR-16-5p|rno-miR-320-3p|rno-miR-27b-3p|rno-miR-195-5p|rno-miR-27a-3p|rno-miR-15b-5p
NM_001014008	1	rno-miR-26a-5p
NM_001135893	3	rno-miR-26a-5p|rno-miR-6216|rno-miR-30c-5p
NM_001109228	1	rno-miR-26a-5p
NM_001164157	5	rno-miR-26a-5p|rno-miR-23a-3p|rno-miR-23b-3p|rno-miR-6216|rno-miR-30c-5p
NM_198785	3	rno-miR-26a-5p|rno-miR-205|rno-miR-30c-5p
NM_001106726	2	rno-miR-26a-5p|rno-miR-199a-3p

NM_001107114	7	rno-miR-26a-5p|rno-miR-16-5p|rno-miR-320-3p|rno-miR-27b-3p|rno-miR-195-5p|rno-miR-27a-3p|rno-miR-15b-5p
NM_001003401	3	rno-miR-26a-5p|rno-miR-205|rno-miR-151-5p
NM_053483	3	rno-miR-26a-5p|rno-miR-17-5p|rno-miR-93-5p
NM_153737	2	rno-miR-26a-5p|rno-miR-22-3p
NM_001105969	1	rno-miR-26a-5p
NM_001108656	2	rno-miR-26a-5p|rno-miR-30c-5p
NM_001107138	7	rno-miR-26a-5p|rno-miR-23a-3p|rno-miR-23b-3p|rno-miR-125b-5p|rno-miR-103-3p|rno-miR-125a-5p|rno-miR-107-3p
NM_001037295	1	rno-miR-26a-5p
NM_019276	5	rno-miR-26a-5p|rno-miR-27b-3p|rno-miR-27a-3p|rno-miR-200b-3p|rno-miR-30c-5p
NM_001305179	1	rno-miR-26a-5p
NM_001034198	4	rno-miR-26a-5p|rno-miR-27b-3p|rno-miR-22-3p|rno-miR-27a-3p
NM_001308149	2	rno-miR-26a-5p|rno-miR-24-3p
NM_001271296	3	rno-miR-26a-5p|rno-miR-320-3p|rno-miR-152-3p
NM_001108044	5	rno-miR-26a-5p|rno-miR-23a-3p|rno-miR-23b-3p|rno-miR-27b-3p|rno-miR-27a-3p
NM_001007693	1	rno-miR-26a-5p
NM_001109089	1	rno-miR-26a-5p
NM_001024316	1	rno-miR-26a-5p
NM_001108530	6	rno-miR-26a-5p|rno-miR-23a-3p|rno-miR-23b-3p|rno-miR-191a-5p|rno-miR-200b-3p|rno-miR-152-3p
NM_001106144	2	rno-miR-26a-5p|rno-miR-6216
NM_001130999	2	rno-miR-26a-5p|rno-miR-181a-5p
NM_022631	1	rno-miR-26a-5p
NM_001014073	6	rno-miR-26a-5p|rno-miR-103-3p|rno-miR-107-3p|rno-miR-27b-3p|rno-miR-27a-3p|rno-miR-181a-5p
NM_001025424	3	rno-miR-26a-5p|rno-miR-17-5p|rno-miR-93-5p
NM_001037652	1	rno-miR-26a-5p

NM_013117	2	rno-miR-26a-5p|rno-miR-29a-3p
NM_001109884	1	rno-miR-26a-5p
NM_053616	1	rno-miR-26a-5p
NM_001013912	1	rno-miR-26a-5p
NM_138856	2	rno-miR-26a-5p|rno-miR-181a-5p
NM_001024327	2	rno-miR-26a-5p|rno-miR-181a-5p
NM_001106404	1	rno-miR-26a-5p
NM_001109625	4	rno-miR-26a-5p|rno-miR-27b-3p|rno-miR-27a-3p|rno-miR-30c-5p
NM_001271327	1	rno-miR-26a-5p
NM_001034004	2	rno-miR-26a-5p|rno-miR-24-3p
NM_001012062	3	rno-miR-26a-5p|rno-miR-24-3p|rno-miR-181a-5p
NM_001107651	2	rno-miR-26a-5p|rno-miR-200b-3p
NM_033485	2	rno-miR-26a-5p|rno-miR-30c-5p
NM_001029900	1	rno-miR-26a-5p
NM_017309	5	rno-miR-26a-5p|rno-miR-181a-5p|rno-miR-30c-5p|rno-miR-17-5p|rno-miR-93-5p
NM_001009622	3	rno-miR-26a-5p|rno-miR-17-5p|rno-miR-93-5p
NM_001135714	1	rno-miR-26a-5p
NM_001308148	3	rno-miR-26a-5p|rno-miR-24-3p|rno-miR-200b-3p
NM_001127490	4	rno-miR-26a-5p|rno-miR-29a-3p|rno-miR-30c-5p|rno-miR-152-3p
NM_053812	4	rno-miR-26a-5p|rno-miR-125b-5p|rno-miR-125a-5p|rno-miR-29a-3p
NM_030995	2	rno-miR-26a-5p|rno-miR-181a-5p
NM_001008362	6	rno-miR-26a-5p|rno-miR-23a-3p|rno-miR-23b-3p|rno-miR-103-3p|rno-miR-107-3p|rno-miR-181a-5p
NM_001134715	1	rno-miR-26a-5p
NM_001108701	1	rno-miR-26a-5p
NM_153297	7	rno-miR-26a-5p|rno-miR-16-5p|rno-miR-103-3p|rno-miR-107-3p|rno-miR-320-3p|rno-miR-195-5p|rno-miR-15b-5p
NM_001106537	1	rno-miR-26a-5p
NM_001106358	3	rno-miR-26a-5p|rno-miR-27b-3p|rno-miR-27a-3p

NM_019362	5	rno-miR-26a-5p|rno-miR-3473|rno-miR-27b-3p|rno-miR-27a-3p|rno-miR-30c-5p
NM_001025407	1	rno-miR-26a-5p
NM_001108500	4	rno-miR-26a-5p|rno-miR-23a-3p|rno-miR-23b-3p|rno-miR-31a-5p
NM_001003708	1	rno-miR-26a-5p
NM_001126083	2	rno-miR-26a-5p|rno-miR-6216
NM_001109313	1	rno-miR-26a-5p
NM_001270947	7	rno-miR-26a-5p|rno-miR-16-5p|rno-miR-320-3p|rno-miR-195-5p|rno-miR-17-5p|rno-miR-15b-5p|rno-miR-93-5p
NM_001100780	3	rno-miR-26a-5p|rno-miR-27b-3p|rno-miR-27a-3p
NM_001134993	1	rno-miR-26a-5p
NM_053416	5	rno-miR-26a-5p|rno-miR-3473|rno-miR-27b-3p|rno-miR-205|rno-miR-27a-3p
NM_001013125	1	rno-miR-26a-5p
NM_001106605	2	rno-miR-26a-5p|rno-miR-200b-3p
NM_053927	2	rno-miR-26a-5p|rno-miR-30c-5p
NM_053622	6	rno-miR-26a-5p|rno-miR-16-5p|rno-miR-27b-3p|rno-miR-195-5p|rno-miR-27a-3p|rno-miR-15b-5p
NM_001108581	3	rno-miR-26a-5p|rno-miR-181a-5p|rno-miR-30c-5p
NM_133317	2	rno-miR-26a-5p|rno-miR-200b-3p
NM_001030054	4	rno-miR-26a-5p|rno-miR-27b-3p|rno-miR-27a-3p|rno-miR-181a-5p
NM_001108081	1	rno-miR-26a-5p
NM_001109307	7	rno-miR-26a-5p|rno-miR-24-3p|rno-miR-16-5p|rno-miR-27b-3p|rno-miR-195-5p|rno-miR-27a-3p|rno-miR-15b-5p
NM_001108329	1	rno-miR-26a-5p
NM_017206	4	rno-miR-26a-5p|rno-miR-6216|rno-miR-31a-5p|rno-miR-30c-5p
NM_053287	2	rno-miR-26a-5p|rno-miR-181a-5p
NM_001033891	1	rno-miR-26a-5p
NM_001106091	3	rno-miR-26a-5p|rno-miR-27b-3p|rno-miR-27a-3p

NM_001134756	6	rno-miR-26a-5p|rno-miR-23a-3p|rno-miR-23b-3p|rno-miR-6216|rno-miR-103-3p|rno-miR-107-3p
NM_001191627	1	rno-miR-26a-5p
NM_001106950	13	rno-miR-26a-5p|rno-miR-24-3p|rno-miR-16-5p|rno-miR-103-3p|rno-miR-107-3p|rno-miR-27b-3p|rno-miR-195-5p|rno-miR-27a-3p|rno-miR-200b-3p|rno-miR-30c-5p|rno-miR-17-5p|rno-miR-15b-5p|rno-miR-93-5p
NM_001113335	1	rno-miR-26a-5p
NM_001173437	4	rno-miR-26a-5p|rno-miR-16-5p|rno-miR-195-5p|rno-miR-15b-5p
NM_001106021	2	rno-miR-26a-5p|rno-miR-378a-3p
NM_001039606	1	rno-miR-26a-5p
NM_021587	2	rno-miR-26a-5p|rno-miR-152-3p
NM_053392	4	rno-miR-26a-5p|rno-miR-27b-3p|rno-miR-27a-3p|rno-miR-200b-3p
NM_001030024	8	rno-miR-26a-5p|rno-miR-16-5p|rno-miR-195-5p|rno-miR-205|rno-miR-200b-3p|rno-miR-17-5p|rno-miR-15b-5p|rno-miR-93-5p
NM_001002823	10	rno-miR-26a-5p|rno-miR-23a-3p|rno-miR-23b-3p|rno-miR-103-3p|rno-miR-107-3p|rno-miR-200b-3p|rno-miR-181a-5p|rno-miR-31a-5p|rno-miR-17-5p|rno-miR-93-5p
NM_001031813	3	rno-miR-26a-5p|rno-miR-23a-3p|rno-miR-23b-3p
NM_001106785	7	rno-miR-26a-5p|rno-miR-23a-3p|rno-miR-23b-3p|rno-miR-125b-5p|rno-miR-125a-5p|rno-miR-205|rno-miR-30c-5p
NM_184051	3	rno-miR-26a-5p|rno-miR-181a-5p|rno-miR-152-3p
NM_053489	1	rno-miR-26a-5p
NM_134365	1	rno-miR-26a-5p
NM_001109345	3	rno-miR-26a-5p|rno-miR-6216|rno-miR-152-3p
NM_022602	1	rno-miR-26a-5p
NM_001037546	3	rno-miR-26a-5p|rno-miR-378a-3p|rno-miR-31a-5p
NM_173119	1	rno-miR-26a-5p

NM_001044293	9	rno-miR-26a-5p|rno-miR-23a-3p|rno-miR-23b-3p|rno-miR-6216|rno-miR-320-3p|rno-miR-27b-3p|rno-miR-27a-3p|rno-miR-200b-3p|rno-miR-30c-5p
NM_001037773	7	rno-miR-26a-5p|rno-miR-16-5p|rno-miR-125b-5p|rno-miR-125a-5p|rno-miR-195-5p|rno-miR-30c-5p|rno-miR-15b-5p
NM_001012202	1	rno-miR-26a-5p
NM_001109435	1	rno-miR-26a-5p
NM_001108604	1	rno-miR-26a-5p
NM_001108791	2	rno-miR-26a-5p|rno-miR-152-3p
NM_173317	1	rno-miR-26a-5p
NM_001108371	1	rno-miR-26a-5p
NM_001107875	3	rno-miR-26a-5p|rno-miR-22-3p|rno-miR-200b-3p
NM_001134727	1	rno-miR-26a-5p
NM_001008286	1	rno-miR-26a-5p
NM_001305280	5	rno-miR-26a-5p|rno-miR-16-5p|rno-miR-195-5p|rno-miR-30c-5p|rno-miR-15b-5p
NM_001009697	1	rno-miR-26a-5p
NM_001011985	7	rno-miR-26a-5p|rno-miR-125b-5p|rno-miR-125a-5p|rno-miR-27b-3p|rno-miR-27a-3p|rno-miR-17-5p|rno-miR-93-5p
NM_001108977	1	rno-miR-26a-5p
NM_021688	1	rno-miR-26a-5p
NM_012991	4	rno-miR-26a-5p|rno-miR-16-5p|rno-miR-195-5p|rno-miR-15b-5p
NM_019147	1	rno-miR-26a-5p
NM_053865	1	rno-miR-26a-5p
NM_001134546	2	rno-miR-26a-5p|rno-miR-152-3p
NM_001108390	5	rno-miR-26a-5p|rno-miR-23a-3p|rno-miR-23b-3p|rno-miR-199a-3p|rno-miR-200b-3p
NM_001108341	4	rno-miR-26a-5p|rno-miR-320-3p|rno-miR-17-5p|rno-miR-93-5p
NM_001134553	2	rno-miR-26a-5p|rno-miR-30c-5p
NM_001135858	4	rno-miR-26a-5p|rno-miR-6216|rno-miR-199a-3p|rno-miR-29a-3p

NM_001107557	4	rno-miR-26a-5p|rno-miR-27b-3p|rno-miR-27a-3p|rno-miR-30c-5p
NM_001106416	3	rno-miR-26a-5p|rno-miR-200b-3p|rno-miR-30c-5p
NM_001014274	5	rno-miR-26a-5p|rno-miR-6216|rno-miR-320-3p|rno-miR-27b-3p|rno-miR-27a-3p
NM_053936	5	rno-miR-26a-5p|rno-miR-23a-3p|rno-miR-23b-3p|rno-miR-205|rno-miR-200b-3p
NM_001080149	1	rno-miR-26a-5p
NM_175869	2	rno-miR-26a-5p|rno-miR-24-3p
NM_001108092	5	rno-miR-26a-5p|rno-miR-27b-3p|rno-miR-27a-3p|rno-miR-181a-5p|rno-miR-30c-5p
NM_019149	4	rno-miR-26a-5p|rno-miR-24-3p|rno-miR-200b-3p|rno-miR-30c-5p
NM_031722	2	rno-miR-26a-5p|rno-miR-30c-5p
NM_001270802	3	rno-miR-26a-5p|rno-miR-17-5p|rno-miR-93-5p
NM_053794	10	rno-miR-26a-5p|rno-miR-23a-3p|rno-miR-23b-3p|rno-miR-103-3p|rno-miR-107-3p|rno-miR-200b-3p|rno-miR-181a-5p|rno-miR-31a-5p|rno-miR-17-5p|rno-miR-93-5p
NM_001107903	3	rno-miR-26a-5p|rno-miR-27b-3p|rno-miR-27a-3p
NM_001305047	1	rno-miR-26a-5p
NM_133307	3	rno-miR-26a-5p|rno-miR-6216|rno-miR-181a-5p
NM_001107211	1	rno-miR-26a-5p
NM_001014070	1	rno-miR-26a-5p
NM_001145469	4	rno-miR-26a-5p|rno-miR-23a-3p|rno-miR-23b-3p|rno-miR-181a-5p
NM_001107378	4	rno-miR-26a-5p|rno-miR-27b-3p|rno-miR-27a-3p|rno-miR-30c-5p
NM_001100860	3	rno-miR-26a-5p|rno-miR-27b-3p|rno-miR-27a-3p
NM_001024998	2	rno-miR-26a-5p|rno-miR-199a-3p
NM_080906	5	rno-miR-26a-5p|rno-miR-199a-3p|rno-miR-22-3p|rno-miR-181a-5p|rno-miR-30c-5p
NM_001106656	3	rno-miR-26a-5p|rno-miR-17-5p|rno-miR-93-5p

NM_001107712	11	rno-miR-26a-5p|rno-miR-23a-3p|rno-miR-23b-3p|rno-miR-6216|rno-miR-16-5p|rno-miR-103-3p|rno-miR-107-3p|rno-miR-195-5p|rno-miR-181a-5p|rno-miR-152-3p|rno-miR-15b-5p
NM_053965	1	rno-miR-26a-5p
NM_133411	1	rno-miR-26a-5p
NM_012979	6	rno-miR-26a-5p|rno-miR-103-3p|rno-miR-107-3p|rno-miR-27b-3p|rno-miR-27a-3p|rno-miR-181a-5p
NM_053885	8	rno-miR-26a-5p|rno-miR-24-3p|rno-miR-16-5p|rno-miR-103-3p|rno-miR-107-3p|rno-miR-195-5p|rno-miR-29a-3p|rno-miR-15b-5p
NM_001013854	1	rno-miR-26a-5p
NM_001107149	3	rno-miR-26a-5p|rno-miR-23a-3p|rno-miR-23b-3p
NM_001134781	11	rno-miR-26a-5p|rno-miR-23a-3p|rno-miR-23b-3p|rno-miR-16-5p|rno-miR-103-3p|rno-miR-107-3p|rno-miR-195-5p|rno-miR-200b-3p|rno-miR-17-5p|rno-miR-15b-5p|rno-miR-93-5p
NM_001103363	1	rno-miR-26a-5p
NM_001170541	5	rno-miR-26a-5p|rno-miR-125b-5p|rno-miR-125a-5p|rno-miR-378a-3p|rno-miR-29a-3p
NM_021771	1	rno-miR-26a-5p
NM_024390	3	rno-miR-26a-5p|rno-miR-320-3p|rno-miR-181a-5p
NM_031793	1	rno-miR-26a-5p
NM_001033931	2	rno-miR-26a-5p|rno-miR-320-3p
NM_001271295	3	rno-miR-26a-5p|rno-miR-320-3p|rno-miR-152-3p
NM_012653	1	rno-miR-26a-5p
NM_001034088	3	rno-miR-26a-5p|rno-miR-3473|rno-miR-181a-5p
NM_001107641	7	rno-miR-26a-5p|rno-miR-125b-5p|rno-miR-125a-5p|rno-miR-27b-3p|rno-miR-27a-3p|rno-miR-181a-5p|rno-miR-31a-5p
NM_001302217	7	rno-miR-26a-5p|rno-miR-199a-3p|rno-miR-200b-3p|rno-miR-30c-5p|rno-miR-17-5p|rno-miR-152-3p|rno-miR-93-5p
NM_001106753	2	rno-miR-26a-5p|rno-miR-31a-5p
NM_138871	1	rno-miR-26a-5p

NM_001033703	3	rno-miR-26a-5p|rno-miR-27b-3p|rno-miR-27a-3p
NM_001037210	2	rno-miR-26a-5p|rno-miR-181a-5p
NM_001003959	2	rno-miR-26a-5p|rno-miR-29a-3p
NM_001014090	1	rno-miR-26a-5p
NM_181631	4	rno-miR-26a-5p|rno-miR-6216|rno-miR-17-5p|rno-miR-93-5p
NM_001107203	1	rno-miR-26a-5p
NM_001106914	1	rno-miR-26a-5p
NM_012517	5	rno-miR-26a-5p|rno-miR-24-3p|rno-miR-103-3p|rno-miR-107-3p|rno-miR-200b-3p
NM_001106821	1	rno-miR-26a-5p
NM_001011939	4	rno-miR-26a-5p|rno-miR-24-3p|rno-miR-22-3p|rno-miR-17-5p
NM_020306	2	rno-miR-26a-5p|rno-miR-152-3p
NM_053522	4	rno-miR-26a-5p|rno-miR-125b-5p|rno-miR-125a-5p|rno-miR-205
NM_001107780	1	rno-miR-26a-5p
NM_017011	1	rno-miR-26a-5p
NM_001107465	7	rno-miR-26a-5p|rno-miR-103-3p|rno-miR-107-3p|rno-miR-200b-3p|rno-miR-181a-5p|rno-miR-30c-5p|rno-miR-152-3p
NM_001134871	2	rno-miR-26a-5p|rno-miR-30c-5p
NM_001106109	1	rno-miR-26a-5p
NM_001135018	1	rno-miR-26a-5p
NM_001271371	1	rno-miR-26a-5p
NM_001108394	6	rno-miR-26a-5p|rno-miR-6216|rno-miR-27b-3p|rno-miR-27a-3p|rno-miR-200b-3p|rno-miR-30c-5p
NM_024159	1	rno-miR-26a-5p
NM_001100515	1	rno-miR-26a-5p
NM_001191652	3	rno-miR-26a-5p|rno-miR-27b-3p|rno-miR-27a-3p
NM_001109239	1	rno-miR-26a-5p
NM_012715	2	rno-miR-26a-5p|rno-miR-24-3p
NM_001107269	2	rno-miR-26a-5p|rno-miR-24-3p

NM_001014216	6	rno-miR-26a-5p|rno-miR-99b-5p|rno-miR-27b-3p|rno-miR-27a-3p|rno-miR-17-5p|rno-miR-93-5p
NM_001080150	2	rno-miR-26a-5p|rno-miR-6216
NM_001025279	1	rno-miR-26a-5p
NM_001107922	1	rno-miR-26a-5p
NM_001014054	8	rno-miR-26a-5p|rno-miR-23a-3p|rno-miR-23b-3p|rno-miR-125b-5p|rno-miR-125a-5p|rno-miR-181a-5p|rno-miR-31a-5p|rno-miR-152-3p
NM_001037789	4	rno-miR-26a-5p|rno-miR-16-5p|rno-miR-195-5p|rno-miR-15b-5p
NM_001037354	3	rno-miR-26a-5p|rno-miR-17-5p|rno-miR-93-5p
NM_144745	4	rno-miR-26a-5p|rno-miR-23a-3p|rno-miR-23b-3p|rno-miR-181a-5p
NM_001107374	2	rno-miR-26a-5p|rno-miR-152-3p
NM_001107381	2	rno-miR-26a-5p|rno-miR-181a-5p
NM_001107552	3	rno-miR-26a-5p|rno-miR-22-3p|rno-miR-152-3p
NM_001191645	4	rno-miR-26a-5p|rno-miR-27b-3p|rno-miR-27a-3p|rno-miR-200b-3p
NM_182820	2	rno-miR-26a-5p|rno-miR-31a-5p
NM_001107424	5	rno-miR-26a-5p|rno-miR-24-3p|rno-miR-378a-3p|rno-miR-205|rno-miR-30c-5p
NM_001108128	1	rno-miR-26a-5p
NM_019275	2	rno-miR-26a-5p|rno-miR-205
NM_001160155	6	rno-miR-26a-5p|rno-miR-16-5p|rno-miR-27b-3p|rno-miR-195-5p|rno-miR-27a-3p|rno-miR-15b-5p
NM_145783	1	rno-miR-26a-5p
NM_001107075	3	rno-miR-26a-5p|rno-miR-195-5p|rno-miR-152-3p
NM_053713	6	rno-miR-26a-5p|rno-miR-103-3p|rno-miR-107-3p|rno-miR-29a-3p|rno-miR-200b-3p|rno-miR-152-3p
NM_031152	5	rno-miR-26a-5p|rno-miR-6216|rno-miR-3473|rno-miR-181a-5p|rno-miR-30c-5p
NM_001039339	1	rno-miR-26a-5p
NM_001107799	1	rno-miR-26a-5p

NM_001009542	4	rno-miR-26a-5p|rno-miR-103-3p|rno-miR-107-3p|rno-miR-30c-5p
NM_001131014	1	rno-miR-26a-5p
NM_001108741	2	rno-miR-26a-5p|rno-miR-24-3p
NM_001025123	3	rno-miR-26a-5p|rno-miR-200b-3p|rno-miR-181a-5p
NM_001006955	5	rno-miR-26a-5p|rno-miR-27b-3p|rno-miR-22-3p|rno-miR-27a-3p|rno-miR-30c-5p
NM_001106576	1	rno-miR-26a-5p
NM_001034948	1	rno-miR-26a-5p
NM_198791	1	rno-miR-26a-5p
NM_001159655	6	rno-miR-26a-5p|rno-miR-125b-5p|rno-miR-125a-5p|rno-miR-199a-3p|rno-miR-27b-3p|rno-miR-27a-3p
NM_001136162	1	rno-miR-26a-5p
NM_001006994	5	rno-miR-26a-5p|rno-miR-23a-3p|rno-miR-23b-3p|rno-miR-200b-3p|rno-miR-152-3p
NM_001033965	2	rno-miR-26a-5p|rno-miR-152-3p
NM_139040	3	rno-miR-26a-5p|rno-miR-17-5p|rno-miR-93-5p
NM_001009702	1	rno-miR-26a-5p
NM_001106836	7	rno-miR-26a-5p|rno-miR-6216|rno-miR-16-5p|rno-miR-27b-3p|rno-miR-195-5p|rno-miR-27a-3p|rno-miR-15b-5p
NM_001271541	5	rno-miR-26a-5p|rno-miR-23a-3p|rno-miR-23b-3p|rno-miR-17-5p|rno-miR-93-5p
NM_001108061	1	rno-miR-26a-5p
NM_030831	1	rno-miR-26a-5p
NM_012922	3	rno-let-7b-5p|rno-let-7i-5p|rno-let-7f-5p
NM_001106528	1	rno-let-7b-5p
NM_001107592	4	rno-let-7b-5p|rno-miR-125b-5p|rno-miR-125a-5p|rno-miR-29a-3p
NM_001107272	3	rno-miR-23a-3p|rno-miR-23b-3p|rno-miR-30c-5p
NM_021693	3	rno-miR-23a-3p|rno-miR-23b-3p|rno-miR-152-3p
NM_001276714	2	rno-miR-23a-3p|rno-miR-23b-3p

NM_001105825	6	rno-miR-23a-3p|rno-miR-23b-3p|rno-miR-16-5p|rno-miR-195-5p|rno-miR-29a-3p|rno-miR-15b-5p
NM_001025710	3	rno-miR-23a-3p|rno-miR-23b-3p|rno-miR-181a-5p
NM_001039516	4	rno-miR-23a-3p|rno-miR-23b-3p|rno-miR-27b-3p|rno-miR-27a-3p
NM_001100542	2	rno-miR-23a-3p|rno-miR-23b-3p
NM_001037184	4	rno-miR-23a-3p|rno-miR-23b-3p|rno-miR-27b-3p|rno-miR-27a-3p
NM_001271149	9	rno-miR-23a-3p|rno-miR-23b-3p|rno-miR-6216|rno-miR-16-5p|rno-miR-195-5p|rno-miR-30c-5p|rno-miR-17-5p|rno-miR-15b-5p|rno-miR-93-5p
NM_001103351	2	rno-miR-23a-3p|rno-miR-23b-3p
NM_001126275	5	rno-miR-23a-3p|rno-miR-23b-3p|rno-miR-27b-3p|rno-miR-205|rno-miR-27a-3p
NM_013022	11	rno-miR-23a-3p|rno-miR-23b-3p|rno-miR-6216|rno-miR-16-5p|rno-miR-3473|rno-miR-195-5p|rno-miR-205|rno-miR-200b-3p|rno-miR-17-5p|rno-miR-15b-5p|rno-miR-93-5p
NM_001108230	3	rno-miR-23a-3p|rno-miR-23b-3p|rno-miR-320-3p
NM_001110165	2	rno-miR-23a-3p|rno-miR-23b-3p
NM_001047093	3	rno-miR-23a-3p|rno-miR-23b-3p|rno-miR-181a-5p
NM_022219	5	rno-miR-23a-3p|rno-miR-23b-3p|rno-miR-24-3p|rno-miR-6216|rno-miR-30c-5p
NM_001108219	5	rno-miR-23a-3p|rno-miR-23b-3p|rno-miR-199a-3p|rno-miR-200b-3p|rno-miR-181a-5p
NM_001008888	2	rno-miR-23a-3p|rno-miR-23b-3p
NM_001107377	9	rno-miR-23a-3p|rno-miR-23b-3p|rno-miR-6216|rno-miR-16-5p|rno-miR-195-5p|rno-miR-200b-3p|rno-miR-30c-5p|rno-miR-152-3p|rno-miR-15b-5p
NM_080886	2	rno-miR-23a-3p|rno-miR-23b-3p
NM_019376	3	rno-miR-23a-3p|rno-miR-23b-3p|rno-miR-200b-3p
NM_001107588	4	rno-miR-23a-3p|rno-miR-23b-3p|rno-miR-6216|rno-miR-152-3p
NM_001108189	2	rno-miR-23a-3p|rno-miR-23b-3p

NM_001106321	2	rno-miR-23a-3p|rno-miR-23b-3p
NM_001011923	2	rno-miR-23a-3p|rno-miR-23b-3p
NM_001108125	3	rno-miR-23a-3p|rno-miR-23b-3p|rno-miR-199a-3p
NM_001191719	3	rno-miR-23a-3p|rno-miR-23b-3p|rno-miR-6216
NM_198744	3	rno-miR-23a-3p|rno-miR-23b-3p|rno-miR-3473
NM_001108313	2	rno-miR-23a-3p|rno-miR-23b-3p
NM_173149	2	rno-miR-23a-3p|rno-miR-23b-3p
NM_001037980	4	rno-miR-23a-3p|rno-miR-23b-3p|rno-miR-27b-3p|rno-miR-27a-3p
NM_130399	2	rno-miR-23a-3p|rno-miR-23b-3p
NM_001024354	2	rno-miR-23a-3p|rno-miR-23b-3p
NM_022226	2	rno-miR-23a-3p|rno-miR-23b-3p
NM_053821	2	rno-miR-23a-3p|rno-miR-23b-3p
NM_001017493	2	rno-miR-23a-3p|rno-miR-23b-3p
NM_001169129	4	rno-miR-23a-3p|rno-miR-23b-3p|rno-miR-6216|rno-miR-200b-3p
NM_001017509	2	rno-miR-23a-3p|rno-miR-23b-3p
NM_017222	2	rno-miR-23a-3p|rno-miR-23b-3p
NM_001163153	2	rno-miR-23a-3p|rno-miR-23b-3p
NM_001191917	4	rno-miR-23a-3p|rno-miR-23b-3p|rno-miR-24-3p|rno-miR-152-3p
NM_001108097	5	rno-miR-23a-3p|rno-miR-23b-3p|rno-miR-6216|rno-miR-17-5p|rno-miR-93-5p
NM_001106088	6	rno-miR-23a-3p|rno-miR-23b-3p|rno-miR-16-5p|rno-miR-195-5p|rno-miR-29a-3p|rno-miR-15b-5p
NM_001107928	3	rno-miR-23a-3p|rno-miR-23b-3p|rno-miR-24-3p
NM_001108633	6	rno-miR-23a-3p|rno-miR-23b-3p|rno-miR-24-3p|rno-miR-16-5p|rno-miR-195-5p|rno-miR-15b-5p
NM_031824	4	rno-miR-23a-3p|rno-miR-23b-3p|rno-miR-27b-3p|rno-miR-27a-3p
NM_001108062	2	rno-miR-23a-3p|rno-miR-23b-3p
NM_022939	4	rno-miR-23a-3p|rno-miR-23b-3p|rno-miR-6216|rno-miR-31a-5p
NM_001302211	8	rno-miR-23a-3p|rno-miR-23b-3p|rno-miR-16-5p|rno-miR-27b-3p|rno-miR-195-5p|rno-miR-27a-3p|rno-miR-200b-3p|rno-miR-15b-5p

NM_013111	5	rno-miR-23a-3p|rno-miR-23b-3p|rno-miR-125b-5p|rno-miR-125a-5p|rno-miR-181a-5p
NM_001177682	3	rno-miR-23a-3p|rno-miR-23b-3p|rno-miR-24-3p
NM_001277278	3	rno-miR-23a-3p|rno-miR-23b-3p|rno-miR-205
NM_139194	4	rno-miR-23a-3p|rno-miR-23b-3p|rno-miR-27b-3p|rno-miR-27a-3p
NM_001105984	5	rno-miR-23a-3p|rno-miR-23b-3p|rno-miR-16-5p|rno-miR-195-5p|rno-miR-15b-5p
NM_001109094	3	rno-miR-23a-3p|rno-miR-23b-3p|rno-miR-30c-5p
NM_001107923	7	rno-miR-23a-3p|rno-miR-23b-3p|rno-miR-16-5p|rno-miR-199a-3p|rno-miR-195-5p|rno-miR-152-3p|rno-miR-15b-5p
NM_001109652	4	rno-miR-23a-3p|rno-miR-23b-3p|rno-miR-6216|rno-miR-30c-5p
NM_199106	2	rno-miR-23a-3p|rno-miR-23b-3p
NM_001012060	3	rno-miR-23a-3p|rno-miR-23b-3p|rno-miR-30c-5p
NM_001108654	4	rno-miR-23a-3p|rno-miR-23b-3p|rno-miR-125b-5p|rno-miR-125a-5p
NM_001109677	3	rno-miR-23a-3p|rno-miR-23b-3p|rno-miR-181a-5p
NM_001013434	5	rno-miR-23a-3p|rno-miR-23b-3p|rno-miR-6216|rno-miR-181a-5p|rno-miR-30c-5p
NM_031614	5	rno-miR-23a-3p|rno-miR-23b-3p|rno-miR-125b-5p|rno-miR-125a-5p|rno-miR-205
NM_001077651	8	rno-miR-23a-3p|rno-miR-23b-3p|rno-miR-6216|rno-miR-125b-5p|rno-miR-125a-5p|rno-miR-181a-5p|rno-miR-30c-5p|rno-miR-152-3p
NM_212488	3	rno-miR-23a-3p|rno-miR-23b-3p|rno-miR-30c-5p
NM_134417	6	rno-miR-23a-3p|rno-miR-23b-3p|rno-miR-125b-5p|rno-miR-125a-5p|rno-miR-181a-5p|rno-miR-30c-5p
NM_001107755	2	rno-miR-23a-3p|rno-miR-23b-3p
NM_173322	4	rno-miR-23a-3p|rno-miR-23b-3p|rno-miR-6216|rno-miR-199a-3p
NM_001108652	3	rno-miR-23a-3p|rno-miR-23b-3p|rno-miR-151-5p
NM_001106630	2	rno-miR-23a-3p|rno-miR-23b-3p
NM_053646	2	rno-miR-23a-3p|rno-miR-23b-3p

NM_001005765	7	rno-miR-23a-3p|rno-miR-23b-3p|rno-miR-24-3p|rno-miR-6216|rno-miR-125b-5p|rno-miR-125a-5p|rno-miR-320-3p
NM_001024968	4	rno-miR-23a-3p|rno-miR-23b-3p|rno-miR-125b-5p|rno-miR-125a-5p
NM_001107533	5	rno-miR-23a-3p|rno-miR-23b-3p|rno-miR-199a-3p|rno-miR-27b-3p|rno-miR-27a-3p
NM_001106740	4	rno-miR-23a-3p|rno-miR-23b-3p|rno-miR-320-3p|rno-miR-199a-3p
NM_053682	2	rno-miR-23a-3p|rno-miR-23b-3p
NM_001271240	2	rno-miR-23a-3p|rno-miR-23b-3p
NM_001108984	3	rno-miR-23a-3p|rno-miR-23b-3p|rno-miR-30c-5p
NM_031808	6	rno-miR-23a-3p|rno-miR-23b-3p|rno-miR-16-5p|rno-miR-195-5p|rno-miR-200b-3p|rno-miR-15b-5p
NM_001007680	6	rno-miR-23a-3p|rno-miR-23b-3p|rno-miR-125b-5p|rno-miR-125a-5p|rno-miR-27b-3p|rno-miR-27a-3p
NM_001168642	2	rno-miR-23a-3p|rno-miR-23b-3p
NM_001170560	4	rno-miR-23a-3p|rno-miR-23b-3p|rno-miR-103-3p|rno-miR-107-3p
NM_001014248	8	rno-miR-23a-3p|rno-miR-23b-3p|rno-miR-16-5p|rno-miR-22-3p|rno-miR-195-5p|rno-miR-17-5p|rno-miR-15b-5p|rno-miR-93-5p
NM_001025701	6	rno-miR-23a-3p|rno-miR-23b-3p|rno-miR-378a-3p|rno-miR-181a-5p|rno-miR-17-5p|rno-miR-93-5p
NM_053583	3	rno-miR-23a-3p|rno-miR-23b-3p|rno-miR-15b-5p
NM_001013862	3	rno-miR-23a-3p|rno-miR-23b-3p|rno-miR-24-3p
NM_013031	8	rno-miR-23a-3p|rno-miR-23b-3p|rno-miR-16-5p|rno-miR-195-5p|rno-miR-181a-5p|rno-miR-17-5p|rno-miR-15b-5p|rno-miR-93-5p
NM_001134539	5	rno-miR-23a-3p|rno-miR-23b-3p|rno-miR-6216|rno-miR-17-5p|rno-miR-93-5p
NM_001108991	3	rno-miR-23a-3p|rno-miR-23b-3p|rno-miR-6216
NM_001191661	2	rno-miR-23a-3p|rno-miR-23b-3p
NM_134340	3	rno-miR-23a-3p|rno-miR-23b-3p|rno-miR-205
NM_001191574	4	rno-miR-23a-3p|rno-miR-23b-3p|rno-miR-181a-5p|rno-miR-30c-5p

NM_001107943	8	rno-miR-23a-3p|rno-miR-23b-3p|rno-miR-125b-5p|rno-miR-125a-5p|rno-miR-27b-3p|rno-miR-27a-3p|rno-miR-17-5p|rno-miR-93-5p
NM_001135804	8	rno-miR-23a-3p|rno-miR-23b-3p|rno-miR-125b-5p|rno-miR-125a-5p|rno-miR-27b-3p|rno-miR-27a-3p|rno-miR-17-5p|rno-miR-93-5p
NM_001013974	3	rno-miR-23a-3p|rno-miR-23b-3p|rno-miR-22-3p
NM_001193568	6	rno-miR-23a-3p|rno-miR-23b-3p|rno-miR-16-5p|rno-miR-195-5p|rno-miR-29a-3p|rno-miR-15b-5p
NM_001108719	2	rno-miR-23a-3p|rno-miR-23b-3p
NM_001107092	2	rno-miR-23a-3p|rno-miR-23b-3p
NM_001025624	2	rno-miR-23a-3p|rno-miR-23b-3p
NM_139253	2	rno-miR-23a-3p|rno-miR-23b-3p
NM_198778	2	rno-miR-23a-3p|rno-miR-23b-3p
NM_001135158	2	rno-miR-23a-3p|rno-miR-23b-3p
NM_031754	3	rno-miR-23a-3p|rno-miR-23b-3p|rno-miR-3473
NM_001107810	2	rno-miR-23a-3p|rno-miR-23b-3p
NM_001277210	2	rno-miR-23a-3p|rno-miR-23b-3p
NM_001170471	2	rno-miR-23a-3p|rno-miR-23b-3p
NM_001108082	11	rno-miR-23a-3p|rno-miR-23b-3p|rno-miR-24-3p|rno-miR-16-5p|rno-miR-103-3p|rno-miR-107-3p|rno-miR-199a-3p|rno-miR-195-5p|rno-miR-200b-3p|rno-miR-181a-5p|rno-miR-15b-5p
NM_001024906	3	rno-miR-23a-3p|rno-miR-23b-3p|rno-miR-200b-3p
NM_017187	3	rno-miR-23a-3p|rno-miR-23b-3p|rno-miR-181a-5p
NM_001024295	3	rno-miR-23a-3p|rno-miR-23b-3p|rno-miR-199a-3p
NM_001105718	2	rno-miR-23a-3p|rno-miR-23b-3p
NM_001106997	3	rno-miR-23a-3p|rno-miR-23b-3p|rno-miR-30c-5p
NM_001109358	3	rno-miR-23a-3p|rno-miR-23b-3p|rno-miR-152-3p
NM_001037772	2	rno-miR-23a-3p|rno-miR-23b-3p
NM_001105758	5	rno-miR-23a-3p|rno-miR-23b-3p|rno-miR-27b-3p|rno-miR-27a-3p|rno-miR-152-3p
NM_173328	2	rno-miR-23a-3p|rno-miR-23b-3p

NM_001109092	8	rno-miR-23a-3p|rno-miR-23b-3p|rno-miR-6216|rno-miR-16-5p|rno-miR-103-3p|rno-miR-107-3p|rno-miR-195-5p|rno-miR-15b-5p
NM_001024299	3	rno-miR-23a-3p|rno-miR-23b-3p|rno-miR-181a-5p
NM_001106924	4	rno-miR-23a-3p|rno-miR-23b-3p|rno-miR-103-3p|rno-miR-107-3p
NM_001105713	7	rno-miR-23a-3p|rno-miR-23b-3p|rno-miR-16-5p|rno-miR-126a-3p|rno-miR-195-5p|rno-miR-200b-3p|rno-miR-15b-5p
NM_001107918	4	rno-miR-23a-3p|rno-miR-23b-3p|rno-miR-200b-3p|rno-miR-30c-5p
NM_031351	4	rno-miR-23a-3p|rno-miR-23b-3p|rno-miR-378a-3p|rno-miR-29a-3p
NM_001106086	3	rno-miR-23a-3p|rno-miR-23b-3p|rno-miR-320-3p
NM_001106468	3	rno-miR-23a-3p|rno-miR-23b-3p|rno-miR-16-5p
NM_001127564	2	rno-miR-23a-3p|rno-miR-23b-3p
NM_001107401	5	rno-miR-23a-3p|rno-miR-23b-3p|rno-miR-17-5p|rno-miR-152-3p|rno-miR-93-5p
NM_131914	3	rno-miR-23a-3p|rno-miR-23b-3p|rno-miR-29a-3p
NM_053896	2	rno-miR-23a-3p|rno-miR-23b-3p
NM_001005552	2	rno-miR-23a-3p|rno-miR-23b-3p
NM_053475	3	rno-miR-23a-3p|rno-miR-23b-3p|rno-miR-151-5p
NM_001106414	2	rno-miR-23a-3p|rno-miR-23b-3p
NM_001107480	3	rno-miR-23a-3p|rno-miR-23b-3p|rno-miR-29a-3p
NM_001106389	3	rno-miR-23a-3p|rno-miR-23b-3p|rno-miR-320-3p
NM_001271152	4	rno-miR-23a-3p|rno-miR-23b-3p|rno-miR-27b-3p|rno-miR-27a-3p
NM_001270418	2	rno-miR-23a-3p|rno-miR-23b-3p
NM_057213	2	rno-miR-23a-3p|rno-miR-23b-3p
NM_053487	3	rno-miR-23a-3p|rno-miR-23b-3p|rno-miR-181a-5p
NM_031789	6	rno-miR-23a-3p|rno-miR-23b-3p|rno-miR-27b-3p|rno-miR-27a-3p|rno-miR-17-5p|rno-miR-93-5p
NM_031080	7	rno-miR-23a-3p|rno-miR-23b-3p|rno-miR-6216|rno-miR-125b-5p|rno-miR-125a-5p|rno-miR-205|rno-miR-30c-5p
NM_001106570	5	rno-miR-23a-3p|rno-miR-23b-3p|rno-miR-16-5p|rno-miR-195-5p|rno-miR-15b-5p

NM_001108753	3	rno-miR-23a-3p|rno-miR-23b-3p|rno-miR-6216
NM_001109278	4	rno-miR-23a-3p|rno-miR-23b-3p|rno-miR-6216|rno-miR-378a-3p
NM_001008302	2	rno-miR-23a-3p|rno-miR-23b-3p
NM_001108956	4	rno-miR-23a-3p|rno-miR-23b-3p|rno-miR-22-3p|rno-miR-152-3p
NM_017318	2	rno-miR-23a-3p|rno-miR-23b-3p
NM_001105805	2	rno-miR-23a-3p|rno-miR-23b-3p
NM_001277334	2	rno-miR-23a-3p|rno-miR-23b-3p
NM_001008879	4	rno-miR-23a-3p|rno-miR-23b-3p|rno-miR-6216|rno-miR-200b-3p
NM_032106	6	rno-miR-23a-3p|rno-miR-23b-3p|rno-miR-6216|rno-miR-200b-3p|rno-miR-181a-5p|rno-miR-152-3p
NM_001107061	8	rno-miR-23a-3p|rno-miR-23b-3p|rno-miR-16-5p|rno-miR-320-3p|rno-miR-195-5p|rno-miR-29a-3p|rno-miR-152-3p|rno-miR-15b-5p
NM_001106207	2	rno-miR-23a-3p|rno-miR-23b-3p
NM_001173339	3	rno-miR-23a-3p|rno-miR-23b-3p|rno-miR-152-3p
NM_001190475	8	rno-miR-23a-3p|rno-miR-23b-3p|rno-miR-16-5p|rno-miR-27b-3p|rno-miR-195-5p|rno-miR-27a-3p|rno-miR-200b-3p|rno-miR-15b-5p
NM_001100533	4	rno-miR-23a-3p|rno-miR-23b-3p|rno-miR-199a-3p|rno-miR-205
NM_138896	3	rno-miR-23a-3p|rno-miR-23b-3p|rno-miR-205
NM_001011947	7	rno-miR-23a-3p|rno-miR-23b-3p|rno-miR-103-3p|rno-miR-107-3p|rno-miR-378a-3p|rno-miR-30c-5p|rno-miR-152-3p
NM_001108007	6	rno-miR-23a-3p|rno-miR-23b-3p|rno-miR-24-3p|rno-miR-16-5p|rno-miR-195-5p|rno-miR-15b-5p
NM_001170559	4	rno-miR-23a-3p|rno-miR-23b-3p|rno-miR-103-3p|rno-miR-107-3p
NM_001100509	2	rno-miR-23a-3p|rno-miR-23b-3p
NM_147210	5	rno-miR-23a-3p|rno-miR-23b-3p|rno-miR-6216|rno-miR-27b-3p|rno-miR-27a-3p
NM_022282	4	rno-miR-23a-3p|rno-miR-23b-3p|rno-miR-205|rno-miR-181a-5p
NM_133582	4	rno-miR-23a-3p|rno-miR-23b-3p|rno-miR-320-3p|rno-miR-152-3p
NM_001271040	2	rno-miR-23a-3p|rno-miR-23b-3p
NM_012857	2	rno-miR-23a-3p|rno-miR-23b-3p

NM_001108536	2	rno-miR-23a-3p|rno-miR-23b-3p
NM_019291	2	rno-miR-23a-3p|rno-miR-23b-3p
NM_001080207	6	rno-miR-23a-3p|rno-miR-23b-3p|rno-miR-6216|rno-miR-320-3p|rno-miR-27b-3p|rno-miR-27a-3p
NM_001008891	6	rno-miR-23a-3p|rno-miR-23b-3p|rno-miR-16-5p|rno-miR-195-5p|rno-miR-152-3p|rno-miR-15b-5p
NM_001107094	2	rno-miR-23a-3p|rno-miR-23b-3p
NM_001106106	2	rno-miR-23a-3p|rno-miR-23b-3p
NM_001191927	3	rno-miR-23a-3p|rno-miR-23b-3p|rno-miR-24-3p
NM_001010920	3	rno-miR-23a-3p|rno-miR-23b-3p|rno-miR-320-3p
NM_001108639	5	rno-miR-23a-3p|rno-miR-23b-3p|rno-miR-205|rno-miR-17-5p|rno-miR-93-5p
NM_001108359	12	rno-miR-23a-3p|rno-miR-23b-3p|rno-miR-6216|rno-miR-16-5p|rno-miR-320-3p|rno-miR-27b-3p|rno-miR-195-5p|rno-miR-27a-3p|rno-miR-29a-3p|rno-miR-200b-3p|rno-miR-152-3p|rno-miR-15b-5p
NM_012567	3	rno-miR-23a-3p|rno-miR-23b-3p|rno-miR-30c-5p
NM_001012131	2	rno-miR-23a-3p|rno-miR-23b-3p
NM_153311	2	rno-miR-23a-3p|rno-miR-23b-3p
NM_001191865	2	rno-miR-23a-3p|rno-miR-23b-3p
NM_001106123	2	rno-miR-23a-3p|rno-miR-23b-3p
NM_030862	3	rno-miR-23a-3p|rno-miR-23b-3p|rno-miR-24-3p
NM_001309459	2	rno-miR-23a-3p|rno-miR-23b-3p
NM_001302209	8	rno-miR-23a-3p|rno-miR-23b-3p|rno-miR-16-5p|rno-miR-27b-3p|rno-miR-195-5p|rno-miR-27a-3p|rno-miR-200b-3p|rno-miR-15b-5p
NM_001033882	2	rno-miR-23a-3p|rno-miR-23b-3p
NM_001012021	2	rno-miR-23a-3p|rno-miR-23b-3p
NM_001107726	2	rno-miR-23a-3p|rno-miR-23b-3p
NM_001191735	2	rno-miR-23a-3p|rno-miR-23b-3p
NM_001134632	3	rno-miR-23a-3p|rno-miR-23b-3p|rno-miR-30c-5p
NM_001302889	4	rno-miR-23a-3p|rno-miR-23b-3p|rno-miR-27b-3p|rno-miR-27a-3p

NM_001135039	2	rno-miR-23a-3p|rno-miR-23b-3p
NM_001303144	2	rno-miR-23a-3p|rno-miR-23b-3p
NM_001107230	3	rno-miR-23a-3p|rno-miR-23b-3p|rno-miR-6216
NM_017195	3	rno-miR-23a-3p|rno-miR-23b-3p|rno-miR-152-3p
NM_001244805	2	rno-miR-23a-3p|rno-miR-23b-3p
NM_001107541	2	rno-miR-23a-3p|rno-miR-23b-3p
NM_013006	3	rno-miR-23a-3p|rno-miR-23b-3p|rno-miR-200b-3p
NM_001109613	2	rno-miR-23a-3p|rno-miR-23b-3p
NM_001107021	2	rno-miR-23a-3p|rno-miR-23b-3p
NM_053700	2	rno-miR-23a-3p|rno-miR-23b-3p
NM_001204053	3	rno-miR-23a-3p|rno-miR-23b-3p|rno-miR-30c-5p
NM_001134702	2	rno-miR-23a-3p|rno-miR-23b-3p
NM_013085	2	rno-miR-23a-3p|rno-miR-23b-3p
NM_001193292	2	rno-miR-23a-3p|rno-miR-23b-3p
NM_001024332	5	rno-miR-23a-3p|rno-miR-23b-3p|rno-miR-320-3p|rno-miR-378a-3p|rno-miR-152-3p
NM_001107966	2	rno-miR-23a-3p|rno-miR-23b-3p
NM_001109200	5	rno-miR-23a-3p|rno-miR-23b-3p|rno-miR-27b-3p|rno-miR-27a-3p|rno-miR-29a-3p
NM_001106619	2	rno-miR-23a-3p|rno-miR-23b-3p
NM_001106478	2	rno-miR-23a-3p|rno-miR-23b-3p
NM_001109421	2	rno-miR-23a-3p|rno-miR-23b-3p
NM_001014245	5	rno-miR-23a-3p|rno-miR-23b-3p|rno-miR-16-5p|rno-miR-195-5p|rno-miR-15b-5p
NM_001037347	3	rno-miR-23a-3p|rno-miR-23b-3p|rno-miR-30c-5p
NM_031841	5	rno-miR-23a-3p|rno-miR-23b-3p|rno-miR-199a-3p|rno-miR-378a-3p|rno-miR-200b-3p
NM_080888	5	rno-miR-23a-3p|rno-miR-23b-3p|rno-miR-320-3p|rno-miR-200b-3p|rno-miR-30c-5p

NM_001108120	8	rno-miR-23a-3p|rno-miR-23b-3p|rno-miR-6216|rno-miR-320-3p|rno-miR-199a-3p|rno-miR-27b-3p|rno-miR-378a-3p|rno-miR-27a-3p
NM_001106972	7	rno-miR-23a-3p|rno-miR-23b-3p|rno-miR-16-5p|rno-miR-195-5p|rno-miR-29a-3p|rno-miR-200b-3p|rno-miR-15b-5p
NM_001198653	4	rno-miR-23a-3p|rno-miR-23b-3p|rno-miR-22-3p|rno-miR-378a-3p
NM_001198638	5	rno-miR-23a-3p|rno-miR-23b-3p|rno-miR-16-5p|rno-miR-195-5p|rno-miR-15b-5p
NM_001106428	5	rno-miR-23a-3p|rno-miR-23b-3p|rno-miR-16-5p|rno-miR-195-5p|rno-miR-15b-5p
NM_001106171	2	rno-miR-23a-3p|rno-miR-23b-3p
NM_022585	5	rno-miR-23a-3p|rno-miR-23b-3p|rno-miR-6216|rno-miR-320-3p|rno-miR-30c-5p
NM_053772	7	rno-miR-23a-3p|rno-miR-23b-3p|rno-miR-27b-3p|rno-miR-27a-3p|rno-miR-200b-3p|rno-miR-17-5p|rno-miR-93-5p
NM_001271181	2	rno-miR-23a-3p|rno-miR-23b-3p
NM_001100837	4	rno-miR-23a-3p|rno-miR-23b-3p|rno-miR-205|rno-miR-181a-5p
NM_001108348	2	rno-miR-23a-3p|rno-miR-23b-3p
NM_001305181	10	rno-miR-23a-3p|rno-miR-23b-3p|rno-miR-24-3p|rno-miR-16-5p|rno-miR-103-3p|rno-miR-107-3p|rno-miR-320-3p|rno-miR-195-5p|rno-miR-15b-5p|rno-miR-151-5p
NM_017020	4	rno-miR-23a-3p|rno-miR-23b-3p|rno-miR-27b-3p|rno-miR-27a-3p
NM_001270872	2	rno-miR-23a-3p|rno-miR-23b-3p
NM_001113542	2	rno-miR-23a-3p|rno-miR-23b-3p
NM_001014202	7	rno-miR-23a-3p|rno-miR-23b-3p|rno-miR-320-3p|rno-miR-27b-3p|rno-miR-27a-3p|rno-miR-181a-5p|rno-miR-31a-5p
NM_001012018	4	rno-miR-23a-3p|rno-miR-23b-3p|rno-miR-205|rno-miR-181a-5p
NM_001106186	7	rno-miR-23a-3p|rno-miR-23b-3p|rno-miR-6216|rno-miR-125b-5p|rno-miR-125a-5p|rno-miR-17-5p|rno-miR-93-5p
NM_001108367	2	rno-miR-23a-3p|rno-miR-23b-3p
NM_001135779	2	rno-miR-23a-3p|rno-miR-23b-3p

NM_057103	2	rno-miR-23a-3p|rno-miR-23b-3p
NM_001008762	2	rno-miR-23a-3p|rno-miR-23b-3p
NM_001105960	4	rno-miR-23a-3p|rno-miR-23b-3p|rno-miR-30c-5p|rno-let-7f-5p
NM_001106923	4	rno-miR-23a-3p|rno-miR-23b-3p|rno-miR-181a-5p|rno-miR-152-3p
NM_012781	3	rno-miR-23a-3p|rno-miR-23b-3p|rno-miR-181a-5p
NM_001106125	2	rno-miR-23a-3p|rno-miR-23b-3p
NM_053766	2	rno-miR-23a-3p|rno-miR-23b-3p
NM_001195559	7	rno-miR-23a-3p|rno-miR-23b-3p|rno-miR-27b-3p|rno-miR-27a-3p|rno-miR-30c-5p|rno-miR-17-5p|rno-miR-93-5p
NM_001105974	3	rno-miR-23a-3p|rno-miR-23b-3p|rno-miR-205
NM_144757	3	rno-miR-23a-3p|rno-miR-23b-3p|rno-miR-24-3p
NM_001304365	2	rno-miR-23a-3p|rno-miR-23b-3p
NM_012550	4	rno-miR-23a-3p|rno-miR-23b-3p|rno-miR-6216|rno-miR-30c-5p
NM_001105888	3	rno-miR-23a-3p|rno-miR-23b-3p|rno-miR-6216
NM_001008774	5	rno-miR-23a-3p|rno-miR-23b-3p|rno-miR-27b-3p|rno-miR-27a-3p|rno-miR-181a-5p
NM_001106943	2	rno-miR-23a-3p|rno-miR-23b-3p
NM_001107728	3	rno-miR-23a-3p|rno-miR-23b-3p|rno-miR-29a-3p
NM_212489	3	rno-miR-23a-3p|rno-miR-23b-3p|rno-miR-181a-5p
NM_001302212	8	rno-miR-23a-3p|rno-miR-23b-3p|rno-miR-16-5p|rno-miR-27b-3p|rno-miR-195-5p|rno-miR-27a-3p|rno-miR-200b-3p|rno-miR-15b-5p
NM_134355	3	rno-miR-23a-3p|rno-miR-23b-3p|rno-miR-29a-3p
NM_001025682	2	rno-miR-23a-3p|rno-miR-23b-3p
NM_031131	3	rno-miR-23a-3p|rno-miR-23b-3p|rno-miR-6216
NM_031346	3	rno-miR-23a-3p|rno-miR-23b-3p|rno-miR-30c-5p
NM_001108538	3	rno-miR-23a-3p|rno-miR-23b-3p|rno-miR-181a-5p
NM_001107632	2	rno-miR-23a-3p|rno-miR-23b-3p
NM_001106722	6	rno-miR-23a-3p|rno-miR-23b-3p|rno-miR-6216|rno-miR-103-3p|rno-miR-107-3p|rno-miR-181a-5p

NM_171992	7	rno-miR-23a-3p|rno-miR-23b-3p|rno-miR-16-5p|rno-miR-195-5p|rno-miR-17-5p|rno-miR-15b-5p|rno-miR-93-5p
NM_053716	2	rno-miR-23a-3p|rno-miR-23b-3p
NM_001107549	4	rno-miR-23a-3p|rno-miR-23b-3p|rno-miR-30c-5p|rno-miR-152-3p
NM_022394	2	rno-miR-23a-3p|rno-miR-23b-3p
NM_001012742	8	rno-miR-23a-3p|rno-miR-23b-3p|rno-miR-6216|rno-miR-16-5p|rno-miR-27b-3p|rno-miR-195-5p|rno-miR-27a-3p|rno-miR-15b-5p
NM_001002813	2	rno-miR-23a-3p|rno-miR-23b-3p
NM_053977	2	rno-miR-23a-3p|rno-miR-23b-3p
NM_053647	2	rno-miR-23a-3p|rno-miR-23b-3p
NM_001013989	2	rno-miR-23a-3p|rno-miR-23b-3p
NM_022541	2	rno-miR-23a-3p|rno-miR-23b-3p
NM_080903	3	rno-miR-23a-3p|rno-miR-23b-3p|rno-miR-29a-3p
NM_012773	2	rno-miR-23a-3p|rno-miR-23b-3p
NM_031720	11	rno-miR-23a-3p|rno-miR-23b-3p|rno-miR-6216|rno-miR-16-5p|rno-miR-320-3p|rno-miR-199a-3p|rno-miR-195-5p|rno-miR-29a-3p|rno-miR-181a-5p|rno-miR-30c-5p|rno-miR-15b-5p
NM_001109127	2	rno-miR-23a-3p|rno-miR-23b-3p
NM_001013997	2	rno-miR-23a-3p|rno-miR-23b-3p
NM_001004273	2	rno-miR-23a-3p|rno-miR-23b-3p
NM_001106524	2	rno-miR-23a-3p|rno-miR-23b-3p
NM_145785	4	rno-miR-23a-3p|rno-miR-23b-3p|rno-miR-103-3p|rno-miR-107-3p
NM_001142962	3	rno-miR-23a-3p|rno-miR-23b-3p|rno-miR-181a-5p
NM_001109364	6	rno-miR-23a-3p|rno-miR-23b-3p|rno-miR-16-5p|rno-miR-195-5p|rno-miR-152-3p|rno-miR-15b-5p
NM_024484	2	rno-miR-23a-3p|rno-miR-23b-3p
NM_001106939	3	rno-miR-23a-3p|rno-miR-23b-3p|rno-miR-30c-5p
NM_012869	2	rno-miR-23a-3p|rno-miR-23b-3p
NM_001106808	4	rno-miR-23a-3p|rno-miR-23b-3p|rno-miR-125b-5p|rno-miR-125a-5p
NM_001100514	3	rno-miR-23a-3p|rno-miR-23b-3p|rno-miR-181a-5p

NM_001301664	2	rno-miR-23a-3p|rno-miR-23b-3p
NM_001161809	3	rno-miR-23a-3p|rno-miR-23b-3p|rno-miR-29a-3p
NM_012887	4	rno-miR-23a-3p|rno-miR-23b-3p|rno-miR-6216|rno-miR-30c-5p
NM_001109251	2	rno-miR-23a-3p|rno-miR-23b-3p
NM_001107276	2	rno-miR-23a-3p|rno-miR-23b-3p
NM_001107231	4	rno-miR-23a-3p|rno-miR-23b-3p|rno-miR-27b-3p|rno-miR-27a-3p
NM_001135157	2	rno-miR-23a-3p|rno-miR-23b-3p
NM_001134341	3	rno-miR-23a-3p|rno-miR-23b-3p|rno-miR-200b-3p
NM_001107360	3	rno-miR-23a-3p|rno-miR-23b-3p|rno-miR-126a-3p
NM_001277209	2	rno-miR-23a-3p|rno-miR-23b-3p
NM_001108407	2	rno-miR-23a-3p|rno-miR-23b-3p
NM_001033674	5	rno-miR-23a-3p|rno-miR-23b-3p|rno-miR-320-3p|rno-miR-17-5p|rno-miR-93-5p
NM_001034188	2	rno-miR-23a-3p|rno-miR-23b-3p
NM_001106332	2	rno-miR-23a-3p|rno-miR-23b-3p
NM_001270654	3	rno-miR-23a-3p|rno-miR-23b-3p|rno-miR-6216
NM_033376	2	rno-miR-23a-3p|rno-miR-23b-3p
NM_001191732	2	rno-miR-23a-3p|rno-miR-23b-3p
NM_001191630	3	rno-miR-23a-3p|rno-miR-23b-3p|rno-miR-30c-5p
NM_001106402	4	rno-miR-23a-3p|rno-miR-23b-3p|rno-miR-24-3p|rno-miR-205
NM_001191105	3	rno-miR-23a-3p|rno-miR-23b-3p|rno-miR-31a-5p
NM_012655	6	rno-miR-23a-3p|rno-miR-23b-3p|rno-miR-24-3p|rno-miR-320-3p|rno-miR-199a-3p|rno-miR-29a-3p
NM_001108351	3	rno-miR-23a-3p|rno-miR-23b-3p|rno-miR-181a-5p
NM_012739	3	rno-miR-23a-3p|rno-miR-23b-3p|rno-miR-30c-5p
NM_053847	5	rno-miR-23a-3p|rno-miR-23b-3p|rno-miR-6216|rno-miR-17-5p|rno-miR-93-5p
NM_031798	6	rno-miR-23a-3p|rno-miR-23b-3p|rno-miR-6216|rno-miR-16-5p|rno-miR-195-5p|rno-miR-15b-5p
NM_001033909	3	rno-miR-23a-3p|rno-miR-23b-3p|rno-miR-29a-3p

NM_001170577	4	rno-miR-23a-3p|rno-miR-23b-3p|rno-miR-24-3p|rno-miR-378a-3p
NM_138888	2	rno-miR-23a-3p|rno-miR-23b-3p
NM_001271280	3	rno-miR-23a-3p|rno-miR-23b-3p|rno-miR-24-3p
NM_133563	2	rno-miR-23a-3p|rno-miR-23b-3p
NM_001191710	10	rno-miR-23a-3p|rno-miR-23b-3p|rno-miR-16-5p|rno-miR-320-3p|rno-miR-22-3p|rno-miR-195-5p|rno-miR-31a-5p|rno-miR-17-5p|rno-miR-15b-5p|rno-miR-93-5p
NM_172032	2	rno-miR-23a-3p|rno-miR-23b-3p
NM_001108881	7	rno-miR-23a-3p|rno-miR-23b-3p|rno-miR-6216|rno-miR-16-5p|rno-miR-195-5p|rno-miR-31a-5p|rno-miR-15b-5p
NM_001134608	4	rno-miR-23a-3p|rno-miR-23b-3p|rno-miR-27b-3p|rno-miR-27a-3p
NM_019907	5	rno-miR-23a-3p|rno-miR-23b-3p|rno-miR-16-5p|rno-miR-195-5p|rno-miR-15b-5p
NM_001007660	2	rno-miR-23a-3p|rno-miR-23b-3p
NM_001109391	2	rno-miR-23a-3p|rno-miR-23b-3p
NM_053810	4	rno-miR-23a-3p|rno-miR-23b-3p|rno-miR-27b-3p|rno-miR-27a-3p
NM_001107949	2	rno-miR-23a-3p|rno-miR-23b-3p
NM_001039005	2	rno-miR-23a-3p|rno-miR-23b-3p
NM_001106715	4	rno-miR-23a-3p|rno-miR-23b-3p|rno-miR-6216|rno-miR-200b-3p
NM_017256	7	rno-miR-23a-3p|rno-miR-23b-3p|rno-miR-16-5p|rno-miR-103-3p|rno-miR-107-3p|rno-miR-195-5p|rno-miR-15b-5p
NM_080400	5	rno-miR-23a-3p|rno-miR-23b-3p|rno-miR-16-5p|rno-miR-195-5p|rno-miR-15b-5p
NM_001134995	3	rno-miR-23a-3p|rno-miR-23b-3p|rno-miR-30c-5p
NM_001108797	9	rno-miR-23a-3p|rno-miR-23b-3p|rno-miR-103-3p|rno-miR-107-3p|rno-miR-320-3p|rno-miR-200b-3p|rno-miR-30c-5p|rno-miR-17-5p|rno-miR-93-5p
NM_001191925	2	rno-miR-23a-3p|rno-miR-23b-3p
NM_001039539	8	rno-miR-23a-3p|rno-miR-23b-3p|rno-miR-6216|rno-miR-27b-3p|rno-miR-205|rno-miR-27a-3p|rno-miR-181a-5p|rno-miR-30c-5p

NM_001025046	7	rno-miR-23a-3p|rno-miR-23b-3p|rno-miR-125b-5p|rno-miR-125a-5p|rno-miR-29a-3p|rno-miR-200b-3p|rno-miR-30c-5p
NM_001007619	3	rno-miR-23a-3p|rno-miR-23b-3p|rno-miR-22-3p
NM_001191089	5	rno-miR-23a-3p|rno-miR-23b-3p|rno-miR-125b-5p|rno-miR-125a-5p|rno-miR-181a-5p
NM_001008278	2	rno-miR-23a-3p|rno-miR-23b-3p
NM_001004085	3	rno-miR-23a-3p|rno-miR-23b-3p|rno-miR-24-3p
NM_001108373	3	rno-miR-23a-3p|rno-miR-23b-3p|rno-miR-6216
NM_207600	2	rno-miR-23a-3p|rno-miR-23b-3p
NM_001003403	2	rno-miR-23a-3p|rno-miR-23b-3p
NM_001107414	4	rno-miR-23a-3p|rno-miR-23b-3p|rno-miR-27b-3p|rno-miR-27a-3p
NM_001108124	4	rno-miR-23a-3p|rno-miR-23b-3p|rno-miR-17-5p|rno-miR-93-5p
NM_053705	3	rno-miR-23a-3p|rno-miR-23b-3p|rno-miR-30c-5p
NM_001105738	2	rno-miR-23a-3p|rno-miR-23b-3p
NM_013074	2	rno-miR-23a-3p|rno-miR-23b-3p
NM_001009369	2	rno-miR-23a-3p|rno-miR-23b-3p
NM_001007700	4	rno-miR-23a-3p|rno-miR-23b-3p|rno-miR-125b-5p|rno-miR-125a-5p
NM_001108677	9	rno-miR-23a-3p|rno-miR-23b-3p|rno-miR-6216|rno-miR-125b-5p|rno-miR-125a-5p|rno-miR-181a-5p|rno-miR-17-5p|rno-miR-152-3p|rno-miR-93-5p
NM_001257349	3	rno-miR-23a-3p|rno-miR-23b-3p|rno-miR-3473
NM_019126	3	rno-miR-23a-3p|rno-miR-23b-3p|rno-miR-6216
NM_001014036	3	rno-miR-23a-3p|rno-miR-23b-3p|rno-miR-24-3p
NM_001271107	2	rno-miR-23a-3p|rno-miR-23b-3p
NM_012913	2	rno-miR-23a-3p|rno-miR-23b-3p
NM_001109253	5	rno-miR-23a-3p|rno-miR-23b-3p|rno-miR-24-3p|rno-miR-125b-5p|rno-miR-125a-5p
NM_019282	5	rno-miR-23a-3p|rno-miR-23b-3p|rno-miR-6216|rno-miR-27b-3p|rno-miR-27a-3p
NM_052804	3	rno-miR-23a-3p|rno-miR-23b-3p|rno-miR-152-3p

NM_001037769	2	rno-miR-23a-3p|rno-miR-23b-3p
NM_001107734	5	rno-miR-23a-3p|rno-miR-23b-3p|rno-miR-16-5p|rno-miR-195-5p|rno-miR-15b-5p
NM_001105900	6	rno-miR-23a-3p|rno-miR-23b-3p|rno-miR-6216|rno-miR-200b-3p|rno-miR-181a-5p|rno-miR-152-3p
NM_001012185	6	rno-miR-23a-3p|rno-miR-23b-3p|rno-miR-16-5p|rno-miR-3473|rno-miR-195-5p|rno-miR-15b-5p
NM_001004442	5	rno-miR-23a-3p|rno-miR-23b-3p|rno-miR-16-5p|rno-miR-195-5p|rno-miR-15b-5p
NM_001271104	4	rno-miR-23a-3p|rno-miR-23b-3p|rno-miR-27b-3p|rno-miR-27a-3p
NM_153310	2	rno-miR-23a-3p|rno-miR-23b-3p
NM_001014787	2	rno-miR-23a-3p|rno-miR-23b-3p
NM_001106844	2	rno-miR-23a-3p|rno-miR-23b-3p
NM_001105742	8	rno-miR-23a-3p|rno-miR-23b-3p|rno-miR-6216|rno-miR-27b-3p|rno-miR-27a-3p|rno-miR-31a-5p|rno-miR-17-5p|rno-miR-93-5p
NM_001014191	5	rno-miR-23a-3p|rno-miR-23b-3p|rno-miR-6216|rno-miR-200b-3p|rno-miR-152-3p
NM_198765	4	rno-miR-23a-3p|rno-miR-23b-3p|rno-miR-17-5p|rno-miR-93-5p
NM_057139	3	rno-miR-23a-3p|rno-miR-23b-3p|rno-miR-200b-3p
NM_022380	2	rno-miR-23a-3p|rno-miR-23b-3p
NM_134459	3	rno-miR-23a-3p|rno-miR-23b-3p|rno-miR-30c-5p
NM_019251	2	rno-miR-23a-3p|rno-miR-23b-3p
NM_138509	6	rno-miR-23a-3p|rno-miR-23b-3p|rno-miR-199a-3p|rno-miR-30c-5p|rno-miR-17-5p|rno-miR-93-5p
NM_001109574	4	rno-miR-23a-3p|rno-miR-23b-3p|rno-miR-320-3p|rno-miR-29a-3p
NM_001302208	8	rno-miR-23a-3p|rno-miR-23b-3p|rno-miR-16-5p|rno-miR-27b-3p|rno-miR-195-5p|rno-miR-27a-3p|rno-miR-200b-3p|rno-miR-15b-5p
NM_001107460	2	rno-miR-23a-3p|rno-miR-23b-3p

NM_001173450	10	rno-miR-23a-3p|rno-miR-23b-3p|rno-miR-6216|rno-miR-125b-5p|rno-miR-125a-5p|rno-miR-205|rno-miR-181a-5p|rno-miR-30c-5p|rno-miR-17-5p|rno-miR-93-5p
NM_053363	11	rno-miR-23a-3p|rno-miR-23b-3p|rno-miR-24-3p|rno-miR-16-5p|rno-miR-103-3p|rno-miR-107-3p|rno-miR-27b-3p|rno-miR-195-5p|rno-miR-27a-3p|rno-miR-152-3p|rno-miR-15b-5p
NM_001033899	4	rno-miR-23a-3p|rno-miR-23b-3p|rno-miR-29a-3p|rno-miR-152-3p
NM_001301702	5	rno-miR-23a-3p|rno-miR-23b-3p|rno-miR-6216|rno-miR-320-3p|rno-miR-30c-5p
NM_138912	4	rno-miR-23a-3p|rno-miR-23b-3p|rno-miR-17-5p|rno-miR-93-5p
NM_001014119	2	rno-miR-23a-3p|rno-miR-23b-3p
NM_001101006	2	rno-miR-23a-3p|rno-miR-23b-3p
NM_054010	2	rno-miR-23a-3p|rno-miR-23b-3p
NM_001191659	4	rno-miR-23a-3p|rno-miR-23b-3p|rno-miR-24-3p|rno-miR-320-3p
NM_031783	3	rno-miR-23a-3p|rno-miR-23b-3p|rno-miR-30c-5p
NM_013065	3	rno-miR-23a-3p|rno-miR-23b-3p|rno-miR-152-3p
NM_052981	2	rno-miR-23a-3p|rno-miR-23b-3p
NM_001015020	2	rno-miR-23a-3p|rno-miR-23b-3p
NM_022260	2	rno-miR-23a-3p|rno-miR-23b-3p
NM_053663	4	rno-miR-23a-3p|rno-miR-23b-3p|rno-miR-103-3p|rno-miR-107-3p
NM_001108923	3	rno-miR-23a-3p|rno-miR-23b-3p|rno-miR-181a-5p
NM_001025136	3	rno-miR-23a-3p|rno-miR-23b-3p|rno-miR-6216
NM_001109210	5	rno-miR-23a-3p|rno-miR-23b-3p|rno-miR-31a-5p|rno-miR-17-5p|rno-miR-93-5p
NM_001100531	4	rno-miR-23a-3p|rno-miR-23b-3p|rno-miR-24-3p|rno-miR-320-3p
NM_053842	7	rno-miR-23a-3p|rno-miR-23b-3p|rno-miR-320-3p|rno-miR-27b-3p|rno-miR-378a-3p|rno-miR-27a-3p|rno-miR-181a-5p
NM_182953	4	rno-miR-23a-3p|rno-miR-23b-3p|rno-miR-27b-3p|rno-miR-27a-3p
NM_181366	2	rno-miR-23a-3p|rno-miR-23b-3p
NM_001277057	3	rno-miR-23a-3p|rno-miR-23b-3p|rno-miR-30c-5p

NM_145723	2	rno-miR-23a-3p|rno-miR-23b-3p
NM_001108628	8	rno-miR-23a-3p|rno-miR-23b-3p|rno-miR-16-5p|rno-miR-103-3p|rno-miR-107-3p|rno-miR-195-5p|rno-miR-378a-3p|rno-miR-15b-5p
NM_001108662	2	rno-miR-23a-3p|rno-miR-23b-3p
NM_001135119	2	rno-miR-23a-3p|rno-miR-23b-3p
NM_001256509	4	rno-miR-23a-3p|rno-miR-23b-3p|rno-miR-27b-3p|rno-miR-27a-3p
NM_012829	2	rno-miR-23a-3p|rno-miR-23b-3p
NM_001009675	2	rno-miR-23a-3p|rno-miR-23b-3p
NM_001302210	8	rno-miR-23a-3p|rno-miR-23b-3p|rno-miR-16-5p|rno-miR-27b-3p|rno-miR-195-5p|rno-miR-27a-3p|rno-miR-200b-3p|rno-miR-15b-5p
NM_001107409	2	rno-miR-23a-3p|rno-miR-23b-3p
NM_001108186	5	rno-miR-23a-3p|rno-miR-23b-3p|rno-miR-16-5p|rno-miR-195-5p|rno-miR-15b-5p
NM_001106307	2	rno-miR-23a-3p|rno-miR-23b-3p
NM_001106720	2	rno-miR-23a-3p|rno-miR-23b-3p
NM_133519	2	rno-miR-23a-3p|rno-miR-23b-3p
NM_145789	3	rno-miR-23a-3p|rno-miR-23b-3p|rno-miR-30c-5p
NM_001270871	2	rno-miR-23a-3p|rno-miR-23b-3p
NM_199394	2	rno-miR-23a-3p|rno-miR-23b-3p
NM_203338	6	rno-miR-23a-3p|rno-miR-23b-3p|rno-miR-6216|rno-miR-3473|rno-miR-320-3p|rno-miR-30c-5p
NM_001109297	4	rno-miR-23a-3p|rno-miR-23b-3p|rno-miR-17-5p|rno-miR-93-5p
NM_001107759	2	rno-miR-23a-3p|rno-miR-23b-3p
NM_001106518	2	rno-miR-23a-3p|rno-miR-23b-3p
NM_031237	3	rno-miR-23a-3p|rno-miR-23b-3p|rno-miR-152-3p
NM_001106893	4	rno-miR-23a-3p|rno-miR-23b-3p|rno-miR-27b-3p|rno-miR-27a-3p
NM_001034135	2	rno-miR-23a-3p|rno-miR-23b-3p
NM_173141	2	rno-miR-23a-3p|rno-miR-23b-3p
NM_001015010	4	rno-miR-23a-3p|rno-miR-23b-3p|rno-miR-17-5p|rno-miR-93-5p
NM_001024370	2	rno-miR-23a-3p|rno-miR-23b-3p

NM_001135819	2	rno-miR-23a-3p|rno-miR-23b-3p
NM_001008886	4	rno-miR-23a-3p|rno-miR-23b-3p|rno-miR-24-3p|rno-miR-152-3p
NM_001012026	3	rno-miR-23a-3p|rno-miR-23b-3p|rno-miR-200b-3p
NM_001014793	3	rno-miR-23a-3p|rno-miR-23b-3p|rno-miR-181a-5p
NM_001109031	2	rno-miR-23a-3p|rno-miR-23b-3p
NM_001136261	3	rno-miR-23a-3p|rno-miR-23b-3p|rno-miR-31a-5p
NM_001170602	2	rno-miR-23a-3p|rno-miR-23b-3p
NM_134457	3	rno-miR-23a-3p|rno-miR-23b-3p|rno-miR-30c-5p
NM_001106759	2	rno-miR-23a-3p|rno-miR-23b-3p
NM_133517	2	rno-miR-23a-3p|rno-miR-23b-3p
NM_001009693	2	rno-miR-23a-3p|rno-miR-23b-3p
NM_053773	3	rno-miR-23a-3p|rno-miR-23b-3p|rno-miR-151-5p
NM_173154	2	rno-miR-23a-3p|rno-miR-23b-3p
NM_147138	2	rno-miR-23a-3p|rno-miR-23b-3p
NM_001105907	2	rno-miR-23a-3p|rno-miR-23b-3p
NM_001109575	6	rno-miR-23a-3p|rno-miR-23b-3p|rno-miR-16-5p|rno-miR-195-5p|rno-miR-30c-5p|rno-miR-15b-5p
NM_001126276	2	rno-miR-23a-3p|rno-miR-23b-3p
NM_001037981	2	rno-miR-23a-3p|rno-miR-23b-3p
NM_001107773	2	rno-miR-23a-3p|rno-miR-23b-3p
NM_134408	8	rno-miR-23a-3p|rno-miR-23b-3p|rno-miR-16-5p|rno-miR-27b-3p|rno-miR-195-5p|rno-miR-27a-3p|rno-miR-200b-3p|rno-miR-15b-5p
NM_031068	2	rno-miR-23a-3p|rno-miR-23b-3p
NM_001308265	5	rno-miR-23a-3p|rno-miR-23b-3p|rno-miR-199a-3p|rno-miR-205|rno-miR-200b-3p
NM_001107800	3	rno-miR-23a-3p|rno-miR-23b-3p|rno-miR-29a-3p
NM_001106842	2	rno-miR-23a-3p|rno-miR-23b-3p
NM_001008768	2	rno-miR-23a-3p|rno-miR-23b-3p
NM_001173426	5	rno-miR-23a-3p|rno-miR-23b-3p|rno-miR-27b-3p|rno-miR-205|rno-miR-27a-3p

NM_001109492	3	rno-miR-23a-3p|rno-miR-23b-3p|rno-miR-200b-3p
NM_030849	7	rno-miR-23a-3p|rno-miR-23b-3p|rno-miR-16-5p|rno-miR-27b-3p|rno-miR-195-5p|rno-miR-27a-3p|rno-miR-15b-5p
NM_001024778	4	rno-miR-23a-3p|rno-miR-23b-3p|rno-miR-205|rno-miR-200b-3p
NM_001024275	6	rno-miR-23a-3p|rno-miR-23b-3p|rno-miR-24-3p|rno-miR-125b-5p|rno-miR-125a-5p|rno-miR-151-5p
NM_001127635	5	rno-miR-23a-3p|rno-miR-23b-3p|rno-miR-181a-5p|rno-miR-17-5p|rno-miR-93-5p
NM_080397	2	rno-miR-23a-3p|rno-miR-23b-3p
NM_001013248	3	rno-miR-23a-3p|rno-miR-23b-3p|rno-miR-30c-5p
NM_001014117	3	rno-miR-23a-3p|rno-miR-23b-3p|rno-miR-3473
NM_001108018	6	rno-miR-23a-3p|rno-miR-23b-3p|rno-miR-125b-5p|rno-miR-125a-5p|rno-miR-199a-3p|rno-miR-152-3p
NM_001107367	3	rno-miR-23a-3p|rno-miR-23b-3p|rno-miR-200b-3p
NM_001108939	3	rno-miR-23a-3p|rno-miR-23b-3p|rno-miR-29a-3p
NM_198745	2	rno-miR-23a-3p|rno-miR-23b-3p
NM_001034010	7	rno-miR-23a-3p|rno-miR-23b-3p|rno-miR-125b-5p|rno-miR-125a-5p|rno-miR-27b-3p|rno-miR-27a-3p|rno-miR-30c-5p
NM_001170558	5	rno-miR-23a-3p|rno-miR-23b-3p|rno-miR-103-3p|rno-miR-107-3p|rno-miR-181a-5p
NM_031741	1	rno-miR-24-3p
NM_001126082	1	rno-miR-24-3p
NM_001011919	1	rno-miR-24-3p
NM_001024256	1	rno-miR-24-3p
NM_022180	2	rno-miR-24-3p|rno-miR-152-3p
NM_001109506	3	rno-miR-24-3p|rno-miR-200b-3p|rno-miR-30c-5p
NM_001172151	1	rno-miR-24-3p
NM_013045	2	rno-miR-24-3p|rno-miR-205
NM_001168542	1	rno-miR-24-3p
NM_001007730	2	rno-miR-24-3p|rno-miR-152-3p

NM_031779	3	rno-miR-24-3p|rno-miR-103-3p|rno-miR-107-3p
NM_001106238	3	rno-miR-24-3p|rno-miR-199a-3p|rno-miR-181a-5p
NM_001025014	1	rno-miR-24-3p
NM_001134969	1	rno-miR-24-3p
NM_001135875	2	rno-miR-24-3p|rno-miR-200b-3p
NM_001109294	2	rno-miR-24-3p|rno-miR-152-3p
NM_001127455	2	rno-miR-24-3p|rno-miR-31a-5p
NM_001305447	1	rno-miR-24-3p
NM_001014185	1	rno-miR-24-3p
NM_199390	1	rno-miR-24-3p
NM_001008509	4	rno-miR-24-3p|rno-miR-6216|rno-miR-27b-3p|rno-miR-27a-3p
NM_001106357	5	rno-miR-24-3p|rno-miR-16-5p|rno-miR-195-5p|rno-miR-30c-5p|rno-miR-15b-5p
NM_013013	2	rno-miR-24-3p|rno-miR-181a-5p
NM_001107486	1	rno-miR-24-3p
NM_001008333	1	rno-miR-24-3p
NM_001107678	2	rno-miR-24-3p|rno-miR-6216
NM_172042	3	rno-miR-24-3p|rno-miR-27b-3p|rno-miR-27a-3p
NM_021664	1	rno-miR-24-3p
NM_001276487	1	rno-miR-24-3p
NM_001270789	2	rno-miR-24-3p|rno-miR-31a-5p
NM_001195488	1	rno-miR-24-3p
NM_001107581	1	rno-miR-24-3p
NM_001134763	1	rno-miR-24-3p
NM_001025066	1	rno-miR-24-3p
NM_001108947	5	rno-miR-24-3p|rno-miR-27b-3p|rno-miR-27a-3p|rno-miR-200b-3p|rno-miR-152-3p
NM_001109338	6	rno-miR-24-3p|rno-miR-320-3p|rno-miR-27b-3p|rno-miR-27a-3p|rno-miR-30c-5p|rno-miR-151-5p
NM_001025691	4	rno-miR-24-3p|rno-miR-16-5p|rno-miR-195-5p|rno-miR-15b-5p

NM_001108135	1	rno-miR-24-3p
NM_019368	3	rno-miR-24-3p|rno-miR-205|rno-miR-29a-3p
NM_001163492	1	rno-miR-24-3p
NM_001008371	2	rno-miR-24-3p|rno-miR-320-3p
NM_001039012	1	rno-miR-24-3p
NM_001191822	1	rno-miR-24-3p
NM_001108335	1	rno-miR-24-3p
NM_001107363	1	rno-miR-24-3p
NM_001107850	1	rno-miR-24-3p
NM_017007	1	rno-miR-24-3p
NM_001011905	1	rno-miR-24-3p
NM_013222	1	rno-miR-24-3p
NM_001164297	1	rno-miR-24-3p
NM_212499	4	rno-miR-24-3p|rno-miR-16-5p|rno-miR-195-5p|rno-miR-15b-5p
NM_001034015	4	rno-miR-24-3p|rno-miR-29a-3p|rno-miR-181a-5p|rno-miR-30c-5p
NM_001025667	2	rno-miR-24-3p|rno-miR-6216
NM_033352	3	rno-miR-24-3p|rno-miR-6216|rno-miR-30c-5p
NM_001012068	1	rno-miR-24-3p
NM_001143817	1	rno-miR-24-3p
NM_001271101	4	rno-miR-24-3p|rno-miR-16-5p|rno-miR-195-5p|rno-miR-15b-5p
NM_001313940	1	rno-miR-24-3p
NM_001107779	1	rno-miR-24-3p
NM_001301646	1	rno-miR-24-3p
NM_001164307	1	rno-miR-24-3p
NM_012719	2	rno-miR-24-3p|rno-miR-6216
NM_019331	3	rno-miR-24-3p|rno-miR-17-5p|rno-miR-93-5p
NM_001191765	11	rno-miR-24-3p|rno-miR-16-5p|rno-miR-103-3p|rno-miR-107-3p|rno-miR-199a-3p|rno-miR-195-5p|rno-miR-30c-5p|rno-miR-17-5p|rno-miR-152-3p|rno-miR-15b-5p|rno-miR-93-5p
NM_024361	2	rno-miR-24-3p|rno-miR-200b-3p

NM_001107187	3	rno-miR-24-3p|rno-miR-125b-5p|rno-miR-125a-5p
NM_001004244	1	rno-miR-24-3p
NM_053322	6	rno-miR-24-3p|rno-miR-16-5p|rno-miR-125b-5p|rno-miR-125a-5p|rno-miR-195-5p|rno-miR-15b-5p
NM_139339	1	rno-miR-24-3p
NM_001109520	1	rno-miR-24-3p
NM_001106396	7	rno-miR-24-3p|rno-miR-16-5p|rno-miR-27b-3p|rno-miR-195-5p|rno-miR-27a-3p|rno-miR-200b-3p|rno-miR-15b-5p
NM_152242	1	rno-miR-24-3p
NM_001012345	1	rno-miR-24-3p
NM_001039699	4	rno-miR-24-3p|rno-miR-125b-5p|rno-miR-125a-5p|rno-miR-30c-5p
NM_001106876	2	rno-miR-24-3p|rno-miR-22-3p
NM_130739	4	rno-miR-24-3p|rno-miR-16-5p|rno-miR-195-5p|rno-miR-15b-5p
NM_001007707	1	rno-miR-24-3p
NM_001107475	4	rno-miR-24-3p|rno-miR-125b-5p|rno-miR-125a-5p|rno-miR-29a-3p
NM_031541	3	rno-miR-24-3p|rno-miR-125b-5p|rno-miR-125a-5p
NM_001009706	1	rno-miR-24-3p
NM_001271248	4	rno-miR-24-3p|rno-miR-16-5p|rno-miR-195-5p|rno-miR-15b-5p
NM_001270790	2	rno-miR-24-3p|rno-miR-31a-5p
NM_001009503	1	rno-miR-24-3p
NM_001177321	1	rno-miR-24-3p
NM_001001508	4	rno-miR-24-3p|rno-miR-200b-3p|rno-miR-181a-5p|rno-miR-30c-5p
NM_001024744	1	rno-miR-24-3p
NM_001107356	1	rno-miR-24-3p
NM_001170455	5	rno-miR-24-3p|rno-miR-320-3p|rno-miR-181a-5p|rno-miR-17-5p|rno-miR-93-5p
NM_001164142	2	rno-miR-24-3p|rno-miR-205
NM_001014082	5	rno-miR-24-3p|rno-miR-125b-5p|rno-miR-125a-5p|rno-miR-22-3p|rno-miR-152-3p
NM_001164143	2	rno-miR-24-3p|rno-miR-205

NM_001014141	1	rno-miR-24-3p
NM_001006973	1	rno-miR-24-3p
NM_001008297	3	rno-miR-24-3p|rno-miR-320-3p|rno-miR-29a-3p
NM_053816	4	rno-miR-24-3p|rno-miR-29a-3p|rno-miR-181a-5p|rno-miR-30c-5p
NM_001134613	1	rno-miR-24-3p
NM_001277263	1	rno-miR-24-3p
NM_031147	1	rno-miR-24-3p
NM_031812	1	rno-miR-24-3p
NM_198739	1	rno-miR-24-3p
NM_001012203	4	rno-miR-24-3p|rno-miR-27b-3p|rno-miR-27a-3p|rno-miR-29a-3p
NM_017301	5	rno-miR-24-3p|rno-miR-181a-5p|rno-miR-17-5p|rno-miR-152-3p|rno-miR-93-5p
NM_001164304	1	rno-miR-24-3p
NM_001014260	1	rno-miR-24-3p
NM_001172103	5	rno-miR-24-3p|rno-miR-16-5p|rno-miR-195-5p|rno-miR-200b-3p|rno-miR-15b-5p
NM_012528	1	rno-miR-24-3p
NM_001160314	8	rno-miR-24-3p|rno-miR-16-5p|rno-miR-125b-5p|rno-miR-125a-5p|rno-miR-27b-3p|rno-miR-195-5p|rno-miR-27a-3p|rno-miR-15b-5p
NM_001105998	2	rno-miR-24-3p|rno-miR-31a-5p
NM_001108967	1	rno-miR-24-3p
NM_001128184	1	rno-miR-24-3p
NM_053966	8	rno-miR-24-3p|rno-miR-16-5p|rno-miR-22-3p|rno-miR-195-5p|rno-miR-205|rno-miR-29a-3p|rno-miR-200b-3p|rno-miR-15b-5p
NM_001100561	4	rno-miR-24-3p|rno-miR-191a-5p|rno-miR-205|rno-miR-200b-3p
NM_017280	2	rno-miR-24-3p|rno-miR-29a-3p
NM_001108717	3	rno-miR-24-3p|rno-miR-125b-5p|rno-miR-125a-5p
NM_001277253	1	rno-miR-24-3p
NM_012912	1	rno-miR-24-3p
NM_001107390	2	rno-miR-24-3p|rno-miR-181a-5p

NM_031020	6	rno-miR-24-3p|rno-miR-6216|rno-miR-125b-5p|rno-miR-125a-5p|rno-miR-27b-3p|rno-miR-27a-3p
NM_001106662	1	rno-miR-24-3p
NM_032073	1	rno-miR-24-3p
NM_001013063	2	rno-miR-24-3p|rno-miR-199a-3p
NM_001014792	2	rno-miR-24-3p|rno-miR-31a-5p
NM_012864	1	rno-miR-24-3p
NM_001044252	1	rno-miR-24-3p
NM_001025028	1	rno-miR-24-3p
NM_001271246	8	rno-miR-24-3p|rno-miR-16-5p|rno-miR-125b-5p|rno-miR-125a-5p|rno-miR-27b-3p|rno-miR-195-5p|rno-miR-27a-3p|rno-miR-15b-5p
NM_022187	2	rno-miR-24-3p|rno-miR-320-3p
NM_031531	1	rno-miR-24-3p
NM_001105802	1	rno-miR-24-3p
NM_001107035	1	rno-miR-24-3p
NM_001106365	9	rno-miR-24-3p|rno-miR-191a-5p|rno-miR-16-5p|rno-miR-103-3p|rno-miR-107-3p|rno-miR-320-3p|rno-miR-195-5p|rno-miR-29a-3p|rno-miR-15b-5p
NM_012507	1	rno-miR-24-3p
NM_001143847	1	rno-miR-24-3p
NM_001164302	1	rno-miR-24-3p
NM_001107145	1	rno-miR-24-3p
NM_001277264	1	rno-miR-24-3p
NM_001270933	2	rno-miR-24-3p|rno-miR-152-3p
NM_019208	1	rno-miR-24-3p
NM_017332	4	rno-miR-24-3p|rno-miR-16-5p|rno-miR-195-5p|rno-miR-15b-5p
NM_001106870	3	rno-miR-24-3p|rno-miR-17-5p|rno-miR-93-5p
NM_001017508	1	rno-miR-24-3p
NM_012918	3	rno-miR-24-3p|rno-miR-27b-3p|rno-miR-27a-3p
NM_001108515	4	rno-miR-24-3p|rno-miR-29a-3p|rno-miR-17-5p|rno-miR-93-5p

NM_019623	1	rno-miR-24-3p
NM_001109363	3	rno-miR-24-3p|rno-miR-125b-5p|rno-miR-125a-5p
NM_001014121	1	rno-miR-24-3p
NM_001106071	1	rno-miR-24-3p
NM_001107425	7	rno-miR-24-3p|rno-miR-6216|rno-miR-29a-3p|rno-miR-181a-5p|rno-miR-30c-5p|rno-miR-17-5p|rno-miR-93-5p
NM_001191608	2	rno-miR-24-3p|rno-miR-6216
NM_001106006	3	rno-miR-24-3p|rno-miR-22-3p|rno-miR-31a-5p
NM_001107668	1	rno-miR-24-3p
NM_001107498	3	rno-miR-24-3p|rno-miR-6216|rno-miR-31a-5p
NM_001173432	1	rno-miR-24-3p
NM_012871	2	rno-miR-24-3p|rno-miR-29a-3p
NM_001107271	4	rno-miR-24-3p|rno-miR-191a-5p|rno-miR-125b-5p|rno-miR-125a-5p
NM_001135872	1	rno-miR-24-3p
NM_138516	2	rno-miR-24-3p|rno-miR-200b-3p
NM_001317181	8	rno-miR-24-3p|rno-miR-103-3p|rno-miR-107-3p|rno-miR-199a-3p|rno-miR-30c-5p|rno-miR-17-5p|rno-miR-152-3p|rno-miR-93-5p
NM_017116	1	rno-miR-24-3p
NM_001109446	1	rno-miR-24-3p
NM_001107223	2	rno-miR-24-3p|rno-miR-152-3p
NM_001037496	1	rno-miR-24-3p
NM_001108702	2	rno-miR-24-3p|rno-miR-30c-5p
NM_001108564	1	rno-miR-24-3p
NM_001277251	1	rno-miR-24-3p
NM_022401	1	rno-miR-24-3p
NM_138529	4	rno-miR-24-3p|rno-miR-27b-3p|rno-miR-27a-3p|rno-miR-29a-3p
NM_001126281	2	rno-miR-24-3p|rno-miR-6216
NM_001044263	1	rno-miR-24-3p
NM_001013040	1	rno-miR-24-3p
NM_001001517	1	rno-miR-24-3p

NM_031507	3	rno-miR-24-3p|rno-miR-27b-3p|rno-miR-27a-3p
NM_178097	1	rno-miR-24-3p
NM_144758	2	rno-miR-24-3p|rno-miR-199a-3p
NM_153470	4	rno-miR-24-3p|rno-miR-125b-5p|rno-miR-125a-5p|rno-miR-181a-5p
NM_001271027	2	rno-miR-24-3p|rno-miR-30c-5p
NM_138840	5	rno-miR-24-3p|rno-miR-125b-5p|rno-miR-125a-5p|rno-miR-17-5p|rno-miR-93-5p
NM_031544	5	rno-miR-24-3p|rno-miR-27b-3p|rno-miR-27a-3p|rno-miR-17-5p|rno-miR-93-5p
NM_001109122	1	rno-miR-24-3p
NM_001013856	1	rno-miR-24-3p
NM_001014168	1	rno-miR-24-3p
NM_001035001	1	rno-miR-24-3p
NM_031607	1	rno-miR-24-3p
NM_012598	1	rno-miR-24-3p
NM_001105874	4	rno-miR-24-3p|rno-miR-16-5p|rno-miR-195-5p|rno-miR-15b-5p
NM_001106563	5	rno-miR-24-3p|rno-miR-125b-5p|rno-miR-125a-5p|rno-miR-27b-3p|rno-miR-27a-3p
NM_001164505	1	rno-miR-24-3p
NM_001108013	3	rno-miR-24-3p|rno-miR-27b-3p|rno-miR-27a-3p
NM_001271167	3	rno-miR-24-3p|rno-miR-17-5p|rno-miR-93-5p
NM_001190237	2	rno-miR-24-3p|rno-miR-181a-5p
NM_001109300	1	rno-miR-24-3p
NM_001106866	1	rno-miR-24-3p
NM_053588	4	rno-miR-24-3p|rno-miR-16-5p|rno-miR-195-5p|rno-miR-15b-5p
NM_001170566	1	rno-miR-24-3p
NM_001164308	1	rno-miR-24-3p
NM_031234	1	rno-miR-24-3p
NM_001107752	1	rno-miR-24-3p

NM_001271247	8	rno-miR-24-3p|rno-miR-16-5p|rno-miR-125b-5p|rno-miR-125a-5p|rno-miR-27b-3p|rno-miR-195-5p|rno-miR-27a-3p|rno-miR-15b-5p
NM_019322	1	rno-miR-24-3p
NM_001108872	4	rno-miR-24-3p|rno-miR-103-3p|rno-miR-107-3p|rno-miR-31a-5p
NM_001109052	1	rno-miR-24-3p
NM_001024902	1	rno-miR-24-3p
NM_001110860	8	rno-miR-24-3p|rno-miR-16-5p|rno-miR-125b-5p|rno-miR-125a-5p|rno-miR-27b-3p|rno-miR-195-5p|rno-miR-27a-3p|rno-miR-15b-5p
NM_001127547	2	rno-miR-24-3p|rno-miR-378a-3p
NM_001134708	2	rno-miR-24-3p|rno-miR-200b-3p
NM_001109507	5	rno-miR-24-3p|rno-miR-103-3p|rno-miR-107-3p|rno-miR-320-3p|rno-miR-200b-3p
NM_001006966	1	rno-miR-24-3p
NM_001106665	2	rno-miR-24-3p|rno-miR-181a-5p
NM_212528	1	rno-miR-24-3p
NM_001105719	4	rno-miR-24-3p|rno-miR-125b-5p|rno-miR-125a-5p|rno-miR-199a-3p
NM_001040155	2	rno-miR-24-3p|rno-miR-29a-3p
NM_001100748	1	rno-miR-24-3p
NM_001134978	1	rno-miR-24-3p
NM_001106077	2	rno-miR-24-3p|rno-miR-22-3p
NM_001105741	2	rno-miR-24-3p|rno-miR-31a-5p
NM_001025059	3	rno-miR-24-3p|rno-miR-6216|rno-miR-200b-3p
NM_001025662	1	rno-miR-24-3p
NM_012785	1	rno-miR-24-3p
NM_019256	2	rno-miR-24-3p|rno-miR-151-5p
NM_001160313	8	rno-miR-24-3p|rno-miR-16-5p|rno-miR-125b-5p|rno-miR-125a-5p|rno-miR-27b-3p|rno-miR-195-5p|rno-miR-27a-3p|rno-miR-15b-5p
NM_001007702	3	rno-miR-24-3p|rno-miR-29a-3p|rno-miR-30c-5p
NM_017334	4	rno-miR-24-3p|rno-miR-16-5p|rno-miR-195-5p|rno-miR-15b-5p
NM_001164296	1	rno-miR-24-3p

NM_031077	3	rno-miR-24-3p|rno-miR-125b-5p|rno-miR-125a-5p
NM_017322	2	rno-miR-24-3p|rno-miR-320-3p
NM_031717	7	rno-miR-24-3p|rno-miR-103-3p|rno-miR-107-3p|rno-miR-27b-3p|rno-miR-27a-3p|rno-miR-200b-3p|rno-miR-30c-5p
NM_031757	6	rno-miR-24-3p|rno-miR-103-3p|rno-miR-107-3p|rno-miR-29a-3p|rno-miR-17-5p|rno-miR-93-5p
NM_001107427	1	rno-miR-24-3p
NM_012664	1	rno-miR-24-3p
NM_001107330	2	rno-miR-24-3p|rno-miR-30c-5p
NM_001013858	1	rno-miR-24-3p
NM_001015009	1	rno-miR-24-3p
NM_001169127	1	rno-miR-24-3p
NM_001013091	1	rno-miR-24-3p
NM_080576	1	rno-miR-24-3p
NM_001110099	2	rno-miR-24-3p|rno-miR-31a-5p
NM_001106316	1	rno-miR-24-3p
NM_001271245	4	rno-miR-24-3p|rno-miR-16-5p|rno-miR-195-5p|rno-miR-15b-5p
NM_001107008	3	rno-miR-24-3p|rno-miR-27b-3p|rno-miR-27a-3p
NM_017006	3	rno-miR-24-3p|rno-miR-103-3p|rno-miR-107-3p
NM_023963	2	rno-miR-24-3p|rno-miR-22-3p
NM_199091	1	rno-miR-24-3p
NM_001137641	1	rno-miR-24-3p
NM_031774	1	rno-miR-24-3p
NM_001017483	4	rno-miR-24-3p|rno-miR-16-5p|rno-miR-195-5p|rno-miR-15b-5p
NM_173143	3	rno-miR-24-3p|rno-miR-17-5p|rno-miR-93-5p
NM_017001	1	rno-miR-24-3p
NM_001108297	2	rno-miR-24-3p|rno-miR-205
NM_019261	1	rno-miR-24-3p
NM_001107958	2	rno-miR-24-3p|rno-miR-6216
NM_031855	1	rno-miR-24-3p

NM_134384	1	rno-miR-24-3p
NM_053909	8	rno-miR-24-3p|rno-miR-16-5p|rno-miR-125b-5p|rno-miR-125a-5p|rno-miR-27b-3p|rno-miR-195-5p|rno-miR-27a-3p|rno-miR-15b-5p
NM_001164298	1	rno-miR-24-3p
NM_001100551	1	rno-miR-24-3p
NM_001134481	12	rno-miR-24-3p|rno-miR-16-5p|rno-miR-125b-5p|rno-miR-103-3p|rno-miR-125a-5p|rno-miR-107-3p|rno-miR-27b-3p|rno-miR-195-5p|rno-miR-27a-3p|rno-miR-17-5p|rno-miR-15b-5p|rno-miR-93-5p
NM_001105953	1	rno-miR-24-3p
NM_171988	6	rno-miR-24-3p|rno-miR-181a-5p|rno-miR-30c-5p|rno-miR-17-5p|rno-miR-152-3p|rno-miR-93-5p
NM_001100512	1	rno-miR-24-3p
NM_147209	1	rno-miR-24-3p
NM_001034109	1	rno-miR-24-3p
NM_001106173	2	rno-miR-24-3p|rno-miR-200b-3p
NM_212547	1	rno-miR-24-3p
NM_080786	3	rno-miR-24-3p|rno-miR-6216|rno-miR-205
NM_001170434	5	rno-miR-24-3p|rno-miR-16-5p|rno-miR-195-5p|rno-miR-181a-5p|rno-miR-15b-5p
NM_001271039	1	rno-miR-24-3p
NM_001271102	8	rno-miR-24-3p|rno-miR-16-5p|rno-miR-125b-5p|rno-miR-125a-5p|rno-miR-27b-3p|rno-miR-195-5p|rno-miR-27a-3p|rno-miR-15b-5p
NM_001109036	4	rno-miR-24-3p|rno-miR-16-5p|rno-miR-195-5p|rno-miR-15b-5p
NM_001160315	8	rno-miR-24-3p|rno-miR-16-5p|rno-miR-125b-5p|rno-miR-125a-5p|rno-miR-27b-3p|rno-miR-195-5p|rno-miR-27a-3p|rno-miR-15b-5p
NM_178102	2	rno-miR-24-3p|rno-miR-31a-5p
NM_001271394	1	rno-miR-24-3p
NM_001108779	1	rno-miR-24-3p
NM_001109260	1	rno-miR-24-3p
NM_001100493	1	rno-miR-24-3p

NM_001014015	4	rno-miR-24-3p|rno-miR-16-5p|rno-miR-195-5p|rno-miR-15b-5p
NM_001108846	1	rno-miR-24-3p
NM_001109120	2	rno-miR-24-3p|rno-miR-30c-5p
NM_001034932	2	rno-miR-24-3p|rno-miR-29a-3p
NM_001100840	2	rno-miR-24-3p|rno-miR-181a-5p
NM_001190238	2	rno-miR-24-3p|rno-miR-181a-5p
NM_053486	1	rno-miR-24-3p
NM_031604	1	rno-miR-24-3p
NM_031678	5	rno-miR-24-3p|rno-miR-103-3p|rno-miR-107-3p|rno-miR-181a-5p|rno-miR-30c-5p
NM_024380	1	rno-miR-24-3p
NM_001004132	1	rno-miR-24-3p
NM_001013907	1	rno-miR-24-3p
NM_001108976	3	rno-miR-24-3p|rno-miR-125b-5p|rno-miR-125a-5p
NM_001013231	2	rno-miR-24-3p|rno-miR-152-3p
NM_001127297	1	rno-miR-24-3p
NM_001107570	1	rno-miR-24-3p
NM_001024290	1	rno-miR-24-3p
NM_001107393	2	rno-miR-24-3p|rno-miR-181a-5p
NM_001271072	6	rno-miR-24-3p|rno-miR-16-5p|rno-miR-320-3p|rno-miR-22-3p|rno-miR-195-5p|rno-miR-15b-5p
NM_001164299	1	rno-miR-24-3p
NM_001271226	7	rno-miR-24-3p|rno-miR-16-5p|rno-miR-195-5p|rno-miR-378a-3p|rno-miR-17-5p|rno-miR-15b-5p|rno-miR-93-5p
NM_001107697	2	rno-miR-24-3p|rno-miR-320-3p
NM_001011994	2	rno-miR-24-3p|rno-miR-3473
NM_001270544	2	rno-miR-24-3p|rno-miR-320-3p
NM_080890	1	rno-miR-24-3p
NM_001105834	1	rno-miR-24-3p
NM_001107170	4	rno-miR-24-3p|rno-miR-6216|rno-miR-320-3p|rno-miR-378a-3p

NM_019231	3	rno-miR-24-3p|rno-miR-125b-5p|rno-miR-125a-5p
NM_001277236	5	rno-miR-24-3p|rno-miR-125b-5p|rno-miR-125a-5p|rno-miR-17-5p|rno-miR-93-5p
NM_173145	1	rno-miR-24-3p
NM_001007626	1	rno-miR-24-3p
NM_001025707	2	rno-miR-24-3p|rno-miR-29a-3p
NM_001105875	1	rno-miR-24-3p
NM_001109217	2	rno-miR-24-3p|rno-miR-6216
NM_019249	1	rno-miR-24-3p
NM_031028	1	rno-miR-24-3p
NM_053429	2	rno-miR-24-3p|rno-miR-99b-5p
NM_032063	6	rno-miR-24-3p|rno-miR-16-5p|rno-miR-103-3p|rno-miR-107-3p|rno-miR-195-5p|rno-miR-15b-5p
NM_057143	1	rno-miR-24-3p
NM_001107829	1	rno-miR-24-3p
NM_001107840	1	rno-miR-24-3p
NM_001024749	3	rno-miR-24-3p|rno-miR-6216|rno-miR-30c-5p
NM_001136141	1	rno-miR-24-3p
NM_001024287	1	rno-miR-24-3p
NM_182817	2	rno-miR-24-3p|rno-miR-378a-3p
NM_001107064	3	rno-miR-24-3p|rno-miR-103-3p|rno-miR-107-3p
NM_001012078	1	rno-miR-24-3p
NM_001164305	1	rno-miR-24-3p
NM_001108403	1	rno-miR-24-3p
NM_001105889	1	rno-miR-24-3p
NM_001037187	3	rno-miR-24-3p|rno-miR-6216|rno-miR-3473
NM_001129777	2	rno-miR-24-3p|rno-miR-152-3p
NM_021660	1	rno-miR-24-3p
NM_001029920	1	rno-miR-24-3p
NM_001108821	2	rno-miR-24-3p|rno-miR-205

NM_001037191	1	rno-miR-24-3p
NM_001106188	1	rno-miR-24-3p
NM_001177326	1	rno-miR-24-3p
NM_019287	1	rno-miR-24-3p
NM_022183	1	rno-miR-24-3p
NM_031728	1	rno-miR-24-3p
NM_001190236	2	rno-miR-24-3p|rno-miR-181a-5p
NM_001007744	3	rno-miR-24-3p|rno-miR-125b-5p|rno-miR-125a-5p
NM_001013214	2	rno-miR-24-3p|rno-miR-181a-5p
NM_001015027	6	rno-miR-24-3p|rno-miR-16-5p|rno-miR-195-5p|rno-miR-205|rno-miR-181a-5p|rno-miR-15b-5p
NM_001013170	4	rno-miR-24-3p|rno-miR-125b-5p|rno-miR-125a-5p|rno-miR-320-3p
NM_001108408	3	rno-miR-24-3p|rno-miR-17-5p|rno-miR-93-5p
NM_031704	1	rno-miR-24-3p
NM_001107279	3	rno-miR-24-3p|rno-miR-6216|rno-miR-30c-5p
NM_001277252	1	rno-miR-24-3p
NM_001105832	1	rno-miR-24-3p
NM_001129882	2	rno-miR-24-3p|rno-miR-30c-5p
NM_001109420	2	rno-miR-24-3p|rno-miR-205
NM_001007737	1	rno-miR-24-3p
NM_001106606	3	rno-miR-24-3p|rno-miR-103-3p|rno-miR-107-3p
NM_001108100	4	rno-miR-24-3p|rno-miR-6216|rno-miR-103-3p|rno-miR-107-3p
NM_001108383	4	rno-miR-24-3p|rno-miR-320-3p|rno-miR-205|rno-miR-30c-5p
NM_053498	2	rno-miR-24-3p|rno-miR-181a-5p
NM_001107124	6	rno-miR-24-3p|rno-miR-27b-3p|rno-miR-22-3p|rno-miR-27a-3p|rno-miR-181a-5p|rno-miR-152-3p
NM_031079	1	rno-miR-24-3p
NM_001037097	1	rno-miR-24-3p
NM_001024966	1	rno-miR-24-3p
NM_001270931	2	rno-miR-24-3p|rno-miR-152-3p

NM_001173557	1	rno-miR-24-3p
NM_001277250	1	rno-miR-24-3p
NM_138857	1	rno-miR-24-3p
NM_031760	3	rno-miR-24-3p|rno-miR-125b-5p|rno-miR-125a-5p
NM_001270604	1	rno-miR-24-3p
NM_053415	2	rno-miR-24-3p|rno-miR-152-3p
NM_001008360	3	rno-miR-24-3p|rno-miR-27b-3p|rno-miR-27a-3p
NM_001134619	1	rno-miR-24-3p
NM_019202	1	rno-miR-24-3p
NM_001127521	1	rno-miR-24-3p
NM_001271216	3	rno-miR-24-3p|rno-miR-181a-5p|rno-miR-30c-5p
NM_001108940	3	rno-miR-24-3p|rno-miR-27b-3p|rno-miR-27a-3p
NM_001024792	1	rno-miR-24-3p
NM_001024883	1	rno-miR-24-3p
NM_001164303	1	rno-miR-24-3p
NM_001013969	1	rno-miR-24-3p
NM_001107410	1	rno-miR-24-3p
NM_001107842	2	rno-miR-24-3p|rno-miR-181a-5p
NM_001105803	1	rno-miR-24-3p
NM_001105829	1	rno-miR-24-3p
NM_001034934	1	rno-miR-24-3p
NM_057130	2	rno-miR-24-3p|rno-miR-29a-3p
NM_021850	5	rno-miR-24-3p|rno-miR-16-5p|rno-miR-195-5p|rno-miR-378a-3p|rno-miR-15b-5p
NM_001127566	1	rno-miR-24-3p
NM_001100680	1	rno-miR-191a-5p
NM_001107013	4	rno-miR-191a-5p|rno-miR-27b-3p|rno-miR-27a-3p|rno-miR-152-3p
NM_001301720	1	rno-miR-191a-5p
NM_001301715	1	rno-miR-191a-5p
NM_001109534	1	rno-miR-191a-5p

NM_022277	3	rno-miR-191a-5p|rno-miR-17-5p|rno-miR-93-5p
NM_001014140	1	rno-miR-191a-5p
NM_001108064	1	rno-miR-191a-5p
NM_199100	1	rno-miR-191a-5p
NM_012760	1	rno-miR-191a-5p
NM_022184	6	rno-miR-191a-5p|rno-miR-16-5p|rno-miR-27b-3p|rno-miR-195-5p|rno-miR-27a-3p|rno-miR-15b-5p
NM_024125	1	rno-miR-191a-5p
NM_001008316	1	rno-miR-191a-5p
NM_001106266	3	rno-miR-191a-5p|rno-miR-103-3p|rno-miR-107-3p
NM_001108512	4	rno-miR-191a-5p|rno-miR-27b-3p|rno-miR-27a-3p|rno-miR-29a-3p
NM_017035	1	rno-miR-191a-5p
NM_001025003	1	rno-miR-191a-5p
NM_001107528	1	rno-miR-191a-5p
NM_013019	1	rno-miR-191a-5p
NM_012508	9	rno-miR-191a-5p|rno-miR-16-5p|rno-miR-195-5p|rno-miR-205|rno-miR-181a-5p|rno-miR-30c-5p|rno-miR-17-5p|rno-miR-15b-5p|rno-miR-93-5p
NM_001107730	4	rno-miR-191a-5p|rno-miR-16-5p|rno-miR-195-5p|rno-miR-15b-5p
NM_001130061	1	rno-miR-191a-5p
NM_001100564	3	rno-miR-191a-5p|rno-miR-6216|rno-miR-205
NM_001017449	1	rno-miR-191a-5p
NM_001163277	3	rno-miR-191a-5p|rno-miR-200b-3p|rno-miR-31a-5p
NM_031724	1	rno-miR-191a-5p
NM_001007656	3	rno-miR-191a-5p|rno-miR-17-5p|rno-miR-93-5p
NM_013055	4	rno-miR-191a-5p|rno-miR-30c-5p|rno-miR-17-5p|rno-miR-93-5p
NM_001127574	1	rno-miR-191a-5p
NM_001024757	2	rno-miR-191a-5p|rno-miR-6216
NM_001107289	1	rno-miR-191a-5p
NM_001106494	1	rno-miR-191a-5p

NM_001109514	1	rno-miR-191a-5p
NM_019348	1	rno-miR-191a-5p
NM_001008863	1	rno-miR-191a-5p
NM_001014771	1	rno-miR-191a-5p
NM_001304747	1	rno-miR-191a-5p
NM_001257345	4	rno-miR-191a-5p|rno-miR-320-3p|rno-miR-199a-3p|rno-miR-200b-3p
NM_001173472	1	rno-miR-191a-5p
NM_031740	7	rno-miR-191a-5p|rno-miR-125b-5p|rno-miR-125a-5p|rno-miR-205|rno-miR-31a-5p|rno-miR-30c-5p|rno-miR-152-3p
NM_001107415	8	rno-miR-191a-5p|rno-miR-16-5p|rno-miR-103-3p|rno-miR-107-3p|rno-miR-195-5p|rno-miR-17-5p|rno-miR-15b-5p|rno-miR-93-5p
NM_203336	7	rno-miR-191a-5p|rno-miR-125b-5p|rno-miR-125a-5p|rno-miR-320-3p|rno-miR-205|rno-miR-200b-3p|rno-miR-152-3p
NM_001025764	1	rno-miR-191a-5p
NM_001025013	3	rno-miR-191a-5p|rno-miR-181a-5p|rno-miR-30c-5p
NM_001127545	5	rno-miR-191a-5p|rno-miR-103-3p|rno-miR-107-3p|rno-miR-27b-3p|rno-miR-27a-3p
NM_001012350	1	rno-miR-191a-5p
NM_001135612	3	rno-miR-191a-5p|rno-miR-17-5p|rno-miR-93-5p
NM_001100579	1	rno-miR-6216
NM_133440	4	rno-miR-6216|rno-miR-320-3p|rno-miR-17-5p|rno-miR-93-5p
NM_001107001	13	rno-miR-6216|rno-miR-16-5p|rno-miR-103-3p|rno-miR-107-3p|rno-miR-320-3p|rno-miR-27b-3p|rno-miR-195-5p|rno-miR-27a-3p|rno-miR-29a-3p|rno-miR-30c-5p|rno-miR-17-5p|rno-miR-15b-5p|rno-miR-93-5p
NM_001109439	3	rno-miR-6216|rno-miR-16-5p|rno-miR-195-5p
NM_001134543	8	rno-miR-6216|rno-miR-16-5p|rno-miR-103-3p|rno-miR-107-3p|rno-miR-320-3p|rno-miR-195-5p|rno-miR-181a-5p|rno-miR-15b-5p
NM_001164073	1	rno-miR-6216
NM_001106538	1	rno-miR-6216
NM_138827	3	rno-miR-6216|rno-miR-378a-3p|rno-miR-152-3p

NM_001107841	1	rno-miR-6216
NM_001108937	2	rno-miR-6216|rno-miR-200b-3p
NM_001107753	3	rno-miR-6216|rno-miR-103-3p|rno-miR-107-3p
NM_001004257	1	rno-miR-6216
NM_001107664	2	rno-miR-6216|rno-miR-181a-5p
NM_020097	1	rno-miR-6216
NM_001108503	2	rno-miR-6216|rno-miR-30c-5p
NM_001130695	4	rno-miR-6216|rno-miR-125b-5p|rno-miR-125a-5p|rno-miR-320-3p
NM_057104	3	rno-miR-6216|rno-miR-103-3p|rno-miR-107-3p
NM_001128078	1	rno-miR-6216
NM_001108465	4	rno-miR-6216|rno-miR-205|rno-miR-17-5p|rno-miR-93-5p
NM_024379	3	rno-miR-6216|rno-miR-320-3p|rno-miR-152-3p
NM_012845	1	rno-miR-6216
NM_172327	1	rno-miR-6216
NM_175603	1	rno-miR-6216
NM_001271092	1	rno-miR-6216
NM_001191569	2	rno-miR-6216|rno-miR-30c-5p
NM_017127	5	rno-miR-6216|rno-miR-199a-3p|rno-miR-27b-3p|rno-miR-27a-3p|rno-miR-30c-5p
NM_053637	4	rno-miR-6216|rno-miR-16-5p|rno-miR-195-5p|rno-miR-15b-5p
NM_001271502	2	rno-miR-6216|rno-miR-30c-5p
NM_001191723	1	rno-miR-6216
NM_001134850	6	rno-miR-6216|rno-miR-16-5p|rno-miR-27b-3p|rno-miR-195-5p|rno-miR-27a-3p|rno-miR-15b-5p
NM_030858	4	rno-miR-6216|rno-miR-16-5p|rno-miR-195-5p|rno-miR-15b-5p
NM_001107584	2	rno-miR-6216|rno-miR-199a-3p
NM_001107694	7	rno-miR-6216|rno-miR-16-5p|rno-miR-125b-5p|rno-miR-125a-5p|rno-miR-195-5p|rno-miR-181a-5p|rno-miR-15b-5p
NM_001169137	6	rno-miR-6216|rno-miR-199a-3p|rno-miR-200b-3p|rno-miR-181a-5p|rno-miR-17-5p|rno-miR-93-5p

NM_022247	2	rno-miR-6216|rno-miR-30c-5p
NM_001109440	2	rno-miR-6216|rno-miR-31a-5p
NM_153729	1	rno-miR-6216
NM_001040176	1	rno-miR-6216
NM_001107705	1	rno-miR-6216
NM_013087	1	rno-miR-6216
NM_001115046	1	rno-miR-6216
NM_001009679	1	rno-miR-6216
NM_001271238	1	rno-miR-6216
NM_080904	5	rno-miR-6216|rno-miR-27b-3p|rno-miR-27a-3p|rno-miR-181a-5p|rno-miR-152-3p
NM_134374	2	rno-miR-6216|rno-miR-29a-3p
NM_001107181	1	rno-miR-6216
NM_199502	3	rno-miR-6216|rno-miR-200b-3p|rno-miR-181a-5p
NM_001039342	1	rno-miR-6216
NM_001106758	1	rno-miR-6216
NM_001109224	3	rno-miR-6216|rno-miR-103-3p|rno-miR-107-3p
NM_001102383	3	rno-miR-6216|rno-miR-27b-3p|rno-miR-27a-3p
NM_001007728	1	rno-miR-6216
NM_001170606	1	rno-miR-6216
NM_080910	1	rno-miR-6216
NM_012990	3	rno-miR-6216|rno-miR-200b-3p|rno-miR-152-3p
NM_001108798	7	rno-miR-6216|rno-miR-103-3p|rno-miR-107-3p|rno-miR-27b-3p|rno-miR-27a-3p|rno-miR-17-5p|rno-miR-93-5p
NM_001127485	1	rno-miR-6216
NM_001107277	1	rno-miR-6216
NM_012647	2	rno-miR-6216|rno-miR-30c-5p
NM_001007740	1	rno-miR-6216
NM_001270775	1	rno-miR-6216
NM_001107268	4	rno-miR-6216|rno-miR-320-3p|rno-miR-30c-5p|rno-miR-152-3p

NM_024385	1	rno-miR-6216
NM_001106370	2	rno-miR-6216|rno-miR-22-3p
NM_001014035	1	rno-miR-6216
NM_001034154	1	rno-miR-6216
NM_139060	3	rno-miR-6216|rno-miR-27b-3p|rno-miR-27a-3p
NM_030846	4	rno-miR-6216|rno-miR-27b-3p|rno-miR-378a-3p|rno-miR-27a-3p
NM_001271195	1	rno-miR-6216
NM_001191842	1	rno-miR-6216
NM_031321	2	rno-miR-6216|rno-miR-205
NM_053947	5	rno-miR-6216|rno-miR-125b-5p|rno-miR-125a-5p|rno-miR-27b-3p|rno-miR-27a-3p
NM_017238	1	rno-miR-6216
NM_001098216	1	rno-miR-6216
NM_001015015	1	rno-miR-6216
NM_212515	1	rno-miR-6216
NM_001037292	1	rno-miR-6216
NM_001107506	3	rno-miR-6216|rno-miR-17-5p|rno-miR-93-5p
NM_001109107	3	rno-miR-6216|rno-miR-17-5p|rno-miR-93-5p
NM_001106448	1	rno-miR-6216
NM_001271094	1	rno-miR-6216
NM_001271207	2	rno-miR-6216|rno-miR-181a-5p
NM_001106800	3	rno-miR-6216|rno-miR-27b-3p|rno-miR-27a-3p
NM_001108352	1	rno-miR-6216
NM_001105751	2	rno-miR-6216|rno-miR-152-3p
NM_001270963	1	rno-miR-6216
NM_013093	1	rno-miR-6216
NM_001110141	2	rno-miR-6216|rno-miR-200b-3p
NM_001107689	7	rno-miR-6216|rno-miR-16-5p|rno-miR-103-3p|rno-miR-107-3p|rno-miR-195-5p|rno-miR-31a-5p|rno-miR-15b-5p
NM_001271038	1	rno-miR-6216

NM_001127578	3	rno-miR-6216|rno-miR-27b-3p|rno-miR-27a-3p
NM_001107313	8	rno-miR-6216|rno-miR-320-3p|rno-miR-199a-3p|rno-miR-27b-3p|rno-miR-27a-3p|rno-miR-200b-3p|rno-miR-17-5p|rno-miR-93-5p
NM_053458	1	rno-miR-6216
NM_138854	1	rno-miR-6216
NM_021751	1	rno-miR-6216
NM_001025409	1	rno-miR-6216
NM_001109319	2	rno-miR-6216|rno-miR-320-3p
NM_022538	4	rno-miR-6216|rno-miR-16-5p|rno-miR-195-5p|rno-miR-15b-5p
NM_001108742	2	rno-miR-6216|rno-miR-30c-5p
NM_133603	2	rno-miR-6216|rno-miR-181a-5p
NM_131909	1	rno-miR-6216
NM_001134714	1	rno-miR-6216
NM_053633	4	rno-miR-6216|rno-miR-3473|rno-miR-17-5p|rno-miR-93-5p
NM_001106898	2	rno-miR-6216|rno-miR-181a-5p
NM_001169139	1	rno-miR-6216
NM_053655	1	rno-miR-6216
NM_019337	1	rno-miR-6216
NM_001015026	6	rno-miR-6216|rno-miR-16-5p|rno-miR-125b-5p|rno-miR-125a-5p|rno-miR-195-5p|rno-miR-15b-5p
NM_001009624	1	rno-miR-6216
NM_001106150	6	rno-miR-6216|rno-miR-16-5p|rno-miR-195-5p|rno-miR-181a-5p|rno-miR-31a-5p|rno-miR-15b-5p
NM_001100528	2	rno-miR-6216|rno-miR-30c-5p
NM_001106820	1	rno-miR-6216
NM_001014222	1	rno-miR-6216
NM_001100756	3	rno-miR-6216|rno-miR-125b-5p|rno-miR-125a-5p
NM_001033688	3	rno-miR-6216|rno-miR-30c-5p|rno-miR-152-3p
NM_001108028	5	rno-miR-6216|rno-miR-27b-3p|rno-miR-27a-3p|rno-miR-17-5p|rno-miR-93-5p

NM_001271196	4	rno-miR-6216|rno-miR-27b-3p|rno-miR-27a-3p|rno-miR-29a-3p
NM_017203	4	rno-miR-6216|rno-miR-3473|rno-miR-17-5p|rno-miR-93-5p
NM_001270779	1	rno-miR-6216
NM_001009493	1	rno-miR-6216
NM_001305138	3	rno-miR-6216|rno-miR-17-5p|rno-miR-93-5p
NM_001008517	3	rno-miR-6216|rno-miR-199a-3p|rno-miR-200b-3p
NM_001126087	3	rno-miR-6216|rno-miR-29a-3p|rno-miR-30c-5p
NM_172072	1	rno-miR-6216
NM_001134856	4	rno-miR-6216|rno-miR-27b-3p|rno-miR-27a-3p|rno-miR-152-3p
NM_001013956	1	rno-miR-6216
NM_053521	5	rno-miR-6216|rno-miR-320-3p|rno-miR-27b-3p|rno-miR-27a-3p|rno-miR-181a-5p
NM_001127690	1	rno-miR-6216
NM_001025756	1	rno-miR-6216
NM_001108960	1	rno-miR-6216
NM_001025641	1	rno-miR-6216
NM_001287134	6	rno-miR-6216|rno-miR-125b-5p|rno-miR-125a-5p|rno-miR-200b-3p|rno-miR-17-5p|rno-miR-93-5p
NM_022280	3	rno-miR-6216|rno-miR-200b-3p|rno-miR-152-3p
NM_001173528	3	rno-miR-6216|rno-miR-200b-3p|rno-miR-30c-5p
NM_206845	7	rno-miR-6216|rno-miR-3473|rno-miR-27b-3p|rno-miR-22-3p|rno-miR-27a-3p|rno-miR-17-5p|rno-miR-93-5p
NM_001191606	1	rno-miR-6216
NM_017104	1	rno-miR-6216
NM_001107713	3	rno-miR-6216|rno-miR-125b-5p|rno-miR-125a-5p
NM_001270774	1	rno-miR-6216
NM_001025740	3	rno-miR-6216|rno-miR-17-5p|rno-miR-93-5p
NM_013043	3	rno-miR-6216|rno-miR-205|rno-miR-200b-3p
NM_001127539	1	rno-miR-6216
NM_133610	1	rno-miR-6216

NM_001007673	7	rno-miR-6216|rno-miR-27b-3p|rno-miR-205|rno-miR-27a-3p|rno-miR-200b-3p|rno-miR-30c-5p|rno-miR-152-3p
NM_138891	2	rno-miR-6216|rno-miR-30c-5p
NM_001109005	2	rno-miR-6216|rno-miR-205
NM_001163164	1	rno-miR-6216
NM_001109480	7	rno-miR-6216|rno-miR-16-5p|rno-miR-27b-3p|rno-miR-195-5p|rno-miR-27a-3p|rno-miR-30c-5p|rno-miR-15b-5p
NM_001106189	1	rno-miR-6216
NM_001005905	1	rno-miR-6216
NM_001106932	4	rno-miR-6216|rno-miR-320-3p|rno-miR-17-5p|rno-miR-93-5p
NM_001024310	2	rno-miR-6216|rno-miR-30c-5p
NM_022669	1	rno-miR-6216
NM_001107214	5	rno-miR-6216|rno-miR-103-3p|rno-miR-107-3p|rno-miR-17-5p|rno-miR-93-5p
NM_001109411	1	rno-miR-6216
NM_012654	4	rno-miR-6216|rno-miR-103-3p|rno-miR-107-3p|rno-miR-181a-5p
NM_001100636	3	rno-miR-6216|rno-miR-200b-3p|rno-miR-30c-5p
NM_001007630	3	rno-miR-6216|rno-miR-200b-3p|rno-miR-181a-5p
NM_207602	4	rno-miR-6216|rno-miR-27b-3p|rno-miR-27a-3p|rno-miR-200b-3p
NM_024143	1	rno-miR-6216
NM_001108490	3	rno-miR-6216|rno-miR-29a-3p|rno-miR-181a-5p
NM_001031663	1	rno-miR-6216
NM_001042505	2	rno-miR-6216|rno-miR-181a-5p
NM_001106042	7	rno-miR-6216|rno-miR-16-5p|rno-miR-320-3p|rno-miR-195-5p|rno-miR-205|rno-miR-152-3p|rno-miR-15b-5p
NM_001134532	2	rno-miR-6216|rno-miR-152-3p
NM_012702	1	rno-miR-6216
NM_053625	1	rno-miR-6216
NM_017316	2	rno-miR-6216|rno-miR-200b-3p
NM_001031659	2	rno-miR-6216|rno-miR-30c-5p

NM_053662	2	rno-miR-6216|rno-miR-3473
NM_001108291	1	rno-miR-6216
NM_001025635	1	rno-miR-6216
NM_001108176	1	rno-miR-6216
NM_138866	4	rno-miR-6216|rno-miR-125b-5p|rno-miR-125a-5p|rno-miR-200b-3p
NM_024355	7	rno-miR-6216|rno-miR-16-5p|rno-miR-103-3p|rno-miR-107-3p|rno-miR-195-5p|rno-miR-205|rno-miR-15b-5p
NM_001107849	2	rno-miR-6216|rno-miR-29a-3p
NM_172074	1	rno-miR-6216
NM_020542	1	rno-miR-6216
NM_001014211	2	rno-miR-6216|rno-miR-200b-3p
NM_133600	1	rno-miR-6216
NM_001106220	1	rno-miR-6216
NM_001109968	2	rno-miR-6216|rno-miR-181a-5p
NM_021657	1	rno-miR-6216
NM_012576	5	rno-miR-6216|rno-miR-320-3p|rno-miR-200b-3p|rno-miR-181a-5p|rno-miR-30c-5p
NM_001039002	2	rno-miR-6216|rno-miR-30c-5p
NM_001011998	5	rno-miR-6216|rno-miR-103-3p|rno-miR-107-3p|rno-miR-27b-3p|rno-miR-27a-3p
NM_001105979	3	rno-miR-6216|rno-miR-125b-5p|rno-miR-125a-5p
NM_001107634	5	rno-miR-6216|rno-miR-125b-5p|rno-miR-125a-5p|rno-miR-205|rno-miR-152-3p
NM_012784	7	rno-miR-6216|rno-miR-199a-3p|rno-miR-27b-3p|rno-miR-27a-3p|rno-miR-29a-3p|rno-miR-181a-5p|rno-miR-152-3p
NM_001135084	1	rno-miR-6216
NM_001108216	2	rno-miR-6216|rno-miR-200b-3p
NM_001271091	1	rno-miR-6216

NM_017269	9	rno-miR-6216|rno-miR-16-5p|rno-miR-27b-3p|rno-miR-195-5p|rno-miR-205|rno-miR-27a-3p|rno-miR-17-5p|rno-miR-15b-5p|rno-miR-93-5p
NM_001109912	3	rno-miR-6216|rno-miR-205|rno-miR-200b-3p
NM_001100563	1	rno-miR-6216
NM_001270772	1	rno-miR-6216
NM_001270777	1	rno-miR-6216
NM_030860	1	rno-miR-6216
NM_022218	3	rno-miR-6216|rno-miR-103-3p|rno-miR-107-3p
NM_001108539	3	rno-miR-6216|rno-miR-27b-3p|rno-miR-27a-3p
NM_001107462	1	rno-miR-6216
NM_001134549	2	rno-miR-6216|rno-miR-152-3p
NM_001037181	1	rno-miR-6216
NM_001100986	2	rno-miR-6216|rno-miR-200b-3p
NM_001107254	2	rno-miR-6216|rno-miR-30c-5p
NM_001142949	1	rno-miR-6216
NM_021843	2	rno-miR-6216|rno-miR-320-3p
NM_019197	2	rno-miR-6216|rno-miR-199a-3p
NM_001108658	3	rno-miR-6216|rno-miR-17-5p|rno-miR-93-5p
NM_001108508	1	rno-miR-6216
NM_001079894	2	rno-miR-6216|rno-miR-31a-5p
NM_001105723	1	rno-miR-6216
NM_001109306	4	rno-miR-6216|rno-miR-27b-3p|rno-miR-205|rno-miR-27a-3p
NM_001009466	1	rno-miR-6216
NM_182821	3	rno-miR-6216|rno-miR-17-5p|rno-miR-93-5p
NM_053785	1	rno-miR-6216
NM_001106718	2	rno-miR-6216|rno-miR-30c-5p
NM_012804	3	rno-miR-6216|rno-miR-195-5p|rno-miR-152-3p
NM_001106984	1	rno-miR-6216
NM_001128079	3	rno-miR-6216|rno-miR-125b-5p|rno-miR-125a-5p

NM_024139	1	rno-miR-6216
NM_001108750	5	rno-miR-6216|rno-miR-27b-3p|rno-miR-27a-3p|rno-miR-29a-3p|rno-miR-30c-5p
NM_001107984	5	rno-miR-6216|rno-miR-22-3p|rno-miR-205|rno-miR-29a-3p|rno-miR-200b-3p
NM_001106204	4	rno-miR-6216|rno-miR-16-5p|rno-miR-195-5p|rno-miR-15b-5p
NM_013144	3	rno-miR-6216|rno-miR-103-3p|rno-miR-107-3p
NM_001108103	3	rno-miR-6216|rno-miR-181a-5p|rno-miR-30c-5p
NM_001007092	1	rno-miR-6216
NM_001115027	2	rno-miR-6216|rno-miR-205
NM_001005878	1	rno-miR-6216
NM_001270778	1	rno-miR-6216
NM_001101805	4	rno-miR-6216|rno-miR-27b-3p|rno-miR-27a-3p|rno-miR-200b-3p
NM_001127379	3	rno-miR-6216|rno-miR-199a-3p|rno-miR-181a-5p
NM_001106023	5	rno-miR-6216|rno-miR-27b-3p|rno-miR-27a-3p|rno-miR-17-5p|rno-miR-93-5p
NM_001106733	1	rno-miR-6216
NM_001011894	3	rno-miR-6216|rno-miR-103-3p|rno-miR-107-3p
NM_001106472	3	rno-miR-6216|rno-miR-27b-3p|rno-miR-27a-3p
NM_001109214	3	rno-miR-6216|rno-miR-27b-3p|rno-miR-27a-3p
NM_001108775	6	rno-miR-6216|rno-miR-125b-5p|rno-miR-125a-5p|rno-miR-29a-3p|rno-miR-181a-5p|rno-miR-31a-5p
NM_001017490	1	rno-miR-6216
NM_001044296	1	rno-miR-6216
NM_001107868	1	rno-miR-6216
NM_022262	1	rno-miR-6216
NM_001191775	1	rno-miR-6216
NM_001106985	2	rno-miR-6216|rno-miR-30c-5p
NM_001017498	1	rno-miR-6216
NM_001014114	1	rno-miR-6216

NM_001318118	1	rno-miR-6216
NM_001025044	1	rno-miR-6216
NM_001100529	3	rno-miR-6216|rno-miR-17-5p|rno-miR-93-5p
NM_053594	4	rno-miR-6216|rno-miR-16-5p|rno-miR-195-5p|rno-miR-15b-5p
NM_001106112	1	rno-miR-6216
NM_001106158	2	rno-miR-6216|rno-miR-181a-5p
NM_053476	7	rno-miR-6216|rno-miR-16-5p|rno-miR-103-3p|rno-miR-107-3p|rno-miR-195-5p|rno-miR-152-3p|rno-miR-15b-5p
NM_013057	3	rno-miR-6216|rno-miR-17-5p|rno-miR-93-5p
NM_031771	2	rno-miR-6216|rno-miR-320-3p
NM_001108318	2	rno-miR-6216|rno-miR-181a-5p
NM_001127523	1	rno-miR-6216
NM_001106937	1	rno-miR-6216
NM_001134589	1	rno-miR-6216
NM_001270982	1	rno-miR-6216
NM_031583	1	rno-miR-6216
NM_133441	4	rno-miR-6216|rno-miR-320-3p|rno-miR-17-5p|rno-miR-93-5p
NM_013082	4	rno-miR-6216|rno-miR-199a-3p|rno-miR-27b-3p|rno-miR-27a-3p
NM_001011943	1	rno-miR-6216
NM_001107174	4	rno-miR-6216|rno-miR-320-3p|rno-miR-27b-3p|rno-miR-27a-3p
NM_001037350	3	rno-miR-6216|rno-miR-125b-5p|rno-miR-125a-5p
NM_001106303	1	rno-miR-6216
NM_001024884	1	rno-miR-6216
NM_012560	3	rno-miR-6216|rno-miR-200b-3p|rno-miR-30c-5p
NM_001145273	1	rno-miR-6216
NM_001008317	1	rno-miR-6216
NM_001271292	2	rno-miR-6216|rno-miR-29a-3p
NM_001106864	1	rno-miR-6216
NM_001271093	1	rno-miR-6216
NM_001108295	1	rno-miR-6216

NM_012561	2	rno-miR-6216|rno-miR-30c-5p
NM_001100479	10	rno-miR-6216|rno-miR-16-5p|rno-miR-103-3p|rno-miR-107-3p|rno-miR-199a-3p|rno-miR-195-5p|rno-miR-181a-5p|rno-miR-17-5p|rno-miR-15b-5p|rno-miR-93-5p
NM_134463	3	rno-miR-6216|rno-miR-125b-5p|rno-miR-125a-5p
NM_053926	4	rno-miR-6216|rno-miR-30c-5p|rno-miR-17-5p|rno-miR-93-5p
NM_133421	1	rno-miR-6216
NM_001135845	1	rno-miR-6216
NM_001004102	1	rno-miR-6216
NM_017046	1	rno-miR-6216
NM_001270784	1	rno-miR-6216
NM_019219	1	rno-miR-6216
NM_001169138	1	rno-miR-6216
NM_001128192	1	rno-miR-6216
NM_031967	1	rno-miR-6216
NM_031642	6	rno-miR-6216|rno-miR-22-3p|rno-miR-181a-5p|rno-miR-17-5p|rno-miR-152-3p|rno-miR-93-5p
NM_053742	1	rno-miR-6216
NM_031693	1	rno-miR-6216
NM_001304355	5	rno-miR-6216|rno-miR-125b-5p|rno-miR-125a-5p|rno-miR-22-3p|rno-miR-205
NM_178105	4	rno-miR-6216|rno-miR-205|rno-miR-200b-3p|rno-miR-152-3p
NM_001106698	5	rno-miR-6216|rno-miR-16-5p|rno-miR-22-3p|rno-miR-195-5p|rno-miR-15b-5p
NM_012769	3	rno-miR-6216|rno-miR-17-5p|rno-miR-93-5p
NM_001191715	1	rno-miR-6216
NM_001109184	1	rno-miR-6216
NM_001039722	2	rno-miR-6216|rno-miR-181a-5p
NM_001108922	1	rno-miR-6216
NM_021844	2	rno-miR-6216|rno-miR-320-3p

NM_013221	4	rno-miR-6216|rno-miR-29a-3p|rno-miR-17-5p|rno-miR-93-5p
NM_001110137	1	rno-miR-6216
NM_001109900	1	rno-miR-6216
NM_001107519	1	rno-miR-6216
NM_001009470	1	rno-miR-6216
NM_001077667	1	rno-miR-6216
NM_001127551	1	rno-miR-6216
NM_001012197	2	rno-miR-6216|rno-miR-181a-5p
NM_001106963	1	rno-miR-6216
NM_001108684	2	rno-miR-6216|rno-miR-205
NM_001108666	2	rno-miR-6216|rno-miR-200b-3p
NM_001006960	1	rno-miR-6216
NM_001100985	1	rno-miR-6216
NM_022254	1	rno-miR-6216
NM_001270776	1	rno-miR-6216
NM_001107724	2	rno-miR-6216|rno-miR-200b-3p
NM_001109016	4	rno-miR-6216|rno-miR-16-5p|rno-miR-195-5p|rno-miR-15b-5p
NM_001100560	4	rno-miR-6216|rno-miR-27b-3p|rno-miR-27a-3p|rno-miR-30c-5p
NM_001170596	2	rno-miR-6216|rno-miR-181a-5p
NM_001107861	4	rno-miR-6216|rno-miR-16-5p|rno-miR-195-5p|rno-miR-15b-5p
NM_001011901	3	rno-miR-6216|rno-miR-320-3p|rno-miR-31a-5p
NM_012980	1	rno-miR-6216
NM_013053	6	rno-miR-6216|rno-miR-16-5p|rno-miR-27b-3p|rno-miR-195-5p|rno-miR-27a-3p|rno-miR-15b-5p
NM_001108434	2	rno-miR-6216|rno-miR-30c-5p
NM_001012044	1	rno-miR-6216
NM_022617	1	rno-miR-6216
NM_001109639	1	rno-miR-6216
NM_001024776	1	rno-miR-6216
NM_001100968	1	rno-miR-6216

NM_001271192	4	rno-miR-6216|rno-miR-16-5p|rno-miR-195-5p|rno-miR-15b-5p
NM_001108030	5	rno-miR-6216|rno-miR-125b-5p|rno-miR-125a-5p|rno-miR-27b-3p|rno-miR-27a-3p
NM_001270773	1	rno-miR-6216
NM_139190	1	rno-miR-6216
NM_031559	1	rno-miR-6216
NM_001271095	1	rno-miR-6216
NM_001191773	1	rno-miR-6216
NM_001109503	2	rno-miR-6216|rno-miR-29a-3p
NM_001106779	2	rno-miR-6216|rno-miR-200b-3p
NM_001033663	1	rno-miR-6216
NM_001037774	2	rno-miR-6216|rno-miR-205
NM_001100718	1	rno-miR-6216
NM_145788	1	rno-miR-6216
NM_001012156	1	rno-miR-6216
NM_021836	2	rno-miR-6216|rno-miR-30c-5p
NM_001107838	3	rno-miR-6216|rno-miR-378a-3p|rno-miR-205
NM_001109483	1	rno-miR-6216
NM_001108733	5	rno-miR-6216|rno-miR-27b-3p|rno-miR-27a-3p|rno-miR-29a-3p|rno-miR-200b-3p
NM_001109060	4	rno-miR-6216|rno-miR-125b-5p|rno-miR-125a-5p|rno-miR-152-3p
NM_001305205	1	rno-miR-6216
NM_053974	3	rno-miR-6216|rno-miR-205|rno-miR-200b-3p
NM_001270962	1	rno-miR-6216
NM_001106638	1	rno-miR-6216
NM_031138	2	rno-miR-6216|rno-miR-181a-5p
NM_147211	5	rno-miR-6216|rno-miR-320-3p|rno-miR-27b-3p|rno-miR-27a-3p|rno-miR-30c-5p
NM_001191915	1	rno-miR-6216
NM_001014233	4	rno-miR-6216|rno-miR-16-5p|rno-miR-195-5p|rno-miR-15b-5p

NM_017051	1	rno-miR-6216
NM_172008	4	rno-miR-6216|rno-miR-320-3p|rno-miR-30c-5p|rno-miR-152-3p
NM_138889	1	rno-miR-6216
NM_022387	1	rno-miR-6216
NM_019299	2	rno-miR-6216|rno-miR-205
NM_031699	2	rno-miR-6216|rno-miR-29a-3p
NM_012527	1	rno-miR-6216
NM_031762	2	rno-miR-6216|rno-miR-152-3p
NM_001047893	4	rno-miR-6216|rno-miR-27b-3p|rno-miR-27a-3p|rno-miR-29a-3p
NM_001305460	1	rno-miR-6216
NM_207610	1	rno-miR-6216
NM_001109232	1	rno-miR-6216
NM_012750	1	rno-miR-6216
NM_001047915	3	rno-miR-6216|rno-miR-125b-5p|rno-miR-125a-5p
NM_001134513	1	rno-miR-6216
NM_001271495	3	rno-miR-6216|rno-miR-29a-3p|rno-miR-30c-5p
NM_001029923	3	rno-miR-6216|rno-miR-27b-3p|rno-miR-27a-3p
NM_001004255	6	rno-miR-6216|rno-miR-16-5p|rno-miR-195-5p|rno-miR-200b-3p|rno-miR-181a-5p|rno-miR-15b-5p
NM_001113390	4	rno-miR-6216|rno-miR-16-5p|rno-miR-195-5p|rno-miR-15b-5p
NM_001033852	1	rno-miR-6216
NM_001106691	4	rno-miR-6216|rno-miR-16-5p|rno-miR-195-5p|rno-miR-15b-5p
NM_019268	1	rno-miR-6216
NM_019142	2	rno-miR-6216|rno-miR-152-3p
NM_001107160	4	rno-miR-6216|rno-miR-16-5p|rno-miR-195-5p|rno-miR-15b-5p
NM_001107901	4	rno-miR-6216|rno-miR-29a-3p|rno-miR-17-5p|rno-miR-93-5p
NM_017065	1	rno-miR-6216
NM_001105768	2	rno-miR-6216|rno-miR-30c-5p
NM_001114606	1	rno-miR-6216
NM_001305274	4	rno-miR-6216|rno-miR-27b-3p|rno-miR-27a-3p|rno-miR-30c-5p

NM_017229	8	rno-miR-6216|rno-miR-103-3p|rno-miR-107-3p|rno-miR-27b-3p|rno-miR-205|rno-miR-27a-3p|rno-miR-17-5p|rno-miR-93-5p
NM_001271410	5	rno-miR-6216|rno-miR-27b-3p|rno-miR-27a-3p|rno-miR-200b-3p|rno-miR-30c-5p
NM_001100548	1	rno-miR-6216
NM_019280	1	rno-miR-6216
NM_001085353	3	rno-miR-6216|rno-miR-320-3p|rno-miR-181a-5p
NM_021869	1	rno-miR-6216
NM_020077	1	rno-miR-6216
NM_001108594	1	rno-miR-6216
NM_022503	1	rno-miR-6216
NM_001108547	1	rno-miR-6216
NM_001039454	1	rno-miR-6216
NM_001109012	3	rno-miR-6216|rno-miR-17-5p|rno-miR-93-5p
NM_138884	1	rno-miR-6216
NM_001305139	3	rno-miR-6216|rno-miR-17-5p|rno-miR-93-5p
NM_001100898	2	rno-miR-6216|rno-miR-199a-3p
NM_001100541	8	rno-miR-6216|rno-miR-103-3p|rno-miR-107-3p|rno-miR-199a-3p|rno-miR-27b-3p|rno-miR-27a-3p|rno-miR-181a-5p|rno-miR-152-3p
NM_001106226	2	rno-miR-6216|rno-miR-31a-5p
NM_001107995	2	rno-miR-6216|rno-miR-30c-5p
NM_001271106	1	rno-miR-6216
NM_001037093	1	rno-miR-6216
NM_001107084	4	rno-miR-6216|rno-miR-200b-3p|rno-miR-181a-5p|rno-miR-30c-5p
NM_173330	3	rno-miR-6216|rno-miR-17-5p|rno-miR-93-5p
NM_024388	1	rno-miR-6216
NM_019334	2	rno-miR-6216|rno-miR-181a-5p
NM_021763	2	rno-miR-6216|rno-miR-152-3p
NM_001106635	3	rno-miR-6216|rno-miR-31a-5p|rno-miR-30c-5p
NM_001108144	1	rno-miR-6216

NM_001191807	5	rno-miR-6216|rno-miR-199a-3p|rno-miR-27b-3p|rno-miR-27a-3p|rno-miR-200b-3p
NM_001012082	3	rno-miR-6216|rno-miR-27b-3p|rno-miR-27a-3p
NM_001107863	4	rno-miR-6216|rno-miR-16-5p|rno-miR-195-5p|rno-miR-15b-5p
NM_001033694	1	rno-miR-6216
NM_001107944	2	rno-miR-6216|rno-miR-200b-3p
NM_001024993	3	rno-miR-6216|rno-miR-103-3p|rno-miR-107-3p
NM_001011999	2	rno-miR-6216|rno-miR-29a-3p
NM_001100716	7	rno-miR-6216|rno-miR-27b-3p|rno-miR-27a-3p|rno-miR-30c-5p|rno-miR-17-5p|rno-miR-152-3p|rno-miR-93-5p
NM_001106687	3	rno-miR-16-5p|rno-miR-195-5p|rno-miR-15b-5p
NM_001014005	3	rno-miR-16-5p|rno-miR-195-5p|rno-miR-15b-5p
NM_001100707	3	rno-miR-16-5p|rno-miR-195-5p|rno-miR-15b-5p
NM_001108957	3	rno-miR-16-5p|rno-miR-195-5p|rno-miR-15b-5p
NM_001007662	4	rno-miR-16-5p|rno-miR-195-5p|rno-miR-200b-3p|rno-miR-15b-5p
NM_019167	3	rno-miR-16-5p|rno-miR-195-5p|rno-miR-15b-5p
NM_024357	5	rno-miR-16-5p|rno-miR-103-3p|rno-miR-107-3p|rno-miR-195-5p|rno-miR-15b-5p
NM_145877	3	rno-miR-16-5p|rno-miR-195-5p|rno-miR-15b-5p
NM_001108155	7	rno-miR-16-5p|rno-miR-103-3p|rno-miR-107-3p|rno-miR-195-5p|rno-miR-17-5p|rno-miR-15b-5p|rno-miR-93-5p
NM_001014027	3	rno-miR-16-5p|rno-miR-195-5p|rno-miR-15b-5p
NM_001106273	3	rno-miR-16-5p|rno-miR-195-5p|rno-miR-15b-5p
NM_201991	3	rno-miR-16-5p|rno-miR-195-5p|rno-miR-15b-5p
NM_031763	5	rno-miR-16-5p|rno-miR-125b-5p|rno-miR-125a-5p|rno-miR-195-5p|rno-miR-15b-5p
NM_057190	3	rno-miR-16-5p|rno-miR-195-5p|rno-miR-15b-5p
NM_001134467	6	rno-miR-16-5p|rno-miR-125b-5p|rno-miR-125a-5p|rno-miR-195-5p|rno-miR-29a-3p|rno-miR-15b-5p

NM_001008324	5	rno-miR-16-5p|rno-miR-103-3p|rno-miR-107-3p|rno-miR-195-5p|rno-miR-15b-5p
NM_001013952	3	rno-miR-16-5p|rno-miR-195-5p|rno-miR-15b-5p
NM_012570	7	rno-miR-16-5p|rno-miR-103-3p|rno-miR-107-3p|rno-miR-195-5p|rno-miR-378a-3p|rno-miR-30c-5p|rno-miR-15b-5p
NM_001305229	7	rno-miR-16-5p|rno-miR-125b-5p|rno-miR-103-3p|rno-miR-125a-5p|rno-miR-107-3p|rno-miR-195-5p|rno-miR-15b-5p
NM_080902	4	rno-miR-16-5p|rno-miR-195-5p|rno-miR-152-3p|rno-miR-15b-5p
NM_133591	4	rno-miR-16-5p|rno-miR-195-5p|rno-miR-152-3p|rno-miR-15b-5p
NM_001024799	5	rno-miR-16-5p|rno-miR-27b-3p|rno-miR-195-5p|rno-miR-27a-3p|rno-miR-15b-5p
NM_001107870	4	rno-miR-16-5p|rno-miR-195-5p|rno-miR-31a-5p|rno-miR-15b-5p
NM_001107908	4	rno-miR-16-5p|rno-miR-195-5p|rno-miR-205|rno-miR-15b-5p
NM_001130578	3	rno-miR-16-5p|rno-miR-195-5p|rno-miR-15b-5p
NM_033349	3	rno-miR-16-5p|rno-miR-195-5p|rno-miR-15b-5p
NM_001100821	3	rno-miR-16-5p|rno-miR-195-5p|rno-miR-15b-5p
NM_001012128	3	rno-miR-16-5p|rno-miR-195-5p|rno-miR-15b-5p
NM_001100572	5	rno-miR-16-5p|rno-miR-103-3p|rno-miR-107-3p|rno-miR-195-5p|rno-miR-15b-5p
NM_001276483	3	rno-miR-16-5p|rno-miR-195-5p|rno-miR-15b-5p
NM_001128287	7	rno-miR-16-5p|rno-miR-103-3p|rno-miR-107-3p|rno-miR-195-5p|rno-miR-17-5p|rno-miR-15b-5p|rno-miR-93-5p
NM_053751	3	rno-miR-16-5p|rno-miR-195-5p|rno-miR-15b-5p
NM_001108786	5	rno-miR-16-5p|rno-miR-27b-3p|rno-miR-195-5p|rno-miR-27a-3p|rno-miR-15b-5p
NM_001191612	5	rno-miR-16-5p|rno-miR-195-5p|rno-miR-17-5p|rno-miR-15b-5p|rno-miR-93-5p
NM_001110335	8	rno-miR-16-5p|rno-miR-195-5p|rno-miR-205|rno-miR-29a-3p|rno-miR-200b-3p|rno-miR-17-5p|rno-miR-15b-5p|rno-miR-93-5p
NM_001013185	3	rno-miR-16-5p|rno-miR-195-5p|rno-miR-15b-5p

NM_001007750	4	rno-miR-16-5p|rno-miR-195-5p|rno-miR-205|rno-miR-15b-5p
NM_001107795	7	rno-miR-16-5p|rno-miR-125b-5p|rno-miR-103-3p|rno-miR-125a-5p|rno-miR-107-3p|rno-miR-195-5p|rno-miR-15b-5p
NM_017336	5	rno-miR-16-5p|rno-miR-195-5p|rno-miR-17-5p|rno-miR-15b-5p|rno-miR-93-5p
NM_138879	3	rno-miR-16-5p|rno-miR-195-5p|rno-miR-15b-5p
NM_001270712	3	rno-miR-16-5p|rno-miR-195-5p|rno-miR-15b-5p
NM_001107179	5	rno-miR-16-5p|rno-miR-125b-5p|rno-miR-125a-5p|rno-miR-195-5p|rno-miR-15b-5p
NM_001107258	5	rno-miR-16-5p|rno-miR-320-3p|rno-miR-195-5p|rno-miR-152-3p|rno-miR-15b-5p
NM_001287108	5	rno-miR-16-5p|rno-miR-195-5p|rno-miR-29a-3p|rno-miR-200b-3p|rno-miR-15b-5p
NM_001025275	5	rno-miR-16-5p|rno-miR-195-5p|rno-miR-181a-5p|rno-miR-30c-5p|rno-miR-15b-5p
NM_001039101	6	rno-miR-16-5p|rno-miR-103-3p|rno-miR-107-3p|rno-miR-195-5p|rno-miR-205|rno-miR-15b-5p
NM_001107801	3	rno-miR-16-5p|rno-miR-195-5p|rno-miR-15b-5p
NM_001271042	3	rno-miR-16-5p|rno-miR-195-5p|rno-miR-15b-5p
NM_001009180	3	rno-miR-16-5p|rno-miR-195-5p|rno-miR-15b-5p
NM_001108631	7	rno-miR-16-5p|rno-miR-27b-3p|rno-miR-195-5p|rno-miR-205|rno-miR-27a-3p|rno-miR-181a-5p|rno-miR-15b-5p
NM_001004198	4	rno-miR-16-5p|rno-miR-195-5p|rno-miR-152-3p|rno-miR-15b-5p
NM_024128	6	rno-miR-16-5p|rno-miR-103-3p|rno-miR-107-3p|rno-miR-195-5p|rno-miR-30c-5p|rno-miR-15b-5p
NM_001111055	8	rno-miR-16-5p|rno-miR-199a-3p|rno-miR-22-3p|rno-miR-195-5p|rno-miR-181a-5p|rno-miR-17-5p|rno-miR-15b-5p|rno-miR-93-5p
NM_031612	3	rno-miR-16-5p|rno-miR-195-5p|rno-miR-15b-5p
NM_001108386	5	rno-miR-16-5p|rno-miR-199a-3p|rno-miR-195-5p|rno-miR-181a-5p|rno-miR-15b-5p

NM_001126089	3	rno-miR-16-5p|rno-miR-195-5p|rno-miR-15b-5p
NM_001270702	3	rno-miR-16-5p|rno-miR-195-5p|rno-miR-15b-5p
NM_019195	3	rno-miR-16-5p|rno-miR-195-5p|rno-miR-15b-5p
NM_001100519	7	rno-miR-16-5p|rno-miR-125b-5p|rno-miR-103-3p|rno-miR-125a-5p|rno-miR-107-3p|rno-miR-195-5p|rno-miR-15b-5p
NM_001008526	6	rno-miR-16-5p|rno-miR-195-5p|rno-miR-200b-3p|rno-miR-17-5p|rno-miR-15b-5p|rno-miR-93-5p
NM_001287113	5	rno-miR-16-5p|rno-miR-195-5p|rno-miR-29a-3p|rno-miR-200b-3p|rno-miR-15b-5p
NM_133589	5	rno-miR-16-5p|rno-miR-103-3p|rno-miR-107-3p|rno-miR-195-5p|rno-miR-15b-5p
NM_053505	4	rno-miR-16-5p|rno-miR-195-5p|rno-miR-152-3p|rno-miR-15b-5p
NM_001170474	5	rno-miR-16-5p|rno-miR-195-5p|rno-miR-17-5p|rno-miR-15b-5p|rno-miR-93-5p
NM_057138	3	rno-miR-16-5p|rno-miR-195-5p|rno-miR-15b-5p
NM_001033715	3	rno-miR-16-5p|rno-miR-200b-3p|rno-miR-30c-5p
NM_001107711	3	rno-miR-16-5p|rno-miR-195-5p|rno-miR-15b-5p
NM_001170584	4	rno-miR-16-5p|rno-miR-195-5p|rno-miR-31a-5p|rno-miR-15b-5p
NM_001127553	3	rno-miR-16-5p|rno-miR-195-5p|rno-miR-15b-5p
NM_019153	3	rno-miR-16-5p|rno-miR-195-5p|rno-miR-15b-5p
NM_001033970	3	rno-miR-16-5p|rno-miR-195-5p|rno-miR-15b-5p
NM_001015031	3	rno-miR-16-5p|rno-miR-195-5p|rno-miR-15b-5p
NM_001013976	3	rno-miR-16-5p|rno-miR-195-5p|rno-miR-15b-5p
NM_012994	6	rno-miR-16-5p|rno-miR-199a-3p|rno-miR-27b-3p|rno-miR-195-5p|rno-miR-27a-3p|rno-miR-15b-5p
NM_031034	4	rno-miR-16-5p|rno-miR-199a-3p|rno-miR-195-5p|rno-miR-15b-5p
NM_031643	4	rno-miR-16-5p|rno-miR-195-5p|rno-miR-181a-5p|rno-miR-15b-5p
NM_001145141	4	rno-miR-16-5p|rno-miR-195-5p|rno-miR-205|rno-miR-15b-5p
NM_138904	3	rno-miR-16-5p|rno-miR-195-5p|rno-miR-15b-5p
NM_001170398	3	rno-miR-16-5p|rno-miR-195-5p|rno-miR-15b-5p

NM_001305241	4	rno-miR-16-5p|rno-miR-195-5p|rno-miR-378a-3p|rno-miR-15b-5p
NM_001173510	3	rno-miR-16-5p|rno-miR-195-5p|rno-miR-15b-5p
NM_001134639	4	rno-miR-16-5p|rno-miR-195-5p|rno-miR-200b-3p|rno-miR-15b-5p
NM_053691	4	rno-miR-16-5p|rno-miR-195-5p|rno-miR-29a-3p|rno-miR-15b-5p
NM_022626	5	rno-miR-16-5p|rno-miR-103-3p|rno-miR-107-3p|rno-miR-195-5p|rno-miR-15b-5p
NM_001034997	9	rno-miR-16-5p|rno-miR-103-3p|rno-miR-107-3p|rno-miR-195-5p|rno-miR-200b-3p|rno-miR-30c-5p|rno-miR-17-5p|rno-miR-15b-5p|rno-miR-93-5p
NM_001107781	8	rno-miR-16-5p|rno-miR-320-3p|rno-miR-195-5p|rno-miR-200b-3p|rno-miR-181a-5p|rno-miR-17-5p|rno-miR-15b-5p|rno-miR-93-5p
NM_012756	4	rno-miR-16-5p|rno-miR-195-5p|rno-miR-30c-5p|rno-miR-15b-5p
NM_001111056	8	rno-miR-16-5p|rno-miR-199a-3p|rno-miR-22-3p|rno-miR-195-5p|rno-miR-181a-5p|rno-miR-17-5p|rno-miR-15b-5p|rno-miR-93-5p
NM_001100476	5	rno-miR-16-5p|rno-miR-103-3p|rno-miR-107-3p|rno-miR-195-5p|rno-miR-15b-5p
NM_001100172	7	rno-miR-16-5p|rno-miR-27b-3p|rno-miR-195-5p|rno-miR-27a-3p|rno-miR-200b-3p|rno-miR-30c-5p|rno-miR-15b-5p
NM_181087	5	rno-miR-16-5p|rno-miR-195-5p|rno-miR-17-5p|rno-miR-15b-5p|rno-miR-93-5p
NM_001107808	4	rno-miR-16-5p|rno-miR-195-5p|rno-miR-29a-3p|rno-miR-15b-5p
NM_001106327	3	rno-miR-16-5p|rno-miR-195-5p|rno-miR-15b-5p
NM_001173509	3	rno-miR-16-5p|rno-miR-195-5p|rno-miR-15b-5p
NM_022866	4	rno-miR-16-5p|rno-miR-22-3p|rno-miR-195-5p|rno-miR-15b-5p
NM_012953	4	rno-miR-16-5p|rno-miR-22-3p|rno-miR-195-5p|rno-miR-15b-5p
NM_001024882	3	rno-miR-16-5p|rno-miR-195-5p|rno-miR-15b-5p
NM_053933	8	rno-miR-16-5p|rno-miR-320-3p|rno-miR-195-5p|rno-miR-29a-3p|rno-miR-181a-5p|rno-miR-17-5p|rno-miR-15b-5p|rno-miR-93-5p
NM_001012051	5	rno-miR-16-5p|rno-miR-195-5p|rno-miR-17-5p|rno-miR-15b-5p|rno-miR-93-5p

NM_001025664	6	rno-miR-16-5p|rno-miR-27b-3p|rno-miR-195-5p|rno-miR-27a-3p|rno-miR-200b-3p|rno-miR-15b-5p
NM_080909	4	rno-miR-16-5p|rno-miR-195-5p|rno-miR-205|rno-miR-15b-5p
NM_031322	7	rno-miR-16-5p|rno-miR-125b-5p|rno-miR-125a-5p|rno-miR-195-5p|rno-miR-200b-3p|rno-miR-152-3p|rno-miR-15b-5p
NM_001100473	5	rno-miR-16-5p|rno-miR-103-3p|rno-miR-107-3p|rno-miR-195-5p|rno-miR-15b-5p
NM_001270984	5	rno-miR-16-5p|rno-miR-103-3p|rno-miR-107-3p|rno-miR-195-5p|rno-miR-15b-5p
NM_001287107	5	rno-miR-16-5p|rno-miR-195-5p|rno-miR-29a-3p|rno-miR-200b-3p|rno-miR-15b-5p
NM_001009658	3	rno-miR-16-5p|rno-miR-195-5p|rno-miR-15b-5p
NM_001011710	3	rno-miR-16-5p|rno-miR-195-5p|rno-miR-15b-5p
NM_031026	8	rno-miR-16-5p|rno-miR-103-3p|rno-miR-107-3p|rno-miR-195-5p|rno-miR-378a-3p|rno-miR-17-5p|rno-miR-15b-5p|rno-miR-93-5p
NM_001173471	3	rno-miR-16-5p|rno-miR-195-5p|rno-miR-15b-5p
NM_001261387	3	rno-miR-16-5p|rno-miR-195-5p|rno-miR-15b-5p
NM_054006	5	rno-miR-16-5p|rno-miR-125a-5p|rno-miR-195-5p|rno-miR-205|rno-miR-15b-5p
NM_001011904	3	rno-miR-16-5p|rno-miR-195-5p|rno-miR-15b-5p
NM_001303017	3	rno-miR-16-5p|rno-miR-195-5p|rno-miR-15b-5p
NM_134403	5	rno-miR-16-5p|rno-miR-195-5p|rno-miR-205|rno-miR-181a-5p|rno-miR-15b-5p
NM_001044243	4	rno-miR-16-5p|rno-miR-195-5p|rno-miR-30c-5p|rno-miR-15b-5p
NM_001163320	3	rno-miR-16-5p|rno-miR-195-5p|rno-miR-15b-5p
NM_001013228	7	rno-miR-16-5p|rno-miR-125b-5p|rno-miR-103-3p|rno-miR-125a-5p|rno-miR-107-3p|rno-miR-195-5p|rno-miR-15b-5p
NM_001106166	3	rno-miR-16-5p|rno-miR-195-5p|rno-miR-15b-5p
NM_001164396	3	rno-miR-16-5p|rno-miR-195-5p|rno-miR-15b-5p
NM_001106288	3	rno-miR-16-5p|rno-miR-195-5p|rno-miR-15b-5p

NM_001107873	3	rno-miR-16-5p|rno-miR-195-5p|rno-miR-15b-5p
NM_001191549	4	rno-miR-16-5p|rno-miR-320-3p|rno-miR-195-5p|rno-miR-15b-5p
NM_001168703	3	rno-miR-16-5p|rno-miR-195-5p|rno-miR-15b-5p
NM_030838	3	rno-miR-16-5p|rno-miR-195-5p|rno-miR-15b-5p
NM_019214	3	rno-miR-16-5p|rno-miR-195-5p|rno-miR-15b-5p
NM_001100988	4	rno-miR-16-5p|rno-miR-195-5p|rno-miR-30c-5p|rno-miR-15b-5p
NM_001108841	4	rno-miR-16-5p|rno-miR-195-5p|rno-miR-200b-3p|rno-miR-15b-5p
NM_001103354	6	rno-miR-16-5p|rno-miR-27b-3p|rno-miR-195-5p|rno-miR-27a-3p|rno-miR-200b-3p|rno-miR-15b-5p
NM_001109178	3	rno-miR-16-5p|rno-miR-195-5p|rno-miR-15b-5p
NM_139217	3	rno-miR-16-5p|rno-miR-195-5p|rno-miR-15b-5p
NM_001047104	4	rno-miR-16-5p|rno-miR-195-5p|rno-miR-152-3p|rno-miR-15b-5p
NM_001109526	3	rno-miR-16-5p|rno-miR-195-5p|rno-miR-15b-5p
NM_022639	3	rno-miR-16-5p|rno-miR-195-5p|rno-miR-15b-5p
NM_001261388	3	rno-miR-16-5p|rno-miR-195-5p|rno-miR-15b-5p
NM_001107111	4	rno-miR-16-5p|rno-miR-195-5p|rno-miR-181a-5p|rno-miR-15b-5p
NM_013000	5	rno-miR-16-5p|rno-miR-195-5p|rno-miR-200b-3p|rno-miR-181a-5p|rno-miR-15b-5p
NM_001107464	3	rno-miR-16-5p|rno-miR-195-5p|rno-miR-15b-5p
NM_133528	3	rno-miR-16-5p|rno-miR-195-5p|rno-miR-15b-5p
NM_001134983	5	rno-miR-16-5p|rno-miR-199a-3p|rno-miR-195-5p|rno-miR-152-3p|rno-miR-15b-5p
NM_001107646	3	rno-miR-16-5p|rno-miR-195-5p|rno-miR-15b-5p
NM_001127549	4	rno-miR-16-5p|rno-miR-199a-3p|rno-miR-195-5p|rno-miR-15b-5p
NM_080905	4	rno-miR-16-5p|rno-miR-195-5p|rno-miR-200b-3p|rno-miR-15b-5p
NM_001105909	9	rno-miR-16-5p|rno-miR-103-3p|rno-miR-107-3p|rno-miR-27b-3p|rno-miR-195-5p|rno-miR-27a-3p|rno-miR-29a-3p|rno-miR-181a-5p|rno-miR-15b-5p
NM_001136272	3	rno-miR-16-5p|rno-miR-195-5p|rno-miR-15b-5p
NM_012639	3	rno-miR-16-5p|rno-miR-195-5p|rno-miR-15b-5p

NM_001025625	3	rno-miR-16-5p|rno-miR-195-5p|rno-miR-15b-5p
NM_053598	5	rno-miR-16-5p|rno-miR-22-3p|rno-miR-195-5p|rno-miR-200b-3p|rno-miR-15b-5p
NM_001191788	5	rno-miR-16-5p|rno-miR-103-3p|rno-miR-107-3p|rno-miR-195-5p|rno-miR-15b-5p
NM_001110333	5	rno-miR-16-5p|rno-miR-195-5p|rno-miR-29a-3p|rno-miR-200b-3p|rno-miR-15b-5p
NM_001270834	3	rno-miR-16-5p|rno-miR-195-5p|rno-miR-15b-5p
NM_133567	3	rno-miR-16-5p|rno-miR-195-5p|rno-miR-15b-5p
NM_001108406	3	rno-miR-16-5p|rno-miR-195-5p|rno-miR-15b-5p
NM_001107681	4	rno-miR-16-5p|rno-miR-22-3p|rno-miR-195-5p|rno-miR-15b-5p
NM_001105848	4	rno-miR-16-5p|rno-miR-320-3p|rno-miR-195-5p|rno-miR-15b-5p
NM_001107892	3	rno-miR-16-5p|rno-miR-195-5p|rno-miR-15b-5p
NM_057148	5	rno-miR-16-5p|rno-miR-195-5p|rno-miR-17-5p|rno-miR-15b-5p|rno-miR-93-5p
NM_001017454	5	rno-miR-16-5p|rno-miR-125b-5p|rno-miR-125a-5p|rno-miR-195-5p|rno-miR-15b-5p
NM_001270786	3	rno-miR-16-5p|rno-miR-195-5p|rno-miR-15b-5p
NM_001107382	4	rno-miR-16-5p|rno-miR-195-5p|rno-miR-200b-3p|rno-miR-15b-5p
NM_001011908	6	rno-miR-16-5p|rno-miR-27b-3p|rno-miR-195-5p|rno-miR-27a-3p|rno-miR-200b-3p|rno-miR-15b-5p
NM_030856	5	rno-miR-16-5p|rno-miR-103-3p|rno-miR-107-3p|rno-miR-195-5p|rno-miR-15b-5p
NM_001109090	3	rno-miR-16-5p|rno-miR-195-5p|rno-miR-15b-5p
NM_001173371	4	rno-miR-16-5p|rno-miR-22-3p|rno-miR-195-5p|rno-miR-15b-5p
NM_001106272	3	rno-miR-16-5p|rno-miR-195-5p|rno-miR-15b-5p
NM_001077200	6	rno-miR-16-5p|rno-miR-103-3p|rno-miR-107-3p|rno-miR-195-5p|rno-miR-205|rno-miR-15b-5p
NM_053585	3	rno-miR-16-5p|rno-miR-195-5p|rno-miR-15b-5p
NM_001106497	4	rno-miR-16-5p|rno-miR-195-5p|rno-miR-152-3p|rno-miR-15b-5p

NM_001287114	5	rno-miR-16-5p|rno-miR-195-5p|rno-miR-29a-3p|rno-miR-200b-3p|rno-miR-15b-5p
NM_022600	3	rno-miR-16-5p|rno-miR-195-5p|rno-miR-15b-5p
NM_001013870	3	rno-miR-16-5p|rno-miR-195-5p|rno-miR-15b-5p
NM_001105959	3	rno-miR-16-5p|rno-miR-195-5p|rno-miR-15b-5p
NM_001024245	3	rno-miR-16-5p|rno-miR-195-5p|rno-miR-15b-5p
NM_001012094	6	rno-miR-16-5p|rno-miR-195-5p|rno-miR-181a-5p|rno-miR-17-5p|rno-miR-15b-5p|rno-miR-93-5p
NM_016997	3	rno-miR-16-5p|rno-miR-195-5p|rno-miR-15b-5p
NM_001191790	4	rno-miR-16-5p|rno-miR-195-5p|rno-miR-15b-5p|rno-miR-151-5p
NM_013109	3	rno-miR-16-5p|rno-miR-195-5p|rno-miR-15b-5p
NM_001134863	4	rno-miR-16-5p|rno-miR-195-5p|rno-miR-30c-5p|rno-miR-15b-5p
NM_001191918	3	rno-miR-16-5p|rno-miR-195-5p|rno-miR-15b-5p
NM_001270703	3	rno-miR-16-5p|rno-miR-195-5p|rno-miR-15b-5p
NM_001014164	3	rno-miR-16-5p|rno-miR-195-5p|rno-miR-15b-5p
NM_019264	3	rno-miR-16-5p|rno-miR-195-5p|rno-miR-15b-5p
NM_001007148	4	rno-miR-16-5p|rno-miR-195-5p|rno-miR-200b-3p|rno-miR-15b-5p
NM_001261389	3	rno-miR-16-5p|rno-miR-195-5p|rno-miR-15b-5p
NM_001107889	5	rno-miR-16-5p|rno-miR-125b-5p|rno-miR-125a-5p|rno-miR-195-5p|rno-miR-15b-5p
NM_001107300	7	rno-miR-16-5p|rno-miR-103-3p|rno-miR-107-3p|rno-miR-195-5p|rno-miR-29a-3p|rno-miR-181a-5p|rno-miR-15b-5p
NM_001106747	8	rno-miR-16-5p|rno-miR-3473|rno-miR-195-5p|rno-miR-205|rno-miR-31a-5p|rno-miR-17-5p|rno-miR-15b-5p|rno-miR-93-5p
NM_001014029	3	rno-miR-16-5p|rno-miR-195-5p|rno-miR-15b-5p
NM_001108657	5	rno-miR-16-5p|rno-miR-103-3p|rno-miR-107-3p|rno-miR-195-5p|rno-miR-15b-5p
NM_001009632	3	rno-miR-16-5p|rno-miR-195-5p|rno-miR-15b-5p
NM_001106661	3	rno-miR-16-5p|rno-miR-195-5p|rno-miR-15b-5p
NM_019232	4	rno-miR-16-5p|rno-miR-195-5p|rno-miR-29a-3p|rno-miR-15b-5p

NM_001109018	7	rno-miR-16-5p|rno-miR-103-3p|rno-miR-107-3p|rno-miR-195-5p|rno-miR-205|rno-miR-31a-5p|rno-miR-15b-5p
NM_001013948	5	rno-miR-16-5p|rno-miR-27b-3p|rno-miR-195-5p|rno-miR-27a-3p|rno-miR-15b-5p
NM_001193569	4	rno-miR-16-5p|rno-miR-195-5p|rno-miR-29a-3p|rno-miR-15b-5p
NM_001107345	4	rno-miR-16-5p|rno-miR-195-5p|rno-miR-200b-3p|rno-miR-15b-5p
NM_001011896	3	rno-miR-16-5p|rno-miR-195-5p|rno-miR-15b-5p
NM_001270787	3	rno-miR-16-5p|rno-miR-195-5p|rno-miR-15b-5p
NM_017223	3	rno-miR-16-5p|rno-miR-195-5p|rno-miR-15b-5p
NM_001009689	3	rno-miR-16-5p|rno-miR-195-5p|rno-miR-15b-5p
NM_001171177	5	rno-miR-16-5p|rno-miR-103-3p|rno-miR-107-3p|rno-miR-195-5p|rno-miR-15b-5p
NM_001110336	5	rno-miR-16-5p|rno-miR-195-5p|rno-miR-29a-3p|rno-miR-200b-3p|rno-miR-15b-5p
NM_001108432	6	rno-miR-16-5p|rno-miR-195-5p|rno-miR-17-5p|rno-miR-152-3p|rno-miR-15b-5p|rno-miR-93-5p
NM_053761	3	rno-miR-16-5p|rno-miR-195-5p|rno-miR-15b-5p
NM_001107248	6	rno-miR-16-5p|rno-miR-103-3p|rno-miR-107-3p|rno-miR-195-5p|rno-miR-29a-3p|rno-miR-15b-5p
NM_001309449	6	rno-miR-16-5p|rno-miR-103-3p|rno-miR-107-3p|rno-miR-195-5p|rno-miR-200b-3p|rno-miR-15b-5p
NM_001108495	3	rno-miR-16-5p|rno-miR-195-5p|rno-miR-15b-5p
NM_183326	5	rno-miR-16-5p|rno-miR-195-5p|rno-miR-181a-5p|rno-miR-152-3p|rno-miR-15b-5p
NM_001110491	6	rno-miR-16-5p|rno-miR-125b-5p|rno-miR-125a-5p|rno-miR-195-5p|rno-miR-152-3p|rno-miR-15b-5p
NM_198787	5	rno-miR-16-5p|rno-miR-103-3p|rno-miR-107-3p|rno-miR-195-5p|rno-miR-15b-5p
NM_001108374	3	rno-miR-16-5p|rno-miR-195-5p|rno-miR-15b-5p
NM_001110838	3	rno-miR-16-5p|rno-miR-195-5p|rno-miR-15b-5p

NM_001013153	5	rno-miR-16-5p|rno-miR-125b-5p|rno-miR-125a-5p|rno-miR-195-5p|rno-miR-15b-5p
NM_001317043	5	rno-miR-16-5p|rno-miR-195-5p|rno-miR-29a-3p|rno-miR-200b-3p|rno-miR-15b-5p
NM_138881	5	rno-miR-16-5p|rno-miR-27b-3p|rno-miR-195-5p|rno-miR-27a-3p|rno-miR-15b-5p
NM_133522	5	rno-miR-16-5p|rno-miR-125b-5p|rno-miR-125a-5p|rno-miR-195-5p|rno-miR-15b-5p
NM_001287110	5	rno-miR-16-5p|rno-miR-195-5p|rno-miR-29a-3p|rno-miR-200b-3p|rno-miR-15b-5p
NM_001127572	3	rno-miR-16-5p|rno-miR-195-5p|rno-miR-15b-5p
NM_001126300	3	rno-miR-16-5p|rno-miR-195-5p|rno-miR-15b-5p
NM_001007005	3	rno-miR-16-5p|rno-miR-195-5p|rno-miR-15b-5p
NM_001257278	3	rno-miR-16-5p|rno-miR-195-5p|rno-miR-15b-5p
NM_001109204	5	rno-miR-16-5p|rno-miR-103-3p|rno-miR-107-3p|rno-miR-195-5p|rno-miR-15b-5p
NM_001107324	6	rno-miR-16-5p|rno-miR-125b-5p|rno-miR-125a-5p|rno-miR-320-3p|rno-miR-195-5p|rno-miR-15b-5p
NM_001107496	3	rno-miR-16-5p|rno-miR-195-5p|rno-miR-15b-5p
NM_001108420	5	rno-miR-16-5p|rno-miR-125b-5p|rno-miR-125a-5p|rno-miR-195-5p|rno-miR-15b-5p
NM_017355	4	rno-miR-16-5p|rno-miR-195-5p|rno-miR-30c-5p|rno-miR-15b-5p
NM_001305243	5	rno-miR-16-5p|rno-miR-22-3p|rno-miR-195-5p|rno-miR-30c-5p|rno-miR-15b-5p
NM_001111057	8	rno-miR-16-5p|rno-miR-199a-3p|rno-miR-22-3p|rno-miR-195-5p|rno-miR-181a-5p|rno-miR-17-5p|rno-miR-15b-5p|rno-miR-93-5p
NM_053477	3	rno-miR-16-5p|rno-miR-195-5p|rno-miR-15b-5p
NM_001135094	3	rno-miR-16-5p|rno-miR-195-5p|rno-miR-15b-5p
NM_001013860	3	rno-miR-16-5p|rno-miR-195-5p|rno-miR-15b-5p

NM_001106743	5	rno-miR-16-5p|rno-miR-125b-5p|rno-miR-125a-5p|rno-miR-195-5p|rno-miR-15b-5p
NM_001107732	3	rno-miR-16-5p|rno-miR-195-5p|rno-miR-15b-5p
NM_001008354	5	rno-miR-16-5p|rno-miR-27b-3p|rno-miR-195-5p|rno-miR-27a-3p|rno-miR-15b-5p
NM_031529	5	rno-miR-16-5p|rno-miR-195-5p|rno-miR-29a-3p|rno-miR-30c-5p|rno-miR-15b-5p
NM_053384	3	rno-miR-16-5p|rno-miR-195-5p|rno-miR-15b-5p
NM_001107962	3	rno-miR-16-5p|rno-miR-195-5p|rno-miR-15b-5p
NM_001270628	3	rno-miR-16-5p|rno-miR-195-5p|rno-miR-15b-5p
NM_001108342	5	rno-miR-16-5p|rno-miR-320-3p|rno-miR-195-5p|rno-miR-181a-5p|rno-miR-15b-5p
NM_001134643	4	rno-miR-16-5p|rno-miR-195-5p|rno-miR-181a-5p|rno-miR-15b-5p
NM_019141	7	rno-miR-16-5p|rno-miR-103-3p|rno-miR-107-3p|rno-miR-195-5p|rno-miR-17-5p|rno-miR-15b-5p|rno-miR-93-5p
NM_013038	3	rno-miR-16-5p|rno-miR-195-5p|rno-miR-15b-5p
NM_021697	3	rno-miR-16-5p|rno-miR-195-5p|rno-miR-15b-5p
NM_212490	3	rno-miR-16-5p|rno-miR-195-5p|rno-miR-15b-5p
NM_001106567	5	rno-miR-16-5p|rno-miR-195-5p|rno-miR-29a-3p|rno-miR-30c-5p|rno-miR-15b-5p
NM_001287111	5	rno-miR-16-5p|rno-miR-195-5p|rno-miR-29a-3p|rno-miR-200b-3p|rno-miR-15b-5p
NM_001191819	6	rno-miR-16-5p|rno-miR-27b-3p|rno-miR-195-5p|rno-miR-27a-3p|rno-miR-181a-5p|rno-miR-15b-5p
NM_001009661	3	rno-miR-16-5p|rno-miR-195-5p|rno-miR-15b-5p
NM_001143750	6	rno-miR-16-5p|rno-miR-27b-3p|rno-miR-195-5p|rno-miR-27a-3p|rno-miR-152-3p|rno-miR-15b-5p
NM_001106664	3	rno-miR-16-5p|rno-miR-195-5p|rno-miR-15b-5p
NM_001135046	4	rno-miR-16-5p|rno-miR-195-5p|rno-miR-200b-3p|rno-miR-15b-5p
NM_001170328	3	rno-miR-16-5p|rno-miR-195-5p|rno-miR-15b-5p

NM_001173376	4	rno-miR-16-5p|rno-miR-320-3p|rno-miR-195-5p|rno-miR-15b-5p
NM_053918	3	rno-miR-16-5p|rno-miR-195-5p|rno-miR-15b-5p
NM_001031661	5	rno-miR-16-5p|rno-miR-103-3p|rno-miR-107-3p|rno-miR-195-5p|rno-miR-15b-5p
NM_178866	4	rno-miR-16-5p|rno-miR-195-5p|rno-miR-29a-3p|rno-miR-15b-5p
NM_001012119	4	rno-miR-16-5p|rno-miR-195-5p|rno-miR-29a-3p|rno-miR-15b-5p
NM_001191871	3	rno-miR-16-5p|rno-miR-195-5p|rno-miR-15b-5p
NM_001106857	4	rno-miR-16-5p|rno-miR-195-5p|rno-miR-152-3p|rno-miR-15b-5p
NM_001108142	3	rno-miR-16-5p|rno-miR-195-5p|rno-miR-15b-5p
NM_001109238	5	rno-miR-16-5p|rno-miR-103-3p|rno-miR-107-3p|rno-miR-195-5p|rno-miR-15b-5p
NM_001025658	5	rno-miR-16-5p|rno-miR-103-3p|rno-miR-107-3p|rno-miR-195-5p|rno-miR-15b-5p
NM_053339	3	rno-miR-16-5p|rno-miR-195-5p|rno-miR-15b-5p
NM_001106544	3	rno-miR-16-5p|rno-miR-195-5p|rno-miR-15b-5p
NM_012894	8	rno-miR-16-5p|rno-miR-199a-3p|rno-miR-22-3p|rno-miR-195-5p|rno-miR-181a-5p|rno-miR-17-5p|rno-miR-15b-5p|rno-miR-93-5p
NM_001107005	5	rno-miR-16-5p|rno-miR-27b-3p|rno-miR-195-5p|rno-miR-27a-3p|rno-miR-15b-5p
NM_001127201	4	rno-miR-16-5p|rno-miR-195-5p|rno-miR-181a-5p|rno-miR-15b-5p
NM_052803	6	rno-miR-16-5p|rno-miR-125b-5p|rno-miR-125a-5p|rno-miR-195-5p|rno-miR-152-3p|rno-miR-15b-5p
NM_024366	4	rno-miR-16-5p|rno-miR-195-5p|rno-miR-200b-3p|rno-miR-15b-5p
NM_001024900	4	rno-miR-16-5p|rno-miR-195-5p|rno-miR-181a-5p|rno-miR-15b-5p
NM_001107527	3	rno-miR-16-5p|rno-miR-195-5p|rno-miR-15b-5p
NM_031571	4	rno-miR-16-5p|rno-miR-199a-3p|rno-miR-195-5p|rno-miR-15b-5p
NM_001109316	4	rno-miR-16-5p|rno-miR-320-3p|rno-miR-195-5p|rno-miR-15b-5p
NM_145777	6	rno-miR-16-5p|rno-miR-27b-3p|rno-miR-195-5p|rno-miR-27a-3p|rno-miR-181a-5p|rno-miR-15b-5p
NM_199256	3	rno-miR-16-5p|rno-miR-195-5p|rno-miR-15b-5p

NM_001008382	4	rno-miR-16-5p|rno-miR-195-5p|rno-miR-30c-5p|rno-miR-15b-5p
NM_001025274	11	rno-miR-16-5p|rno-miR-103-3p|rno-miR-107-3p|rno-miR-27b-3p|rno-miR-22-3p|rno-miR-195-5p|rno-miR-27a-3p|rno-miR-200b-3p|rno-miR-181a-5p|rno-miR-30c-5p|rno-miR-15b-5p
NM_001270627	3	rno-miR-16-5p|rno-miR-195-5p|rno-miR-15b-5p
NM_001024880	5	rno-miR-16-5p|rno-miR-103-3p|rno-miR-107-3p|rno-miR-195-5p|rno-miR-15b-5p
NM_001108246	5	rno-miR-16-5p|rno-miR-195-5p|rno-miR-29a-3p|rno-miR-181a-5p|rno-miR-15b-5p
NM_001108262	5	rno-miR-16-5p|rno-miR-195-5p|rno-miR-205|rno-miR-181a-5p|rno-miR-15b-5p
NM_001107178	4	rno-miR-16-5p|rno-miR-195-5p|rno-miR-152-3p|rno-miR-15b-5p
NM_001246183	3	rno-miR-16-5p|rno-miR-195-5p|rno-miR-15b-5p
NM_001127542	3	rno-miR-16-5p|rno-miR-195-5p|rno-miR-15b-5p
NM_001107661	3	rno-miR-16-5p|rno-miR-195-5p|rno-miR-15b-5p
NM_001109668	6	rno-miR-16-5p|rno-miR-195-5p|rno-miR-378a-3p|rno-miR-17-5p|rno-miR-15b-5p|rno-miR-93-5p
NM_053346	3	rno-miR-16-5p|rno-miR-195-5p|rno-miR-15b-5p
NM_001108023	10	rno-miR-16-5p|rno-miR-320-3p|rno-miR-27b-3p|rno-miR-195-5p|rno-miR-378a-3p|rno-miR-27a-3p|rno-miR-200b-3p|rno-miR-181a-5p|rno-miR-152-3p|rno-miR-15b-5p
NM_001106636	3	rno-miR-16-5p|rno-miR-195-5p|rno-miR-15b-5p
NM_001108557	4	rno-miR-16-5p|rno-miR-195-5p|rno-miR-31a-5p|rno-miR-15b-5p
NM_001103353	3	rno-miR-16-5p|rno-miR-195-5p|rno-miR-15b-5p
NM_001106854	5	rno-miR-16-5p|rno-miR-125b-5p|rno-miR-125a-5p|rno-miR-195-5p|rno-miR-15b-5p
NM_183332	3	rno-miR-16-5p|rno-miR-195-5p|rno-miR-15b-5p
NM_001108807	5	rno-miR-16-5p|rno-miR-125b-5p|rno-miR-125a-5p|rno-miR-195-5p|rno-miR-15b-5p
NM_001034150	3	rno-miR-16-5p|rno-miR-195-5p|rno-miR-15b-5p

NM_001270626	3	rno-miR-16-5p|rno-miR-195-5p|rno-miR-15b-5p
NM_001108447	5	rno-miR-16-5p|rno-miR-195-5p|rno-miR-29a-3p|rno-miR-30c-5p|rno-miR-15b-5p
NM_001271136	6	rno-miR-16-5p|rno-miR-195-5p|rno-miR-181a-5p|rno-miR-17-5p|rno-miR-15b-5p|rno-miR-93-5p
NM_017359	10	rno-miR-16-5p|rno-miR-103-3p|rno-miR-107-3p|rno-miR-195-5p|rno-miR-378a-3p|rno-miR-30c-5p|rno-miR-17-5p|rno-miR-152-3p|rno-miR-15b-5p|rno-miR-93-5p
NM_019365	5	rno-miR-16-5p|rno-miR-103-3p|rno-miR-107-3p|rno-miR-195-5p|rno-miR-15b-5p
NM_001108417	4	rno-miR-16-5p|rno-miR-195-5p|rno-miR-30c-5p|rno-miR-15b-5p
NM_080394	7	rno-miR-16-5p|rno-miR-27b-3p|rno-miR-195-5p|rno-miR-205|rno-miR-27a-3p|rno-miR-200b-3p|rno-miR-15b-5p
NM_001017474	5	rno-miR-16-5p|rno-miR-125b-5p|rno-miR-125a-5p|rno-miR-195-5p|rno-miR-15b-5p
NM_001011930	3	rno-miR-16-5p|rno-miR-195-5p|rno-miR-15b-5p
NM_019333	3	rno-miR-16-5p|rno-miR-195-5p|rno-miR-15b-5p
NM_013034	3	rno-miR-16-5p|rno-miR-195-5p|rno-miR-15b-5p
NM_022929	3	rno-miR-16-5p|rno-miR-195-5p|rno-miR-15b-5p
NM_213564	5	rno-miR-16-5p|rno-miR-195-5p|rno-miR-17-5p|rno-miR-15b-5p|rno-miR-93-5p
NM_001107016	3	rno-miR-16-5p|rno-miR-195-5p|rno-miR-15b-5p
NM_001107843	7	rno-miR-16-5p|rno-miR-103-3p|rno-miR-107-3p|rno-miR-195-5p|rno-miR-205|rno-miR-200b-3p|rno-miR-15b-5p
NM_001025134	3	rno-miR-16-5p|rno-miR-195-5p|rno-miR-15b-5p
NM_001109650	5	rno-miR-16-5p|rno-miR-27b-3p|rno-miR-195-5p|rno-miR-27a-3p|rno-miR-15b-5p
NM_212542	4	rno-miR-16-5p|rno-miR-195-5p|rno-miR-31a-5p|rno-miR-15b-5p
NM_001082477	5	rno-miR-16-5p|rno-miR-320-3p|rno-miR-195-5p|rno-miR-29a-3p|rno-miR-15b-5p

NM_001106816	3	rno-miR-16-5p|rno-miR-195-5p|rno-miR-15b-5p
NM_001127528	5	rno-miR-16-5p|rno-miR-125b-5p|rno-miR-125a-5p|rno-miR-195-5p|rno-miR-15b-5p
NM_001037197	7	rno-miR-16-5p|rno-miR-320-3p|rno-miR-195-5p|rno-miR-200b-3p|rno-miR-181a-5p|rno-miR-31a-5p|rno-miR-15b-5p
NM_001107067	3	rno-miR-16-5p|rno-miR-195-5p|rno-miR-15b-5p
NM_001108982	7	rno-miR-16-5p|rno-miR-195-5p|rno-miR-29a-3p|rno-miR-200b-3p|rno-miR-17-5p|rno-miR-15b-5p|rno-miR-93-5p
NM_130738	3	rno-miR-16-5p|rno-miR-195-5p|rno-miR-15b-5p
NM_001004107	6	rno-miR-16-5p|rno-miR-320-3p|rno-miR-195-5p|rno-miR-378a-3p|rno-miR-31a-5p|rno-miR-15b-5p
NM_001108236	3	rno-miR-16-5p|rno-miR-195-5p|rno-miR-15b-5p
NM_001106640	8	rno-miR-16-5p|rno-miR-103-3p|rno-miR-107-3p|rno-miR-195-5p|rno-miR-30c-5p|rno-miR-17-5p|rno-miR-15b-5p|rno-miR-93-5p
NM_001163168	7	rno-miR-16-5p|rno-miR-103-3p|rno-miR-107-3p|rno-miR-199a-3p|rno-miR-22-3p|rno-miR-195-5p|rno-miR-15b-5p
NM_001042561	6	rno-miR-16-5p|rno-miR-27b-3p|rno-miR-195-5p|rno-miR-27a-3p|rno-miR-200b-3p|rno-miR-15b-5p
NM_001008353	3	rno-miR-16-5p|rno-miR-195-5p|rno-miR-15b-5p
NM_001164726	4	rno-miR-16-5p|rno-miR-195-5p|rno-miR-31a-5p|rno-miR-15b-5p
NM_031836	5	rno-miR-16-5p|rno-miR-195-5p|rno-miR-29a-3p|rno-miR-200b-3p|rno-miR-15b-5p
NM_022632	4	rno-miR-16-5p|rno-miR-195-5p|rno-miR-200b-3p|rno-miR-15b-5p
NM_001107242	3	rno-miR-16-5p|rno-miR-195-5p|rno-miR-15b-5p
NM_138833	4	rno-miR-16-5p|rno-miR-22-3p|rno-miR-195-5p|rno-miR-15b-5p
NM_019234	3	rno-miR-16-5p|rno-miR-195-5p|rno-miR-15b-5p
NM_001048243	3	rno-miR-16-5p|rno-miR-195-5p|rno-miR-15b-5p
NM_001017467	3	rno-miR-16-5p|rno-miR-195-5p|rno-miR-15b-5p
NM_001134633	3	rno-miR-16-5p|rno-miR-195-5p|rno-miR-15b-5p
NM_001109418	4	rno-miR-16-5p|rno-miR-195-5p|rno-miR-30c-5p|rno-miR-15b-5p

NM_001005898	5	rno-miR-16-5p|rno-miR-195-5p|rno-miR-205|rno-miR-30c-5p|rno-miR-15b-5p
NM_001108053	4	rno-miR-16-5p|rno-miR-195-5p|rno-miR-29a-3p|rno-miR-15b-5p
NM_001012348	3	rno-miR-16-5p|rno-miR-195-5p|rno-miR-15b-5p
NM_001105987	3	rno-miR-16-5p|rno-miR-195-5p|rno-miR-15b-5p
NM_022286	3	rno-miR-16-5p|rno-miR-195-5p|rno-miR-15b-5p
NM_001105925	3	rno-miR-16-5p|rno-miR-195-5p|rno-miR-15b-5p
NM_001191689	3	rno-miR-16-5p|rno-miR-195-5p|rno-miR-15b-5p
NM_001100991	3	rno-miR-16-5p|rno-miR-195-5p|rno-miR-15b-5p
NM_001106735	6	rno-miR-16-5p|rno-miR-195-5p|rno-miR-29a-3p|rno-miR-17-5p|rno-miR-15b-5p|rno-miR-93-5p
NM_001110345	5	rno-miR-16-5p|rno-miR-27b-3p|rno-miR-195-5p|rno-miR-27a-3p|rno-miR-15b-5p
NM_001025022	3	rno-miR-16-5p|rno-miR-195-5p|rno-miR-15b-5p
NM_001130567	3	rno-miR-16-5p|rno-miR-195-5p|rno-miR-15b-5p
NM_001109598	5	rno-miR-16-5p|rno-miR-125b-5p|rno-miR-125a-5p|rno-miR-195-5p|rno-miR-15b-5p
NM_001105807	6	rno-miR-16-5p|rno-miR-27b-3p|rno-miR-195-5p|rno-miR-27a-3p|rno-miR-152-3p|rno-miR-15b-5p
NM_001107595	5	rno-miR-16-5p|rno-miR-195-5p|rno-miR-17-5p|rno-miR-15b-5p|rno-miR-93-5p
NM_001287112	5	rno-miR-16-5p|rno-miR-195-5p|rno-miR-29a-3p|rno-miR-200b-3p|rno-miR-15b-5p
NM_001107954	5	rno-miR-16-5p|rno-miR-195-5p|rno-miR-200b-3p|rno-miR-181a-5p|rno-miR-15b-5p
NM_053563	3	rno-miR-16-5p|rno-miR-195-5p|rno-miR-15b-5p
NM_001109246	3	rno-miR-16-5p|rno-miR-195-5p|rno-miR-15b-5p
NM_001107212	4	rno-miR-16-5p|rno-miR-195-5p|rno-miR-30c-5p|rno-miR-15b-5p
NM_001191618	3	rno-miR-16-5p|rno-miR-195-5p|rno-miR-15b-5p

NM_022548	5	rno-miR-16-5p|rno-miR-27b-3p|rno-miR-195-5p|rno-miR-27a-3p|rno-miR-15b-5p
NM_001009652	3	rno-miR-16-5p|rno-miR-195-5p|rno-miR-15b-5p
NM_001103357	5	rno-miR-16-5p|rno-miR-103-3p|rno-miR-107-3p|rno-miR-195-5p|rno-miR-15b-5p
NM_001109513	4	rno-miR-16-5p|rno-miR-195-5p|rno-miR-30c-5p|rno-miR-15b-5p
NM_001011941	8	rno-miR-16-5p|rno-miR-103-3p|rno-miR-107-3p|rno-miR-195-5p|rno-miR-30c-5p|rno-miR-17-5p|rno-miR-15b-5p|rno-miR-93-5p
NM_023021	3	rno-miR-16-5p|rno-miR-195-5p|rno-miR-15b-5p
NM_053355	3	rno-miR-16-5p|rno-miR-195-5p|rno-miR-15b-5p
NM_013192	8	rno-miR-16-5p|rno-miR-27b-3p|rno-miR-195-5p|rno-miR-27a-3p|rno-miR-30c-5p|rno-miR-17-5p|rno-miR-15b-5p|rno-miR-93-5p
NM_001108453	5	rno-miR-16-5p|rno-miR-27b-3p|rno-miR-195-5p|rno-miR-27a-3p|rno-miR-15b-5p
NM_001014198	7	rno-miR-16-5p|rno-miR-103-3p|rno-miR-107-3p|rno-miR-195-5p|rno-miR-181a-5p|rno-miR-30c-5p|rno-miR-15b-5p
NM_001107158	5	rno-miR-16-5p|rno-miR-103-3p|rno-miR-107-3p|rno-miR-195-5p|rno-miR-15b-5p
NM_013052	7	rno-miR-16-5p|rno-miR-103-3p|rno-miR-107-3p|rno-miR-320-3p|rno-miR-195-5p|rno-miR-31a-5p|rno-miR-15b-5p
NM_001107545	6	rno-miR-16-5p|rno-miR-22-3p|rno-miR-195-5p|rno-miR-17-5p|rno-miR-15b-5p|rno-miR-93-5p
NM_001107539	3	rno-miR-16-5p|rno-miR-195-5p|rno-miR-15b-5p
NM_001033716	3	rno-miR-16-5p|rno-miR-200b-3p|rno-miR-30c-5p
NM_001024323	3	rno-miR-16-5p|rno-miR-195-5p|rno-miR-15b-5p
NM_133562	5	rno-miR-16-5p|rno-miR-27b-3p|rno-miR-195-5p|rno-miR-27a-3p|rno-miR-15b-5p
NM_013073	5	rno-miR-16-5p|rno-miR-27b-3p|rno-miR-195-5p|rno-miR-27a-3p|rno-miR-15b-5p

NM_001107524	7	rno-miR-16-5p|rno-miR-195-5p|rno-miR-181a-5p|rno-miR-17-5p|rno-miR-152-3p|rno-miR-15b-5p|rno-miR-93-5p
NM_001009704	5	rno-miR-16-5p|rno-miR-103-3p|rno-miR-107-3p|rno-miR-195-5p|rno-miR-15b-5p
NM_001014012	3	rno-miR-16-5p|rno-miR-195-5p|rno-miR-15b-5p
NM_001024793	4	rno-miR-16-5p|rno-miR-195-5p|rno-miR-205|rno-miR-15b-5p
NM_001009953	4	rno-miR-16-5p|rno-miR-195-5p|rno-miR-205|rno-miR-15b-5p
NM_012643	6	rno-miR-16-5p|rno-miR-103-3p|rno-miR-107-3p|rno-miR-195-5p|rno-miR-200b-3p|rno-miR-15b-5p
NM_001107725	3	rno-miR-16-5p|rno-miR-195-5p|rno-miR-15b-5p
NM_013193	5	rno-miR-16-5p|rno-miR-195-5p|rno-miR-29a-3p|rno-miR-31a-5p|rno-miR-15b-5p
NM_012820	4	rno-miR-16-5p|rno-miR-195-5p|rno-miR-205|rno-miR-15b-5p
NM_001009967	3	rno-miR-16-5p|rno-miR-195-5p|rno-miR-15b-5p
NM_001108256	5	rno-miR-16-5p|rno-miR-195-5p|rno-miR-17-5p|rno-miR-15b-5p|rno-miR-93-5p
NM_001012190	5	rno-miR-16-5p|rno-miR-195-5p|rno-miR-17-5p|rno-miR-15b-5p|rno-miR-93-5p
NM_032079	4	rno-miR-16-5p|rno-miR-320-3p|rno-miR-195-5p|rno-miR-15b-5p
NM_053628	5	rno-miR-16-5p|rno-miR-125b-5p|rno-miR-125a-5p|rno-miR-195-5p|rno-miR-15b-5p
NM_031349	6	rno-miR-16-5p|rno-miR-195-5p|rno-miR-205|rno-miR-29a-3p|rno-miR-31a-5p|rno-miR-15b-5p
NM_031711	3	rno-miR-16-5p|rno-miR-195-5p|rno-miR-15b-5p
NM_001007235	5	rno-miR-16-5p|rno-miR-103-3p|rno-miR-107-3p|rno-miR-195-5p|rno-miR-15b-5p
NM_001110334	5	rno-miR-16-5p|rno-miR-195-5p|rno-miR-29a-3p|rno-miR-200b-3p|rno-miR-15b-5p
NM_001034941	3	rno-miR-16-5p|rno-miR-195-5p|rno-miR-15b-5p
NM_001037654	4	rno-miR-16-5p|rno-miR-195-5p|rno-miR-378a-3p|rno-miR-15b-5p

NM_001107051	5	rno-miR-16-5p|rno-miR-195-5p|rno-miR-17-5p|rno-miR-15b-5p|rno-miR-93-5p
NM_030830	4	rno-miR-16-5p|rno-miR-195-5p|rno-miR-181a-5p|rno-miR-15b-5p
NM_001007597	5	rno-miR-16-5p|rno-miR-27b-3p|rno-miR-195-5p|rno-miR-27a-3p|rno-miR-15b-5p
NM_001108338	3	rno-miR-16-5p|rno-miR-195-5p|rno-miR-15b-5p
NM_001013235	3	rno-miR-16-5p|rno-miR-195-5p|rno-miR-15b-5p
NM_001270701	3	rno-miR-16-5p|rno-miR-195-5p|rno-miR-15b-5p
NM_001082478	4	rno-miR-16-5p|rno-miR-195-5p|rno-miR-29a-3p|rno-miR-15b-5p
NM_001134628	4	rno-miR-16-5p|rno-miR-195-5p|rno-miR-200b-3p|rno-miR-15b-5p
NM_001191848	4	rno-miR-16-5p|rno-miR-3473|rno-miR-195-5p|rno-miR-15b-5p
NM_001033707	3	rno-miR-16-5p|rno-miR-195-5p|rno-miR-15b-5p
NM_053295	3	rno-miR-16-5p|rno-miR-200b-3p|rno-miR-30c-5p
NM_022867	3	rno-miR-16-5p|rno-miR-195-5p|rno-miR-15b-5p
NM_001009639	2	rno-miR-16-5p|rno-miR-195-5p
NM_022275	5	rno-miR-16-5p|rno-miR-27b-3p|rno-miR-195-5p|rno-miR-27a-3p|rno-miR-15b-5p
NM_017044	5	rno-miR-16-5p|rno-miR-103-3p|rno-miR-107-3p|rno-miR-195-5p|rno-miR-15b-5p
NM_001108724	7	rno-miR-16-5p|rno-miR-103-3p|rno-miR-107-3p|rno-miR-22-3p|rno-miR-195-5p|rno-miR-29a-3p|rno-miR-15b-5p
NM_001191725	3	rno-miR-16-5p|rno-miR-195-5p|rno-miR-15b-5p
NM_133620	11	rno-miR-16-5p|rno-miR-103-3p|rno-miR-107-3p|rno-miR-320-3p|rno-miR-199a-3p|rno-miR-27b-3p|rno-miR-195-5p|rno-miR-205|rno-miR-27a-3p|rno-miR-29a-3p|rno-miR-15b-5p
NM_001037214	4	rno-miR-16-5p|rno-miR-195-5p|rno-miR-29a-3p|rno-miR-15b-5p
NM_001108385	3	rno-miR-16-5p|rno-miR-195-5p|rno-miR-15b-5p
NM_001007615	3	rno-miR-16-5p|rno-miR-195-5p|rno-miR-15b-5p
NM_001082479	4	rno-miR-16-5p|rno-miR-195-5p|rno-miR-29a-3p|rno-miR-15b-5p
NM_001107784	3	rno-miR-16-5p|rno-miR-195-5p|rno-miR-15b-5p

NM_001108507	3	rno-miR-16-5p|rno-miR-195-5p|rno-miR-15b-5p
NM_012609	6	rno-miR-16-5p|rno-miR-103-3p|rno-miR-107-3p|rno-miR-195-5p|rno-miR-205|rno-miR-15b-5p
NM_001011915	5	rno-miR-16-5p|rno-miR-195-5p|rno-miR-29a-3p|rno-miR-200b-3p|rno-miR-15b-5p
NM_001108764	4	rno-miR-16-5p|rno-miR-195-5p|rno-miR-29a-3p|rno-miR-15b-5p
NM_053888	3	rno-miR-16-5p|rno-miR-195-5p|rno-miR-15b-5p
NM_031548	2	rno-miR-125b-5p|rno-miR-125a-5p
NM_001037556	2	rno-miR-125b-5p|rno-miR-125a-5p
NM_001271050	2	rno-miR-125b-5p|rno-miR-125a-5p
NM_001108643	2	rno-miR-125b-5p|rno-miR-125a-5p
NM_001012219	7	rno-miR-125b-5p|rno-miR-125a-5p|rno-miR-27b-3p|rno-miR-27a-3p|rno-miR-30c-5p|rno-miR-17-5p|rno-miR-93-5p
NM_001109462	2	rno-miR-125b-5p|rno-miR-125a-5p
NM_030585	2	rno-miR-125b-5p|rno-miR-125a-5p
NM_001170404	2	rno-miR-125b-5p|rno-miR-125a-5p
NM_001107163	3	rno-miR-125b-5p|rno-miR-125a-5p|rno-miR-205
NM_001107034	2	rno-miR-125b-5p|rno-miR-125a-5p
NM_024154	2	rno-miR-125b-5p|rno-miR-125a-5p
NM_019246	2	rno-miR-125b-5p|rno-miR-125a-5p
NM_001109459	2	rno-miR-125b-5p|rno-miR-125a-5p
NM_001108527	4	rno-miR-125b-5p|rno-miR-125a-5p|rno-miR-27b-3p|rno-miR-27a-3p
NM_001270801	4	rno-miR-125b-5p|rno-miR-103-3p|rno-miR-125a-5p|rno-miR-107-3p
NM_001108004	3	rno-miR-125b-5p|rno-miR-125a-5p|rno-miR-200b-3p
NM_001025716	4	rno-miR-125b-5p|rno-miR-103-3p|rno-miR-125a-5p|rno-miR-107-3p
NM_001005890	2	rno-miR-125b-5p|rno-miR-125a-5p
NM_030842	2	rno-miR-125b-5p|rno-miR-125a-5p
NM_001105928	3	rno-miR-125b-5p|rno-miR-125a-5p|rno-miR-320-3p
NM_001009601	2	rno-miR-125b-5p|rno-miR-125a-5p
NM_001130566	2	rno-miR-125b-5p|rno-miR-125a-5p

NM_001126099	3	rno-miR-125b-5p|rno-miR-125a-5p|rno-miR-320-3p
NM_181377	2	rno-miR-125b-5p|rno-miR-125a-5p
NM_001009663	2	rno-miR-125b-5p|rno-miR-125a-5p
NM_199117	4	rno-miR-125b-5p|rno-miR-125a-5p|rno-miR-181a-5p|rno-miR-30c-5p
NM_001012125	2	rno-miR-125b-5p|rno-miR-125a-5p
NM_001107652	3	rno-miR-125b-5p|rno-miR-125a-5p|rno-miR-30c-5p
NM_001013996	2	rno-miR-125b-5p|rno-miR-125a-5p
NM_001134629	4	rno-miR-125b-5p|rno-miR-125a-5p|rno-miR-378a-3p|rno-miR-30c-5p
NM_031830	4	rno-miR-125b-5p|rno-miR-103-3p|rno-miR-125a-5p|rno-miR-107-3p
NM_001109470	4	rno-miR-125b-5p|rno-miR-125a-5p|rno-miR-27b-3p|rno-miR-27a-3p
NM_001109434	3	rno-miR-125b-5p|rno-miR-125a-5p|rno-miR-320-3p
NM_001108166	2	rno-miR-125b-5p|rno-miR-125a-5p
NM_001039016	4	rno-miR-125b-5p|rno-miR-103-3p|rno-miR-125a-5p|rno-miR-107-3p
NM_001013221	2	rno-miR-125b-5p|rno-miR-125a-5p
NM_001106645	2	rno-miR-125b-5p|rno-miR-125a-5p
NM_031820	2	rno-miR-125b-5p|rno-miR-125a-5p
NM_053400	2	rno-miR-125b-5p|rno-miR-125a-5p
NM_053801	3	rno-miR-125b-5p|rno-miR-125a-5p|rno-miR-30c-5p
NM_001106016	2	rno-miR-125b-5p|rno-miR-125a-5p
NM_001106572	3	rno-miR-125b-5p|rno-miR-125a-5p|rno-miR-22-3p
NM_013076	2	rno-miR-125b-5p|rno-miR-125a-5p
NM_001012101	2	rno-miR-125b-5p|rno-miR-125a-5p
NM_001014772	2	rno-miR-125b-5p|rno-miR-125a-5p
NM_001135855	3	rno-miR-125b-5p|rno-miR-125a-5p|rno-miR-30c-5p
NM_001107121	2	rno-miR-125b-5p|rno-miR-125a-5p
NM_001009275	2	rno-miR-125b-5p|rno-miR-125a-5p
NM_182825	3	rno-miR-125b-5p|rno-miR-125a-5p|rno-miR-30c-5p
NM_001015036	4	rno-miR-125b-5p|rno-miR-125a-5p|rno-miR-17-5p|rno-miR-93-5p
NM_177933	4	rno-miR-125b-5p|rno-miR-125a-5p|rno-miR-205|rno-miR-181a-5p
NM_001135089	4	rno-miR-125b-5p|rno-miR-125a-5p|rno-miR-27b-3p|rno-miR-27a-3p

NM_031522	2	rno-miR-125b-5p|rno-miR-125a-5p
NM_001024762	2	rno-miR-125b-5p|rno-miR-125a-5p
NM_001107968	2	rno-miR-125b-5p|rno-miR-125a-5p
NM_139258	5	rno-miR-125b-5p|rno-miR-125a-5p|rno-miR-320-3p|rno-miR-205|rno-miR-29a-3p
NM_001106956	2	rno-miR-125b-5p|rno-miR-125a-5p
NM_001303537	4	rno-miR-125b-5p|rno-miR-125a-5p|rno-miR-17-5p|rno-miR-93-5p
NM_001191621	2	rno-miR-125b-5p|rno-miR-125a-5p
NM_175758	2	rno-miR-125b-5p|rno-miR-125a-5p
NM_001100890	3	rno-miR-125b-5p|rno-miR-125a-5p|rno-miR-378a-3p
NM_001009703	2	rno-miR-125b-5p|rno-miR-125a-5p
NM_001108516	2	rno-miR-125b-5p|rno-miR-125a-5p
NM_080780	2	rno-miR-125b-5p|rno-miR-125a-5p
NM_031342	2	rno-miR-125b-5p|rno-miR-125a-5p
NM_001106805	3	rno-miR-125b-5p|rno-miR-125a-5p|rno-miR-320-3p
NM_001126298	2	rno-miR-125b-5p|rno-miR-125a-5p
NM_001013984	2	rno-miR-125b-5p|rno-miR-125a-5p
NM_001106675	3	rno-miR-125b-5p|rno-miR-125a-5p|rno-miR-320-3p
NM_053984	2	rno-miR-125b-5p|rno-miR-125a-5p
NM_001108203	2	rno-miR-125b-5p|rno-miR-125a-5p
NM_001107701	2	rno-miR-125b-5p|rno-miR-125a-5p
NM_001029917	2	rno-miR-125b-5p|rno-miR-125a-5p
NM_001108965	2	rno-miR-125b-5p|rno-miR-125a-5p
NM_001109467	2	rno-miR-125b-5p|rno-miR-125a-5p
NM_001107225	2	rno-miR-125b-5p|rno-miR-125a-5p
NM_031653	2	rno-miR-125b-5p|rno-miR-125a-5p
NM_001107479	2	rno-miR-125b-5p|rno-miR-125a-5p
NM_031107	5	rno-miR-125b-5p|rno-miR-125a-5p|rno-miR-378a-3p|rno-miR-17-5p|rno-miR-93-5p
NM_001106833	2	rno-miR-125b-5p|rno-miR-125a-5p

NM_001271309	2	rno-miR-125b-5p|rno-miR-125a-5p
NM_001109141	4	rno-miR-125b-5p|rno-miR-125a-5p|rno-miR-29a-3p|rno-miR-200b-3p
NM_001134884	2	rno-miR-125b-5p|rno-miR-125a-5p
NM_001024969	6	rno-miR-125b-5p|rno-miR-125a-5p|rno-miR-200b-3p|rno-miR-30c-5p|rno-miR-17-5p|rno-miR-93-5p
NM_001109407	2	rno-miR-125b-5p|rno-miR-125a-5p
NM_001009455	2	rno-miR-125b-5p|rno-miR-125a-5p
NM_001194951	2	rno-miR-125b-5p|rno-miR-125a-5p
NM_012758	2	rno-miR-125b-5p|rno-miR-125a-5p
NM_053294	2	rno-miR-125b-5p|rno-miR-125a-5p
NM_031647	4	rno-miR-125b-5p|rno-miR-125a-5p|rno-miR-17-5p|rno-miR-93-5p
NM_181823	2	rno-miR-125b-5p|rno-miR-125a-5p
NM_001033860	3	rno-miR-125b-5p|rno-miR-125a-5p|rno-miR-30c-5p
NM_001109576	2	rno-miR-125b-5p|rno-miR-125a-5p
NM_001109528	4	rno-miR-125b-5p|rno-miR-103-3p|rno-miR-125a-5p|rno-miR-107-3p
NM_012668	2	rno-miR-125b-5p|rno-miR-125a-5p
NM_001108384	2	rno-miR-125b-5p|rno-miR-125a-5p
NM_001106456	3	rno-miR-125b-5p|rno-miR-125a-5p|rno-miR-152-3p
NM_147141	3	rno-miR-125b-5p|rno-miR-125a-5p|rno-miR-181a-5p
NM_032061	6	rno-miR-125b-5p|rno-miR-103-3p|rno-miR-125a-5p|rno-miR-107-3p|rno-miR-17-5p|rno-miR-93-5p
NM_001107600	2	rno-miR-125b-5p|rno-miR-125a-5p
NM_001009647	2	rno-miR-125b-5p|rno-miR-125a-5p
NM_001017386	2	rno-miR-125b-5p|rno-miR-125a-5p
NM_001144850	2	rno-miR-125b-5p|rno-miR-125a-5p
NM_001127599	2	rno-miR-125b-5p|rno-miR-125a-5p
NM_001114599	5	rno-miR-125b-5p|rno-miR-125a-5p|rno-miR-27b-3p|rno-miR-27a-3p|rno-miR-152-3p
NM_001017513	2	rno-miR-125b-5p|rno-miR-125a-5p
NM_139087	2	rno-miR-125b-5p|rno-miR-125a-5p

NM_001101000	3	rno-miR-125b-5p|rno-miR-125a-5p|rno-miR-151-5p
NM_001170462	5	rno-miR-125b-5p|rno-miR-125a-5p|rno-miR-378a-3p|rno-miR-17-5p|rno-miR-93-5p
NM_001191794	2	rno-miR-125b-5p|rno-miR-125a-5p
NM_001244784	2	rno-miR-125b-5p|rno-miR-125a-5p
NM_001107442	2	rno-miR-125b-5p|rno-miR-125a-5p
NM_001009541	3	rno-miR-125b-5p|rno-miR-125a-5p|rno-miR-30c-5p
NM_031778	3	rno-miR-125b-5p|rno-miR-125a-5p|rno-miR-320-3p
NM_022294	3	rno-miR-125b-5p|rno-miR-125a-5p|rno-miR-181a-5p
NM_017129	2	rno-miR-125b-5p|rno-miR-125a-5p
NM_001108598	2	rno-miR-125b-5p|rno-miR-125a-5p
NM_001033862	3	rno-miR-125b-5p|rno-miR-125a-5p|rno-miR-30c-5p
NM_001034855	2	rno-miR-125b-5p|rno-miR-125a-5p
NM_001107601	2	rno-miR-125b-5p|rno-miR-125a-5p
NM_001108573	2	rno-miR-125b-5p|rno-miR-125a-5p
NM_001013136	3	rno-miR-125b-5p|rno-miR-125a-5p|rno-miR-22-3p
NM_001108698	2	rno-miR-125b-5p|rno-miR-125a-5p
NM_001106378	5	rno-miR-125b-5p|rno-miR-3473|rno-miR-125a-5p|rno-miR-27b-3p|rno-miR-27a-3p
NM_031755	3	rno-miR-125b-5p|rno-miR-125a-5p|rno-miR-30c-5p
NM_001109426	2	rno-miR-125b-5p|rno-miR-125a-5p
NM_001017383	2	rno-miR-125b-5p|rno-miR-125a-5p
NM_001107469	2	rno-miR-125b-5p|rno-miR-125a-5p
NM_080580	2	rno-miR-125b-5p|rno-miR-125a-5p
NM_001134413	4	rno-miR-125b-5p|rno-miR-125a-5p|rno-miR-27b-3p|rno-miR-27a-3p
NM_053503	5	rno-miR-125b-5p|rno-miR-125a-5p|rno-miR-17-5p|rno-miR-152-3p|rno-miR-93-5p
NM_001106899	4	rno-miR-125b-5p|rno-miR-125a-5p|rno-miR-27b-3p|rno-miR-27a-3p
NM_001105721	2	rno-miR-125b-5p|rno-miR-125a-5p
NM_053938	2	rno-miR-125b-5p|rno-miR-125a-5p

NM_001172137	2	rno-miR-125b-5p|rno-miR-125a-5p
NM_031796	4	rno-miR-125b-5p|rno-miR-125a-5p|rno-miR-27b-3p|rno-miR-27a-3p
NM_138898	2	rno-miR-125b-5p|rno-miR-125a-5p
NM_001106977	6	rno-miR-125b-5p|rno-miR-125a-5p|rno-miR-22-3p|rno-miR-29a-3p|rno-miR-31a-5p|rno-miR-30c-5p
NM_001013034	3	rno-miR-125b-5p|rno-miR-125a-5p|rno-miR-22-3p
NM_001108615	5	rno-miR-125b-5p|rno-miR-125a-5p|rno-miR-27b-3p|rno-miR-27a-3p|rno-miR-200b-3p
NM_001109033	3	rno-miR-125b-5p|rno-miR-125a-5p|rno-miR-320-3p
NM_001277069	3	rno-miR-125b-5p|rno-miR-125a-5p|rno-miR-200b-3p
NM_001107884	2	rno-miR-125b-5p|rno-miR-125a-5p
NM_017058	2	rno-miR-125b-5p|rno-miR-125a-5p
NM_001044234	2	rno-miR-125b-5p|rno-miR-125a-5p
NM_001014256	3	rno-miR-125b-5p|rno-miR-125a-5p|rno-miR-199a-3p
NM_001108249	2	rno-miR-125b-5p|rno-miR-125a-5p
NM_001108143	3	rno-miR-125b-5p|rno-miR-125a-5p|rno-miR-30c-5p
NM_001105767	2	rno-miR-125b-5p|rno-miR-125a-5p
NM_001013225	3	rno-miR-125b-5p|rno-miR-125a-5p|rno-miR-29a-3p
NM_001191838	2	rno-miR-125b-5p|rno-miR-125a-5p
NM_001173972	7	rno-miR-125b-5p|rno-miR-125a-5p|rno-miR-320-3p|rno-miR-199a-3p|rno-miR-27b-3p|rno-miR-27a-3p|rno-miR-30c-5p
NM_001012109	2	rno-miR-125b-5p|rno-miR-125a-5p
NM_001037192	2	rno-miR-125b-5p|rno-miR-125a-5p
NM_012637	2	rno-miR-125b-5p|rno-miR-125a-5p
NM_017108	2	rno-miR-125b-5p|rno-miR-125a-5p
NM_031236	2	rno-miR-125b-5p|rno-miR-125a-5p
NM_001170429	2	rno-miR-125b-5p|rno-miR-125a-5p
NM_001108663	2	rno-miR-125b-5p|rno-miR-125a-5p
NM_001024285	4	rno-miR-125b-5p|rno-miR-125a-5p|rno-miR-22-3p|rno-miR-152-3p
NM_001270800	4	rno-miR-125b-5p|rno-miR-103-3p|rno-miR-125a-5p|rno-miR-107-3p

NM_001107164	2	rno-miR-125b-5p|rno-miR-125a-5p
NM_057198	2	rno-miR-125b-5p|rno-miR-125a-5p
NM_017205	3	rno-miR-125b-5p|rno-miR-125a-5p|rno-miR-31a-5p
NM_001109236	2	rno-miR-125b-5p|rno-miR-125a-5p
NM_019189	5	rno-miR-125b-5p|rno-miR-125a-5p|rno-miR-320-3p|rno-miR-27b-3p|rno-miR-27a-3p
NM_001009709	2	rno-miR-125b-5p|rno-miR-125a-5p
NM_001039024	2	rno-miR-125b-5p|rno-miR-125a-5p
NM_001100648	1	rno-miR-125b-5p
NM_012720	3	rno-miR-125b-5p|rno-miR-125a-5p|rno-miR-31a-5p
NM_178092	2	rno-miR-125b-5p|rno-miR-125a-5p
NM_001108890	4	rno-miR-125b-5p|rno-miR-125a-5p|rno-miR-27b-3p|rno-miR-27a-3p
NM_001044236	4	rno-miR-125b-5p|rno-miR-125a-5p|rno-miR-205|rno-miR-29a-3p
NM_001033079	6	rno-miR-125b-5p|rno-miR-125a-5p|rno-miR-27b-3p|rno-miR-22-3p|rno-miR-27a-3p|rno-miR-200b-3p
NM_001329136	3	rno-miR-125b-5p|rno-miR-125a-5p|rno-miR-31a-5p
NM_001012030	2	rno-miR-125b-5p|rno-miR-125a-5p
NM_022512	2	rno-miR-125b-5p|rno-miR-125a-5p
NM_001195245	2	rno-miR-125b-5p|rno-miR-125a-5p
NM_001108468	2	rno-miR-125b-5p|rno-miR-125a-5p
NM_001170563	2	rno-miR-125b-5p|rno-miR-125a-5p
NM_201635	3	rno-miR-125b-5p|rno-miR-125a-5p|rno-miR-30c-5p
NM_001127600	3	rno-miR-125b-5p|rno-miR-125a-5p|rno-miR-30c-5p
NM_001115045	3	rno-miR-125b-5p|rno-miR-125a-5p|rno-miR-200b-3p
NM_024001	2	rno-miR-125b-5p|rno-miR-125a-5p
NM_199109	3	rno-miR-125b-5p|rno-miR-125a-5p|rno-miR-22-3p
NM_001301297	2	rno-miR-125b-5p|rno-miR-125a-5p
NM_001108674	2	rno-miR-125b-5p|rno-miR-125a-5p
NM_001108687	2	rno-miR-125b-5p|rno-miR-125a-5p
NM_001107292	4	rno-miR-125b-5p|rno-miR-125a-5p|rno-miR-200b-3p|rno-miR-31a-5p

NM_022693	2	rno-miR-125b-5p|rno-miR-125a-5p
NM_001127581	3	rno-miR-125b-5p|rno-miR-125a-5p|rno-miR-29a-3p
NM_001013150	2	rno-miR-125b-5p|rno-miR-125a-5p
NM_013178	2	rno-miR-125b-5p|rno-miR-125a-5p
NM_001127602	4	rno-miR-125b-5p|rno-miR-125a-5p|rno-miR-378a-3p|rno-miR-152-3p
NM_022404	5	rno-miR-125b-5p|rno-miR-103-3p|rno-miR-125a-5p|rno-miR-107-3p|rno-miR-152-3p
NM_001100788	2	rno-miR-125b-5p|rno-miR-125a-5p
NM_001192018	2	rno-miR-125b-5p|rno-miR-125a-5p
NM_024000	2	rno-miR-125b-5p|rno-miR-125a-5p
NM_001003975	2	rno-miR-125b-5p|rno-miR-125a-5p
NM_001108215	4	rno-miR-125b-5p|rno-miR-125a-5p|rno-miR-199a-3p|rno-miR-30c-5p
NM_001025018	4	rno-miR-125b-5p|rno-miR-125a-5p|rno-miR-27b-3p|rno-miR-27a-3p
NM_001109892	2	rno-miR-125b-5p|rno-miR-125a-5p
NM_031055	2	rno-miR-125b-5p|rno-miR-125a-5p
NM_001008377	2	rno-miR-125b-5p|rno-miR-125a-5p
NM_001105817	2	rno-miR-125b-5p|rno-miR-125a-5p
NM_001106940	3	rno-miR-125b-5p|rno-miR-125a-5p|rno-miR-199a-3p
NM_001109196	2	rno-miR-125b-5p|rno-miR-125a-5p
NM_001130013	2	rno-miR-125b-5p|rno-miR-125a-5p
NM_212508	2	rno-miR-125b-5p|rno-miR-125a-5p
NM_022251	5	rno-miR-125b-5p|rno-miR-125a-5p|rno-miR-320-3p|rno-miR-27b-3p|rno-miR-27a-3p
NM_182738	2	rno-miR-125b-5p|rno-miR-125a-5p
NM_001098724	2	rno-miR-125b-5p|rno-miR-125a-5p
NM_001009690	2	rno-miR-125b-5p|rno-miR-125a-5p
NM_001024244	2	rno-miR-125b-5p|rno-miR-125a-5p
NM_001005883	3	rno-miR-125b-5p|rno-miR-125a-5p|rno-miR-181a-5p
NM_001039044	3	rno-miR-125b-5p|rno-miR-3473|rno-miR-125a-5p
NM_001007658	3	rno-miR-125b-5p|rno-miR-125a-5p|rno-miR-151-5p

NM_001037196	2	rno-miR-125b-5p|rno-miR-125a-5p
NM_001144840	2	rno-miR-125b-5p|rno-miR-125a-5p
NM_022389	2	rno-miR-125b-5p|rno-miR-125a-5p
NM_001135088	4	rno-miR-125b-5p|rno-miR-125a-5p|rno-miR-27b-3p|rno-miR-27a-3p
NM_001007705	2	rno-miR-125b-5p|rno-miR-125a-5p
NM_001107754	2	rno-miR-125b-5p|rno-miR-125a-5p
NM_001100739	3	rno-miR-125b-5p|rno-miR-125a-5p|rno-miR-200b-3p
NM_001105853	2	rno-miR-125b-5p|rno-miR-125a-5p
NM_001134755	2	rno-miR-125b-5p|rno-miR-125a-5p
NM_001033709	2	rno-miR-125b-5p|rno-miR-125a-5p
NM_001024780	3	rno-miR-125b-5p|rno-miR-125a-5p|rno-miR-29a-3p
NM_024401	3	rno-miR-125b-5p|rno-miR-3473|rno-miR-125a-5p
NM_001014135	3	rno-miR-125b-5p|rno-miR-125a-5p|rno-miR-320-3p
NM_012747	2	rno-miR-125b-5p|rno-miR-125a-5p
NM_031012	2	rno-miR-125b-5p|rno-miR-125a-5p
NM_001079705	4	rno-miR-125b-5p|rno-miR-125a-5p|rno-miR-17-5p|rno-miR-93-5p
NM_013185	2	rno-miR-125b-5p|rno-miR-125a-5p
NM_001130565	2	rno-miR-125b-5p|rno-miR-125a-5p
NM_053857	2	rno-miR-125b-5p|rno-miR-125a-5p
NM_172018	2	rno-miR-125b-5p|rno-miR-125a-5p
NM_001106162	4	rno-miR-125b-5p|rno-miR-125a-5p|rno-miR-199a-3p|rno-miR-181a-5p
NM_001025748	2	rno-miR-125b-5p|rno-miR-125a-5p
NM_001082410	2	rno-miR-125b-5p|rno-miR-125a-5p
NM_012863	2	rno-miR-125b-5p|rno-miR-125a-5p
NM_001191752	2	rno-miR-125b-5p|rno-miR-125a-5p
NM_001107572	2	rno-miR-125b-5p|rno-miR-125a-5p
NM_001291359	2	rno-miR-125b-5p|rno-miR-125a-5p
NM_001017494	3	rno-miR-125b-5p|rno-miR-125a-5p|rno-miR-320-3p
NM_001105749	2	rno-miR-125b-5p|rno-miR-125a-5p
NM_133393	2	rno-miR-125b-5p|rno-miR-125a-5p

NM_001109027	2	rno-miR-125b-5p|rno-miR-125a-5p
NM_001013980	2	rno-miR-125b-5p|rno-miR-125a-5p
NM_001008511	2	rno-miR-125b-5p|rno-miR-125a-5p
NM_001109243	4	rno-miR-125b-5p|rno-miR-103-3p|rno-miR-125a-5p|rno-miR-107-3p
NM_001109302	3	rno-miR-125b-5p|rno-miR-125a-5p|rno-miR-200b-3p
NM_147205	2	rno-miR-125b-5p|rno-miR-125a-5p
NM_001013224	3	rno-miR-125b-5p|rno-miR-125a-5p|rno-miR-181a-5p
NM_053805	3	rno-miR-125b-5p|rno-miR-125a-5p|rno-miR-30c-5p
NM_001033861	3	rno-miR-125b-5p|rno-miR-125a-5p|rno-miR-30c-5p
NM_053552	2	rno-miR-125b-5p|rno-miR-125a-5p
NM_001109899	2	rno-miR-125b-5p|rno-miR-125a-5p
NM_001005902	2	rno-miR-125b-5p|rno-miR-125a-5p
NM_053690	2	rno-miR-125b-5p|rno-miR-125a-5p
NM_001271369	2	rno-miR-125b-5p|rno-miR-125a-5p
NM_001007012	2	rno-miR-125b-5p|rno-miR-125a-5p
NM_147140	2	rno-miR-125b-5p|rno-miR-125a-5p
NM_001030026	3	rno-miR-125b-5p|rno-miR-125a-5p|rno-miR-29a-3p
NM_138539	2	rno-miR-125b-5p|rno-miR-125a-5p
NM_001106108	5	rno-miR-125b-5p|rno-miR-125a-5p|rno-miR-27b-3p|rno-miR-27a-3p|rno-miR-30c-5p
NM_053340	2	rno-miR-125b-5p|rno-miR-125a-5p
NM_031594	4	rno-miR-125b-5p|rno-miR-125a-5p|rno-miR-17-5p|rno-miR-93-5p
NM_001107482	2	rno-miR-125b-5p|rno-miR-125a-5p
NM_001013154	3	rno-miR-125b-5p|rno-miR-125a-5p|rno-miR-30c-5p
NM_053944	2	rno-miR-125b-5p|rno-miR-125a-5p
NM_001191791	4	rno-miR-125b-5p|rno-miR-125a-5p|rno-miR-200b-3p|rno-miR-30c-5p
NM_019254	2	rno-miR-103-3p|rno-miR-107-3p
NM_001106455	5	rno-miR-103-3p|rno-miR-107-3p|rno-miR-200b-3p|rno-miR-31a-5p|rno-miR-30c-5p
NM_001106527	4	rno-miR-103-3p|rno-miR-107-3p|rno-miR-27b-3p|rno-miR-27a-3p

NM_031806	6	rno-miR-103-3p|rno-miR-107-3p|rno-miR-27b-3p|rno-miR-27a-3p|rno-miR-17-5p|rno-miR-93-5p
NM_080787	3	rno-miR-103-3p|rno-miR-107-3p|rno-miR-200b-3p
NM_001309381	2	rno-miR-103-3p|rno-miR-107-3p
NM_212523	4	rno-miR-103-3p|rno-miR-107-3p|rno-miR-17-5p|rno-miR-93-5p
NM_001109979	2	rno-miR-103-3p|rno-miR-107-3p
NM_183333	2	rno-miR-103-3p|rno-miR-107-3p
NM_001033067	2	rno-miR-103-3p|rno-miR-107-3p
NM_001170459	2	rno-miR-103-3p|rno-miR-107-3p
NM_133611	3	rno-miR-103-3p|rno-miR-107-3p|rno-miR-200b-3p
NM_001013120	5	rno-miR-103-3p|rno-miR-107-3p|rno-miR-199a-3p|rno-miR-181a-5p|rno-miR-31a-5p
NM_001007739	3	rno-miR-103-3p|rno-miR-107-3p|rno-miR-378a-3p
NM_001108768	2	rno-miR-103-3p|rno-miR-107-3p
NM_001109071	2	rno-miR-103-3p|rno-miR-107-3p
NM_001107283	6	rno-miR-103-3p|rno-miR-107-3p|rno-miR-27b-3p|rno-miR-27a-3p|rno-miR-200b-3p|rno-miR-181a-5p
NM_001011898	2	rno-miR-103-3p|rno-miR-107-3p
NM_001127568	3	rno-miR-103-3p|rno-miR-107-3p|rno-miR-200b-3p
NM_017231	4	rno-miR-103-3p|rno-miR-107-3p|rno-miR-17-5p|rno-miR-93-5p
NM_017227	2	rno-miR-103-3p|rno-miR-107-3p
NM_001309297	2	rno-miR-103-3p|rno-miR-107-3p
NM_001030032	3	rno-miR-103-3p|rno-miR-107-3p|rno-miR-205
NM_001107891	2	rno-miR-103-3p|rno-miR-107-3p
NM_001007727	2	rno-miR-103-3p|rno-miR-107-3p
NM_022926	4	rno-miR-103-3p|rno-miR-107-3p|rno-miR-27b-3p|rno-miR-27a-3p
NM_001083336	2	rno-miR-103-3p|rno-miR-107-3p
NM_001107514	5	rno-miR-103-3p|rno-miR-107-3p|rno-miR-181a-5p|rno-miR-17-5p|rno-miR-93-5p
NM_019125	3	rno-miR-103-3p|rno-miR-107-3p|rno-miR-181a-5p

NM_012878	2	rno-miR-103-3p|rno-miR-107-3p
NM_019319	4	rno-miR-103-3p|rno-miR-107-3p|rno-miR-17-5p|rno-miR-93-5p
NM_001270597	3	rno-miR-103-3p|rno-miR-107-3p|rno-miR-15b-5p
NM_001012076	3	rno-miR-103-3p|rno-miR-107-3p|rno-miR-199a-3p
NM_001008312	2	rno-miR-103-3p|rno-miR-107-3p
NM_019210	2	rno-miR-103-3p|rno-miR-107-3p
NM_023102	3	rno-miR-103-3p|rno-miR-107-3p|rno-miR-378a-3p
NM_001044283	2	rno-miR-103-3p|rno-miR-107-3p
NM_001191562	4	rno-miR-103-3p|rno-miR-107-3p|rno-miR-205|rno-miR-181a-5p
NM_001108755	2	rno-miR-103-3p|rno-miR-107-3p
NM_001308636	2	rno-miR-103-3p|rno-miR-107-3p
NM_001024261	2	rno-miR-103-3p|rno-miR-107-3p
NM_001033870	3	rno-miR-103-3p|rno-miR-107-3p|rno-miR-378a-3p
NM_001107520	2	rno-miR-103-3p|rno-miR-107-3p
NM_001270596	3	rno-miR-103-3p|rno-miR-107-3p|rno-miR-15b-5p
NM_133651	2	rno-miR-103-3p|rno-miR-107-3p
NM_001106614	5	rno-miR-103-3p|rno-miR-107-3p|rno-miR-27b-3p|rno-miR-27a-3p|rno-miR-30c-5p
NM_001271385	2	rno-miR-103-3p|rno-miR-107-3p
NM_053557	2	rno-miR-103-3p|rno-miR-107-3p
NM_001004277	2	rno-miR-103-3p|rno-miR-107-3p
NM_001107735	2	rno-miR-103-3p|rno-miR-107-3p
NM_019218	3	rno-miR-103-3p|rno-miR-107-3p|rno-miR-30c-5p
NM_133427	3	rno-miR-103-3p|rno-miR-107-3p|rno-miR-152-3p
NM_013090	2	rno-miR-103-3p|rno-miR-107-3p
NM_001035234	2	rno-miR-103-3p|rno-miR-107-3p
NM_133320	5	rno-miR-103-3p|rno-miR-107-3p|rno-miR-30c-5p|rno-miR-17-5p|rno-miR-93-5p
NM_001139507	2	rno-miR-103-3p|rno-miR-107-3p
NM_012669	2	rno-miR-103-3p|rno-miR-107-3p

NM_054011	4	rno-miR-103-3p|rno-miR-107-3p|rno-miR-17-5p|rno-miR-93-5p
NM_001191861	3	rno-miR-103-3p|rno-miR-107-3p|rno-miR-320-3p
NM_001127573	2	rno-miR-103-3p|rno-miR-107-3p
NM_001013933	2	rno-miR-103-3p|rno-miR-107-3p
NM_001106521	2	rno-miR-103-3p|rno-miR-107-3p
NM_001106367	4	rno-miR-103-3p|rno-miR-107-3p|rno-miR-200b-3p|rno-miR-30c-5p
NM_001007759	2	rno-miR-103-3p|rno-miR-107-3p
NM_001191769	2	rno-miR-103-3p|rno-miR-107-3p
NM_053481	4	rno-miR-103-3p|rno-miR-107-3p|rno-miR-199a-3p|rno-miR-200b-3p
NM_001106841	2	rno-miR-103-3p|rno-miR-107-3p
NM_001108001	2	rno-miR-103-3p|rno-miR-107-3p
NM_001039009	4	rno-miR-103-3p|rno-miR-107-3p|rno-miR-29a-3p|rno-miR-30c-5p
NM_001107886	4	rno-miR-103-3p|rno-miR-107-3p|rno-miR-320-3p|rno-miR-29a-3p
NM_031805	2	rno-miR-103-3p|rno-miR-107-3p
NM_001007625	3	rno-miR-103-3p|rno-miR-107-3p|rno-miR-99b-5p
NM_001115048	2	rno-miR-103-3p|rno-miR-107-3p
NM_145682	2	rno-miR-103-3p|rno-miR-107-3p
NM_017023	2	rno-miR-103-3p|rno-miR-107-3p
NM_012673	2	rno-miR-103-3p|rno-miR-107-3p
NM_001309299	2	rno-miR-103-3p|rno-miR-107-3p
NM_001025020	2	rno-miR-103-3p|rno-miR-107-3p
NM_080411	2	rno-miR-103-3p|rno-miR-107-3p
NM_001109175	2	rno-miR-103-3p|rno-miR-107-3p
NM_001108595	4	rno-miR-103-3p|rno-miR-107-3p|rno-miR-320-3p|rno-miR-22-3p
NM_001134955	3	rno-miR-103-3p|rno-miR-107-3p|rno-miR-29a-3p
NM_001012160	2	rno-miR-103-3p|rno-miR-107-3p
NM_001108938	2	rno-miR-103-3p|rno-miR-107-3p
NM_171983	2	rno-miR-103-3p|rno-miR-107-3p
NM_001012235	5	rno-miR-103-3p|rno-miR-107-3p|rno-miR-27b-3p|rno-miR-27a-3p|rno-miR-30c-5p

NM_001170436	2	rno-miR-103-3p|rno-miR-107-3p
NM_001108635	2	rno-miR-103-3p|rno-miR-107-3p
NM_017041	3	rno-miR-103-3p|rno-miR-107-3p|rno-miR-30c-5p
NM_001276491	2	rno-miR-103-3p|rno-miR-107-3p
NM_001276470	3	rno-miR-103-3p|rno-miR-107-3p|rno-miR-30c-5p
NM_031662	2	rno-miR-103-3p|rno-miR-107-3p
NM_001107076	2	rno-miR-103-3p|rno-miR-107-3p
NM_001009966	3	rno-miR-103-3p|rno-miR-107-3p|rno-miR-15b-5p
NM_001013433	2	rno-miR-103-3p|rno-miR-107-3p
NM_001105778	3	rno-miR-103-3p|rno-miR-107-3p|rno-miR-181a-5p
NM_012880	2	rno-miR-103-3p|rno-miR-107-3p
NM_001309298	2	rno-miR-103-3p|rno-miR-107-3p
NM_001173429	2	rno-miR-103-3p|rno-miR-107-3p
NM_001004203	4	rno-miR-103-3p|rno-miR-107-3p|rno-miR-17-5p|rno-miR-93-5p
NM_001309301	2	rno-miR-103-3p|rno-miR-107-3p
NM_001024984	3	rno-miR-103-3p|rno-miR-107-3p|rno-miR-152-3p
NM_001109072	2	rno-miR-103-3p|rno-miR-107-3p
NM_001107890	6	rno-miR-103-3p|rno-miR-107-3p|rno-miR-29a-3p|rno-miR-31a-5p|rno-miR-17-5p|rno-miR-93-5p
NM_001100778	5	rno-miR-103-3p|rno-miR-107-3p|rno-miR-320-3p|rno-miR-17-5p|rno-miR-93-5p
NM_001107290	2	rno-miR-103-3p|rno-miR-107-3p
NM_001170475	2	rno-miR-103-3p|rno-miR-107-3p
NM_001004276	2	rno-miR-103-3p|rno-miR-107-3p
NM_173105	4	rno-miR-103-3p|rno-miR-107-3p|rno-miR-27b-3p|rno-miR-27a-3p
NM_001173354	5	rno-miR-103-3p|rno-miR-107-3p|rno-miR-29a-3p|rno-miR-17-5p|rno-miR-93-5p
NM_001033967	4	rno-miR-103-3p|rno-miR-107-3p|rno-miR-27b-3p|rno-miR-27a-3p
NM_017235	2	rno-miR-103-3p|rno-miR-107-3p
NM_001108498	4	rno-miR-103-3p|rno-miR-107-3p|rno-miR-17-5p|rno-miR-93-5p

NM_001012275	3	rno-miR-103-3p|rno-miR-107-3p|rno-miR-181a-5p
NM_001100894	3	rno-miR-103-3p|rno-miR-107-3p|rno-miR-152-3p
NM_001047847	2	rno-miR-103-3p|rno-miR-107-3p
NM_053464	2	rno-miR-103-3p|rno-miR-107-3p
NM_001025400	2	rno-miR-103-3p|rno-miR-107-3p
NM_001108165	3	rno-miR-103-3p|rno-miR-107-3p|rno-miR-181a-5p
NM_001109380	2	rno-miR-103-3p|rno-miR-107-3p
NM_001025151	2	rno-miR-103-3p|rno-miR-107-3p
NM_001108424	2	rno-miR-103-3p|rno-miR-107-3p
NM_001108180	4	rno-miR-103-3p|rno-miR-107-3p|rno-miR-205|rno-miR-29a-3p
NM_001109444	3	rno-miR-103-3p|rno-miR-3473|rno-miR-107-3p
NM_130822	4	rno-miR-103-3p|rno-miR-107-3p|rno-miR-27b-3p|rno-miR-27a-3p
NM_080584	2	rno-miR-103-3p|rno-miR-107-3p
NM_001105865	4	rno-miR-103-3p|rno-miR-107-3p|rno-miR-205|rno-miR-152-3p
NM_001191786	4	rno-miR-103-3p|rno-miR-107-3p|rno-miR-27b-3p|rno-miR-27a-3p
NM_001025757	4	rno-miR-103-3p|rno-miR-107-3p|rno-miR-27b-3p|rno-miR-27a-3p
NM_001108690	3	rno-miR-103-3p|rno-miR-107-3p|rno-miR-199a-3p
NM_001034163	2	rno-miR-103-3p|rno-miR-107-3p
NM_001108617	5	rno-miR-103-3p|rno-miR-107-3p|rno-miR-27b-3p|rno-miR-22-3p|rno-miR-27a-3p
NM_022243	2	rno-miR-103-3p|rno-miR-107-3p
NM_001100827	2	rno-miR-103-3p|rno-miR-107-3p
NM_031556	2	rno-miR-103-3p|rno-miR-107-3p
NM_001014196	2	rno-miR-103-3p|rno-miR-107-3p
NM_001024253	2	rno-miR-103-3p|rno-miR-107-3p
NM_001278475	3	rno-miR-103-3p|rno-miR-107-3p|rno-miR-320-3p
NM_001001799	3	rno-miR-103-3p|rno-miR-3473|rno-miR-107-3p
NM_053360	3	rno-miR-103-3p|rno-miR-107-3p|rno-miR-22-3p
NM_001024750	2	rno-miR-103-3p|rno-miR-107-3p
NM_012919	3	rno-miR-103-3p|rno-miR-107-3p|rno-miR-152-3p

NM_001014102	2	rno-miR-103-3p|rno-miR-107-3p
NM_001107334	3	rno-miR-103-3p|rno-miR-107-3p|rno-miR-205
NM_001017445	2	rno-miR-103-3p|rno-miR-107-3p
NM_001100576	2	rno-miR-103-3p|rno-miR-107-3p
NM_001106789	2	rno-miR-103-3p|rno-miR-107-3p
NM_001108985	1	rno-let-7i-5p
NM_001106560	1	rno-let-7i-5p
NM_001013054	1	rno-miR-3473
NM_001108441	1	rno-miR-3473
NM_001105952	2	rno-miR-3473|rno-miR-22-3p
NM_001007620	1	rno-miR-3473
NM_001025692	1	rno-miR-3473
NM_033096	4	rno-miR-3473|rno-miR-320-3p|rno-miR-200b-3p|rno-miR-181a-5p
NM_013155	6	rno-miR-3473|rno-miR-320-3p|rno-miR-199a-3p|rno-miR-200b-3p|rno-miR-17-5p|rno-miR-93-5p
NM_017348	1	rno-miR-3473
NM_001107476	1	rno-miR-3473
NM_022934	1	rno-miR-3473
NM_023990	1	rno-miR-3473
NM_001106807	1	rno-miR-3473
NM_031134	1	rno-miR-3473
NM_013002	2	rno-miR-3473|rno-miR-151-5p
NM_012651	1	rno-miR-3473
NM_134358	1	rno-miR-3473
NM_001107982	4	rno-miR-3473|rno-miR-27b-3p|rno-miR-27a-3p|rno-miR-200b-3p
NM_001106005	3	rno-miR-3473|rno-miR-320-3p|rno-miR-30c-5p
NM_031969	2	rno-miR-3473|rno-miR-181a-5p
NM_001173430	1	rno-miR-3473
NM_001100978	3	rno-miR-3473|rno-miR-17-5p|rno-miR-93-5p
NM_001108576	1	rno-miR-3473

NM_001014080	1	rno-miR-3473
NM_057188	1	rno-miR-3473
NM_001044276	1	rno-miR-3473
NM_001100865	1	rno-miR-3473
NM_001107109	2	rno-miR-3473|rno-miR-181a-5p
NM_001270538	2	rno-miR-3473|rno-miR-151-5p
NM_001014030	1	rno-miR-3473
NM_001108301	6	rno-miR-3473|rno-miR-27b-3p|rno-miR-205|rno-miR-27a-3p|rno-miR-17-5p|rno-miR-93-5p
NM_053619	1	rno-miR-3473
NM_001106287	1	rno-miR-3473
NM_001024346	1	rno-miR-3473
NM_023955	4	rno-miR-3473|rno-miR-181a-5p|rno-miR-17-5p|rno-miR-93-5p
NM_001305435	1	rno-miR-3473
NM_001134626	1	rno-miR-3473
NM_031705	1	rno-miR-3473
NM_017173	2	rno-miR-3473|rno-miR-29a-3p
NM_001002808	1	rno-miR-3473
NM_013199	1	rno-miR-3473
NM_012713	1	rno-miR-3473
NM_001108761	1	rno-miR-3473
NM_001007654	1	rno-miR-3473
NM_001108520	3	rno-miR-3473|rno-miR-181a-5p|rno-miR-152-3p
NM_019181	1	rno-miR-3473
NM_031732	3	rno-miR-3473|rno-miR-17-5p|rno-miR-93-5p
NM_012648	1	rno-miR-3473
NM_001107115	3	rno-miR-3473|rno-miR-17-5p|rno-miR-93-5p
NM_001107436	1	rno-miR-3473
NM_001271325	1	rno-miR-3473
NM_017317	1	rno-miR-3473

NM_001109622	1	rno-miR-3473
NM_031782	1	rno-miR-3473
NM_031011	1	rno-miR-3473
NM_001127681	4	rno-miR-3473|rno-miR-30c-5p|rno-miR-17-5p|rno-miR-93-5p
NM_001009683	1	rno-miR-3473
NM_001032285	2	rno-miR-3473|rno-miR-200b-3p
NM_001109255	1	rno-miR-3473
NM_001127607	1	rno-miR-3473
NM_001012195	1	rno-miR-3473
NM_001107887	1	rno-miR-3473
NM_001108780	1	rno-miR-3473
NM_031534	1	rno-miR-3473
NM_053679	1	rno-miR-3473
NM_001013911	1	rno-miR-3473
NM_130823	1	rno-miR-3473
NM_001007671	3	rno-miR-3473|rno-miR-17-5p|rno-miR-93-5p
NM_001191862	1	rno-miR-3473
NM_001108869	1	rno-miR-3473
NM_001104527	3	rno-miR-3473|rno-miR-17-5p|rno-miR-93-5p
NM_001100671	3	rno-miR-125a-5p|rno-miR-200b-3p|rno-miR-30c-5p
NM_033098	1	rno-miR-125a-5p
NM_053997	1	rno-miR-125a-5p
NM_053572	1	rno-miR-320-3p
NM_001271251	1	rno-miR-320-3p
NM_001024313	1	rno-miR-320-3p
NM_001108739	1	rno-miR-320-3p
NM_001163568	1	rno-miR-320-3p
NM_001270846	1	rno-miR-320-3p
NM_001107657	1	rno-miR-320-3p
NM_001034942	3	rno-miR-320-3p|rno-miR-17-5p|rno-miR-93-5p

NM_013214	1	rno-miR-320-3p
NM_001040271	1	rno-miR-320-3p
NM_030865	1	rno-miR-320-3p
NM_031024	1	rno-miR-320-3p
NM_001271108	2	rno-miR-320-3p|rno-miR-181a-5p
NM_001134523	1	rno-miR-320-3p
NM_001025743	1	rno-miR-320-3p
NM_212519	1	rno-miR-320-3p
NM_001106847	1	rno-miR-320-3p
NM_001013967	2	rno-miR-320-3p|rno-miR-200b-3p
NM_001009688	1	rno-miR-320-3p
NM_001017451	1	rno-miR-320-3p
NM_053729	3	rno-miR-320-3p|rno-miR-29a-3p|rno-miR-30c-5p
NM_012628	1	rno-miR-320-3p
NM_001111294	1	rno-miR-320-3p
NM_001146061	1	rno-miR-320-3p
NM_001108871	1	rno-miR-320-3p
NM_053668	1	rno-miR-320-3p
NM_053994	1	rno-miR-320-3p
NM_001013233	1	rno-miR-320-3p
NM_001007691	1	rno-miR-320-3p
NM_001013151	1	rno-miR-320-3p
NM_181365	1	rno-miR-320-3p
NM_001107680	2	rno-miR-320-3p|rno-miR-29a-3p
NM_001130580	2	rno-miR-320-3p|rno-miR-31a-5p
NM_001271731	1	rno-miR-320-3p
NM_031123	3	rno-miR-320-3p|rno-miR-181a-5p|rno-miR-30c-5p
NM_001270848	1	rno-miR-320-3p
NM_001271301	2	rno-miR-320-3p|rno-miR-30c-5p
NM_017060	1	rno-miR-320-3p

NM_001108019	1	rno-miR-320-3p
NM_001134737	1	rno-miR-320-3p
NM_001108709	3	rno-miR-320-3p|rno-miR-17-5p|rno-miR-93-5p
NM_001007687	2	rno-miR-320-3p|rno-miR-31a-5p
NM_001012226	1	rno-miR-320-3p
NM_001107824	1	rno-miR-320-3p
NM_001107793	2	rno-miR-320-3p|rno-miR-152-3p
NM_001107198	1	rno-miR-320-3p
NM_001039033	1	rno-miR-320-3p
NM_212538	3	rno-miR-320-3p|rno-miR-17-5p|rno-miR-93-5p
NM_001135015	1	rno-miR-320-3p
NM_001271227	1	rno-miR-320-3p
NM_130779	1	rno-miR-320-3p
NM_001001511	1	rno-miR-320-3p
NM_001006970	1	rno-miR-320-3p
NM_001012011	1	rno-miR-320-3p
NM_001271494	1	rno-miR-320-3p
NM_001105977	1	rno-miR-320-3p
NM_001130493	1	rno-miR-320-3p
NM_001012148	2	rno-miR-320-3p|rno-miR-22-3p
NM_022858	2	rno-miR-320-3p|rno-miR-378a-3p
NM_001191653	1	rno-miR-320-3p
NM_001105976	2	rno-miR-320-3p|rno-miR-30c-5p
NM_001008366	1	rno-miR-320-3p
NM_001013106	1	rno-miR-320-3p
NM_024137	3	rno-miR-320-3p|rno-miR-27b-3p|rno-miR-27a-3p
NM_053710	1	rno-miR-320-3p
NM_001130495	1	rno-miR-320-3p
NM_153629	1	rno-miR-320-3p
NM_019184	1	rno-miR-320-3p

NM_001109898	1	rno-miR-320-3p
NM_019300	1	rno-miR-320-3p
NM_001135561	5	rno-miR-320-3p|rno-miR-199a-3p|rno-miR-378a-3p|rno-miR-200b-3p|rno-miR-152-3p
NM_001107227	1	rno-miR-320-3p
NM_207587	1	rno-miR-320-3p
NM_001005534	1	rno-miR-320-3p
NM_183325	1	rno-miR-320-3p
NM_019243	4	rno-miR-320-3p|rno-miR-30c-5p|rno-miR-17-5p|rno-miR-93-5p
NM_017303	1	rno-miR-320-3p
NM_133593	2	rno-miR-320-3p|rno-miR-30c-5p
NM_001170403	1	rno-miR-320-3p
NM_001106546	1	rno-miR-320-3p
NM_001134519	1	rno-miR-320-3p
NM_001134504	1	rno-miR-320-3p
NM_001191626	4	rno-miR-320-3p|rno-miR-29a-3p|rno-miR-181a-5p|rno-miR-30c-5p
NM_053979	2	rno-miR-320-3p|rno-miR-181a-5p
NM_001106032	1	rno-miR-320-3p
NM_173120	1	rno-miR-320-3p
NM_013133	1	rno-miR-320-3p
NM_001030037	4	rno-miR-320-3p|rno-miR-27b-3p|rno-miR-27a-3p|rno-miR-30c-5p
NM_053523	1	rno-miR-320-3p
NM_001305175	4	rno-miR-320-3p|rno-miR-27b-3p|rno-miR-27a-3p|rno-miR-181a-5p
NM_013004	2	rno-miR-320-3p|rno-miR-200b-3p
NM_001077231	3	rno-miR-320-3p|rno-miR-17-5p|rno-miR-93-5p
NM_001013236	1	rno-miR-320-3p
NM_001191958	2	rno-miR-320-3p|rno-miR-205
NM_001107183	3	rno-miR-320-3p|rno-miR-27b-3p|rno-miR-27a-3p
NM_001271299	2	rno-miR-320-3p|rno-miR-30c-5p
NM_182822	1	rno-miR-320-3p

NM_001109678	2	rno-miR-320-3p|rno-miR-30c-5p
NM_024362	3	rno-miR-320-3p|rno-miR-27b-3p|rno-miR-27a-3p
NM_139263	4	rno-miR-320-3p|rno-miR-29a-3p|rno-miR-17-5p|rno-miR-93-5p
NM_001003978	3	rno-miR-320-3p|rno-miR-27b-3p|rno-miR-27a-3p
NM_001013210	1	rno-miR-320-3p
NM_001271271	3	rno-miR-320-3p|rno-miR-27b-3p|rno-miR-27a-3p
NM_001134621	2	rno-miR-320-3p|rno-miR-200b-3p
NM_031821	4	rno-miR-320-3p|rno-miR-27b-3p|rno-miR-27a-3p|rno-miR-200b-3p
NM_001107637	1	rno-miR-320-3p
NM_001013908	1	rno-miR-320-3p
NM_021846	2	rno-miR-320-3p|rno-miR-29a-3p
NM_001108034	3	rno-miR-320-3p|rno-miR-200b-3p|rno-miR-31a-5p
NM_001004267	2	rno-miR-320-3p|rno-miR-30c-5p
NM_019358	1	rno-miR-320-3p
NM_053347	1	rno-miR-320-3p
NM_017356	1	rno-miR-320-3p
NM_017243	1	rno-miR-320-3p
NM_053289	1	rno-miR-320-3p
NM_001025669	1	rno-miR-320-3p
NM_001270847	1	rno-miR-320-3p
NM_001108718	1	rno-miR-320-3p
NM_001008311	1	rno-miR-320-3p
NM_019372	2	rno-miR-320-3p|rno-miR-181a-5p
NM_001109203	1	rno-miR-320-3p
NM_001105761	1	rno-miR-320-3p
NM_001025029	1	rno-miR-320-3p
NM_001107446	2	rno-miR-320-3p|rno-miR-378a-3p
NM_133310	2	rno-miR-320-3p|rno-miR-152-3p
NM_001033923	1	rno-miR-320-3p
NM_022194	1	rno-miR-320-3p

NM_001106631	1	rno-miR-320-3p
NM_001106191	1	rno-miR-320-3p
NM_052807	1	rno-miR-320-3p
NM_176078	1	rno-miR-320-3p
NM_001108362	1	rno-miR-320-3p
NM_001107929	1	rno-miR-320-3p
NM_053342	1	rno-miR-320-3p
NM_139114	2	rno-miR-320-3p|rno-miR-30c-5p
NM_031715	1	rno-miR-320-3p
NM_001134501	1	rno-miR-320-3p
NM_053697	1	rno-miR-320-3p
NM_001014223	1	rno-miR-320-3p
NM_001034020	1	rno-miR-320-3p
NM_001109317	1	rno-miR-320-3p
NM_001107991	1	rno-miR-320-3p
NM_001079689	1	rno-miR-320-3p
NM_001108934	1	rno-miR-320-3p
NM_001108578	2	rno-miR-320-3p|rno-miR-205
NM_022589	2	rno-miR-320-3p|rno-miR-181a-5p
NM_001109599	2	rno-miR-320-3p|rno-miR-181a-5p
NM_001037643	1	rno-miR-320-3p
NM_001008771	1	rno-miR-320-3p
NM_013145	2	rno-miR-320-3p|rno-miR-30c-5p
NM_001097581	1	rno-miR-320-3p
NM_001109118	2	rno-miR-320-3p|rno-miR-22-3p
NM_053877	1	rno-miR-320-3p
NM_001012103	1	rno-miR-320-3p
NM_001014067	1	rno-miR-320-3p
NM_031002	2	rno-miR-320-3p|rno-miR-200b-3p
NM_001100518	2	rno-miR-320-3p|rno-miR-200b-3p

NM_053592	1	rno-miR-320-3p
NM_001305212	1	rno-miR-320-3p
NM_001100558	5	rno-miR-320-3p|rno-miR-22-3p|rno-miR-29a-3p|rno-miR-17-5p|rno-miR-93-5p
NM_001107371	3	rno-miR-320-3p|rno-miR-27b-3p|rno-miR-27a-3p
NM_001007003	1	rno-miR-320-3p
NM_001014176	1	rno-miR-320-3p
NM_001108711	1	rno-miR-320-3p
NM_001106122	1	rno-miR-320-3p
NM_017022	2	rno-miR-320-3p|rno-miR-29a-3p
NM_001107625	1	rno-miR-320-3p
NM_001004210	1	rno-miR-320-3p
NM_001024756	3	rno-miR-320-3p|rno-miR-29a-3p|rno-miR-31a-5p
NM_001109056	3	rno-miR-320-3p|rno-miR-378a-3p|rno-miR-181a-5p
NM_001134447	1	rno-miR-320-3p
NM_012619	1	rno-miR-320-3p
NM_001173527	1	rno-miR-320-3p
NM_133399	4	rno-miR-320-3p|rno-miR-27b-3p|rno-miR-27a-3p|rno-miR-200b-3p
NM_001127492	1	rno-miR-320-3p
NM_001014069	1	rno-miR-320-3p
NM_001109374	2	rno-miR-320-3p|rno-miR-151-5p
NM_001126092	1	rno-miR-320-3p
NM_001013182	1	rno-miR-320-3p
NM_001108214	4	rno-miR-320-3p|rno-miR-378a-3p|rno-miR-17-5p|rno-miR-93-5p
NM_001014083	3	rno-miR-320-3p|rno-miR-200b-3p|rno-miR-30c-5p
NM_133545	5	rno-miR-320-3p|rno-miR-200b-3p|rno-miR-30c-5p|rno-miR-17-5p|rno-miR-93-5p
NM_012541	1	rno-miR-320-3p
NM_001109531	1	rno-miR-320-3p
NM_001107748	2	rno-miR-320-3p|rno-miR-30c-5p

NM_001108422	1	rno-miR-320-3p
NM_145092	1	rno-miR-320-3p
NM_031983	2	rno-miR-320-3p|rno-miR-30c-5p
NM_001108916	2	rno-miR-320-3p|rno-miR-30c-5p
NM_001025735	1	rno-miR-320-3p
NM_001134861	1	rno-miR-320-3p
NM_001144851	1	rno-miR-320-3p
NM_001166586	2	rno-miR-320-3p|rno-miR-181a-5p
NM_001034912	2	rno-miR-320-3p|rno-miR-31a-5p
NM_031616	1	rno-miR-320-3p
NM_001106495	1	rno-miR-320-3p
NM_001008313	3	rno-miR-320-3p|rno-miR-99b-5p|rno-miR-200b-3p
NM_001134603	2	rno-miR-320-3p|rno-miR-200b-3p
NM_001017511	1	rno-miR-320-3p
NM_181370	1	rno-miR-99b-5p
NM_001044251	1	rno-miR-99b-5p
NM_001270985	1	rno-miR-99b-5p
NM_001109250	3	rno-miR-99b-5p|rno-miR-27b-3p|rno-miR-27a-3p
NM_019906	2	rno-miR-99b-5p|rno-miR-199a-3p
NM_001107419	2	rno-miR-99b-5p|rno-miR-181a-5p
NM_001109564	5	rno-miR-99b-5p|rno-miR-27b-3p|rno-miR-378a-3p|rno-miR-27a-3p|rno-miR-29a-3p
NM_001191867	1	rno-miR-99b-5p
NM_001106275	4	rno-miR-99b-5p|rno-miR-27b-3p|rno-miR-205|rno-miR-27a-3p
NM_001173334	1	rno-miR-99b-5p
NM_001077669	1	rno-miR-99b-5p
NM_199092	3	rno-miR-99b-5p|rno-miR-27b-3p|rno-miR-27a-3p
NM_001191850	2	rno-miR-99b-5p|rno-miR-181a-5p
NM_001025019	1	rno-miR-99b-5p
NM_001106480	1	rno-miR-199a-3p

NM_013080	2	rno-miR-199a-3p|rno-miR-200b-3p
NM_001108796	2	rno-miR-199a-3p|rno-miR-30c-5p
NM_001191570	5	rno-miR-199a-3p|rno-miR-378a-3p|rno-miR-181a-5p|rno-miR-17-5p|rno-miR-93-5p
NM_001106677	3	rno-miR-199a-3p|rno-miR-17-5p|rno-miR-93-5p
NM_001003957	4	rno-miR-199a-3p|rno-miR-29a-3p|rno-miR-200b-3p|rno-miR-30c-5p
NM_001105989	1	rno-miR-199a-3p
NM_001017466	1	rno-miR-199a-3p
NM_001012005	1	rno-miR-199a-3p
NM_001025132	1	rno-miR-199a-3p
NM_053999	2	rno-miR-199a-3p|rno-miR-31a-5p
NM_001108585	1	rno-miR-199a-3p
NM_001037653	2	rno-miR-199a-3p|rno-miR-200b-3p
NM_001035002	1	rno-miR-199a-3p
NM_001105777	1	rno-miR-199a-3p
NM_001014269	1	rno-miR-199a-3p
NM_001034147	1	rno-miR-199a-3p
NM_031686	1	rno-miR-199a-3p
NM_001011991	1	rno-miR-199a-3p
NM_019164	1	rno-miR-199a-3p
NM_001106192	1	rno-miR-199a-3p
NM_022523	1	rno-miR-199a-3p
NM_001107926	1	rno-miR-199a-3p
NM_001030022	1	rno-miR-199a-3p
NM_022689	2	rno-miR-199a-3p|rno-miR-31a-5p
NM_199269	1	rno-miR-199a-3p
NM_001271232	1	rno-miR-199a-3p
NM_001012227	1	rno-miR-199a-3p
NM_012872	1	rno-miR-199a-3p
NM_031797	2	rno-miR-199a-3p|rno-miR-22-3p

NM_012986	3	rno-miR-199a-3p|rno-miR-378a-3p|rno-miR-30c-5p
NM_001003958	4	rno-miR-199a-3p|rno-miR-29a-3p|rno-miR-200b-3p|rno-miR-30c-5p
NM_131907	1	rno-miR-199a-3p
NM_001191610	1	rno-miR-199a-3p
NM_001134994	2	rno-miR-199a-3p|rno-miR-29a-3p
NM_001107456	1	rno-miR-199a-3p
NM_001107847	1	rno-miR-199a-3p
NM_001108068	1	rno-miR-199a-3p
NM_001191567	1	rno-miR-199a-3p
NM_001169578	1	rno-miR-199a-3p
NM_001107722	2	rno-miR-199a-3p|rno-miR-181a-5p
NM_001277177	2	rno-miR-199a-3p|rno-miR-30c-5p
NM_001109384	1	rno-miR-199a-3p
NM_001013082	4	rno-miR-199a-3p|rno-miR-30c-5p|rno-miR-17-5p|rno-miR-93-5p
NM_001107395	1	rno-miR-199a-3p
NM_001107096	3	rno-miR-199a-3p|rno-miR-17-5p|rno-miR-93-5p
NM_001107630	1	rno-miR-199a-3p
NM_001108012	2	rno-miR-199a-3p|rno-miR-152-3p
NM_022520	1	rno-miR-199a-3p
NM_138514	1	rno-miR-199a-3p
NM_012906	2	rno-miR-199a-3p|rno-miR-200b-3p
NM_198769	1	rno-miR-199a-3p
NM_019143	2	rno-miR-199a-3p|rno-miR-200b-3p
NM_031833	1	rno-miR-199a-3p
NM_019269	1	rno-miR-199a-3p
NM_133401	1	rno-miR-199a-3p
NM_001106496	1	rno-miR-199a-3p
NM_133391	1	rno-miR-199a-3p
NM_001108866	1	rno-miR-199a-3p
NM_022610	4	rno-miR-199a-3p|rno-miR-29a-3p|rno-miR-17-5p|rno-miR-93-5p

NM_001014092	1	rno-miR-199a-3p
NM_013070	2	rno-miR-199a-3p|rno-miR-200b-3p
NM_012924	1	rno-miR-199a-3p
NM_001107323	1	rno-miR-199a-3p
NM_001012095	1	rno-miR-199a-3p
NM_001170399	1	rno-miR-199a-3p
NM_001127579	1	rno-miR-199a-3p
NM_031136	1	rno-miR-199a-3p
NM_130413	1	rno-miR-199a-3p
NM_001271365	1	rno-miR-199a-3p
NM_013007	1	rno-miR-199a-3p
NM_199388	1	rno-miR-199a-3p
NM_001007612	1	rno-miR-199a-3p
NM_001100702	2	rno-miR-199a-3p|rno-miR-200b-3p
NM_001014268	1	rno-miR-199a-3p
NM_001109422	1	rno-miR-199a-3p
NM_001106019	1	rno-miR-199a-3p
NM_001107281	1	rno-miR-199a-3p
NM_001033066	3	rno-miR-199a-3p|rno-miR-17-5p|rno-miR-93-5p
NM_001005885	1	rno-miR-199a-3p
NM_001170586	1	rno-miR-199a-3p
NM_001011929	1	rno-miR-199a-3p
NM_001169120	4	rno-miR-199a-3p|rno-miR-29a-3p|rno-miR-17-5p|rno-miR-93-5p
NM_001135755	1	rno-miR-199a-3p
NM_001106061	5	rno-miR-199a-3p|rno-miR-181a-5p|rno-miR-30c-5p|rno-miR-17-5p|rno-miR-93-5p
NM_001105999	6	rno-miR-199a-3p|rno-miR-27b-3p|rno-miR-27a-3p|rno-miR-30c-5p|rno-miR-17-5p|rno-miR-93-5p
NM_001034995	3	rno-miR-199a-3p|rno-miR-27b-3p|rno-miR-27a-3p
NM_173133	1	rno-miR-199a-3p

NM_001100508	1	rno-miR-199a-3p
NM_012928	2	rno-miR-27b-3p|rno-miR-27a-3p
NM_017074	3	rno-miR-27b-3p|rno-miR-27a-3p|rno-miR-30c-5p
NM_001108244	2	rno-miR-27b-3p|rno-miR-27a-3p
NM_001109183	3	rno-miR-27b-3p|rno-miR-27a-3p|rno-miR-200b-3p
NM_019177	2	rno-miR-27b-3p|rno-miR-27a-3p
NM_133511	2	rno-miR-27b-3p|rno-miR-27a-3p
NM_001025660	2	rno-miR-27b-3p|rno-miR-27a-3p
NM_001106114	2	rno-miR-27b-3p|rno-miR-27a-3p
NM_001108555	3	rno-miR-27b-3p|rno-miR-27a-3p|rno-miR-181a-5p
NM_021261	2	rno-miR-27b-3p|rno-miR-27a-3p
NM_031027	4	rno-miR-27b-3p|rno-miR-27a-3p|rno-miR-17-5p|rno-miR-93-5p
NM_001191586	3	rno-miR-27b-3p|rno-miR-27a-3p|rno-miR-30c-5p
NM_001039340	3	rno-miR-27b-3p|rno-miR-27a-3p|rno-miR-181a-5p
NM_022498	2	rno-miR-27b-3p|rno-miR-27a-3p
NM_001044237	2	rno-miR-27b-3p|rno-miR-27a-3p
NM_013124	2	rno-miR-27b-3p|rno-miR-27a-3p
NM_053635	2	rno-miR-27b-3p|rno-miR-27a-3p
NM_001270635	3	rno-miR-27b-3p|rno-miR-27a-3p|rno-miR-151-5p
NM_001106892	4	rno-miR-27b-3p|rno-miR-205|rno-miR-27a-3p|rno-miR-152-3p
NM_001108084	5	rno-miR-27b-3p|rno-miR-27a-3p|rno-miR-17-5p|rno-miR-15b-5p|rno-miR-93-5p
NM_001106493	2	rno-miR-27b-3p|rno-miR-27a-3p
NM_173100	2	rno-miR-27b-3p|rno-miR-27a-3p
NM_001014125	2	rno-miR-27b-3p|rno-miR-27a-3p
NM_001134840	2	rno-miR-27b-3p|rno-miR-27a-3p
NM_198737	3	rno-miR-27b-3p|rno-miR-27a-3p|rno-miR-152-3p
NM_001107518	2	rno-miR-27b-3p|rno-miR-27a-3p
NM_001013085	5	rno-miR-27b-3p|rno-miR-27a-3p|rno-miR-30c-5p|rno-miR-17-5p|rno-miR-93-5p

NM_001100697	4	rno-miR-27b-3p|rno-miR-27a-3p|rno-miR-200b-3p|rno-miR-30c-5p
NM_001270576	3	rno-miR-27b-3p|rno-miR-27a-3p|rno-miR-200b-3p
NM_001106678	3	rno-miR-27b-3p|rno-miR-27a-3p|rno-miR-181a-5p
NM_001271344	2	rno-miR-27b-3p|rno-miR-27a-3p
NM_013150	3	rno-miR-27b-3p|rno-miR-378a-3p|rno-miR-27a-3p
NM_031355	2	rno-miR-27b-3p|rno-miR-27a-3p
NM_001108091	2	rno-miR-27b-3p|rno-miR-27a-3p
NM_001034922	2	rno-miR-27b-3p|rno-miR-27a-3p
NM_001108745	4	rno-miR-27b-3p|rno-miR-27a-3p|rno-miR-30c-5p|rno-miR-152-3p
NM_001108404	2	rno-miR-27b-3p|rno-miR-27a-3p
NM_001037287	2	rno-miR-27b-3p|rno-miR-27a-3p
NM_012675	3	rno-miR-27b-3p|rno-miR-27a-3p|rno-miR-181a-5p
NM_053519	4	rno-miR-27b-3p|rno-miR-27a-3p|rno-miR-17-5p|rno-miR-93-5p
NM_001014232	2	rno-miR-27b-3p|rno-miR-27a-3p
NM_199373	2	rno-miR-27b-3p|rno-miR-27a-3p
NM_031635	2	rno-miR-27b-3p|rno-miR-27a-3p
NM_053440	3	rno-miR-27b-3p|rno-miR-27a-3p|rno-miR-29a-3p
NM_001134699	3	rno-miR-27b-3p|rno-miR-27a-3p|rno-miR-200b-3p
NM_001034130	2	rno-miR-27b-3p|rno-miR-27a-3p
NM_021681	2	rno-miR-27b-3p|rno-miR-27a-3p
NM_053380	2	rno-miR-27b-3p|rno-miR-27a-3p
NM_001191952	2	rno-miR-27b-3p|rno-miR-27a-3p
NM_001308255	3	rno-miR-27b-3p|rno-miR-27a-3p|rno-miR-29a-3p
NM_001142366	2	rno-miR-27b-3p|rno-miR-27a-3p
NM_022397	2	rno-miR-27b-3p|rno-miR-27a-3p
NM_183330	2	rno-miR-27b-3p|rno-miR-27a-3p
NM_001034000	2	rno-miR-27b-3p|rno-miR-27a-3p
NM_001127337	2	rno-miR-27b-3p|rno-miR-27a-3p
NM_022594	2	rno-miR-27b-3p|rno-miR-27a-3p
NM_001024895	2	rno-miR-27b-3p|rno-miR-27a-3p

NM_001039337	2	rno-miR-27b-3p|rno-miR-27a-3p
NM_001012175	2	rno-miR-27b-3p|rno-miR-27a-3p
NM_001100969	2	rno-miR-27b-3p|rno-miR-27a-3p
NM_001270631	3	rno-miR-27b-3p|rno-miR-27a-3p|rno-miR-151-5p
NM_001077674	2	rno-miR-27b-3p|rno-miR-27a-3p
NM_001271445	3	rno-miR-27b-3p|rno-miR-27a-3p|rno-miR-29a-3p
NM_001109219	2	rno-miR-27b-3p|rno-miR-27a-3p
NM_001276712	2	rno-miR-27b-3p|rno-miR-27a-3p
NM_001305279	3	rno-miR-27b-3p|rno-miR-27a-3p|rno-miR-181a-5p
NM_001008291	2	rno-miR-27b-3p|rno-miR-27a-3p
NM_021584	2	rno-miR-27b-3p|rno-miR-27a-3p
NM_001107285	2	rno-miR-27b-3p|rno-miR-27a-3p
NM_001108339	3	rno-miR-27b-3p|rno-miR-27a-3p|rno-miR-30c-5p
NM_001100974	3	rno-miR-27b-3p|rno-miR-27a-3p|rno-miR-152-3p
NM_024402	2	rno-miR-27b-3p|rno-miR-27a-3p
NM_001271278	3	rno-miR-27b-3p|rno-miR-27a-3p|rno-miR-30c-5p
NM_001135013	2	rno-miR-27b-3p|rno-miR-27a-3p
NM_001025289	2	rno-miR-27b-3p|rno-miR-27a-3p
NM_001134751	3	rno-miR-27b-3p|rno-miR-27a-3p|rno-miR-30c-5p
NM_001270785	2	rno-miR-27b-3p|rno-miR-27a-3p
NM_017071	2	rno-miR-27b-3p|rno-miR-27a-3p
NM_001170542	2	rno-miR-27b-3p|rno-miR-27a-3p
NM_133530	3	rno-miR-27b-3p|rno-miR-27a-3p|rno-miR-29a-3p
NM_053851	5	rno-miR-27b-3p|rno-miR-27a-3p|rno-miR-181a-5p|rno-miR-31a-5p|rno-miR-30c-5p
NM_001271354	2	rno-miR-27b-3p|rno-miR-27a-3p
NM_001105735	2	rno-miR-27b-3p|rno-miR-27a-3p
NM_030875	5	rno-miR-27b-3p|rno-miR-27a-3p|rno-miR-30c-5p|rno-miR-17-5p|rno-miR-93-5p
NM_001112712	3	rno-miR-27b-3p|rno-miR-27a-3p|rno-miR-181a-5p

NM_001108072	3	rno-miR-27b-3p|rno-miR-27a-3p|rno-miR-181a-5p
NM_001270796	3	rno-miR-27b-3p|rno-miR-205|rno-miR-27a-3p
NM_053401	2	rno-miR-27b-3p|rno-miR-27a-3p
NM_175578	2	rno-miR-27b-3p|rno-miR-27a-3p
NM_001039197	2	rno-miR-27b-3p|rno-miR-27a-3p
NM_001013202	2	rno-miR-27b-3p|rno-miR-27a-3p
NM_001034924	2	rno-miR-27b-3p|rno-miR-27a-3p
NM_133537	2	rno-miR-27b-3p|rno-miR-27a-3p
NM_001108610	2	rno-miR-27b-3p|rno-miR-27a-3p
NM_001015025	4	rno-miR-27b-3p|rno-miR-27a-3p|rno-miR-17-5p|rno-miR-93-5p
NM_001013991	3	rno-miR-27b-3p|rno-miR-27a-3p|rno-miR-30c-5p
NM_001145367	2	rno-miR-27b-3p|rno-miR-27a-3p
NM_001106809	3	rno-miR-27b-3p|rno-miR-27a-3p|rno-miR-31a-5p
NM_021692	2	rno-miR-27b-3p|rno-miR-27a-3p
NM_199110	2	rno-miR-27b-3p|rno-miR-27a-3p
NM_001107477	2	rno-miR-27b-3p|rno-miR-27a-3p
NM_001107397	2	rno-miR-27b-3p|rno-miR-27a-3p
NM_173323	2	rno-miR-27b-3p|rno-miR-27a-3p
NM_001106147	2	rno-miR-27b-3p|rno-miR-27a-3p
NM_001109149	2	rno-miR-27b-3p|rno-miR-27a-3p
NM_001271185	2	rno-miR-27b-3p|rno-miR-27a-3p
NM_001025146	2	rno-miR-27b-3p|rno-miR-27a-3p
NM_001033685	2	rno-miR-27b-3p|rno-miR-27a-3p
NM_022186	2	rno-miR-27b-3p|rno-miR-27a-3p
NM_001107196	2	rno-miR-27b-3p|rno-miR-27a-3p
NM_012825	2	rno-miR-27b-3p|rno-miR-27a-3p
NM_001037286	2	rno-miR-27b-3p|rno-miR-27a-3p
NM_199391	3	rno-miR-27b-3p|rno-miR-27a-3p|rno-miR-31a-5p
NM_012969	4	rno-miR-27b-3p|rno-miR-126a-3p|rno-miR-27a-3p|rno-miR-30c-5p
NM_133597	2	rno-miR-27b-3p|rno-miR-27a-3p

NM_001009409	3	rno-miR-27b-3p|rno-miR-27a-3p|rno-miR-29a-3p
NM_017268	3	rno-miR-27b-3p|rno-miR-27a-3p|rno-miR-29a-3p
NM_001007686	2	rno-miR-27b-3p|rno-miR-27a-3p
NM_001008337	2	rno-miR-27b-3p|rno-miR-27a-3p
NM_001012075	2	rno-miR-27b-3p|rno-miR-27a-3p
NM_001037285	2	rno-miR-27b-3p|rno-miR-27a-3p
NM_001002815	2	rno-miR-27b-3p|rno-miR-27a-3p
NM_001106215	4	rno-miR-27b-3p|rno-miR-27a-3p|rno-miR-17-5p|rno-miR-93-5p
NM_001009920	2	rno-miR-27b-3p|rno-miR-27a-3p
NM_001270581	2	rno-miR-27b-3p|rno-miR-27a-3p
NM_001270580	2	rno-miR-27b-3p|rno-miR-27a-3p
NM_001105882	2	rno-miR-27b-3p|rno-miR-27a-3p
NM_133290	3	rno-miR-27b-3p|rno-miR-27a-3p|rno-miR-29a-3p
NM_001108179	2	rno-miR-27b-3p|rno-miR-27a-3p
NM_212491	2	rno-miR-27b-3p|rno-miR-27a-3p
NM_001126266	2	rno-miR-27b-3p|rno-miR-27a-3p
NM_001100811	2	rno-miR-27b-3p|rno-miR-27a-3p
NM_001270575	3	rno-miR-27b-3p|rno-miR-27a-3p|rno-miR-200b-3p
NM_013146	4	rno-miR-27b-3p|rno-miR-27a-3p|rno-miR-17-5p|rno-miR-93-5p
NM_133526	2	rno-miR-27b-3p|rno-miR-27a-3p
NM_001024789	2	rno-miR-27b-3p|rno-miR-27a-3p
NM_001014254	3	rno-miR-27b-3p|rno-miR-27a-3p|rno-miR-29a-3p
NM_001025681	2	rno-miR-27b-3p|rno-miR-27a-3p
NM_001107747	4	rno-miR-27b-3p|rno-miR-27a-3p|rno-miR-17-5p|rno-miR-93-5p
NM_001013884	2	rno-miR-27b-3p|rno-miR-27a-3p
NM_001034953	2	rno-miR-27b-3p|rno-miR-27a-3p
NM_175754	3	rno-miR-27b-3p|rno-miR-27a-3p|rno-miR-29a-3p
NM_001008381	3	rno-miR-27b-3p|rno-miR-27a-3p|rno-miR-30c-5p
NM_019217	3	rno-miR-27b-3p|rno-miR-27a-3p|rno-miR-181a-5p
NM_001270632	3	rno-miR-27b-3p|rno-miR-27a-3p|rno-miR-151-5p

NM_001270854	3	rno-miR-27b-3p|rno-miR-27a-3p|rno-miR-181a-5p
NM_130401	2	rno-miR-27b-3p|rno-miR-27a-3p
NM_001271120	3	rno-miR-27b-3p|rno-miR-27a-3p|rno-miR-200b-3p
NM_001107030	2	rno-miR-27b-3p|rno-miR-27a-3p
NM_145768	2	rno-miR-27b-3p|rno-miR-27a-3p
NM_001107079	2	rno-miR-27b-3p|rno-miR-27a-3p
NM_057155	2	rno-miR-27b-3p|rno-miR-27a-3p
NM_001107633	2	rno-miR-27b-3p|rno-miR-27a-3p
NM_001287025	2	rno-miR-27b-3p|rno-miR-27a-3p
NM_001134616	2	rno-miR-27b-3p|rno-miR-27a-3p
NM_001308254	2	rno-miR-27b-3p|rno-miR-27a-3p
NM_001128195	2	rno-miR-27b-3p|rno-miR-27a-3p
NM_001107760	3	rno-miR-27b-3p|rno-miR-27a-3p|rno-miR-30c-5p
NM_001109608	2	rno-miR-27b-3p|rno-miR-27a-3p
NM_138882	2	rno-miR-27b-3p|rno-miR-27a-3p
NM_001024742	2	rno-miR-27b-3p|rno-miR-27a-3p
NM_001106187	2	rno-miR-27b-3p|rno-miR-27a-3p
NM_019336	2	rno-miR-27b-3p|rno-miR-27a-3p
NM_001108671	2	rno-miR-27b-3p|rno-miR-27a-3p
NM_001100637	3	rno-miR-27b-3p|rno-miR-27a-3p|rno-miR-30c-5p
NM_001270637	3	rno-miR-27b-3p|rno-miR-27a-3p|rno-miR-151-5p
NM_001271279	3	rno-miR-27b-3p|rno-miR-27a-3p|rno-miR-30c-5p
NM_001108127	3	rno-miR-27b-3p|rno-miR-27a-3p|rno-miR-152-3p
NM_013067	2	rno-miR-27b-3p|rno-miR-27a-3p
NM_001191778	2	rno-miR-27b-3p|rno-miR-27a-3p
NM_001191846	2	rno-miR-27b-3p|rno-miR-27a-3p
NM_001108290	3	rno-miR-27b-3p|rno-miR-27a-3p|rno-miR-200b-3p
NM_001008725	3	rno-miR-27b-3p|rno-miR-27a-3p|rno-miR-152-3p
NM_001033676	2	rno-miR-27b-3p|rno-miR-27a-3p
NM_001109466	2	rno-miR-27b-3p|rno-miR-27a-3p

NM_001108281	2	rno-miR-27b-3p|rno-miR-27a-3p
NM_201989	2	rno-miR-27b-3p|rno-miR-27a-3p
NM_001037776	2	rno-miR-27b-3p|rno-miR-27a-3p
NM_001145366	2	rno-miR-27b-3p|rno-miR-27a-3p
NM_138905	3	rno-miR-27b-3p|rno-miR-27a-3p|rno-miR-200b-3p
NM_130826	2	rno-miR-27b-3p|rno-miR-27a-3p
NM_001100975	2	rno-miR-27b-3p|rno-miR-27a-3p
NM_001107077	2	rno-miR-27b-3p|rno-miR-27a-3p
NM_057131	2	rno-miR-27b-3p|rno-miR-27a-3p
NM_001008336	2	rno-miR-27b-3p|rno-miR-27a-3p
NM_001108788	3	rno-miR-27b-3p|rno-miR-27a-3p|rno-miR-30c-5p
NM_001024904	3	rno-miR-27b-3p|rno-miR-27a-3p|rno-miR-152-3p
NM_001134642	2	rno-miR-27b-3p|rno-miR-27a-3p
NM_172090	3	rno-miR-27b-3p|rno-miR-27a-3p|rno-miR-31a-5p
NM_001107123	3	rno-miR-27b-3p|rno-miR-27a-3p|rno-miR-29a-3p
NM_012613	2	rno-miR-27b-3p|rno-miR-27a-3p
NM_021579	2	rno-miR-27b-3p|rno-miR-27a-3p
NM_001106045	2	rno-miR-27b-3p|rno-miR-27a-3p
NM_001106401	2	rno-miR-27b-3p|rno-miR-27a-3p
NM_001005560	3	rno-miR-27b-3p|rno-miR-205|rno-miR-27a-3p
NM_001017382	2	rno-miR-27b-3p|rno-miR-27a-3p
NM_001007712	2	rno-miR-27b-3p|rno-miR-27a-3p
NM_001107764	2	rno-miR-27b-3p|rno-miR-27a-3p
NM_133601	4	rno-miR-27b-3p|rno-miR-22-3p|rno-miR-27a-3p|rno-miR-30c-5p
NM_013149	3	rno-miR-27b-3p|rno-miR-27a-3p|rno-miR-29a-3p
NM_001270558	2	rno-miR-27b-3p|rno-miR-27a-3p
NM_012615	2	rno-miR-27b-3p|rno-miR-27a-3p
NM_133529	2	rno-miR-27b-3p|rno-miR-27a-3p
NM_001191967	2	rno-miR-27b-3p|rno-miR-27a-3p
NM_017278	2	rno-miR-27b-3p|rno-miR-27a-3p

NM_001106172	2	rno-miR-27b-3p|rno-miR-27a-3p
NM_001108921	3	rno-miR-27b-3p|rno-miR-27a-3p|rno-miR-30c-5p
NM_001270630	3	rno-miR-27b-3p|rno-miR-27a-3p|rno-miR-151-5p
NM_016986	2	rno-miR-27b-3p|rno-miR-27a-3p
NM_030997	2	rno-miR-27b-3p|rno-miR-27a-3p
NM_053928	2	rno-miR-27b-3p|rno-miR-27a-3p
NM_001108357	2	rno-miR-27b-3p|rno-miR-27a-3p
NM_001306054	2	rno-miR-27b-3p|rno-miR-27a-3p
NM_022297	3	rno-miR-27b-3p|rno-miR-27a-3p|rno-miR-30c-5p
NM_001106120	3	rno-miR-27b-3p|rno-miR-27a-3p|rno-miR-200b-3p
NM_001012743	3	rno-miR-27b-3p|rno-miR-27a-3p|rno-miR-30c-5p
NM_001134634	2	rno-miR-27b-3p|rno-miR-27a-3p
NM_001270633	3	rno-miR-27b-3p|rno-miR-27a-3p|rno-miR-151-5p
NM_001106566	3	rno-miR-27b-3p|rno-miR-27a-3p|rno-miR-181a-5p
NM_019123	5	rno-miR-27b-3p|rno-miR-205|rno-miR-27a-3p|rno-miR-17-5p|rno-miR-93-5p
NM_199268	2	rno-miR-27b-3p|rno-miR-27a-3p
NM_001012116	2	rno-miR-27b-3p|rno-miR-27a-3p
NM_001109615	3	rno-miR-27b-3p|rno-miR-27a-3p|rno-miR-152-3p
NM_012821	2	rno-miR-27b-3p|rno-miR-27a-3p
NM_001047097	3	rno-miR-27b-3p|rno-miR-205|rno-miR-27a-3p
NM_053370	2	rno-miR-27b-3p|rno-miR-27a-3p
NM_001113184	2	rno-miR-27b-3p|rno-miR-27a-3p
NM_001106164	2	rno-miR-27b-3p|rno-miR-27a-3p
NM_001271336	2	rno-miR-27b-3p|rno-miR-27a-3p
NM_173139	2	rno-miR-27b-3p|rno-miR-27a-3p
NM_001034028	3	rno-miR-27b-3p|rno-miR-27a-3p|rno-miR-181a-5p
NM_001012046	2	rno-miR-27b-3p|rno-miR-27a-3p
NM_023991	2	rno-miR-27b-3p|rno-miR-27a-3p
NM_022215	2	rno-miR-27b-3p|rno-miR-27a-3p

NM_001024988	2	rno-miR-27b-3p|rno-miR-27a-3p
NM_001115043	2	rno-miR-27b-3p|rno-miR-27a-3p
NM_001135600	2	rno-miR-27b-3p|rno-miR-27a-3p
NM_001127484	4	rno-miR-27b-3p|rno-miR-27a-3p|rno-miR-29a-3p|rno-miR-30c-5p
NM_001145005	2	rno-miR-27b-3p|rno-miR-27a-3p
NM_198783	2	rno-miR-27b-3p|rno-miR-27a-3p
NM_031242	3	rno-miR-27b-3p|rno-miR-27a-3p|rno-miR-152-3p
NM_001276304	2	rno-miR-27b-3p|rno-miR-27a-3p
NM_012542	2	rno-miR-27b-3p|rno-miR-27a-3p
NM_001106089	2	rno-miR-27b-3p|rno-miR-27a-3p
NM_001270582	2	rno-miR-27b-3p|rno-miR-27a-3p
NM_001170408	3	rno-miR-27b-3p|rno-miR-27a-3p|rno-miR-29a-3p
NM_017161	2	rno-miR-27b-3p|rno-miR-27a-3p
NM_021676	2	rno-miR-27b-3p|rno-miR-27a-3p
NM_001108153	2	rno-miR-27b-3p|rno-miR-27a-3p
NM_017213	2	rno-miR-27b-3p|rno-miR-27a-3p
NM_022797	2	rno-miR-27b-3p|rno-miR-27a-3p
NM_001108740	3	rno-miR-27b-3p|rno-miR-27a-3p|rno-miR-200b-3p
NM_001317749	2	rno-miR-27b-3p|rno-miR-27a-3p
NM_001135698	3	rno-miR-27b-3p|rno-miR-27a-3p|rno-miR-152-3p
NM_001191792	2	rno-miR-27b-3p|rno-miR-27a-3p
NM_021680	2	rno-miR-27b-3p|rno-miR-27a-3p
NM_053970	3	rno-miR-27b-3p|rno-miR-27a-3p|rno-miR-181a-5p
NM_001134689	2	rno-miR-27b-3p|rno-miR-27a-3p
NM_001191727	2	rno-miR-27b-3p|rno-miR-27a-3p
NM_001107930	2	rno-miR-27b-3p|rno-miR-27a-3p
NM_175761	2	rno-miR-27b-3p|rno-miR-27a-3p
NM_001017491	2	rno-miR-27b-3p|rno-miR-27a-3p
NM_001302083	2	rno-miR-27b-3p|rno-miR-27a-3p
NM_001106373	2	rno-miR-27b-3p|rno-miR-27a-3p

NM_017325	2	rno-miR-27b-3p|rno-miR-27a-3p
NM_199118	2	rno-miR-27b-3p|rno-miR-27a-3p
NM_001270579	2	rno-miR-27b-3p|rno-miR-27a-3p
NM_001271339	2	rno-miR-27b-3p|rno-miR-27a-3p
NM_001108810	2	rno-miR-27b-3p|rno-miR-27a-3p
NM_001108048	3	rno-miR-27b-3p|rno-miR-27a-3p|rno-miR-152-3p
NM_001100735	4	rno-miR-27b-3p|rno-miR-205|rno-miR-27a-3p|rno-miR-181a-5p
NM_001112742	3	rno-miR-27b-3p|rno-miR-27a-3p|rno-miR-181a-5p
NM_001127657	3	rno-miR-27b-3p|rno-miR-205|rno-miR-27a-3p
NM_001107720	2	rno-miR-27b-3p|rno-miR-27a-3p
NM_001109502	2	rno-miR-27b-3p|rno-miR-27a-3p
NM_001270638	3	rno-miR-27b-3p|rno-miR-27a-3p|rno-miR-151-5p
NM_001106802	2	rno-miR-27b-3p|rno-miR-27a-3p
NM_001166342	2	rno-miR-27b-3p|rno-miR-27a-3p
NM_023979	4	rno-miR-27b-3p|rno-miR-27a-3p|rno-miR-181a-5p|rno-miR-30c-5p
NM_019380	3	rno-miR-27b-3p|rno-miR-27a-3p|rno-miR-152-3p
NM_001170456	3	rno-miR-27b-3p|rno-miR-27a-3p|rno-miR-152-3p
NM_001034111	2	rno-miR-27b-3p|rno-miR-27a-3p
NM_001270636	3	rno-miR-27b-3p|rno-miR-27a-3p|rno-miR-151-5p
NM_001270713	2	rno-miR-27b-3p|rno-miR-27a-3p
NM_001014250	4	rno-miR-27b-3p|rno-miR-27a-3p|rno-miR-181a-5p|rno-miR-30c-5p
NM_001108444	4	rno-miR-27b-3p|rno-miR-27a-3p|rno-miR-17-5p|rno-miR-93-5p
NM_001109258	2	rno-miR-27b-3p|rno-miR-27a-3p
NM_019342	2	rno-miR-27b-3p|rno-miR-27a-3p
NM_031698	2	rno-miR-27b-3p|rno-miR-27a-3p
NM_001012087	2	rno-miR-27b-3p|rno-miR-27a-3p
NM_173146	2	rno-miR-27b-3p|rno-miR-27a-3p
NM_001009539	2	rno-miR-27b-3p|rno-miR-27a-3p
NM_053379	4	rno-miR-27b-3p|rno-miR-27a-3p|rno-miR-29a-3p|rno-miR-30c-5p

NM_001024371	5	rno-miR-27b-3p|rno-miR-27a-3p|rno-miR-181a-5p|rno-miR-31a-5p|rno-miR-30c-5p
NM_001270559	2	rno-miR-27b-3p|rno-miR-27a-3p
NM_033442	3	rno-miR-27b-3p|rno-miR-27a-3p|rno-miR-200b-3p
NM_030836	2	rno-miR-27b-3p|rno-miR-27a-3p
NM_198781	2	rno-miR-27b-3p|rno-miR-27a-3p
NM_203337	2	rno-miR-27b-3p|rno-miR-27a-3p
NM_030991	3	rno-miR-27b-3p|rno-miR-27a-3p|rno-miR-200b-3p
NM_001014088	2	rno-miR-27b-3p|rno-miR-27a-3p
NM_001108734	2	rno-miR-27b-3p|rno-miR-27a-3p
NM_001301876	2	rno-miR-27b-3p|rno-miR-27a-3p
NM_017033	3	rno-miR-27b-3p|rno-miR-27a-3p|rno-miR-30c-5p
NM_031121	2	rno-miR-27b-3p|rno-miR-27a-3p
NM_001106990	4	rno-miR-27b-3p|rno-miR-27a-3p|rno-miR-17-5p|rno-miR-93-5p
NM_017077	2	rno-miR-27b-3p|rno-miR-27a-3p
NM_012945	2	rno-miR-27b-3p|rno-miR-27a-3p
NM_001106251	2	rno-miR-27b-3p|rno-miR-27a-3p
NM_001106160	2	rno-miR-27b-3p|rno-miR-27a-3p
NM_021759	2	rno-miR-27b-3p|rno-miR-27a-3p
NM_001108843	2	rno-miR-27b-3p|rno-miR-27a-3p
NM_001108504	2	rno-miR-27b-3p|rno-miR-27a-3p
NM_001134510	2	rno-miR-27b-3p|rno-miR-27a-3p
NM_001012169	2	rno-miR-27b-3p|rno-miR-27a-3p
NM_001106589	3	rno-miR-27b-3p|rno-miR-27a-3p|rno-miR-181a-5p
NM_183053	2	rno-miR-27b-3p|rno-miR-27a-3p
NM_139113	2	rno-miR-27b-3p|rno-miR-27a-3p
NM_017353	3	rno-miR-27b-3p|rno-miR-27a-3p|rno-miR-152-3p
NM_001080153	2	rno-miR-27b-3p|rno-miR-27a-3p
NM_001172305	3	rno-miR-27b-3p|rno-miR-27a-3p|rno-miR-200b-3p
NM_001109672	4	rno-miR-27b-3p|rno-miR-27a-3p|rno-miR-181a-5p|rno-miR-30c-5p

NM_012568	2	rno-miR-27b-3p|rno-miR-27a-3p
NM_001305995	3	rno-miR-27b-3p|rno-miR-27a-3p|rno-miR-29a-3p
NM_001109667	2	rno-miR-27b-3p|rno-miR-27a-3p
NM_001108743	4	rno-miR-27b-3p|rno-miR-27a-3p|rno-miR-17-5p|rno-miR-93-5p
NM_001167665	2	rno-miR-27b-3p|rno-miR-27a-3p
NM_001013149	2	rno-miR-27b-3p|rno-miR-27a-3p
NM_031851	3	rno-miR-27b-3p|rno-miR-205|rno-miR-27a-3p
NM_001107347	2	rno-miR-27b-3p|rno-miR-27a-3p
NM_001109180	2	rno-miR-27b-3p|rno-miR-27a-3p
NM_016990	2	rno-miR-27b-3p|rno-miR-27a-3p
NM_001271054	6	rno-miR-27b-3p|rno-miR-27a-3p|rno-miR-200b-3p|rno-miR-30c-5p|rno-miR-17-5p|rno-miR-93-5p
NM_001106483	2	rno-miR-27b-3p|rno-miR-27a-3p
NM_173138	2	rno-miR-27b-3p|rno-miR-27a-3p
NM_031657	3	rno-miR-27b-3p|rno-miR-27a-3p|rno-miR-181a-5p
NM_001015005	2	rno-miR-27b-3p|rno-miR-27a-3p
NM_001013205	2	rno-miR-27b-3p|rno-miR-27a-3p
NM_001109372	2	rno-miR-27b-3p|rno-miR-27a-3p
NM_053605	3	rno-miR-27b-3p|rno-miR-27a-3p|rno-miR-29a-3p
NM_138867	3	rno-miR-27b-3p|rno-miR-27a-3p|rno-miR-181a-5p
NM_001113185	3	rno-miR-27b-3p|rno-miR-27a-3p|rno-miR-181a-5p
NM_057127	2	rno-miR-27b-3p|rno-miR-27a-3p
NM_001015032	5	rno-miR-27b-3p|rno-miR-22-3p|rno-miR-378a-3p|rno-miR-27a-3p|rno-miR-30c-5p
NM_001109545	2	rno-miR-27b-3p|rno-miR-27a-3p
NM_031004	3	rno-miR-27b-3p|rno-miR-27a-3p|rno-miR-181a-5p
NM_001134750	3	rno-miR-27b-3p|rno-miR-27a-3p|rno-miR-30c-5p
NM_001045843	2	rno-miR-27b-3p|rno-miR-27a-3p
NM_080395	3	rno-miR-27b-3p|rno-miR-27a-3p|rno-miR-181a-5p
NM_001012230	2	rno-miR-27b-3p|rno-miR-27a-3p

NM_013134	4	rno-miR-27b-3p|rno-miR-27a-3p|rno-miR-29a-3p|rno-miR-200b-3p
NM_001270583	2	rno-miR-27b-3p|rno-miR-27a-3p
NM_001109237	3	rno-miR-27b-3p|rno-miR-27a-3p|rno-miR-30c-5p
NM_001105963	2	rno-miR-27b-3p|rno-miR-27a-3p
NM_001159739	2	rno-miR-27b-3p|rno-miR-27a-3p
NM_012672	3	rno-miR-27b-3p|rno-miR-27a-3p|rno-miR-181a-5p
NM_173837	3	rno-miR-27b-3p|rno-miR-27a-3p|rno-miR-152-3p
NM_001036626	5	rno-miR-27b-3p|rno-miR-378a-3p|rno-miR-27a-3p|rno-miR-181a-5p|rno-miR-30c-5p
NM_001134718	2	rno-miR-27b-3p|rno-miR-27a-3p
NM_001106264	2	rno-miR-27b-3p|rno-miR-27a-3p
NM_021658	2	rno-miR-27b-3p|rno-miR-27a-3p
NM_001007758	2	rno-miR-27b-3p|rno-miR-27a-3p
NM_001004204	2	rno-miR-27b-3p|rno-miR-27a-3p
NM_001270634	3	rno-miR-27b-3p|rno-miR-27a-3p|rno-miR-151-5p
NM_001108609	4	rno-miR-27b-3p|rno-miR-205|rno-miR-27a-3p|rno-miR-200b-3p
NM_001002819	3	rno-miR-27b-3p|rno-miR-27a-3p|rno-miR-30c-5p
NM_001191921	3	rno-miR-27b-3p|rno-miR-27a-3p|rno-miR-181a-5p
NM_001135603	3	rno-miR-27b-3p|rno-miR-27a-3p|rno-miR-31a-5p
NM_001103356	4	rno-miR-27b-3p|rno-miR-27a-3p|rno-miR-17-5p|rno-miR-93-5p
NM_001111095	3	rno-miR-27b-3p|rno-miR-27a-3p|rno-miR-151-5p
NM_017263	2	rno-miR-27b-3p|rno-miR-27a-3p
NM_012513	3	rno-miR-27b-3p|rno-miR-27a-3p|rno-miR-151-5p
NM_023981	4	rno-miR-27b-3p|rno-miR-205|rno-miR-27a-3p|rno-miR-152-3p
NM_012898	2	rno-miR-27b-3p|rno-miR-27a-3p
NM_022185	2	rno-miR-126a-3p|rno-miR-30c-5p
NM_001106817	1	rno-miR-22-3p
NM_001108891	1	rno-miR-22-3p
NM_001108207	4	rno-miR-22-3p|rno-miR-181a-5p|rno-miR-17-5p|rno-miR-93-5p
NM_053621	1	rno-miR-22-3p

NM_001106103	1	rno-miR-22-3p
NM_001106579	1	rno-miR-22-3p
NM_001039346	1	rno-miR-22-3p
NM_001048215	1	rno-miR-22-3p
NM_001162898	1	rno-miR-22-3p
NM_139189	1	rno-miR-22-3p
NM_001024899	1	rno-miR-22-3p
NM_134383	1	rno-miR-22-3p
NM_001108475	3	rno-miR-22-3p|rno-miR-17-5p|rno-miR-93-5p
NM_001012173	1	rno-miR-22-3p
NM_001134463	1	rno-miR-22-3p
NM_001166300	1	rno-miR-22-3p
NM_001009676	1	rno-miR-22-3p
NM_021997	2	rno-miR-22-3p|rno-miR-200b-3p
NM_001100755	1	rno-miR-22-3p
NM_001270803	1	rno-miR-22-3p
NM_213567	1	rno-miR-22-3p
NM_012843	1	rno-miR-22-3p
NM_053990	2	rno-miR-22-3p|rno-miR-30c-5p
NM_001127303	2	rno-miR-22-3p|rno-miR-205
NM_001106672	1	rno-miR-22-3p
NM_053985	2	rno-miR-22-3p|rno-miR-378a-3p
NM_001169144	1	rno-miR-22-3p
NM_001024992	1	rno-miR-22-3p
NM_001108829	1	rno-miR-22-3p
NM_017258	1	rno-miR-22-3p
NM_001024999	1	rno-miR-22-3p
NM_053959	1	rno-miR-22-3p
NM_001270804	1	rno-miR-22-3p
NM_001108253	1	rno-miR-22-3p

NM_001173507	3	rno-miR-22-3p|rno-miR-29a-3p|rno-miR-30c-5p
NM_057210	1	rno-miR-22-3p
NM_031742	1	rno-miR-22-3p
NM_001014046	1	rno-miR-22-3p
NM_001107306	1	rno-miR-22-3p
NM_138858	2	rno-miR-22-3p|rno-miR-378a-3p
NM_153722	2	rno-miR-22-3p|rno-miR-29a-3p
NM_001009660	1	rno-miR-22-3p
NM_001024274	1	rno-miR-22-3p
NM_001107553	2	rno-miR-22-3p|rno-miR-152-3p
NM_022500	1	rno-miR-22-3p
NM_001202552	1	rno-miR-22-3p
NM_022507	2	rno-miR-22-3p|rno-miR-152-3p
NM_053912	2	rno-miR-22-3p|rno-miR-200b-3p
NM_019155	1	rno-miR-22-3p
NM_024126	1	rno-miR-22-3p
NM_001127546	1	rno-miR-22-3p
NM_001106586	1	rno-miR-22-3p
NM_001109000	1	rno-miR-22-3p
NM_001109111	4	rno-miR-22-3p|rno-miR-205|rno-miR-181a-5p|rno-miR-31a-5p
NM_001100691	2	rno-miR-22-3p|rno-miR-152-3p
NM_001024298	1	rno-miR-22-3p
NM_031549	1	rno-miR-22-3p
NM_001168641	1	rno-miR-22-3p
NM_139255	1	rno-miR-22-3p
NM_001134883	1	rno-miR-22-3p
NM_001107487	1	rno-miR-22-3p
NM_001107069	1	rno-miR-22-3p
NM_001104630	2	rno-miR-22-3p|rno-miR-200b-3p
NM_172332	1	rno-miR-22-3p

NM_001107172	1	rno-miR-22-3p
NM_001079936	3	rno-miR-22-3p|rno-miR-17-5p|rno-miR-93-5p
NM_001004222	1	rno-miR-22-3p
NM_001014186	1	rno-miR-22-3p
NM_001025696	1	rno-miR-22-3p
NM_030828	1	rno-miR-22-3p
NM_001013906	1	rno-miR-22-3p
NM_001106674	1	rno-miR-22-3p
NM_001271134	2	rno-miR-22-3p|rno-miR-29a-3p
NM_001107148	2	rno-miR-22-3p|rno-miR-29a-3p
NM_012652	1	rno-miR-22-3p
NM_001105913	1	rno-miR-22-3p
NM_001013159	1	rno-miR-22-3p
NM_139089	2	rno-miR-22-3p|rno-miR-181a-5p
NM_001127456	1	rno-miR-22-3p
NM_001271086	1	rno-miR-22-3p
NM_019252	1	rno-miR-22-3p
NM_001014201	1	rno-miR-22-3p
NM_001014022	4	rno-miR-22-3p|rno-miR-17-5p|rno-miR-152-3p|rno-miR-93-5p
NM_001108716	1	rno-miR-22-3p
NM_001025114	4	rno-miR-22-3p|rno-miR-200b-3p|rno-miR-17-5p|rno-miR-93-5p
NM_001024760	1	rno-miR-22-3p
NM_001108978	2	rno-miR-22-3p|rno-miR-30c-5p
NM_001105943	2	rno-miR-22-3p|rno-miR-31a-5p
NM_001135873	1	rno-miR-22-3p
NM_001109114	1	rno-miR-22-3p
NM_138910	1	rno-miR-22-3p
NM_001108305	1	rno-miR-22-3p
NM_022595	2	rno-miR-22-3p|rno-miR-181a-5p
NM_001135915	1	rno-miR-22-3p

NM_053529	1	rno-miR-22-3p
NM_031022	1	rno-miR-22-3p
NM_013106	2	rno-miR-22-3p|rno-miR-200b-3p
NM_013154	2	rno-miR-22-3p|rno-miR-200b-3p
NM_001013109	1	rno-miR-22-3p
NM_001029901	1	rno-miR-22-3p
NM_001012055	1	rno-miR-22-3p
NM_001106038	1	rno-miR-22-3p
NM_031582	1	rno-miR-195-5p
NM_001109028	1	rno-miR-195-5p
NM_001012107	2	rno-miR-378a-3p|rno-miR-30c-5p
NM_053376	1	rno-miR-378a-3p
NM_001008289	1	rno-miR-378a-3p
NM_001107423	1	rno-miR-378a-3p
NM_001106223	1	rno-miR-378a-3p
NM_001277425	1	rno-miR-378a-3p
NM_198731	1	rno-miR-378a-3p
NM_053575	1	rno-miR-378a-3p
NM_001037657	2	rno-miR-378a-3p|rno-miR-30c-5p
NM_134375	1	rno-miR-378a-3p
NM_001106685	2	rno-miR-378a-3p|rno-miR-30c-5p
NM_001170590	1	rno-miR-378a-3p
NM_001005551	1	rno-miR-378a-3p
NM_001108832	1	rno-miR-378a-3p
NM_001134730	5	rno-miR-378a-3p|rno-miR-200b-3p|rno-miR-181a-5p|rno-miR-31a-5p|rno-miR-152-3p
NM_012693	2	rno-miR-378a-3p|rno-miR-30c-5p
NM_001107744	3	rno-miR-378a-3p|rno-miR-29a-3p|rno-miR-200b-3p
NM_001271396	3	rno-miR-378a-3p|rno-miR-29a-3p|rno-miR-152-3p
NM_012764	1	rno-miR-378a-3p

NM_001134839	1	rno-miR-378a-3p
NM_001107229	1	rno-miR-378a-3p
NM_001131013	2	rno-miR-378a-3p|rno-miR-29a-3p
NM_001014182	1	rno-miR-378a-3p
NM_001100815	1	rno-miR-378a-3p
NM_001107282	1	rno-miR-378a-3p
NM_001009671	1	rno-miR-378a-3p
NM_001277386	1	rno-miR-378a-3p
NM_001271376	1	rno-miR-378a-3p
NM_001199178	1	rno-miR-378a-3p
NM_001085352	1	rno-miR-378a-3p
NM_001191684	1	rno-miR-378a-3p
NM_020095	1	rno-miR-378a-3p
NM_001108486	2	rno-miR-378a-3p|rno-miR-30c-5p
NM_001115039	1	rno-miR-378a-3p
NM_001109381	2	rno-miR-378a-3p|rno-miR-152-3p
NM_017128	1	rno-miR-378a-3p
NM_001011665	1	rno-miR-378a-3p
NM_001017457	1	rno-miR-378a-3p
NM_001047907	1	rno-miR-378a-3p
NM_001107267	4	rno-miR-378a-3p|rno-miR-200b-3p|rno-miR-31a-5p|rno-miR-30c-5p
NM_001109133	1	rno-miR-378a-3p
NM_001033961	1	rno-miR-378a-3p
NM_138835	1	rno-miR-378a-3p
NM_001014162	1	rno-miR-378a-3p
NM_001012034	1	rno-miR-378a-3p
NM_024150	3	rno-miR-378a-3p|rno-miR-29a-3p|rno-miR-181a-5p
NM_001109079	4	rno-miR-378a-3p|rno-miR-29a-3p|rno-miR-17-5p|rno-miR-93-5p
NM_001013859	1	rno-miR-378a-3p
NM_001107025	1	rno-miR-378a-3p

NM_013414	1	rno-miR-378a-3p
NM_022599	1	rno-miR-378a-3p
NM_001013128	1	rno-miR-378a-3p
NM_001107974	1	rno-miR-378a-3p
NM_020094	1	rno-miR-378a-3p
NM_001130540	1	rno-miR-378a-3p
NM_001012218	1	rno-miR-378a-3p
NM_133577	1	rno-miR-378a-3p
NM_001044246	2	rno-miR-378a-3p|rno-miR-205
NM_001107445	1	rno-miR-378a-3p
NM_133314	1	rno-miR-378a-3p
NM_001008355	1	rno-miR-378a-3p
NM_001106046	1	rno-miR-378a-3p
NM_012905	1	rno-miR-378a-3p
NM_001108551	1	rno-miR-378a-3p
NM_030843	1	rno-miR-378a-3p
NM_131906	1	rno-miR-378a-3p
NM_001127597	2	rno-miR-378a-3p|rno-miR-200b-3p
NM_001271026	1	rno-miR-378a-3p
NM_001106900	1	rno-miR-378a-3p
NM_001109070	1	rno-miR-378a-3p
NM_181550	1	rno-miR-378a-3p
NM_138542	1	rno-miR-205
NM_001013935	1	rno-miR-205
NM_022525	1	rno-miR-205
NM_001033997	1	rno-miR-205
NM_173324	1	rno-miR-205
NM_001134361	1	rno-miR-205
NM_182823	3	rno-miR-205|rno-miR-181a-5p|rno-miR-30c-5p
NM_022950	2	rno-miR-205|rno-miR-152-3p

NM_021686	1	rno-miR-205
NM_001008314	2	rno-miR-205|rno-miR-31a-5p
NM_019357	1	rno-miR-205
NM_001135799	1	rno-miR-205
NM_001108532	1	rno-miR-205
NM_001025652	1	rno-miR-205
NM_001126296	1	rno-miR-205
NM_001012194	1	rno-miR-205
NM_001166676	2	rno-miR-205|rno-miR-181a-5p
NM_001014253	2	rno-miR-205|rno-miR-152-3p
NM_001007714	2	rno-miR-205|rno-miR-29a-3p
NM_001008331	1	rno-miR-205
NM_017136	1	rno-miR-205
NM_199253	2	rno-miR-205|rno-miR-152-3p
NM_001005554	1	rno-miR-205
NM_031743	4	rno-miR-205|rno-miR-17-5p|rno-miR-152-3p|rno-miR-93-5p
NM_001004209	1	rno-miR-205
NM_001108173	1	rno-miR-205
NM_001105898	1	rno-miR-205
NM_001013046	1	rno-miR-205
NM_001106130	1	rno-miR-205
NM_001005882	1	rno-miR-205
NM_001017502	2	rno-miR-205|rno-miR-30c-5p
NM_001106882	1	rno-miR-205
NM_053577	1	rno-miR-205
NM_199111	1	rno-miR-205
NM_020072	1	rno-miR-205
NM_001161846	1	rno-miR-205
NM_001278484	3	rno-miR-205|rno-miR-30c-5p|rno-miR-152-3p
NM_001039207	2	rno-miR-205|rno-miR-29a-3p

NM_001100728	2	rno-miR-205|rno-miR-30c-5p
NM_019161	1	rno-miR-205
NM_001108354	1	rno-miR-205
NM_001013169	1	rno-miR-205
NM_001106713	2	rno-miR-205|rno-miR-181a-5p
NM_001017480	1	rno-miR-205
NM_012557	1	rno-miR-205
NM_001014071	3	rno-miR-205|rno-miR-200b-3p|rno-miR-152-3p
NM_001135742	1	rno-miR-205
NM_001134585	1	rno-miR-205
NM_001035221	2	rno-miR-205|rno-miR-30c-5p
NM_001130490	2	rno-miR-205|rno-miR-200b-3p
NM_001107877	3	rno-miR-205|rno-miR-29a-3p|rno-miR-30c-5p
NM_012671	2	rno-miR-205|rno-miR-152-3p
NM_053465	1	rno-miR-205
NM_001108149	1	rno-miR-205
NM_001109341	1	rno-miR-205
NM_001024997	1	rno-miR-205
NM_001109240	1	rno-miR-205
NM_001012038	3	rno-miR-205|rno-miR-17-5p|rno-miR-93-5p
NM_017358	2	rno-miR-205|rno-miR-181a-5p
NM_022697	1	rno-miR-205
NM_001014209	1	rno-miR-205
NM_022535	3	rno-miR-205|rno-miR-200b-3p|rno-miR-30c-5p
NM_001025747	1	rno-miR-205
NM_001135895	1	rno-miR-205
NM_001011893	1	rno-miR-205
NM_016988	1	rno-miR-205
NM_001107525	1	rno-miR-205
NM_001013863	1	rno-miR-205

NM_001278483	3	rno-miR-205|rno-miR-30c-5p|rno-miR-152-3p
NM_001007641	2	rno-miR-205|rno-miR-29a-3p
NM_001107375	1	rno-miR-205
NM_001012023	1	rno-miR-205
NM_181082	2	rno-miR-205|rno-miR-31a-5p
NM_001109644	1	rno-miR-205
NM_183052	2	rno-miR-205|rno-miR-30c-5p
NM_001007613	3	rno-miR-205|rno-miR-17-5p|rno-miR-93-5p
NM_001271210	2	rno-miR-205|rno-miR-181a-5p
NM_001009422	1	rno-miR-205
NM_001108531	4	rno-miR-205|rno-miR-17-5p|rno-miR-152-3p|rno-miR-93-5p
NM_001108819	1	rno-miR-205
NM_022713	1	rno-miR-205
NM_012751	4	rno-miR-205|rno-miR-31a-5p|rno-miR-17-5p|rno-miR-93-5p
NM_001127532	1	rno-miR-205
NM_053525	1	rno-miR-205
NM_001042621	1	rno-miR-205
NM_053358	1	rno-miR-205
NM_001025646	3	rno-miR-205|rno-miR-17-5p|rno-miR-93-5p
NM_053457	1	rno-miR-205
NM_001037792	2	rno-miR-205|rno-miR-30c-5p
NM_001271139	1	rno-miR-205
NM_001107127	1	rno-miR-205
NM_001002016	1	rno-miR-205
NM_053520	1	rno-miR-205
NM_001013964	1	rno-miR-205
NM_001305153	1	rno-miR-205
NM_001109606	2	rno-miR-205|rno-miR-181a-5p
NM_001168632	1	rno-miR-205
NM_001033898	3	rno-miR-205|rno-miR-200b-3p|rno-miR-30c-5p

NM_001139483	1	rno-miR-205
NM_001011961	1	rno-miR-205
NM_013217	2	rno-miR-205|rno-miR-152-3p
NM_021696	1	rno-miR-205
NM_001024312	1	rno-miR-205
NM_031337	1	rno-miR-205
NM_001100472	1	rno-miR-205
NM_001077641	1	rno-miR-205
NM_001100683	1	rno-miR-205
NM_001013187	1	rno-miR-205
NM_001012092	2	rno-miR-205|rno-miR-29a-3p
NM_001137646	1	rno-miR-205
NM_001012172	1	rno-miR-205
NM_001191833	1	rno-miR-205
NM_012932	1	rno-miR-205
NM_001079711	1	rno-miR-205
NM_001108668	1	rno-miR-205
NM_001191872	1	rno-miR-205
NM_053383	1	rno-miR-205
NM_001107924	1	rno-miR-205
NM_017070	1	rno-miR-205
NM_001107670	1	rno-miR-205
NM_001270843	1	rno-miR-205
NM_022604	2	rno-miR-205|rno-miR-181a-5p
NM_001107993	1	rno-miR-205
NM_032083	1	rno-miR-205
NM_001101680	1	rno-miR-205
NM_001173386	2	rno-miR-205|rno-miR-31a-5p
NM_001109429	1	rno-miR-205
NM_001109669	1	rno-miR-205

NM_022944	1	rno-miR-205
NM_001271306	3	rno-miR-205|rno-miR-181a-5p|rno-miR-30c-5p
NM_001271090	2	rno-miR-205|rno-miR-200b-3p
NM_133536	1	rno-miR-205
NM_053620	1	rno-miR-205
NM_001106823	1	rno-miR-205
NM_133560	5	rno-miR-205|rno-miR-200b-3p|rno-miR-181a-5p|rno-miR-31a-5p|rno-miR-152-3p
NM_001109222	1	rno-miR-205
NM_001281824	1	rno-miR-205
NM_017242	1	rno-miR-205
NM_001134881	1	rno-miR-205
NM_001271283	1	rno-miR-205
NM_022540	1	rno-miR-205
NM_012587	1	rno-miR-29a-3p
NM_001014218	1	rno-miR-29a-3p
NM_001039006	1	rno-miR-29a-3p
NM_013174	1	rno-miR-29a-3p
NM_017030	1	rno-miR-29a-3p
NM_001107307	1	rno-miR-29a-3p
NM_053702	2	rno-miR-29a-3p|rno-miR-181a-5p
NM_001100535	1	rno-miR-29a-3p
NM_001034074	1	rno-miR-29a-3p
NM_031318	1	rno-miR-29a-3p
NM_053895	1	rno-miR-29a-3p
NM_001013070	1	rno-miR-29a-3p
NM_031973	1	rno-miR-29a-3p
NM_001107622	2	rno-miR-29a-3p|rno-miR-31a-5p
NM_001107394	1	rno-miR-29a-3p
NM_001106350	2	rno-miR-29a-3p|rno-miR-31a-5p

NM_017172	2	rno-miR-29a-3p|rno-miR-31a-5p
NM_001004215	1	rno-miR-29a-3p
NM_053312	2	rno-miR-29a-3p|rno-miR-200b-3p
NM_001134588	5	rno-miR-29a-3p|rno-miR-200b-3p|rno-miR-31a-5p|rno-miR-17-5p|rno-miR-93-5p
NM_001081634	1	rno-miR-29a-3p
NM_001013871	1	rno-miR-29a-3p
NM_001108563	1	rno-miR-29a-3p
NM_017321	1	rno-miR-29a-3p
NM_001007020	1	rno-miR-29a-3p
NM_001107676	3	rno-miR-29a-3p|rno-miR-181a-5p|rno-miR-30c-5p
NM_001100666	2	rno-miR-29a-3p|rno-miR-181a-5p
NM_001024302	1	rno-miR-29a-3p
NM_001190998	1	rno-miR-29a-3p
NM_001134963	1	rno-miR-29a-3p
NM_022957	1	rno-miR-29a-3p
NM_001191863	1	rno-miR-29a-3p
NM_138979	1	rno-miR-29a-3p
NM_001008826	1	rno-miR-29a-3p
NM_133402	3	rno-miR-29a-3p|rno-miR-200b-3p|rno-miR-31a-5p
NM_001108927	1	rno-miR-29a-3p
NM_001009316	1	rno-miR-29a-3p
NM_001191584	1	rno-miR-29a-3p
NM_001162930	1	rno-miR-29a-3p
NM_001106296	1	rno-miR-29a-3p
NM_001105868	1	rno-miR-29a-3p
NM_032069	1	rno-miR-29a-3p
NM_001025638	1	rno-miR-29a-3p
NM_001004217	1	rno-miR-29a-3p
NM_053796	1	rno-miR-29a-3p

NM_021752	1	rno-miR-29a-3p
NM_001134514	1	rno-miR-29a-3p
NM_173300	1	rno-miR-29a-3p
NM_001271177	1	rno-miR-29a-3p
NM_001271268	1	rno-miR-29a-3p
NM_001108679	1	rno-miR-29a-3p
NM_001077670	1	rno-miR-29a-3p
NM_001034071	1	rno-miR-29a-3p
NM_001201354	1	rno-miR-29a-3p
NM_001106928	1	rno-miR-29a-3p
NM_001107814	2	rno-miR-29a-3p|rno-miR-30c-5p
NM_001008560	2	rno-miR-29a-3p|rno-miR-30c-5p
NM_001037215	2	rno-miR-29a-3p|rno-miR-30c-5p
NM_001109130	1	rno-miR-29a-3p
NM_031979	2	rno-miR-29a-3p|rno-miR-30c-5p
NM_001106616	1	rno-miR-29a-3p
NM_031525	2	rno-miR-29a-3p|rno-miR-30c-5p
NM_001271138	1	rno-miR-29a-3p
NM_001282336	1	rno-miR-29a-3p
NM_053760	1	rno-miR-29a-3p
NM_001107858	1	rno-miR-29a-3p
NM_012716	1	rno-miR-29a-3p
NM_001007710	1	rno-miR-29a-3p
NM_001173974	1	rno-miR-29a-3p
NM_001012216	1	rno-miR-29a-3p
NM_001007731	2	rno-miR-29a-3p|rno-miR-200b-3p
NM_001034069	1	rno-miR-29a-3p
NM_001105717	4	rno-miR-29a-3p|rno-miR-30c-5p|rno-miR-17-5p|rno-miR-93-5p
NM_001109102	1	rno-miR-29a-3p
NM_001106792	1	rno-miR-29a-3p

NM_001015021	1	rno-miR-29a-3p
NM_001108299	1	rno-miR-29a-3p
NM_001034073	1	rno-miR-29a-3p
NM_001277228	2	rno-miR-29a-3p|rno-miR-200b-3p
NM_001191587	1	rno-miR-29a-3p
NM_138864	1	rno-miR-29a-3p
NM_001024892	1	rno-miR-29a-3p
NM_139102	2	rno-miR-29a-3p|rno-miR-152-3p
NM_001011971	3	rno-miR-29a-3p|rno-miR-200b-3p|rno-miR-30c-5p
NM_001008364	1	rno-miR-29a-3p
NM_012988	2	rno-miR-29a-3p|rno-miR-200b-3p
NM_032085	1	rno-miR-29a-3p
NM_012722	1	rno-miR-29a-3p
NM_031059	1	rno-miR-29a-3p
NM_001130560	2	rno-miR-29a-3p|rno-miR-200b-3p
NM_001106999	1	rno-miR-29a-3p
NM_001109352	2	rno-miR-29a-3p|rno-miR-17-5p
NM_001201355	1	rno-miR-29a-3p
NM_001105812	2	rno-miR-29a-3p|rno-miR-31a-5p
NM_001105826	1	rno-miR-29a-3p
NM_001105856	2	rno-miR-29a-3p|rno-miR-200b-3p
NM_053703	1	rno-miR-29a-3p
NM_001014226	1	rno-miR-29a-3p
NM_001106978	1	rno-miR-29a-3p
NM_001100520	1	rno-miR-29a-3p
NM_001108949	1	rno-miR-29a-3p
NM_182824	1	rno-miR-29a-3p
NM_001126268	1	rno-miR-29a-3p
NM_001108156	2	rno-miR-29a-3p|rno-miR-152-3p
NM_001191096	1	rno-miR-29a-3p

NM_022955	2	rno-miR-29a-3p|rno-miR-30c-5p
NM_001100741	1	rno-miR-29a-3p
NM_001108393	1	rno-miR-29a-3p
NM_001024774	1	rno-miR-29a-3p
NM_031339	1	rno-miR-29a-3p
NM_022863	1	rno-miR-29a-3p
NM_030850	1	rno-miR-29a-3p
NM_130829	1	rno-miR-29a-3p
NM_199102	1	rno-miR-29a-3p
NM_001106083	2	rno-miR-29a-3p|rno-miR-152-3p
NM_001107989	1	rno-miR-29a-3p
NM_057201	1	rno-miR-29a-3p
NM_001109473	3	rno-miR-29a-3p|rno-miR-200b-3p|rno-miR-31a-5p
NM_001013172	3	rno-miR-29a-3p|rno-miR-17-5p|rno-miR-93-5p
NM_001107139	2	rno-miR-29a-3p|rno-miR-30c-5p
NM_024369	2	rno-miR-29a-3p|rno-miR-200b-3p
NM_001108619	1	rno-miR-29a-3p
NM_024149	1	rno-miR-29a-3p
NM_001108655	1	rno-miR-29a-3p
NM_001002803	1	rno-miR-29a-3p
NM_001107017	1	rno-miR-29a-3p
NM_172319	1	rno-miR-29a-3p
NM_001107876	3	rno-miR-29a-3p|rno-miR-181a-5p|rno-miR-152-3p
NM_001012089	3	rno-miR-29a-3p|rno-miR-17-5p|rno-miR-93-5p
NM_013091	1	rno-miR-29a-3p
NM_001047101	1	rno-miR-29a-3p
NM_001013971	1	rno-miR-29a-3p
NM_012656	1	rno-miR-29a-3p
NM_001107199	2	rno-miR-29a-3p|rno-miR-30c-5p
NM_145090	3	rno-miR-29a-3p|rno-miR-17-5p|rno-miR-93-5p

NM_001106673	1	rno-miR-29a-3p
NM_001106858	1	rno-miR-29a-3p
NM_001105827	1	rno-miR-29a-3p
NM_023023	3	rno-miR-29a-3p|rno-miR-17-5p|rno-miR-93-5p
NM_001035222	2	rno-miR-29a-3p|rno-miR-181a-5p
NM_001172056	1	rno-miR-29a-3p
NM_001106536	2	rno-miR-29a-3p|rno-miR-30c-5p
NM_031524	1	rno-miR-29a-3p
NM_001100842	1	rno-miR-29a-3p
NM_001277306	1	rno-miR-29a-3p
NM_001034070	1	rno-miR-29a-3p
NM_212544	1	rno-miR-29a-3p
NM_001108008	2	rno-miR-29a-3p|rno-miR-30c-5p
NM_024358	1	rno-miR-29a-3p
NM_001201353	1	rno-miR-29a-3p
NM_053449	1	rno-miR-29a-3p
NM_001271175	1	rno-miR-29a-3p
NM_001244739	1	rno-miR-29a-3p
NM_001195564	1	rno-miR-29a-3p
NM_080582	1	rno-miR-29a-3p
NM_001003402	1	rno-miR-29a-3p
NM_001107177	1	rno-miR-29a-3p
NM_012789	1	rno-miR-29a-3p
NM_001047858	2	rno-miR-29a-3p|rno-miR-200b-3p
NM_001014120	1	rno-miR-29a-3p
NM_001013227	1	rno-miR-200b-3p
NM_184050	1	rno-miR-200b-3p
NM_017246	1	rno-miR-200b-3p
NM_001309260	1	rno-miR-200b-3p
NM_001107026	1	rno-miR-200b-3p

NM_031041	1	rno-miR-200b-3p
NM_139097	1	rno-miR-200b-3p
NM_001047743	1	rno-miR-200b-3p
NM_001106525	1	rno-miR-200b-3p
NM_053830	1	rno-miR-200b-3p
NM_001136241	1	rno-miR-200b-3p
NM_057153	4	rno-miR-200b-3p|rno-miR-30c-5p|rno-miR-17-5p|rno-miR-93-5p
NM_001277288	3	rno-miR-200b-3p|rno-miR-17-5p|rno-miR-93-5p
NM_001024765	1	rno-miR-200b-3p
NM_001108774	1	rno-miR-200b-3p
NM_001271121	1	rno-miR-200b-3p
NM_001030020	1	rno-miR-200b-3p
NM_130405	1	rno-miR-200b-3p
NM_133397	1	rno-miR-200b-3p
NM_013062	1	rno-miR-200b-3p
NM_001106637	1	rno-miR-200b-3p
NM_001010956	1	rno-miR-200b-3p
NM_001126270	1	rno-miR-200b-3p
NM_001106716	3	rno-miR-200b-3p|rno-miR-17-5p|rno-miR-93-5p
NM_031730	1	rno-miR-200b-3p
NM_001012042	1	rno-miR-200b-3p
NM_017164	1	rno-miR-200b-3p
NM_031019	1	rno-miR-200b-3p
NM_001199168	1	rno-miR-200b-3p
NM_001271118	1	rno-miR-200b-3p
NM_001277287	3	rno-miR-200b-3p|rno-miR-17-5p|rno-miR-93-5p
NM_001100556	1	rno-miR-200b-3p
NM_001106786	1	rno-miR-200b-3p
NM_001025047	1	rno-miR-200b-3p
NM_001100658	1	rno-miR-200b-3p

NM_001197907	4	rno-miR-200b-3p|rno-miR-30c-5p|rno-miR-17-5p|rno-miR-93-5p
NM_057119	1	rno-miR-200b-3p
NM_053599	1	rno-miR-200b-3p
NM_001108016	3	rno-miR-200b-3p|rno-miR-181a-5p|rno-miR-152-3p
NM_133318	1	rno-miR-200b-3p
NM_013187	2	rno-miR-200b-3p|rno-miR-30c-5p
NM_001009665	1	rno-miR-200b-3p
NM_001164060	1	rno-miR-200b-3p
NM_001271126	1	rno-miR-200b-3p
NM_001271151	1	rno-miR-200b-3p
NM_001009635	1	rno-miR-200b-3p
NM_173118	1	rno-miR-200b-3p
NM_031239	1	rno-miR-200b-3p
NM_001191591	1	rno-miR-200b-3p
NM_001006959	1	rno-miR-200b-3p
NM_001108388	2	rno-miR-200b-3p|rno-miR-31a-5p
NM_012573	1	rno-miR-200b-3p
NM_001106701	1	rno-miR-200b-3p
NM_019359	1	rno-miR-200b-3p
NM_001025137	1	rno-miR-200b-3p
NM_001082540	1	rno-miR-200b-3p
NM_001107772	1	rno-miR-200b-3p
NM_017039	1	rno-miR-200b-3p
NM_001011974	1	rno-miR-200b-3p
NM_138544	1	rno-miR-200b-3p
NM_001270562	1	rno-miR-200b-3p
NM_053517	1	rno-miR-200b-3p
NM_001014184	1	rno-miR-200b-3p
NM_001270869	1	rno-miR-200b-3p
NM_013186	1	rno-miR-200b-3p

NM_022951	2	rno-miR-200b-3p|rno-miR-152-3p
NM_001106632	3	rno-miR-200b-3p|rno-miR-181a-5p|rno-miR-31a-5p
NM_001105957	3	rno-miR-200b-3p|rno-miR-17-5p|rno-miR-93-5p
NM_173102	1	rno-miR-200b-3p
NM_001013873	2	rno-miR-200b-3p|rno-miR-30c-5p
NM_001100710	1	rno-miR-200b-3p
NM_001109124	1	rno-miR-200b-3p
NM_001251926	1	rno-miR-200b-3p
NM_001271200	1	rno-miR-200b-3p
NM_001106970	3	rno-miR-200b-3p|rno-miR-31a-5p|rno-miR-151-5p
NM_203334	3	rno-miR-200b-3p|rno-miR-17-5p|rno-miR-93-5p
NM_001025283	1	rno-miR-200b-3p
NM_001271342	1	rno-miR-200b-3p
NM_001106909	1	rno-miR-200b-3p
NM_001109287	1	rno-miR-200b-3p
NM_001106737	1	rno-miR-200b-3p
NM_031023	1	rno-miR-200b-3p
NM_144730	1	rno-miR-200b-3p
NM_001108837	1	rno-miR-200b-3p
NM_001271273	1	rno-miR-200b-3p
NM_001034032	1	rno-miR-200b-3p
NM_001004227	1	rno-miR-200b-3p
NM_057205	1	rno-miR-200b-3p
NM_053814	1	rno-miR-200b-3p
NM_001108056	3	rno-miR-200b-3p|rno-miR-17-5p|rno-miR-93-5p
NM_031588	1	rno-miR-200b-3p
NM_012726	2	rno-miR-200b-3p|rno-miR-152-3p
NM_057185	1	rno-miR-200b-3p
NM_001192004	1	rno-miR-200b-3p
NM_001257352	3	rno-miR-200b-3p|rno-miR-17-5p|rno-miR-93-5p

NM_052979	1	rno-miR-200b-3p
NM_012950	1	rno-miR-200b-3p
NM_001277160	1	rno-miR-200b-3p
NM_001271201	1	rno-miR-200b-3p
NM_053765	2	rno-miR-200b-3p|rno-miR-31a-5p
NM_001270972	1	rno-miR-200b-3p
NM_001107546	1	rno-miR-200b-3p
NM_130819	1	rno-miR-200b-3p
NM_001130539	1	rno-miR-200b-3p
NM_012862	1	rno-miR-200b-3p
NM_001109096	1	rno-miR-200b-3p
NM_001271199	1	rno-miR-200b-3p
NM_001004238	1	rno-miR-200b-3p
NM_031695	1	rno-miR-200b-3p
NM_001271127	1	rno-miR-200b-3p
NM_053759	2	rno-miR-200b-3p|rno-miR-30c-5p
NM_053604	2	rno-miR-200b-3p|rno-miR-181a-5p
NM_001106746	1	rno-miR-200b-3p
NM_001004446	1	rno-miR-200b-3p
NM_001082539	1	rno-miR-200b-3p
NM_001199167	1	rno-miR-200b-3p
NM_139254	1	rno-miR-200b-3p
NM_001145755	2	rno-miR-200b-3p|rno-miR-30c-5p
NM_001025703	1	rno-miR-200b-3p
NM_001007688	1	rno-miR-200b-3p
NM_001160162	1	rno-miR-200b-3p
NM_031084	1	rno-miR-200b-3p
NM_001271119	1	rno-miR-200b-3p
NM_001108958	1	rno-miR-200b-3p
NM_001109563	1	rno-miR-200b-3p

NM_001270563	1	rno-miR-200b-3p
NM_001008369	1	rno-miR-200b-3p
NM_001009699	1	rno-miR-200b-3p
NM_001197332	4	rno-miR-200b-3p|rno-miR-30c-5p|rno-miR-17-5p|rno-miR-93-5p
NM_001173341	1	rno-miR-200b-3p
NM_001191068	4	rno-miR-200b-3p|rno-miR-181a-5p|rno-miR-30c-5p|rno-miR-152-3p
NM_001107978	1	rno-miR-200b-3p
NM_001192016	2	rno-miR-200b-3p|rno-miR-31a-5p
NM_001127531	1	rno-miR-200b-3p
NM_001014002	1	rno-miR-200b-3p
NM_172321	2	rno-miR-200b-3p|rno-miR-152-3p
NM_001271361	1	rno-miR-200b-3p
NM_001014150	2	rno-miR-200b-3p|rno-miR-152-3p
NM_001108795	1	rno-miR-200b-3p
NM_013088	1	rno-miR-200b-3p
NM_021835	1	rno-miR-200b-3p
NM_053421	4	rno-miR-200b-3p|rno-miR-30c-5p|rno-miR-17-5p|rno-miR-93-5p
NM_001039025	1	rno-miR-200b-3p
NM_001034022	1	rno-miR-200b-3p
NM_001100647	1	rno-miR-200b-3p
NM_001025770	2	rno-miR-200b-3p|rno-miR-30c-5p
NM_001109158	1	rno-miR-200b-3p
NM_001107391	1	rno-miR-200b-3p
NM_001127642	1	rno-miR-200b-3p
NM_153624	1	rno-miR-200b-3p
NM_001271173	2	rno-miR-200b-3p|rno-miR-30c-5p
NM_001270561	1	rno-miR-200b-3p
NM_053474	3	rno-miR-200b-3p|rno-miR-31a-5p|rno-miR-152-3p
NM_153735	2	rno-miR-200b-3p|rno-miR-152-3p
NM_031985	1	rno-miR-200b-3p

NM_001271129	1	rno-miR-200b-3p
NM_001170603	3	rno-miR-200b-3p|rno-miR-181a-5p|rno-miR-30c-5p
NM_031073	1	rno-miR-200b-3p
NM_001109414	1	rno-miR-200b-3p
NM_031804	1	rno-miR-200b-3p
NM_001044267	1	rno-miR-200b-3p
NM_019196	1	rno-miR-200b-3p
NM_022593	1	rno-miR-200b-3p
NM_053769	1	rno-miR-200b-3p
NM_001109157	1	rno-miR-200b-3p
NM_001170326	3	rno-miR-200b-3p|rno-miR-17-5p|rno-miR-93-5p
NM_001108873	1	rno-miR-200b-3p
NM_001108621	1	rno-miR-200b-3p
NM_001033987	1	rno-miR-200b-3p
NM_012865	1	rno-miR-200b-3p
NM_022521	1	rno-miR-200b-3p
NM_001111064	3	rno-miR-200b-3p|rno-miR-181a-5p|rno-miR-30c-5p
NM_001008558	1	rno-miR-200b-3p
NM_001107882	3	rno-miR-200b-3p|rno-miR-17-5p|rno-miR-93-5p
NM_001047856	2	rno-miR-200b-3p|rno-miR-181a-5p
NM_001011934	1	rno-miR-200b-3p
NM_001109454	5	rno-miR-200b-3p|rno-miR-181a-5p|rno-miR-17-5p|rno-miR-152-3p|rno-miR-93-5p
NM_001024787	1	rno-miR-200b-3p
NM_001012357	1	rno-miR-200b-3p
NM_130421	1	rno-miR-200b-3p
NM_139332	1	rno-miR-200b-3p
NM_053456	1	rno-miR-200b-3p
NM_001109552	2	rno-miR-200b-3p|rno-miR-181a-5p
NM_001108566	1	rno-miR-200b-3p

NM_001082541	1	rno-miR-200b-3p
NM_001270868	1	rno-miR-200b-3p
NM_001107086	1	rno-miR-200b-3p
NM_134389	1	rno-miR-200b-3p
NM_001017463	1	rno-miR-200b-3p
NM_001270870	1	rno-miR-200b-3p
NM_001126287	2	rno-miR-200b-3p|rno-miR-30c-5p
NM_012895	1	rno-miR-200b-3p
NM_175765	2	rno-miR-200b-3p|rno-miR-30c-5p
NM_182672	1	rno-miR-200b-3p
NM_024404	1	rno-miR-200b-3p
NM_145669	1	rno-miR-200b-3p
NM_181473	1	rno-miR-200b-3p
NM_001009357	2	rno-miR-200b-3p|rno-miR-30c-5p
NM_001033926	1	rno-miR-200b-3p
NM_203493	1	rno-miR-200b-3p
NM_198768	1	rno-miR-200b-3p
NM_012566	1	rno-miR-200b-3p
NM_032060	1	rno-miR-200b-3p
NM_001109076	1	rno-miR-200b-3p
NM_001108457	1	rno-miR-200b-3p
NM_173293	2	rno-miR-200b-3p|rno-miR-181a-5p
NM_001134472	1	rno-miR-200b-3p
NM_001108175	5	rno-miR-200b-3p|rno-miR-31a-5p|rno-miR-30c-5p|rno-miR-17-5p|rno-miR-93-5p
NM_001014170	1	rno-miR-200b-3p
NM_017315	1	rno-miR-200b-3p
NM_001107314	1	rno-miR-200b-3p
NM_012699	3	rno-miR-200b-3p|rno-miR-17-5p|rno-miR-93-5p
NM_001177593	1	rno-miR-200b-3p

NM_001109161	1	rno-miR-200b-3p
NM_001007628	1	rno-miR-200b-3p
NM_001005553	1	rno-miR-200b-3p
NM_001010963	1	rno-miR-200b-3p
NM_001271130	1	rno-miR-200b-3p
NM_012790	1	rno-miR-200b-3p
NM_013050	2	rno-miR-200b-3p|rno-miR-30c-5p
NM_182951	2	rno-miR-200b-3p|rno-miR-30c-5p
NM_001107666	2	rno-miR-200b-3p|rno-miR-181a-5p
NM_001191600	1	rno-miR-200b-3p
NM_001106993	1	rno-miR-200b-3p
NM_001127552	1	rno-miR-181a-5p
NM_001114401	1	rno-miR-181a-5p
NM_001029910	1	rno-miR-181a-5p
NM_001107275	1	rno-miR-181a-5p
NM_001305127	2	rno-miR-181a-5p|rno-miR-152-3p
NM_023020	2	rno-miR-181a-5p|rno-miR-30c-5p
NM_001008319	1	rno-miR-181a-5p
NM_031579	2	rno-miR-181a-5p|rno-miR-30c-5p
NM_001109176	1	rno-miR-181a-5p
NM_053657	1	rno-miR-181a-5p
NM_001107238	1	rno-miR-181a-5p
NM_001304352	3	rno-miR-181a-5p|rno-miR-17-5p|rno-miR-93-5p
NM_001134858	4	rno-miR-181a-5p|rno-miR-30c-5p|rno-miR-17-5p|rno-miR-93-5p
NM_001005538	1	rno-miR-181a-5p
NM_012546	4	rno-miR-181a-5p|rno-miR-30c-5p|rno-miR-17-5p|rno-miR-93-5p
NM_053644	1	rno-miR-181a-5p
NM_001047852	1	rno-miR-181a-5p
NM_013102	1	rno-miR-181a-5p
NM_001004075	1	rno-miR-181a-5p

NM_001107019	1	rno-miR-181a-5p
NM_012592	1	rno-miR-181a-5p
NM_175597	3	rno-miR-181a-5p|rno-miR-17-5p|rno-miR-93-5p
NM_012836	1	rno-miR-181a-5p
NM_001014273	1	rno-miR-181a-5p
NM_001105733	1	rno-miR-181a-5p
NM_053802	1	rno-miR-181a-5p
NM_053696	1	rno-miR-181a-5p
NM_001108667	1	rno-miR-181a-5p
NM_001025672	1	rno-miR-181a-5p
NM_133621	1	rno-miR-181a-5p
NM_001127557	1	rno-miR-181a-5p
NM_013113	1	rno-miR-181a-5p
NM_001100863	1	rno-miR-181a-5p
NM_001108664	1	rno-miR-181a-5p
NM_001012749	1	rno-miR-181a-5p
NM_001108333	1	rno-miR-181a-5p
NM_021857	1	rno-miR-181a-5p
NM_001107638	1	rno-miR-181a-5p
NM_001105808	1	rno-miR-181a-5p
NM_013083	1	rno-miR-181a-5p
NM_001013077	3	rno-miR-181a-5p|rno-miR-17-5p|rno-miR-93-5p
NM_001130988	2	rno-miR-181a-5p|rno-miR-31a-5p
NM_001013157	1	rno-miR-181a-5p
NM_001271330	1	rno-miR-181a-5p
NM_023974	1	rno-miR-181a-5p
NM_001130503	1	rno-miR-181a-5p
NM_001106476	1	rno-miR-181a-5p
NM_001106411	1	rno-miR-181a-5p
NM_138823	1	rno-miR-181a-5p

NM_001305213	1	rno-miR-181a-5p
NM_080697	2	rno-miR-181a-5p|rno-miR-152-3p
NM_001177442	2	rno-miR-181a-5p|rno-miR-30c-5p
NM_001015030	1	rno-miR-181a-5p
NM_001024802	1	rno-miR-181a-5p
NM_001106128	1	rno-miR-181a-5p
NM_001134998	1	rno-miR-181a-5p
NM_001012098	1	rno-miR-181a-5p
NM_012955	1	rno-miR-181a-5p
NM_001134726	1	rno-miR-181a-5p
NM_001037096	1	rno-miR-181a-5p
NM_001025412	1	rno-miR-181a-5p
NM_017180	1	rno-miR-181a-5p
NM_001005561	2	rno-miR-181a-5p|rno-miR-31a-5p
NM_203366	1	rno-miR-181a-5p
NM_001106634	1	rno-miR-181a-5p
NM_031120	1	rno-miR-181a-5p
NM_031056	1	rno-miR-181a-5p
NM_139112	1	rno-miR-181a-5p
NM_001108969	1	rno-miR-181a-5p
NM_001107342	1	rno-miR-181a-5p
NM_013110	1	rno-miR-181a-5p
NM_001009831	1	rno-miR-181a-5p
NM_031816	3	rno-miR-181a-5p|rno-miR-17-5p|rno-miR-93-5p
NM_001107729	1	rno-miR-181a-5p
NM_001134565	2	rno-miR-181a-5p|rno-miR-152-3p
NM_001025649	1	rno-miR-181a-5p
NM_133394	1	rno-miR-181a-5p
NM_019185	1	rno-miR-181a-5p
NM_001103360	2	rno-miR-181a-5p|rno-miR-30c-5p

NM_017043	1	rno-miR-181a-5p
NM_001107507	3	rno-miR-181a-5p|rno-miR-17-5p|rno-miR-93-5p
NM_012942	1	rno-miR-181a-5p
NM_001105750	2	rno-miR-181a-5p|rno-miR-30c-5p
NM_001037205	1	rno-miR-181a-5p
NM_001014785	1	rno-miR-181a-5p
NM_001191632	4	rno-miR-181a-5p|rno-miR-17-5p|rno-miR-152-3p|rno-miR-93-5p
NM_001110365	1	rno-miR-181a-5p
NM_053311	1	rno-miR-181a-5p
NM_182473	1	rno-miR-181a-5p
NM_001134754	1	rno-miR-181a-5p
NM_001107002	2	rno-miR-181a-5p|rno-miR-30c-5p
NM_198750	1	rno-miR-181a-5p
NM_001109135	2	rno-miR-181a-5p|rno-miR-30c-5p
NM_001105801	3	rno-miR-181a-5p|rno-miR-17-5p|rno-miR-93-5p
NM_001101828	1	rno-miR-181a-5p
NM_001025749	1	rno-miR-181a-5p
NM_001007754	1	rno-miR-181a-5p
NM_001025051	1	rno-miR-181a-5p
NM_031828	1	rno-miR-181a-5p
NM_001271143	1	rno-miR-181a-5p
NM_021702	2	rno-miR-181a-5p|rno-miR-31a-5p
NM_001302967	1	rno-miR-181a-5p
NM_001008299	2	rno-miR-181a-5p|rno-miR-30c-5p
NM_001109456	1	rno-miR-181a-5p
NM_024367	1	rno-miR-181a-5p
NM_017111	1	rno-miR-181a-5p
NM_012600	2	rno-miR-181a-5p|rno-miR-30c-5p
NM_134327	3	rno-miR-181a-5p|rno-miR-17-5p|rno-miR-93-5p
NM_001108115	1	rno-miR-181a-5p

NM_001106949	2	rno-miR-181a-5p|rno-miR-30c-5p
NM_139324	1	rno-miR-181a-5p
NM_053536	1	rno-miR-181a-5p
NM_001191094	2	rno-miR-181a-5p|rno-miR-152-3p
NM_001106356	2	rno-miR-181a-5p|rno-miR-30c-5p
NM_031510	3	rno-miR-181a-5p|rno-miR-30c-5p|rno-miR-152-3p
NM_001134421	1	rno-miR-181a-5p
NM_001108902	2	rno-miR-181a-5p|rno-miR-30c-5p
NM_001035007	1	rno-miR-181a-5p
NM_001106575	1	rno-miR-181a-5p
NM_001106320	1	rno-miR-181a-5p
NM_001107370	1	rno-miR-181a-5p
NM_017091	1	rno-miR-181a-5p
NM_031628	3	rno-miR-181a-5p|rno-miR-17-5p|rno-miR-93-5p
NM_032990	1	rno-miR-181a-5p
NM_001191743	1	rno-miR-181a-5p
NM_001106913	1	rno-miR-181a-5p
NM_001134864	1	rno-miR-181a-5p
NM_001108760	1	rno-miR-181a-5p
NM_001100562	1	rno-miR-181a-5p
NM_001108392	1	rno-miR-181a-5p
NM_001108535	1	rno-miR-181a-5p
NM_130417	1	rno-miR-181a-5p
NM_001003404	1	rno-miR-181a-5p
NM_012881	1	rno-miR-181a-5p
NM_001108814	1	rno-miR-181a-5p
NM_012705	1	rno-miR-181a-5p
NM_001025751	1	rno-miR-181a-5p
NM_001106502	1	rno-miR-181a-5p
NM_001108087	1	rno-miR-181a-5p

NM_001024358	1	rno-miR-181a-5p
NM_001039035	2	rno-miR-181a-5p|rno-miR-30c-5p
NM_001108159	1	rno-miR-181a-5p
NM_001108202	1	rno-miR-181a-5p
NM_001107384	1	rno-miR-181a-5p
NM_001105887	1	rno-miR-181a-5p
NM_017345	1	rno-miR-181a-5p
NM_001107564	1	rno-miR-181a-5p
NM_001106349	1	rno-miR-181a-5p
NM_001014107	4	rno-miR-181a-5p|rno-miR-17-5p|rno-miR-152-3p|rno-miR-93-5p
NM_001014220	1	rno-miR-181a-5p
NM_023092	1	rno-miR-181a-5p
NM_001008335	1	rno-miR-181a-5p
NM_001107232	1	rno-miR-31a-5p
NM_001287578	1	rno-miR-31a-5p
NM_001191844	1	rno-miR-31a-5p
NM_001107241	1	rno-miR-31a-5p
NM_001106958	1	rno-miR-31a-5p
NM_001170576	2	rno-miR-31a-5p|rno-miR-30c-5p
NM_001191628	1	rno-miR-31a-5p
NM_001034007	1	rno-miR-31a-5p
NM_031785	1	rno-miR-31a-5p
NM_022587	1	rno-miR-31a-5p
NM_017054	1	rno-miR-31a-5p
NM_030989	1	rno-miR-31a-5p
NM_001108924	1	rno-miR-31a-5p
NM_001105745	1	rno-miR-31a-5p
NM_001106090	1	rno-miR-31a-5p
NM_001039034	1	rno-miR-31a-5p
NM_001115034	1	rno-miR-31a-5p

NM_001270981	1	rno-miR-31a-5p
NM_172040	1	rno-miR-31a-5p
NM_001305159	1	rno-miR-31a-5p
NM_022249	1	rno-miR-31a-5p
NM_181382	1	rno-miR-31a-5p
NM_207615	2	rno-miR-31a-5p|rno-miR-30c-5p
NM_001014267	1	rno-miR-31a-5p
NM_001106865	1	rno-miR-31a-5p
NM_001007745	1	rno-miR-31a-5p
NM_001108132	1	rno-miR-31a-5p
NM_001109083	1	rno-miR-31a-5p
NM_173305	1	rno-miR-31a-5p
NM_053856	1	rno-miR-31a-5p
NM_001013432	1	rno-miR-31a-5p
NM_001329200	1	rno-miR-31a-5p
NM_001024267	1	rno-miR-31a-5p
NM_001271032	1	rno-miR-31a-5p
NM_001113754	1	rno-miR-31a-5p
NM_001034011	1	rno-miR-31a-5p
NM_001014116	2	rno-miR-31a-5p|rno-miR-152-3p
NM_001100470	1	rno-miR-31a-5p
NM_001012074	1	rno-miR-31a-5p
NM_001107941	1	rno-miR-31a-5p
NM_012948	1	rno-miR-31a-5p
NM_001170336	1	rno-miR-31a-5p
NM_012524	1	rno-miR-31a-5p
NM_001171981	1	rno-miR-31a-5p
NM_001100684	3	rno-miR-31a-5p|rno-miR-17-5p|rno-miR-93-5p
NM_001080151	1	rno-miR-31a-5p
NM_001108116	1	rno-miR-31a-5p

NM_001025648	2	rno-miR-31a-5p|rno-miR-30c-5p
NM_024382	1	rno-miR-31a-5p
NM_001127550	1	rno-miR-31a-5p
NM_001109386	2	rno-miR-31a-5p|rno-miR-152-3p
NM_001037780	1	rno-miR-31a-5p
NM_001108098	1	rno-miR-31a-5p
NM_001105981	2	rno-miR-31a-5p|rno-miR-152-3p
NM_022198	1	rno-miR-31a-5p
NM_001108250	1	rno-miR-31a-5p
NM_134345	1	rno-miR-31a-5p
NM_171990	1	rno-miR-31a-5p
NM_001013084	1	rno-miR-31a-5p
NM_022177	3	rno-miR-31a-5p|rno-miR-17-5p|rno-miR-93-5p
NM_001113749	1	rno-miR-31a-5p
NM_001135836	1	rno-miR-31a-5p
NM_001105819	1	rno-miR-31a-5p
NM_001007692	1	rno-miR-31a-5p
NM_012809	1	rno-miR-31a-5p
NM_001287579	1	rno-miR-31a-5p
NM_001106259	1	rno-miR-31a-5p
NM_001014177	1	rno-miR-31a-5p
NM_001107240	1	rno-miR-31a-5p
NM_133385	1	rno-miR-31a-5p
NM_001107586	3	rno-miR-31a-5p|rno-miR-17-5p|rno-miR-93-5p
NM_001108632	1	rno-miR-31a-5p
NM_001008386	1	rno-miR-31a-5p
NM_001163736	3	rno-miR-31a-5p|rno-miR-17-5p|rno-miR-93-5p
NM_198738	1	rno-miR-31a-5p
NM_001108864	4	rno-miR-31a-5p|rno-miR-30c-5p|rno-miR-17-5p|rno-miR-93-5p
NM_001106988	1	rno-miR-31a-5p

NM_001007746	1	rno-miR-31a-5p
NM_001253859	1	rno-miR-31a-5p
NM_001109554	1	rno-miR-31a-5p
NM_001109537	1	rno-miR-31a-5p
NM_053366	2	rno-miR-31a-5p|rno-miR-15b-5p
NM_001025766	1	rno-miR-31a-5p
NM_022701	1	rno-miR-31a-5p
NM_001170472	1	rno-miR-31a-5p
NM_001108752	1	rno-miR-31a-5p
NM_001034008	1	rno-miR-31a-5p
NM_001107040	1	rno-miR-31a-5p
NM_001109547	1	rno-miR-31a-5p
NM_001108848	1	rno-miR-31a-5p
NM_001025004	1	rno-miR-31a-5p
NM_053871	1	rno-miR-31a-5p
NM_001033679	1	rno-miR-31a-5p
NM_001012084	1	rno-miR-31a-5p
NM_001108194	1	rno-miR-31a-5p
NM_001108080	1	rno-miR-31a-5p
NM_001109066	1	rno-miR-31a-5p
NM_001106285	1	rno-miR-31a-5p
NM_001108968	1	rno-miR-31a-5p
NM_001009391	1	rno-miR-31a-5p
NM_134466	1	rno-miR-31a-5p
NM_001109515	1	rno-miR-31a-5p
NM_138922	1	rno-miR-31a-5p
NM_001105766	1	rno-miR-31a-5p
NM_057192	2	rno-miR-31a-5p|rno-miR-30c-5p
NM_012641	1	rno-miR-31a-5p
NM_031537	1	rno-miR-31a-5p

NM_001287577	1	rno-miR-31a-5p
NM_022712	1	rno-miR-31a-5p
NM_001080380	1	rno-miR-31a-5p
NM_001007150	1	rno-miR-31a-5p
NM_053693	3	rno-miR-30c-5p|rno-miR-17-5p|rno-miR-93-5p
NM_001108272	1	rno-miR-30c-5p
NM_019206	1	rno-miR-30c-5p
NM_053951	1	rno-miR-30c-5p
NM_001106547	1	rno-miR-30c-5p
NM_053836	1	rno-miR-30c-5p
NM_001191782	1	rno-miR-30c-5p
NM_001108284	1	rno-miR-30c-5p
NM_001134882	1	rno-miR-30c-5p
NM_012984	1	rno-miR-30c-5p
NM_001309453	1	rno-miR-30c-5p
NM_001127488	1	rno-miR-30c-5p
NM_001191985	1	rno-miR-30c-5p
NM_053407	1	rno-miR-30c-5p
NM_001100908	1	rno-miR-30c-5p
NM_053754	1	rno-miR-30c-5p
NM_001109231	1	rno-miR-30c-5p
NM_022386	1	rno-miR-30c-5p
NM_031143	1	rno-miR-30c-5p
NM_019187	1	rno-miR-30c-5p
NM_001109172	1	rno-miR-30c-5p
NM_019277	1	rno-miR-30c-5p
NM_001109026	1	rno-miR-30c-5p
NM_001024273	1	rno-miR-30c-5p
NM_031520	1	rno-miR-30c-5p
NM_001191678	1	rno-miR-30c-5p

NM_017333	1	rno-miR-30c-5p
NM_145879	1	rno-miR-30c-5p
NM_001108029	1	rno-miR-30c-5p
NM_138846	1	rno-miR-30c-5p
NM_001014139	1	rno-miR-30c-5p
NM_001134554	1	rno-miR-30c-5p
NM_172085	2	rno-miR-30c-5p|rno-miR-152-3p
NM_001113740	1	rno-miR-30c-5p
NM_001011992	1	rno-miR-30c-5p
NM_013177	1	rno-miR-30c-5p
NM_001108714	1	rno-miR-30c-5p
NM_001106452	1	rno-miR-30c-5p
NM_031672	1	rno-miR-30c-5p
NM_001108737	3	rno-miR-30c-5p|rno-miR-17-5p|rno-miR-93-5p
NM_001169152	3	rno-miR-30c-5p|rno-miR-17-5p|rno-miR-93-5p
NM_024483	1	rno-miR-30c-5p
NM_139101	1	rno-miR-30c-5p
NM_001109080	1	rno-miR-30c-5p
NM_001108826	1	rno-miR-30c-5p
NM_001008338	1	rno-miR-30c-5p
NM_001191551	1	rno-miR-30c-5p
NM_001100823	1	rno-miR-30c-5p
NM_001170343	1	rno-miR-30c-5p
NM_001170327	1	rno-miR-30c-5p
NM_001025009	1	rno-miR-30c-5p
NM_001025718	1	rno-miR-30c-5p
NM_001047854	1	rno-miR-30c-5p
NM_012519	1	rno-miR-30c-5p
NM_001106590	1	rno-miR-30c-5p
NM_001106457	1	rno-miR-30c-5p

NM_030992	1	rno-miR-30c-5p
NM_172062	1	rno-miR-30c-5p
NM_001100895	1	rno-miR-30c-5p
NM_053660	1	rno-miR-30c-5p
NM_001107280	1	rno-miR-30c-5p
NM_053818	3	rno-miR-30c-5p|rno-miR-17-5p|rno-miR-93-5p
NM_001135903	1	rno-miR-30c-5p
NM_001108630	1	rno-miR-30c-5p
NM_001106397	1	rno-miR-30c-5p
NM_001131002	1	rno-miR-30c-5p
NM_031082	1	rno-miR-30c-5p
NM_031566	1	rno-miR-30c-5p
NM_001106822	1	rno-miR-30c-5p
NM_001108017	1	rno-miR-30c-5p
NM_001134867	1	rno-miR-30c-5p
NM_001107783	1	rno-miR-30c-5p
NM_024151	1	rno-miR-30c-5p
NM_001025143	1	rno-miR-30c-5p
NM_001109190	1	rno-miR-30c-5p
NM_001163063	1	rno-miR-30c-5p
NM_021668	1	rno-miR-30c-5p
NM_001134879	3	rno-miR-30c-5p|rno-miR-17-5p|rno-miR-93-5p
NM_001008345	1	rno-miR-30c-5p
NM_001108439	1	rno-miR-30c-5p
NM_053467	1	rno-miR-30c-5p
NM_022937	1	rno-miR-30c-5p
NM_001013995	1	rno-miR-30c-5p
NM_001100704	1	rno-miR-30c-5p
NM_001271332	1	rno-miR-30c-5p
NM_001108608	2	rno-miR-30c-5p|rno-miR-152-3p

NM_001034926	1	rno-miR-30c-5p
NM_199396	1	rno-miR-30c-5p
NM_001012469	1	rno-miR-30c-5p
NM_001271066	1	rno-miR-30c-5p
NM_031140	1	rno-miR-30c-5p
NM_001108560	1	rno-miR-30c-5p
NM_001008308	1	rno-miR-30c-5p
NM_001024980	1	rno-miR-30c-5p
NM_001082574	1	rno-miR-30c-5p
NM_022616	1	rno-miR-30c-5p
NM_053570	1	rno-miR-30c-5p
NM_001198804	3	rno-miR-30c-5p|rno-miR-17-5p|rno-miR-93-5p
NM_001108691	1	rno-miR-30c-5p
NM_001037655	1	rno-miR-30c-5p
NM_133425	1	rno-miR-30c-5p
NM_053471	1	rno-miR-30c-5p
NM_019286	1	rno-miR-30c-5p
NM_001107973	1	rno-miR-30c-5p
NM_053890	1	rno-miR-30c-5p
NM_031775	1	rno-miR-30c-5p
NM_001108400	1	rno-miR-30c-5p
NM_001107583	1	rno-miR-30c-5p
NM_031315	1	rno-miR-30c-5p
NM_207595	1	rno-miR-30c-5p
NM_022705	1	rno-miR-30c-5p
NM_001166351	1	rno-miR-30c-5p
NM_001106578	3	rno-miR-30c-5p|rno-miR-17-5p|rno-miR-93-5p
NM_030857	1	rno-miR-30c-5p
NM_012884	1	rno-miR-30c-5p
NM_030873	3	rno-miR-30c-5p|rno-miR-17-5p|rno-miR-93-5p

NM_001100488	1	rno-miR-30c-5p
NM_022236	1	rno-miR-30c-5p
NM_001006999	1	rno-miR-30c-5p
NM_053414	1	rno-miR-30c-5p
NM_001131000	1	rno-miR-30c-5p
NM_001107192	3	rno-miR-30c-5p|rno-miR-17-5p|rno-miR-93-5p
NM_001011955	1	rno-miR-30c-5p
NM_001106916	1	rno-miR-30c-5p
NM_001107056	1	rno-miR-30c-5p
NM_001191904	1	rno-miR-30c-5p
NM_031563	1	rno-miR-30c-5p
NM_001134798	1	rno-miR-30c-5p
NM_001106364	3	rno-miR-30c-5p|rno-miR-17-5p|rno-miR-93-5p
NM_001100565	1	rno-miR-30c-5p
NM_013119	1	rno-miR-30c-5p
NM_017295	1	rno-miR-30c-5p
NM_001079891	1	rno-miR-30c-5p
NM_001170594	1	rno-miR-30c-5p
NM_012549	1	rno-miR-30c-5p
NM_212501	1	rno-miR-30c-5p
NM_001135003	1	rno-miR-30c-5p
NM_001108213	2	rno-miR-30c-5p|rno-miR-152-3p
NM_001083811	3	rno-miR-30c-5p|rno-miR-17-5p|rno-miR-93-5p
NM_021576	1	rno-miR-30c-5p
NM_001106371	1	rno-miR-30c-5p
NM_139183	1	rno-miR-30c-5p
NM_001014126	1	rno-miR-30c-5p
NM_001047899	1	rno-miR-30c-5p
NM_173327	1	rno-miR-30c-5p
NM_053726	1	rno-miR-30c-5p

NM_134396	1	rno-miR-30c-5p
NM_021682	1	rno-miR-30c-5p
NM_001107141	1	rno-miR-30c-5p
NM_133398	1	rno-miR-30c-5p
NM_001009714	1	rno-miR-30c-5p
NM_001008341	1	rno-miR-30c-5p
NM_001008284	1	rno-miR-30c-5p
NM_001134636	1	rno-miR-30c-5p
NM_001108308	1	rno-miR-30c-5p
NM_001107400	1	rno-miR-30c-5p
NM_001025651	1	rno-miR-30c-5p
NM_001105712	1	rno-miR-30c-5p
NM_023969	1	rno-miR-30c-5p
NM_001109630	1	rno-miR-30c-5p
NM_012732	2	rno-miR-30c-5p|rno-miR-152-3p
NM_001111098	1	rno-miR-30c-5p
NM_001107515	1	rno-miR-30c-5p
NM_022588	1	rno-miR-30c-5p
NM_001130939	1	rno-miR-30c-5p
NM_001134436	1	rno-miR-30c-5p
NM_198749	1	rno-miR-30c-5p
NM_001107933	1	rno-miR-30c-5p
NM_001011927	1	rno-miR-30c-5p
NM_001135250	1	rno-miR-30c-5p
NM_001107257	1	rno-miR-30c-5p
NM_001100667	1	rno-miR-30c-5p
NM_001144859	1	rno-miR-30c-5p
NM_001033683	1	rno-miR-30c-5p
NM_001105899	1	rno-miR-30c-5p
NM_139084	1	rno-miR-30c-5p

NM_001106347	1	rno-miR-30c-5p
NM_031035	1	rno-miR-30c-5p
NM_017149	2	rno-miR-30c-5p|rno-miR-152-3p
NM_001134559	1	rno-miR-30c-5p
NM_019266	1	rno-miR-30c-5p
NM_001106778	1	rno-miR-30c-5p
NM_001025155	1	rno-miR-30c-5p
NM_001004279	1	rno-miR-30c-5p
NM_001108966	3	rno-miR-30c-5p|rno-miR-17-5p|rno-miR-93-5p
NM_001109468	3	rno-miR-30c-5p|rno-miR-17-5p|rno-miR-93-5p
NM_001191707	1	rno-miR-30c-5p
NM_021776	1	rno-miR-30c-5p
NM_001100852	1	rno-miR-30c-5p
NM_134461	1	rno-miR-30c-5p
NM_053338	1	rno-miR-30c-5p
NM_001271067	1	rno-miR-30c-5p
NM_001305184	1	rno-miR-30c-5p
NM_057124	1	rno-miR-30c-5p
NM_022396	1	rno-miR-30c-5p
NM_001170600	1	rno-miR-30c-5p
NM_019178	1	rno-miR-30c-5p
NM_013170	1	rno-miR-30c-5p
NM_001134762	1	rno-miR-30c-5p
NM_001108879	1	rno-miR-30c-5p
NM_001107426	1	rno-miR-30c-5p
NM_001107309	3	rno-miR-30c-5p|rno-miR-17-5p|rno-miR-93-5p
NM_001107522	1	rno-miR-30c-5p
NM_001107169	1	rno-miR-30c-5p
NM_053998	1	rno-miR-30c-5p
NM_001108944	3	rno-miR-30c-5p|rno-miR-17-5p|rno-miR-93-5p

NM_001106942	1	rno-miR-30c-5p
NM_001270954	3	rno-miR-30c-5p|rno-miR-17-5p|rno-miR-93-5p
NM_001135249	1	rno-miR-30c-5p
NM_134337	1	rno-miR-30c-5p
NM_001024236	1	rno-miR-30c-5p
NM_001017473	1	rno-miR-30c-5p
NM_019183	1	rno-miR-30c-5p
NM_001024341	1	rno-miR-30c-5p
NM_019329	1	rno-miR-30c-5p
NM_001039341	2	rno-miR-30c-5p|rno-miR-152-3p
NM_001107727	1	rno-miR-30c-5p
NM_001007720	1	rno-miR-30c-5p
NM_001106642	1	rno-miR-30c-5p
NM_001271984	1	rno-miR-30c-5p
NM_053981	1	rno-miR-30c-5p
NM_001107404	1	rno-miR-30c-5p
NM_012956	1	rno-miR-30c-5p
NM_017261	3	rno-miR-30c-5p|rno-miR-17-5p|rno-miR-93-5p
NM_001100850	1	rno-miR-30c-5p
NM_001106235	1	rno-miR-30c-5p
NM_001106520	1	rno-miR-30c-5p
NM_183331	1	rno-miR-30c-5p
NM_001106319	1	rno-miR-30c-5p
NM_053921	1	rno-miR-30c-5p
NM_012815	1	rno-miR-30c-5p
NM_001134796	1	rno-miR-30c-5p
NM_053920	2	rno-miR-17-5p|rno-miR-93-5p
NM_001014037	2	rno-miR-17-5p|rno-miR-93-5p
NM_024395	2	rno-miR-17-5p|rno-miR-93-5p
NM_001107369	2	rno-miR-17-5p|rno-miR-93-5p

NM_001037209	2	rno-miR-17-5p|rno-miR-93-5p
NM_001109003	2	rno-miR-17-5p|rno-miR-93-5p
NM_001017960	2	rno-miR-17-5p|rno-miR-93-5p
NM_001039018	2	rno-miR-17-5p|rno-miR-93-5p
NM_001134541	2	rno-miR-17-5p|rno-miR-93-5p
NM_024359	2	rno-miR-17-5p|rno-miR-93-5p
NM_001106022	2	rno-miR-17-5p|rno-miR-93-5p
NM_001106392	2	rno-miR-17-5p|rno-miR-93-5p
NM_001031651	2	rno-miR-17-5p|rno-miR-93-5p
NM_001191934	2	rno-miR-17-5p|rno-miR-93-5p
NM_080782	2	rno-miR-17-5p|rno-miR-93-5p
NM_033653	2	rno-miR-17-5p|rno-miR-93-5p
NM_001008293	2	rno-miR-17-5p|rno-miR-93-5p
NM_001013048	2	rno-miR-17-5p|rno-miR-93-5p
NM_031356	2	rno-miR-17-5p|rno-miR-93-5p
NM_001004229	2	rno-miR-17-5p|rno-miR-93-5p
NM_022702	2	rno-miR-17-5p|rno-miR-93-5p
NM_207617	2	rno-miR-17-5p|rno-miR-93-5p
NM_001108889	2	rno-miR-17-5p|rno-miR-93-5p
NM_001012182	2	rno-miR-17-5p|rno-miR-93-5p
NM_001108069	2	rno-miR-17-5p|rno-miR-93-5p
NM_001105970	2	rno-miR-17-5p|rno-miR-93-5p
NM_031609	2	rno-miR-17-5p|rno-miR-93-5p
NM_001106174	2	rno-miR-17-5p|rno-miR-93-5p
NM_001107222	2	rno-miR-17-5p|rno-miR-93-5p
NM_001025693	2	rno-miR-17-5p|rno-miR-93-5p
NM_019371	2	rno-miR-17-5p|rno-miR-93-5p
NM_001009677	1	rno-miR-17-5p
NM_031094	2	rno-miR-17-5p|rno-miR-93-5p
NM_001013241	2	rno-miR-17-5p|rno-miR-93-5p

NM_030835	2	rno-miR-17-5p|rno-miR-93-5p
NM_001109271	2	rno-miR-17-5p|rno-miR-93-5p
NM_001109371	2	rno-miR-17-5p|rno-miR-93-5p
NM_001109455	2	rno-miR-17-5p|rno-miR-93-5p
NM_001108147	2	rno-miR-17-5p|rno-miR-93-5p
NM_001014231	2	rno-miR-17-5p|rno-miR-93-5p
NM_001109546	2	rno-miR-17-5p|rno-miR-93-5p
NM_012636	2	rno-miR-17-5p|rno-miR-93-5p
NM_001014242	2	rno-miR-17-5p|rno-miR-93-5p
NM_001270763	2	rno-miR-17-5p|rno-miR-93-5p
NM_001107837	2	rno-miR-17-5p|rno-miR-93-5p
NM_001107133	2	rno-miR-17-5p|rno-miR-93-5p
NM_001106553	2	rno-miR-17-5p|rno-miR-93-5p
NM_001130564	2	rno-miR-17-5p|rno-miR-93-5p
NM_001191805	2	rno-miR-17-5p|rno-miR-93-5p
NM_001107761	2	rno-miR-17-5p|rno-miR-93-5p
NM_001007698	3	rno-miR-17-5p|rno-miR-151-5p|rno-miR-93-5p
NM_020538	2	rno-miR-17-5p|rno-miR-93-5p
NM_001191888	2	rno-miR-17-5p|rno-miR-93-5p
NM_001106543	2	rno-miR-17-5p|rno-miR-93-5p
NM_001107406	2	rno-miR-17-5p|rno-miR-93-5p
NM_001100872	3	rno-miR-17-5p|rno-miR-152-3p|rno-miR-93-5p
NM_130416	1	rno-miR-17-5p
NM_021698	2	rno-miR-17-5p|rno-miR-93-5p
NM_001107344	2	rno-miR-17-5p|rno-miR-93-5p
NM_001107263	2	rno-miR-17-5p|rno-miR-93-5p
NM_001135718	2	rno-miR-17-5p|rno-miR-93-5p
NM_001108696	2	rno-miR-17-5p|rno-miR-93-5p
NM_001108863	2	rno-miR-17-5p|rno-miR-93-5p
NM_001039344	2	rno-miR-17-5p|rno-miR-93-5p

NM_001008767	2	rno-miR-17-5p|rno-miR-93-5p
NM_133315	2	rno-miR-17-5p|rno-miR-93-5p
NM_001191615	2	rno-miR-17-5p|rno-miR-93-5p
NM_001191951	2	rno-miR-17-5p|rno-miR-93-5p
NM_053396	2	rno-miR-17-5p|rno-miR-93-5p
NM_001271305	2	rno-miR-17-5p|rno-miR-93-5p
NM_057121	2	rno-miR-17-5p|rno-miR-93-5p
NM_031007	3	rno-miR-17-5p|rno-miR-152-3p|rno-miR-93-5p
NM_022686	2	rno-miR-17-5p|rno-miR-93-5p
NM_022954	2	rno-miR-17-5p|rno-miR-93-5p
NM_001044259	2	rno-miR-17-5p|rno-miR-93-5p
NM_001107252	2	rno-miR-17-5p|rno-miR-93-5p
NM_053686	2	rno-miR-17-5p|rno-miR-93-5p
NM_001271488	2	rno-miR-17-5p|rno-miR-93-5p
NM_001109885	2	rno-miR-17-5p|rno-miR-93-5p
NM_001024903	2	rno-miR-17-5p|rno-miR-93-5p
NM_001107667	2	rno-miR-17-5p|rno-miR-93-5p
NM_001108892	2	rno-miR-17-5p|rno-miR-93-5p
NM_001108809	2	rno-miR-17-5p|rno-miR-93-5p
NM_001108510	2	rno-miR-17-5p|rno-miR-93-5p
NM_080480	2	rno-miR-17-5p|rno-miR-93-5p
NM_001012460	2	rno-miR-17-5p|rno-miR-93-5p
NM_022596	2	rno-miR-17-5p|rno-miR-93-5p
NM_001013223	2	rno-miR-17-5p|rno-miR-93-5p
NM_001163156	2	rno-miR-17-5p|rno-miR-93-5p
NM_001005876	2	rno-miR-17-5p|rno-miR-93-5p
NM_080407	2	rno-miR-17-5p|rno-miR-93-5p
NM_001107131	2	rno-miR-17-5p|rno-miR-93-5p
NM_001013869	2	rno-miR-17-5p|rno-miR-93-5p
NM_001106752	2	rno-miR-17-5p|rno-miR-93-5p

NM_001106783	2	rno-miR-17-5p|rno-miR-93-5p
NM_001108855	2	rno-miR-17-5p|rno-miR-93-5p
NM_031713	2	rno-miR-17-5p|rno-miR-93-5p
NM_001108108	2	rno-miR-17-5p|rno-miR-93-5p
NM_031786	2	rno-miR-17-5p|rno-miR-93-5p
NM_138517	2	rno-miR-17-5p|rno-miR-93-5p
NM_001037656	2	rno-miR-17-5p|rno-miR-93-5p
NM_001108517	2	rno-miR-17-5p|rno-miR-93-5p
NM_001107180	2	rno-miR-17-5p|rno-miR-93-5p
NM_012983	2	rno-miR-17-5p|rno-miR-93-5p
NM_001025771	2	rno-miR-17-5p|rno-miR-93-5p
NM_001012139	2	rno-miR-17-5p|rno-miR-93-5p
NM_001013144	2	rno-miR-17-5p|rno-miR-93-5p
NM_001109584	2	rno-miR-17-5p|rno-miR-93-5p
NM_031615	2	rno-miR-17-5p|rno-miR-93-5p
NM_001014056	2	rno-miR-17-5p|rno-miR-93-5p
NM_031016	2	rno-miR-17-5p|rno-miR-93-5p
NM_001011926	2	rno-miR-17-5p|rno-miR-93-5p
NM_001037768	2	rno-miR-17-5p|rno-miR-93-5p
NM_138503	2	rno-miR-17-5p|rno-miR-93-5p
NM_001191634	2	rno-miR-17-5p|rno-miR-93-5p
NM_001044282	2	rno-miR-17-5p|rno-miR-93-5p
NM_001100634	2	rno-miR-17-5p|rno-miR-93-5p
NM_001109469	2	rno-miR-17-5p|rno-miR-93-5p
NM_001106409	2	rno-miR-17-5p|rno-miR-93-5p
NM_001014193	2	rno-miR-17-5p|rno-miR-93-5p
NM_001108720	3	rno-miR-17-5p|rno-miR-152-3p|rno-miR-93-5p
NM_001135897	2	rno-miR-17-5p|rno-miR-93-5p
NM_001191996	2	rno-miR-17-5p|rno-miR-93-5p
NM_053795	2	rno-miR-17-5p|rno-miR-93-5p

NM_001191705	2	rno-miR-17-5p|rno-miR-93-5p
NM_181628	2	rno-miR-17-5p|rno-miR-93-5p
NM_130894	2	rno-miR-17-5p|rno-miR-93-5p
NM_001024886	2	rno-miR-17-5p|rno-miR-93-5p
NM_031987	2	rno-miR-17-5p|rno-miR-93-5p
NM_001108990	2	rno-miR-17-5p|rno-miR-93-5p
NM_019207	2	rno-miR-17-5p|rno-miR-93-5p
NM_001170468	2	rno-miR-17-5p|rno-miR-93-5p
NM_001106461	2	rno-miR-17-5p|rno-miR-93-5p
NM_001002830	2	rno-miR-17-5p|rno-miR-93-5p
NM_001014160	2	rno-miR-17-5p|rno-miR-93-5p
NM_001134731	2	rno-miR-17-5p|rno-miR-93-5p
NM_175843	2	rno-miR-17-5p|rno-miR-93-5p
NM_134370	2	rno-miR-17-5p|rno-miR-93-5p
NM_139105	2	rno-miR-17-5p|rno-miR-93-5p
NM_001191110	2	rno-miR-17-5p|rno-miR-93-5p
NM_001271132	2	rno-miR-17-5p|rno-miR-93-5p
NM_001126372	2	rno-miR-17-5p|rno-miR-93-5p
NM_001013074	2	rno-miR-17-5p|rno-miR-93-5p
NM_001039099	2	rno-miR-17-5p|rno-miR-93-5p
NM_001009492	2	rno-miR-17-5p|rno-miR-93-5p
NM_134332	2	rno-miR-17-5p|rno-miR-93-5p
NM_001271205	2	rno-miR-17-5p|rno-miR-93-5p
NM_001191564	2	rno-miR-17-5p|rno-miR-93-5p
NM_001270762	2	rno-miR-17-5p|rno-miR-93-5p
NM_012727	2	rno-miR-17-5p|rno-miR-93-5p
NM_019290	2	rno-miR-17-5p|rno-miR-93-5p
NM_001015024	2	rno-miR-17-5p|rno-miR-93-5p
NM_001025042	2	rno-miR-17-5p|rno-miR-93-5p
NM_001107934	1	rno-miR-17-5p

NM_031665	2	rno-miR-17-5p|rno-miR-93-5p
NM_199384	2	rno-miR-17-5p|rno-miR-93-5p
NM_057211	2	rno-miR-17-5p|rno-miR-93-5p
NM_001014007	2	rno-miR-17-5p|rno-miR-93-5p
NM_024397	2	rno-miR-17-5p|rno-miR-93-5p
NM_001106731	2	rno-miR-17-5p|rno-miR-93-5p
NM_001107582	2	rno-miR-17-5p|rno-miR-93-5p
NM_001106651	2	rno-miR-17-5p|rno-miR-93-5p
NM_001044770	2	rno-miR-17-5p|rno-miR-93-5p
NM_053962	2	rno-miR-17-5p|rno-miR-93-5p
NM_001024247	2	rno-miR-17-5p|rno-miR-93-5p
NM_001106366	2	rno-miR-17-5p|rno-miR-93-5p
NM_001144991	2	rno-miR-17-5p|rno-miR-93-5p
NM_133392	2	rno-miR-17-5p|rno-miR-93-5p
NM_001038992	1	rno-miR-152-3p
NM_001106976	1	rno-miR-152-3p
NM_053562	1	rno-miR-152-3p
NM_013167	1	rno-miR-152-3p
NM_001134749	1	rno-miR-152-3p
NM_001305172	1	rno-miR-152-3p
NM_001107208	1	rno-miR-152-3p
NM_001007701	1	rno-miR-152-3p
NM_001005547	1	rno-miR-152-3p
NM_001109295	1	rno-miR-152-3p
NM_001014085	1	rno-miR-152-3p
NM_001127636	1	rno-miR-152-3p
NM_001012140	1	rno-miR-152-3p
NM_012620	1	rno-miR-152-3p
NM_001108485	1	rno-miR-152-3p
NM_001037327	1	rno-miR-152-3p

NM_001100474	1	rno-miR-152-3p
NM_013029	1	rno-miR-152-3p
NM_001305628	1	rno-miR-152-3p
NM_001034164	1	rno-miR-152-3p
NM_001039196	1	rno-miR-152-3p
NM_139088	1	rno-miR-152-3p
NM_001134956	1	rno-miR-152-3p
NM_022715	1	rno-miR-152-3p
NM_001034108	1	rno-miR-152-3p
NM_134364	1	rno-miR-152-3p
NM_017105	1	rno-miR-152-3p
NM_001106168	1	rno-miR-152-3p
NM_031584	1	rno-miR-152-3p
NM_001013122	1	rno-miR-152-3p
NM_001108150	1	rno-miR-152-3p
NM_001029899	1	rno-miR-152-3p
NM_001109522	1	rno-miR-152-3p
NM_139115	1	rno-miR-152-3p
NM_053354	1	rno-miR-152-3p
NM_001013158	1	rno-miR-152-3p
NM_001109292	1	rno-miR-152-3p
NM_001134800	1	rno-miR-152-3p
NM_001079889	1	rno-miR-152-3p
NM_001025402	1	rno-miR-152-3p
NM_001191633	1	rno-miR-152-3p
NM_001100846	1	rno-miR-152-3p
NM_133552	1	rno-miR-152-3p
NM_001012150	1	rno-miR-152-3p
NM_001108606	1	rno-miR-152-3p
NM_001077666	1	rno-miR-152-3p

NM_001145021	1	rno-miR-152-3p
NM_017319	1	rno-miR-152-3p
NM_001108277	1	rno-miR-152-3p
NM_001143894	1	rno-miR-152-3p
NM_001135813	1	rno-miR-152-3p
NM_053771	1	rno-miR-152-3p
NM_138532	1	rno-miR-152-3p
NM_001107806	1	rno-miR-152-3p
NM_019281	1	rno-miR-152-3p
NM_199383	1	rno-miR-152-3p
NM_133604	1	rno-miR-152-3p
NM_001107437	1	rno-miR-152-3p
NM_001135849	1	rno-miR-152-3p
NM_001038994	1	rno-miR-152-3p
NM_001276430	1	rno-miR-152-3p
NM_001014017	1	rno-miR-152-3p
NM_016996	1	rno-miR-152-3p
NM_134467	1	rno-miR-152-3p
NM_013165	1	rno-miR-152-3p
NM_001107862	1	rno-miR-152-3p
NM_012532	1	rno-miR-152-3p
NM_001130989	1	rno-miR-152-3p
NM_001271103	1	rno-miR-152-3p
NM_001270390	1	rno-miR-152-3p
NM_001109121	1	rno-miR-152-3p
NM_031753	1	rno-miR-152-3p
NM_001014215	1	rno-miR-152-3p
NM_001009710	1	rno-miR-152-3p
NM_013066	1	rno-miR-152-3p
NM_001025001	1	rno-miR-152-3p

NM_031540	1	rno-miR-152-3p
NM_182842	1	rno-miR-152-3p
NM_001109430	1	rno-miR-152-3p
NM_001191749	1	rno-miR-152-3p
NM_001108722	1	rno-miR-152-3p
NM_024127	1	rno-miR-152-3p
NM_024394	1	rno-miR-152-3p
NM_012929	1	rno-miR-152-3p
NM_031124	1	rno-miR-152-3p
NM_001014272	1	rno-miR-152-3p
NM_001107778	1	rno-miR-152-3p
NM_001100900	1	rno-miR-152-3p
NM_001309638	1	rno-miR-152-3p
NM_001108466	1	rno-miR-152-3p
NM_001109388	1	rno-miR-152-3p
NM_001017496	1	rno-miR-152-3p
NM_001109086	1	rno-miR-152-3p
NM_001106832	1	rno-miR-152-3p
NM_001170469	1	rno-miR-152-3p
NM_001012174	1	rno-miR-152-3p
NM_207605	1	rno-miR-152-3p
NM_022399	1	rno-miR-152-3p
NM_054004	1	rno-miR-152-3p
NM_024138	1	rno-miR-152-3p
NM_053917	1	rno-miR-152-3p
NM_031831	1	rno-miR-152-3p
NM_199389	1	rno-miR-152-3p
NM_173839	1	rno-miR-152-3p
NM_031357	1	rno-miR-152-3p
NM_001007751	1	rno-miR-152-3p

NM_019377	1	rno-miR-152-3p
NM_001105714	1	rno-miR-152-3p
NM_001106000	1	rno-miR-152-3p
NM_001171096	1	rno-miR-152-3p
NM_001191658	1	rno-miR-152-3p
NM_053750	1	rno-miR-152-3p
NM_001107880	1	rno-miR-152-3p
NM_001108997	1	rno-miR-152-3p
NM_001033914	1	rno-miR-152-3p
NM_001107073	1	rno-miR-152-3p
NM_021852	1	rno-miR-152-3p
NM_001191620	1	rno-miR-152-3p
NM_022628	1	rno-miR-152-3p
NM_001014221	1	rno-miR-152-3p
NM_001007674	1	rno-miR-152-3p
NM_133554	1	rno-miR-152-3p
NM_001034151	1	rno-miR-152-3p
NM_017063	1	rno-miR-152-3p
NM_133608	1	rno-miR-152-3p
NM_001107972	1	rno-miR-152-3p
NM_001276429	1	rno-miR-152-3p
NM_001134698	1	rno-miR-152-3p
NM_001191572	1	rno-miR-152-3p
NM_053866	1	rno-miR-152-3p
NM_022685	1	rno-miR-152-3p
NM_053547	1	rno-miR-152-3p
NM_001024981	1	rno-miR-152-3p
NM_001098667	1	rno-miR-152-3p
NM_001106877	1	rno-miR-15b-5p
NM_057137	1	rno-miR-151-5p

NM_199108	1	rno-miR-151-5p
NM_001100833	1	rno-miR-151-5p
NM_001014255	1	rno-miR-151-5p
NM_001313942	1	rno-miR-151-5p
NM_001106597	1	rno-miR-151-5p
NM_001191855	1	rno-miR-151-5p
NM_031701	1	rno-miR-151-5p
NM_013158	1	rno-miR-151-5p
NM_001162408	1	rno-miR-151-5p
NM_001077356	1	rno-miR-151-5p
NM_024132	1	rno-miR-151-5p
NM_001108455	1	rno-miR-151-5p
NM_001013066	1	rno-miR-151-5p
NM_020083	1	rno-miR-151-5p
NM_001107251	1	rno-miR-151-5p
NM_019222	1	rno-miR-151-5p
NM_134363	1	rno-miR-151-5p
NM_001131001	1	rno-miR-151-5p
NM_001013184	1	rno-miR-151-5p
NM_001033884	1	rno-miR-151-5p
NM_024157	1	rno-miR-151-5p
NM_145782	1	rno-miR-151-5p
NM_001006968	1	rno-miR-151-5p
NM_001105992	1	rno-miR-151-5p
NM_001106451	1	rno-miR-151-5p
NM_016987	1	rno-miR-151-5p

Supplementary table S2: GO associated with the target genes for the TOP- 40 highest expressed microRNAS

Analysis Type:	PANTHER Overrepresentation Test (Released 20210224)
Annotation Version and Release Date:	GO Ontology database DOI: 10.5281/zenodo.5080993 Released 2021-07-02
Analyzed List:	Client Text Box Input (Rattus norvegicus)
Reference List:	Rattus norvegicus (all genes in database)
Test Type:	FISHER
Correction:	FDR

GO biological process complete	Rattus norvegicus - REFLIST (21586)	Client Text Box Input (3987)	Client Text Box Input (expected)	Client Text Box Input (over/under)	Client Text Box Input (fold Enrichment)	Client Text Box Input (raw P-value)	Client Text Box Input (FDR)
morphogenesis of an epithelial sheet (GO:0002011)	56	26	10.34	+	2.51	3.18E-04	1.14E-02
regulation of endothelial cell differentiation (GO:0045601)	41	19	7.57	+	2.51	1.87E-03	4.95E-02
endocrine pancreas development (GO:0031018)	41	19	7.57	+	2.51	1.87E-03	4.94E-02
proteoglycan biosynthetic process (GO:0030166)	48	22	8.87	+	2.48	7.86E-04	2.42E-02
glandular epithelial cell differentiation (GO:0002067)	45	20	8.31	+	2.41	1.79E-03	4.82E-02
aorta development (GO:0035904)	63	28	11.64	+	2.41	2.37E-04	8.85E-03
proteoglycan metabolic process (GO:0006029)	71	31	13.11	+	2.36	1.64E-04	6.37E-03
endosome organization (GO:0007032)	53	23	9.79	+	2.35	1.23E-03	3.54E-02
response to increased oxygen levels (GO:0036296)	59	25	10.90	+	2.29	1.24E-03	3.57E-02
positive regulation of macroautophagy (GO:0016239)	64	27	11.82	+	2.28	6.88E-04	2.16E-02
protein K48-linked ubiquitination (GO:0070936)	60	25	11.08	+	2.26	1.34E-03	3.82E-02
regulation of intracellular pH (GO:0051453)	72	30	13.30	+	2.26	4.91E-04	1.63E-02
heart valve development (GO:0003170)	60	25	11.08	+	2.26	1.34E-03	3.81E-02
cell cycle G1/S phase transition (GO:0044843)	77	32	14.22	+	2.25	2.75E-04	1.00E-02
gene silencing by RNA (GO:0031047)	68	28	12.56	+	2.23	6.37E-04	2.02E-02
regulation of pH (GO:0006885)	95	39	17.55	+	2.22	6.50E-05	2.84E-03
negative regulation of cell-substrate adhesion (GO:0010812)	61	25	11.27	+	2.22	1.47E-03	4.16E-02
columnar/cuboidal epithelial cell differentiation (GO:0002065)	83	34	15.33	+	2.22	2.71E-04	9.95E-03
regulation of cellular pH (GO:0030641)	81	33	14.96	+	2.21	2.53E-04	9.34E-03

neuromuscular process controlling balance (GO:0050885)	69	28	12.74	+	2.20	1.02E-03	3.02E-02
regulation of carbohydrate biosynthetic process (GO:0043255)	112	45	20.69	+	2.18	3.31E-05	1.55E-03
G1/S transition of mitotic cell cycle (GO:0000082)	75	30	13.85	+	2.17	6.46E-04	2.04E-02
monovalent inorganic cation homeostasis (GO:0055067)	140	56	25.86	+	2.17	3.85E-06	2.28E-04
positive regulation of smooth muscle cell proliferation (GO:0048661)	116	46	21.43	+	2.15	2.95E-05	1.40E-03
glycoprotein biosynthetic process (GO:0009101)	240	95	44.33	+	2.14	2.11E-09	2.36E-07
pancreas development (GO:0031016)	86	34	15.88	+	2.14	3.48E-04	1.22E-02
cellular monovalent inorganic cation homeostasis (GO:0030004)	107	42	19.76	+	2.13	9.22E-05	3.85E-03
transforming growth factor beta receptor signaling pathway (GO:0007179)	79	31	14.59	+	2.12	8.19E-04	2.50E-02
artery morphogenesis (GO:0048844)	69	27	12.74	+	2.12	1.67E-03	4.56E-02
protein glycosylation (GO:0006486)	190	74	35.09	+	2.11	2.20E-07	1.71E-05
macromolecule glycosylation (GO:0043413)	190	74	35.09	+	2.11	2.20E-07	1.71E-05
cellular polysaccharide metabolic process (GO:0044264)	70	27	12.93	+	2.09	1.84E-03	4.91E-02
peptidyl-threonine modification (GO:0018210)	81	31	14.96	+	2.07	9.75E-04	2.91E-02
inositol lipid-mediated signaling (GO:0048017)	82	31	15.15	+	2.05	1.56E-03	4.34E-02
glycoprotein metabolic process (GO:0009100)	299	113	55.23	+	2.05	6.71E-10	8.24E-08
mitotic cell cycle phase transition (GO:0044772)	151	57	27.89	+	2.04	1.23E-05	6.48E-04
artery development (GO:0060840)	105	39	19.39	+	2.01	4.61E-04	1.55E-02
regulation of striated muscle cell differentiation (GO:0051153)	100	37	18.47	+	2.00	5.48E-04	1.79E-02
carbohydrate biosynthetic process (GO:0016051)	103	38	19.02	+	2.00	6.25E-04	1.99E-02
negative regulation of intrinsic apoptotic signaling pathway (GO:2001243)	106	39	19.58	+	1.99	4.88E-04	1.63E-02
cell cycle phase transition (GO:0044770)	161	59	29.74	+	1.98	2.11E-05	1.05E-03
response to leukemia inhibitory factor (GO:1990823)	126	46	23.27	+	1.98	1.86E-04	7.14E-03
glycosylation (GO:0070085)	206	75	38.05	+	1.97	1.85E-06	1.16E-04
sodium ion transmembrane transport (GO:0035725)	113	41	20.87	+	1.96	4.71E-04	1.58E-02
cellular response to leukemia inhibitory factor (GO:1990830)	125	45	23.09	+	1.95	2.65E-04	9.76E-03
ceramide metabolic process (GO:0006672)	92	33	16.99	+	1.94	1.80E-03	4.82E-02
positive regulation of protein binding (GO:0032092)	98	35	18.10	+	1.93	1.61E-03	4.46E-02
osteoblast differentiation (GO:0001649)	114	40	21.06	+	1.90	7.74E-04	2.39E-02
regulation of smooth muscle cell proliferation (GO:0048660)	180	63	33.25	+	1.89	3.62E-05	1.68E-03

sphingolipid metabolic process (GO:0006665)	133	46	24.57	+	1.87	5.67E-04	1.84E-02
regulation of muscle adaptation (GO:0043502)	108	37	19.95	+	1.85	1.81E-03	4.84E-02
protein polyubiquitination (GO:0000209)	214	73	39.53	+	1.85	1.74E-05	8.87E-04
ossification (GO:0001503)	258	87	47.65	+	1.83	3.07E-06	1.85E-04
negative regulation of protein-containing complex assembly (GO:0031333)	143	48	26.41	+	1.82	6.02E-04	1.92E-02
regulation of muscle cell differentiation (GO:0051147)	146	49	26.97	+	1.82	4.88E-04	1.62E-02
Ras protein signal transduction (GO:0007265)	153	51	28.26	+	1.80	4.46E-04	1.50E-02
regulation of mitochondrion organization (GO:0010821)	138	46	25.49	+	1.80	9.90E-04	2.94E-02
carbohydrate derivative biosynthetic process (GO:1901137)	488	162	90.14	+	1.80	4.45E-10	5.72E-08
cellular response to oxygen levels (GO:0071453)	199	66	36.76	+	1.80	7.93E-05	3.38E-03
neuromuscular process (GO:0050905)	187	62	34.54	+	1.80	1.41E-04	5.61E-03
membrane lipid metabolic process (GO:0006643)	178	59	32.88	+	1.79	2.00E-04	7.60E-03
cytosolic transport (GO:0016482)	133	44	24.57	+	1.79	1.20E-03	3.49E-02
cellular response to hypoxia (GO:0071456)	164	54	30.29	+	1.78	4.67E-04	1.56E-02
cellular response to transforming growth factor beta stimulus (GO:0071560)	158	52	29.18	+	1.78	5.32E-04	1.74E-02
endocrine system development (GO:0035270)	149	49	27.52	+	1.78	7.61E-04	2.36E-02
cellular response to carbohydrate stimulus (GO:0071322)	131	43	24.20	+	1.78	1.57E-03	4.36E-02
regulation of tube size (GO:0035150)	180	59	33.25	+	1.77	2.23E-04	8.35E-03
cellular response to starvation (GO:0009267)	174	57	32.14	+	1.77	3.41E-04	1.21E-02
organic hydroxy compound transport (GO:0015850)	159	52	29.37	+	1.77	5.62E-04	1.83E-02
angiogenesis (GO:0001525)	286	93	52.83	+	1.76	5.91E-06	3.36E-04
blood vessel diameter maintenance (GO:0097746)	179	58	33.06	+	1.75	4.05E-04	1.39E-02
regulation of tube diameter (GO:0035296)	179	58	33.06	+	1.75	4.05E-04	1.38E-02
vacuole organization (GO:0007033)	152	49	28.07	+	1.75	1.17E-03	3.43E-02
positive regulation of binding (GO:0051099)	196	63	36.20	+	1.74	2.69E-04	9.88E-03
vacuolar transport (GO:0007034)	150	48	27.71	+	1.73	1.52E-03	4.28E-02
regulation of endothelial cell migration (GO:0010594)	172	55	31.77	+	1.73	6.30E-04	2.00E-02
cellular response to steroid hormone stimulus (GO:0071383)	191	61	35.28	+	1.73	3.23E-04	1.15E-02
response to transforming growth factor beta (GO:0071559)	166	53	30.66	+	1.73	9.69E-04	2.90E-02
regulation of myeloid cell differentiation (GO:0045637)	213	68	39.34	+	1.73	1.78E-04	6.88E-03

cellular response to nutrient levels (GO:0031669)	245	78	45.25	+	1.72	5.41E-05	2.40E-03
blood vessel development (GO:0001568)	503	160	92.91	+	1.72	7.09E-09	7.25E-07
peptidyl-serine modification (GO:0018209)	217	69	40.08	+	1.72	1.50E-04	5.92E-03
digestive system development (GO:0055123)	173	55	31.95	+	1.72	8.81E-04	2.66E-02
blood vessel morphogenesis (GO:0048514)	400	127	73.88	+	1.72	2.89E-07	2.15E-05
response to endoplasmic reticulum stress (GO:0034976)	218	69	40.27	+	1.71	2.08E-04	7.87E-03
vesicle organization (GO:0016050)	263	83	48.58	+	1.71	4.96E-05	2.24E-03
negative regulation of cytoskeleton organization (GO:0051494)	165	52	30.48	+	1.71	1.29E-03	3.71E-02
cellular response to decreased oxygen levels (GO:0036294)	178	56	32.88	+	1.70	7.68E-04	2.38E-02
negative regulation of apoptotic signaling pathway (GO:2001234)	245	77	45.25	+	1.70	9.94E-05	4.09E-03
peptidyl-serine phosphorylation (GO:0018105)	191	60	35.28	+	1.70	5.95E-04	1.91E-02
regulation of carbohydrate metabolic process (GO:0006109)	204	64	37.68	+	1.70	3.53E-04	1.24E-02
vasculature development (GO:0001944)	536	168	99.00	+	1.70	7.45E-09	7.57E-07
negative regulation of phosphorus metabolic process (GO:0010563)	476	149	87.92	+	1.69	5.15E-08	4.44E-06
negative regulation of phosphate metabolic process (GO:0045936)	476	149	87.92	+	1.69	5.15E-08	4.42E-06
regulation of regulated secretory pathway (GO:1903305)	190	59	35.09	+	1.68	7.92E-04	2.44E-02
placenta development (GO:0001890)	187	58	34.54	+	1.68	9.80E-04	2.92E-02
regulation of cellular carbohydrate metabolic process (GO:0010675)	171	53	31.58	+	1.68	1.55E-03	4.34E-02
negative regulation of translation (GO:0017148)	165	51	30.48	+	1.67	1.81E-03	4.85E-02
glucose homeostasis (GO:0042593)	256	79	47.28	+	1.67	1.36E-04	5.47E-03
response to oxygen levels (GO:0070482)	475	146	87.73	+	1.66	2.18E-07	1.70E-05
regulation of neuron apoptotic process (GO:0043523)	277	85	51.16	+	1.66	7.51E-05	3.24E-03
gastrulation (GO:0007369)	176	54	32.51	+	1.66	1.78E-03	4.80E-02
ubiquitin-dependent protein catabolic process (GO:0006511)	502	154	92.72	+	1.66	1.03E-07	8.58E-06
response to starvation (GO:0042594)	238	73	43.96	+	1.66	2.73E-04	9.98E-03
carbohydrate homeostasis (GO:0033500)	258	79	47.65	+	1.66	1.45E-04	5.79E-03
regulation of intrinsic apoptotic signaling pathway (GO:2001242)	180	55	33.25	+	1.65	1.51E-03	4.25E-02
vascular process in circulatory system (GO:0003018)	239	73	44.14	+	1.65	2.82E-04	1.03E-02
learning (GO:0007612)	190	58	35.09	+	1.65	1.44E-03	4.06E-02
axon development (GO:0061564)	400	122	73.88	+	1.65	3.16E-06	1.89E-04
negative regulation of phosphorylation (GO:0042326)	400	122	73.88	+	1.65	3.16E-06	1.89E-04
negative regulation of protein phosphorylation (GO:0001933)	355	108	65.57	+	1.65	1.32E-05	6.91E-04

cellular response to extracellular stimulus (GO:0031668)	283	86	52.27	+	1.65	8.86E-05	3.76E-03
proteasome-mediated ubiquitin-dependent protein catabolic process (GO:0043161)	310	94	57.26	+	1.64	5.50E-05	2.43E-03
heart morphogenesis (GO:0003007)	261	79	48.21	+	1.64	2.09E-04	7.88E-03
establishment of protein localization to membrane (GO:0090150)	205	62	37.86	+	1.64	1.19E-03	3.46E-02
regulation of catabolic process (GO:0009894)	906	274	167.34	+	1.64	2.19E-12	3.88E-10
response to hypoxia (GO:0001666)	414	125	76.47	+	1.63	3.37E-06	2.01E-04
positive regulation of protein catabolic process (GO:0045732)	226	68	41.74	+	1.63	6.45E-04	2.04E-02
positive regulation of catabolic process (GO:0009896)	486	146	89.77	+	1.63	5.22E-07	3.67E-05
modification-dependent protein catabolic process (GO:0019941)	516	155	95.31	+	1.63	2.60E-07	1.97E-05
axonogenesis (GO:0007409)	353	106	65.20	+	1.63	2.21E-05	1.08E-03
regulation of cellular catabolic process (GO:0031329)	753	226	139.08	+	1.62	4.56E-10	5.83E-08
connective tissue development (GO:0061448)	240	72	44.33	+	1.62	5.13E-04	1.69E-02
second-messenger-mediated signaling (GO:0019932)	211	63	38.97	+	1.62	1.37E-03	3.90E-02
regulation of exocytosis (GO:0017157)	258	77	47.65	+	1.62	3.45E-04	1.22E-02
proteasomal protein catabolic process (GO:0010498)	352	105	65.02	+	1.62	2.88E-05	1.37E-03
lipid biosynthetic process (GO:0008610)	504	150	93.09	+	1.61	5.99E-07	4.16E-05
positive regulation of cellular catabolic process (GO:0031331)	417	124	77.02	+	1.61	6.35E-06	3.57E-04
regulation of muscle system process (GO:0090257)	259	77	47.84	+	1.61	3.59E-04	1.25E-02
response to decreased oxygen levels (GO:0036293)	434	129	80.16	+	1.61	4.14E-06	2.44E-04
regulation of epithelial cell migration (GO:0010632)	239	71	44.14	+	1.61	6.68E-04	2.10E-02
regulation of protein catabolic process (GO:0042176)	395	117	72.96	+	1.60	1.49E-05	7.77E-04
response to growth factor (GO:0070848)	581	172	107.31	+	1.60	1.30E-07	1.05E-05
regulation of mRNA metabolic process (GO:1903311)	291	86	53.75	+	1.60	2.46E-04	9.13E-03
regulation of cell junction assembly (GO:1901888)	220	65	40.63	+	1.60	1.29E-03	3.70E-02
cellular protein catabolic process (GO:0044257)	631	186	116.55	+	1.60	4.60E-08	4.01E-06
regulation of neuron death (GO:1901214)	391	115	72.22	+	1.59	2.33E-05	1.13E-03
modification-dependent macromolecule catabolic process (GO:0043632)	527	155	97.34	+	1.59	7.57E-07	5.14E-05
cellular response to growth factor stimulus (GO:0071363)	541	159	99.92	+	1.59	6.02E-07	4.16E-05
response to mechanical stimulus (GO:0009612)	320	94	59.10	+	1.59	1.23E-04	4.97E-03
regulation of protein binding (GO:0043393)	218	64	40.27	+	1.59	1.63E-03	4.48E-02
wound healing (GO:0042060)	327	96	60.40	+	1.59	1.09E-04	4.45E-03

apoptotic signaling pathway (GO:0097190)	266	78	49.13	+	1.59	5.42E-04	1.77E-02
autophagy (GO:0006914)	222	65	41.00	+	1.59	1.77E-03	4.79E-02
process utilizing autophagic mechanism (GO:0061919)	222	65	41.00	+	1.59	1.77E-03	4.78E-02
proteolysis involved in cellular protein catabolic process (GO:0051603)	602	176	111.19	+	1.58	1.61E-07	1.29E-05
regulation of autophagy (GO:0010506)	250	73	46.18	+	1.58	8.58E-04	2.60E-02
enzyme linked receptor protein signaling pathway (GO:0007167)	572	167	105.65	+	1.58	4.06E-07	2.90E-05
anatomical structure formation involved in morphogenesis (GO:0048646)	942	275	173.99	+	1.58	4.51E-11	6.54E-09
negative regulation of protein modification process (GO:0031400)	552	161	101.96	+	1.58	7.60E-07	5.14E-05
protein catabolic process (GO:0030163)	693	202	128.00	+	1.58	2.68E-08	2.43E-06
apoptotic process (GO:0006915)	714	208	131.88	+	1.58	1.47E-08	1.42E-06
regulation of cellular response to growth factor stimulus (GO:0090287)	292	85	53.93	+	1.58	3.32E-04	1.18E-02
locomotory behavior (GO:0007626)	244	71	45.07	+	1.58	1.00E-03	2.98E-02
protein transport (GO:0015031)	1059	308	195.60	+	1.57	4.23E-12	6.97E-10
regulation of small molecule metabolic process (GO:0062012)	365	106	67.42	+	1.57	6.62E-05	2.88E-03
response to steroid hormone (GO:0048545)	448	130	82.75	+	1.57	9.74E-06	5.27E-04
glycerolipid metabolic process (GO:0046486)	338	98	62.43	+	1.57	1.38E-04	5.54E-03
glycerophospholipid metabolic process (GO:0006650)	266	77	49.13	+	1.57	7.20E-04	2.24E-02
extracellular matrix organization (GO:0030198)	266	77	49.13	+	1.57	7.20E-04	2.23E-02
regulation of developmental growth (GO:0048638)	394	114	72.77	+	1.57	4.22E-05	1.93E-03
neuron projection development (GO:0031175)	730	211	134.83	+	1.56	2.02E-08	1.89E-06
regulation of neuron differentiation (GO:0045664)	232	67	42.85	+	1.56	1.71E-03	4.66E-02
extracellular structure organization (GO:0043062)	267	77	49.32	+	1.56	9.39E-04	2.82E-02
negative regulation of kinase activity (GO:0033673)	243	70	44.88	+	1.56	1.63E-03	4.47E-02
cell morphogenesis involved in neuron differentiation (GO:0048667)	448	129	82.75	+	1.56	1.60E-05	8.25E-04
cellular response to external stimulus (GO:0071496)	389	112	71.85	+	1.56	6.36E-05	2.80E-03
cell death (GO:0008219)	792	228	146.28	+	1.56	6.84E-09	7.05E-07
external encapsulating structure organization (GO:0045229)	268	77	49.50	+	1.56	9.59E-04	2.87E-02
programmed cell death (GO:0012501)	752	216	138.90	+	1.56	1.90E-08	1.78E-06
neuron development (GO:0048666)	902	259	166.60	+	1.55	7.52E-10	9.16E-08
posttranscriptional regulation of gene expression (GO:0010608)	488	140	90.14	+	1.55	7.69E-06	4.23E-04
mitochondrion organization (GO:0007005)	436	125	80.53	+	1.55	2.71E-05	1.29E-03
cell fate commitment (GO:0045165)	262	75	48.39	+	1.55	1.11E-03	3.28E-02

organic acid biosynthetic process (GO:0016053)	245	70	45.25	+	1.55	1.72E-03	4.67E-02
endomembrane system organization (GO:0010256)	449	128	82.93	+	1.54	2.70E-05	1.29E-03
intracellular protein transport (GO:0006886)	674	192	124.49	+	1.54	2.36E-07	1.80E-05
regulation of transmembrane receptor protein serine/threonine kinase signaling pathway (GO:0090092)	253	72	46.73	+	1.54	1.57E-03	4.36E-02
positive regulation of cell migration (GO:0030335)	580	165	107.13	+	1.54	1.71E-06	1.09E-04
nitrogen compound transport (GO:0071705)	1439	409	265.79	+	1.54	1.48E-14	3.63E-12
chemical homeostasis (GO:0048878)	1225	348	226.26	+	1.54	1.70E-12	3.04E-10
intracellular signal transduction (GO:0035556)	1377	391	254.34	+	1.54	6.20E-14	1.43E-11
transmembrane receptor protein tyrosine kinase signaling pathway (GO:0007169)	370	105	68.34	+	1.54	1.57E-04	6.13E-03
cellular cation homeostasis (GO:0030003)	675	191	124.67	+	1.53	3.93E-07	2.83E-05
myeloid cell differentiation (GO:0030099)	258	73	47.65	+	1.53	1.71E-03	4.67E-02
neuron projection morphogenesis (GO:0048812)	488	138	90.14	+	1.53	1.63E-05	8.35E-04
circulatory system development (GO:0072359)	955	270	176.39	+	1.53	1.21E-09	1.43E-07
cellular macromolecule catabolic process (GO:0044265)	803	227	148.32	+	1.53	2.91E-08	2.62E-06
small molecule biosynthetic process (GO:0044283)	407	115	75.17	+	1.53	9.00E-05	3.77E-03
cellular chemical homeostasis (GO:0055082)	829	234	153.12	+	1.53	1.74E-08	1.66E-06
cellular ion homeostasis (GO:0006873)	691	195	127.63	+	1.53	3.21E-07	2.34E-05
regulation of cellular response to stress (GO:0080135)	663	187	122.46	+	1.53	5.23E-07	3.66E-05
establishment of protein localization (GO:0045184)	1160	327	214.26	+	1.53	2.17E-11	3.21E-09
positive regulation of cytosolic calcium ion concentration (GO:0007204)	323	91	59.66	+	1.53	6.05E-04	1.93E-02
positive regulation of apoptotic process (GO:0043065)	600	169	110.82	+	1.52	2.44E-06	1.49E-04
phospholipid metabolic process (GO:0006644)	341	96	62.98	+	1.52	4.02E-04	1.38E-02
regulation of apoptotic signaling pathway (GO:2001233)	405	114	74.80	+	1.52	1.11E-04	4.50E-03
plasma membrane bounded cell projection morphogenesis (GO:0120039)	494	139	91.24	+	1.52	1.82E-05	9.20E-04
response to corticosteroid (GO:0031960)	320	90	59.10	+	1.52	5.80E-04	1.87E-02
tube morphogenesis (GO:0035239)	701	197	129.48	+	1.52	3.07E-07	2.24E-05
developmental growth (GO:0048589)	470	132	86.81	+	1.52	3.15E-05	1.49E-03
cellular response to peptide (GO:1901653)	385	108	71.11	+	1.52	2.04E-04	7.74E-03
cell division (GO:0051301)	364	102	67.23	+	1.52	2.93E-04	1.06E-02
carbohydrate derivative metabolic process (GO:1901135)	871	244	160.88	+	1.52	1.39E-08	1.36E-06

ion homeostasis (GO:0050801)	814	228	150.35	+	1.52	4.51E-08	3.96E-06
positive regulation of locomotion (GO:0040017)	625	175	115.44	+	1.52	1.85E-06	1.16E-04
regulation of angiogenesis (GO:0045765)	293	82	54.12	+	1.52	1.21E-03	3.49E-02
rhythmic process (GO:0048511)	336	94	62.06	+	1.51	5.97E-04	1.91E-02
positive regulation of programmed cell death (GO:0043068)	615	172	113.59	+	1.51	2.50E-06	1.52E-04
cation homeostasis (GO:0055080)	755	211	139.45	+	1.51	1.80E-07	1.44E-05
regulation of vasculature development (GO:1901342)	297	83	54.86	+	1.51	1.29E-03	3.69E-02
regulation of lipid metabolic process (GO:0019216)	365	102	67.42	+	1.51	3.01E-04	1.09E-02
negative regulation of catabolic process (GO:0009895)	315	88	58.18	+	1.51	8.56E-04	2.60E-02
neuron differentiation (GO:0030182)	1124	314	207.61	+	1.51	1.34E-10	1.84E-08
negative regulation of signal transduction (GO:0009968)	1233	344	227.74	+	1.51	1.74E-11	2.64E-09
lipid catabolic process (GO:0016042)	276	77	50.98	+	1.51	1.85E-03	4.92E-02
tissue morphogenesis (GO:0048729)	639	178	118.03	+	1.51	2.32E-06	1.43E-04
cell projection morphogenesis (GO:0048858)	499	139	92.17	+	1.51	3.17E-05	1.49E-03
growth (GO:0040007)	474	132	87.55	+	1.51	4.31E-05	1.97E-03
regulation of binding (GO:0051098)	395	110	72.96	+	1.51	1.94E-04	7.39E-03
positive regulation of cell death (GO:0010942)	690	192	127.45	+	1.51	8.25E-07	5.55E-05
macromolecule catabolic process (GO:0009057)	903	251	166.79	+	1.50	1.47E-08	1.42E-06
lipid localization (GO:0010876)	331	92	61.14	+	1.50	7.04E-04	2.20E-02
learning or memory (GO:0007611)	331	92	61.14	+	1.50	7.04E-04	2.20E-02
positive regulation of secretion by cell (GO:1903532)	367	102	67.79	+	1.50	3.91E-04	1.35E-02
tube development (GO:0035295)	979	272	180.82	+	1.50	3.76E-09	4.00E-07
negative regulation of signaling (GO:0023057)	1361	378	251.38	+	1.50	2.37E-12	4.16E-10
inorganic ion homeostasis (GO:0098771)	767	213	141.67	+	1.50	2.21E-07	1.70E-05
negative regulation of cell communication (GO:0010648)	1359	377	251.01	+	1.50	2.92E-12	4.90E-10
response to glucocorticoid (GO:0051384)	292	81	53.93	+	1.50	1.52E-03	4.28E-02
positive regulation of cell motility (GO:2000147)	606	168	111.93	+	1.50	5.49E-06	3.15E-04
cellular homeostasis (GO:0019725)	914	253	168.82	+	1.50	1.76E-08	1.66E-06
regulation of phosphate metabolic process (GO:0019220)	1483	410	273.91	+	1.50	4.58E-13	9.03E-11
regulation of apoptotic process (GO:0042981)	1502	415	277.42	+	1.50	3.04E-13	6.31E-11
regulation of phosphorus metabolic process (GO:0051174)	1484	410	274.10	+	1.50	4.66E-13	9.06E-11
heart development (GO:0007507)	619	171	114.33	+	1.50	5.37E-06	3.10E-04

regulation of growth (GO:0040008)	682	188	125.97	+	1.49	1.84E-06	1.16E-04
regulation of anatomical structure size (GO:0090066)	595	164	109.90	+	1.49	9.22E-06	5.00E-04
regulation of cytosolic calcium ion concentration (GO:0051480)	374	103	69.08	+	1.49	4.44E-04	1.50E-02
regulation of anatomical structure morphogenesis (GO:0022603)	977	269	180.45	+	1.49	9.28E-09	9.37E-07
regulation of protein serine/threonine kinase activity (GO:0071900)	367	101	67.79	+	1.49	5.03E-04	1.66E-02
cellular response to hormone stimulus (GO:0032870)	600	165	110.82	+	1.49	9.96E-06	5.37E-04
regulation of translation (GO:0006417)	382	105	70.56	+	1.49	4.03E-04	1.38E-02
organic substance transport (GO:0071702)	1784	490	329.51	+	1.49	3.49E-15	9.45E-13
response to wounding (GO:0009611)	477	131	88.10	+	1.49	8.94E-05	3.77E-03
positive regulation of cellular component movement (GO:0051272)	623	171	115.07	+	1.49	7.18E-06	4.01E-04
regulation of neuron projection development (GO:0010975)	554	152	102.33	+	1.49	2.41E-05	1.17E-03
skeletal system development (GO:0001501)	514	141	94.94	+	1.49	5.08E-05	2.28E-03
negative regulation of apoptotic process (GO:0043066)	953	261	176.02	+	1.48	2.52E-08	2.31E-06
regulation of programmed cell death (GO:0043067)	1535	420	283.52	+	1.48	7.96E-13	1.49E-10
positive regulation of ion transport (GO:0043270)	329	90	60.77	+	1.48	1.36E-03	3.87E-02
blood circulation (GO:0008015)	432	118	79.79	+	1.48	2.22E-04	8.32E-03
cellular lipid metabolic process (GO:0044255)	894	244	165.12	+	1.48	9.99E-08	8.35E-06
organelle localization (GO:0051640)	491	134	90.69	+	1.48	8.88E-05	3.76E-03
regulation of protein modification process (GO:0031399)	1587	433	293.12	+	1.48	4.27E-13	8.52E-11
morphogenesis of an epithelium (GO:0002009)	528	144	97.52	+	1.48	5.03E-05	2.26E-03
regulation of protein-containing complex assembly (GO:0043254)	451	123	83.30	+	1.48	1.87E-04	7.14E-03
lipid metabolic process (GO:0006629)	1126	307	207.98	+	1.48	1.79E-09	2.02E-07
cellular response to organic cyclic compound (GO:0071407)	642	175	118.58	+	1.48	7.65E-06	4.24E-04
regulation of protein kinase activity (GO:0045859)	657	179	121.35	+	1.48	6.11E-06	3.45E-04
in utero embryonic development (GO:0001701)	514	140	94.94	+	1.47	6.43E-05	2.82E-03
negative regulation of programmed cell death (GO:0043069)	974	265	179.90	+	1.47	3.42E-08	3.05E-06
regulation of protein phosphorylation (GO:0001932)	1151	313	212.59	+	1.47	1.63E-09	1.89E-07
cellular response to endogenous stimulus (GO:0071495)	1255	341	231.80	+	1.47	3.12E-10	4.09E-08
regulation of phosphorylation (GO:0042325)	1305	354	241.04	+	1.47	1.35E-10	1.83E-08
cellular calcium ion homeostasis (GO:0006874)	483	131	89.21	+	1.47	1.53E-04	6.02E-03
muscle structure development (GO:0061061)	509	138	94.01	+	1.47	9.26E-05	3.86E-03
cellular divalent inorganic cation homeostasis (GO:0072503)	509	138	94.01	+	1.47	9.26E-05	3.85E-03

cellular metal ion homeostasis (GO:0006875)	609	165	112.48	+	1.47	1.80E-05	9.14E-04
head development (GO:0060322)	990	268	182.86	+	1.47	3.50E-08	3.11E-06
behavior (GO:0007610)	713	193	131.69	+	1.47	3.65E-06	2.16E-04
muscle tissue development (GO:0060537)	351	95	64.83	+	1.47	1.22E-03	3.53E-02
negative regulation of cell death (GO:0060548)	1094	296	202.07	+	1.46	6.80E-09	7.04E-07
telencephalon development (GO:0021537)	333	90	61.51	+	1.46	1.82E-03	4.85E-02
regulation of cell growth (GO:0001558)	433	117	79.98	+	1.46	3.51E-04	1.23E-02
regulation of cell death (GO:0010941)	1703	460	314.55	+	1.46	2.80E-13	5.88E-11
cell part morphogenesis (GO:0032990)	526	142	97.15	+	1.46	9.47E-05	3.92E-03
embryonic morphogenesis (GO:0048598)	653	176	120.61	+	1.46	1.39E-05	7.27E-04
protein localization (GO:0008104)	1863	502	344.10	+	1.46	2.18E-14	5.27E-12
modulation of chemical synaptic transmission (GO:0050804)	542	146	100.11	+	1.46	7.67E-05	3.29E-03
negative regulation of intracellular signal transduction (GO:1902532)	531	143	98.08	+	1.46	8.27E-05	3.52E-03
response to hormone (GO:0009725)	1077	290	198.93	+	1.46	1.60E-08	1.53E-06
cellular response to organonitrogen compound (GO:0071417)	702	189	129.66	+	1.46	6.03E-06	3.41E-04
cellular response to lipid (GO:0071396)	624	168	115.25	+	1.46	2.21E-05	1.09E-03
response to peptide (GO:1901652)	624	168	115.25	+	1.46	2.21E-05	1.08E-03
negative regulation of transport (GO:0051051)	520	140	96.05	+	1.46	1.08E-04	4.43E-03
regulation of trans-synaptic signaling (GO:0099177)	543	146	100.29	+	1.46	7.81E-05	3.34E-03
reproductive system development (GO:0061458)	558	150	103.06	+	1.46	6.24E-05	2.75E-03
epithelium development (GO:0060429)	1098	295	202.80	+	1.45	1.38E-08	1.35E-06
cellular protein modification process (GO:0006464)	2479	666	457.88	+	1.45	5.67E-19	2.45E-16
protein modification process (GO:0036211)	2479	666	457.88	+	1.45	5.67E-19	2.38E-16
cell morphogenesis involved in differentiation (GO:0000904)	577	155	106.57	+	1.45	5.24E-05	2.33E-03
circulatory system process (GO:0003013)	462	124	85.33	+	1.45	2.78E-04	1.01E-02
regulation of secretion by cell (GO:1903530)	697	187	128.74	+	1.45	8.63E-06	4.72E-04
regulation of cellular amide metabolic process (GO:0034248)	440	118	81.27	+	1.45	4.79E-04	1.60E-02
cellular response to nitrogen compound (GO:1901699)	772	207	142.59	+	1.45	2.78E-06	1.69E-04
protein localization to membrane (GO:0072657)	463	124	85.52	+	1.45	3.44E-04	1.22E-02
epithelial cell differentiation (GO:0030855)	564	151	104.17	+	1.45	6.80E-05	2.95E-03
reproductive structure development (GO:0048608)	553	148	102.14	+	1.45	8.92E-05	3.76E-03
homeostatic process (GO:0042592)	1689	452	311.96	+	1.45	1.39E-12	2.52E-10

macromolecule localization (GO:0033036)	2240	598	413.73	+	1.45	1.87E-16	5.96E-14
protein ubiquitination (GO:0016567)	592	158	109.34	+	1.44	5.22E-05	2.33E-03
urogenital system development (GO:0001655)	405	108	74.80	+	1.44	8.78E-04	2.65E-02
negative regulation of response to stimulus (GO:0048585)	1621	432	299.40	+	1.44	7.94E-12	1.24E-09
positive regulation of phosphorus metabolic process (GO:0010562)	942	251	173.99	+	1.44	3.03E-07	2.24E-05
positive regulation of phosphate metabolic process (GO:0045937)	942	251	173.99	+	1.44	3.03E-07	2.23E-05
generation of neurons (GO:0048699)	1400	373	258.58	+	1.44	3.04E-10	4.01E-08
regulation of cellular localization (GO:0060341)	916	244	169.19	+	1.44	5.00E-07	3.53E-05
export from cell (GO:0140352)	432	115	79.79	+	1.44	6.61E-04	2.08E-02
cellular component morphogenesis (GO:0032989)	620	165	114.52	+	1.44	4.01E-05	1.85E-03
mitotic cell cycle process (GO:1903047)	481	128	88.84	+	1.44	3.54E-04	1.24E-02
metal ion homeostasis (GO:0055065)	684	182	126.34	+	1.44	1.70E-05	8.69E-04
positive regulation of cell population proliferation (GO:0008284)	1019	271	188.21	+	1.44	1.21E-07	9.83E-06
localization within membrane (GO:0051668)	527	140	97.34	+	1.44	1.82E-04	7.03E-03
phosphorus metabolic process (GO:0006793)	1766	469	326.19	+	1.44	1.25E-12	2.30E-10
calcium ion homeostasis (GO:0055074)	501	133	92.54	+	1.44	2.48E-04	9.19E-03
brain development (GO:0007420)	938	249	173.25	+	1.44	4.39E-07	3.13E-05
response to lipid (GO:0033993)	1202	319	222.01	+	1.44	9.58E-09	9.61E-07
divalent inorganic cation homeostasis (GO:0072507)	539	143	99.55	+	1.44	1.73E-04	6.70E-03
regulation of secretion (GO:0051046)	781	207	144.25	+	1.43	4.83E-06	2.81E-04
regulation of cell migration (GO:0030334)	970	257	179.16	+	1.43	3.47E-07	2.52E-05
secretion by cell (GO:0032940)	385	102	71.11	+	1.43	1.57E-03	4.36E-02
tissue development (GO:0009888)	1744	462	322.12	+	1.43	2.77E-12	4.76E-10
embryonic organ development (GO:0048568)	521	138	96.23	+	1.43	2.10E-04	7.91E-03
positive regulation of signal transduction (GO:0009967)	1553	411	286.84	+	1.43	6.78E-11	9.66E-09
regulation of system process (GO:0044057)	643	170	118.76	+	1.43	4.34E-05	1.97E-03
organophosphate biosynthetic process (GO:0090407)	435	115	80.35	+	1.43	8.47E-04	2.58E-02
organonitrogen compound catabolic process (GO:1901565)	999	264	184.52	+	1.43	2.68E-07	2.01E-05
protein phosphorylation (GO:0006468)	685	181	126.52	+	1.43	2.61E-05	1.25E-03
regulation of transmembrane transport (GO:0034762)	602	159	111.19	+	1.43	8.96E-05	3.76E-03
positive regulation of intracellular signal transduction (GO:1902533)	1015	268	187.47	+	1.43	2.64E-07	1.99E-05
intracellular transport (GO:0046907)	1216	321	224.60	+	1.43	1.40E-08	1.36E-06

phosphate-containing compound metabolic process (GO:0006796)	1743	460	321.94	+	1.43	5.18E-12	8.43E-10
regulation of transferase activity (GO:0051338)	887	234	163.83	+	1.43	1.50E-06	9.67E-05
regulation of kinase activity (GO:0043549)	781	206	144.25	+	1.43	7.23E-06	4.02E-04
negative regulation of cellular protein metabolic process (GO:0032269)	1039	274	191.91	+	1.43	1.90E-07	1.51E-05
cellular protein localization (GO:0034613)	1411	372	260.62	+	1.43	8.21E-10	9.92E-08
regulation of cytoskeleton organization (GO:0051493)	539	142	99.55	+	1.43	2.14E-04	8.02E-03
cellular macromolecule localization (GO:0070727)	1421	374	262.46	+	1.42	9.18E-10	1.10E-07
response to nutrient levels (GO:0031667)	707	186	130.59	+	1.42	2.28E-05	1.11E-03
response to organic cyclic compound (GO:0014070)	1319	347	243.62	+	1.42	4.06E-09	4.29E-07
establishment of localization in cell (GO:0051649)	1589	418	293.49	+	1.42	8.47E-11	1.19E-08
positive regulation of protein kinase activity (GO:0045860)	403	106	74.44	+	1.42	1.60E-03	4.43E-02
response to organonitrogen compound (GO:0010243)	1308	344	241.59	+	1.42	5.33E-09	5.56E-07
regulation of cellular component size (GO:0032535)	426	112	78.68	+	1.42	1.15E-03	3.36E-02
response to alcohol (GO:0097305)	411	108	75.91	+	1.42	1.44E-03	4.07E-02
metal ion transport (GO:0030001)	609	160	112.48	+	1.42	9.76E-05	4.03E-03
secretion (GO:0046903)	533	140	98.45	+	1.42	2.97E-04	1.07E-02
response to extracellular stimulus (GO:0009991)	739	194	136.50	+	1.42	1.81E-05	9.17E-04
macromolecule modification (GO:0043412)	2671	701	493.34	+	1.42	5.86E-18	2.03E-15
carbohydrate metabolic process (GO:0005975)	442	116	81.64	+	1.42	9.32E-04	2.80E-02
protein modification by small protein conjugation or removal (GO:0070647)	766	201	141.48	+	1.42	1.13E-05	5.98E-04
peptidyl-amino acid modification (GO:0018193)	847	222	156.44	+	1.42	4.70E-06	2.74E-04
cellular catabolic process (GO:0044248)	1577	413	291.28	+	1.42	1.70E-10	2.28E-08
regulation of cell projection organization (GO:0031344)	760	199	140.37	+	1.42	1.55E-05	8.06E-04
regulation of locomotion (GO:0040012)	1074	281	198.37	+	1.42	2.35E-07	1.80E-05
response to endogenous stimulus (GO:0009719)	1682	440	310.67	+	1.42	4.73E-11	6.79E-09
positive regulation of protein metabolic process (GO:0051247)	1495	391	276.13	+	1.42	6.39E-10	7.90E-08
positive regulation of cell communication (GO:0010647)	1767	462	326.37	+	1.42	1.57E-11	2.41E-09
regulation of signal transduction (GO:0009966)	2858	747	527.88	+	1.42	7.49E-19	3.06E-16
regulation of protein localization (GO:0032880)	953	249	176.02	+	1.41	1.45E-06	9.38E-05
cation transport (GO:0006812)	827	216	152.75	+	1.41	6.88E-06	3.85E-04
response to metal ion (GO:0010038)	517	135	95.49	+	1.41	4.50E-04	1.52E-02

regulation of intracellular signal transduction (GO:1902531)	1633	426	301.62	+	1.41	1.40E-10	1.89E-08
phosphorylation (GO:0016310)	924	241	170.67	+	1.41	2.31E-06	1.43E-04
positive regulation of cellular metabolic process (GO:0031325)	3244	846	599.18	+	1.41	2.52E-21	1.22E-18
negative regulation of protein metabolic process (GO:0051248)	1101	287	203.36	+	1.41	2.15E-07	1.69E-05
positive regulation of secretion (GO:0051047)	422	110	77.94	+	1.41	1.64E-03	4.50E-02
regulation of cellular component biogenesis (GO:0044087)	990	258	182.86	+	1.41	9.95E-07	6.56E-05
protein modification by small protein conjugation (GO:0032446)	637	166	117.66	+	1.41	1.11E-04	4.49E-03
regulation of cell communication (GO:0010646)	3293	858	608.23	+	1.41	1.41E-21	7.24E-19
regulation of cellular protein localization (GO:1903827)	595	155	109.90	+	1.41	1.85E-04	7.10E-03
positive regulation of signaling (GO:0023056)	1774	462	327.66	+	1.41	2.56E-11	3.75E-09
regulation of cell motility (GO:2000145)	1026	267	189.51	+	1.41	6.76E-07	4.63E-05
regulation of signaling (GO:0023051)	3305	860	610.44	+	1.41	1.60E-21	7.98E-19
positive regulation of cell projection organization (GO:0031346)	442	115	81.64	+	1.41	1.39E-03	3.93E-02
response to abiotic stimulus (GO:0009628)	1407	366	259.88	+	1.41	4.87E-09	5.11E-07
positive regulation of protein phosphorylation (GO:0001934)	773	201	142.78	+	1.41	1.83E-05	9.19E-04
regulation of MAPK cascade (GO:0043408)	708	184	130.77	+	1.41	5.11E-05	2.28E-03
regulation of biological quality (GO:0065008)	3920	1018	724.04	+	1.41	7.51E-26	6.65E-23
positive regulation of phosphorylation (GO:0042327)	855	222	157.92	+	1.41	7.68E-06	4.24E-04
regulation of cellular protein metabolic process (GO:0032268)	2425	629	447.90	+	1.40	3.80E-15	9.95E-13
positive regulation of nitrogen compound metabolic process (GO:0051173)	3032	786	560.02	+	1.40	3.65E-19	1.62E-16
animal organ morphogenesis (GO:0009887)	1061	275	195.97	+	1.40	6.67E-07	4.58E-05
forebrain development (GO:0030900)	490	127	90.50	+	1.40	8.91E-04	2.68E-02
regulation of ion transmembrane transport (GO:0034765)	498	129	91.98	+	1.40	8.01E-04	2.46E-02
inorganic cation transmembrane transport (GO:0098662)	560	145	103.43	+	1.40	3.38E-04	1.20E-02
positive regulation of protein modification process (GO:0031401)	1020	264	188.40	+	1.40	1.14E-06	7.43E-05
cellular response to cytokine stimulus (GO:0071345)	796	206	147.02	+	1.40	1.92E-05	9.62E-04
regulation of plasma membrane bounded cell projection organization (GO:0120035)	742	192	137.05	+	1.40	4.14E-05	1.90E-03
positive regulation of transferase activity (GO:0051347)	565	146	104.36	+	1.40	3.56E-04	1.24E-02
cellular response to organic substance (GO:0071310)	2233	577	412.44	+	1.40	1.31E-13	2.87E-11
anatomical structure morphogenesis (GO:0009653)	2272	587	419.65	+	1.40	7.19E-14	1.62E-11

response to nitrogen compound (GO:1901698)	1398	361	258.21	+	1.40	1.19E-08	1.18E-06
neurogenesis (GO:0022008)	1553	401	286.84	+	1.40	1.47E-09	1.71E-07
cellular localization (GO:0051641)	2278	588	420.75	+	1.40	9.24E-14	2.05E-11
positive regulation of cellular protein metabolic process (GO:0032270)	1411	364	260.62	+	1.40	1.12E-08	1.12E-06
regulation of membrane potential (GO:0042391)	477	123	88.10	+	1.40	1.15E-03	3.36E-02
chordate embryonic development (GO:0043009)	807	208	149.06	+	1.40	2.17E-05	1.07E-03
positive regulation of cellular biosynthetic process (GO:0031328)	1983	511	366.27	+	1.40	6.34E-12	1.01E-09
regulation of cellular component organization (GO:0051128)	2501	644	461.94	+	1.39	5.56E-15	1.41E-12
regulation of protein metabolic process (GO:0051246)	2595	668	479.30	+	1.39	1.51E-15	4.47E-13
positive regulation of nucleobase-containing compound metabolic process (GO:0045935)	1921	494	354.81	+	1.39	2.01E-11	3.03E-09
positive regulation of biosynthetic process (GO:0009891)	2030	522	374.95	+	1.39	5.57E-12	8.98E-10
positive regulation of RNA metabolic process (GO:0051254)	1751	450	323.42	+	1.39	2.21E-10	2.94E-08
positive regulation of transcription by RNA polymerase II (GO:0045944)	1257	323	232.17	+	1.39	1.10E-07	9.05E-06
central nervous system development (GO:0007417)	1191	306	219.98	+	1.39	2.58E-07	1.96E-05
embryo development (GO:0009790)	1220	313	225.34	+	1.39	1.97E-07	1.56E-05
regulation of cellular component movement (GO:0051270)	1107	284	204.47	+	1.39	8.90E-07	5.92E-05
negative regulation of cellular component organization (GO:0051129)	768	197	141.85	+	1.39	4.54E-05	2.05E-03
positive regulation of cellular component biogenesis (GO:0044089)	539	138	99.55	+	1.39	7.08E-04	2.21E-02
cell morphogenesis (GO:0000902)	750	192	138.53	+	1.39	6.61E-05	2.88E-03
positive regulation of macromolecule biosynthetic process (GO:0010557)	1868	478	345.03	+	1.39	8.41E-11	1.19E-08
response to drug (GO:0042493)	641	164	118.39	+	1.39	2.51E-04	9.28E-03
positive regulation of nucleic acid-templated transcription (GO:1903508)	1619	414	299.03	+	1.38	2.34E-09	2.57E-07
positive regulation of transcription, DNA-templated (GO:0045893)	1619	414	299.03	+	1.38	2.34E-09	2.56E-07
inorganic ion transmembrane transport (GO:0098660)	626	160	115.62	+	1.38	3.13E-04	1.13E-02
positive regulation of RNA biosynthetic process (GO:1902680)	1621	414	299.40	+	1.38	2.41E-09	2.62E-07
positive regulation of MAPK cascade (GO:0043410)	517	132	95.49	+	1.38	1.19E-03	3.46E-02
embryo development ending in birth or egg hatching (GO:0009792)	823	210	152.01	+	1.38	3.71E-05	1.72E-03
positive regulation of metabolic process (GO:0009893)	3743	955	691.34	+	1.38	6.67E-22	3.67E-19
negative regulation of gene expression (GO:0010629)	925	236	170.85	+	1.38	1.09E-05	5.80E-04
plasma membrane bounded cell projection organization (GO:0120036)	1138	290	210.19	+	1.38	1.01E-06	6.61E-05
cation transmembrane transport (GO:0098655)	628	160	115.99	+	1.38	3.23E-04	1.15E-02

regulation of nervous system development (GO:0051960)	538	137	99.37	+	1.38	1.01E-03	2.99E-02
positive regulation of cell differentiation (GO:0045597)	943	240	174.17	+	1.38	1.08E-05	5.76E-04
cell population proliferation (GO:0008283)	558	142	103.06	+	1.38	8.50E-04	2.59E-02
cell development (GO:0048468)	1808	460	333.94	+	1.38	4.68E-10	5.92E-08
positive regulation of developmental process (GO:0051094)	1443	367	266.53	+	1.38	3.34E-08	3.00E-06
regulation of ion transport (GO:0043269)	751	191	138.71	+	1.38	9.69E-05	4.01E-03
regulation of localization (GO:0032879)	2950	750	544.87	+	1.38	1.90E-16	5.96E-14
regulation of response to stress (GO:0080134)	1271	323	234.76	+	1.38	2.69E-07	2.01E-05
cell projection organization (GO:0030030)	1177	299	217.40	+	1.38	8.41E-07	5.64E-05
mitotic cell cycle (GO:0000278)	559	142	103.25	+	1.38	8.59E-04	2.60E-02
regulation of cell population proliferation (GO:0042127)	1728	438	319.17	+	1.37	1.77E-09	2.00E-07
membrane organization (GO:0061024)	760	192	140.37	+	1.37	1.27E-04	5.11E-03
cell migration (GO:0016477)	883	223	163.09	+	1.37	3.20E-05	1.50E-03
regulation of organelle organization (GO:0033043)	1212	306	223.86	+	1.37	9.71E-07	6.43E-05
positive regulation of macromolecule metabolic process (GO:0010604)	3439	868	635.19	+	1.37	1.42E-18	5.52E-16
organic substance catabolic process (GO:1901575)	1550	391	286.29	+	1.37	2.46E-08	2.27E-06
negative regulation of transcription by RNA polymerase II (GO:0000122)	924	233	170.67	+	1.37	2.27E-05	1.11E-03
catabolic process (GO:0009056)	1838	463	339.48	+	1.36	1.09E-09	1.30E-07
regulation of multicellular organismal development (GO:2000026)	1449	365	267.63	+	1.36	8.87E-08	7.45E-06
nervous system development (GO:0007399)	2351	592	434.24	+	1.36	2.90E-12	4.93E-10
ion transport (GO:0006811)	1165	293	215.18	+	1.36	2.25E-06	1.39E-04
regulation of developmental process (GO:0050793)	2538	638	468.78	+	1.36	4.07E-13	8.22E-11
negative regulation of developmental process (GO:0051093)	973	244	179.72	+	1.36	2.06E-05	1.03E-03
cellular response to chemical stimulus (GO:0070887)	2827	708	522.16	+	1.36	2.78E-14	6.63E-12
protein localization to organelle (GO:0033365)	679	170	125.41	+	1.36	4.36E-04	1.48E-02
positive regulation of catalytic activity (GO:0043085)	1123	281	207.42	+	1.35	5.38E-06	3.10E-04
regulation of protein transport (GO:0051223)	580	145	107.13	+	1.35	1.26E-03	3.63E-02
cell-cell signaling (GO:0007267)	809	202	149.42	+	1.35	1.39E-04	5.56E-03
positive regulation of cellular component organization (GO:0051130)	1238	309	228.66	+	1.35	2.08E-06	1.30E-04
proteolysis (GO:0006508)	1214	303	224.23	+	1.35	2.43E-06	1.49E-04
transport (GO:0006810)	3617	902	668.07	+	1.35	3.46E-18	1.26E-15
transmembrane transport (GO:0055085)	1175	293	217.03	+	1.35	4.16E-06	2.44E-04

negative regulation of cell population proliferation (GO:0008285)	718	179	132.62	+	1.35	3.63E-04	1.26E-02
regulation of establishment of protein localization (GO:0070201)	606	151	111.93	+	1.35	1.12E-03	3.28E-02
organophosphate metabolic process (GO:0019637)	799	199	147.58	+	1.35	1.80E-04	6.96E-03
cellular response to oxygen-containing compound (GO:1901701)	1265	315	233.65	+	1.35	1.85E-06	1.16E-04
negative regulation of cell differentiation (GO:0045596)	699	174	129.11	+	1.35	5.17E-04	1.70E-02
cellular response to stress (GO:0033554)	1555	387	287.21	+	1.35	1.08E-07	8.89E-06
ion transmembrane transport (GO:0034220)	824	205	152.20	+	1.35	1.57E-04	6.14E-03
gland development (GO:0048732)	612	152	113.04	+	1.34	1.18E-03	3.44E-02
response to inorganic substance (GO:0010035)	757	188	139.82	+	1.34	3.01E-04	1.09E-02
regulation of cell differentiation (GO:0045595)	1615	401	298.30	+	1.34	7.33E-08	6.19E-06
regulation of transport (GO:0051049)	1950	484	360.17	+	1.34	2.77E-09	2.99E-07
positive regulation of transport (GO:0051050)	1085	269	200.40	+	1.34	1.57E-05	8.10E-04
regulation of cell development (GO:0060284)	594	147	109.71	+	1.34	1.69E-03	4.62E-02
positive regulation of molecular function (GO:0044093)	1496	370	276.32	+	1.34	3.78E-07	2.73E-05
response to cytokine (GO:0034097)	934	231	172.51	+	1.34	7.10E-05	3.07E-03
establishment of localization (GO:0051234)	3784	935	698.92	+	1.34	5.73E-18	2.03E-15
regulation of multicellular organismal process (GO:0051239)	2812	693	519.38	+	1.33	8.39E-13	1.56E-10
negative regulation of cellular process (GO:0048523)	4880	1202	901.35	+	1.33	9.78E-24	6.50E-21
positive regulation of cellular process (GO:0048522)	5779	1423	1067.40	+	1.33	1.71E-29	1.95E-26
negative regulation of multicellular organismal process (GO:0051241)	1105	272	204.10	+	1.33	2.18E-05	1.08E-03
regulation of primary metabolic process (GO:0080090)	5576	1372	1029.90	+	1.33	5.16E-28	5.15E-25
immune system development (GO:0002520)	760	187	140.37	+	1.33	5.11E-04	1.69E-02
regulation of cell adhesion (GO:0030155)	760	187	140.37	+	1.33	5.11E-04	1.68E-02
locomotion (GO:0040011)	1230	302	227.18	+	1.33	9.04E-06	4.92E-04
cell differentiation (GO:0030154)	3458	849	638.70	+	1.33	1.70E-15	4.93E-13
negative regulation of catalytic activity (GO:0043086)	754	185	139.27	+	1.33	5.78E-04	1.87E-02
negative regulation of biological process (GO:0048519)	5307	1301	980.22	+	1.33	1.63E-25	1.30E-22
cell cycle process (GO:0022402)	767	188	141.67	+	1.33	5.41E-04	1.77E-02
positive regulation of biological process (GO:0048518)	6257	1533	1155.69	+	1.33	1.06E-31	1.54E-28
regulation of cellular metabolic process (GO:0031323)	5753	1409	1062.60	+	1.33	3.58E-28	3.81E-25
regulation of response to stimulus (GO:0048583)	3835	939	708.34	+	1.33	4.47E-17	1.48E-14
multicellular organism development (GO:0007275)	4848	1187	895.44	+	1.33	1.60E-22	9.44E-20

system development (GO:0048731)	4523	1107	835.41	+	1.33	1.19E-20	5.58E-18
negative regulation of metabolic process (GO:0009892)	3005	734	555.03	+	1.32	6.76E-13	1.28E-10
vesicle-mediated transport (GO:0016192)	1253	306	231.43	+	1.32	1.07E-05	5.74E-04
regulation of metabolic process (GO:0019222)	6433	1571	1188.19	+	1.32	3.31E-32	5.28E-29
hemopoiesis (GO:0030097)	660	161	121.90	+	1.32	1.72E-03	4.68E-02
cellular developmental process (GO:0048869)	3506	855	647.57	+	1.32	4.93E-15	1.27E-12
cell cycle (GO:0007049)	849	207	156.81	+	1.32	3.74E-04	1.29E-02
negative regulation of molecular function (GO:0044092)	1124	274	207.61	+	1.32	3.51E-05	1.64E-03
hematopoietic or lymphoid organ development (GO:0048534)	722	176	133.36	+	1.32	1.03E-03	3.04E-02
response to organic substance (GO:0010033)	3282	800	606.20	+	1.32	6.51E-14	1.48E-11
animal organ development (GO:0048513)	3576	871	660.50	+	1.32	3.21E-15	8.82E-13
cellular protein metabolic process (GO:0044267)	3232	787	596.96	+	1.32	1.34E-13	2.89E-11
negative regulation of cellular metabolic process (GO:0031324)	2581	628	476.72	+	1.32	1.02E-10	1.42E-08
localization (GO:0051179)	5024	1222	927.95	+	1.32	1.85E-22	1.05E-19
negative regulation of macromolecule metabolic process (GO:0010605)	2788	678	514.95	+	1.32	1.39E-11	2.16E-09
negative regulation of RNA metabolic process (GO:0051253)	1399	340	258.40	+	1.32	4.36E-06	2.55E-04
regulation of catalytic activity (GO:0050790)	2255	547	416.51	+	1.31	3.32E-09	3.56E-07
negative regulation of biosynthetic process (GO:0009890)	1634	396	301.80	+	1.31	8.74E-07	5.84E-05
regulation of nitrogen compound metabolic process (GO:0051171)	5398	1306	997.03	+	1.31	1.30E-23	8.29E-21
anatomical structure development (GO:0048856)	5251	1269	969.88	+	1.31	1.26E-22	7.71E-20
regulation of macromolecule metabolic process (GO:0060255)	5963	1441	1101.38	+	1.31	9.81E-27	9.20E-24
negative regulation of nitrogen compound metabolic process (GO:0051172)	2423	585	447.54	+	1.31	1.41E-09	1.65E-07
negative regulation of cellular biosynthetic process (GO:0031327)	1591	384	293.86	+	1.31	1.71E-06	1.10E-04
localization of cell (GO:0051674)	1032	249	190.61	+	1.31	1.46E-04	5.80E-03
cell motility (GO:0048870)	1032	249	190.61	+	1.31	1.46E-04	5.79E-03
negative regulation of macromolecule biosynthetic process (GO:0010558)	1530	369	282.60	+	1.31	2.96E-06	1.79E-04
regulation of hydrolase activity (GO:0051336)	1083	261	200.03	+	1.30	1.10E-04	4.47E-03
response to oxygen-containing compound (GO:1901700)	2038	491	376.42	+	1.30	5.44E-08	4.62E-06
regulation of molecular function (GO:0065009)	2935	707	542.10	+	1.30	2.17E-11	3.23E-09
protein metabolic process (GO:0019538)	3867	931	714.25	+	1.30	2.91E-15	8.15E-13
negative regulation of nucleic acid-templated transcription (GO:1903507)	1292	310	238.64	+	1.30	3.08E-05	1.46E-03

developmental process (GO:0032502)	5730	1373	1058.35	+	1.30	9.68E-24	6.71E-21
negative regulation of RNA biosynthetic process (GO:1902679)	1294	310	239.01	+	1.30	3.63E-05	1.68E-03
regulation of biosynthetic process (GO:0009889)	3927	940	725.33	+	1.30	7.00E-15	1.74E-12
organonitrogen compound metabolic process (GO:1901564)	4800	1148	886.57	+	1.29	1.07E-18	4.26E-16
negative regulation of cellular macromolecule biosynthetic process (GO:2000113)	1514	362	279.64	+	1.29	7.85E-06	4.30E-04
negative regulation of transcription, DNA-templated (GO:0045892)	1289	308	238.08	+	1.29	4.11E-05	1.89E-03
positive regulation of response to stimulus (GO:0048584)	2196	524	405.61	+	1.29	4.83E-08	4.19E-06
movement of cell or subcellular component (GO:0006928)	1452	346	268.19	+	1.29	1.56E-05	8.08E-04
small molecule metabolic process (GO:0044281)	1515	361	279.83	+	1.29	1.07E-05	5.74E-04
biosynthetic process (GO:0009058)	2305	549	425.74	+	1.29	2.65E-08	2.42E-06
regulation of cellular biosynthetic process (GO:0031326)	3847	916	710.55	+	1.29	5.65E-14	1.33E-11
organic substance biosynthetic process (GO:1901576)	2244	534	414.47	+	1.29	5.43E-08	4.63E-06
regulation of transcription by RNA polymerase II (GO:0006357)	2345	558	433.13	+	1.29	2.43E-08	2.26E-06
regulation of nucleobase-containing compound metabolic process (GO:0019219)	3702	880	683.77	+	1.29	3.58E-13	7.32E-11
cellular macromolecule metabolic process (GO:0044260)	4327	1028	799.21	+	1.29	1.07E-15	3.28E-13
regulation of RNA metabolic process (GO:0051252)	3452	820	637.59	+	1.29	3.86E-12	6.41E-10
cell surface receptor signaling pathway (GO:0007166)	1826	433	337.27	+	1.28	1.80E-06	1.14E-04
regulation of cell cycle (GO:0051726)	983	233	181.56	+	1.28	6.57E-04	2.07E-02
negative regulation of nucleobase-containing compound metabolic process (GO:0045934)	1512	358	279.27	+	1.28	1.90E-05	9.52E-04
cellular biosynthetic process (GO:0044249)	2183	516	403.21	+	1.28	1.76E-07	1.41E-05
regulation of macromolecule biosynthetic process (GO:0010556)	3671	866	678.04	+	1.28	2.49E-12	4.32E-10
regulation of gene expression (GO:0010468)	4545	1072	839.48	+	1.28	1.18E-15	3.55E-13
developmental process involved in reproduction (GO:0003006)	1106	260	204.28	+	1.27	4.17E-04	1.42E-02
regulation of cellular macromolecule biosynthetic process (GO:2000112)	3633	854	671.03	+	1.27	7.27E-12	1.15E-09
positive regulation of multicellular organismal process (GO:0051240)	1614	378	298.11	+	1.27	2.46E-05	1.19E-03
primary metabolic process (GO:0044238)	6775	1584	1251.36	+	1.27	2.45E-24	1.78E-21
organelle organization (GO:0006996)	3300	771	609.52	+	1.26	3.50E-10	4.54E-08
cellular metabolic process (GO:0044237)	6897	1611	1273.90	+	1.26	8.68E-25	6.60E-22
cellular component organization (GO:0016043)	5238	1222	967.47	+	1.26	5.20E-17	1.69E-14

regulation of nucleic acid-templated transcription (GO:1903506)	3160	736	583.66	+	1.26	1.66E-09	1.91E-07
regulation of RNA biosynthetic process (GO:2001141)	3166	737	584.77	+	1.26	1.71E-09	1.95E-07
response to stress (GO:0006950)	3339	777	616.72	+	1.26	4.92E-10	6.19E-08
regulation of transcription, DNA-templated (GO:0006355)	3158	734	583.29	+	1.26	2.22E-09	2.46E-07
organonitrogen compound biosynthetic process (GO:1901566)	1280	296	236.42	+	1.25	4.38E-04	1.49E-02
positive regulation of gene expression (GO:0010628)	1213	279	224.04	+	1.25	8.14E-04	2.49E-02
nitrogen compound metabolic process (GO:0006807)	6243	1433	1153.10	+	1.24	2.01E-18	7.44E-16
response to external stimulus (GO:0009605)	2574	590	475.43	+	1.24	7.33E-07	5.00E-05
cellular component organization or biogenesis (GO:0071840)	5458	1250	1008.11	+	1.24	3.63E-15	9.65E-13
organic substance metabolic process (GO:0071704)	7364	1668	1360.15	+	1.23	1.68E-20	7.65E-18
metabolic process (GO:0008152)	7826	1768	1445.49	+	1.22	7.34E-22	3.90E-19
reproductive process (GO:0022414)	1651	372	304.94	+	1.22	3.90E-04	1.35E-02
reproduction (GO:0000003)	1654	372	305.50	+	1.22	4.43E-04	1.50E-02
macromolecule metabolic process (GO:0043170)	5687	1275	1050.41	+	1.21	4.82E-13	9.27E-11
cellular component assembly (GO:0022607)	2208	493	407.82	+	1.21	7.59E-05	3.27E-03
cellular component biogenesis (GO:0044085)	2465	526	455.29	+	1.16	1.55E-03	4.34E-02
organic cyclic compound metabolic process (GO:1901360)	2695	571	497.77	+	1.15	1.62E-03	4.48E-02
biological regulation (GO:0065007)	12735	2645	2352.19	+	1.12	1.96E-18	7.45E-16
regulation of biological process (GO:0050789)	12042	2493	2224.19	+	1.12	2.28E-15	6.50E-13
regulation of cellular process (GO:0050794)	11596	2394	2141.82	+	1.12	1.56E-13	3.32E-11
cellular process (GO:0009987)	15558	3185	2873.61	+	1.11	1.03E-25	8.62E-23
biological_process (GO:0008150)	18079	3609	3339.25	+	1.08	1.65E-30	2.03E-27
system process (GO:0003008)	2787	378	514.77	-	.73	5.25E-10	6.55E-08
peptide biosynthetic process (GO:0043043)	497	57	91.80	-	.62	3.65E-04	1.27E-02
defense response to bacterium (GO:0042742)	372	42	68.71	-	.61	1.62E-03	4.47E-02
nervous system process (GO:0050877)	2244	248	414.47	-	.60	9.80E-18	3.33E-15
translation (GO:0006412)	479	52	88.47	-	.59	1.04E-04	4.27E-03
Unclassified (UNCLASSIFIED)	3507	378	647.75	-	.58	1.65E-30	2.20E-27
humoral immune response (GO:0006959)	302	30	55.78	-	.54	5.76E-04	1.86E-02
ribosome biogenesis (GO:0042254)	347	34	64.09	-	.53	1.52E-04	5.99E-03
leukocyte mediated immunity (GO:0002443)	271	24	50.05	-	.48	1.87E-04	7.13E-03
G protein-coupled receptor signaling pathway (GO:0007186)	2058	162	380.12	-	.43	2.04E-34	3.62E-31

cytoplasmic translation (GO:0002181)	155	12	28.63	-	.42	1.75E-03	4.74E-02
sensory perception (GO:0007600)	1719	111	317.50	-	.35	4.45E-38	8.88E-35
lymphocyte mediated immunity (GO:0002449)	225	14	41.56	-	.34	5.81E-06	3.31E-04
B cell receptor signaling pathway (GO:0050853)	123	7	22.72	-	.31	5.85E-04	1.88E-02
B cell mediated immunity (GO:0019724)	181	8	33.43	-	.24	1.47E-06	9.49E-05
ribosomal large subunit biogenesis (GO:0042273)	125	5	23.09	-	.22	4.53E-05	2.05E-03
immunoglobulin mediated immune response (GO:0016064)	176	7	32.51	-	.22	5.95E-07	4.14E-05
phagocytosis, recognition (GO:0006910)	104	4	19.21	-	.21	1.53E-04	6.02E-03
production of molecular mediator of immune response (GO:0002440)	178	6	32.88	-	.18	1.10E-07	9.01E-06
complement activation (GO:0006956)	128	4	23.64	-	.17	5.50E-06	3.14E-04
detection of stimulus (GO:0051606)	1426	44	263.39	-	.17	1.29E-58	2.95E-55
immunoglobulin production (GO:0002377)	174	5	32.14	-	.16	3.81E-08	3.36E-06
response to pheromone (GO:0019236)	99	2	18.29	-	.11	1.22E-05	6.47E-04
detection of stimulus involved in sensory perception (GO:0050906)	1320	24	243.81	-	.10	5.00E-68	1.33E-64
complement activation, classical pathway (GO:0006958)	114	2	21.06	-	.09	1.10E-06	7.22E-05
humoral immune response mediated by circulating immunoglobulin (GO:0002455)	119	2	21.98	-	.09	4.94E-07	3.51E-05
sensory perception of chemical stimulus (GO:0007606)	1310	19	241.96	-	.08	2.74E-72	8.74E-69
detection of chemical stimulus (GO:0009593)	1270	16	234.57	-	.07	8.86E-73	3.53E-69
sensory perception of smell (GO:0007608)	1226	11	226.45	-	.05	2.25E-75	1.20E-71
detection of chemical stimulus involved in sensory perception (GO:0050907)	1236	6	228.29	-	.03	7.46E-83	5.95E-79
detection of chemical stimulus involved in sensory perception of smell (GO:0050911)	1179	3	217.76	-	.01	2.32E-83	3.70E-79
ribosomal large subunit assembly (GO:0000027)	40	0	7.39	-	< 0.01	1.75E-03	4.74E-02

Suppleme mntary table S3 List of differentially expressed genes (DEGs at 6h)

ID	Log2 Fold Change	P-val	FDR P-val	Transcript ID(Array Design)
20506567	0,757231443	1,84E-06	0,0023	rno-miR-92b-5p
20535035	-1,084064265	9,63E-05	0,0491	rno-mir-200b
20501521	-0,466275727	0,0002	0,0491	rno-miR-200a-3p
20535720	-0,321454226	0,0002	0,0491	rno-mir-182
20501467	-1,78808091	0,0003	0,0491	rno-miR-141-3p
20506507	-0,280230021	0,0003	0,0491	rno-miR-182
20501523	-0,274076577	0,0004	0,0491	rno-miR-200b-3p
20501525	-0,407175382	0,0004	0,0491	rno-miR-203a-3p
20524321	0,272133025	0,0004	0,0491	rno-miR-3473
20524322	0,883402243	0,0004	0,0491	rno-miR-6215
20513720	0,240660713	0,0009	0,1043	rno-miR-1224
20500949	0,221442342	0,0012	0,1177	rno-miR-328a-5p
20501522	-0,544829314	0,0012	0,1177	rno-miR-200b-5p
20501526	-1,603167096	0,0015	0,1355	rno-miR-204-5p
20513758	-0,520664007	0,0016	0,1355	rno-miR-667-3p
20501520	-1,289784459	0,0017	0,1355	rno-miR-200a-5p
20502363	-0,42015647	0,0019	0,1406	rno-miR-429
20501466	-1,867362231	0,0021	0,1406	rno-miR-141-5p
20517116	0,704657567	0,0021	0,1406	rno-miR-1949
20501519	-0,279670407	0,0026	0,1653	rno-miR-200c-3p
20501424	-0,285104664	0,0029	0,1668	rno-miR-106b-5p
20517082	-0,75802721	0,0029	0,1668	rno-miR-3556b
20501354	-0,195034306	0,0033	0,179	rno-miR-22-3p
20506485	-0,877143252	0,0036	0,179	rno-miR-872-5p

S

20535080	-0,533978572	0,0036	0,179	rno-mir-421
20501528	-0,287783996	0,004	0,1945	rno-miR-205
20501020	-0,152415233	0,0042	0,1952	rno-miR-351-5p
20506533	-0,143238552	0,0048	0,2151	rno-miR-455-3p
20501351	-0,275357684	0,0054	0,2348	rno-miR-21-5p
20504139	-0,103884861	0,0065	0,2704	rno-miR-361-5p
20502439	-1,479167837	0,007	0,2823	rno-miR-431
20513765	1,918863237	0,0074	0,2855	rno-miR-678
20501371	-0,158567203	0,0076	0,2855	rno-miR-27a-3p
20501334	-0,312200463	0,0078	0,2855	rno-miR-10a-5p
20501479	1,338983259	0,0088	0,3042	rno-miR-150-3p
20522997	0,61667136	0,0091	0,3042	rno-miR-1199-3p
20501500	0,641952479	0,0095	0,3042	rno-miR-187-5p
20534742	-0,280107919	0,0095	0,3042	rno-mir-135b
20535003	-0,182122092	0,0096	0,3042	rno-mir-139
20517148	0,701313285	0,01	0,3042	rno-miR-702-5p
20535106	-0,529370175	0,0103	0,3042	rno-mir-365
20501385	-0,219337922	0,0105	0,3042	rno-miR-30b-5p
20513736	1,544320516	0,0105	0,3042	rno-miR-653-3p
20501493	-0,895217383	0,0107	0,3042	rno-miR-183-5p
20534973	-0,634350528	0,0112	0,3118	rno-mir-107
20506537	0,882643049	0,0115	0,313	rno-miR-471-3p
20501446	-0,138349431	0,0128	0,3417	rno-miR-130a-3p
20501377	-0,126184839	0,0139	0,3447	rno-miR-29a-3p
20501506	-0,709182438	0,0143	0,3447	rno-miR-192-5p
20501393	-1,288527523	0,0146	0,3447	rno-miR-31a-3p
20535034	-0,878693704	0,0155	0,3447	rno-mir-200a
20501015	-1,178902598	0,0156	0,3447	rno-miR-350
20513725	-1,723290657	0,0158	0,3447	rno-miR-504
20500933	-1,619519723	0,0159	0,3447	rno-miR-301a-3p

20501389	-0,152356219	0,016	0,3447	rno-miR-30a-5p
20536373	0,571156701	0,016	0,3447	rno-mir-299b
20524615	2,106915204	0,0167	0,3447	rno-miR-6332
20501481	-0,160991877	0,017	0,3447	rno-miR-152-3p
20501544	-0,986943847	0,0171	0,3447	rno-miR-217-5p
20517120	1,055282436	0,0172	0,3447	rno-miR-3573-5p
20536378	-0,489384841	0,0172	0,3447	rno-mir-9b-1
20535276	-0,382616022	0,0175	0,3447	rno-mir-494
20501369	-0,135283566	0,0176	0,3447	rno-miR-27b-3p
20534739	-0,138953156	0,0177	0,3447	rno-mir-351
20535734	-0,464403577	0,0199	0,3798	rno-mir-455
20501402	-0,188303531	0,0203	0,3798	rno-miR-34a-5p
20536059	1,040641984	0,0206	0,3798	rno-mir-632
20501516	-0,122777492	0,0207	0,3798	rno-miR-199a-5p
20535431	-1,321928095	0,0214	0,3877	rno-mir-497
20506523	-2,523561956	0,0224	0,3991	rno-miR-410-3p
20536050	-0,597035333	0,0228	0,3991	rno-mir-362
20500957	-1,584962501	0,0231	0,3991	rno-miR-330-5p
20535051	0,807354922	0,0238	0,3991	rno-mir-219a-1
20501387	-0,114902095	0,0243	0,3991	rno-miR-30d-5p
20506486	-0,765199275	0,0244	0,3991	rno-miR-872-3p
20506496	-1,459431619	0,0248	0,3991	rno-miR-881-3p
20501381	-0,129837438	0,025	0,3991	rno-miR-30c-5p
20506524	-0,739805546	0,025	0,3991	rno-miR-411-5p
20501032	-2,799406169	0,0258	0,3991	rno-miR-101b-3p
20524315	-0,823677227	0,026	0,3991	rno-miR-1843-5p
20501373	-0,156504486	0,0262	0,3991	rno-miR-28-3p
20501372	-0,168476408	0,0268	0,3991	rno-miR-28-5p
20501413	-0,124972887	0,0269	0,3991	rno-miR-99a-5p
20506543	-0,129617013	0,0276	0,3991	rno-miR-532-3p

20501398	-1	0,0281	0,3991	rno-miR-34b-5p
20524606	-0,707218251	0,0283	0,3991	rno-miR-6325
20502380	0,529839929	0,0288	0,3991	rno-miR-365-5p
20501431	-1,927068478	0,0292	0,3991	rno-miR-124-3p
20501388	-0,777607579	0,0301	0,3991	rno-miR-30d-3p
20529127	-0,759865996	0,0303	0,3991	rno-miR-155-3p
20501380	-1,025952696	0,0304	0,3991	rno-miR-29c-3p
20501003	-0,738119289	0,0308	0,3991	rno-miR-345-3p
20504212	-0,186413124	0,0309	0,3991	rno-miR-541-5p
20501352	1,007905198	0,0312	0,3991	rno-miR-21-3p
20534985	-0,277295099	0,0315	0,3991	rno-mir-125b-2
20500950	-0,22523891	0,032	0,3991	rno-miR-328a-3p
20535705	-0,708537186	0,032	0,3991	rno-mir-872
20536347	0,764187063	0,0323	0,3991	rno-mir-3547
20535041	-0,155278225	0,0324	0,3991	rno-mir-210
20534975	-0,726036034	0,0326	0,3991	rno-mir-124-3
20534977	-0,726036034	0,0326	0,3991	rno-mir-124-1
20534980	-0,726036034	0,0326	0,3991	rno-mir-124-2
20501409	-1,027480736	0,0332	0,4034	rno-miR-96-5p
20534963	-0,425021588	0,0351	0,4213	rno-mir-93
20535063	0,38827059	0,0357	0,4246	rno-mir-299a
20500945	-1,927354698	0,0361	0,4254	rno-miR-326-3p
20524591	-0,276840205	0,037	0,4266	rno-miR-344i
20537310	-0,694373717	0,037	0,4266	rno-mir-6324
20506542	-0,149211353	0,0374	0,4266	rno-miR-532-5p
20534964	0,693896872	0,0376	0,4266	rno-mir-96
20501399	-1,52420847	0,0381	0,4266	rno-miR-34b-3p
20524599	1,236298023	0,0382	0,4266	rno-miR-3075
20501029	-0,068221491	0,0394	0,4355	rno-miR-151-5p
20513715	-1,294620749	0,0397	0,4356	rno-miR-202-5p

20501361	-0,148196244	0,0404	0,4381	rno-miR-24-2-5p
20534987	-1,584962501	0,0407	0,4381	rno-mir-127
20500948	-0,324506639	0,0415	0,4403	rno-let-7d-3p
20534698	-0,271673324	0,0416	0,4403	rno-let-7d
20534972	-0,462343214	0,0423	0,4448	rno-mir-106b
20517161	1,124328135	0,0432	0,446	rno-miR-3593-3p
20501391	-0,268433169	0,0436	0,446	rno-miR-30c-2-3p
20501357	-2,738913866	0,0438	0,446	rno-miR-23b-5p
20517101	2,187627003	0,0439	0,446	rno-miR-3564
20500967	-1,226388809	0,0461	0,4591	rno-miR-335
20535040	0,514573173	0,0464	0,4591	rno-mir-208a
20517150	-0,584962501	0,0472	0,4591	rno-miR-3596a
20500944	0,649312912	0,0474	0,4591	rno-miR-326-5p
20501417	-0,102287882	0,048	0,4591	rno-miR-100-5p
20517118	0,800691192	0,0487	0,4591	rno-miR-1188-5p
20501030	-0,058738968	0,0492	0,4591	rno-miR-151-3p
20506555	-0,440572591	0,0497	0,4591	rno-miR-742-5p
20506527	-0,119119732	0,05	0,4591	rno-miR-423-3p
20536372	0,433653177	0,05	0,4591	rno-mir-299b

Supplememntary table S4. List of differentially expressed genes (DEGs at 2d)

ID	Log2 Fold Change	Log2 Difference	P-val	FDR P-val	Transcript ID(Array Design)
20506567	-0,074767768	-1,28	0,8481	0,9917	rno-miR-92b-5p
20535035	-0,011587974	-1,01	0,9666	0,9989	rno-mir-200b
20501521	0,06721508	1,24	0,8301	0,9891	rno-miR-200a-3p
20535720	0,064851144	1,22	0,6664	0,9645	rno-mir-182
20501467	-0,048094288	-1,04	0,8713	0,9917	rno-miR-141-3p
20506507	0,018562451	1,07	0,714	0,973	rno-miR-182
20501523	0,108376161	1,59	0,2697	0,8971	rno-miR-200b-3p
20501525	0,145086695	1,66	0,5517	0,959	rno-miR-203a-3p
20524321	0,073220919	1,44	0,3065	0,9087	rno-miR-3473
20524322	-0,167195209	-1,58	0,0465	0,8526	rno-miR-6215
20501522	0,020631333	1,05	0,9621	0,9989	rno-miR-200b-5p
20501526	0,080170349	1,05	0,5529	0,959	rno-miR-204-5p
20501520	0,215389034	1,3	0,8154	0,989	rno-miR-200a-5p
20502363	0,033788313	1,11	0,5103	0,9526	rno-miR-429
20501466	0,434402824	1,31	0,6609	0,9645	rno-miR-141-5p
20501519	0,076044191	1,34	0,7074	0,973	rno-miR-200c-3p
20517082	0,141355849	1,05	0,75	0,9797	rno-miR-3556b
20501354	0,037760804	1,19	0,6101	0,9623	rno-miR-22-3p
20506485	-0,014797002	-1,01	0,9121	0,9966	rno-miR-872-5p
20535080	-0,015106892	-1	0,8925	0,9917	rno-mir-421
20501528	0,047416781	1,22	0,9431	0,9989	rno-miR-205
20506533	-0,026265655	-1,1	0,6758	0,9645	rno-miR-455-3p
20504139	-0,066861954	-1,29	0,292	0,9087	rno-miR-361-5p
20502439	0,255729605	1,25	0,6005	0,9623	rno-miR-431
20501334	-0,073393259	-1,24	0,5575	0,959	rno-miR-10a-5p
20535003	-0,096628565	-1,31	0,3793	0,9303	rno-mir-139
20517148	-0,139251186	-1,3	0,9547	0,9989	rno-miR-702-5p

20535106	-0,173648087	-1,07	0,7485	0,9797	rno-mir-365
20501385	0,032637208	1,13	0,8532	0,9917	rno-miR-30b-5p
20501493	0,080919995	1,15	0,4954	0,9501	rno-miR-183-5p
20534973	0,123735368	1,04	0,6727	0,9645	rno-mir-107
20501446	-0,024405313	-1,09	0,494	0,9501	rno-miR-130a-3p
20501377	-0,015784567	-1,07	0,8375	0,9917	rno-miR-29a-3p
20500933	0,705140148	2,06	0,8402	0,9917	rno-miR-301a-3p
20501389	-0,059482424	-1,26	0,3344	0,9129	rno-miR-30a-5p
20524615	0,342392197	1,23	0,8669	0,9917	rno-miR-6332
20501481	-0,005018075	-1,02	0,4774	0,9501	rno-miR-152-3p
20501544	-0,165059246	-1,09	0,5819	0,959	rno-miR-217-5p
20501369	-0,024609467	-1,12	0,6624	0,9645	rno-miR-27b-3p
20535734	-0,094327383	-1,03	0,4578	0,9501	rno-mir-455
20501516	-0,011697591	-1,04	0,9673	0,9989	rno-miR-199a-5p
20500957	-0,459431619	-1,04	0,9861	0,9989	rno-miR-330-5p
20501387	-0,084662397	-1,36	0,2976	0,9087	rno-miR-30d-5p
20506486	0,104469267	1,13	0,8428	0,9917	rno-miR-872-3p
20506496	-0,101879614	-1,06	0,3752	0,9303	rno-miR-881-3p
20501381	-0,02611284	-1,12	0,6546	0,9645	rno-miR-30c-5p
20506524	-0,525784565	-1,53	0,254	0,8971	rno-miR-411-5p
20524315	0,025311089	1,03	0,4808	0,9501	rno-miR-1843-5p
20501373	-0,061400545	-1,21	0,7617	0,9825	rno-miR-28-3p
20501372	-0,039631787	-1,16	0,3108	0,9087	rno-miR-28-5p
20501413	-0,147917714	-1,78	0,0394	0,8526	rno-miR-99a-5p
20506543	0,013961674	1,05	0,8969	0,9926	rno-miR-532-3p
20524606	0,192645078	1,04	0,9	0,9926	rno-miR-6325
20501431	0,242570303	1,1	0,6324	0,9623	rno-miR-124-3p
20529127	0	1	0,8281	0,9891	rno-miR-155-3p
20501380	0,216257635	1,41	0,8038	0,989	rno-miR-29c-3p
20501003	0,147957881	1,21	0,8301	0,9891	rno-miR-345-3p

20501352	0,644171498	2,05	0,2545	0,8971	rno-miR-21-3p
20534985	-0,36804002	-1,26	0,0482	0,8526	rno-mir-125b-2
20500950	-0,065742963	-1,22	0,4875	0,9501	rno-miR-328a-3p
20535705	-0,053771256	-1,03	0,6888	0,9645	rno-mir-872
20534975	-0,40599236	-1,31	0,4911	0,9501	rno-mir-124-3
20534977	-0,40599236	-1,31	0,4911	0,9501	rno-mir-124-1
20534980	-0,40599236	-1,31	0,4911	0,9501	rno-mir-124-2
20500945	0,724026538	1,66	0,9145	0,9975	rno-miR-326-3p
20537310	0	1	0,9978	0,9989	rno-mir-6324
20501029	-0,076739443	-1,37	0,3297	0,9097	rno-miR-151-5p
20513715	-0,142019005	-1,04	0,3638	0,9153	rno-miR-202-5p
20501361	-0,026249899	-1,1	0,4966	0,9501	rno-miR-24-2-5p
20534972	-0,08246216	-1,04	0,6698	0,9645	rno-mir-106b
20501391	-0,341233618	-2,04	0,2677	0,8971	rno-miR-30c-2-3p
20517150	-0,15565087	-1,06	0,6021	0,9623	rno-miR-3596a
20501417	-0,14941029	-1,72	0,0897	0,8526	rno-miR-100-5p
20501030	-0,104239968	-1,44	0,3922	0,9303	rno-miR-151-3p
20501557	0,099699514	1,34	0,8821	0,9917	rno-miR-223-3p
20535238	-0,438884241	-1,12	0,5556	0,959	rno-mir-1
20501498	-0,115477217	-1,02	0,776	0,9833	rno-miR-186-5p
20501463	0	1,01	0,582	0,959	rno-miR-138-1-3p
20501464	-0,18143029	-2,08	0,4156	0,9438	rno-miR-139-5p
20506553	-0,211106614	-1,71	0,1905	0,8526	rno-miR-708-5p
20500975	-0,060204634	-1,11	0,8987	0,9926	rno-miR-148b-3p
20504486	-0,037393757	-1,19	0,2418	0,8971	rno-miR-378a-3p
20536066	0,042228235	1,07	0,9104	0,9956	rno-mir-665
20524323	-0,094215342	-1,46	0,0559	0,8526	rno-miR-378b
20535428	-0,182864057	-1,04	0,6828	0,9645	rno-mir-499
20535100	0	1	0,6213	0,9623	rno-mir-449a
20501775	-0,556213562	-2,84	0,1401	0,8526	rno-miR-196b-5p

20501415	-0,062503696	-1,32	0,3674	0,9205	rno-miR-99b-5p
20501432	-0,043327432	-1,23	0,3885	0,9303	rno-miR-125a-5p
20504258	-0,40275917	-1,15	0,0577	0,8526	rno-miR-369-5p
20501564	0,158697746	1,27	0,4704	0,9501	rno-miR-296-3p
20535230	-0,033997333	-1,12	0,8184	0,989	rno-mir-361
20535747	-0,145850866	-1,04	0,4422	0,9466	rno-mir-742
20501359	-0,258402957	-1,42	0,4919	0,9501	rno-miR-24-1-5p
20501484	-0,502500341	-1,15	0,7513	0,9797	rno-miR-154-5p
20537300	0,053111336	1,02	0,2624	0,8971	rno-mir-6316
20504485	-0,413482033	-2,91	0,0495	0,8526	rno-miR-378a-5p
20501358	-0,031554734	-1,2	0,3604	0,9153	rno-miR-23b-3p
20506572	-0,139330877	-1,74	0,193	0,8526	rno-miR-652-3p
20534720	0,731183242	1,25	0,3506	0,9148	rno-mir-341
20501374	-0,184059286	-1,34	0,5948	0,9623	rno-miR-29b-2-5p
20500935	-0,090670359	-1,29	0,5271	0,958	rno-miR-324-3p
20501002	-0,723638063	-3,23	0,0181	0,8526	rno-miR-345-5p
20534744	-0,48112669	-1,14	0,1311	0,8526	rno-mir-151
20501422	-0,020639766	-1,11	0,5948	0,9623	rno-miR-103-3p
20501776	-0,381249186	-1,97	0,6892	0,9645	rno-miR-196b-3p
20535731	-0,050185757	-1,12	0,4924	0,9501	rno-mir-423
20524594	0,49546435	2,14	0,2499	0,8971	rno-miR-344g
20500970	-0,499353785	-1,59	0,4083	0,9423	rno-miR-337-5p
20501510	0,073166413	1,2	0,4558	0,9501	rno-miR-194-5p
20524602	-1	-1,23	0,1943	0,8526	rno-miR-1298
20504495	-0,35614381	-1,05	0,6256	0,9623	rno-miR-497-3p
20501333	-0,428843299	-1,14	0,6299	0,9623	rno-miR-9a-3p
20524612	-0,229481846	-1,03	0,5575	0,959	rno-miR-6331
20500934	-0,092237949	-1,33	0,5846	0,959	rno-miR-324-5p
20501470	-0,06248413	-1,21	0,2808	0,9087	rno-miR-143-5p
20501512	-0,030631619	-1,14	0,3115	0,9087	rno-miR-195-5p

20501542	-0,530514717	-1,18	0,166	0,8526	rno-miR-216a-5p
20501451	-0,465663572	-1,12	0,1941	0,8526	rno-miR-133a-5p
20500994	-0,10581412	-1,48	0,4972	0,9501	rno-miR-342-3p
20504493	-0,556230782	-3,2	0,001	0,8526	rno-miR-664-2-5p
20534929	-0,160464672	-1,03	0,2788	0,9087	rno-mir-23a
20536352	0,161463423	1,06	0,1967	0,8526	rno-mir-3550
20506531	-0,173064709	-1,57	0,3621	0,9153	rno-miR-434-3p
20501508	-0,143952961	-1,58	0,1177	0,8526	rno-miR-193-5p
20501434	-0,065464314	-1,4	0,2094	0,8526	rno-miR-125b-5p
20506520	-0,327164743	-1,1	0,4112	0,9424	rno-miR-384-5p
20504251	-0,777148633	-3,66	0,2582	0,8971	rno-miR-487b-3p
20506476	0,678666954	3,55	0,2375	0,8958	rno-miR-466b-5p
20506807	-0,345135486	-1,07	0,1888	0,8526	rno-miR-551b-3p
20524311	-0,074000581	-1,25	0,1232	0,8526	rno-miR-1839-5p
20535425	-0,697971463	-1,21	0,4424	0,9466	rno-mir-378a
20535281	-0,423211431	-1,11	0,3075	0,9087	rno-mir-381
20537281	-0,762960803	-1,17	0,0789	0,8526	rno-mir-509
20506509	-1,087462841	-1,13	0,3842	0,9303	rno-miR-188-3p
20501565	1,392317423	3,52	0,0017	0,8526	rno-miR-297
20501390	-0,217043157	-1,91	0,0185	0,8526	rno-miR-30a-3p
20535232	-0,395928676	-1,04	0,2775	0,9087	rno-mir-224
20504214	-1,006484034	-4,74	0,0594	0,8526	rno-miR-542-5p
20535033	-0,334419039	-1,13	0,0858	0,8526	rno-mir-200c
20501433	-0,196397213	-1,7	0,0407	0,8526	rno-miR-125a-3p
20501436	-0,273537692	-1,99	0,0118	0,8526	rno-miR-125b-2-3p
20501348	#NUM!	-1,03	0,2466	0,8971	rno-miR-19b-2-5p
20502443	-1,035877036	-2,75	0,2042	0,8526	rno-miR-433-3p
20513718	-1,081388327	-5,65	0,0617	0,8526	rno-miR-490-3p
20501452	-0,140177658	-1,56	0,0788	0,8526	rno-miR-133a-3p
20500990	0,783606319	2,01	0,3031	0,9087	rno-miR-341

20501488	-0,078175073	-1,41	0,1398	0,8526	rno-miR-181a-5p
20536074	0,731661562	1,65	0,044	0,8526	rno-mir-667
20504238	-0,123472339	-1,38	0,0477	0,8526	rno-miR-379-5p
20500958	-0,59588615	-2,96	0,0135	0,8526	rno-miR-330-3p
20501384	-0,363109707	-1,8	0,2514	0,8971	rno-miR-30e-3p
20534950	-0,321928095	-1,18	0,2488	0,8971	rno-mir-30b
20501440	-0,266483768	-2,19	0,0059	0,8526	rno-miR-127-3p
20534935	-0,193307929	-1,31	0,1405	0,8526	rno-mir-25
20506505	-0,025901863	-1,08	0,169	0,8526	rno-miR-181d-5p
20501509	-0,332923102	-1,93	0,1462	0,8526	rno-miR-193-3p
20500954	-0,620476787	-2,22	0,2268	0,8793	rno-miR-329-3p
20504494	-0,108557803	-1,4	0,1182	0,8526	rno-miR-497-5p
20535011	-0,206450877	-1,12	0,2021	0,8526	rno-mir-152
20537318	0,423499078	1,22	0,1679	0,8526	rno-mir-3072
20501524	-1,087462841	-1,14	0,1608	0,8526	rno-miR-203a-5p
20504142	-0,528144369	-2,74	0,1107	0,8526	rno-miR-224-5p
20535022	-0,239670413	-1,1	0,133	0,8526	rno-mir-187
20501423	0,973032952	1,2	0,1768	0,8526	rno-miR-103-1-5p
20537284	0,267480311	1,08	0,1698	0,8526	rno-mir-6215
20504157	-0,389566812	-2,92	0,0317	0,8526	rno-miR-133b-3p
20513753	0,49539176	2,87	0,0491	0,8526	rno-miR-465-5p
20500974	-0,748461233	-1,12	0,164	0,8526	rno-miR-148b-5p
20513717	-0,625457279	-3,92	0,0529	0,8526	rno-miR-490-5p
20506503	0,387023123	1,29	0,1877	0,8526	rno-miR-147
20506479	0,618909833	1,37	0,1599	0,8526	rno-miR-466c-5p
20517122	1,652076697	1,23	0,1712	0,8526	rno-miR-1193-5p
20536047	-0,377069649	-1,18	0,0339	0,8526	rno-mir-490
20501553	0,130153236	1,69	0,0047	0,8526	rno-miR-221-3p
20501560	0,721698838	1,29	0,1299	0,8526	rno-miR-291a-3p
20501315	-0,031187878	-1,19	0,1159	0,8526	rno-let-7a-5p

20501418	-0,64385619	-1,13	0,054	0,8526	rno-miR-100-3p
20535038	-0,678071905	-1,18	0,0862	0,8526	rno-mir-205
20534989	-0,560300446	-1,22	0,1262	0,8526	rno-mir-128-2
20501323	-0,080531338	-1,54	0,0541	0,8526	rno-let-7e-5p
20504215	-1,078002512	-1,32	0,0851	0,8526	rno-miR-542-3p
20501006	0,652515272	3,29	0,1004	0,8526	rno-miR-346
20506804	0,090558219	1,46	0,0323	0,8526	rno-miR-146b-5p
20506571	-0,532013621	-3,15	0,0136	0,8526	rno-miR-652-5p
20513760	0,722466024	1,2	0,0802	0,8526	rno-miR-764-5p
20504237	1,378511623	1,84	0,0196	0,8526	rno-miR-493-3p
20536072	0,641546029	1,21	0,0184	0,8526	rno-mir-465
20536077	0,687212559	1,28	0,0332	0,8526	rno-mir-666
20501018	1,247927513	2,31	0,05	0,8526	rno-miR-7a-5p
20504155	-0,266876472	-2,42	0,0198	0,8526	rno-miR-1-3p
20501395	1,422233001	5,71	0,026	0,8526	rno-miR-32-3p
20536432	1,071790683	2,12	0,0109	0,8526	rno-mir-328b
20524617	-0,937264245	-1,35	0,041	0,8526	rno-miR-6334
20504252	-0,421415069	-2,54	0,0298	0,8526	rno-miR-382-5p
20537302	1,690315501	1,4	0,0411	0,8526	rno-mir-6318
20501514	-1,225465255	-6,46	0,0394	0,8526	rno-miR-196a-5p
20501318	-0,046281275	-1,32	0,0134	0,8526	rno-let-7b-5p
20501320	-0,045662242	-1,33	0,0094	0,8526	rno-let-7c-5p

Supplementary table S5. List of differentially expressed genes (DEGs) at 8d

ID	Log2 Fold Change	P-val	FDR P-val	Transcript ID(Array Design)
20506567	0,367643813	0,0016	0,1501	rno-miR-92b-5p
20500957	-3,044394119	0,0135	0,3194	rno-miR-330-5p
20501466	-2,376375879	0,0008	0,0966	rno-miR-141-5p
20500933	-2,313416595	0,0023	0,1736	rno-miR-301a-3p
20513725	-2,165808893	0,0094	0,2757	rno-miR-504
20501526	-1,778253803	0,0008	0,0966	rno-miR-204-5p
20524321	0,350732124	1,60E-05	0,02	rno-miR-3473
20513720	0,209758433	0,0005	0,0832	rno-miR-1224
20500949	0,159297821	0,0216	0,3679	rno-miR-328a-5p
20506523	-1,353636955	0,048	0,5132	rno-miR-410-3p
20513715	-1,294620749	0,0122	0,3055	rno-miR-202-5p
20517116	1,00994966	0,0001	0,0349	rno-miR-1949
20534975	-1,231564067	0,0042	0,1874	rno-mir-124-3
20534977	-1,231564067	0,0042	0,1874	rno-mir-124-1
20534980	-1,231564067	0,0042	0,1874	rno-mir-124-2
20501544	-1,166649869	0,0041	0,1874	rno-miR-217-5p
20501479	1,246695669	0,0429	0,4922	rno-miR-150-3p
20506485	-1,08246216	0,0018	0,1651	rno-miR-872-5p
20501431	-1,043392596	0,05	0,5168	rno-miR-124-3p
20537310	-0,861023587	0,0112	0,3043	rno-mir-6324
20513736	1,874469118	0,0194	0,3483	rno-miR-653-3p
20501467	-0,852569636	0,0032	0,1874	rno-miR-141-3p
20517082	-0,845490051	0,0008	0,0966	rno-miR-3556b
20501506	-0,819427754	0,0122	0,3055	rno-miR-192-5p
20535106	-0,703018262	0,0066	0,2302	rno-mir-365
20524606	-0,649502753	0,022	0,3679	rno-miR-6325

20506524	-0,597786541	0,0421	0,492	rno-miR-411-5p
20534985	-0,365952719	0,0038	0,1874	rno-mir-125b-2
20500948	-0,350548773	0,0268	0,4182	rno-let-7d-3p
20535003	-0,349652578	8,13E-05	0,0339	rno-mir-139
20501521	-0,255982783	0,0077	0,2468	rno-miR-200a-3p
20501525	-0,248913298	0,009	0,2754	rno-miR-203a-3p
20501389	-0,21367999	0,0037	0,1874	rno-miR-30a-5p
20500950	-0,205430429	0,0346	0,4786	rno-miR-328a-3p
20501413	-0,204091605	0,0059	0,2143	rno-miR-99a-5p
20501385	-0,200050268	0,0068	0,2302	rno-miR-30b-5p
20501361	-0,180572246	0,0036	0,1874	rno-miR-24-2-5p
20501417	-0,168461292	0,0162	0,3291	rno-miR-100-5p
20501369	-0,152157384	0,0109	0,3037	rno-miR-27b-3p
20501352	1,266992419	0,0333	0,4786	rno-miR-21-3p
20501387	-0,150853077	0,0198	0,3483	rno-miR-30d-5p
20506507	-0,149346545	0,0413	0,4917	rno-miR-182
20501372	-0,144477089	0,0224	0,3679	rno-miR-28-5p
20501381	-0,14446771	0,0056	0,2143	rno-miR-30c-5p
20501377	-0,134055536	0,0138	0,32	rno-miR-29a-3p
20535720	-0,124669417	0,05	0,5168	rno-mir-182
20501371	-0,119928176	0,0181	0,3333	rno-miR-27a-3p
20501446	-0,118016429	0,0087	0,2716	rno-miR-130a-3p
20501481	-0,114979479	0,0159	0,3291	rno-miR-152-3p
20504139	-0,091576602	0,0426	0,4922	rno-miR-361-5p
20501375	-0,95247163	0,0179	0,3333	rno-miR-29b-3p
20535238	-0,550197083	0,0359	0,4786	rno-mir-1
20501463	-2,146841388	0,0365	0,4786	rno-miR-138-1-3p
20501464	-0,270720599	0,0009	0,0966	rno-miR-139-5p
20500975	-1,320621897	0,0056	0,2143	rno-miR-148b-3p
20501392	0,148214004	0,0037	0,1874	rno-miR-31a-5p

20504486	-0,116302524	0,0234	0,3798	rno-miR-378a-3p
20524323	-0,124127346	0,0175	0,3333	rno-miR-378b
20535428	-0,628031223	0,0334	0,4786	rno-mir-499
20504210	-1,611434712	0,0489	0,5137	rno-miR-539-5p
20504258	-0,840801405	0,0255	0,4086	rno-miR-369-5p
20501564	0,78447631	0,0024	0,1736	rno-miR-296-3p
20501359	-1,196969824	0,006	0,2143	rno-miR-24-1-5p
20501566	1,641685091	0,0003	0,0575	rno-miR-298-5p
20501374	-0,767681614	0,0146	0,3264	rno-miR-29b-2-5p
20501324	-1,05626822	0,0323	0,4747	rno-let-7e-3p
20501541	0,156798577	6,88E-05	0,0339	rno-miR-214-3p
20524594	1,539928747	0,0042	0,1874	rno-miR-344g
20524612	-0,949373927	0,0453	0,5031	rno-miR-6331
20501470	-0,3104932	0,0032	0,1874	rno-miR-143-5p
20501512	-0,118356849	0,0156	0,3291	rno-miR-195-5p
20529126	0,322892786	0,0023	0,1736	rno-miR-155-5p
20501451	-2,233313708	0,0131	0,3188	rno-miR-133a-5p
20534929	-0,978626349	0,0153	0,3291	rno-mir-23a
20517129	-0,5360529	0,0459	0,5031	rno-miR-1912-3p
20501508	-0,128277304	0,0353	0,4786	rno-miR-193-5p
20535721	0,811927652	0,0057	0,2143	rno-mir-188
20524593	-1,519374159	0,006	0,2143	rno-miR-3102
20501476	0,156725504	0,0391	0,4851	rno-miR-146a-5p
20537281	-0,449307401	0,0383	0,4837	rno-mir-509
20501390	-0,223067999	0,0148	0,3264	rno-miR-30a-3p
20535232	-0,847996907	0,0352	0,4786	rno-mir-224
20535033	-0,277984747	0,0472	0,5091	rno-mir-200c
20517069	1,033947332	0,0119	0,3055	rno-miR-3085
20500978	-1,898913875	0,0001	0,0339	rno-miR-338-5p
20513718	-0,69632361	0,0095	0,2757	rno-miR-490-3p

20501452	-0,453956489	0,0163	0,3291	rno-miR-133a-3p
20501024	-0,127944503	0,0369	0,4786	rno-miR-352
20500958	-0,351187037	0,0172	0,3333	rno-miR-330-3p
20501384	-2,042644337	0,0054	0,2143	rno-miR-30e-3p
20501555	0,116171522	0,0361	0,4786	rno-miR-222-3p
20534926	0,664815808	0,0466	0,506	rno-mir-19a
20501509	-0,475272606	0,0222	0,3679	rno-miR-193-3p
20513747	-0,352301744	0,0403	0,4851	rno-miR-201-3p
20501366	-0,605107235	0,0485	0,5132	rno-miR-26b-5p
20500954	-1,753716958	0,0106	0,3006	rno-miR-329-3p
20535011	-0,365871442	0,0456	0,5031	rno-mir-152
20501495	1,098032083	0,0175	0,3333	rno-miR-184
20534716	0,795641501	0,0149	0,3264	rno-mir-339
20501524	-1,070389328	0,0457	0,5031	rno-miR-203a-5p
20504142	-1,599684006	0,0009	0,0966	rno-miR-224-5p
20504157	-0,454713273	0,0419	0,492	rno-miR-133b-3p
20513717	-0,411918157	0,0264	0,4176	rno-miR-490-5p
20537982	0,295455884	0,0369	0,4786	rno-mir-155
20501426	-1,754887502	0,0371	0,4786	rno-miR-107-5p
20536047	-0,490325627	0,0195	0,3483	rno-mir-490
20501553	0,194185596	0,007	0,2306	rno-miR-221-3p
20535289	0,425606741	0,0181	0,3333	rno-mir-20b
20534701	-0,345135486	0,0319	0,4747	rno-mir-329
20502357	-2,5360529	0,0395	0,4851	rno-miR-448-5p
20537306	0,652076697	0,0305	0,4591	rno-mir-6321
20502377	0,842458723	0,0445	0,5031	rno-miR-450a-5p
20506804	0,136866713	0,0377	0,4814	rno-miR-146b-5p
20504263	1,876617584	0,0214	0,3679	rno-miR-363-3p
20536051	0,38332864	0,0398	0,4851	rno-mir-511
20535005	0,740912779	0,0133	0,3188	rno-mir-142

20504155	-0,3551811	0,0115	0,3055	rno-miR-1-3p
20501318	0,058389403	0,0347	0,4786	rno-let-7b-5p
20501403	1,47883415	0,0285	0,4337	rno-miR-34a-3p
20534708	0,646767785	0,0279	0,4304	rno-mir-335
20501448	0,387693832	0,0404	0,4851	rno-miR-130b-3p

Supplementary table S6. List of differentially expressed genes at 6h and the associated over and under represented Biological Processes. Raw Panther results for all DEGs

Analysis Type:	PANTHER Overrepresentation Test (Released 20200728)
Annotation Version and Release Date:	GO Ontology database DOI: 10.5281/zenodo.4081749 Released 2020-10-09
Analyzed List:	Client Text Box Input (Rattus norvegicus)
Reference List:	Rattus norvegicus (all genes in database)
Test Type:	FISHER
Correction:	FDR

GO biological process complete	Rattus norvegicus - REFLIST (21677)	Client Text Box Input (1353)	Client Text Box Input (expected)	Client Text Box Input (over/under)	Client Text Box Input (fold Enrichment)	Client Text Box Input (raw P-value)	Client Text Box Input (FDR)
positive regulation of cyclic-nucleotide phosphodiesterase activity (GO:0051343)	5	4	.31	+	12.82	1.18E-03	4.06E-02
exosomal secretion (GO:1990182)	5	4	.31	+	12.82	1.18E-03	4.05E-02
mesonephric tubule formation (GO:0072172)	10	5	.62	+	8.01	1.27E-03	4.27E-02
negative regulation of cellular senescence (GO:2000773)	20	7	1.25	+	5.61	7.46E-04	2.81E-02
protein mannosylation (GO:0035268)	22	7	1.37	+	5.10	1.18E-03	4.07E-02
positive regulation of morphogenesis of an epithelium (GO:1905332)	36	11	2.25	+	4.90	6.77E-05	3.77E-03
positive regulation of branching involved in ureteric bud morphogenesis (GO:0090190)	23	7	1.44	+	4.88	1.47E-03	4.82E-02

negative regulation of cartilage development (GO:0061037)	30	9	1.87	+	4.81	3.46E-04	1.48E-02
regulation of kidney development (GO:0090183)	35	10	2.18	+	4.58	2.28E-04	1.04E-02
RNA destabilization (GO:0050779)	35	10	2.18	+	4.58	2.28E-04	1.04E-02
regulation of release of sequestered calcium ion into cytosol by sarcoplasmic reticulum (GO:0010880)	28	8	1.75	+	4.58	9.60E-04	3.42E-02
mRNA destabilization (GO:0061157)	32	9	2.00	+	4.51	5.14E-04	2.08E-02
positive regulation of pri-miRNA transcription by RNA polymerase II (GO:1902895)	44	11	2.75	+	4.01	3.02E-04	1.32E-02
negative regulation of cell-matrix adhesion (GO:0001953)	37	9	2.31	+	3.90	1.24E-03	4.19E-02
developmental cell growth (GO:0048588)	104	25	6.49	+	3.85	1.14E-07	1.35E-05
regulation of cellular senescence (GO:2000772)	42	10	2.62	+	3.81	7.79E-04	2.91E-02
peptidyl-threonine phosphorylation (GO:0018107)	73	17	4.56	+	3.73	1.68E-05	1.12E-03
cellular polysaccharide biosynthetic process (GO:0033692)	43	10	2.68	+	3.73	9.11E-04	3.29E-02
cell fate determination (GO:0001709)	43	10	2.68	+	3.73	9.11E-04	3.29E-02
polysaccharide biosynthetic process (GO:0000271)	43	10	2.68	+	3.73	9.11E-04	3.28E-02
cell growth (GO:0016049)	108	25	6.74	+	3.71	2.11E-07	2.27E-05
mitochondrion localization (GO:0051646)	48	11	3.00	+	3.67	5.70E-04	2.25E-02
peptidyl-threonine modification (GO:0018210)	81	18	5.06	+	3.56	1.66E-05	1.11E-03

regulation of morphogenesis of an epithelium (GO:1905330)	68	15	4.24	+	3.53	8.83E-05	4.67E-03
smooth muscle cell differentiation (GO:0051145)	46	10	2.87	+	3.48	1.42E-03	4.69E-02
positive regulation of mRNA catabolic process (GO:0061014)	51	11	3.18	+	3.46	8.81E-04	3.21E-02
aminoglycan biosynthetic process (GO:0006023)	52	11	3.25	+	3.39	1.01E-03	3.58E-02
cellular carbohydrate biosynthetic process (GO:0034637)	57	12	3.56	+	3.37	6.29E-04	2.43E-02
regulation of pri-miRNA transcription by RNA polymerase II (GO:1902893)	57	12	3.56	+	3.37	6.29E-04	2.43E-02
multicellular organismal water homeostasis (GO:0050891)	59	12	3.68	+	3.26	8.20E-04	3.04E-02
negative regulation of protein depolymerization (GO:1901880)	74	15	4.62	+	3.25	1.99E-04	9.27E-03
negative regulation of translation (GO:0017148)	134	27	8.36	+	3.23	7.64E-07	6.95E-05
protein O-linked glycosylation (GO:0006493)	55	11	3.43	+	3.20	1.50E-03	4.89E-02
regulation of morphogenesis of a branching structure (GO:0060688)	66	13	4.12	+	3.16	6.58E-04	2.52E-02
negative regulation of cellular amide metabolic process (GO:0034249)	151	29	9.42	+	3.08	7.16E-07	6.59E-05
regulation of protein depolymerization (GO:1901879)	95	18	5.93	+	3.04	1.02E-04	5.20E-03
regulation of glucose import (GO:0046324)	74	14	4.62	+	3.03	5.89E-04	2.31E-02

water homeostasis (GO:0030104)	69	13	4.31	+	3.02	9.44E-04	3.38E-02
visual behavior (GO:0007632)	75	14	4.68	+	2.99	6.62E-04	2.53E-02
cellular response to dexamethasone stimulus (GO:0071549)	70	13	4.37	+	2.98	1.06E-03	3.73E-02
proteoglycan metabolic process (GO:0006029)	70	13	4.37	+	2.98	1.06E-03	3.72E-02
neuron projection extension (GO:1990138)	70	13	4.37	+	2.98	1.06E-03	3.71E-02
negative regulation of transporter activity (GO:0032410)	87	16	5.43	+	2.95	3.26E-04	1.41E-02
pigmentation (GO:0043473)	87	16	5.43	+	2.95	3.26E-04	1.40E-02
visual learning (GO:0008542)	71	13	4.43	+	2.93	1.19E-03	4.07E-02
metanephros development (GO:0001656)	88	16	5.49	+	2.91	3.65E-04	1.54E-02
mesonephric tubule development (GO:0072164)	99	18	6.18	+	2.91	1.61E-04	7.65E-03
mesonephric epithelium development (GO:0072163)	99	18	6.18	+	2.91	1.61E-04	7.63E-03
developmental growth involved in morphogenesis (GO:0060560)	132	24	8.24	+	2.91	1.41E-05	9.51E-04
bone morphogenesis (GO:0060349)	111	20	6.93	+	2.89	7.96E-05	4.29E-03
positive regulation of mRNA metabolic process (GO:1903313)	89	16	5.56	+	2.88	4.07E-04	1.70E-02
negative regulation of ion transmembrane transporter activity (GO:0032413)	78	14	4.87	+	2.88	9.28E-04	3.33E-02
negative regulation of protein-containing complex disassembly (GO:0043242)	84	15	5.24	+	2.86	6.49E-04	2.49E-02

retrograde transport, endosome to Golgi (GO:0042147)	80	14	4.99	+	2.80	1.15E-03	3.98E-02
mesonephros development (GO:0001823)	103	18	6.43	+	2.80	2.48E-04	1.11E-02
response to dexamethasone (GO:0071548)	98	17	6.12	+	2.78	3.92E-04	1.65E-02
ureteric bud development (GO:0001657)	98	17	6.12	+	2.78	3.92E-04	1.65E-02
regulation of alternative mRNA splicing, via spliceosome (GO:0000381)	81	14	5.06	+	2.77	1.28E-03	4.29E-02
organ growth (GO:0035265)	99	17	6.18	+	2.75	4.35E-04	1.80E-02
glycoprotein biosynthetic process (GO:0009101)	245	42	15.29	+	2.75	6.99E-08	8.96E-06
sodium ion transmembrane transport (GO:0035725)	111	19	6.93	+	2.74	2.13E-04	9.85E-03
regulation of cell-matrix adhesion (GO:0001952)	123	21	7.68	+	2.74	1.05E-04	5.34E-03
cellular response to carbohydrate stimulus (GO:0071322)	129	22	8.05	+	2.73	7.37E-05	4.00E-03
negative regulation of cellular response to growth factor stimulus (GO:0090288)	100	17	6.24	+	2.72	4.82E-04	1.96E-02
regulation of mRNA stability (GO:0043488)	106	18	6.62	+	2.72	3.37E-04	1.45E-02
regulation of protein-containing complex disassembly (GO:0043244)	130	22	8.11	+	2.71	8.15E-05	4.38E-03
positive regulation of smooth muscle cell proliferation (GO:0048661)	114	19	7.12	+	2.67	2.88E-04	1.27E-02
negative regulation of transmembrane transport (GO:0034763)	138	23	8.61	+	2.67	6.97E-05	3.86E-03

positive regulation of autophagy (GO:0010508)	133	22	8.30	+	2.65	1.09E-04	5.53E-03
cellular response to monosaccharide stimulus (GO:0071326)	115	19	7.18	+	2.65	3.17E-04	1.38E-02
associative learning (GO:0008306)	115	19	7.18	+	2.65	3.17E-04	1.37E-02
regulation of axon extension (GO:0030516)	116	19	7.24	+	2.62	3.49E-04	1.49E-02
embryonic epithelial tube formation (GO:0001838)	153	25	9.55	+	2.62	6.59E-05	3.68E-03
viral life cycle (GO:0019058)	98	16	6.12	+	2.62	1.02E-03	3.60E-02
cellular response to corticosteroid stimulus (GO:0071384)	123	20	7.68	+	2.61	4.38E-04	1.80E-02
glycoprotein metabolic process (GO:0009100)	303	49	18.91	+	2.59	2.88E-08	4.24E-06
cellular response to steroid hormone stimulus (GO:0071383)	192	31	11.98	+	2.59	8.65E-06	6.16E-04
regulation of RNA stability (GO:0043487)	118	19	7.37	+	2.58	6.65E-04	2.53E-02
aminoglycan metabolic process (GO:0006022)	106	17	6.62	+	2.57	8.62E-04	3.17E-02
response to osmotic stress (GO:0006970)	100	16	6.24	+	2.56	1.23E-03	4.17E-02
mitotic cell cycle phase transition (GO:0044772)	150	24	9.36	+	2.56	1.12E-04	5.62E-03
tube formation (GO:0035148)	175	28	10.92	+	2.56	2.75E-05	1.69E-03
regulation of extent of cell growth (GO:0061387)	132	21	8.24	+	2.55	3.41E-04	1.46E-02
protein glycosylation (GO:0006486)	195	31	12.17	+	2.55	1.08E-05	7.53E-04
macromolecule glycosylation (GO:0043413)	195	31	12.17	+	2.55	1.08E-05	7.50E-04

cardiocyte differentiation (GO:0035051)	126	20	7.86	+	2.54	4.96E-04	2.01E-02
positive regulation of developmental growth (GO:0048639)	216	34	13.48	+	2.52	4.69E-06	3.67E-04
epithelial tube formation (GO:0072175)	160	25	9.99	+	2.50	1.00E-04	5.14E-03
cell cycle phase transition (GO:0044770)	160	25	9.99	+	2.50	1.00E-04	5.12E-03
learning (GO:0007612)	186	29	11.61	+	2.50	2.76E-05	1.69E-03
regulation of mRNA catabolic process (GO:0061013)	129	20	8.05	+	2.48	5.82E-04	2.29E-02
morphogenesis of embryonic epithelium (GO:0016331)	176	27	10.99	+	2.46	6.55E-05	3.67E-03
glycosylation (GO:0070085)	210	32	13.11	+	2.44	2.15E-05	1.39E-03
dendrite development (GO:0016358)	138	21	8.61	+	2.44	4.87E-04	1.98E-02
calcium-mediated signaling (GO:0019722)	145	22	9.05	+	2.43	3.63E-04	1.54E-02
cellular glucose homeostasis (GO:0001678)	133	20	8.30	+	2.41	7.48E-04	2.81E-02
kidney epithelium development (GO:0072073)	147	22	9.18	+	2.40	4.18E-04	1.73E-02
response to hydrogen peroxide (GO:0042542)	168	25	10.49	+	2.38	2.77E-04	1.23E-02
positive regulation of catabolic process (GO:0009896)	451	67	28.15	+	2.38	1.15E-09	2.15E-07
regulation of animal organ morphogenesis (GO:2000027)	128	19	7.99	+	2.38	1.15E-03	3.98E-02
positive regulation of cellular catabolic process (GO:0031331)	384	57	23.97	+	2.38	2.69E-08	3.99E-06
positive regulation of epithelial cell migration (GO:0010634)	150	22	9.36	+	2.35	5.19E-04	2.09E-02

small GTPase mediated signal transduction (GO:0007264)	232	34	14.48	+	2.35	1.87E-05	1.23E-03
learning or memory (GO:0007611)	323	47	20.16	+	2.33	5.94E-07	5.57E-05
regulation of translation (GO:0006417)	352	51	21.97	+	2.32	2.69E-07	2.80E-05
cerebral cortex development (GO:0021987)	145	21	9.05	+	2.32	1.23E-03	4.16E-02
regulation of signaling receptor activity (GO:0010469)	159	23	9.92	+	2.32	6.35E-04	2.44E-02
neural tube formation (GO:0001841)	132	19	8.24	+	2.31	1.50E-03	4.89E-02
Ras protein signal transduction (GO:0007265)	154	22	9.61	+	2.29	9.45E-04	3.38E-02
response to endoplasmic reticulum stress (GO:0034976)	217	31	13.54	+	2.29	9.90E-05	5.11E-03
endocrine system development (GO:0035270)	147	21	9.18	+	2.29	1.31E-03	4.37E-02
protein localization to plasma membrane (GO:0072659)	205	29	12.80	+	2.27	1.30E-04	6.37E-03
positive regulation of growth (GO:0045927)	304	43	18.97	+	2.27	4.44E-06	3.50E-04
sodium ion transport (GO:0006814)	170	24	10.61	+	2.26	5.57E-04	2.21E-02
negative regulation of cytoskeleton organization (GO:0051494)	163	23	10.17	+	2.26	7.54E-04	2.83E-02
positive regulation of cell growth (GO:0030307)	192	27	11.98	+	2.25	2.45E-04	1.11E-02
regulation of neuron differentiation (GO:0045664)	235	33	14.67	+	2.25	6.12E-05	3.48E-03
pallium development (GO:0021543)	228	32	14.23	+	2.25	8.29E-05	4.44E-03

regulation of developmental growth (GO:0048638)	392	55	24.47	+	2.25	2.18E-07	2.33E-05
respiratory system development (GO:0060541)	279	39	17.41	+	2.24	1.73E-05	1.15E-03
vesicle organization (GO:0016050)	258	36	16.10	+	2.24	2.83E-05	1.72E-03
lung development (GO:0030324)	245	34	15.29	+	2.22	5.32E-05	3.07E-03
regulation of endothelial cell migration (GO:0010594)	166	23	10.36	+	2.22	8.80E-04	3.21E-02
cognition (GO:0050890)	361	50	22.53	+	2.22	1.17E-06	1.03E-04
negative regulation of supramolecular fiber organization (GO:1902904)	159	22	9.92	+	2.22	1.19E-03	4.07E-02
positive regulation of binding (GO:0051099)	196	27	12.23	+	2.21	4.42E-04	1.81E-02
peptidyl-serine phosphorylation (GO:0018105)	189	26	11.80	+	2.20	6.08E-04	2.37E-02
posttranscriptional regulation of gene expression (GO:0010608)	459	63	28.65	+	2.20	7.58E-08	9.41E-06
second-messenger-mediated signaling (GO:0019932)	328	45	20.47	+	2.20	5.83E-06	4.47E-04
ossification (GO:0001503)	248	34	15.48	+	2.20	9.54E-05	4.98E-03
respiratory tube development (GO:0030323)	248	34	15.48	+	2.20	9.54E-05	4.96E-03
bone development (GO:0060348)	219	30	13.67	+	2.19	1.90E-04	8.91E-03
signal transduction by protein phosphorylation (GO:0023014)	190	26	11.86	+	2.19	6.26E-04	2.43E-02
positive regulation of protein catabolic process (GO:0045732)	220	30	13.73	+	2.18	2.00E-04	9.28E-03

protein localization to cell periphery (GO:1990778)	257	35	16.04	+	2.18	7.35E-05	4.00E-03
regulation of cellular amide metabolic process (GO:0034248)	405	55	25.28	+	2.18	5.75E-07	5.49E-05
peptidyl-serine modification (GO:0018209)	215	29	13.42	+	2.16	2.96E-04	1.30E-02
regulation of autophagy (GO:0010506)	262	35	16.35	+	2.14	8.92E-05	4.70E-03
regulation of stress-activated protein kinase signaling cascade (GO:0070302)	188	25	11.73	+	2.13	1.02E-03	3.59E-02
axonogenesis (GO:0007409)	354	47	22.10	+	2.13	7.67E-06	5.66E-04
epithelial tube morphogenesis (GO:0060562)	370	49	23.09	+	2.12	4.53E-06	3.56E-04
regulation of mRNA metabolic process (GO:1903311)	272	36	16.98	+	2.12	7.73E-05	4.18E-03
plasma membrane bounded cell projection morphogenesis (GO:0120039)	492	65	30.71	+	2.12	1.27E-07	1.47E-05
cellular response to oxygen levels (GO:0071453)	197	26	12.30	+	2.11	8.23E-04	3.04E-02
neuron projection morphogenesis (GO:0048812)	487	64	30.40	+	2.11	1.85E-07	2.04E-05
cell projection morphogenesis (GO:0048858)	497	65	31.02	+	2.10	2.32E-07	2.46E-05
neural tube development (GO:0021915)	192	25	11.98	+	2.09	1.21E-03	4.13E-02
skeletal system morphogenesis (GO:0048705)	254	33	15.85	+	2.08	2.11E-04	9.75E-03
positive regulation of neurogenesis (GO:0050769)	287	37	17.91	+	2.07	1.11E-04	5.58E-03
developmental growth (GO:0048589)	466	60	29.09	+	2.06	7.97E-07	7.21E-05

regulation of catabolic process (GO:0009894)	873	112	54.49	+	2.06	1.48E-11	5.51E-09
regulation of epithelial cell migration (GO:0010632)	234	30	14.61	+	2.05	5.20E-04	2.08E-02
cell part morphogenesis (GO:0032990)	524	67	32.71	+	2.05	2.88E-07	2.96E-05
axon development (GO:0061564)	399	51	24.90	+	2.05	6.56E-06	4.89E-04
cell morphogenesis involved in neuron differentiation (GO:0048667)	447	57	27.90	+	2.04	2.03E-06	1.68E-04
endosomal transport (GO:0016197)	212	27	13.23	+	2.04	1.21E-03	4.13E-02
growth (GO:0040007)	473	60	29.52	+	2.03	1.41E-06	1.22E-04
regulation of cell size (GO:0008361)	221	28	13.79	+	2.03	9.68E-04	3.44E-02
angiogenesis (GO:0001525)	285	36	17.79	+	2.02	1.83E-04	8.59E-03
regulation of Wnt signaling pathway (GO:0030111)	301	38	18.79	+	2.02	1.54E-04	7.35E-03
regulation of cellular catabolic process (GO:0031329)	722	91	45.06	+	2.02	3.05E-09	5.25E-07
regulation of growth (GO:0040008)	683	86	42.63	+	2.02	8.17E-09	1.34E-06
regulation of protein catabolic process (GO:0042176)	390	49	24.34	+	2.01	1.99E-05	1.30E-03
positive regulation of cytoskeleton organization (GO:0051495)	215	27	13.42	+	2.01	1.35E-03	4.48E-02
telencephalon development (GO:0021537)	328	41	20.47	+	2.00	1.17E-04	5.85E-03
cellular component morphogenesis (GO:0032989)	617	77	38.51	+	2.00	7.08E-08	9.00E-06
organic acid catabolic process (GO:0016054)	225	28	14.04	+	1.99	1.15E-03	3.99E-02

carboxylic acid catabolic process (GO:0046395)	225	28	14.04	+	1.99	1.15E-03	3.98E-02
endomembrane system organization (GO:0010256)	394	49	24.59	+	1.99	2.21E-05	1.42E-03
positive regulation of nervous system development (GO:0051962)	346	43	21.60	+	1.99	7.17E-05	3.96E-03
carbohydrate derivative biosynthetic process (GO:1901137)	491	61	30.65	+	1.99	2.17E-06	1.79E-04
regulation of muscle system process (GO:0090257)	258	32	16.10	+	1.99	5.91E-04	2.31E-02
glucose homeostasis (GO:0042593)	251	31	15.67	+	1.98	7.87E-04	2.93E-02
blood vessel morphogenesis (GO:0048514)	398	49	24.84	+	1.97	2.54E-05	1.58E-03
carbohydrate homeostasis (GO:0033500)	253	31	15.79	+	1.96	8.47E-04	3.12E-02
cellular response to hormone stimulus (GO:0032870)	605	74	37.76	+	1.96	3.06E-07	3.13E-05
regulation of actin filament organization (GO:0110053)	278	34	17.35	+	1.96	5.73E-04	2.26E-02
morphogenesis of an epithelium (GO:0002009)	524	64	32.71	+	1.96	1.91E-06	1.59E-04
Golgi vesicle transport (GO:0048193)	254	31	15.85	+	1.96	8.81E-04	3.20E-02
blood vessel development (GO:0001568)	501	61	31.27	+	1.95	4.20E-06	3.36E-04
neuron projection development (GO:0031175)	728	88	45.44	+	1.94	3.34E-08	4.81E-06
behavior (GO:0007610)	704	85	43.94	+	1.93	7.31E-08	9.15E-06
regulation of blood circulation (GO:1903522)	257	31	16.04	+	1.93	1.38E-03	4.59E-02

intracellular signal transduction (GO:0035556)	1397	168	87.20	+	1.93	1.14E-14	7.01E-12
positive regulation of cell development (GO:0010720)	367	44	22.91	+	1.92	1.18E-04	5.87E-03
kidney development (GO:0001822)	342	41	21.35	+	1.92	2.47E-04	1.11E-02
regulation of vasculature development (GO:1901342)	292	35	18.23	+	1.92	7.74E-04	2.90E-02
vasculature development (GO:0001944)	534	64	33.33	+	1.92	3.56E-06	2.88E-04
regulation of supramolecular fiber organization (GO:1902903)	378	45	23.59	+	1.91	1.41E-04	6.82E-03
response to carbohydrate (GO:0009743)	270	32	16.85	+	1.90	1.21E-03	4.12E-02
renal system development (GO:0072001)	355	42	22.16	+	1.90	2.19E-04	1.01E-02
cell morphogenesis involved in differentiation (GO:0000904)	575	68	35.89	+	1.89	3.36E-06	2.73E-04
regulation of cytoskeleton organization (GO:0051493)	534	63	33.33	+	1.89	8.12E-06	5.94E-04
cell morphogenesis (GO:0000902)	746	88	46.56	+	1.89	1.08E-07	1.31E-05
regulation of cellular response to stress (GO:0080135)	645	76	40.26	+	1.89	7.18E-07	6.57E-05
cellular response to growth factor stimulus (GO:0071363)	535	63	33.39	+	1.89	8.27E-06	5.99E-04
tube morphogenesis (GO:0035239)	697	82	43.50	+	1.88	3.45E-07	3.46E-05
urogenital system development (GO:0001655)	401	47	25.03	+	1.88	1.45E-04	6.97E-03
protein localization to membrane (GO:0072657)	444	52	27.71	+	1.88	6.20E-05	3.50E-03
regulation of cellular response to growth factor stimulus (GO:0090287)	282	33	17.60	+	1.87	1.48E-03	4.85E-02

response to growth factor (GO:0070848)	574	67	35.83	+	1.87	5.00E-06	3.89E-04
response to steroid hormone (GO:0048545)	446	52	27.84	+	1.87	6.44E-05	3.62E-03
regulation of actin filament-based process (GO:0032970)	413	48	25.78	+	1.86	1.22E-04	6.01E-03
negative regulation of phosphorylation (GO:0042326)	475	55	29.65	+	1.86	4.82E-05	2.83E-03
negative regulation of cell motility (GO:2000146)	304	35	18.97	+	1.84	1.47E-03	4.83E-02
regulation of cell growth (GO:0001558)	435	50	27.15	+	1.84	1.20E-04	5.91E-03
regulation of actin cytoskeleton organization (GO:0032956)	366	42	22.84	+	1.84	4.05E-04	1.69E-02
heart development (GO:0007507)	611	70	38.14	+	1.84	6.35E-06	4.78E-04
neuron development (GO:0048666)	895	102	55.86	+	1.83	3.99E-08	5.55E-06
regulation of transmembrane transport (GO:0034762)	599	68	37.39	+	1.82	1.13E-05	7.83E-04
positive regulation of cell differentiation (GO:0045597)	925	105	57.74	+	1.82	2.98E-08	4.33E-06
tube development (GO:0035295)	971	110	60.61	+	1.81	1.37E-08	2.13E-06
modulation of chemical synaptic transmission (GO:0050804)	530	60	33.08	+	1.81	3.77E-05	2.24E-03
regulation of trans-synaptic signaling (GO:0099177)	531	60	33.14	+	1.81	3.85E-05	2.28E-03
regulation of cellular component size (GO:0032535)	425	48	26.53	+	1.81	2.26E-04	1.03E-02
regulation of neuron projection development (GO:0010975)	549	62	34.27	+	1.81	2.52E-05	1.57E-03
cellular response to lipid (GO:0071396)	629	71	39.26	+	1.81	8.50E-06	6.11E-04

regulation of anatomical structure size (GO:0090066)	595	67	37.14	+	1.80	1.56E-05	1.05E-03
cellular response to peptide (GO:1901653)	382	43	23.84	+	1.80	5.24E-04	2.09E-02
cellular response to endogenous stimulus (GO:0071495)	1253	140	78.21	+	1.79	3.17E-10	6.97E-08
establishment of organelle localization (GO:0051656)	342	38	21.35	+	1.78	1.31E-03	4.39E-02
negative regulation of phosphorus metabolic process (GO:0010563)	585	65	36.51	+	1.78	2.88E-05	1.74E-03
negative regulation of phosphate metabolic process (GO:0045936)	585	65	36.51	+	1.78	2.88E-05	1.73E-03
forebrain development (GO:0030900)	487	54	30.40	+	1.78	1.34E-04	6.54E-03
tissue morphogenesis (GO:0048729)	634	70	39.57	+	1.77	1.94E-05	1.27E-03
regulation of anatomical structure morphogenesis (GO:0022603)	962	106	60.04	+	1.77	1.09E-07	1.31E-05
negative regulation of cellular macromolecule biosynthetic process (GO:2000113)	1444	159	90.13	+	1.76	3.94E-11	1.19E-08
negative regulation of macromolecule biosynthetic process (GO:0010558)	1491	164	93.06	+	1.76	2.39E-11	7.81E-09
regulation of epithelial cell proliferation (GO:0050678)	375	41	23.41	+	1.75	1.44E-03	4.74E-02
epithelial cell differentiation (GO:0030855)	559	61	34.89	+	1.75	8.51E-05	4.53E-03
negative regulation of protein phosphorylation (GO:0001933)	431	47	26.90	+	1.75	5.33E-04	2.12E-02
neuron differentiation (GO:0030182)	1119	122	69.84	+	1.75	1.79E-08	2.72E-06

positive regulation of cell migration (GO:0030335)	570	62	35.58	+	1.74	7.18E-05	3.95E-03
protein phosphorylation (GO:0006468)	779	84	48.62	+	1.73	5.69E-06	4.38E-04
cell population proliferation (GO:0008283)	548	59	34.20	+	1.72	1.51E-04	7.22E-03
positive regulation of developmental process (GO:0051094)	1419	152	88.57	+	1.72	7.25E-10	1.40E-07
response to hormone (GO:0009725)	1074	115	67.04	+	1.72	1.18E-07	1.38E-05
brain development (GO:0007420)	925	99	57.74	+	1.71	1.14E-06	1.01E-04
negative regulation of programmed cell death (GO:0043069)	964	103	60.17	+	1.71	6.63E-07	6.14E-05
cell junction organization (GO:0034330)	515	55	32.14	+	1.71	2.86E-04	1.27E-02
positive regulation of RNA metabolic process (GO:0051254)	1724	184	107.61	+	1.71	9.88E-12	4.06E-09
positive regulation of cell projection organization (GO:0031346)	441	47	27.53	+	1.71	8.67E-04	3.17E-02
head development (GO:0060322)	976	104	60.92	+	1.71	5.66E-07	5.43E-05
cellular response to organic cyclic compound (GO:0071407)	639	68	39.88	+	1.70	6.18E-05	3.50E-03
positive regulation of locomotion (GO:0040017)	611	65	38.14	+	1.70	8.76E-05	4.65E-03
anatomical structure formation involved in morphogenesis (GO:0048646)	931	99	58.11	+	1.70	1.25E-06	1.09E-04
negative regulation of cellular biosynthetic process (GO:0031327)	1554	165	97.00	+	1.70	2.30E-10	5.26E-08
negative regulation of biosynthetic process (GO:0009890)	1594	169	99.49	+	1.70	1.35E-10	3.43E-08

negative regulation of apoptotic process (GO:0043066)	944	100	58.92	+	1.70	1.44E-06	1.24E-04
positive regulation of cell motility (GO:2000147)	595	63	37.14	+	1.70	1.38E-04	6.69E-03
organelle localization (GO:0051640)	492	52	30.71	+	1.69	5.82E-04	2.29E-02
regulation of transferase activity (GO:0051338)	956	101	59.67	+	1.69	1.22E-06	1.07E-04
skeletal system development (GO:0001501)	502	53	31.33	+	1.69	4.81E-04	1.97E-02
chemical homeostasis (GO:0048878)	1203	127	75.09	+	1.69	5.14E-08	6.91E-06
regulation of cellular localization (GO:0060341)	910	96	56.80	+	1.69	2.65E-06	2.18E-04
cellular response to organonitrogen compound (GO:0071417)	693	73	43.25	+	1.69	4.38E-05	2.58E-03
regulation of kinase activity (GO:0043549)	855	90	53.37	+	1.69	5.45E-06	4.22E-04
embryonic morphogenesis (GO:0048598)	648	68	40.45	+	1.68	9.56E-05	4.96E-03
regulation of plasma membrane bounded cell projection organization (GO:0120035)	734	77	45.81	+	1.68	3.54E-05	2.12E-03
cell development (GO:0048468)	1784	187	111.35	+	1.68	2.79E-11	8.95E-09
nervous system development (GO:0007399)	2319	243	144.74	+	1.68	1.57E-14	9.33E-12
plasma membrane bounded cell projection organization (GO:0120036)	1117	117	69.72	+	1.68	2.76E-07	2.85E-05
establishment of localization in cell (GO:0051649)	1557	163	97.18	+	1.68	6.95E-10	1.36E-07
cell projection organization (GO:0030030)	1156	121	72.15	+	1.68	1.61E-07	1.81E-05
carbohydrate derivative metabolic process (GO:1901135)	870	91	54.30	+	1.68	6.39E-06	4.78E-04

positive regulation of cellular component movement (GO:0051272)	612	64	38.20	+	1.68	1.70E-04	7.99E-03
negative regulation of cell death (GO:0060548)	1082	113	67.53	+	1.67	5.01E-07	4.86E-05
negative regulation of transcription, DNA-templated (GO:0045892)	1246	130	77.77	+	1.67	6.10E-08	8.01E-06
positive regulation of nucleobase-containing compound metabolic process (GO:0045935)	1893	197	118.15	+	1.67	1.67E-11	5.94E-09
negative regulation of gene expression (GO:0010629)	1904	198	118.84	+	1.67	1.35E-11	5.15E-09
negative regulation of nucleic acid-templated transcription (GO:1903507)	1279	133	79.83	+	1.67	4.68E-08	6.35E-06
negative regulation of RNA biosynthetic process (GO:1902679)	1281	133	79.96	+	1.66	6.37E-08	8.30E-06
response to peptide (GO:1901652)	617	64	38.51	+	1.66	2.46E-04	1.11E-02
intracellular transport (GO:0046907)	1198	124	74.77	+	1.66	1.87E-07	2.03E-05
circulatory system development (GO:0072359)	948	98	59.17	+	1.66	4.20E-06	3.35E-04
actin filament-based process (GO:0030029)	571	59	35.64	+	1.66	3.98E-04	1.67E-02
gland development (GO:0048732)	610	63	38.07	+	1.65	3.15E-04	1.37E-02
neurogenesis (GO:0022008)	1540	159	96.12	+	1.65	2.98E-09	5.19E-07
epithelium development (GO:0060429)	1085	112	67.72	+	1.65	9.90E-07	8.86E-05
negative regulation of transcription by RNA polymerase II (GO:0000122)	901	93	56.24	+	1.65	8.89E-06	6.30E-04
phosphorylation (GO:0016310)	1128	116	70.41	+	1.65	6.23E-07	5.80E-05

negative regulation of RNA metabolic process (GO:0051253)	1381	142	86.20	+	1.65	3.66E-08	5.23E-06
cellular macromolecule localization (GO:0070727)	1470	151	91.75	+	1.65	1.34E-08	2.11E-06
regulation of cell projection organization (GO:0031344)	750	77	46.81	+	1.64	6.02E-05	3.44E-03
central nervous system development (GO:0007417)	1179	121	73.59	+	1.64	4.01E-07	3.94E-05
regulation of nervous system development (GO:0051960)	536	55	33.46	+	1.64	7.17E-04	2.71E-02
cellular protein localization (GO:0034613)	1462	150	91.25	+	1.64	1.29E-08	2.04E-06
generation of neurons (GO:0048699)	1387	142	86.57	+	1.64	3.97E-08	5.58E-06
response to endogenous stimulus (GO:0009719)	1672	171	104.36	+	1.64	1.63E-09	2.94E-07
cellular localization (GO:0051641)	2219	226	138.50	+	1.63	2.42E-12	1.05E-09
regulation of ion transmembrane transport (GO:0034765)	501	51	31.27	+	1.63	1.29E-03	4.33E-02
positive regulation of macromolecule biosynthetic process (GO:0010557)	1867	190	116.53	+	1.63	1.84E-10	4.52E-08
enzyme linked receptor protein signaling pathway (GO:0007167)	561	57	35.02	+	1.63	9.08E-04	3.29E-02
positive regulation of nucleic acid-templated transcription (GO:1903508)	1625	165	101.43	+	1.63	5.00E-09	8.44E-07
regulation of cell differentiation (GO:0045595)	1596	162	99.62	+	1.63	7.08E-09	1.17E-06
positive regulation of RNA biosynthetic process (GO:1902680)	1626	165	101.49	+	1.63	5.06E-09	8.45E-07

regulation of multicellular organismal development (GO:2000026)	1429	145	89.19	+	1.63	4.48E-08	6.18E-06
response to organonitrogen compound (GO:0010243)	1291	130	80.58	+	1.61	3.57E-07	3.55E-05
positive regulation of transcription by RNA polymerase II (GO:0045944)	1272	128	79.39	+	1.61	5.31E-07	5.12E-05
regulation of cell development (GO:0060284)	587	59	36.64	+	1.61	8.65E-04	3.17E-02
regulation of system process (GO:0044057)	627	63	39.14	+	1.61	5.19E-04	2.08E-02
positive regulation of cellular metabolic process (GO:0031325)	3375	339	210.66	+	1.61	4.15E-18	4.75E-15
intracellular protein transport (GO:0006886)	767	77	47.87	+	1.61	1.30E-04	6.38E-03
animal organ morphogenesis (GO:0009887)	1048	105	65.41	+	1.61	8.19E-06	5.96E-04
protein localization (GO:0008104)	1920	192	119.84	+	1.60	5.64E-10	1.14E-07
cellular chemical homeostasis (GO:0055082)	811	81	50.62	+	1.60	1.08E-04	5.47E-03
negative regulation of protein modification process (GO:0031400)	621	62	38.76	+	1.60	6.65E-04	2.54E-02
regulation of cell migration (GO:0030334)	952	95	59.42	+	1.60	2.73E-05	1.68E-03
positive regulation of transcription, DNA-templated (GO:0045893)	1566	156	97.74	+	1.60	4.60E-08	6.30E-06
regulation of protein kinase activity (GO:0045859)	743	74	46.38	+	1.60	2.54E-04	1.13E-02
negative regulation of developmental process (GO:0051093)	964	96	60.17	+	1.60	2.29E-05	1.47E-03

regulation of response to stress (GO:0080134)	1246	124	77.77	+	1.59	1.39E-06	1.21E-04
positive regulation of nitrogen compound metabolic process (GO:0051173)	3176	316	198.23	+	1.59	2.82E-16	2.06E-13
regulation of cellular component biogenesis (GO:0044087)	965	96	60.23	+	1.59	2.32E-05	1.48E-03
negative regulation of cellular protein metabolic process (GO:0032269)	1077	107	67.22	+	1.59	8.04E-06	5.91E-04
cellular protein catabolic process (GO:0044257)	604	60	37.70	+	1.59	1.02E-03	3.59E-02
positive regulation of cell population proliferation (GO:0008284)	1027	102	64.10	+	1.59	1.27E-05	8.66E-04
anatomical structure morphogenesis (GO:0009653)	2255	223	140.75	+	1.58	4.28E-11	1.25E-08
positive regulation of biosynthetic process (GO:0009891)	2025	200	126.39	+	1.58	5.77E-10	1.16E-07
regulation of programmed cell death (GO:0043067)	1510	149	94.25	+	1.58	1.68E-07	1.86E-05
positive regulation of transferase activity (GO:0051347)	639	63	39.88	+	1.58	7.93E-04	2.95E-02
negative regulation of protein metabolic process (GO:0051248)	1126	111	70.28	+	1.58	7.02E-06	5.20E-04
positive regulation of macromolecule metabolic process (GO:0010604)	3534	348	220.58	+	1.58	1.69E-17	1.51E-14
positive regulation of metabolic process (GO:0009893)	3820	376	238.43	+	1.58	4.71E-19	6.28E-16
cellular response to nitrogen compound (GO:1901699)	762	75	47.56	+	1.58	3.01E-04	1.32E-02

regulation of cell motility (GO:2000145)	1006	99	62.79	+	1.58	2.51E-05	1.58E-03
negative regulation of nucleobase-containing compound metabolic process (GO:0045934)	1494	147	93.25	+	1.58	2.03E-07	2.19E-05
positive regulation of cellular biosynthetic process (GO:0031328)	1982	195	123.71	+	1.58	1.33E-09	2.45E-07
regulation of apoptotic process (GO:0042981)	1485	146	92.69	+	1.58	2.47E-07	2.59E-05
proteolysis involved in cellular protein catabolic process (GO:0051603)	580	57	36.20	+	1.57	1.51E-03	4.90E-02
movement of cell or subcellular component (GO:0006928)	1417	139	88.44	+	1.57	5.90E-07	5.56E-05
cellular response to organic substance (GO:0071310)	2216	217	138.31	+	1.57	1.89E-10	4.59E-08
metal ion transport (GO:0030001)	613	60	38.26	+	1.57	1.47E-03	4.83E-02
regulation of phosphorylation (GO:0042325)	1606	157	100.24	+	1.57	1.17E-07	1.38E-05
regulation of cell death (GO:0010941)	1678	164	104.73	+	1.57	6.61E-08	8.54E-06
tissue development (GO:0009888)	1771	173	110.54	+	1.57	2.49E-08	3.73E-06
cell migration (GO:0016477)	860	84	53.68	+	1.56	1.66E-04	7.83E-03
cell death (GO:0008219)	789	77	49.25	+	1.56	2.87E-04	1.27E-02
regulation of protein modification process (GO:0031399)	1867	182	116.53	+	1.56	1.27E-08	2.03E-06
protein transport (GO:0015031)	1160	113	72.40	+	1.56	9.39E-06	6.60E-04
localization of cell (GO:0051674)	996	97	62.17	+	1.56	5.31E-05	3.08E-03
cell motility (GO:0048870)	996	97	62.17	+	1.56	5.31E-05	3.07E-03
nitrogen compound transport (GO:0071705)	1490	145	93.00	+	1.56	4.62E-07	4.51E-05

regulation of cellular component movement (GO:0051270)	1090	106	68.03	+	1.56	2.13E-05	1.38E-03
response to nitrogen compound (GO:1901698)	1379	134	86.07	+	1.56	1.79E-06	1.50E-04
cellular protein modification process (GO:0006464)	2553	248	159.35	+	1.56	1.33E-11	5.31E-09
protein modification process (GO:0036211)	2553	248	159.35	+	1.56	1.33E-11	5.18E-09
locomotion (GO:0040011)	1196	116	74.65	+	1.55	9.25E-06	6.53E-04
protein modification by small protein conjugation or removal (GO:0070647)	753	73	47.00	+	1.55	4.98E-04	2.01E-02
regulation of developmental process (GO:0050793)	2509	243	156.60	+	1.55	3.06E-11	9.61E-09
cellular response to oxygen-containing compound (GO:1901701)	1250	121	78.02	+	1.55	6.16E-06	4.68E-04
positive regulation of gene expression (GO:0010628)	2337	226	145.87	+	1.55	2.05E-10	4.83E-08
regulation of locomotion (GO:0040012)	1055	102	65.85	+	1.55	3.74E-05	2.24E-03
negative regulation of nitrogen compound metabolic process (GO:0051172)	2422	234	151.17	+	1.55	9.29E-11	2.48E-08
regulation of protein phosphorylation (GO:0001932)	1461	141	91.19	+	1.55	1.12E-06	9.95E-05
positive regulation of multicellular organismal process (GO:0051240)	1575	152	98.31	+	1.55	3.83E-07	3.79E-05
negative regulation of cellular metabolic process (GO:0031324)	2623	253	163.72	+	1.55	1.66E-11	6.03E-09
cellular macromolecule catabolic process (GO:0044265)	778	75	48.56	+	1.54	4.65E-04	1.91E-02

cellular homeostasis (GO:0019725)	915	88	57.11	+	1.54	1.52E-04	7.27E-03
regulation of protein metabolic process (GO:0051246)	2799	269	174.70	+	1.54	4.39E-12	1.85E-09
negative regulation of cell communication (GO:0010648)	1323	127	82.58	+	1.54	6.13E-06	4.68E-04
negative regulation of metabolic process (GO:0009892)	3044	292	190.00	+	1.54	4.30E-13	2.03E-10
negative regulation of signaling (GO:0023057)	1325	127	82.70	+	1.54	6.22E-06	4.70E-04
peptide transport (GO:0015833)	1191	114	74.34	+	1.53	2.02E-05	1.32E-03
establishment of protein localization (GO:0045184)	1244	119	77.65	+	1.53	1.33E-05	8.98E-04
positive regulation of cell death (GO:0010942)	680	65	42.44	+	1.53	1.46E-03	4.82E-02
regulation of phosphate metabolic process (GO:0019220)	1800	172	112.35	+	1.53	9.91E-08	1.22E-05
regulation of phosphorus metabolic process (GO:0051174)	1801	172	112.41	+	1.53	1.27E-07	1.46E-05
regulation of organelle organization (GO:0033043)	1184	113	73.90	+	1.53	2.51E-05	1.57E-03
peptidyl-amino acid modification (GO:0018193)	849	81	52.99	+	1.53	3.58E-04	1.52E-02
phosphorus metabolic process (GO:0006793)	1909	182	119.15	+	1.53	5.34E-08	7.12E-06
macromolecule localization (GO:0033036)	2256	215	140.81	+	1.53	2.51E-09	4.47E-07
regulation of cellular component organization (GO:0051128)	2435	232	151.98	+	1.53	4.16E-10	8.66E-08

negative regulation of signal transduction (GO:0009968)	1198	114	74.77	+	1.52	2.78E-05	1.69E-03
regulation of signaling (GO:0023051)	3237	308	202.04	+	1.52	2.14E-13	1.11E-10
response to lipid (GO:0033993)	1209	115	75.46	+	1.52	2.32E-05	1.48E-03
regulation of cellular protein metabolic process (GO:0032268)	2651	252	165.47	+	1.52	6.57E-11	1.78E-08
regulation of ion transport (GO:0043269)	768	73	47.94	+	1.52	9.56E-04	3.41E-02
amide transport (GO:0042886)	1221	116	76.21	+	1.52	2.51E-05	1.58E-03
regulation of cell population proliferation (GO:0042127)	1737	165	108.42	+	1.52	3.15E-07	3.17E-05
regulation of protein localization (GO:0032880)	1011	96	63.10	+	1.52	1.35E-04	6.55E-03
regulation of cell communication (GO:0010646)	3225	306	201.29	+	1.52	3.52E-13	1.71E-10
phosphate-containing compound metabolic process (GO:0006796)	1888	179	117.84	+	1.52	1.04E-07	1.27E-05
cell differentiation (GO:0030154)	3397	322	212.03	+	1.52	6.28E-14	3.59E-11
response to organic cyclic compound (GO:0014070)	1311	124	81.83	+	1.52	1.28E-05	8.69E-04
negative regulation of macromolecule metabolic process (GO:0010605)	2794	264	174.39	+	1.51	4.15E-11	1.23E-08
regulation of intracellular signal transduction (GO:1902531)	1594	150	99.49	+	1.51	1.73E-06	1.46E-04
cellular developmental process (GO:0048869)	3447	324	215.15	+	1.51	1.53E-13	8.19E-11
regulation of signal transduction (GO:0009966)	2793	262	174.33	+	1.50	9.51E-11	2.50E-08
embryo development (GO:0009790)	1206	113	75.27	+	1.50	4.99E-05	2.91E-03

positive regulation of signal transduction (GO:0009967)	1527	143	95.31	+	1.50	4.06E-06	3.27E-04
positive regulation of cellular component organization (GO:0051130)	1207	113	75.34	+	1.50	5.04E-05	2.93E-03
macromolecule catabolic process (GO:0009057)	876	82	54.68	+	1.50	5.68E-04	2.25E-02
cell cycle process (GO:0022402)	802	75	50.06	+	1.50	1.21E-03	4.11E-02
macromolecule modification (GO:0043412)	2743	256	171.21	+	1.50	2.70E-10	6.00E-08
ion homeostasis (GO:0050801)	827	77	51.62	+	1.49	1.11E-03	3.88E-02
vesicle-mediated transport (GO:0016192)	1237	115	77.21	+	1.49	6.07E-05	3.46E-03
organic substance transport (GO:0071702)	1836	170	114.60	+	1.48	8.02E-07	7.22E-05
negative regulation of cellular process (GO:0048523)	4852	449	302.84	+	1.48	1.34E-18	1.65E-15
cellular catabolic process (GO:0044248)	1542	142	96.25	+	1.48	1.20E-05	8.27E-04
negative regulation of biological process (GO:0048519)	5297	487	330.62	+	1.47	4.99E-20	8.00E-17
organonitrogen compound catabolic process (GO:1901565)	970	89	60.54	+	1.47	6.23E-04	2.42E-02
regulation of localization (GO:0032879)	2945	270	183.82	+	1.47	4.05E-10	8.53E-08
negative regulation of response to stimulus (GO:0048585)	1586	145	98.99	+	1.46	1.22E-05	8.36E-04
positive regulation of phosphorus metabolic process (GO:0010562)	1172	107	73.15	+	1.46	2.29E-04	1.04E-02
positive regulation of phosphate metabolic process (GO:0045937)	1172	107	73.15	+	1.46	2.29E-04	1.03E-02
regulation of cell cycle (GO:0051726)	986	90	61.54	+	1.46	6.84E-04	2.60E-02

positive regulation of signaling (GO:0023056)	1742	159	108.73	+	1.46	4.31E-06	3.41E-04
positive regulation of intracellular signal transduction (GO:1902533)	997	91	62.23	+	1.46	7.21E-04	2.72E-02
positive regulation of cellular process (GO:0048522)	5738	523	358.15	+	1.46	3.69E-21	7.38E-18
cell cycle (GO:0007049)	878	80	54.80	+	1.46	1.53E-03	4.95E-02
system development (GO:0048731)	4474	407	279.25	+	1.46	2.33E-15	1.62E-12
positive regulation of protein metabolic process (GO:0051247)	1694	154	105.73	+	1.46	8.52E-06	6.09E-04
negative regulation of molecular function (GO:0044092)	1167	106	72.84	+	1.46	2.82E-04	1.25E-02
animal organ development (GO:0048513)	3535	320	220.64	+	1.45	1.96E-11	6.67E-09
positive regulation of cell communication (GO:0010647)	1736	157	108.35	+	1.45	8.48E-06	6.12E-04
positive regulation of protein modification process (GO:0031401)	1250	113	78.02	+	1.45	2.20E-04	1.01E-02
homeostatic process (GO:0042592)	1743	157	108.79	+	1.44	1.11E-05	7.68E-04
regulation of multicellular organismal process (GO:0051239)	2767	249	172.71	+	1.44	1.14E-08	1.84E-06
response to abiotic stimulus (GO:0009628)	1390	125	86.76	+	1.44	1.15E-04	5.74E-03
regulation of macromolecule biosynthetic process (GO:0010556)	3654	328	228.07	+	1.44	2.19E-11	7.31E-09
positive regulation of biological process (GO:0048518)	6198	556	386.86	+	1.44	2.03E-21	4.64E-18
regulation of cellular macromolecule biosynthetic process (GO:2000112)	3569	320	222.76	+	1.44	5.07E-11	1.43E-08

regulation of biological quality (GO:0065008)	4007	359	250.10	+	1.44	1.99E-12	8.86E-10
cellular response to chemical stimulus (GO:0070887)	2803	251	174.95	+	1.43	1.70E-08	2.63E-06
regulation of RNA metabolic process (GO:0051252)	3438	307	214.59	+	1.43	2.50E-10	5.64E-08
anatomical structure development (GO:0048856)	5286	472	329.93	+	1.43	6.47E-17	4.93E-14
multicellular organism development (GO:0007275)	4915	437	306.78	+	1.42	4.81E-15	3.08E-12
regulation of response to stimulus (GO:0048583)	3773	335	235.50	+	1.42	4.81E-11	1.38E-08
developmental process (GO:0032502)	5713	507	356.58	+	1.42	4.83E-18	5.16E-15
regulation of catalytic activity (GO:0050790)	2232	198	139.31	+	1.42	1.55E-06	1.32E-04
regulation of molecular function (GO:0065009)	2922	259	182.38	+	1.42	1.90E-08	2.88E-06
response to organic substance (GO:0010033)	3263	289	203.66	+	1.42	2.57E-09	4.52E-07
regulation of nitrogen compound metabolic process (GO:0051171)	5556	491	346.79	+	1.42	5.34E-17	4.27E-14
organic substance catabolic process (GO:1901575)	1539	136	96.06	+	1.42	9.90E-05	5.10E-03
regulation of cellular metabolic process (GO:0031323)	5917	521	369.32	+	1.41	4.84E-18	4.85E-15
regulation of primary metabolic process (GO:0080090)	5716	503	356.77	+	1.41	4.34E-17	3.66E-14

regulation of transcription by RNA polymerase II (GO:0006357)	2342	206	146.18	+	1.41	1.63E-06	1.38E-04
positive regulation of phosphorylation (GO:0042327)	1092	96	68.16	+	1.41	1.49E-03	4.86E-02
positive regulation of catalytic activity (GO:0043085)	1343	118	83.83	+	1.41	4.36E-04	1.80E-02
positive regulation of cellular protein metabolic process (GO:0032270)	1618	142	100.99	+	1.41	9.20E-05	4.82E-03
regulation of nucleobase-containing compound metabolic process (GO:0019219)	3685	323	230.00	+	1.40	4.73E-10	9.70E-08
regulation of cellular biosynthetic process (GO:0031326)	3826	335	238.81	+	1.40	2.08E-10	4.82E-08
positive regulation of molecular function (GO:0044093)	1714	150	106.98	+	1.40	7.26E-05	3.97E-03
regulation of macromolecule metabolic process (GO:0060255)	6088	532	379.99	+	1.40	7.01E-18	6.61E-15
regulation of biosynthetic process (GO:0009889)	3904	341	243.67	+	1.40	1.77E-10	4.43E-08
regulation of metabolic process (GO:0019222)	6552	572	408.95	+	1.40	1.43E-19	2.09E-16
catabolic process (GO:0009056)	1802	156	112.47	+	1.39	8.51E-05	4.54E-03
localization (GO:0051179)	4958	428	309.46	+	1.38	9.12E-13	4.17E-10
regulation of gene expression (GO:0010468)	4470	385	279.00	+	1.38	3.64E-11	1.12E-08
response to oxygen-containing compound (GO:1901700)	2021	174	126.14	+	1.38	3.88E-05	2.29E-03

regulation of RNA biosynthetic process (GO:2001141)	3172	273	197.98	+	1.38	1.04E-07	1.26E-05
regulation of transcription, DNA-templated (GO:0006355)	3118	268	194.61	+	1.38	1.62E-07	1.82E-05
regulation of nucleic acid-templated transcription (GO:1903506)	3167	272	197.67	+	1.38	1.27E-07	1.47E-05
positive regulation of response to stimulus (GO:0048584)	2168	186	135.32	+	1.37	2.44E-05	1.54E-03
regulation of transport (GO:0051049)	1966	168	122.71	+	1.37	7.19E-05	3.94E-03
cellular protein metabolic process (GO:0044267)	3289	279	205.29	+	1.36	2.41E-07	2.54E-05
transport (GO:0006810)	3611	305	225.39	+	1.35	7.22E-08	9.11E-06
cellular component organization (GO:0016043)	5159	432	322.01	+	1.34	5.52E-11	1.52E-08
establishment of localization (GO:0051234)	3756	311	234.44	+	1.33	3.06E-07	3.11E-05
organelle organization (GO:0006996)	3243	268	202.42	+	1.32	3.34E-06	2.73E-04
cellular macromolecule metabolic process (GO:0044260)	4362	359	272.26	+	1.32	3.87E-08	5.49E-06
protein metabolic process (GO:0019538)	3929	321	245.23	+	1.31	5.89E-07	5.58E-05
cellular component organization or biogenesis (GO:0071840)	5382	438	335.92	+	1.30	1.51E-09	2.75E-07
organonitrogen compound metabolic process (GO:1901564)	4859	392	303.28	+	1.29	5.89E-08	7.80E-06
cellular metabolic process (GO:0044237)	6893	552	430.24	+	1.28	1.71E-11	5.97E-09
biosynthetic process (GO:0009058)	2315	185	144.49	+	1.28	8.46E-04	3.12E-02
response to stress (GO:0006950)	3335	265	208.16	+	1.27	5.87E-05	3.37E-03

organic substance biosynthetic process (GO:1901576)	2257	179	140.87	+	1.27	1.42E-03	4.69E-02
primary metabolic process (GO:0044238)	6804	535	424.68	+	1.26	9.47E-10	1.78E-07
organic substance metabolic process (GO:0071704)	7304	572	455.89	+	1.25	1.98E-10	4.73E-08
macromolecule metabolic process (GO:0043170)	5649	442	352.59	+	1.25	1.79E-07	1.98E-05
nitrogen compound metabolic process (GO:0006807)	6266	484	391.10	+	1.24	1.34E-07	1.53E-05
metabolic process (GO:0008152)	7856	604	490.34	+	1.23	8.04E-10	1.53E-07
regulation of biological process (GO:0050789)	12029	867	750.81	+	1.15	5.85E-10	1.16E-07
regulation of cellular process (GO:0050794)	11578	834	722.66	+	1.15	3.37E-09	5.74E-07
biological regulation (GO:0065007)	12713	912	793.50	+	1.15	1.31E-10	3.38E-08
multicellular organismal process (GO:0032501)	7487	532	467.31	+	1.14	4.13E-04	1.72E-02
cellular process (GO:0009987)	15509	1089	968.02	+	1.12	2.26E-13	1.13E-10
biological_process (GO:0008150)	18114	1214	1130.61	+	1.07	3.42E-10	7.31E-08
nervous system process (GO:0050877)	2200	95	137.32	-	.69	1.21E-04	5.98E-03
Unclassified (UNCLASSIFIED)	3563	139	222.39	-	.63	3.42E-10	7.40E-08
G protein-coupled receptor signaling pathway (GO:0007186)	2088	56	130.33	-	.43	1.45E-13	8.01E-11
sensory perception (GO:0007600)	1721	37	107.42	-	.34	4.27E-15	2.85E-12
ribosome biogenesis (GO:0042254)	362	5	22.59	-	.22	2.60E-05	1.61E-03
humoral immune response (GO:0006959)	304	4	18.97	-	.21	1.18E-04	5.88E-03
detection of stimulus (GO:0051606)	1427	17	89.07	-	.19	1.99E-20	3.55E-17
rRNA processing (GO:0006364)	230	2	14.36	-	.14	1.93E-04	9.03E-03

rRNA metabolic process (GO:0016072)	241	2	15.04	-	.13	9.12E-05	4.79E-03
detection of stimulus involved in sensory perception (GO:0050906)	1324	9	82.64	-	.11	1.35E-24	3.60E-21
detection of chemical stimulus (GO:0009593)	1275	6	79.58	-	.08	1.74E-26	5.57E-23
sensory perception of chemical stimulus (GO:0007606)	1315	5	82.08	-	.06	1.33E-28	5.33E-25
sensory perception of smell (GO:0007608)	1230	2	76.77	-	.03	3.75E-30	2.00E-26
detection of chemical stimulus involved in sensory perception (GO:0050907)	1241	2	77.46	-	.03	1.54E-30	1.23E-26
detection of chemical stimulus involved in sensory perception of smell (GO:0050911)	1184	1	73.90	-	.01	1.22E-30	1.95E-26

Supplementary table S7. List of differentially expressed genes at 2d and the associated over and under represented Biological Processes. Raw Panther results for all DEGs

Analysis Type:	PANTHER Overrepresentation Test (Released 20200728)
Annotation Version and Release Date:	GO Ontology database DOI: 10.5281/zenodo.4081749 Released 2020-10-09
Analyzed List:	Client Text Box Input (Rattus norvegicus)
Reference List:	Rattus norvegicus (all genes in database)
Test Type:	FISHER
Correction:	FDR

GO biological process complete	Rattus norvegicus - REFLIST (21677)	Client Text Box Input (587)	Client Text Box Input (expected)	Client Text Box Input (over/under)	Client Text Box Input (fold Enrichment)	Client Text Box Input (raw P-value)	Client Text Box Input (FDR)
regulation of glucocorticoid mediated signaling pathway (GO:1900169)	3	3	.08	+	36.93	3.44E-04	4.79E-02
positive regulation of cyclic-nucleotide phosphodiesterase activity (GO:0051343)	5	4	.14	+	29.54	5.42E-05	1.50E-02
positive regulation of ryanodine-sensitive calcium-release channel activity (GO:0060316)	9	4	.24	±	16.41	2.83E-04	4.49E-02
regulation of cyclic-nucleotide phosphodiesterase activity (GO:0051342)	9	4	.24	+	16.41	2.83E-04	4.44E-02

regulation of release of sequestered calcium ion into cytosol by sarcoplasmic reticulum (GO:0010880)	28	6	.76	+	7.91	2.35E-04	4.09E-02
negative regulation of transporter activity (GO:0032410)	87	10	2.36	+	4.24	2.42E-04	4.07E-02
negative regulation of transmembrane transport (GO:0034763)	138	13	3.74	+	3.48	1.89E-04	3.60E-02
cellular response to steroid hormone stimulus (GO:0071383)	192	16	5.20	+	3.08	1.36E-04	2.95E-02
adult behavior (GO:0030534)	182	15	4.93	+	3.04	2.44E-04	4.07E-02
establishment of protein localization to membrane (GO:0090150)	186	15	5.04	+	2.98	3.03E-04	4.45E-02
protein localization to membrane (GO:0072657)	444	31	12.02	+	2.58	3.95E-06	1.98E-03
kidney development (GO:0001822)	342	23	9.26	+	2.48	1.70E-04	3.40E-02
cell division (GO:0051301)	361	24	9.78	+	2.46	1.29E-04	2.92E-02
axon development (GO:0061564)	399	26	10.80	+	2.41	8.01E-05	2.00E-02
renal system development (GO:0072001)	355	23	9.61	+	2.39	2.32E-04	4.17E-02
cell morphogenesis involved in neuron differentiation (GO:0048667)	447	27	12.10	+	2.23	2.05E-04	3.81E-02
posttranscriptional regulation of gene expression (GO:0010608)	459	27	12.43	+	2.17	2.76E-04	4.51E-02
cellular response to hormone stimulus (GO:0032870)	605	33	16.38	+	2.01	2.34E-04	4.13E-02
neuron development (GO:0048666)	895	46	24.24	+	1.90	6.29E-05	1.71E-02
cation homeostasis (GO:0055080)	742	38	20.09	+	1.89	3.48E-04	4.80E-02
negative regulation of transcription by RNA polymerase II (GO:0000122)	901	46	24.40	+	1.89	6.86E-05	1.80E-02

intracellular protein transport (GO:0006886)	767	39	20.77	+	1.88	2.87E-04	4.30E-02
response to organonitrogen compound (GO:0010243)	1291	65	34.96	+	1.86	2.86E-06	1.48E-03
regulation of kinase activity (GO:0043549)	855	43	23.15	+	1.86	1.69E-04	3.43E-02
ion homeostasis (GO:0050801)	827	41	22.39	+	1.83	3.19E-04	4.56E-02
regulation of catabolic process (GO:0009894)	873	43	23.64	+	1.82	2.92E-04	4.32E-02
nervous system development (GO:0007399)	2319	114	62.80	+	1.82	1.01E-09	1.47E-06
brain development (GO:0007420)	925	45	25.05	+	1.80	2.86E-04	4.45E-02
positive regulation of cell differentiation (GO:0045597)	925	45	25.05	+	1.80	2.86E-04	4.41E-02
regulation of transferase activity (GO:0051338)	956	46	25.89	+	1.78	2.54E-04	4.20E-02
response to nitrogen compound (GO:1901698)	1379	66	37.34	+	1.77	1.25E-05	4.46E-03
response to hormone (GO:0009725)	1074	51	29.08	+	1.75	1.71E-04	3.34E-02
cellular protein localization (GO:0034613)	1462	69	39.59	+	1.74	1.42E-05	4.95E-03
cellular macromolecule localization (GO:0070727)	1470	69	39.81	+	1.73	1.52E-05	5.17E-03
chemical homeostasis (GO:0048878)	1203	56	32.58	+	1.72	1.27E-04	2.91E-02
intracellular signal transduction (GO:0035556)	1397	65	37.83	+	1.72	4.30E-05	1.23E-02
cell development (GO:0048468)	1784	83	48.31	+	1.72	2.38E-06	1.31E-03
neuron differentiation (GO:0030182)	1119	52	30.30	+	1.72	2.34E-04	4.16E-02
central nervous system development (GO:0007417)	1179	54	31.93	+	1.69	3.21E-04	4.55E-02

regulation of cell differentiation (GO:0045595)	1596	72	43.22	+	1.67	3.23E-05	9.77E-03
positive regulation of developmental process (GO:0051094)	1419	64	38.43	+	1.67	1.02E-04	2.39E-02
negative regulation of macromolecule biosynthetic process (GO:0010558)	1491	67	40.38	+	1.66	7.43E-05	1.89E-02
neurogenesis (GO:0022008)	1540	69	41.70	+	1.65	6.65E-05	1.78E-02
generation of neurons (GO:0048699)	1387	62	37.56	+	1.65	1.71E-04	3.31E-02
tissue development (GO:0009888)	1771	79	47.96	+	1.65	1.93E-05	6.32E-03
establishment of localization in cell (GO:0051649)	1557	69	42.16	+	1.64	1.01E-04	2.41E-02
response to organic cyclic compound (GO:0014070)	1311	58	35.50	+	1.63	3.37E-04	4.73E-02
regulation of developmental process (GO:0050793)	2509	110	67.94	+	1.62	6.69E-07	4.46E-04
negative regulation of cellular biosynthetic process (GO:0031327)	1554	68	42.08	+	1.62	1.42E-04	2.99E-02
negative regulation of cellular macromolecule biosynthetic process (GO:2000113)	1444	63	39.10	+	1.61	3.11E-04	4.49E-02
nitrogen compound transport (GO:0071705)	1490	65	40.35	+	1.61	2.09E-04	3.84E-02
cell differentiation (GO:0030154)	3397	148	91.99	+	1.61	4.29E-09	5.73E-06
negative regulation of biosynthetic process (GO:0009890)	1594	69	43.16	+	1.60	1.70E-04	3.37E-02
protein localization (GO:0008104)	1920	83	51.99	+	1.60	3.77E-05	1.10E-02
system development (GO:0048731)	4474	193	121.15	+	1.59	1.11E-11	2.22E-08

cellular developmental process (GO:0048869)	3447	148	93.34	+	1.59	1.07E-08	1.08E-05
anatomical structure morphogenesis (GO:0009653)	2255	96	61.06	+	1.57	1.59E-05	5.31E-03
negative regulation of gene expression (GO:0010629)	1904	81	51.56	+	1.57	9.05E-05	2.20E-02
cellular localization (GO:0051641)	2219	94	60.09	+	1.56	1.95E-05	6.24E-03
regulation of cellular component organization (GO:0051128)	2435	103	65.94	+	1.56	9.10E-06	3.55E-03
anatomical structure development (GO:0048856)	5286	222	143.14	+	1.55	1.34E-12	4.29E-09
phosphorus metabolic process (GO:0006793)	1909	80	51.69	+	1.55	1.30E-04	2.88E-02
regulation of protein modification process (GO:0031399)	1867	78	50.56	+	1.54	1.96E-04	3.70E-02
developmental process (GO:0032502)	5713	238	154.70	+	1.54	2.09E-13	8.39E-10
multicellular organism development (GO:0007275)	4915	204	133.10	+	1.53	5.50E-11	9.78E-08
phosphate-containing compound metabolic process (GO:0006796)	1888	78	51.13	+	1.53	2.82E-04	4.56E-02
positive regulation of macromolecule metabolic process (GO:0010604)	3534	146	95.70	+	1.53	1.86E-07	1.42E-04
animal organ development (GO:0048513)	3535	144	95.73	+	1.50	4.73E-07	3.30E-04
positive regulation of metabolic process (GO:0009893)	3820	155	103.44	+	1.50	1.82E-07	1.46E-04
negative regulation of metabolic process (GO:0009892)	3044	123	82.43	+	1.49	6.55E-06	2.76E-03

positive regulation of nitrogen compound metabolic process (GO:0051173)	3176	128	86.00	+	1.49	5.26E-06	2.28E-03
positive regulation of cellular metabolic process (GO:0031325)	3375	136	91.39	+	1.49	2.24E-06	1.28E-03
macromolecule localization (GO:0033036)	2256	90	61.09	+	1.47	2.87E-04	4.37E-02
regulation of signaling (GO:0023051)	3237	129	87.66	+	1.47	8.23E-06	3.29E-03
regulation of cell communication (GO:0010646)	3225	128	87.33	+	1.47	1.05E-05	3.92E-03
positive regulation of gene expression (GO:0010628)	2337	92	63.28	+	1.45	3.58E-04	4.90E-02
negative regulation of cellular metabolic process (GO:0031324)	2623	103	71.03	+	1.45	1.57E-04	3.22E-02
negative regulation of biological process (GO:0048519)	5297	207	143.44	+	1.44	8.72E-09	9.32E-06
regulation of signal transduction (GO:0009966)	2793	109	75.63	+	1.44	1.12E-04	2.60E-02
negative regulation of cellular process (GO:0048523)	4852	188	131.39	+	1.43	1.33E-07	1.12E-04
cellular response to chemical stimulus (GO:0070887)	2803	108	75.90	+	1.42	2.38E-04	4.05E-02
positive regulation of cellular process (GO:0048522)	5738	221	155.38	+	1.42	5.26E-09	6.01E-06
regulation of biological quality (GO:0065008)	4007	154	108.51	+	1.42	4.78E-06	2.25E-03
regulation of response to stimulus (GO:0048583)	3773	145	102.17	+	1.42	1.05E-05	4.01E-03
regulation of multicellular organismal process (GO:0051239)	2767	106	74.93	+	1.41	2.87E-04	4.33E-02

regulation of protein metabolic process (GO:0051246)	2799	107	75.80	+	1.41	3.03E-04	4.42E-02
positive regulation of biological process (GO:0048518)	6198	235	167.84	+	1.40	5.09E-09	6.27E-06
response to stress (GO:0006950)	3335	126	90.31	+	1.40	1.31E-04	2.88E-02
localization (GO:0051179)	4958	187	134.26	+	1.39	8.30E-07	5.11E-04
response to organic substance (GO:0010033)	3263	122	88.36	+	1.38	2.35E-04	4.04E-02
organelle organization (GO:0006996)	3243	121	87.82	+	1.38	2.83E-04	4.53E-02
cellular component organization (GO:0016043)	5159	188	139.70	+	1.35	7.89E-06	3.24E-03
regulation of metabolic process (GO:0019222)	6552	238	177.42	+	1.34	1.99E-07	1.45E-04
regulation of primary metabolic process (GO:0080090)	5716	206	154.79	+	1.33	4.17E-06	2.03E-03
regulation of cellular metabolic process (GO:0031323)	5917	213	160.23	+	1.33	2.68E-06	1.43E-03
regulation of macromolecule metabolic process (GO:0060255)	6088	219	164.86	+	1.33	2.02E-06	1.20E-03
regulation of nitrogen compound metabolic process (GO:0051171)	5556	199	150.45	+	1.32	1.06E-05	3.86E-03
cellular component organization or biogenesis (GO:0071840)	5382	189	145.74	+	1.30	7.27E-05	1.88E-02
cellular metabolic process (GO:0044237)	6893	234	186.66	+	1.25	5.25E-05	1.48E-02
primary metabolic process (GO:0044238)	6804	230	184.25	+	1.25	8.77E-05	2.16E-02
organic substance metabolic process (GO:0071704)	7304	243	197.79	+	1.23	1.40E-04	3.00E-02
metabolic process (GO:0008152)	7856	258	212.74	+	1.21	1.53E-04	3.19E-02

regulation of biological process (GO:0050789)	12029	376	325.74	+	1.15	3.56E-05	1.06E-02
biological regulation (GO:0065007)	12713	395	344.26	+	1.15	2.47E-05	7.62E-03
regulation of cellular process (GO:0050794)	11578	359	313.53	+	1.15	2.18E-04	3.97E-02
cellular process (GO:0009987)	15509	479	419.97	+	1.14	3.56E-08	3.35E-05
biological_process (GO:0008150)	18114	530	490.52	+	1.08	5.21E-06	2.38E-03
Unclassified (UNCLASSIFIED)	3563	57	96.48	-	.59	5.21E-06	2.32E-03
G protein-coupled receptor signaling pathway (GO:0007186)	2088	28	56.54	-	.50	2.30E-05	7.23E-03
sensory perception (GO:0007600)	1721	17	46.60	-	.36	6.93E-07	4.44E-04
detection of stimulus (GO:0051606)	1427	10	38.64	-	.26	5.37E-08	4.78E-05
detection of chemical stimulus (GO:0009593)	1275	4	34.53	-	.12	6.83E-11	1.09E-07
sensory perception of chemical stimulus (GO:0007606)	1315	3	35.61	-	.08	4.06E-12	9.28E-09
detection of stimulus involved in sensory perception (GO:0050906)	1324	3	35.85	-	.08	2.60E-12	6.93E-09
sensory perception of smell (GO:0007608)	1230	1	33.31	-	.03	1.41E-13	7.52E-10
detection of chemical stimulus involved in sensory perception (GO:0050907)	1241	1	33.61	-	.03	9.21E-14	7.38E-10
detection of chemical stimulus involved in sensory perception of smell (GO:0050911)	1184	0	32.06	-	< 0.01	1.59E-14	2.55E-10

Supplementary table S8. List of differentially expressed genes at 8d and the associated over and under represented Biological Processes. Raw Panther results for all DEGs

Analysis Type:	PANTHER Overrepresentation Test (Released 20200728)
Annotation Version and Release Date:	GO Ontology database DOI: 10.5281/zenodo.4081749 Released 2020-10-09
Analyzed List:	Client Text Box Input (Rattus norvegicus)
Reference List:	Rattus norvegicus (all genes in database)
Test Type:	FISHER
Correction:	FDR

**GO biological process complete**	**Rattus norvegicus - REFLIST (21677)**	**Client Text Box Input (815)**	**Client Text Box Input (expected)**	**Client Text Box Input (over/under)**	**Client Text Box Input (fold Enrichment)**	**Client Text Box Input (raw P-value)**	**Client Text Box Input (FDR)**
positive regulation of cyclic-nucleotide phosphodiesterase activity (GO:0051343)	5	4	.19	+	21.28	1.86E-04	2.84E-02
regulation of store-operated calcium channel activity (GO:1901339)	14	6	.53	+	11.40	5.57E-05	1.05E-02
negative regulation of vascular permeability (GO:0043116)	20	6	.75	+	7.98	2.75E-04	3.79E-02
endosome organization (GO:0007032)	50	10	1.88	+	5.32	5.39E-05	1.03E-02

response to vitamin A (GO:0033189)	41	8	1.54	+	5.19	3.47E-04	4.52E-02
developmental cell growth (GO:0048588)	104	13	3.91	+	3.32	3.25E-04	4.30E-02
dendrite development (GO:0016358)	138	17	5.19	+	3.28	4.92E-05	9.61E-03
negative regulation of translation (GO:0017148)	134	15	5.04	+	2.98	3.46E-04	4.54E-02
regulation of neuron differentiation (GO:0045664)	235	26	8.84	+	2.94	3.41E-06	1.05E-03
positive regulation of neuron projection development (GO:0010976)	219	21	8.23	+	2.55	1.87E-04	2.83E-02
positive regulation of cell projection organization (GO:0031346)	441	33	16.58	+	1.99	4.06E-04	4.93E-02
positive regulation of cell differentiation (GO:0045597)	925	66	34.78	+	1.90	2.01E-06	7.01E-04
regulation of cytoskeleton organization (GO:0051493)	534	38	20.08	+	1.89	3.71E-04	4.58E-02
regulation of anatomical structure size (GO:0090066)	595	42	22.37	+	1.88	2.13E-04	3.08E-02
protein catabolic process (GO:0030163)	663	45	24.93	+	1.81	2.99E-04	4.03E-02
cellular protein localization (GO:0034613)	1462	98	54.97	+	1.78	1.03E-07	7.20E-05
cellular macromolecule localization (GO:0070727)	1470	98	55.27	+	1.77	1.13E-07	6.93E-05

negative regulation of transcription by RNA polymerase II (GO:0000122)	901	60	33.88	+	1.77	4.24E-05	8.94E-03
regulation of anatomical structure morphogenesis (GO:0022603)	962	64	36.17	+	1.77	2.37E-05	5.67E-03
vesicle-mediated transport (GO:0016192)	1237	82	46.51	+	1.76	1.99E-06	7.10E-04
intracellular transport (GO:0046907)	1198	79	45.04	+	1.75	3.12E-06	9.81E-04
intracellular protein transport (GO:0006886)	767	50	28.84	+	1.73	3.68E-04	4.57E-02
regulation of cell differentiation (GO:0045595)	1596	104	60.01	+	1.73	1.13E-07	6.73E-05
cellular localization (GO:0051641)	2219	144	83.43	+	1.73	3.29E-10	5.85E-07
positive regulation of developmental process (GO:0051094)	1419	92	53.35	+	1.72	1.01E-06	4.15E-04
protein localization (GO:0008104)	1920	123	72.19	+	1.70	1.75E-08	1.65E-05
macromolecule catabolic process (GO:0009057)	876	56	32.94	+	1.70	2.18E-04	3.12E-02
regulation of cellular localization (GO:0060341)	910	58	34.21	+	1.70	1.97E-04	2.89E-02
regulation of multicellular organismal development (GO:2000026)	1429	90	53.73	+	1.68	4.51E-06	1.34E-03

establishment of localization in cell (GO:0051649)	1557	97	58.54	+	1.66	2.82E-06	9.23E-04
positive regulation of cellular component organization (GO:0051130)	1207	75	45.38	+	1.65	4.08E-05	8.95E-03
macromolecule localization (GO:0033036)	2256	140	84.82	+	1.65	1.12E-08	1.12E-05
cell development (GO:0048468)	1784	110	67.07	+	1.64	6.53E-07	2.83E-04
localization of cell (GO:0051674)	996	61	37.45	+	1.63	3.63E-04	4.58E-02
cell motility (GO:0048870)	996	61	37.45	+	1.63	3.63E-04	4.55E-02
negative regulation of cellular biosynthetic process (GO:0031327)	1554	95	58.43	+	1.63	7.45E-06	2.09E-03
negative regulation of macromolecule biosynthetic process (GO:0010558)	1491	91	56.06	+	1.62	1.33E-05	3.32E-03
cellular catabolic process (GO:0044248)	1542	94	57.98	+	1.62	9.55E-06	2.51E-03
neuron differentiation (GO:0030182)	1119	68	42.07	+	1.62	2.19E-04	3.10E-02
regulation of developmental process (GO:0050793)	2509	152	94.33	+	1.61	1.00E-08	1.07E-05
establishment of protein localization (GO:0045184)	1244	75	46.77	+	1.60	1.01E-04	1.73E-02

negative regulation of biosynthetic process (GO:0009890)	1594	96	59.93	+	1.60	9.84E-06	2.54E-03
movement of cell or subcellular component (GO:0006928)	1417	85	53.28	+	1.60	4.19E-05	8.95E-03
regulation of cellular component organization (GO:0051128)	2435	146	91.55	+	1.59	3.40E-08	2.72E-05
negative regulation of cellular macromolecule biosynthetic process (GO:2000113)	1444	86	54.29	+	1.58	4.87E-05	9.63E-03
intracellular signal transduction (GO:0035556)	1397	83	52.52	+	1.58	6.98E-05	1.27E-02
generation of neurons (GO:0048699)	1387	82	52.15	+	1.57	8.98E-05	1.56E-02
negative regulation of nucleic acid-templated transcription (GO:1903507)	1279	75	48.09	+	1.56	2.93E-04	4.01E-02
negative regulation of RNA biosynthetic process (GO:1902679)	1281	75	48.16	+	1.56	2.96E-04	4.01E-02
negative regulation of gene expression (GO:0010629)	1904	111	71.59	+	1.55	8.20E-06	2.26E-03
organic substance catabolic process (GO:1901575)	1539	89	57.86	+	1.54	1.06E-04	1.77E-02
catabolic process (GO:0009056)	1802	104	67.75	+	1.54	2.83E-05	6.67E-03

neurogenesis (GO:0022008)	1540	88	57.90	+	1.52	1.86E-04	2.87E-02
positive regulation of transcription, DNA-templated (GO:0045893)	1566	89	58.88	+	1.51	1.63E-04	2.59E-02
regulation of transport (GO:0051049)	1966	111	73.92	+	1.50	3.34E-05	7.53E-03
negative regulation of nucleobase-containing compound metabolic process (GO:0045934)	1494	84	56.17	+	1.50	3.55E-04	4.55E-02
organic substance transport (GO:0071702)	1836	103	69.03	+	1.49	7.65E-05	1.38E-02
positive regulation of RNA metabolic process (GO:0051254)	1724	96	64.82	+	1.48	1.83E-04	2.84E-02
positive regulation of nucleic acid-templated transcription (GO:1903508)	1625	90	61.10	+	1.47	3.53E-04	4.56E-02
positive regulation of RNA biosynthetic process (GO:1902680)	1626	90	61.13	+	1.47	3.56E-04	4.52E-02
anatomical structure morphogenesis (GO:0009653)	2255	123	84.78	+	1.45	4.60E-05	9.20E-03
positive regulation of nucleobase-containing compound metabolic process (GO:0045935)	1893	103	71.17	+	1.45	2.59E-04	3.61E-02

nervous system development (GO:0007399)	2319	126	87.19	+	1.45	4.42E-05	9.08E-03
regulation of localization (GO:0032879)	2945	160	110.72	+	1.45	2.95E-06	9.46E-04
localization (GO:0051179)	4958	269	186.41	+	1.44	1.09E-10	2.17E-07
positive regulation of nitrogen compound metabolic process (GO:0051173)	3176	172	119.41	+	1.44	1.35E-06	5.41E-04
establishment of localization (GO:0051234)	3756	203	141.22	+	1.44	1.05E-07	7.03E-05
transport (GO:0006810)	3611	193	135.76	+	1.42	5.24E-07	2.33E-04
negative regulation of cellular metabolic process (GO:0031324)	2623	140	98.62	+	1.42	3.41E-05	7.59E-03
cellular protein modification process (GO:0006464)	2553	136	95.99	+	1.42	5.60E-05	1.04E-02
protein modification process (GO:0036211)	2553	136	95.99	+	1.42	5.60E-05	1.03E-02
negative regulation of nitrogen compound metabolic process (GO:0051172)	2422	129	91.06	+	1.42	7.89E-05	1.40E-02
positive regulation of macromolecule metabolic process (GO:0010604)	3534	187	132.87	+	1.41	1.47E-06	5.73E-04
positive regulation of cellular metabolic process (GO:0031325)	3375	178	126.89	+	1.40	3.94E-06	1.19E-03

negative regulation of cellular process (GO:0048523)	4852	255	182.42	+	1.40	9.99E-09	1.14E-05
cell differentiation (GO:0030154)	3397	178	127.72	+	1.39	5.36E-06	1.56E-03
organelle organization (GO:0006996)	3243	169	121.93	+	1.39	1.43E-05	3.53E-03
regulation of transcription by RNA polymerase II (GO:0006357)	2342	122	88.05	+	1.39	3.13E-04	4.17E-02
cellular developmental process (GO:0048869)	3447	179	129.60	+	1.38	9.45E-06	2.52E-03
negative regulation of metabolic process (GO:0009892)	3044	158	114.45	+	1.38	4.17E-05	9.03E-03
response to organic substance (GO:0010033)	3263	168	122.68	+	1.37	2.94E-05	6.82E-03
macromolecule modification (GO:0043412)	2743	141	103.13	+	1.37	1.80E-04	2.83E-02
cellular component organization (GO:0016043)	5159	265	193.97	+	1.37	3.30E-08	2.78E-05
regulation of signaling (GO:0023051)	3237	166	121.70	+	1.36	4.25E-05	8.85E-03
positive regulation of cellular process (GO:0048522)	5738	294	215.73	+	1.36	3.61E-09	5.26E-06
negative regulation of macromolecule metabolic process (GO:0010605)	2794	143	105.05	+	1.36	2.02E-04	2.94E-02

regulation of cell communication (GO:0010646)	3225	165	121.25	+	1.36	5.13E-05	9.90E-03
positive regulation of metabolic process (GO:0009893)	3820	195	143.62	+	1.36	9.03E-06	2.45E-03
regulation of biological quality (GO:0065008)	4007	204	150.65	+	1.35	5.62E-06	1.61E-03
cellular macromolecule metabolic process (GO:0044260)	4362	222	164.00	+	1.35	1.63E-06	6.06E-04
positive regulation of biological process (GO:0048518)	6198	315	233.03	+	1.35	1.52E-09	2.44E-06
regulation of transcription, DNA-templated (GO:0006355)	3118	158	117.23	+	1.35	1.43E-04	2.31E-02
regulation of macromolecule metabolic process (GO:0060255)	6088	307	228.89	+	1.34	6.84E-09	8.43E-06
negative regulation of biological process (GO:0048519)	5297	267	199.15	+	1.34	1.72E-07	8.62E-05
cellular component organization or biogenesis (GO:0071840)	5382	271	202.35	+	1.34	1.28E-07	6.86E-05
regulation of nucleic acid-templated transcription (GO:1903506)	3167	159	119.07	+	1.34	1.93E-04	2.89E-02

regulation of nucleobase-containing compound metabolic process (GO:0019219)	3685	185	138.55	+	1.34	4.58E-05	9.29E-03
regulation of RNA biosynthetic process (GO:2001141)	3172	159	119.26	+	1.33	1.96E-04	2.91E-02
regulation of primary metabolic process (GO:0080090)	5716	286	214.91	+	1.33	7.43E-08	5.41E-05
regulation of RNA metabolic process (GO:0051252)	3438	172	129.26	+	1.33	1.18E-04	1.93E-02
regulation of cellular macromolecule biosynthetic process (GO:2000112)	3569	178	134.19	+	1.33	1.01E-04	1.72E-02
regulation of nitrogen compound metabolic process (GO:0051171)	5556	277	208.89	+	1.33	2.15E-07	9.84E-05
regulation of macromolecule biosynthetic process (GO:0010556)	3654	181	137.38	+	1.32	1.17E-04	1.94E-02
regulation of cellular metabolic process (GO:0031323)	5917	293	222.46	+	1.32	1.27E-07	7.01E-05
anatomical structure development (GO:0048856)	5286	261	198.74	+	1.31	1.47E-06	5.62E-04
regulation of metabolic process (GO:0019222)	6552	323	246.34	+	1.31	2.22E-08	1.98E-05
regulation of gene expression (GO:0010468)	4470	220	168.06	+	1.31	1.96E-05	4.77E-03

macromolecule metabolic process (GO:0043170)	5649	277	212.39	+	1.30	8.97E-07	3.78E-04
regulation of biosynthetic process (GO:0009889)	3904	191	146.78	+	1.30	1.45E-04	2.32E-02
developmental process (GO:0032502)	5713	278	214.79	+	1.29	1.80E-06	6.54E-04
regulation of cellular biosynthetic process (GO:0031326)	3826	186	143.85	+	1.29	2.27E-04	3.19E-02
multicellular organism development (GO:0007275)	4915	237	184.79	+	1.28	3.08E-05	7.04E-03
cellular metabolic process (GO:0044237)	6893	332	259.16	+	1.28	1.53E-07	7.90E-05
protein metabolic process (GO:0019538)	3929	189	147.72	+	1.28	3.73E-04	4.56E-02
organic substance metabolic process (GO:0071704)	7304	349	274.61	+	1.27	1.25E-07	7.13E-05
organonitrogen compound metabolic process (GO:1901564)	4859	231	182.69	+	1.26	1.03E-04	1.73E-02
metabolic process (GO:0008152)	7856	371	295.37	+	1.26	1.09E-07	6.98E-05
primary metabolic process (GO:0044238)	6804	321	255.81	+	1.25	2.28E-06	7.77E-04
nitrogen compound metabolic process (GO:0006807)	6266	295	235.59	+	1.25	1.06E-05	2.70E-03
cellular process (GO:0009987)	15509	653	583.10	+	1.12	3.97E-08	3.03E-05

biological_process (GO:0008150)	18114	734	681.04	+	1.08	1.99E-07	9.39E-05
Unclassified (UNCLASSIFIED)	3563	81	133.96	-	.60	1.99E-07	9.68E-05
nervous system process (GO:0050877)	2200	50	82.71	-	.60	8.29E-05	1.46E-02
G protein-coupled receptor signaling pathway (GO:0007186)	2088	41	78.50	-	.52	2.42E-06	8.07E-04
sensory perception (GO:0007600)	1721	24	64.71	-	.37	6.81E-09	9.09E-06
detection of stimulus (GO:0051606)	1427	10	53.65	-	.19	3.27E-13	7.48E-10
detection of chemical stimulus (GO:0009593)	1275	5	47.94	-	.10	4.81E-15	1.28E-11
sensory perception of chemical stimulus (GO:0007606)	1315	3	49.44	-	.06	6.87E-18	2.75E-14
detection of stimulus involved in sensory perception (GO:0050906)	1324	3	49.78	-	.06	7.42E-18	2.38E-14
sensory perception of smell (GO:0007608)	1230	1	46.24	-	.02	6.02E-19	3.22E-15
detection of chemical stimulus involved in sensory perception (GO:0050907)	1241	1	46.66	-	.02	4.01E-19	3.21E-15
detection of chemical stimulus involved in sensory perception of smell (GO:0050911)	1184	0	44.52	-	< 0.01	4.93E-20	7.89E-16

Suplemmentary material
Manuscript ID 37b7d554-82c2-48e4-bcff-08125556c418
A database on differentially expressed microRNAs during rodent bladder healing"

Supplementary table S9

The table shows the most significant Pathways represented in the list of target genes for the DE microRNAS at 6h

Pathway name	Entities				Reactions	
	found	ratio	p-value	FDR*	found	ratio
PTK6 Regulates Proteins Involved in RNA Processing	3 / 7	4.81e-04	0.008	0.683	5 / 7	5.18e-04
Transcriptional Regulation by E2F6	7 / 46	0.003	0.018	0.683	13 / 33	0.002
Nuclear signaling by ERBB4	7 / 47	0.003	0.02	0.683	7 / 34	0.003
Lysosphingolipid and LPA receptors	4 / 19	0.001	0.025	0.683	4 / 5	3.70e-04
Nef mediated downregulation of MHC class I complex cell surface expression	3 / 11	7.56e-04	0.026	0.683	3 / 4	2.96e-04
Nuclear Receptor transcription pathway	10 / 86	0.006	0.029	0.683	2 / 2	1.48e-04
Nef-mediates down modulation of cell surface receptors by recruiting them to clathrin adapters	4 / 22	0.002	0.039	0.683	6 / 15	0.001

G1/S-Specific Transcription	6 / 43	0.003	0.04	0.683	7 / 28	0.002
PI3K/AKT activation	3 / 13	8.94e-04	0.04	0.683	4 / 5	3.70e-04
Dissolution of Fibrin Clot	3 / 14	9.63e-04	0.048	0.683	8 / 19	0.001
MAP kinase activation	8 / 69	0.005	0.048	0.683	15 / 32	0.002
Signaling by ERBB4	9 / 82	0.006	0.05	0.683	19 / 52	0.004
MAPK targets/ Nuclear events mediated by MAP kinases	5 / 35	0.002	0.053	0.683	9 / 16	0.001
Drug resistance of PDGFR mutants	1 / 1	6.88e-05	0.056	0.683	4 / 4	2.96e-04
PDGFR mutants bind TKIs	1 / 1	6.88e-05	0.056	0.683	2 / 2	1.48e-04
Regorafenib-resistant PDGFR mutants	1 / 1	6.88e-05	0.056	0.683	1 / 1	7.40e-05
Imatinib-resistant PDGFR mutants	1 / 1	6.88e-05	0.056	0.683	1 / 1	7.40e-05
Sorafenib-resistant PDGFR mutants	1 / 1	6.88e-05	0.056	0.683	1 / 1	7.40e-05
Sunitinib-resistant PDGFR mutants	1 / 1	6.88e-05	0.056	0.683	1 / 1	7.40e-05
Regulation of gene expression by Hypoxia-inducible Factor	3 / 15	0.001	0.057	0.683	7 / 7	5.18e-04
ERKs are inactivated	3 / 15	0.001	0.057	0.683	2 / 2	1.48e-04
Potential therapeutics for SARS	9 / 84	0.006	0.057	0.683	7 / 32	0.002
VxPx cargo-targeting to cilium	4 / 25	0.002	0.058	0.683	10 / 10	7.40e-04

RHOBTB GTPase Cycle	5 / 36	0.002	0.059	0.683	3 / 4	2.96e-04
ERK/MAPK targets	4 / 26	0.002	0.065	0.683	5 / 7	5.18e-04

* False Discovery Rate

https:// reactome.org

Supplementary table S10

The table shows the most significant Pathways represented in the list of target genes for the DE microRNAS at 2 days

Pathway name	Entities				Reactions	
	found	ratio	p-value	FDR*	found	ratio
Transcriptional Regulation by E2F6	8 / 46	0.003	7.04e-04	0.624	14 / 33	0.002
Nuclear Receptor transcription pathway	10 / 86	0.006	0.003	0.624	2 / 2	1.48e-04
RAB geranylgeranylation	8 / 68	0.005	0.007	0.624	3 / 5	3.70e-04
G1/S-Specific Transcription	6 / 43	0.003	0.009	0.624	7 / 28	0.002
Class I peroxisomal membrane protein import	4 / 20	0.001	0.01	0.624	6 / 6	4.44e-04
PI3K/AKT activation	3 / 13	8.94e-04	0.017	0.624	4 / 5	3.70e-04
G0 and Early G1	5 / 38	0.003	0.021	0.624	18 / 27	0.002
G1/S Transition	12 / 150	0.01	0.022	0.624	31 / 61	0.005
ERKs are inactivated	3 / 15	0.001	0.024	0.624	2 / 2	1.48e-04
Mitotic G1 phase and G1/S transition	13 / 173	0.012	0.027	0.624	58 / 99	0.007
Erythropoietin activates Phosphoinositide-3-kinase (PI3K)	3 / 16	0.001	0.028	0.624	7 / 7	5.18e-04
PTK6 Regulates Proteins Involved in RNA Processing	2 / 7	4.81e-04	0.034	0.624	2 / 7	5.18e-04
Drug resistance of PDGFR mutants	1 / 1	6.88e-05	0.04	0.624	4 / 4	2.96e-04
PDGFR mutants bind TKIs	1 / 1	6.88e-05	0.04	0.624	2 / 2	1.48e-04
Imatinib-resistant PDGFR mutants	1 / 1	6.88e-05	0.04	0.624	1 / 1	7.40e-05
Sorafenib-resistant PDGFR mutants	1 / 1	6.88e-05	0.04	0.624	1 / 1	7.40e-05
Regorafenib-resistant PDGFR mutants	1 / 1	6.88e-05	0.04	0.624	1 / 1	7.40e-05
Sunitinib-resistant PDGFR mutants	1 / 1	6.88e-05	0.04	0.624	1 / 1	7.40e-05
Signaling by MAPK mutants	2 / 8	5.50e-04	0.043	0.624	1 / 1	7.40e-05
Spry regulation of FGF signaling	3 / 19	0.001	0.044	0.624	11 / 11	8.14e-04
Signaling by PDGFRA transmembrane, juxtamembrane and kinase domain mutants	3 / 19	0.001	0.044	0.624	7 / 7	5.18e-04
Signaling by PDGFRA extracellular domain mutants	3 / 19	0.001	0.044	0.624	7 / 7	5.18e-04
Tristetraprolin (TTP, ZFP36) binds and destabilizes mRNA	3 / 19	0.001	0.044	0.624	4 / 4	2.96e-04
CREB3 factors activate genes	2 / 9	6.19e-04	0.053	0.624	5 / 21	0.002
Passive transport by Aquaporins	3 / 21	0.001	0.056	0.624	6 / 8	5.92e-04

* False Discovery Rate

Supplementary table S11

The table shows the most significant Pathways represented in the list of target genes for the DE microRNAS at 8 days

Pathway name	Entities				Reactions	
	found	ratio	p-value	FDR*	found	ratio
Nuclear Receptor transcription pathway	11 / 86	0.006	1.01e-04	0.117	2 / 2	1.48e-04
CREB3 factors activate genes	3 / 9	6.19e-04	0.003	0.573	9 / 21	0.002
Activation of RAC1	3 / 15	0.001	0.012	0.573	4 / 4	2.96e-04
RUNX3 regulates NOTCH signaling	3 / 16	0.001	0.014	0.573	7 / 7	5.18e-04
Signaling by MAP2K mutants	2 / 6	4.13e-04	0.015	0.573	1 / 1	7.40e-05
Golgi Cisternae Pericentriolar Stack Reorganization	3 / 17	0.001	0.016	0.573	6 / 6	4.44e-04
Golgi-to-ER retrograde transport	10 / 148	0.01	0.019	0.573	13 / 18	0.001
Negative feedback regulation of MAPK pathway	2 / 8	5.50e-04	0.026	0.573	3 / 3	2.22e-04
Defective F9 secretion	1 / 1	6.88e-05	0.031	0.573	1 / 1	7.40e-05
Transport of gamma-carboxylated protein precursors from the endoplasmic reticulum to the Golgi apparatus	2 / 9	6.19e-04	0.032	0.573	2 / 9	6.66e-04
Signaling by cytosolic FGFR1 fusion mutants	3 / 23	0.002	0.036	0.573	8 / 14	0.001
Activated NTRK3 signals through PI3K	2 / 10	6.88e-04	0.039	0.573	5 / 5	3.70e-04
PTK6 Expression	2 / 10	6.88e-04	0.039	0.573	3 / 3	2.22e-04
Mitochondrial ABC transporters	2 / 10	6.88e-04	0.039	0.573	2 / 2	1.48e-04
RUNX1 regulates estrogen receptor mediated transcription	2 / 10	6.88e-04	0.039	0.573	2 / 8	5.92e-04

Removal of aminoterminal propeptides from gamma-carboxylated proteins	2 / 10	6.88e-04	0.039	0.573	2 / 9	6.66e-04
Intra-Golgi and retrograde Golgi-to-ER traffic	12 / 218	0.015	0.042	0.573	22 / 48	0.004
Calcitonin-like ligand receptors	2 / 11	7.56e-04	0.046	0.573	3 / 4	2.96e-04
COPI-mediated anterograde transport	7 / 107	0.007	0.052	0.573	12 / 12	8.88e-04
COPI-dependent Golgi-to-ER retrograde traffic	7 / 107	0.007	0.052	0.573	9 / 11	8.14e-04
Activation of the AP-1 family of transcription factors	2 / 12	8.25e-04	0.054	0.573	3 / 5	3.70e-04
Defective BTD causes biotidinase deficiency	1 / 2	1.38e-04	0.06	0.573	2 / 2	1.48e-04

	Differential expression				
	**6hw vs ctl**	**2dw vs ctl**	**8dw vs ctl**	**6hw vs 8dw**	**2dw vs 8dw**
Number of miRNA	10	5	6	4	4
Targets of differentially expressed	2042	888	1204	1259	1102
