# Supplementary material for: A database on differentially expressed microRNAs during rodent bladder healing
Source: Sci Rep. 2021 Nov 8;11:21881. doi: 10.1038/s41598-021-01413-0 (PMC8575992; doi:10.1038/s41598-021-01413-0)
Supplement: Supplementary file 5 — Supplementary Information 5. [file 41598_2021_1413_MOESM5_ESM.docx]

**References for Table 2. Literature review of the functions associated with DE microRNAS**

1 Wu, P., Cao, Y., Zhao, R. & Wang, Y. miR-96-5p regulates wound healing by targeting BNIP3/FAK pathway. *J Cell Biochem* **120**, 12904-12911, doi:10.1002/jcb.28561 (2019).

2 Xue, M., Thompson, P., Kelso, I. & Jackson, C. Activated protein C stimulates proliferation, migration and wound closure, inhibits apoptosis and upregulates MMP-2 activity in cultured human keratinocytes. *Exp Cell Res* **299**, 119-127, doi:10.1016/j.yexcr.2004.05.015 (2004).

3 Huang, J. *et al.* miR-92b targets DAB2IP to promote EMT in bladder cancer migration and invasion. *Oncol Rep* **36**, 1693-1701, doi:10.3892/or.2016.4940 (2016).

4 Gallant-Behm, C. L. *et al.* A synthetic microRNA-92a inhibitor (MRG-110) accelerates angiogenesis and wound healing in diabetic and nondiabetic wounds. *Wound Repair Regen* **26**, 311-323, doi:10.1111/wrr.12660 (2018).

5 Singh, J. *et al.* Role of differentially expressed microRNA-139-5p in the regulation of phenotypic internal anal sphincter smooth muscle tone. *Sci Rep* **7**, 1477, doi:10.1038/s41598-017-01550-5 (2017).

6 Sun, C. *et al.* Hsa-miR-139-5p inhibits proliferation and causes apoptosis associated with down-regulation of c-Met. *Oncotarget* **6**, 39756-39792, doi:10.18632/oncotarget.5476 (2015).

7 Zhang, H., Sun, Z., Yu, L. & Sun, J. MiR-139-5p inhibits proliferation and promoted apoptosis of human airway smooth muscle cells by downregulating the Brg1 gene. *Respir Physiol Neurobiol* **246**, 9-16, doi:10.1016/j.resp.2017.07.004 (2017).

8 Khalili, N. *et al.* Diagnostic, prognostic, and therapeutic significance of miR-139-5p in cancers. *Life Sci* **256**, 117865, doi:10.1016/j.lfs.2020.117865 (2020).

9 Yonemori, M. *et al.* Dual tumor-suppressors miR-139-5p and miR-139-3p targeting matrix metalloprotease 11 in bladder cancer. *Cancer Sci* **107**, 1233-1242, doi:10.1111/cas.13002 (2016).

10 Wang, N. *et al.* miR-141-3p suppresses proliferation and promotes apoptosis by targeting GLI2 in osteosarcoma cells. *Oncol Rep* **39**, 747-754, doi:10.3892/or.2017.6150 (2018).

11 Feng, J., Xue, S., Pang, Q., Rang, Z. & Cui, F. miR-141-3p inhibits fibroblast proliferation and migration by targeting GAB1 in keloids. *Biochem Biophys Res Commun* **490**, 302-308, doi:10.1016/j.bbrc.2017.06.040 (2017).

12 Ghorbanmehr, N. *et al.* miR-21-5p, miR-141-3p, and miR-205-5p levels in urine-promising biomarkers for the identification of prostate and bladder cancer. *Prostate* **79**, 88-95, doi:10.1002/pros.23714 (2019).

13 Zhang, C., Kong, X. & Ma, D. miR-141-3p inhibits vascular smooth muscle cell proliferation and migration via regulating Keap1/Nrf2/HO-1 pathway. *IUBMB Life* **72**, 2167-2179, doi:10.1002/iub.2374 (2020).

14 Dong, N. *et al.* MicroRNA-182 prevents vascular smooth muscle cell dedifferentiation via FGF9/PDGFRbeta signaling. *Int J Mol Med* **39**, 791-798, doi:10.3892/ijmm.2017.2905 (2017).

15 Wang, T. *et al.* Identification of microRNAome in rat bladder reveals miR-1949 as a potential inducer of bladder cancer following spinal cord injury. *Mol Med Rep* **12**, 2849-2857, doi:10.3892/mmr.2015.3769 (2015).

16 Huynh, C. *et al.* Efficient in vivo microRNA targeting of liver metastasis. *Oncogene* **30**, 1481-1488, doi:10.1038/onc.2010.523 (2011).

17 Aunin, E., Broadley, D., Ahmed, M. I., Mardaryev, A. N. & Botchkareva, N. V. Exploring a Role for Regulatory miRNAs In Wound Healing during Ageing:Involvement of miR-200c in wound repair. *Sci Rep* **7**, 3257, doi:10.1038/s41598-017-03331-6 (2017).

18 Tian, Y. *et al.* MicroRNA-200 (miR-200) cluster regulation by achaete scute-like 2 (Ascl2): impact on the epithelial-mesenchymal transition in colon cancer cells. *J Biol Chem* **289**, 36101-36115, doi:10.1074/jbc.M114.598383 (2014).

19 Luo, G. *et al.* DNA Methylation Regulates Corneal Epithelial Wound Healing by Targeting miR-200a and CDKN2B. *Invest Ophthalmol Vis Sci* **60**, 650-660, doi:10.1167/iovs.18-25443 (2019).

20 Chan, Y. C., Roy, S., Khanna, S. & Sen, C. K. Downregulation of endothelial microRNA-200b supports cutaneous wound angiogenesis by desilencing GATA binding protein 2 and vascular endothelial growth factor receptor 2. *Arterioscler Thromb Vasc Biol* **32**, 1372-1382, doi:10.1161/ATVBAHA.112.248583 (2012).

21 Sinha, M., Ghatak, S., Roy, S. & Sen, C. K. microRNA-200b as a Switch for Inducible Adult Angiogenesis. *Antioxid Redox Signal* **22**, 1257-1272, doi:10.1089/ars.2014.6065 (2015).

22 Zhou, Z. *et al.* microRNA-203 Modulates Wound Healing and Scar Formation via Suppressing Hes1 Expression in Epidermal Stem Cells. *Cell Physiol Biochem* **49**, 2333-2347, doi:10.1159/000493834 (2018).

23 Jackson, S. J. *et al.* Rapid and widespread suppression of self-renewal by microRNA-203 during epidermal differentiation. *Development* **140**, 1882-1891, doi:10.1242/dev.089649 (2013).

24 Luo, Z. *et al.* miR-203a-3p promotes loureirin A-induced hair follicle stem cells differentiation by targeting Smad1. *Anat Rec (Hoboken)*, doi:10.1002/ar.24480 (2020).

25 Teng, J. W., Ji, P. F. & Zhao, Z. G. MiR-214-3p inhibits beta-catenin signaling pathway leading to delayed fracture healing. *Eur Rev Med Pharmacol Sci* **22**, 17-24, doi:10.26355/eurrev_201801_14095 (2018).

26 Zhou, L. G. *et al.* MiR-214-3p delays fracture healing in rats with osteoporotic fracture through inhibiting BMP/Smad signaling pathway. *Eur Rev Med Pharmacol Sci* **23**, 449-455, doi:10.26355/eurrev_201901_16854 (2019).

27 Yang, K., Shi, J., Hu, Z. & Hu, X. The deficiency of miR-214-3p exacerbates cardiac fibrosis via miR-214-3p/NLRC5 axis. *Clin Sci (Lond)* **133**, 1845-1856, doi:10.1042/CS20190203 (2019).

28 Sun, Y. *et al.* MiR-214 is an important regulator of the musculoskeletal metabolism and disease. *J Cell Physiol* **234**, 231-245, doi:10.1002/jcp.26856 (2018).

29 Mitash, N., Tiwari, S., Agnihotri, S. & Mandhani, A. Bladder cancer: Micro RNAs as biomolecules for prognostication and surveillance. *Indian J Urol* **33**, 127-133, doi:10.4103/0970-1591.203412 (2017).

30 Lino Cardenas, C. L. *et al.* miR-199a-5p Is upregulated during fibrogenic response to tissue injury and mediates TGFbeta-induced lung fibroblast activation by targeting caveolin-1. *PLoS Genet* **9**, e1003291, doi:10.1371/journal.pgen.1003291 (2013).

31 Xu, H. *et al.* Methylation-mediated miR-214 regulates proliferation and drug sensitivity of renal cell carcinoma cells through targeting LIVIN. *J Cell Mol Med* **24**, 6410-6425, doi:10.1111/jcmm.15287 (2020).

32 Yao, Y. *et al.* miR-297 Protects Human Umbilical Vein Endothelial Cells against LPS-Induced Inflammatory Response and Apoptosis. *Cell Physiol Biochem* **52**, 696-707, doi:10.33594/000000049 (2019).

33 Li, H., Guan, S. B., Lu, Y. & Wang, F. MiR-140-5p inhibits synovial fibroblasts proliferation and inflammatory cytokines secretion through targeting TLR4. *Biomed Pharmacother* **96**, 208-214, doi:10.1016/j.biopha.2017.09.079 (2017).

34 Wang, H. *et al.* miR-219 Cooperates with miR-338 in Myelination and Promotes Myelin Repair in the CNS. *Dev Cell* **40**, 566-582 e565, doi:10.1016/j.devcel.2017.03.001 (2017).

35 Jiang, F. *et al.* MicroRNA-421 promotes inflammatory response of fibroblast-like synoviocytes in rheumatoid arthritis by targeting SPRY1. *Eur Rev Med Pharmacol Sci* **23**, 8186-8193, doi:10.26355/eurrev_201910_19125 (2019).

36 Fang, Y. *et al.* Burkholderia pseudomallei-derived miR-3473 enhances NF-kappaB via targeting TRAF3 and is associated with different inflammatory responses compared to Burkholderia thailandensis in murine macrophages. *BMC Microbiol* **16**, 283, doi:10.1186/s12866-016-0901-6 (2016).

37 Kagawa, T. *et al.* A scrutiny of circulating microRNA biomarkers for drug-induced tubular and glomerular injury in rats. *Toxicology* **415**, 26-36, doi:10.1016/j.tox.2019.01.011 (2019).

38 Yu, L. *et al.* Dysregulation of renal microRNA expression after deep hypothermic circulatory arrest in rats. *Eur J Cardiothorac Surg* **49**, 1725-1731, doi:10.1093/ejcts/ezv460 (2016).

39 Meng, F., Hackenberg, M., Li, Z., Yan, J. & Chen, T. Discovery of novel microRNAs in rat kidney using next generation sequencing and microarray validation. *PLoS One* **7**, e34394, doi:10.1371/journal.pone.0034394 (2012).

40 Tian, H., Wang, X., Lu, J., Tian, W. & Chen, P. MicroRNA-621 inhibits cell proliferation and metastasis in bladder cancer by suppressing Wnt/beta-catenin signaling. *Chem Biol Interact* **308**, 244-251, doi:10.1016/j.cbi.2019.05.042 (2019).
